# Synergistic Fe/Ni catalysis for electrochemical 1,1-difunctionalization of alkenes

**DOI:** 10.1038/s41467-026-72546-x

**Published:** 2026-05-02

**Authors:** Pengwei Hu, Chao Yang, Lin Guo, Wujiong Xia

**Affiliations:** 1 State Key Lab of Urban Water Resource and Environment, Harbin Institute of Technology (Shenzhen), Shenzhen, China; 2 School of Chemistry and Chemical Engineering, Henan Normal University, Xinxiang, Henan China

**Keywords:** Electrocatalysis, Synthetic chemistry methodology

## Abstract

Alkene 1,1-difunctionalization holds significant importance in organic synthesis due to its ability to effectively enhance the complexity and functionality of molecular frameworks. Herein, we report an electrochemical strategy for 1,1-difunctionalization of halogenated aromatics with unactivated alkenes using synergistic Fe/Ni catalysis. This system integrates redox activity of nickel with Lewis acid functionality of iron: the nickel catalyst governs aryl halide oxidative addition and alkene migration, while iron species activates catalytic sites, stabilizes radical acceptors, and precisely regulates electrochemical reduction sequences/selectivities. The reaction system is applicable to a wide range of substrates, including electron-rich and electron-deficient aryl halides, polycyclic compounds, and bioactive natural products (100 examples). Gram-scale synthesis maintains 63% yield, supporting industrial viability. Mechanistic studies elucidate the unique cooperativity of this iron-nickel bimetallic system, providing a theoretical framework for the design of diverse difunctionalization reactions.

## Abstract (web summary)

Alkene 1,1- difunctionalization holds significant importance in organic synthesis due to its ability to effectively enhance the complexity and functionality of molecular frameworks. Here, the authors report an electrochemical strategy for 1,1-difunctionalization of halogenated aromatics with unactivated alkenes, using synergistic Fe/Ni catalysis.

## Introduction

**Fig. 1:**
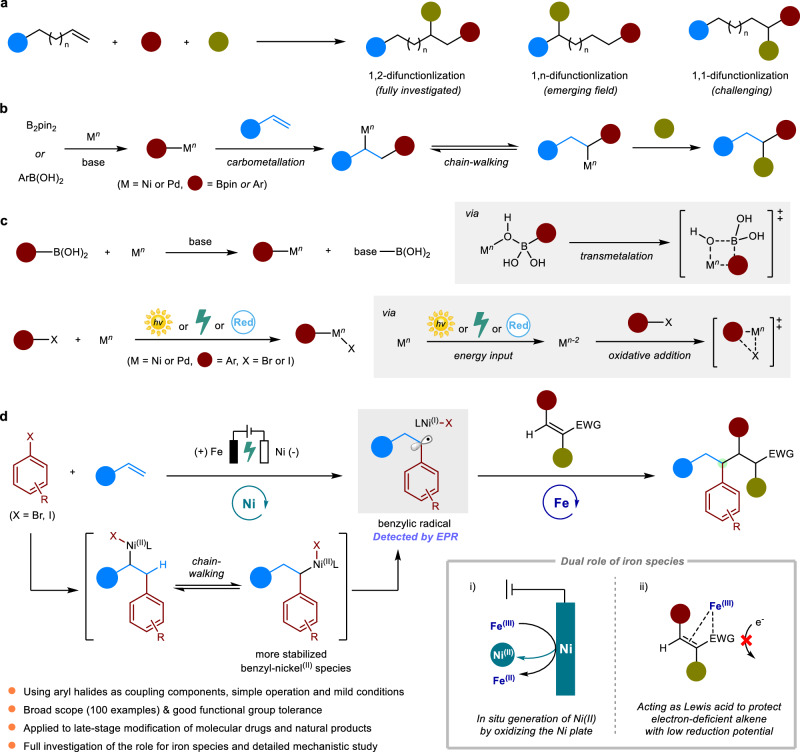
Inspiration and design for alkene 1,1-difunctionalization. **a** Three types of alkene difunctionalization reactions. **b** Previously reported transition-metal-catalyzed alkene 1,1-difunctionalization reactions. **c** Transmetalation and redox processes. **d** Our reaction design.

Alkenes are indispensable molecular building blocks in organic synthesis, and their difunctionalization offers an efficient strategy for modular construction of complex molecules^1–3^. Traditional research has mainly focused on alkene 1,2-difunctionalization, which has enabled a wealth of organic transformations via transition metal catalysis, radical-mediated approaches, and others^4–9^. Recent years have witnessed significant advances in remote alkene difunctionalization through chain-walking processes (1,n-difunctionalization, *n* ≥ 3), providing a new paradigm for precisely controlling the regioselectivity^10–13^. In contrast, alkene 1,1-difunctionalization, where two different functional groups install onto the same carbon atom, still faces challenges. Thermodynamic/kinetic competition and the uncontrollable nature of transient intermediates hinder the regioselectivity and stereoselectivity control in this type of transformations, while also complicating precise mechanistic analysis (Fig. 1a)^14–16^.

Current alkene 1,1-difunctionalization reactions primarily rely on the use of transition metal catalysts (mainly nickel and palladium) and organoboron reagents (such as B_2_pin_2_ and arylboronic acids)^17–27^. Key steps involve in situ formation of aryl- or boryl-metal species, followed by carbometallation and chain-walking processes (Fig. 1b). A representative contribution in this area was made by Yin and co-workers for the development of Ni-catalyzed 1,1-difunctionalization of unactivated alkenes with arylboronic acids^17^. While effective for constructing C − B and C−Ar bonds in multi-component reactions, these strategies face limitations: the high cost of organoboron reagents, air/moisture sensitivity, and restricted substrate versatility of arylboronic acids hinder industrial applications^28,29^. Given the prevalence of 1,1-alkylarene motifs in bioactive and pharmaceutical molecules^30,31^, the development of cheap, stable, and diverse arylation reagents to replace arylboronic acids is crucial for advancing this field. Aryl halides serve as a complementary aryl source to arylboronic acids. Although they proceed through a divergent mechanistic pathway involving oxidative addition, they offer notable practical advantages including lower cost, enhanced stability, and greater structural diversity compared to their boronic acid counterparts^32–34^. However, their application in alkene 1,1-difunctionalization faces two bottlenecks: (1) Compared to the mild transmetalation pathway of C(aryl)−B bonds, C(aryl)−X (X = Cl, Br, I) bonds require overcoming a higher activation barrier for oxidative addition (Fig. 1c)^35–37^. This compels reliance on the addition of reducing agents or external energy input, thus increasing reaction complexity^38–41^. (2) Despite their intrinsic stability, aryl halides remain susceptible to side reactions such as dehalogenation and homocoupling, thereby reducing their efficiency in organic transformations^42–44^. The emergence of electrocatalysis offers alternative solutions to these challenges by directly driving electron transfer through precise control of electrode potential and current density, eliminating dependence on chemical reductants. Compared to traditional methods, organic electrocatalysis not only avoids side reactions and environmental pollution caused by overstoichiometric reductants, but also enables dynamic tuning of kinetic parameters, significantly enhancing product selectivity and atom economy^45–48^. This precise control offers unique opportunities for innovating transition metal catalytic systems. Leveraging electrochemically driven redox cycles, metal catalysts can rapidly interconvert between oxidation states, enabling continuous regeneration of active catalytic centers^40,47,49–51^. Consequently, the integration of organic electrochemistry with transition metal catalysis would overcome inherent reactivity and selectivity constraints, unlocking the synthetic potential of aryl halides in alkene 1,1-difunctionalization reaction.

In this work, we propose an electrochemical strategy for alkene 1,1-difunctionalization with aryl halides using Fe-Ni bimetallic synergistic catalysis. This approach integrates the redox activity of nickel with Lewis acid functionality of iron, facilitating a multi-component reaction of aryl halides, unactivated alkenes and electron-deficient alkenes (Fig. 1d). The reaction commences with the addition of aryl halides to unactivated alkenes, forming alkyl-nickel(II) species, which then undergo a chain-walking process to generate more stabilized benzyl-nickel(II) intermediates^17^. Subsequent carbon-nickel bond homolysis releases Ni(I) species and benzylic radicals, which could undergo radical addition with electron-deficient alkenes (acting as radical acceptors) in the presence of iron catalyst to form the final 1,1-difunctionalization products. In our proposed approach, the nickel catalyst regulates oxidative addition of aryl halides and carbometallation of alkenes, while Fe(III) species activates catalytic sites and stabilizes the electron-deficient alkenes bearing low reduction potentials, precisely regulating electrochemical reduction sequence as well as regioselectivity. Key advantages of this strategy include: (1) excellent substrate generality toward halogenated arenes with electron-rich/deficient substituents and polycyclic/heterocyclic frameworks; (2) oxidant/reductant-free reaction conditions; (3) gram-scale applicability and late-stage modification of pharmaceuticals/natural products, demonstrating significant industrial potential. More intriguingly, the dual role of iron species has been systematically studied. When using a nickel plate cathode, the addition of iron catalyst directly oxidizes the nickel electrode to generate homogeneous high-valent nickel species in situ, enabling an efficient reaction without requiring additional nickel catalysts. Another function of iron catalysts is to serve as effective Lewis acids, overcoming the low redox potentials of electron-deficient alkenes and suppressing disproportionation/coupling side reactions^52–55^. This reaction process reveals the unique catalytic mechanism of the Fe-Ni bimetallic system, providing a theoretical foundation for rational design of such 1,1-difunctionalization reactions. The work not only breaks away from the previous boron-reagent-dependent paradigm but establishes an efficient, sustainable platform for inert alkene functionalization and late-stage pharmaceutical modification through synergistic electrochemistry and bimetallic catalysis.

## Results

### Optimization Studies

As shown in Table 1, our initial investigation focused on the electrochemical three-component reaction of 4-iodobiphenyl (**a-1**), dimethylphenylvinylsilane (**b-1**), and dimethyl fumarate (**c-1**), employing an iron plate as the anode and a nickel plate as the cathode. Under the optimized electrochemical conditions using nBu4NBF4 as the electrolyte, FeCl3 as the catalyst, 2,2,6,6-tetramethyl-3,5-heptanedione (TMHD, **L2**) as the supporting ligand in a DMSO/1,4-dioxane mixed solvent system, the electrochemical system demonstrated good reactivity and excellent regioselectivity control, affording 1,1-difunctionalized product **d-1** in 85% yield with a diastereomeric ratio (d.r.) of 1.3:1 (entry 1). Control experiments confirmed that the use of electric current and Fe(III) catalyst are both essential for the desired transformation (entries 2 and 3). Intriguingly, the reaction could smoothly proceed in the absence of a nickel catalyst, if a nickel plate was utilized as the cathode electrode. The use of Fe(II) species instead of Fe(III) failed to afford the desired product (entry 4). Notably, removal of TMHD severely affected the yield of **d-1** (entry 5), while conventional bipyridine-type ligands such as 6,6’-dimethyl-2,2’-bipyridine (DMBP, **L1**) completely inhibited the difunctionalization process (entry 6). Compared to the absence of ligand, the use of other types of β-dicarbonyl ligands (**L3-L7**) was found to effectively enhance the reaction efficiency (entry 7). We propose that TMHD facilitates the catalytic cycle through dual mechanisms: firstly, its anionic O,O-chelating character finely tunes the electron density of the nickel center, optimizing the reduction potential of the Ni(II)/Ni(I) couple; secondly, its moderate coordination strength stabilizes the alkyl-nickel intermediates during chain-walking without excessively inhibiting the subsequent C–Ni bond homolysis step. In contrast, rigid bidentate nitrogen ligands may form excessively stable nickel complexes that impede the crucial migratory insertion or radical generation steps^56^. Furthermore, substitution of the mixed solvent system with DMSO reduced the yield to 52% (entry 8). Electrode pairing studies indicated that replacing the iron plate anode by aluminum plate in the absence of FeCl3 resulted in complete suppression of product formation (entry 9). However, exogenous nickel supplementation restored the formation of **d-1** (32% yield, entry 10), implicating Fe(III) species in dual roles: oxidation of the nickel plate cathode to generate Ni(II), and Lewis acid-mediated activation of the substrates. Intriguingly, replacement of the Ni cathode with an iron plate entirely abolished product formation (entry 11), whereas the use of 304 stainless steel (containing ~8-10.5% Ni) as cathode instead of nickel plate afforded **d-1** in 28% yield (entry 12). These control experiments unambiguously confirmed that the use of nickel plate cathod plays a critical role in the optimized electrochemical system, with catalytically active nickel species originating from cathodic corrosion/dissolution. Subsequent control experiments involving the addition of NiCl2 and iron cathode substitution further verified that cathode-mediated nickel dissolution constitutes the predominant catalytic source (entry 13).

**Table 1:** Optimization Studies


Entry	Deviation from the above condition^*a*^	d-1 (%)^*b*^
1	None	85 (80^*c*^)
2	Without electricity	N.D.
3	Without FeCl_3_	N.D.
4	Using FeCl_2_ instead of FeCl_3_	N.D.
5	Without TMHD (**L2**)	18
6	DMBP as the ligand	trace
7	**L3**-**L7** as the ligand	37 ~ 72
8	DMSO as the solvent	52
9 ^*d*^	Al(+)/Ni(-) instead of Fe(+)/Ni(-)	N.D.
10 ^*d,e*^	Al(+)/Ni(-) instead of Fe(+)/Ni(-)	32
11	Fe(+)/Fe(-) instead of Fe(+)/Ni(-)	N.D.
12	Fe(+)/304(-) instead of Fe(+)/Ni(-)	28
13^*e*^	Fe(+)/Fe(-) instead of Fe(+)/Ni(-)	60


^*a*^ Reaction conditions: Fe plate anode (10 × 10 × 0.3 mm), Ni plate cathode (10 × 10 × 0.15 mm), **a-1** (0.2 mmol, 1.0 equiv.), **b-1** (0.4 mmol, 2.0 equiv.), **c-1** (0.3 mmol, 1.5 equiv.), *n*Bu_4_NBF_4_ (0.3 mmol, 0.1 M), FeCl_3_ (0.04 mmol, 20 mol%), TMHD (0.06 mmol, 30 mol%), a mixture of dimethyl sulfoxide (DMSO) and 1,4-dioxane as solvent (3 mL, *v*/*v* = 2:1), 60 °C, constant current = 2 mA under N_2_ atmosphere for 12 h, 4.5 F/mol, undivided cell; ^*b*^ Yield determined by ^1^H NMR analysis using 1,3,5-trimethoxybenzene as internal standard. N.D. = Not detected; ^*c*^ Isolated yield; ^*d*^ Without FeCl_3_; ^*e*^ With NiCl_2_ (5 mol%). ^*f*^ Diastereomeric ratio (d.r.) determined by ^1^H NMR (trans:cis).

### Exploration of Substrate Scope

**Fig. 2:**
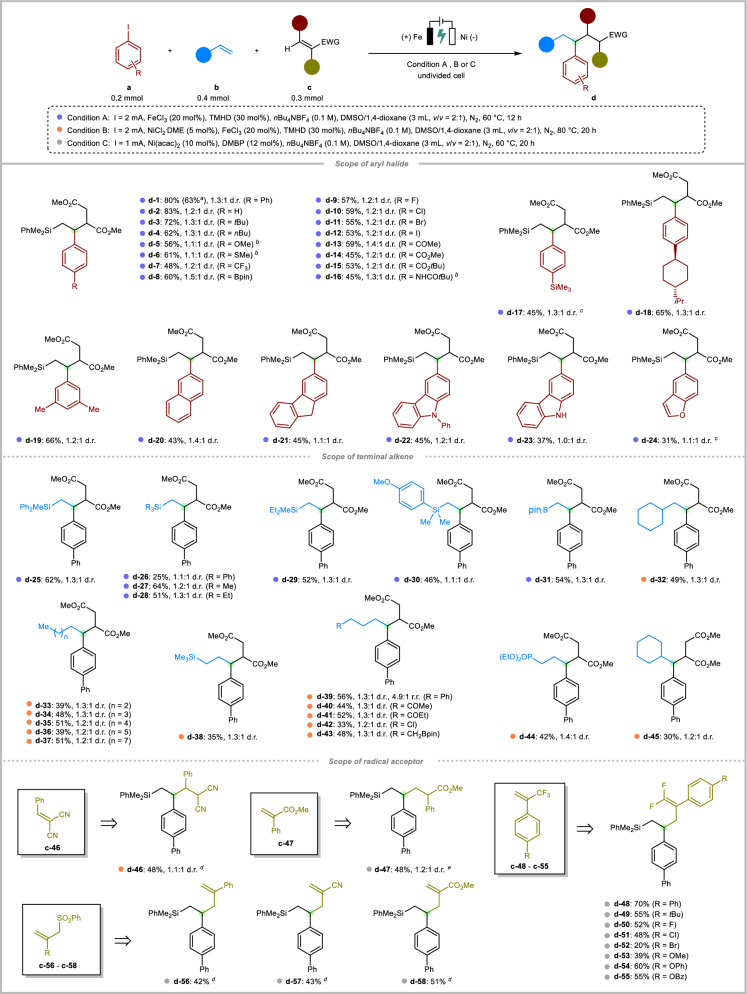
Substrate scope of alkene 1,1-difunctionalization reaction. Condition A: Fe plate anode (10 × 10 × 0.3 mm), Ni plate cathode (10 × 10 × 0.15 mm), **a** (0.2 mmol, 1.0 equiv.), **b** (0.4 mmol, 2.0 equiv.), **c** (0.3 mmol, 1.5 equiv.), *n*Bu_4_NBF_4_ (0.3 mmol, 0.1 M), FeCl_3_ (0.04 mmol, 20 mol%), TMHD (0.06 mmol, 30 mol%), a mixture of dimethyl sulfoxide (DMSO) and 1,4-dioxane as solvent (3 mL, *v*/*v* = 2:1), 60 °C, constant current = 2 mA under N_2_ atmosphere for 12 h, undivided cell. Condition B: Fe plate anode (10 × 10 × 0.3 mm), Ni plate cathode (10 × 10 × 0.15 mm), **a** (0.2 mmol, 1.0 equiv.), **b** (0.4 mmol, 2.0 equiv.), **c** (0.3 mmol, 1.5 equiv.), *n*Bu_4_NBF_4_ (0.3 mmol, 0.1 M), NiCl_2_^.^DME (0.01 mmol, 5 mol%), FeCl_3_ (0.04 mmol, 20 mol%), TMHD (0.06 mmol, 30 mol%), a mixture of dimethyl sulfoxide (DMSO) and 1,4-dioxane as solvent (3 mL, *v*/*v* = 2:1), 80 °C, constant current = 2 mA under N_2_ atmosphere for 20 h, undivided cell. Condition C: Fe plate anode (10 × 10 × 0.3 mm), Ni plate cathode (10 × 10 × 0.15 mm), **a** (0.2 mmol, 1.0 equiv.), **b** (0.4 mmol, 2.0 equiv.), **c** (0.3 mmol, 1.5 equiv.), *n*Bu_4_NBF_4_ (0.3 mmol, 0.1 M), Ni(acac)_2_ (0.02 mmol, 10 mol%), DMBP (0.024 mmol, 12 mol%), a mixture of dimethyl sulfoxide (DMSO) and 1,4-dioxane as solvent (3 mL, *v*/*v* = 2:1), 60 °C, constant current = 1 mA under N_2_ atmosphere for 20 h, undivided cell. ^*a*^ Large-scale reaction conditions: iron plate anode (30 × 30 × 0.3, mm), nickel plate cathode (30 × 30 × 0.2, mm), **a-1** (5.0 mmol), **b**-**1** (10.0 mmol), **c-1** (7.5 mmol), FeCl_3_ (162.5 mg, 20 mol%), TMHD (192.5 mg, 30 mol%) and NiCl_2_·DME (55.0 mg, 5.0 mol%), *n*Bu_4_NBF_4_ (0.10 M), DMSO and 1,4-dioxane as solvent (75 mL, *v*/*v* = 2:1), constant current = 6.0 mA under 60 °C for 100 h, 4.5 F/mol; ^*b*^ 80 °C; ^*c*^ Using aryl bromide instead of aryl iodide, 80 °C; ^*d*^ Using NiCl_2_·DME (4.4 mg, 10 mol%) as catalyst, DMF as solvent (3.0 mL), 80 °C; ^*e*^ Using NiCl_2_·DME (4.4 mg, 10 mol%) as catalyst, 80 °C.

After determining the optimized reaction conditions, we systematically investigated the generality of this transformation across a range of substrates, including aryl halides with varied substituents, unactivated alkenes, and diverse radical acceptors (Fig. 2). A total of 80 different 1,1-difunctionalized products were successfully synthesized using our electrochemical method. The use of aryl halides was firstly evaluated. 4-Biphenyl iodide (**d-1**) and unsubstituted iodobenzene (**d-2**) were converted into their respective products with good yields, excellent regioselectivities (>20:1), and moderate diastereoselectivities (1.3:1 and 1.2:1). Iodobenzene derivatives bearing *para*-alkyl substituents (*tert*-butyl, **d-3**; *n*-butyl, **d-4**) efficiently participated in the three-component reaction, while the successful conversion of the cyclohexyl-propyl-substituted analog (**d-18**) demonstrated its good tolerance toward structures with larger steric hindrance. Aryl iodide substrates bearing electron-withdrawing groups or challenged electron-donating on the aromatics^57,58^, including methoxy (**d-5**), thiomethoxy (**d-6**), trifluoromethyl (**d-7**), boronate ester (**d-8**), halide (**d-9** – **d-12**), ketone (**d-13**), ester (**d-14** and **d-15**), amide (**d-16**), and silane (**d-17**), were successfully employed in this selective electrochemical 1,1-difunctionalization reaction, yielding the corresponding three-component products in moderate to good yields with moderate diastereoselectivities^59–61^. *Meta*-methyl-substituted iodobenzene afforded the target product **d-19** in 66% yield. Furthermore, polycyclic and heteroaromatic systems, including naphthalene (**d-20**), fluorene (**d-21**), carbazole (**d-22** and **d-23**), and benzofuran (**d-24**), all proved to be viable substrates. In comparison to aryl iodides, aryl bromide substrates exhibited lower reactivity (**d-17**: 45%; **d-24**: 31%), primarily due to the inherently higher bond dissociation energy of C–Br bond.

The scope of alkene component was further assessed, including silyl- and boronate ester-subsitituted alkenes and conventional unactivated alkenes. As shown in Fig. 2, the reaction of 4-iodobiphenyl (**a-1**), dimethyl fumarate (**c-1**) with diverse alkenes successfully delivered three-component products **d-25** – **d-45** with moderate diastereoselectivities, which are stereorandom nature of radical recombination. Silane-substituted olefins demonstrated significant superiority, exclusively generating 1,1-difunctionalized products in moderate to good yields (**d-25** – **d-30**). However, the use of triphenylvinylsilane exhibited reduced reactivity under the standard conditions (**d-26**), which may be attributed to steric hindrance caused by its bulky triphenyl substituents, impairing the kinetics of the oxidative addition process. Notably, vinyl boronate ester was successfully incorporated into the reaction system, affording the target product **d-31** in 54% yield. This outcome suggests enhanced stability of the benzyl-nickel intermediate is superior to that of its α-boryl-nickel counterpart under electrochemical conditions. The reaction of vinylcyclohexane achieved moderate yield of the desired product **d-32** with excellent 1,1-regioselectivity (> 20:1) and 1.3:1 d.r., while linear alkenes ranging from 1-pentene to 1-decene maintained moderate yields under electrochemical conditions involving nickel species (5 mol% NiCl_2_^.^DME, **Condition B**). Subsequent substrate scope investigation demonstrated that alkenes bearing a range of functional groups, including allylic silyl (**d-38**), aryl (**d-39**), ketone (**d-40**), ester (**d-41**), halogen (**d-42**), and even boronate ester (**d-43**) and phosphonate (**d-44**), were all compatible with this reaction system, delivering the target products in moderate yields. Notably, methylenecyclohexane (**d-45**), featuring an exocyclic double bond, also exhibited acceptable reactivity. These findings underscore the critical balance between substrate steric demand and the stability of nickel intermediates in determining reaction outcomes.

Next, we systematically investigated the substrate scope of electron-deficient alkenes as suitable radical acceptors, including trifluoropropenyl-substituted arenes, dicyano-, ester-, and sulfonyl-substituted alkenes, which revealed multifaceted interactions among the structure of the radical acceptors, catalytic system, and solvent environment in regulating reaction efficiency (Fig. 2). Trifluoropropenyl-substituted arenes were successfully applied in this electrochemical system involving Ni(acac)_2_ as the catalyst and DMBP as the supporting ligand (**Condition C**), with their reactivity displaying a strong correlation with the electronic nature of substituents. The biphenyl-substituted acceptor delivered the target product **d-48** in high yield, benefiting from radical stabilization via extended conjugation, while the sterically hindered *tert*-butyl-substituted analogue exhibited diminished efficiency (**d-49**, 55%). Trifluoropropenyl-substituted arenes containing electron-donating groups such as methoxy (**d-53**), phenoxy (**d-54**), and benzyloxy (**d-55**) provided the corresponding products in moderate yields. The observed attenuation in alkyl radical trapping efficiency may be attributed to electron-donating groups reducing the substrate’s electrophilicity, thereby diminishing its affinity toward nucleophilic species^62^. Halogenated acceptors exhibited moderate efficiency (**d-50** and **d-51**), whereas the brominated variant achieved acceptable yields despite potential undesirable side reactions arising from C–Br bond cleavage (**d-52**). In addition, the use of benzylidenemalononitrile (**d-46**) and phenyl acrylate (**d-47**) as radical acceptors successfully afforded moderate yields in both DMF and DMSO/dioxane systems. Remarkably, increasing the Ni catalyst loading to 10 mol% enabled sulfonyl-containing acceptors to participate effectively in DMF, furnishing moderate yields of the desired 1,1-difunctionalized products (**d-56** – **d-58**). These results highlight the critical role of electronic fine-tuning and steric-kinetic balancing in optimizing radical-involved electrochemical transformations.

**Fig. 3:**
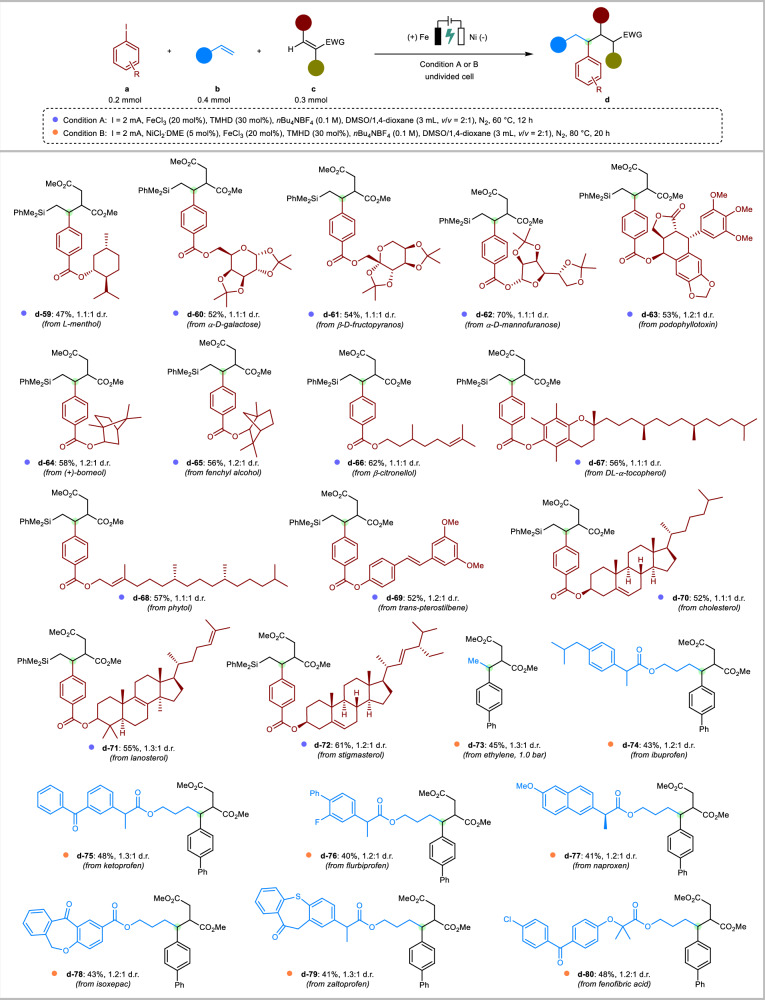
Late-stage functionalization of natural products and drug molecules. Condition A: Fe plate anode (10 × 10 × 0.3 mm), Ni plate cathode (10 × 10 × 0.15 mm), **a** (0.2 mmol, 1.0 equiv.), **b** (0.4 mmol, 2.0 equiv.), **c** (0.3 mmol, 1.5 equiv.), *n*Bu_4_NBF_4_ (0.3 mmol, 0.1 M), FeCl_3_ (0.04 mmol, 20 mol%), TMHD (0.06 mmol, 30 mol%), a mixture of dimethyl sulfoxide (DMSO) and 1,4-dioxane as solvent (3 mL, *v*/*v* = 2:1), 60 °C, constant current = 2 mA under N_2_ atmosphere for 12 h, undivided cell. Condition B: Fe plate anode (10 × 10 × 0.3 mm), Ni plate cathode (10 × 10 × 0.15 mm), **a** (0.2 mmol, 1.0 equiv.), **b** (0.4 mmol, 2.0 equiv.), **c** (0.3 mmol, 1.5 equiv.), *n*Bu_4_NBF_4_ (0.3 mmol, 0.1 M), NiCl_2_^.^DME (0.01 mmol, 5 mol%), FeCl_3_ (0.04 mmol, 20 mol%), TMHD (0.06 mmol, 30 mol%), a mixture of dimethyl sulfoxide (DMSO) and 1,4-dioxane as solvent (3 mL, *v*/*v* = 2:1), 80 °C, constant current = 2 mA under N_2_ atmosphere for 20 h, undivided cell.

Late-stage functionalization constitutes a powerful strategy for the assembly and diversification of novel molecular entities with improved physicochemical or biological activities^63^. To demonstrate this, we leveraged 4-iodobenzoic acid to construct iodinated arene units directly on natural products bearing complex alcohol moieties, thereby enabling the application of our electrochemical 1,1-difunctionalization reaction to diverse bioactive natural products. As shown in Fig. 3, this approach validates its utility in derivatizing structurally intricate scaffolds. Experimental data revealed that our method showed broad compatibility across terpenoids, carbohydrates, steroids, and phenolic natural products. Carbohydrate derivatives, such as α-D-galactose (**d-60**), β-D-fructopyranos (**d-61**), and α-D-mannofuranose (**d-62**) were obtained in moderate to high yields, underscoring their potential as drug carriers or vaccine adjuvant precursors. Monoterpenes, including *L*-menthol, (+)-borneol, and fenchyl alcohol, which possess intrinsic antibacterial and transdermal penetration activities, were functionalized with dimethylphenylvinylsilane (**b-1**) and dimethyl fumarate (**c-1**) to give the corresponding three-component products **d-59,** **d-64**, and **d-65** in 47-58% yields, reflecting the method’s tolerance toward rigid cyclic frameworks. Broad-spectrum pharmacologically active podophyllotoxin was found to be successfully employed, and gave **d-63** in 53% yield with 1.2:1 d.r. Moreover, long-chain aliphatic alcohols, including β-citronellol (**d-66**) and phytol (**d-68**), underwent efficient functionalization, suggesting potential applications in flavor chemistry. Phenolic derivatives (**d-67**, 56%; **d-69**, 52%), as antioxidant or antitumor leads, offer valuable avenues for structure-activity relationship studies through site-selective aryl iodination. Furthermore, sterols (**d-70,** **d-71**) and phytosterols (**d-72**) were efficiently modified, establishing a modular platform for the targeted derivatization of steroid-based therapeutics (e.g., anti-inflammatory or lipid-lowering agents). The consistent yields (52-61%) across these polycyclic systems highlight adaptability toward sterically demanding architectures, and enables flexible modification of iodinated aromatics in natural products. High efficiency in the modification of carbohydrates and aliphatic alcohols provides a robust tool for drug conjugate development, while compatibility with steroids and terpenoids expands pathways for natural product diversification.

Subsequently, our study successfully achieved the 1,1-difunctionalization reaction of alkenes derived from pharmacologically relevant molecules by supplementing the standard reaction conditions with 5 mol% NiCl_2_·DME as a co-catalyst (**Condition B**). This advancement systematically validates the practicality of this method in late-stage drug diversification, as experimental data demonstrated broad compatibility with diverse pharmaceutical scaffolds (Fig. 3). Nonsteroidal anti-inflammatory drug (NSAID) derivatives, including carboxylic acid-based agents such as ibuprofen (**d-74**), ketoprofen (**d-75**), flurbiprofen (**d-76)**, naproxen (**d-77**), Isoxepac (**d-78**), and zaltoprofen (**d-79**), all successfully underwent the 1,1-difunctionalization reaction giving the corresponding products in moderate yields. These modifications offer valuable chemical strategies to enhance drug solubility, membrane permeability, and targeted delivery properties. In addition, the successful derivatization of fenofibric acid (**d-80**) – a phenoxy acid-based lipid-lowering agent – confirms the applicability of this method in cardiovascular drug engineering, opening up promising avenues for prodrug design and metabolic stability optimization. Ethylene, as the simplest alkene, has an annual production of 170 million tons worldwide^64^. However, owing to its inherent simplicity and concerns about handling this flammable gas, ethylene has rarely been utilized in a radical-mediated conversion process without high pressure. Notably, ethylene gas underwent efficient conversion under these conditions (**d-73**), underscoring the method’s versatility toward lightweight alkenes and its broader potential in synthetic organic chemistry.

**Fig. 4:**
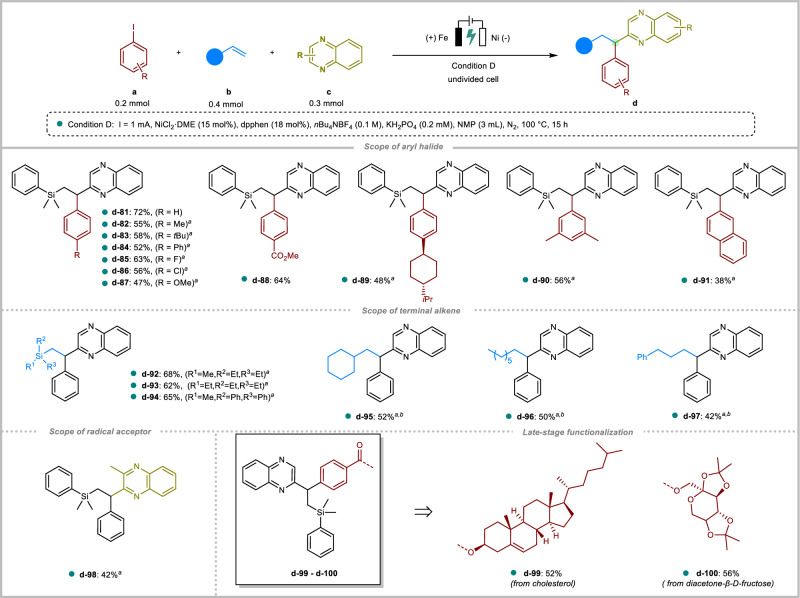
Substrate scope of alkene 1,1-hetero-di-arylation. Condition D: Fe plate anode (10 × 10 × 0.3 mm), Ni plate cathode (10 × 10 × 0.15 mm), **a** (0.2 mmol, 1.0 equiv.), **b** (0.4 mmol, 2.0 equiv.), **c** (0.3 mmol, 1.5 equiv.), *n*Bu_4_NBF_4_ (0.3 mmol, 0.1 M), NiCl_2_^.^DME (0.03 mmol, 15 mol%), dpphen (0.036 mmol, 18 mol%), NMP as solvent (3 mL), 100 °C, constant current = 1 mA under N_2_ atmosphere for 15 h, undivided cell. ^*a*^ 130 °C; ^*b*^ Alkene **b** (0.6 mmol, 3.0 equiv.).

To further expand the applicability of this methodology, we extended the catalytic system to 1,1-hetero-diarylation under **Condition D** (Fig. 4). This system also demonstrated excellent generality, successfully constructing a library of 20 structurally diverse 1,1-hetero-diarylated products. The results demonstrated that the catalytic system exhibited broad compatibility with structurally diverse aryl iodides. Electron-neutral arenes and alkyl-substituted arenes (**d-81** – **d-83**) afforded the coupled products in moderate to good yields. In contrast, substrates with meta-disubstitution or bulky para-substituents (**d-89,** **d-90**) exhibited moderate yield reduction due to kinetically impeded oxidative addition. The halogen substituents on substrates (**d-85,** **d-86**) remained intact under the reaction conditions, providing key sites for subsequent site-specific functionalization. Electron-donating groups (**d-87**) attenuated the electrophilicity of the C − I bond, resulting in diminished yield. Conversely, electron-deficient substrates (**d-88**) displayed enhanced reactivity, likely via stabilization of anionic intermediates. Notably, 4-Iodobiphenyl and 2-iodonaphthalene (**d-84,** **d-91**) reacted smoothly. Thereafter, we further evaluated the tolerance of this system toward un-activated alkenes. Silane-containing alkenes (**d-92** – **d-94**) afforded the target products in higher yields compared to alkyl alkenes, highlighting the pivotal role of silicon atoms in facilitating migratory insertion. Although linear alkyl and cyclohexyl alkenes (**d-95** – **d-97**) exhibited diminished reactivity, moderate efficiencies were maintained. Methyl substitution on the quinoxaline skeleton (**d-98**) led to slightly reduced reactivity due to steric hindrance. Finally, efficient functionalization of complex natural product derivatives was achieved: cholesterol and fructose derivatives (**d-99,** **d-100**) demonstrated the potential of this methodology for late-stage derivatization.

### Mechanistic investigations

**Fig. 5:**
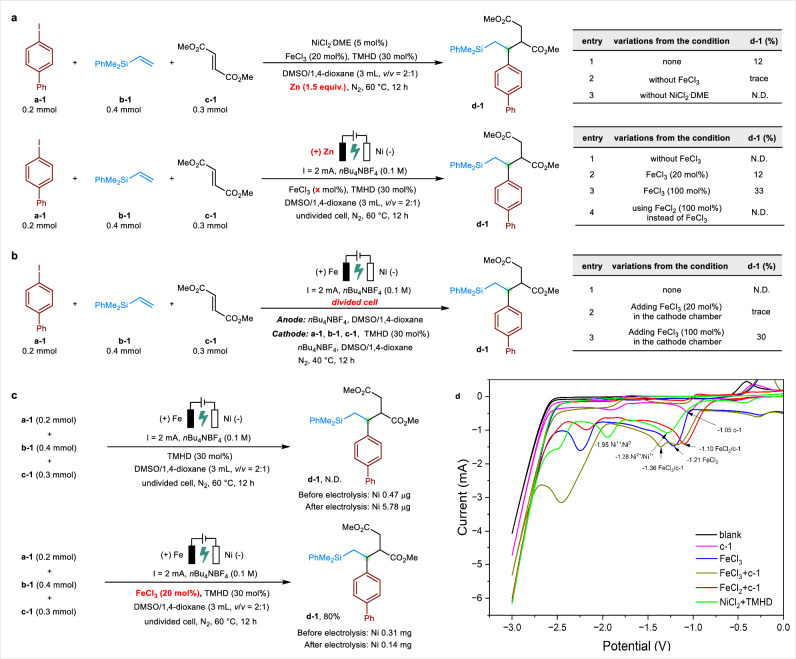
Investigation of the role for iron species. **a** Control experiments. **b** Divided-cell experiments. **c** ICP-MS detection. **d** Cyclic voltammetry (CV) studies.

Given the critical roles of the Fe(III) catalyst and the sacrificial anodes in the designed electrocatalytic system, we systematically investigated the function of the iron species using 4-iodobiphenyl (**a-1**), dimethylphenylvinylsilane (**b-1**), and dimethyl fumarate (**c-1**) as model substrates. As shown in Fig. 5a, employing zinc as a chemical reducing agent without electrochemical input afforded the multi-component product **d-1** in 12% yield in the presence of both Fe and Ni catalysts^65^. Notably, removal of FeCl_3_ severely suppressed activity, indicating the indispensable role of Fe(III) species in maintaining the catalytic cycle. Similarly, the removal of NiCl_2_·DME completely halted the reaction, establishing nickel as the central catalytic component. Furthermore, electrochemical experiments revealed that substituting the iron anode with zinc plate in the absence of FeCl_3_ catalyst resulted in undetectable **d-1** formation, highlighting the dual functions of the Fe(III) species in both electrochemical and catalytic processes. Increasing the FeCl_3_ loading (from 20 to 100 mol%) enhanced the reaction efficiency, suggesting that FeCl_3_ both activates the nickel catalytic cycle and serves as a Lewis acid to facilitate the oxidative process^66^.

To probe the reaction mechanism, we employed a divided cell device with physically isolated anode and cathode chambers (Fig. 5b). The reaction was completely suppressed without FeCl_3_ in the cathodic chamber, indicating Fe(III) species as essential. Addition of 20 mol% FeCl_3_ to the cathode yielded only trace amounts of product **d-1**, increasing the concentration of iron (FeCl_3_: 100 mol%) elevated the yield to 30%. This result conclusively demonstrates that the catalytic cycle operates predominantly at the cathode and is directly driven by Fe(III) species. This configuration eliminates anodic interference, demonstrating iron’s central role at the cathode^67^. Optimization studies previously revealed that Fe(III) species indirectly participate by oxidizing nickel metal at the cathode, yielding key Ni(II) species. To validate Fe(III)-mediated nickel activation, we quantified nickel dissolution via inductively coupled plasma mass spectrometry (ICP-MS)^68,69^. As shown in Fig. 5c, systems without FeCl_3_ showed near-background nickel levels (0.47 μg) before electrolysis, and only a negligible increase (5.78 μg) post-electrolysis, indicating minimal Ni cathode dissolution. In stark contrast, FeCl_3_-supplemented systems exhibited 0.31 mg nickel content pre-electrolysis – a 660-fold increase attributed to Fe(III)-induced oxidative cathode etching. This Fe(III)-activated nickel pool directly correlated with enhanced catalytic efficiency under the standard conditions. All these results mechanistically establish that external Fe(III) mediates oxidative nickel leaching from the cathode, generating catalytically active nickel species in solution. This aligns with prior control experiments in which exogenous nickel salts restored reactivity in FeCl_3_-containing systems, corroborating the indispensability of Fe(III) in initiating the nickel catalytic cycle.

To further elucidate the role of nickel and iron in the reaction, the redox potentials of electron-deficient alkene (**c-1**), FeCl_3_ and NiCl_2_ were evaluated by cyclic voltammetry (CV) experiments, as shown in Fig. 5d^51,70^. Surprisingly, we found that the reduction potential of **c-1** was around -1.05 V vs. Ag/AgNO_3_, lower than that of the NiCl_2_/TMHD complex (*E*_p_^red^ = -1.28 V vs. Ag/AgNO_3_). This indicates **c-1** would undergo preferential reduction in the electrochemical system, preventing the desired 1,1-difunctionalization. Subsequent measurement revealed FeCl_3_ alone reduces at -1.21 V vs. Ag/AgNO_3_. Remarkably, adding **c-1** to FeCl_3_ shifted the mixture’s reduction potential to -1.36 V vs. Ag/AgNO_3_, thereby inverting the reduction sequence^71^. When c-1 was mixed with FeCl_2_, the reduction potential of the mixture was -1.10 V (vs. Ag/AgNO_3_). This value is still higher (i.e., less negative) than that of the NiCl_2_/TMHD complex (*E*_p_^red^ = -1.28 V). This result indicates that Fe^2+^ is unable to effectively protect the radical receptor, unlike Fe^3+^, thereby supporting the crucial role of Fe^3+^ in this process. Crucially, this modified potential (-1.36 V) remains less negative than the Ni(I)/Ni(0) (*E*_p_^red^ = -1.95 V vs Ag/AgNO_3_), preserving the cathode’s ability to reduce Ni(I) species, which is consistent with previous reports^71–73^. Additionally, NMR studies revealed the interaction between Fe(III) catalyst and dimethyl fumarate (**c-1**). Increasing FeCl_3_ concentration (0 → 30 mol%) progressively shifted both carbonyl (C = O) and alkene (C = C) ^13^C NMR resonances downfield (higher δ values) (Supplementary Fig. 8). In marked contrast, control experiments demonstrated that Fe(II) failed to produce similar effects under identical conditions, revealing the critical role of Fe(III) species in modulating **c-1**’s electronic structure via a Lewis acid activation mechanism^74^. Crucially, Fe(III) ensures nickel retains electrochemical dominance, driving selective 1,1-difunctionalization while suppressing side reactions from premature reduction of low-potential substrates like **c-1**.

**Fig. 6:**
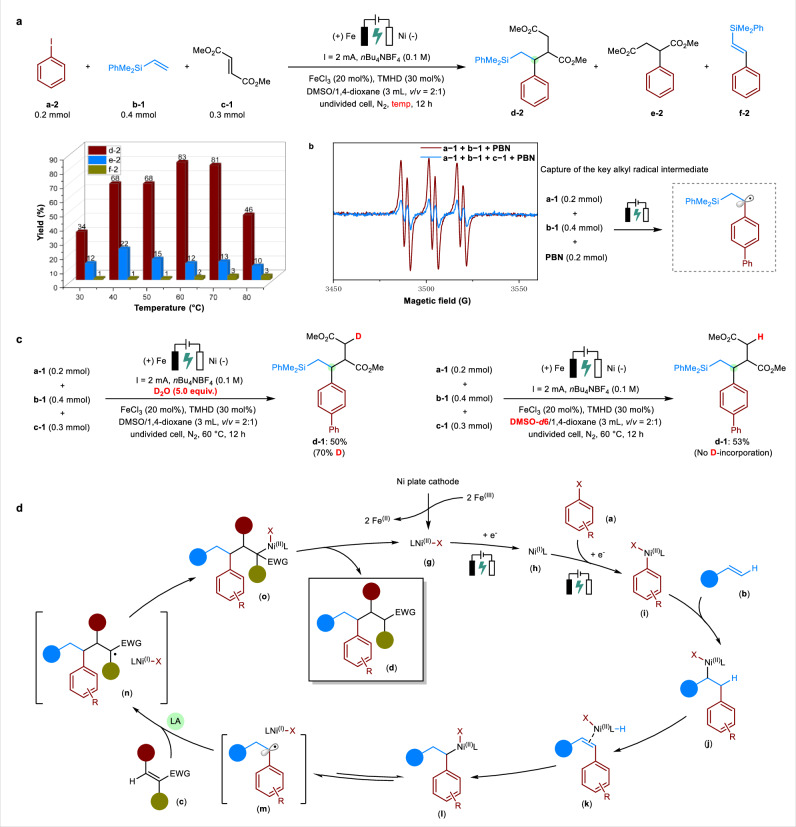
Mechanistic studies. **a** Temperature control experiments. **b** EPR experiments. **c** Isotope-labelling experiments. **d** Proposed reaction mechanism.

After elucidating the role of iron in the electrochemical system, we then investigated the temperature dependence of the reaction. Figure 6a illustrates the temperature-dependent yields of three major products: the target compound **d-2** and two-component byproducts, **e-2** and **f-2**. As temperature increased from 30°C to 80°C, the yield of **d-2** progressively increased to a peak (83%) at 60°C. Concurrently, the yield of **e-2** initially rose but declined at higher temperatures, while **f-2** exhibited low but gradually increasing yields across the tested range. Subsequent electron paramagnetic resonance (EPR) spectroscopy revealed a distinct alkyl radical signal (g = 2.0072, *a*_N_ = 15.05377 G, *a*_H_ = 3.28446 G) upon addition of the radical trapping agent – *N*-*tert*-butyl-α-phenylnitrone (PBN), directly indicating the generation of benzylic radicals via Ni-mediated homolytic cleavage (Fig. 6b)^75^. To probe the hydrogen source in hydrogenated products, deuterium-labeling experiments were conducted (Fig. 6c)^76,77^. Under standard Condition A, the addition of D_2_O (5.0 equiv.) afforded **d-1** in 50% yield with 70% deuterium incorporation, whereas using DMSO-d_6_ as the solvent yielded 53% **d-1** without any detectable deuterium. These results unequivocally identify water as the exclusive proton source for the hydrogenation step, ruling out solvent participation and suggesting a protonation-driven quenching mechanism^78,79^. This behavior parallels iron-catalyzed reductive cross-alkene couplings, underscoring a shared mechanistic paradigm for radical termination in related systems.

Integrating cyclic voltammetry with the above mechanistic investigations, this work establishes an iron-promoted Ni-catalytic cycle for the electrochemical alkene 1,1-difunctionalization reaction. Catalytically active nickel species originate from either exogenous addition (Conditions B/C) or Fe(III)-mediated oxidative etching of nickel plate cathodes (Condition A). As depicted in Fig. 6d, the catalytic cycle commences when Fe(III) oxidizes metallic nickel at the cathode surface, generating homogeneous Ni(II) species (**g**). Cathodic reduction then converts Ni(II) to Ni(I), which undergoes oxidative addition with aryl halides. Further cathodic reduction converts this intermediate into the critical aryl-Ni(II) species (**i**). Alkene activation proceeds via migratory insertion: coordination of (**i**) with alkene (**b**) triggers carbometallation, forming β-aryl-Ni(II) species (**j**). Subsequent β-hydride elimination generates Ni−H species, followed by oxidative addition that drives 1,2-nickel migration to afford thermodynamically stable α-aryl-Ni(II) species (**l**). Homolytic C−Ni(II) bond cleavage in (**l**) – confirmed by EPR spectroscopy – produces Ni(I) and benzyl radical (**m**). The radical is selectively captured by the electron-deficient alkene (**c**) either cooperatively with the Fe(III) Lewis acid (Conditions A/B) or directly (Condition C/D), proceeding via a transient radical adduct intermediate (**n**) to ultimately form the alkyl-Ni(II) intermediate (**o**). The catalytic cycle bifurcates at Intermediate **o**: β-elimination affords alkenylated products, while hydrogenative termination delivers alkyl derivatives^17,80–82^. Throughout the cycle, Fe(III) exhibits dual functionality as both nickel activator (oxidant) and radical selectivity modulator (Lewis acid). Continuous cathodic potential dynamically maintains the Ni(I)/Ni(II) equilibrium, ensuring sustained catalytic turnover. This framework unifies electrochemical activation, migratory insertion kinetics, and redox-state control, establishing a coherent mechanism for nickel-catalyzed electrochemical difunctionalization of unactivated alkenes.

## Discussion

In summary, we have established a synergistic iron-nickel electrocatalytic platform that overcomes longstanding challenges in alkene 1,1-difunctionalization, achieving regioselective coupling of aryl halides, radical acceptors with unactivated alkenes under mild conditions. By exploiting the dual functionality of Fe(III) species—oxidatively activating metallic nickel to generate catalytically active Ni(II) species and modulating substrate redox hierarchies via Lewis acid-mediation—we circumvent premature reduction of low-potential substrates, a critical bottleneck in traditional systems. Mechanistic interrogation through ICP-MS, NMR spectroscopy and CV studies revealed that Fe(III) dynamically regulates nickel dissolution and stabilizes radical acceptor, enabling a cascade involving migratory insertion, radical trapping, and protonation-driven termination. The system demonstrates broad applicability (100 examples, up to 85% yield), accommodating electron-diverse aryl halides, polycycles, and bioactive natural products. Gram-scale synthesis and late-stage drug derivatization underscore practical utility. By replacing organoboron reagents with cheaper, more stable and available aryl halides and eliminating chemical reductants, this approach effectively tackles the challenges of scalability and sustainability in cross-coupling reactions. By combining electrochemically-driven nickel redox cycles with the multifunctionality of iron species, it opens up a practical pathway for the difficult 1,1-difunctionalization reaction, bridging the gap between radical chemistry and transition metal catalysis. This strategy not only enables adaptable catalytic system design but also shows significant promise for pharmaceutical synthesis and functional material development.

## Methods

### General procedure for electrochemical alkene 1,1-difunctionalization (Condition A)

In an oven dried 8 mL reaction vial with a magneton, a Fe anode and a Ni cathode, substrate **a** (0.2 mmol, 1.0 equiv.), **b** (0.4 mmol, 2.0 equiv.) and **c-1** (0.3 mmol, 1.5 equiv.) and electrolyte *n*Bu_4_NBF_4_ (98.8 mg, 0.1 M) were added, followed by the addition of FeCl_3_ (6.5 mg, 20 mol%), 2,2,6,6-tetramethyl-3,5-heptanedione (TMHD, 11.0 mg, 30 mol%) and 3.0 mL solvent (extra dry, DMSO:1,4-dioxane=2:1). The distance of electrodes was approximately 0.5 cm. Weighing of all drugs and assembly of reaction units were completed in a nitrogen-filled glove box, and the flask was then removed from the glove box. The constant current (2.0 mA) electrolysis was then performed at 60 °C under N_2_ atmosphere with stirring for 12 h. Upon completion, the reaction mixture was diluted with EtOAc and washed with NH_4_Cl saturated solution (3 x equal volume) for three times. The combined organic layer was dried over anhydrous Na_2_SO_4_, and the solvent was then removed under reduced pressure. The resulting mixture was purified by column chromatography on silica gel (eluted with PE / EtOAc = 30:1 to 7:1, *v*/*v*) to afford the desired products **d-1** – **d-31,** **d-59** – **d-72**.

### General procedure for electrochemical alkene 1,1-difunctionalization (Condition B)

In an oven dried 8 mL reaction vial with a magneton, a Fe anode and a Ni cathode, substrate **a-1** (0.2 mmol, 1.0 equiv.), **b** (0.6 mmol, 3.0 equiv.) and **c-1** (0.3 mmol, 1.5 equiv.) and electrolyte *n*Bu_4_NBF_4_ (98.8 mg, 0.1 M) were added, followed by the addition of FeCl_3_ (6.5 mg, 20 mol%), TMHD (11.0 mg, 30 mol%), NiCl_2_^.^DME (2.2 mg, 5 mol%) and 3.0 mL solvent (extra dry, DMSO:1,4-dioxane=2:1). The distance of electrodes was approximately 0.5 cm. Weighing of all drugs and assembly of reaction units were completed in a nitrogen-filled glove box, and the flask was then removed from the glove box. The constant current (2.0 mA) electrolysis was then performed at 60 °C under N_2_ atmosphere with stirring for 20 h. Upon completion, the reaction mixture was diluted with EtOAc and washed with NH_4_Cl saturated solution (3 x equal volume) for three times. The combined organic layer was dried over anhydrous Na_2_SO_4_, and the solvent was then removed under reduced pressure. The resulting mixture was purified by column chromatography on silica gel (eluted with PE / EA = 30:1 to 7:1, *v*/*v*) to afford the desired products **d-32** – **d-46, d-73** – **d-80**.

### General procedure for electrochemical alkene 1,1-difunctionalization (Condition C)

In an oven dried 8 mL reaction vial with a magneton, a Fe anode, and a Ni foam cathode, substrate **a-1** (0.2 mmol, 1.0 equiv.), **b-1** (0.4 mmol, 2.0 equiv.) and **c** (0.3 mmol, 1.5 equiv.) and electrolyte *n*Bu_4_NBF_4_ (98.8 mg, 0.1 M) were added, followed by weighting Ni(acac)_2_ (5.1 mg, 10 mol%), DMBP (4.4 mg, 12 mol%) and 3.0 mL solvent (extra dry, DMSO:1,4-dioxane=2:1). The distance of electrodes was approximately 0.5 cm. Weighing of all drugs and assembly of reaction units were completed in a nitrogen-filled glove box, and the flask was then removed from the glove box. The constant current (1.0 mA) electrolysis was then performed at 60 °C under N_2_ atmosphere with stirring for 20 h. Upon completion, the reaction mixture was diluted with EtOAc and washed with NH_4_Cl saturated solution (3 x equal volume) for three times. The combined organic layer was dried over anhydrous Na_2_SO_4_, and the solvent was then removed under reduced pressure. The resulting mixture was purified by column chromatography on silica gel (eluted with PE/EA = 100 to 7:1, *v*/*v*) to afford the desired products **d-47** – **d-58**.

### General procedure for electrochemical alkene 1,1-difunctionalization (Condition D)

In an oven dried 8 mL reaction vial with a magneton, a Fe anode, and a Ni foam cathode was added the substrate **a** (0.2 mmol, 1.0 equiv.), **b** (0.4 – 0.6 mmol, 2.0 – 3.0 equiv.) and **c-81,** **c-97** (0.3 mmol, 1.5 equiv.) and electrolyte *n*Bu_4_NBF_4_ (98.8 mg, 0.1 M), followed by weighting NiCl_2_·DME (6.6 mg, 15 mol%), 2,9-diphenyl-1,10-phenanthroline (dpphen, 12.0 mg, 18 mol%) and KH_2_PO_4_ (0.2 mmol, 27.2 mg), 3.0 mL solvent (extra dry, NMP). The distance of electrodes was approximately 0.5 cm. Weighing of all drugs and assembly of reaction units are done in a nitrogen-filled glove box. The flask was then removed from the glove box (Figure S1). The constant current (1.0 mA) electrolysis was then performed at 100 °C under N_2_ atmosphere with stirring for 15 h, 2.8 F/mol. Upon completion, the reaction mixture was diluted with EtOAc and washed with NH_4_Cl saturated solution (3 x equal volume) for three times. The combined organic layer was dried over anhydrous Na_2_SO_4_, and the solvent was then removed under reduced pressure. The resulting mixture was purified by column chromatography on silica gel (eluted with PE/EA = 30:1 to 10:1, *v*/*v*) to afford the desired products **d-81** – **d-100**.

## Supplementary information

Supplementary Information

Transparent Peer Review file

## Data Availability

Materials and methods, optimization studies, experimental procedures, mechanistic studies, ^1^H NMR spectra, ^13^C NMR spectra, EPR spectroscopy, and mass spectrometry data, generated in this study are provided in the Supplementary Information file. Raw NMR data are available via Zenodo at https://zenodo.org/records/18978625. Data supporting the findings of this manuscript are also available from the corresponding author upon request.
