# Supplementary material for: Synergistic Fe/Ni catalysis for electrochemical 1,1-difunctionalization of alkenes
Source: Nat Commun. 2026 May 2;17:5961. doi: 10.1038/s41467-026-72546-x (PMC13342334; doi:10.1038/s41467-026-72546-x)
Supplement: Supplementary file 1 — Supplementary Information [file 41467_2026_72546_MOESM1_ESM.pdf]

Supplementary Information

Synergistic Fe/Ni Catalysis for Electrochemical 1,1-Difunctionalization of Alkene

Pengwei Hu,[1] Chao Yang,[1] Lin Guo[1], and Wujiong Xia*[1,2]

[1] State Key Lab of Urban Water Resource and Environment, School of Science, Harbin Institute of Technology (Shenzhen), Shenzhen 518055, China

[2] School of Chemistry and Chemical Engineering, Henan Normal University, Xinxiang, Henan 453007, China

* Email: xiawj@hit.edu.cn

Table of Contents

1. Experimental Section 1

a) General information 1

b) Methods for the synthesis of substrates 2

c) Optimization of the reaction conditions 18

d) General procedure for the 1,1-difunctionalization of alkenes 25

e) Mechanistic experiments. 28

2. Characterization Data of the Products 45

3. NMR Spectra for the Products 121

4. References 252

1. Experimental Section

a) General information

All chemicals, unless otherwise noted, were purchased from commercial sources and were used without further purification. Unless stated otherwise, all reactions were carried out under nitrogen atmosphere. Chromatographic purification of products was accomplished by flash chromatography using silica gel. Thin-layer chromatography (TLC) was performed on Silicycle 250 mm silica gel F-254 plates, and visualized using UV fluorescence (λmax = 254 nm), and/or developed using standard $KMnO_4$ stain. The photoreaction instrument (WPTEC-1020L) was purchased from WATTCAS, China.

^{1}H NMR (400 MHz), ^{13}C NMR (101 MHz) and ^{19}F NMR (376 MHz) spectra were recorded on a Bruker AV-400 spectrometer or a Quantum-I Plus 400 in Chloroform-d. For ^{1}H NMR, Chloroform-d (δ = 7.26 ppm) or tetramethylsilane (TMS, δ = 0 ppm) serves as the internal standard; for ^{13}C NMR, Chloroform-*d* (δ = 77.16 ppm) serves as the internal standard. Data are reported as follows: chemical shift (in ppm), multiplicity (s = singlet, d = doublet, t = triplet, q = quartet, p = quintet, hept = heptet, m = multiplet, br = broad), coupling constant (in Hz), and integration. HR-MS spectra were recorded on a Waters Xevo G2QTOF/UPLC mass spectrometer using electrospray ionization. EPR experiments were conducted using Bruker Elexsys E580 Spectrometer. The cyclic voltammetry measurements were detected by using a CHI 760E electrochemical workstation.

b) Methods for the synthesis of substrates

Preparation of natural products and drug molecular derivatives

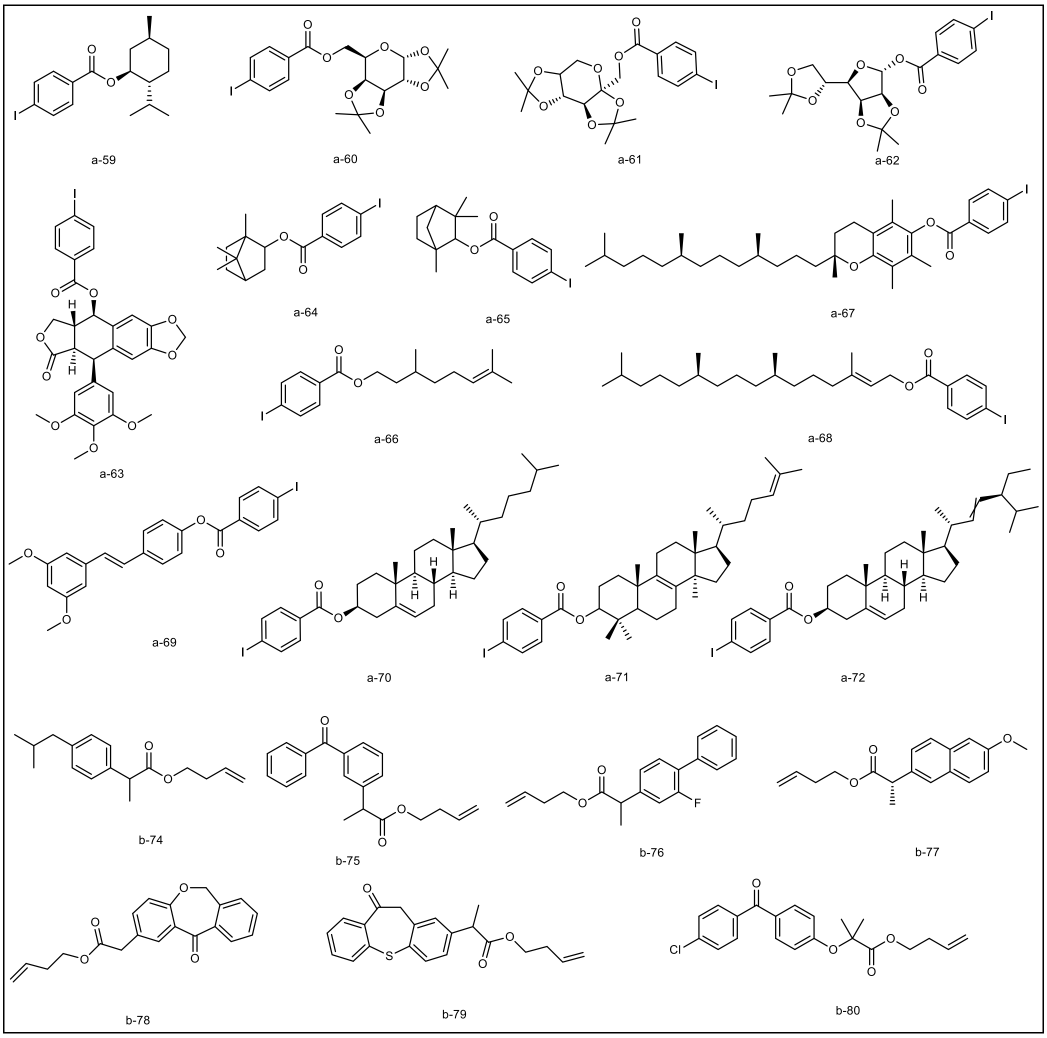

Supplementary Figure 1. Natural products and drug molecular derivatives

General procedure 1 for the synthesis of natural products and drug molecular derivatives:

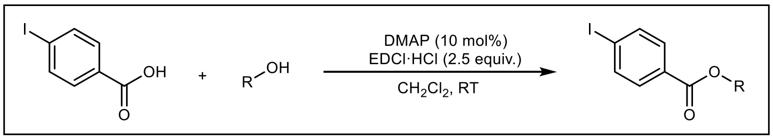

According to a related literature,[1] to a solution of alkanols (5.0 mmol, 1.0 equiv.) and 4-iodobenzoic acid (5.0 mmol,1.0 equiv.) in CH_2Cl_2 (50 mL), DMAP (20 mol%, 1.0 mmol) and EDCI (11.0 mmol, 2.2 equiv.) was added. The mixture was stirred at room temperature under air atmosphere until the reaction was complete as observed from TLC monitoring. The mixture was diluted with distilled water (50 mL) and the DCM

layer was separated, dried over anhydrous Na_2SO_4 and concentrated. The solvent was removed and the residue was purified by silica gel column chromatography (PE / EA as eluent) to give the corresponding compound (**a-59** – **a-66**, **a-68** and **a-70** – **a-72**).

General procedure 2 for the synthesis of natural products and drug molecular derivatives:

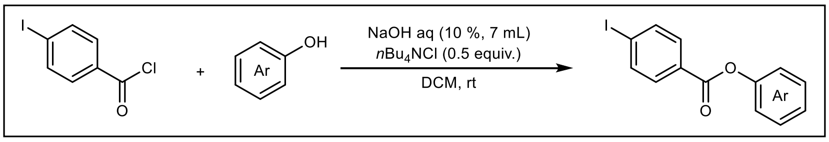

According to the related literature,[2] 4-Iodobenzoyl chloride (15 mmol, 1.0 equiv.) was directly added to a solution of phenol (15 mmol, 1.0 equiv.) and 10% aqueous sodium hydroxide (20.0 mL,50 mmol NaOH) in a 50-mL flask. Solutions of $^{n}Bu_4NCl$ (1.5 mmol) in 5 mL of dichloromethane were prepared. After cooling all solutions to 0 °C, they were mixed at simultaneously. The reaction mixture was kept under vigorous magnetic stirring (400 rpm) at 0 C for 5 minutes and then poured over 20 mL of icy-cold water. The organic layer was separated and the aqueous layer was extracted twice with 20 mL of diethyl ether. The combined organic extracts were washed with saturated NaCl solution. After dried with Na_2SO_4, and concentrated. After solvent evaporation, the product was subjected to column chromatography for purification, giving product **a-67** and **a-69**.

General procedure 3 for the synthesis of natural products and drug molecular derivatives:

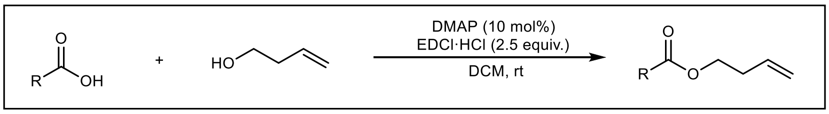

According to a related literature,[1] to a solution of 3-Buten-1-ol (5.0 mmol, 1.0 equiv.) and aliphatic carboxylic acid (5.0 mmol,1.0 equiv.) in CH_2Cl_2 (50 mL), DMAP (20 mol%, 1.0 mmol) and EDCI (11.0 mmol, 2.2 equiv.) was added. The mixture was stirred at room temperature under air atmosphere until the reaction was complete as observed from TLC monitoring. The mixture was diluted with distilled water (50 mL) and the DCM layer was separated, dried over anhydrous Na_2SO_4 and concentrated. The solvent was removed and the residue was purified by silica gel column chromatography (PE /

EA as eluent) to give the corresponding compound (**b-74** – **b-80**).

Characterization data of substrates

(*1S,2R,5S*)-2-isopropyl-5-methylcyclohexyl 4-iodobenzoate (a-59):

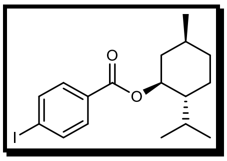

Synthesized by following **General Procedure 1** using L (-)-Menthol (781 mg, 5.0 mmol) and 4-Iodobenzoic acid (1240 mg, 5.0 mmol). Purified by flash column chromatography (PE / EA, 20:1, v/v) as colorless oil.

^{1}H NMR (400 MHz, Chloroform-*d*) δ 7.79 (d, *J* = 8.3 Hz, 2H), 7.32 (s, 2H), 6.17 (d, *J* = 7.9 Hz, 1H), 4.08 (tt, *J* = 12.0, 3.5 Hz, 1H), 3.63 – 3.45 (m, 1H), 2.04 (qd, *J* = 13.1, 12.4, 3.8 Hz, 3H), 1.84 (dt, *J* = 13.6, 4.6 Hz, 5H), 1.72 – 1.54 (m, 7H), 1.42 – 1.11 (m, 7H), 0.95 (tdd, *J* = 12.7, 10.8, 3.1 Hz, 2H). (*see spectrum*)

^{13}C NMR (101 MHz, Chloroform-*d*) δ 170.48, 154.09, 137.78, 136.40, 128.39, 97.24, 77.41, 77.09, 76.77, 57.58, 49.74, 32.36, 30.82, 26.23, 25.39, 25.25, 24.54. (*see spectrum*)

((*3aR,5R,5aS,8aS,8bR*)-2,2,7,7-tetramethyltetrahydro-5H-bis([1,3]dioxolo)[4,5-b:4',5'-d]pyran-5-yl)methyl 4-iodobenzoate (a-60):

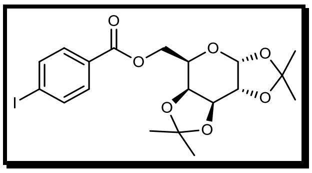

Synthesized by following **General Procedure 1** using 1,2:3,4-Di-O-Isopropylidene-α-D-Galactopyranose (1301 mg, 5.0 mmol) and 4-Iodobenzoic acid (1240 mg, 5.0 mmol). Purified by flash column chromatography (PE / EA, 7:1, v/v) as white solid.

^{1}H NMR (400 MHz, Chloroform-d) δ 7.83 – 7.72 (m, 4H), 4.70 – 4.59 (m, 2H), 4.42 (d, *J* = 2.6 Hz, 1H), 4.31 (d, *J* = 11.8 Hz, 1H), 4.25 (dd, *J* = 8.0, 1.7 Hz, 1H), 3.94 (dd, *J* = 13.0, 1.9 Hz, 1H), 3.79 (d, *J* = 13.0 Hz, 1H), 1.53 (s, 3H), 1.45 (s, 3H), 1.34 (s, 6H). (*see spectrum*)

^{13}C NMR (101 MHz, Chloroform-d) δ 165.55, 137.83, 131.25, 129.44, 109.23, 108.91, 101.63, 101.07, 77.43, 77.11, 76.79, 70.80, 70.63, 70.13, 65.58, 61.42, 26.57, 25.95, 25.54, 24.07. (*see spectrum*)

((*3aS,8aR,8bS*)-2,2,7,7-tetramethyltetrahydro-3aH-bis([1,3]dioxolo)[4,5-b:4',5'-d]pyran-3a-yl)methyl 4-iodobenzoate (a-61):

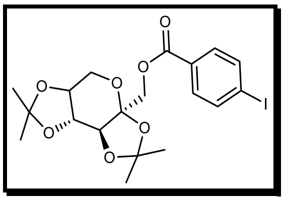

Synthesized by following **General Procedure 1** using Diacetonefructose (1301 mg, 5.0 mmol) and 4-Iodobenzoic acid (1240 mg, 5.0 mmol). Purified by flash column chromatography (PE / EA, 7:1, v/v) as white solid.

^{1}H NMR (400 MHz, Chloroform-*d*) δ 7.84 – 7.70 (m, 4H), 5.55 (d, *J* = 5.0 Hz, 1H), 4.64 (dd, *J* = 7.9, 2.5 Hz, 1H), 4.51 (dd, *J* = 11.5, 4.6 Hz, 1H), 4.41 (dd, *J* = 11.6, 7.7 Hz, 1H), 4.34 (dd, *J* = 5.0, 2.5 Hz, 1H), 4.30 (dd, *J* = 7.9, 1.9 Hz, 1H), 4.15 (ddd, *J* = 7.1, 4.7, 1.9 Hz, 1H), 1.50 (s, 3H), 1.47 (s, 3H), 1.35 (s, 3H), 1.33 (s, 3H). (*see spectrum*)

^{13}C NMR (101 MHz, Chloroform-*d*) δ 166.03, 137.79, 131.21, 129.58, 109.80, 108.88, 100.91, 96.37, 77.42, 77.10, 76.79, 71.18, 70.78, 70.55, 66.16, 64.22, 26.08, 26.04, 25.03, 24.55. (*see spectrum*)

(*3aS,4R,6R,6aS*)-6-((*R*)-2,2-dimethyl-1,3-dioxolan-4-yl)-2,2-dimethyltetrahydrofuro[3,4-d][1,3]dioxol-4-yl 4-iodobenzoate (a-62):

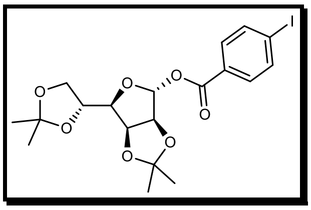

Synthesized by following **General Procedure 1** using Diacetone-d-Glucose (1301 mg, 5.0 mmol) and 4-Iodobenzoic acid (1240 mg, 5.0 mmol). Purified by flash column chromatography (PE / EA, 7:1, v/v) as white solid.

^{1}H NMR (400 MHz, Chloroform-*d*) δ 7.81 (d, *J* = 8.4 Hz, 2H), 7.70 (d, *J* = 8.6 Hz, 2H), 6.34 (s, 1H), 4.93 (dd, *J* = 5.9, 3.6 Hz, 1H), 4.85 (d, *J* = 5.9 Hz, 1H), 4.43 (ddd, *J* = 7.9,

6.1, 4.3 Hz, 1H), 4.13 – 4.08 (m, 2H), 4.04 (dd, *J* = 8.9, 4.3 Hz, 1H), 1.45 (s, 3H), 1.37 (d, *J* = 4.5 Hz, 6H). (see spectrum)

^{13}C NMR (101 MHz, Chloroform-*d*) δ 164.59, 137.92, 131.24, 129.01, 113.48, 109.44, 101.86, 101.52, 85.25, 82.73, 79.40, 77.42, 77.10, 76.78, 72.93, 66.88, 27.03, 26.02, 25.16, 24.73. (see spectrum)

(*5R,5aR,8aR,9R*)-8-oxo-9-(3,4,5-trimethoxyphenyl)-5,5a,6,8,8a,9-hexahydrofuro[3',4':6,7]naphtho[2,3-d][1,3]dioxol-5-yl 4-iodobenzoate (a-63):

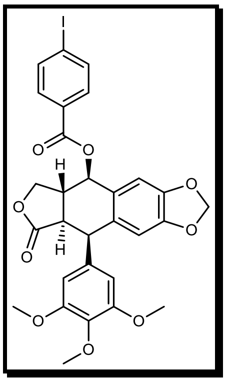

Synthesized by following **General Procedure 1** using Podophyllotoxin (2072 mg, 5.0 mmol) and 4-Iodobenzoic acid (1240 mg, 5.0 mmol). Purified by flash column chromatography (PE / EA, 5:1, v/v) as white solid.

^{1}H NMR (400 MHz, Chloroform-*d*) δ 7.84 (d, *J* = 8.6 Hz, 2H), 7.74 (d, *J* = 8.7 Hz, 2H), 6.83 (s, 1H), 6.58 (s, 1H), 6.43 (s, 2H), 6.09 (d, *J* = 8.5 Hz, 1H), 5.99 (d, *J* = 8.5 Hz, 2H), 4.64 (s, 1H), 4.50 – 4.37 (m, 1H), 4.30 (t, *J* = 8.5 Hz, 1H), 3.78 (dd, *J* = 12.2, 2.8 Hz, 9H), 3.10 – 2.87 (m, 2H). (see spectrum)

^{13}C NMR (101 MHz, Chloroform-*d*) δ 173.64, 166.47, 152.74, 148.36, 147.82, 138.15, 137.30, 134.83, 132.62, 131.07, 128.77, 128.19, 109.92, 108.20, 107.08, 101.80, 101.73, 77.44, 77.13, 76.81, 74.61, 71.50, 60.82, 56.21, 45.70, 43.82, 38.87. (see spectrum)

1,7,7-trimethylbicyclo[2.2.1]heptan-2-yl 4-iodobenzoate (a-64):

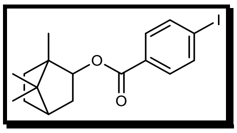

Synthesized by following **General Procedure 1** using Borneol (771 mg, 5.0 mmol) and

4-Iodobenzoic acid (1240 mg, 5.0 mmol). Purified by flash column chromatography (PE / EA, 20:1, v/v) as colorless oil.

^{1}H NMR (400 MHz, Chloroform-*d*) δ 7.82 – 7.74 (m, 4H), 5.10 (ddd, *J* = 10.0, 3.5, 2.1 Hz, 1H), 2.53 – 2.41 (m, 1H), 2.08 (ddd, *J* = 13.3, 9.4, 4.4 Hz, 1H), 1.86 – 1.73 (m, 2H), 1.41 (ddt, *J* = 14.4, 12.0, 2.2 Hz, 1H), 1.30 (ddd, *J* = 12.2, 9.6, 4.5 Hz, 1H), 1.13 – 1.08 (m, 1H), 0.96 (s, 3H), 0.91 (d, *J* = 4.6 Hz, 6H). (see spectrum)

^{13}C NMR (101 MHz, Chloroform-*d*) δ 166.37, 137.76, 131.04, 130.42, 100.51, 80.95, 77.41, 77.10, 76.78, 49.17, 47.97, 45.15, 45.02, 36.92, 28.14, 27.45, 19.78, 18.97, 13.68. (see spectrum)

1,3,3-trimethylbicyclo[2.2.1]heptan-2-yl 4-iodobenzoate (a-65):

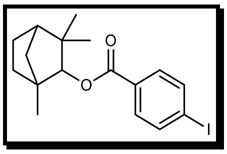

Synthesized by following **General Procedure 1** using Fenchyl Alcohol (771 mg, 5.0 mmol) and 4-Iodobenzoic acid (1240 mg, 5.0 mmol). Purified by flash column chromatography (PE / EA, 20:1, v/v) as colorless oil.

^{1}H NMR (400 MHz, Chloroform-*d*) δ 7.84 – 7.73 (m, 4H), 4.60 (d, *J* = 1.9 Hz, 1H), 1.93 – 1.85 (m, 1H), 1.81 – 1.72 (m, 2H), 1.66 (d, *J* = 9.9 Hz, 1H), 1.56 – 1.47 (m, 1H), 1.27 – 1.16 (m, 5H), 1.10 (s, 3H), 0.82 (d, *J* = 1.6 Hz, 3H). (see spectrum)

^{13}C NMR (101 MHz, Chloroform-*d*) δ 166.45, 137.81, 131.04, 130.22, 100.54, 87.07, 77.40, 77.08, 76.77, 48.67, 48.45, 41.50, 39.90, 29.80, 26.93, 25.95, 20.33, 19.54. (see spectrum)

3,7-dimethyloct-6-en-1-yl 4-iodobenzoate (a-66):

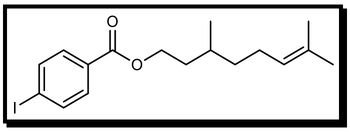

Synthesized by following **General Procedure 1** using Citronellol (781 mg, 5.0 mmol) and 4-Iodobenzoic acid (1240 mg, 5.0 mmol). Purified by flash column chromatography (PE / EA, 20:1, v/v) as colorless oil.

^{1}H NMR (400 MHz, Chloroform-*d*) δ 7.79 (d, *J* = 8.5 Hz, 2H), 7.73 (d, *J* = 8.6 Hz, 2H), 5.09 (td, *J* = 7.2, 3.6 Hz, 1H), 4.40 – 4.28 (m, 2H), 2.00 (hept, *J* = 7.4 Hz, 2H), 1.84 – 1.75 (m, 1H), 1.68 – 1.52 (m, 8H), 1.39 (ddt, *J* = 11.9, 9.5, 6.1 Hz, 1H), 1.28 – 1.20 (m, 1H), 0.96 (d, *J* = 6.3 Hz, 3H). (*see spectrum*)

^{13}C NMR (101 MHz, Chloroform-*d*) δ 166.22, 137.74, 131.49, 131.07, 130.04, 124.58, 100.65, 77.43, 77.12, 76.80, 63.85, 37.02, 35.49, 29.59, 25.80, 25.44, 19.57, 17.75. (*see spectrum*)

(*R*)-2,5,7,8-tetramethyl-2-((*4R,8R*)-4,8,12-trimethyltridecyl)chroman-6-yl 4-iodobenzoate (a-67):

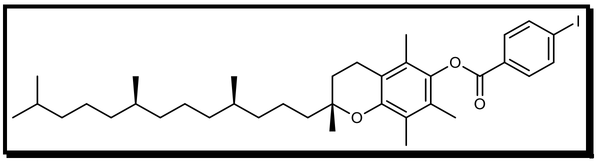

Synthesized by following **General Procedure 2** using DL-alpha-tocopherol (2154 mg, 5.0 mmol) and 4-Iodobenzoyl Chloride (1332 mg, 5.0 mmol). Purified by flash column chromatography (PE / EA, 10:1, v/v) as white solid.

^{1}H NMR (400 MHz, Chloroform-*d*) δ 7.97 (d, *J* = 8.4 Hz, 2H), 7.90 (d, *J* = 8.3 Hz, 2H), 2.64 (t, *J* = 6.8 Hz, 2H), 2.15 (s, 3H), 2.07 (s, 3H), 2.02 (s, 3H), 1.84 (tq, *J* = 19.2, 12.9, 9.9 Hz, 2H), 1.63 – 1.38 (m, 7H), 1.35 – 1.22 (m, 11H), 1.20 – 1.07 (m, 6H), 0.89 (t, *J* = 6.5 Hz, 12H). (*see spectrum*)

^{13}C NMR (101 MHz, Chloroform-*d*) δ 164.80, 149.67, 140.57, 138.07, 131.63, 129.19, 126.86, 125.10, 123.29, 117.60, 101.43, 75.20, 39.47, 37.64, 37.56, 37.51, 37.49, 37.39, 32.88, 28.07, 24.91, 24.55, 22.83, 22.74, 21.14, 20.73, 19.87, 19.80, 19.78, 19.75, 19.71, 13.13, 12.29, 11.97. (*see spectrum*)

(*7R,11R,E*)-3,7,11,15-tetramethylhexadec-2-en-1-yl 4-iodobenzoate (a-68):

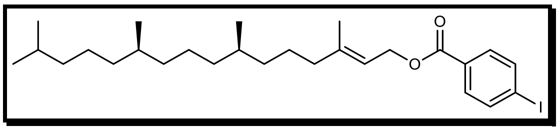

Synthesized by following **General Procedure 1** using (7R,11R,E)-3,7,11,15-Tetramethylhexadec-2-En-1-Ol (1483 mg, 5.0 mmol) and 4-Iodobenzoic acid (1240 mg, 5.0 mmol). Purified by flash column chromatography (petroleum ether: ethyl acetate,

20:1, v/v) as colorless oil.

^{1}H NMR (400 MHz, Chloroform-*d*) δ 7.80 – 7.73 (m, 4H), 5.44 (td, *J* = 7.2, 1.7 Hz, 1H), 4.83 (d, *J* = 7.1 Hz, 2H), 2.03 (t, *J* = 7.8 Hz, 2H), 1.75 (d, *J* = 1.3 Hz, 3H), 1.52 (dt, *J* = 13.2, 6.6 Hz, 1H), 1.47 – 1.35 (m, 4H), 1.26 (td, *J* = 9.3, 4.4 Hz, 8H), 1.16 – 1.03 (m, 6H), 0.85 (dd, *J* = 9.7, 6.4 Hz, 12H). (*see spectrum*)

^{13}C NMR (101 MHz, Chloroform-*d*) δ 166.20, 143.22, 137.70, 131.14, 130.07, 117.96, 100.62, 77.43, 77.11, 76.80, 62.22, 39.94, 39.44, 37.49, 37.43, 37.36, 36.67, 32.85, 32.72, 29.78, 28.05, 25.07, 24.88, 24.53, 22.81, 22.72, 19.83, 19.80, 16.55. (*see spectrum*)

(*E*)-4-(3,5-dimethoxystyryl)phenyl 4-iodobenzoate (a-69):

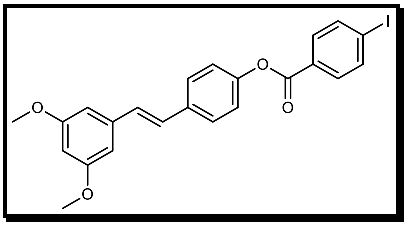

Synthesized by following **General Procedure 2** using trans-pterostilbene (1281 mg, 5.0 mmol) and 4-Iodobenzoyl Chloride (1332 mg, 5.0 mmol). Purified by flash column chromatography (PE / EA, 10:1, v/v) as white solid.

^{1}H NMR (400 MHz, Chloroform-*d*) δ 7.94 – 7.85 (m, 4H), 7.56 (d, *J* = 8.5 Hz, 2H), 7.21 (d, *J* = 8.5 Hz, 2H), 7.05 (q, *J* = 16.3 Hz, 2H), 6.72 – 6.63 (m, 2H), 6.41 (q, *J* = 1.9 Hz, 1H), 3.84 (s, 6H). (*see spectrum*)

^{13}C NMR (101 MHz, Chloroform-*d*) δ 164.77, 161.08, 150.26, 139.22, 138.06, 135.24, 131.60, 129.14, 128.19, 127.65, 121.91, 104.68, 101.73, 100.18, 77.39, 77.08, 76.76, 55.45. (*see spectrum*)

(*3S,8S,9S,10R,13R,14S,17R*)-10,13-dimethyl-17-((*R*)-6-methylheptan-2-yl)-2,3,4,7,8,9,10,11,12,13,14,15,16,17-tetradecahydro-1H-cyclopenta[a]phenanthren-3-yl 4-iodobenzoate (a-70):

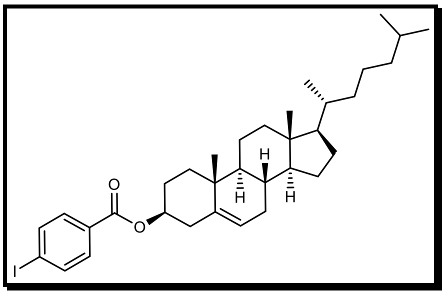

Synthesized by following **General Procedure 1** using Cholesterol (1933 mg, 5.0 mmol) and 4-Iodobenzoic acid (1240 mg, 5.0 mmol). Purified by flash column chromatography (PE / EA, 5:1, v/v) as white solid.

^{1}H NMR (400 MHz, Chloroform-*d*) δ 7.81 – 7.72 (m, 4H), 5.41 (dd, *J* = 5.2, 2.1 Hz, 1H), 4.84 (dtd, *J* = 12.3, 8.4, 4.4 Hz, 1H), 2.45 (d, *J* = 8.1 Hz, 2H), 2.05 – 1.66 (m, 6H), 1.63 – 1.44 (m, 7H), 1.43 – 0.94 (m, 19H), 0.92 (d, *J* = 6.5 Hz, 3H), 0.87 (dd, *J* = 6.6, 1.8 Hz, 6H), 0.69 (s, 3H). (*see spectrum*)

^{13}C NMR (101 MHz, Chloroform-*d*) δ 165.56, 139.57, 137.68, 131.10, 130.37, 122.98, 100.53, 77.41, 77.09, 76.77, 75.00, 56.75, 56.19, 50.08, 42.38, 39.79, 39.58, 38.22, 37.06, 36.70, 36.25, 35.87, 31.99, 31.93, 28.30, 28.08, 27.90, 24.36, 23.90, 22.90, 22.64, 21.11, 19.44, 18.79, 11.93. (*see spectrum*)

(*10S*,*13R*,*14R*,*17R*)-4,4,10,13,14-pentamethyl-17-((*R*)-6-methylhept-5-en-2-yl)-2,3,4,5,6,7,10,11,12,13,14,15,16,17-tetradecahydro-1H-cyclopenta[a]phenanthren-3-yl 4-iodobenzoate (a-71):

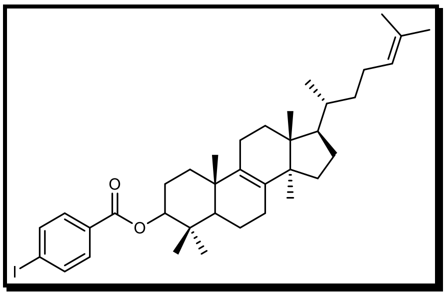

Synthesized by following **General Procedure 1** using Lanosterol (2134 mg, 5.0 mmol) and 4-Iodobenzoic acid (1240 mg, 5.0 mmol). Purified by flash column chromatography (PE / EA, 5:1, v/v) as white solid.

^{1}H NMR (400 MHz, Chloroform-*d*) δ 7.83 – 7.71 (m, 4H), 5.17 – 3.87 (m, 2H), 2.05 (dd, *J* = 15.1, 9.3 Hz, 4H), 1.97 – 1.89 (m, 1H), 1.89 – 1.65 (m, 8H), 1.64 – 1.08 (m, 15H), 1.03 (d, *J* = 9.3 Hz, 6H), 0.95 – 0.69 (m, 13H). (*see spectrum*)

^{13}C NMR (101 MHz, Chloroform-*d*) δ 165.82, 137.74, 134.66, 134.25, 131.07, 130.95, 130.54, 125.33, 100.48, 82.00, 77.43, 77.11, 76.79, 50.62, 50.57, 50.45, 49.89, 44.55, 39.60, 38.26, 37.00, 36.56, 36.43, 36.34, 35.33, 31.04, 30.91, 28.29, 28.16, 28.08, 26.45, 25.82, 25.00, 24.35, 24.29, 24.19, 22.92, 22.64, 21.12, 19.28, 18.81, 18.73, 18.22, 17.73, 16.89, 15.84. (*see spectrum*)

(*3S,8S,9S,10R,13R,14S,17R*)-17-((*2R,5S*)-5-ethyl-6-methylhept-3-en-2-yl)-10,13-dimethyl-2,3,4,7,8,9,10,11,12,13,14,15,16,17-tetradecahydro-1H-cyclopenta[a]phenanthren-3-yl 4-iodobenzoate (a-72):

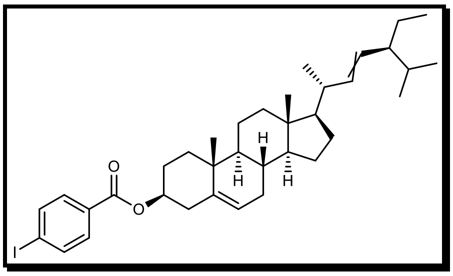

Synthesized by following **General Procedure 1** using stigmasterol (2064 mg, 5.0 mmol) and 4-Iodobenzoic acid (1240 mg, 5.0 mmol). Purified by flash column chromatography (PE / EA, 5:1, v/v) as white solid.

^{1}H NMR (400 MHz, Chloroform-*d*) δ 7.82 – 7.69 (m, 4H), 5.41 (d, *J* = 5.0 Hz, 1H), 5.16 (dd, *J* = 15.2, 8.6 Hz, 1H), 5.02 (dd, *J* = 15.1, 8.5 Hz, 1H), 4.84 (dtd, *J* = 12.3, 8.4, 4.5 Hz, 1H), 2.45 (d, *J* = 8.2 Hz, 2H), 1.97 (dddd, *J* = 36.0, 20.4, 8.8, 5.3 Hz, 5H), 1.79 – 1.66 (m, 2H), 1.61 – 1.39 (m, 8H), 1.31 – 1.14 (m, 6H), 1.10 – 0.95 (m, 9H), 0.83 (dd, *J* = 20.2, 6.4 Hz, 9H), 0.71 (s, 3H).(*see spectrum*)

^{13}C NMR (101 MHz, Chloroform-*d*) δ 165.53, 139.56, 138.38, 137.68, 131.10, 130.38, 129.37, 122.97, 100.52, 77.42, 77.10, 76.78, 74.99, 74.80, 56.85, 56.00, 51.31, 50.12, 42.28, 40.58, 39.70, 38.24, 37.08, 36.72, 31.99, 31.96, 31.93, 28.99, 27.91, 25.49, 24.44, 21.32, 21.18, 21.11, 19.44, 19.08, 12.34, 12.13. (*see spectrum*)

but-3-en-1-yl 2-(4-isobutylphenyl)propanoate (b-74)

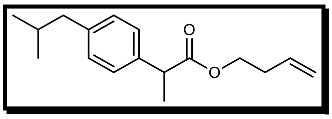

Synthesized by following **General Procedure 3** using 3-Buten-1-ol (360 mg, 5.0 mmol) and Ibuprofen (1030 mg, 5.0 mmol). Purified by flash column chromatography (PE / EA, 20:1, v/v) as colorless oil.

^{1}H NMR (400 MHz, Chloroform-*d*) δ 7.23 (d, *J* = 7.9 Hz, 2H), 7.11 (d, *J* = 7.9 Hz, 2H), 5.71 (ddt, *J* = 17.0, 10.3, 6.8 Hz, 1H), 5.12 – 4.96 (m, 2H), 4.14 (t, *J* = 6.7 Hz, 2H), 3.71 (q, *J* = 7.1 Hz, 1H), 2.47 (d, *J* = 7.2 Hz, 2H), 2.35 (q, *J* = 6.7 Hz, 2H), 1.51 (d, *J* = 7.2 Hz, 3H), 0.93 (d, *J* = 6.7 Hz, 6H). (see spectrum)

^{13}C NMR (101 MHz, Chloroform-*d*) δ 174.71, 140.53, 137.88, 133.98, 129.35, 127.28, 117.22, 77.49, 77.18, 76.86, 63.70, 45.24, 45.13, 33.13, 30.27, 22.46, 18.54. (see spectrum)

but-3-en-1-yl 2-(3-benzoylphenyl)propanoate (b-75)

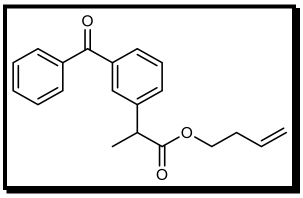

Synthesized by following **General Procedure 3** using 3-Buten-1-ol (360 mg, 5.0 mmol) and Ketoprofen (1271 mg, 5.0 mmol). Purified by flash column chromatography (PE / EA, 5:1, v/v) as colorless oil.

^{1}H NMR (400 MHz, Chloroform-*d*) δ 7.69 – 7.61 (m, 3H), 7.53 (dt, *J* = 7.6, 1.5 Hz, 1H), 7.45 – 7.38 (m, 2H), 7.30 (dt, *J* = 15.2, 7.7 Hz, 3H), 5.55 (ddt, *J* = 17.1, 10.3, 6.7 Hz, 1H), 4.92 – 4.79 (m, 2H), 3.99 (t, *J* = 6.7 Hz, 2H), 3.66 (q, *J* = 7.2 Hz, 1H), 2.19 (qt, *J* = 6.6, 1.4 Hz, 2H), 1.39 (d, *J* = 7.2 Hz, 3H). (see spectrum)

^{13}C NMR (101 MHz, Chloroform-*d*) δ 196.34, 173.92, 140.94, 137.91, 137.55, 133.81, 132.53, 131.62, 130.07, 129.26, 128.97, 128.55, 128.34, 117.32, 77.66, 77.34, 77.02, 63.85, 45.41, 33.05, 18.44. (see spectrum)

but-3-en-1-yl 2-(2-fluoro-[1,1'-biphenyl]-4-yl)propanoate (b-76)

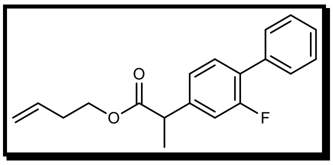

Synthesized by following **General Procedure 3** using 3-Buten-1-ol (360 mg, 5.0 mmol) and Flurbiprofen (620 mg, 5.0 mmol). Purified by flash column chromatography (PE / EA, 10:1, v/v) as colorless oil.

^{1}H NMR (400 MHz, Chloroform-*d*) δ 7.56 (d, *J* = 6.7 Hz, 2H), 7.48 – 7.36 (m, 4H), 7.19 – 7.13 (m, 2H), 5.82 – 5.67 (m, 1H), 5.12 – 5.01 (m, 2H), 4.18 (td, *J* = 6.7, 1.4 Hz, 2H), 3.77 (q, *J* = 7.2 Hz, 1H), 2.38 (qd, *J* = 6.8, 1.8 Hz, 2H), 1.55 (dd, *J* = 7.2, 1.4 Hz, 3H). (see spectrum)

^{13}C NMR (101 MHz, Chloroform-*d*) δ 173.98, 160.99, 158.53, 141.97, 141.90, 135.62, 133.88, 130.84, 130.80, 129.05, 129.02, 128.53, 127.93, 127.80, 127.74, 123.69, 123.66, 117.40, 115.51, 115.27, 77.45, 77.14, 76.82, 63.99, 45.14, 33.13, 18.39. (see spectrum)

^{19}F NMR (376 MHz, Chloroform-d) δ -117.69 (t, *J* = 9.9 Hz). (see spectrum)

but-3-en-1-yl (S)-2-(6-methoxynaphthalen-2-yl)propanoate (b-77)

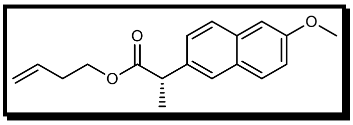

Synthesized by following **General Procedure 3** using 3-Buten-1-ol (360 mg, 5.0 mmol) and (S)-Naproxen (1150 mg, 5.0 mmol). Purified by flash column chromatography (PE / EA, 10:1, v/v) as white solid.

^{1}H NMR (400 MHz, Chloroform-*d*) δ 7.72 – 7.66 (m, 3H), 7.41 (dd, *J* = 8.5, 1.9 Hz, 1H), 7.17 – 7.10 (m, 2H), 5.71 (ddt, *J* = 17.0, 10.3, 6.8 Hz, 1H), 5.05 – 4.96 (m, 2H), 3.92 (s, 3H), 3.85 (q, *J* = 7.2 Hz, 1H), 2.33 (qt, *J* = 6.7, 1.3 Hz, 2H), 1.58 (d, *J* = 7.1 Hz, 3H). (see spectrum)

^{13}C NMR (101 MHz, Chloroform-*d*) δ 174.66, 157.70, 135.78, 133.94, 133.76, 129.33, 129.01, 127.13, 126.37, 126.03, 118.99, 117.24, 105.68, 77.40, 77.08, 76.77, 63.80, 55.36, 45.55, 33.10, 18.55. (see spectrum)

but-3-en-1-yl 2-(11-oxo-6,11-dihydrodibenzo[b,e]oxepin-2-yl)acetate (b-78)

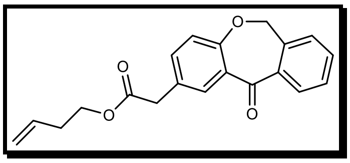

Synthesized by following **General Procedure 3** using 3-Buten-1-ol (360 mg, 5.0 mmol) and Isoxepac (1341 mg, 5.0 mmol). Purified by flash column chromatography (PE / EA, 5:1, v/v) as white solid.

^{1}H NMR (400 MHz, Chloroform-*d*) δ 8.10 (d, *J* = 2.4 Hz, 1H), 7.84 (dd, *J* = 7.6, 1.5 Hz, 1H), 7.47 (td, *J* = 7.4, 1.5 Hz, 1H), 7.43 – 7.35 (m, 2H), 7.32 – 7.24 (m, 1H), 6.98 (d, *J* = 8.4 Hz, 1H), 5.73 (ddt, *J* = 17.0, 10.3, 6.7 Hz, 1H), 5.17 – 4.95 (m, 4H), 4.13 (t, *J* = 6.7 Hz, 2H), 3.61 (s, 2H), 2.35 (qd, *J* = 6.7, 3.3 Hz, 2H). (see spectrum)

^{13}C NMR (101 MHz, Chloroform-*d*) δ 190.64, 171.32, 160.46, 140.42, 136.40, 135.63, 133.93, 132.77, 132.49, 129.46, 129.22, 127.91, 127.87, 125.15, 121.03, 117.38, 77.70, 77.38, 77.06, 73.55, 63.94, 40.18, 33.07. (see spectrum)

but-3-en-1-yl 2-(10-oxo-10,11-dihydrodibenzo[b,f]thiepin-2-yl)propanoate (b-79)

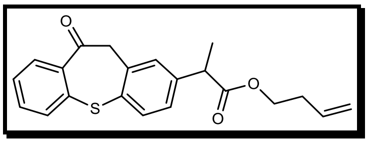

Synthesized by following **General Procedure 3** using 3-Buten-1-ol (360 mg, 5.0 mmol) and Zaltoprofen (1492 mg, 5.0 mmol). Purified by flash column chromatography (PE / EA, 5:1, v/v) as white solid.

^{1}H NMR (400 MHz, Chloroform-*d*) δ 8.19 (dd, *J* = 8.0, 1.7 Hz, 1H), 7.58 (d, *J* = 7.9 Hz, 2H), 7.43 – 7.36 (m, 2H), 7.29 (td, *J* = 7.7, 1.3 Hz, 1H), 7.14 (dd, *J* = 8.1, 2.0 Hz, 1H), 5.68 (ddt, *J* = 17.0, 10.3, 6.7 Hz, 1H), 5.05 – 4.92 (m, 2H), 4.35 (s, 2H), 4.11 (td, *J* = 6.7, 1.9 Hz, 2H), 3.70 (q, *J* = 7.2 Hz, 1H), 2.31 (qt, *J* = 6.7, 1.5 Hz, 2H), 1.47 (d, *J* = 7.2 Hz, 3H). (see spectrum)

^{13}C NMR (101 MHz, Chloroform-*d*) δ 191.31, 173.85, 142.77, 140.27, 137.93, 136.22, 133.82, 133.27, 132.54, 131.58, 131.48, 130.91, 128.76, 126.89, 126.45, 117.33, 77.46, 77.14, 76.82, 63.94, 51.13, 45.26, 33.04, 18.45. (see spectrum)

but-3-en-1-yl 2-(4-(4-chlorobenzoyl)phenoxy)-2-methylpropanoate (b-80)

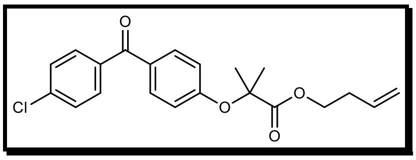

Synthesized by following **General Procedure 3** using 3-Buten-1-ol (360 mg, 5.0 mmol) and Fenofibric acid (1594 mg, 5.0 mmol). Purified by flash column chromatography (PE / EA, 5:1, v/v) as white solid.

^{1}H NMR (400 MHz, Chloroform-*d*) δ 7.74 – 7.68 (m, 4H), 7.46 – 7.41 (m, 2H), 6.88 – 6.84 (m, 2H), 5.67 (ddt, *J* = 17.0, 10.3, 6.7 Hz, 1H), 5.08 – 4.99 (m, 2H), 4.22 (t, *J* = 6.6 Hz, 2H), 2.36 (qt, *J* = 6.6, 1.4 Hz, 2H), 1.66 (s, 6H). (see spectrum)

^{13}C NMR (101 MHz, Chloroform-*d*) δ 194.24, 173.64, 159.72, 138.43, 136.47, 133.54, 132.02, 131.21, 130.46, 128.59, 117.58, 117.43, 79.48, 77.41, 77.09, 76.77, 64.64, 32.95, 25.51. (see spectrum)

Commercially available or preparation of receptor.

Alkenes **c-46**, **c-47** were commercially available substrates and used without further purification, except otherwise stated. Alkenes **c-48 - c-55**[3], **c-56 - c-58**[4], were known compounds and prepared according to reported methods.

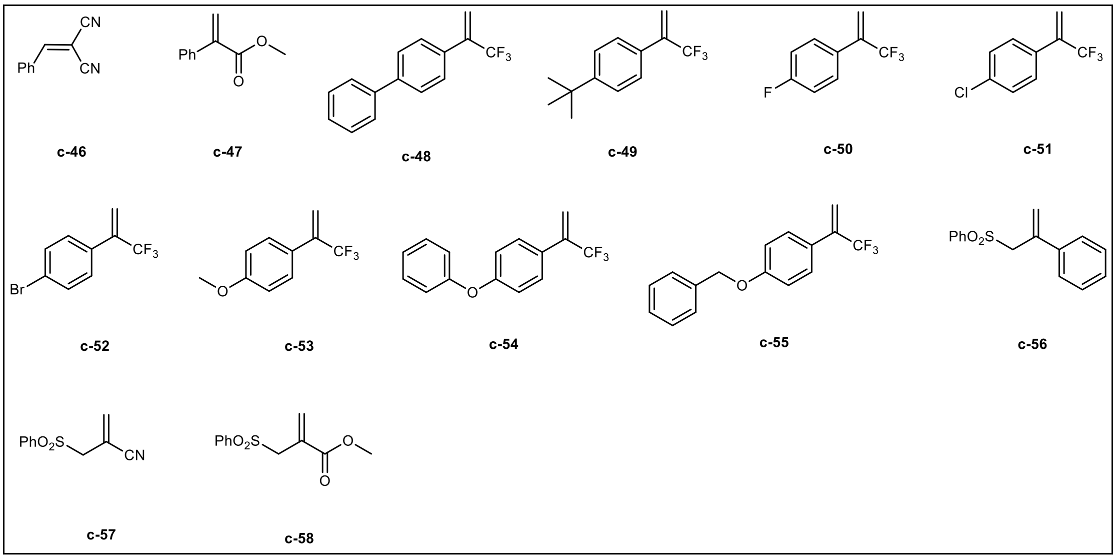

Supplementary Figure 2. Sources and preparation of radical acceptors

Unsuccessful substrates and mass balance analysis

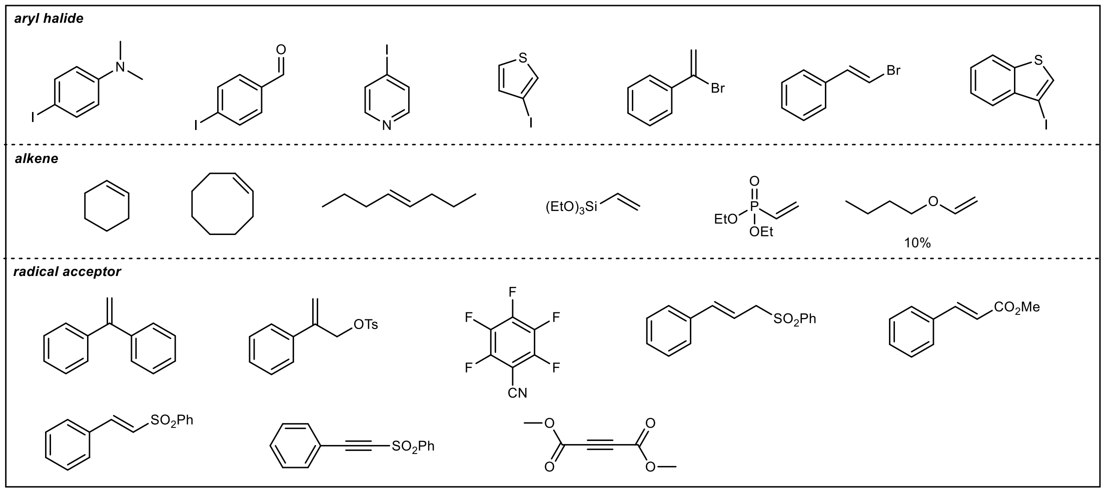

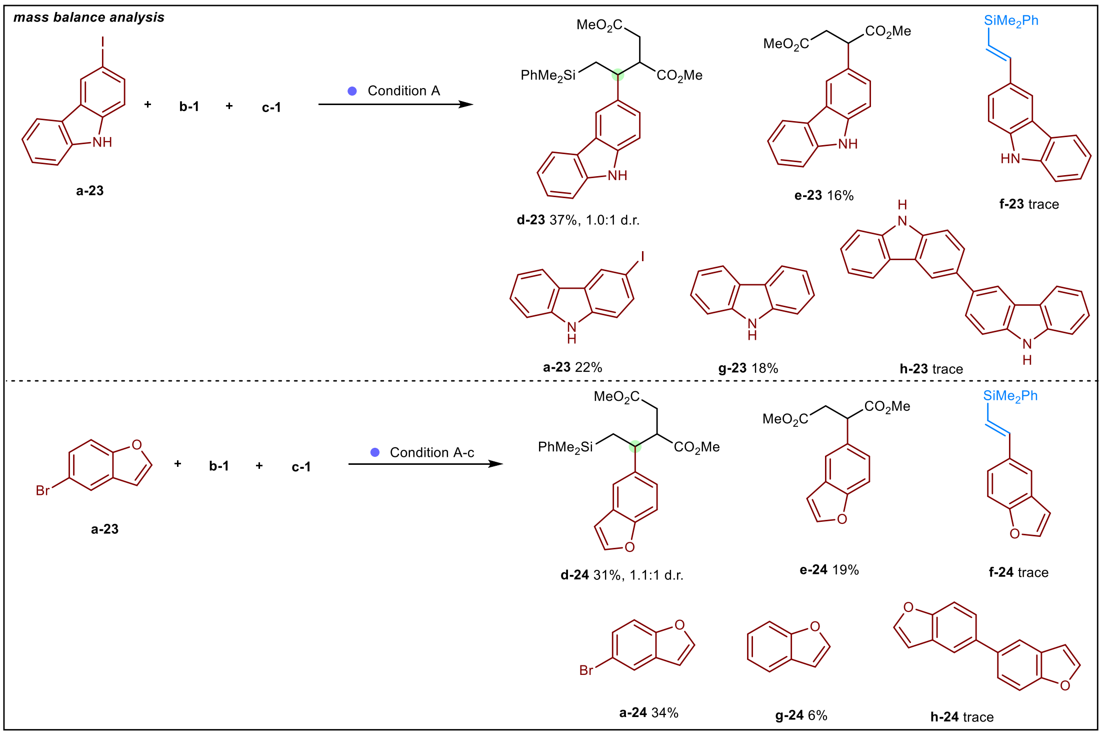

Supplementary Figure 3. Unsuccessful substrates

c) Optimization of the reaction conditions

Supplementary Table 1. Screening of solvents

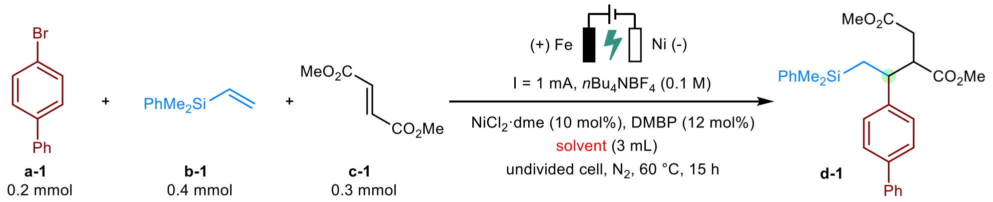

Entry	Solvent	Yield (%)[a]
**1**	DMF	22
**2**	DMA	trace
**3**	DMSO	34
**4**	NMP	28
**5**	NMP:1,4-Dioxane = 2:1 (*v*:*v*)	42
**6**	DMSO:1,4-Dioxane = 2:1 (*v*:*v*)	46

[a] Yield determined by ^{1}H NMR analysis using 1,3,5-trimethoxybenzene as internal standard.

Supplementary Table 2. Screening of substrate ratios

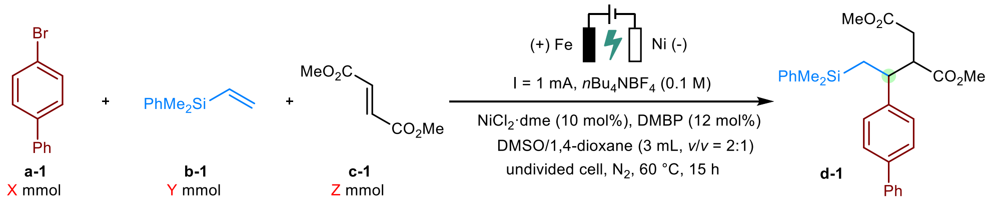

Entry	a / b / c (mmol)	Yield (%)[a]
**1**	0.2/0.4/0.3	50
**2**	0.2/0.3/0.4	43
**3**	0.2/0.4/0.4	40
**4**	0.3/0.2/0.4	37

[a] Yield determined by ^{1}H NMR analysis using 1,3,5-trimethoxybenzene as internal standard.

Supplementary Table 3. Screening of electrolytes

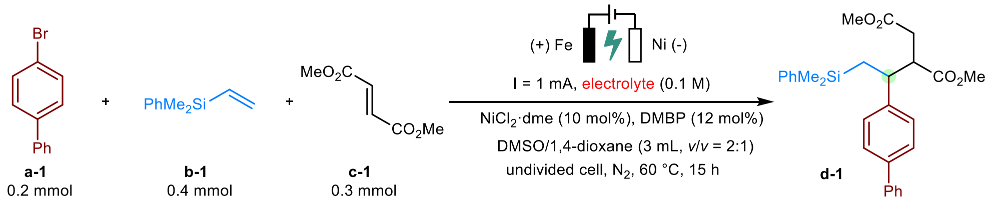

Entry	Electrolyte	Yield (%)[a]
**1**	*n*Bu_4NBF_4	42
**2**	*n*Bu_4NI	trace
**3**	*n*Bu_4NBr	30
**4**	*n*Bu_4NCl	33
**5**	*n*Bu_4NF	20

**6**	nBu$_4$NAc	N.D.
**7**	nBu$_4$NClO$_4$	trace
**8**	nBu$_4$NPF$_6$	10
**9**	KBF$_4$	trace
**10**	KPF$_6$	N.D.
**11**	Et$_4$NCl	26
**12**	Et$_4$NBF$_4$	trace
**13**	Et$_4$NClO$_4$	15
**14**	Me$_4$NCl	47

[a] Yield determined by ^{1}H NMR analysis using 1,3,5-trimethoxybenzene as internal standard. N.D. = no detected.

Supplementary Table 4. Screening of substrate types

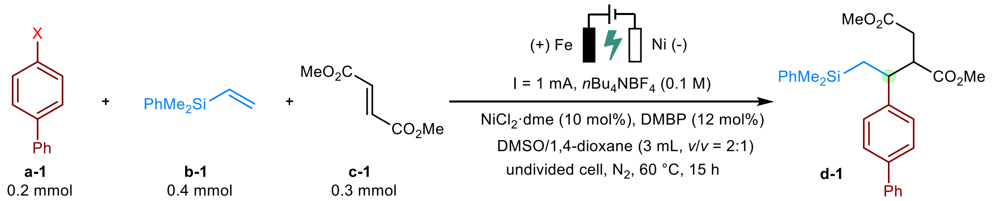

Entry	X	Yield (%)[a]
**1**	X=Cl	trace
**2**	X=Br	43
**3**	X=I	57
**4**	X=OTf	N.D.

[a] Yield determined by ^{1}H NMR analysis using 1,3,5-trimethoxybenzene as internal standard. N.D. = no detected.

Supplementary Table 5. Screening of additions.

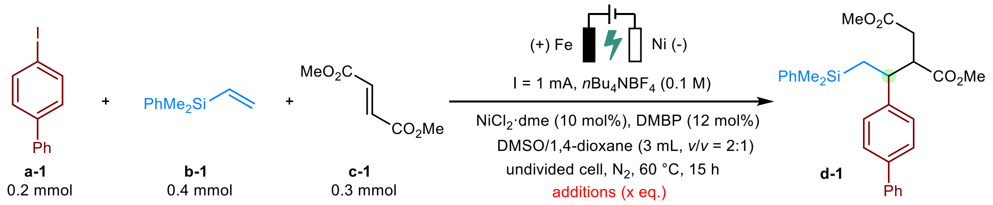

Entry	Additions (x eq)	Yield (%)[a]
**1**	Et$_3$N (1eq)	18
**2**	DIPEA (1eq)	27
**3**	iPr$_3$N(1eq)	35
**4**	H$_2$O(1eq)	13
**5**	ZnCl$_2$ (1eq)	trace
**6**	AlCl$_3$ (1eq)	19
**7**	CH$_3$OH (1eq)	27
**8**	CH$_3$CH$_3$OH (1eq)	39
**9**	IPA (1eq)	23
**10**	FeBr$_3$ (1eq)	36
**11**	FeCl$_2$ (1eq)	24

**12**	Na_2HPO_4 (1eq)	12
**13**	$MgCl_2$ (1eq)	16
**14**	KF (1eq)	15
**15**	LiCl (1eq)	25
**16**	Ferric citrate (1eq)	24
**17**	$Fe(acac)_2$ (1eq)	18
**18**	$Fe_2(SO_4)_3$ (1eq)	12
**19**	$FeCl_3$ (1eq)	71
**20**	$FeCl_3$ (0.75eq)	67
**21**	$FeCl_3$ (0.5eq)	54
**22**	$FeCl_3$ (0.25eq)	45

[a] Yield determined by ^{1}H NMR analysis using 1,3,5-trimethoxybenzene as internal standard.

Supplementary Table 6. Control experiments.

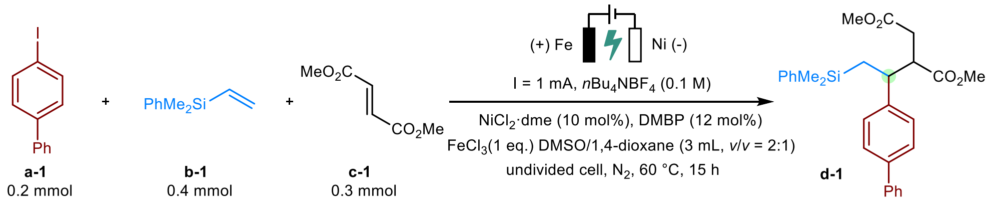

**Entry**	**Variations from standard conditions**	**Yield (%)[a]**
**1**	$NiCl_2$·dme (7%)	65
**2**	$NiCl_2$·dme (5%)	63
**3**	without $NiCl_2$·dme	37
**4**	without DMBP	45
**5**	Without current	N.R.
**6**	without hot	13
**7**	Without $NiCl_2$·dme and DMBP	47
**8**	Without $NiCl_2$·dme and DMBP, within $FeCl_3$(0.5eq)	32
**9**	Without $NiCl_2$·dme and DMBP, within $FeCl_3$(0.2eq)	18
**10**	Without $NiCl_2$·dme, DMBP and $FeCl_3$	trace

[a] Yield determined by ^{1}H NMR analysis using 1,3,5-trimethoxybenzene as internal standard. N.R. = no reaction.

Supplementary Table 7. Screening of ligands.

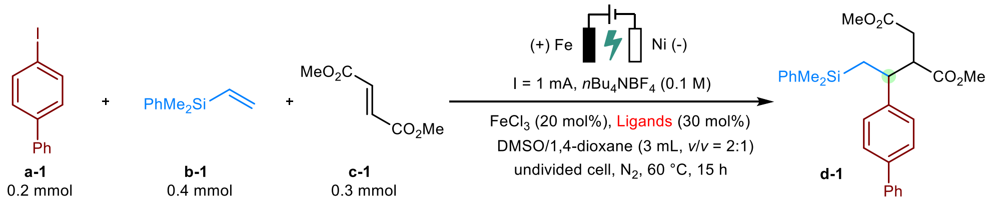

**Entry**	**Ligand**	**Yield (%)[a]**
**1**	TMHD	69
**2**	DMEDA	trace
**3**	DPPE	N.D.
**4**	DPPBz	N.D.

**5**	Cy_3P	42
**6**	R-BINAP	trace
**7**	DPPF	N.D.
**8**	DMBP	trace
**9**	dmbpy	N.D.
**10**	2,6-Lutidine	21
**11**[c]	O-phen	trace
**12**[d]	Neocuproine	trace
**13**	1,3-BDP	13

[a] Yield determined by ^{1}H NMR analysis using 1,3,5-trimethoxybenzene as internal standard. N.D. = no detected.

Supplementary Table 8. Screening of currents and times

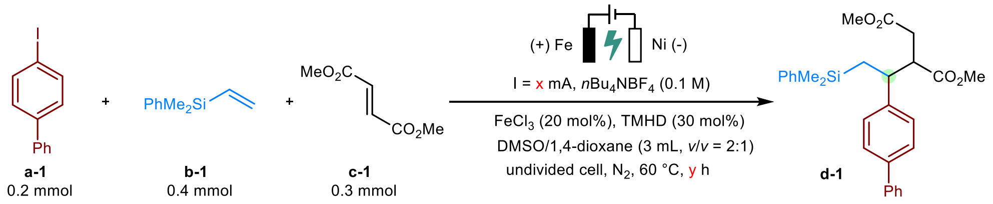

**Entry**	**x**	**y**	**Yield (%)**[a]
**1**	1	10	54
**2**	1	20	82
**3**	2	10	74
**4**	2	12	85

[a] Yield determined by ^{1}H NMR analysis using 1,3,5-trimethoxybenzene as internal standard.

Supplementary Table 9. Screening of base

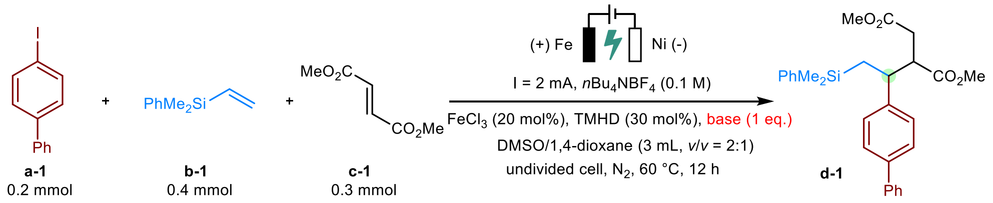

**Entry**	**Base**	**Yield (%)**[a]
**1**	-	85
**2**	iPr_3N	N.D.
**3**	DIPEA	34
**4**	$NaHCO_3$	43
**5**	$KHCO_3$	26
**6**	Na_2HPO_4	52
**7**	K_2HPO_4	66
**8**	Na_2CO_3	25
**9**	K_2CO_3	21
**10**	CS_2CO_3	trace
**11**	K_3PO_4	27

[a] Yield determined by ^1H NMR analysis using 1,3,5-trimethoxybenzene as internal standard. N.D. = no detected.

Supplementary Table 10. Screening of electrode materials and catalysts

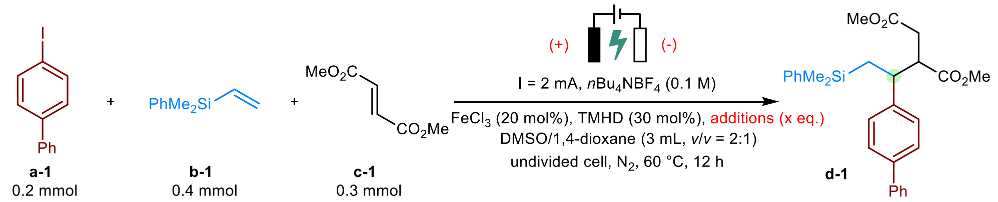

Entry	electrode	Additions	Yield (%)[a]
**1**	Fe/Fe	$FeCl_3$(99.99%, 20%)	N.D.
**2**	Fe/Al	$FeCl_3$(99.99%, 20%)	N.D.
**3**	Fe/Cu	$FeCl_3$(99.99%, 20%)	N.D.
**4**	Fe/CF	$FeCl_3$(99.99%, 20%)	N.D.
**5**	Fe/304	$FeCl_3$(99.99%, 20%)	30
**6**	Fe/Ni	$FeCl_3$(99.99%, 20%)	55
**7**	Fe/Co	$FeCl_3$(99.99%, 20%)	N.D.
**8**	Fe/Nb	$FeCl_3$(99.99%, 20%)	N.D.
**9**	Fe/Cr	$FeCl_3$(99.99%, 20%)	N.D.
**10**	Fe/Sn	$FeCl_3$(99.99%, 20%)	N.D.
**11**	Fe/C	$FeCl_3$(99.99%, 20%)	N.D.
**12**	Fe/Mg	$FeCl_3$(99.99%, 20%)	N.D.
**13**	Fe/Sb	$FeCl_3$(99.99%, 20%)	N.D.
**14**	Fe/Pt	$FeCl_3$(99.99%, 20%)	N.D.
**15**	Fe/Fe	$FeCl_3$(99.99%, 20%), $NiCl_2$(1%)	69
**16**	Fe/Fe	$NiCl_2$(1%)	28
**17**	Fe/Ni	$NiCl_2$(1%)	23
**18**	Mg/Ni	-	N.D.
**19**	Mg/Ni	$FeCl_3$(20%)	13
**20**	Zn/Ni	-	N.D.
**21**	Zn/Ni	$FeCl_3$(20%)	11

[a] Yield determined by ^1H NMR analysis using 1,3,5-trimethoxybenzene as internal standard. N.D. = no detected.

Supplementary Table 11. Ligand-controlled diastereoselectivity and yield

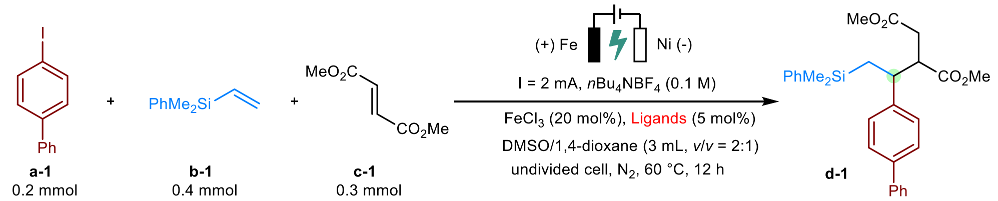

Entry	Ligand	Diastereoselectivity	Yield (%)[a]
**1**	L1	-	trace
**2**	L2	-	trace
**3**	L3	-	N.D.
**4**	L4	-	trace

**5**	L5	-	trace
**6**	L6	-	N.D.
**7**	L7	-	N.D.
**8**	L8	-	trace
**9**	L9	-	N.D.
**10**	L10	-	trace
**11**	L11	1.1:1	12
**12**	L12	-	trace
**13**	L13	1.2:1	13
**14**	L14	1.1:1	23
**15**	L15	-	N.D.
**16**	L16	-	N.D.
**17**	L17	-	trace
**18**	L18	-	N.D.
**19**	L19	-	trace
**20**	L20	-	trace
**21**	L21	-	trace
**22**	L22	1.2:1	20
**23**	L23	1.1:1	25
**24**	PPh_3	1.2:1	32
**25**	P-(Ph-4OMe)$_3$	1.3:1	16

[a] Yield determined by ^{1}H NMR analysis using 1,3,5-trimethoxybenzene as internal standard. N.D. = no detected.

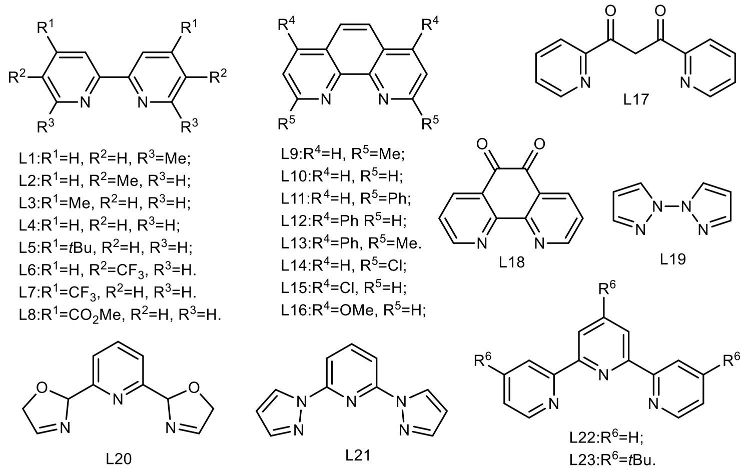

Supplementary Figure 4. Optimization of the ligands

Supplementary Table 12. Screening of Lewis acid

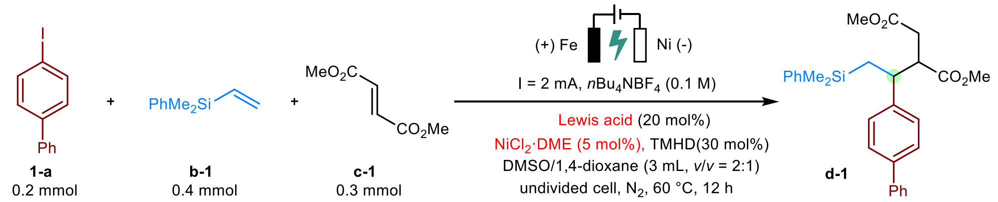

Entry	Lewis acid	$NiCl_2$·DME	Yield (%)[a]

**1**	$ZnCl_2$	-/5 mol%	N.D./trace
**2**	$BiCl_3$	-/5 mol%	N.D./trace
**3**	$SdCl_3$	-/5 mol%	N.D./5
**4**	$NdCl_3$	-/5 mol%	N.D./6
**5**	YCl_3	-/5 mol%	N.D./5
**6**	$AlCl_3$	-/5 mol%	N.D./trace
**7**	$GaCl_3$	-/5 mol%	N.D./trace
**8**	$DyCl_3$	-/5 mol%	N.D./12
**9**	$LaCl_3$	-/5 mol%	N.D./11
**10**	$CrCl_3$	-/5 mol%	N.D./12
**11**	$ScCl_3$	-/5 mol%	N.D./trace
**12**	$Er(OTf)_3$	-/5 mol%	N.D./N.D.
**13**	$Y(OTf)_3$	-/5 mol%	N.D./N.D.
**14**	$La(OTf)_3$	-/5 mol%	N.D./trace

[a] Yield determined by ^{1}H NMR analysis using 1,3,5-trimethoxybenzene as internal standard. N.D. = no detected.

d) General procedure for the 1,1-difunctionalization of alkenes

General procedure A

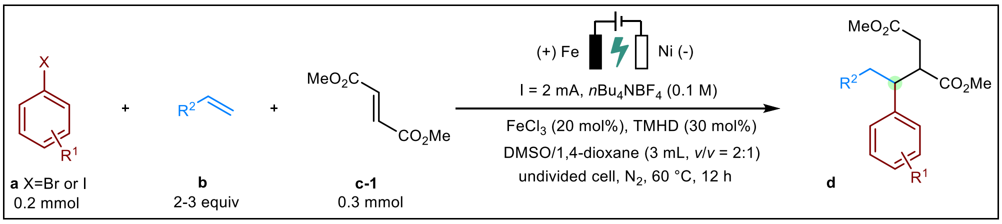

An oven dried 8 mL reaction vial with a magneton, a Fe anode, and a Ni cathode was added the substrate **a** (0.2 mmol, 1.0 equiv.), **b** (0.4 mmol, 2.0 equiv.) and **c-1** (0.3 mmol, 1.5 equiv.) and electrolyte nBu$_4$NBF$_4$ (98.8 mg, 0.1M), followed by weighting $FeCl_3$ (6.5 mg, 20 mol%) and **2,2,6,6-Tetramethyl-3,5-Heptanedione** (**TMHD**, 11.0 mg, 30 mol%), 3.0 mL solvent (Extra Dry, DMSO:1,4-Dioxane=2:1). The distance of electrodes was approximately 0.5 cm. Weighing of all drugs and assembly of reaction units are done in a nitrogen-filled glove box. The flask was then removed from the glove box (**Supplementary Figure 5**). The constant current (2.0 mA) electrolysis was then performed at 60 °C under N_2 atmosphere with stirring for 12 h, 4.5 F/mol. Upon completion, the reaction mixture was diluted with EtOAc and washed with NH_4Cl saturated solution (3 x equal volume) for three times. The combined organic layer was dried over anhydrous Na_2SO_4, and the solvent was then removed under reduced pressure. The resulting mixture was purified by column chromatography on silica gel (eluted with PE / EtOAc = 50:1 to 3:1, v/v) to afford the desired products **d-1** – **d-31**, **d-59** – **d-72**.

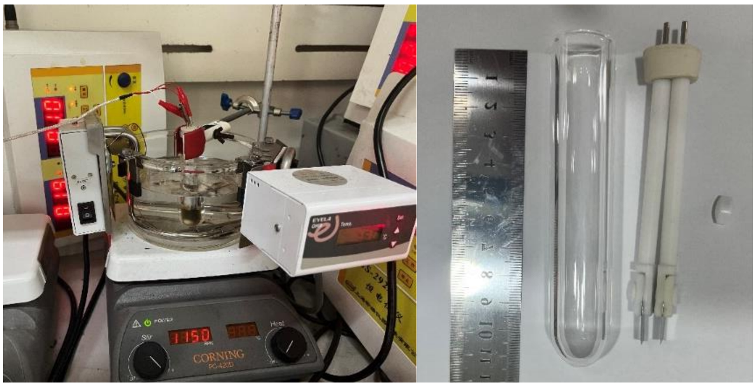

Supplementary Figure 5. Reaction setup for general electrochemical reactions.

General procedure B

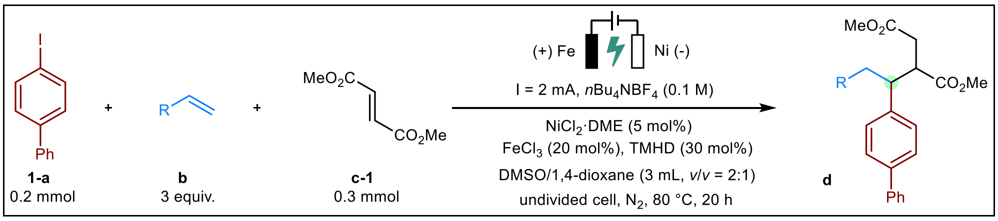

An oven dried 8 mL reaction vial with a magneton, a Fe anode, and a Ni cathode was added the substrate **a-1** (0.2 mmol, 1.0 equiv.), **b** (0.6 mmol, 3.0 equiv.) and **c-1** (0.3 mmol, 1.5 equiv.) and electrolyte *n*Bu_4NBF_4 (98.8 mg, 0.1M), followed by weighting $FeCl_3$ (6.5 mg, 20 mol%), TMHD (11.0 mg, 30 mol%) and $NiCl_2$ DME (2.2 mg, 5 mol%), 3.0 mL solvent (Extra Dry, DMSO:1,4-Dioxane=2:1). The distance of electrodes was approximately 0.5 cm. Weighing of all drugs and assembly of reaction units are done in a nitrogen-filled glove box. The flask was then removed from the glove box (**Supplementary Figure 5**). The constant current (2.0 mA) electrolysis was then performed at 80 °C under N_2 atmosphere with stirring for 20 h, 7.5 F/mol. Upon completion, the reaction mixture was diluted with EtOAc and washed with NH_4Cl saturated solution (3 x equal volume) for three times. The combined organic layer was dried over anhydrous Na_2SO_4, and the solvent was then removed under reduced pressure. The resulting mixture was purified by column chromatography on silica gel (eluted with PE / EA = 50:1 to 3:1, v/v) to afford the desired products **d-32 – d-46, d-73 – d-80**.

General procedure C

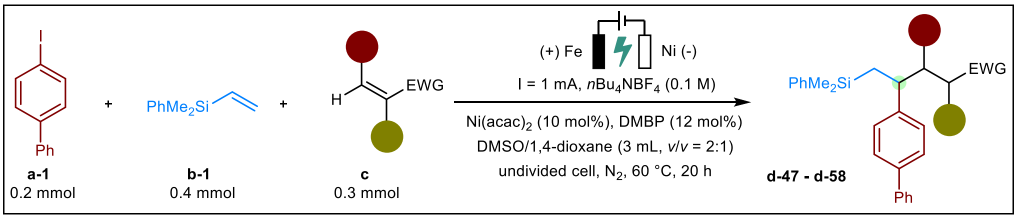

An oven dried 8 mL reaction vial with a magneton, a Fe anode, and a Ni foam cathode was added the substrate **a-1** (0.2 mmol, 1.0 equiv.), **b-1** (0.4 mmol, 2.0 equiv.) and **c** (0.3 mmol, 1.5 equiv.) and electrolyte *n*Bu_4NBF_4 (98.8 mg, 0.1M), followed by weighting $Ni(acac)_2$ (5.1 mg, 10 mol%), DMBP (4.4 mg, 12 mol%), 3.0 mL solvent

(Extra Dry, DMSO:1,4-Dioxane=2:1). The distance of electrodes was approximately 0.5 cm. Weighing of all drugs and assembly of reaction units are done in a nitrogen-filled glove box. The flask was then removed from the glove box (**Supplementary Figure 5**). The constant current (1.0 mA) electrolysis was then performed at 60 °C under N_2 atmosphere with stirring for 20 h, 3.7 F/mol. Upon completion, the reaction mixture was diluted with EtOAc and washed with NH_4Cl saturated solution (3 x equal volume) for three times. The combined organic layer was dried over anhydrous Na_2SO_4, and the solvent was then removed under reduced pressure. The resulting mixture was purified by column chromatography on silica gel (eluted with PE / EA = 100 to 3:1, v/v) to afford the desired products **d-47 – d-58**.

General procedure D

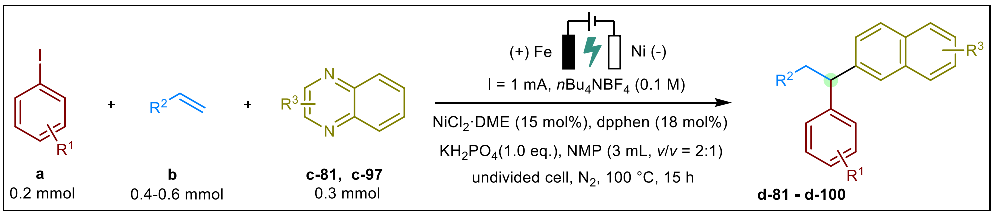

An oven dried 8 mL reaction vial with a magneton, a Fe anode, and a Ni foam cathode was added the substrate **a** (0.2 mmol, 1.0 equiv.), **b** (0.4 – 0.6 mmol, 2.0 – 3.0 equiv.) and **c-81, c-97** (0.3 mmol, 1.5 equiv.) and electrolyte *n*Bu_4NBF_4 (98.8 mg, 0.1M), followed by weighting $NiCl_2$·DME (6.6 mg, 15 mol%), 2,9-diphenyl-1,10-phenanthroline (**dpphen**, 12.0 mg, 18 mol%) and KH_2PO_4 (0.2 mmol, 27.2 mg), 3.0 mL solvent (Extra Dry, NMP). The distance of electrodes was approximately 0.5 cm. Weighing of all drugs and assembly of reaction units are done in a nitrogen-filled glove box. The flask was then removed from the glove box (**Supplementary Figure 5**). The constant current (1.0 mA) electrolysis was then performed at 100 °C under N_2 atmosphere with stirring for 15 h, 2.8 F/mol. Upon completion, the reaction mixture was diluted with EtOAc and washed with NH_4Cl saturated solution (3 x equal volume) for three times. The combined organic layer was dried over anhydrous Na_2SO_4, and the solvent was then removed under reduced pressure. The resulting mixture was purified by column chromatography on silica gel (eluted with PE / EA = 30:1 to 10:1, v/v) to afford the desired products **d-81 – d-100**.

e) Mechanistic experiments.

(1) Conventional zinc reductant control experiment

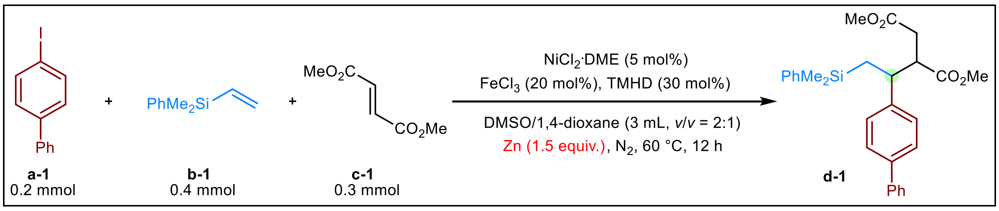

The first control experiments were performed in an 8 mL reaction vial equipped with a stir bar were immersed in the reaction mixture containing 4-iodobiphenyl (**a-1**, 0.2 mmol, 56.0 mg), dimethylphenylvinylsilane (**b-1**, 0.4 mmol, 64.8 mg), dimethyl fumarate (**c-1**, 0.3 mmol, 43.2 mg), $FeCl_3$ (6.5 mg, 20 mol%) and TMHD (11.0 mg, 30 mol%), $NiCl_2$.DME (2.2 mg, 5 mol%), Zn (19.6 mg, 1.5 equiv.). A total of 3.0 mL of anhydrous solvent (DMSO:1,4-dioxane = 2:1, v/v) was added to the system Two distinct additive systems were sequentially tested:

(a) no;

(b) without $FeCl_3$;

(c) without $NiCl_2$·DME.

All reagent handling and cell assembly were performed in a nitrogen-filled glovebox. The assembled reactor was then transferred to an external system, and constant-current electrolysis (2.0 mA) was carried out at 60 °C under a nitrogen atmosphere with vigorous stirring for 12 hours. Product analysis revealed: **(a) d-1** was formed in 12% yield; **(b)** only trace amounts of **d-1** were observed; **(c)** no reaction was initiated.

(2) Criticality evaluation of iron (Fe)

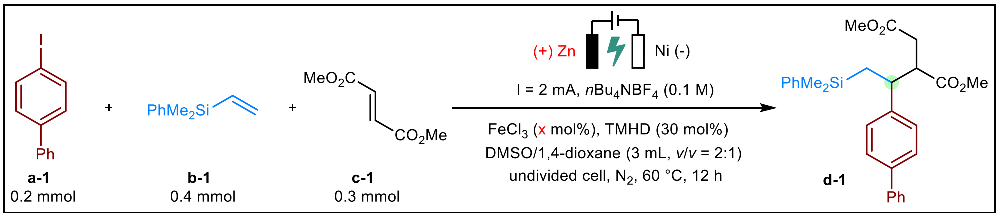

The deuterium-labelled experiments were performed in an 8 mL reaction vial equipped with a stir bar. An zinc plate anode (10 × 10 × 0.3 mm) and a nickel plate cathode (10 × 10 × 0.15 mm) were immersed in the reaction mixture containing 4-iodobiphenyl (**a-**

1, 0.2 mmol, 56.0 mg), dimethylphenylvinylsilane (**b-1**, 0.4 mmol, 64.8 mg), dimethyl fumarate (**c-1**, 0.3 mmol, 43.2 mg), $FeCl_3$ (x mol%) and TMHD (11.0 mg, 30 mol%) and electrolyte *n*Bu_4NBF_4 (98.9 mg, 0.1M). A total of 3.0 mL of anhydrous solvent (DMSO:1,4-dioxane = 2:1, v/v) was added to the system Two distinct additive systems were sequentially tested:

(a) without $FeCl_3$;

(b) $FeCl_3$ (20 mol%);

(c) $FeCl_3$ (100 mol%);

(b) using $FeCl_2$ (100 mol%) instead of $FeCl_3$.

All reagent handling and cell assembly were performed in a nitrogen-filled glovebox. The assembled reactor was then transferred to an external system, and constant-current electrolysis (2.0 mA) was carried out at 60 °C under a nitrogen atmosphere with vigorous stirring for 12 hours. Product analysis revealed: **(a)** no reaction was initiated; **(b)** **d-1** was formed in 12% yield; **(c)** **d-1** was formed in 33% yield; **(d)** No amounts of d-1 were observed.

(3) The divided-cell experiment

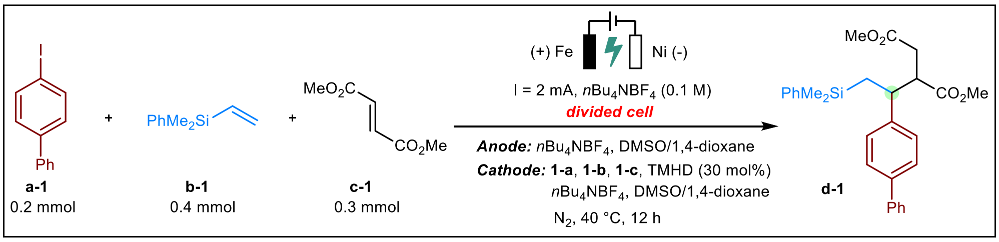

A divided-cell electrolytic setup was employed as follows: The reaction was conducted in a divided-cell electrolytic setup. The cathode compartment was charged with 4-iodobiphenyl (**a-1**, 0.2 mmol, 56.0 mg), dimethylphenylvinylsilane (**b-1**, 0.4 mmol, 64.8 mg), dimethyl fumarate (**c-1**, 0.3 mmol, 43.2 mg), tetrabutylammonium tetrafluoroborate (*n*Bu_4NBF_4, 164.6 mg, 0.1 M), and 5.0 mL of anhydrous mixed solvent (DMSO:1,4-dioxane = 2:1, v/v). The anode compartment contained an equivalent amount of electrolyte (*n*Bu_4NBF_4, 164.6 mg) and 5.0 mL of the same solvent. Subsequently, the following reagents were added to the cathode compartment under

three distinct conditions:

(a) 5.0 mL solvent only (no additives);

(b) $FeCl_3$ (6.5 mg, 20 mol%), TMHD (11.0 mg, 30 mol%), and 5.0 mL solvent;

(c) $FeCl_3$ (32.4 mg, 1.0 equiv.), TMHD (55.2 mg, 1.5 equiv.), and 5.0 mL solvent. A nickel plate (10 × 10 × 0.15 mm) and an iron plate (10 × 10 × 0.3 mm) served as the cathode and anode, respectively. All reagent handling and cell assembly were performed in a nitrogen-filled glovebox. The assembled reactor was then transferred to an external system, and constant-current electrolysis (2.0 mA) was carried out at 40 °C under a nitrogen atmosphere with vigorous stirring for 12 hours. The results revealed that: **(a)** no product **d-1** was detected; **(b)** only trace amounts of **d-1** were observed; **(c)** d-1 was obtained in 30% yield.

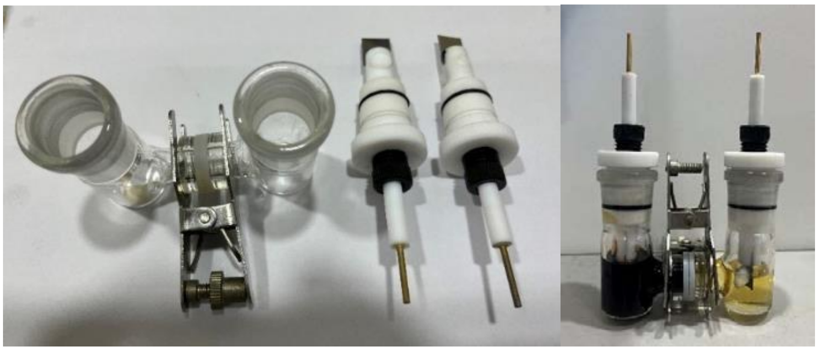

Supplementary Figure 6. Reaction setup for divided-cell experiment.

(4) ICP detection experiment

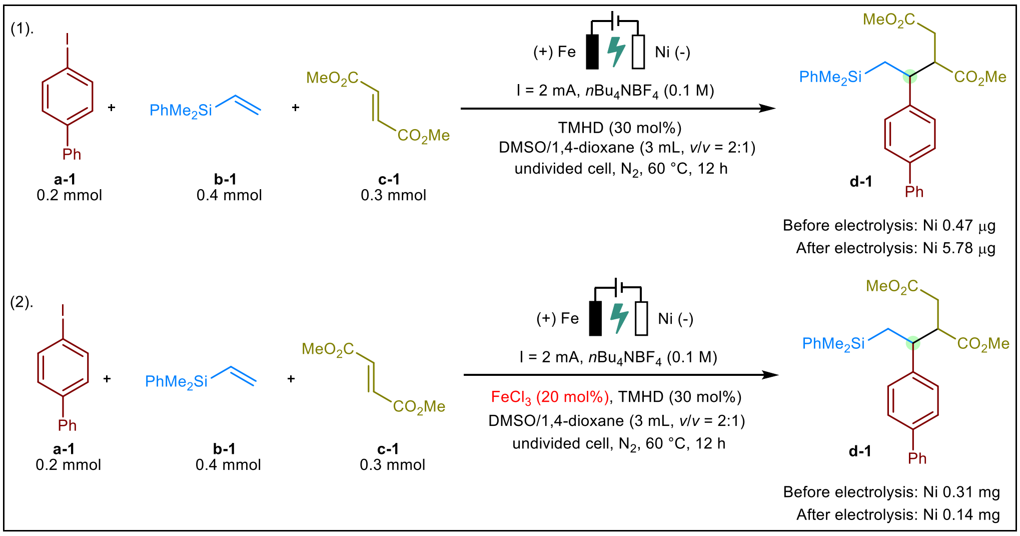

The radical-trapping experiments were performed in an 8 mL reaction vial equipped with a stir bar. An iron plate anode (10 × 10 × 0.3 mm) and a nickel plate cathode (10 × 10 × 0.15 mm) were immersed in the reaction mixture containing 4-iodobiphenyl (**a-1**, 0.2 mmol, 56.0 mg), dimethylphenylvinylsilane (**b-1**, 0.4 mmol, 64.8 mg), dimethyl fumarate (**c-1**, 0.3 mmol, 43.2 mg), TMHD (11.0 g, 30 mol%) and electrolyte *n*Bu_4NBF_4 (98.9 mg, 0.1M). A total of 3.0 mL of anhydrous solvent (DMSO:1,4-dioxane = 2:1, v/v) was added to the system Two distinct additive systems were sequentially tested:

(a) without current electrolysis, and the mixture was stirred for 5 min at 60 °C;

(b) The mixture was stirred at 60 °C under constant-current electrolysis (2.0 mA) for 12 hours;

(c) $FeCl_3$ (6.5 mg, 20 mol%), without current electrolysis and the mixture was stirred for 5 min at 60 °C;

(d) $FeCl_3$ (6.5 mg, 20 mol%) and, and the mixture was stirred at 60 °C under constant-current electrolysis (2.0 mA) for 12 hours.

The nickel leaching efficiency was quantified as follows: (1) The reaction mixture was filtered through a 0.22 μm PTFE membrane filter; (2) A 1 mL aliquot of the filtrate was digested with concentrated HNO_3 and H_2O_2 (3:1, v/v) at 95 °C for 2 h; (3) The digested sample was diluted to 10 mL with ultrapure water and analyzed by ICP-MS (Agilent 7850) with external calibration. Product analysis revealed: **(a)** 0.47 μg; **(b)** 5.78 μg; **(c)** 0.31 mg; **(d)** 0.14 mg.

(5) Cyclic Voltammetry Studies

Substrate redox potentials were determined by cyclic voltammetry (CV). Measurements were performed on a CHI 760E potentiostat using a glassy carbon working electrode, a platinum wire counter electrode, and a silver/silver chloride electrode (Ag/AgCl) reference. Samples contained 0.1 mmol substrate dissolved in 10 mL of anhydrous, degassed DMSO:1,4-dioxane (2:1, v/v) with 0.1 M *n*Bu_4NBF_4 as supporting electrolyte. CV were recorded at a scan rate of 0.1 V s^{-1}, and potentials were

referenced to Ag/AgCl using peak potentials (E_p). Specific test groups included:

(a) Blank, 4-iodobiphenyl(**a-1**), dimethylphenylvinylsilane(**b-1**), dimethyl fumarate(**c-1**);

(b) Dimethyl fumarate(**c-1**), benzalmalononitrile (**c-46**), methyl 2-phenylacrylate (**c-47**), (3,3,3-trifluoroprop-1-en-2-yl)benzene (**c-48**), [(2-phenyl-2-propenyl)sulfonyl]benzene (**c-56**), quinoxaline (**c-81**);

(c) Blank, dimethyl fumarate(**c-1**), $FeCl_3$, $FeCl_3$/dimethyl fumarate, $NiCl_2$/DMHD;

(d) Blank, dimethyl fumarate(**c-1**), $FeCl_3$, $FeCl_3$/dimethyl fumarate, $NiCl_2$/DMHD, $FeCl_2$/dimethyl fumarate, and potentials were referenced to non-aqueous Ag/$AgNO_3$ using peak potentials (E_p). The results show c-1 (E_p^{red} = -1.05 V vs Ag/$AgNO_3$), $FeCl_3$/c-1 (E_p^{red} = -1.36 V vs Ag/ $AgNO_3$), Ni(I)/Ni(0) (E_p^{red} = -1.28 V vs Ag/ $AgNO_3$). Despite differences in the measured reduction potentials, the overall conclusions remain unchanged from those using the Ag/AgCl reference electrode. It is noteworthy that $FeCl_2$/c-1 (E_p^{red} = -1.10 V vs Ag/$AgNO_3$) is more easily reduced than Ni(I)/Ni(0) (E_p^{red} = -1.28 V vs Ag/$AgNO_3$), proving that Fe^{3+}, rather than Fe^{2+}, acts as the passivating acceptor. As shown in the following figure S3.

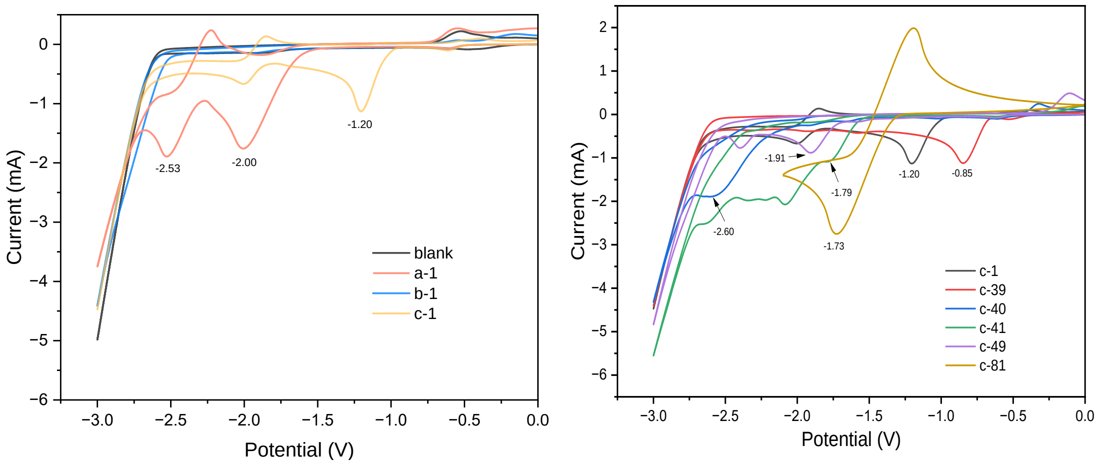

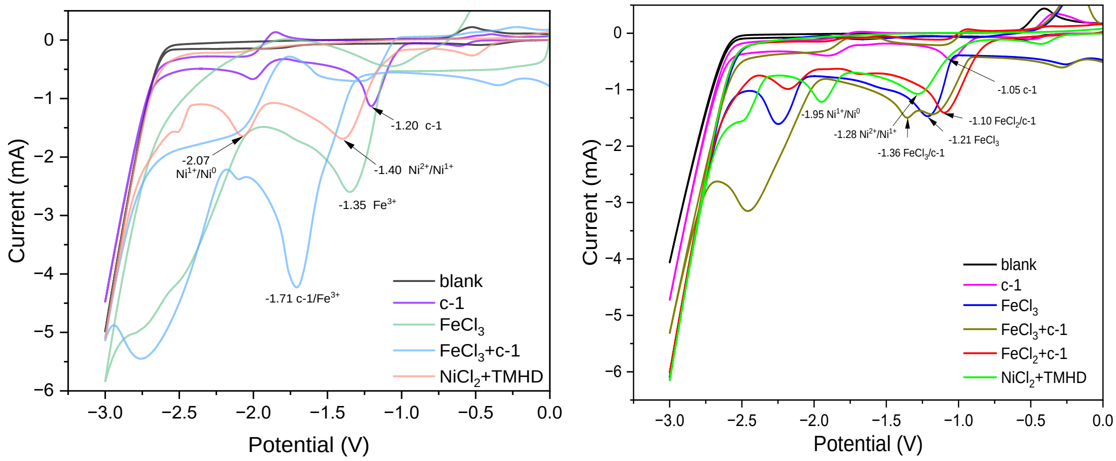

Supplementary Figure 7. Cyclic Voltammetry Studies

(6) $^{13}C\{^1H\}$ NMR spectra studies

(a) $^{13}C\{^1H\}$ NMR spectra of dimethyl fumarate (0.20 M) in the presence of $FeCl_3$ (20.0 mol%) and TMHD (30.0 mol%), $FeCl_3$ (10.0 mol%, 20.0mol%, and 30.0 mol%) were measured in dimethyl sulfoxide-d6 (550 μL) at room temperature. $CHCl_3$ (1.0 equiv., δ 84.4 ppm) was added as a reference standard.

(b) $^{13}C\{^1H\}$ NMR spectra of dimethyl fumarate (0.20 M) in the presence of $FeCl_2$ (20.0 mol%) and TMHD (30.0 mol%), $FeCl_2$ (10.0 mol%, 20.0mol%, and 30.0 mol%) were measured in dimethyl sulfoxide-d6 (550 μL) at room temperature. $CHCl_3$ (1.0 equiv., δ 84.4 ppm) was added as a reference standard.

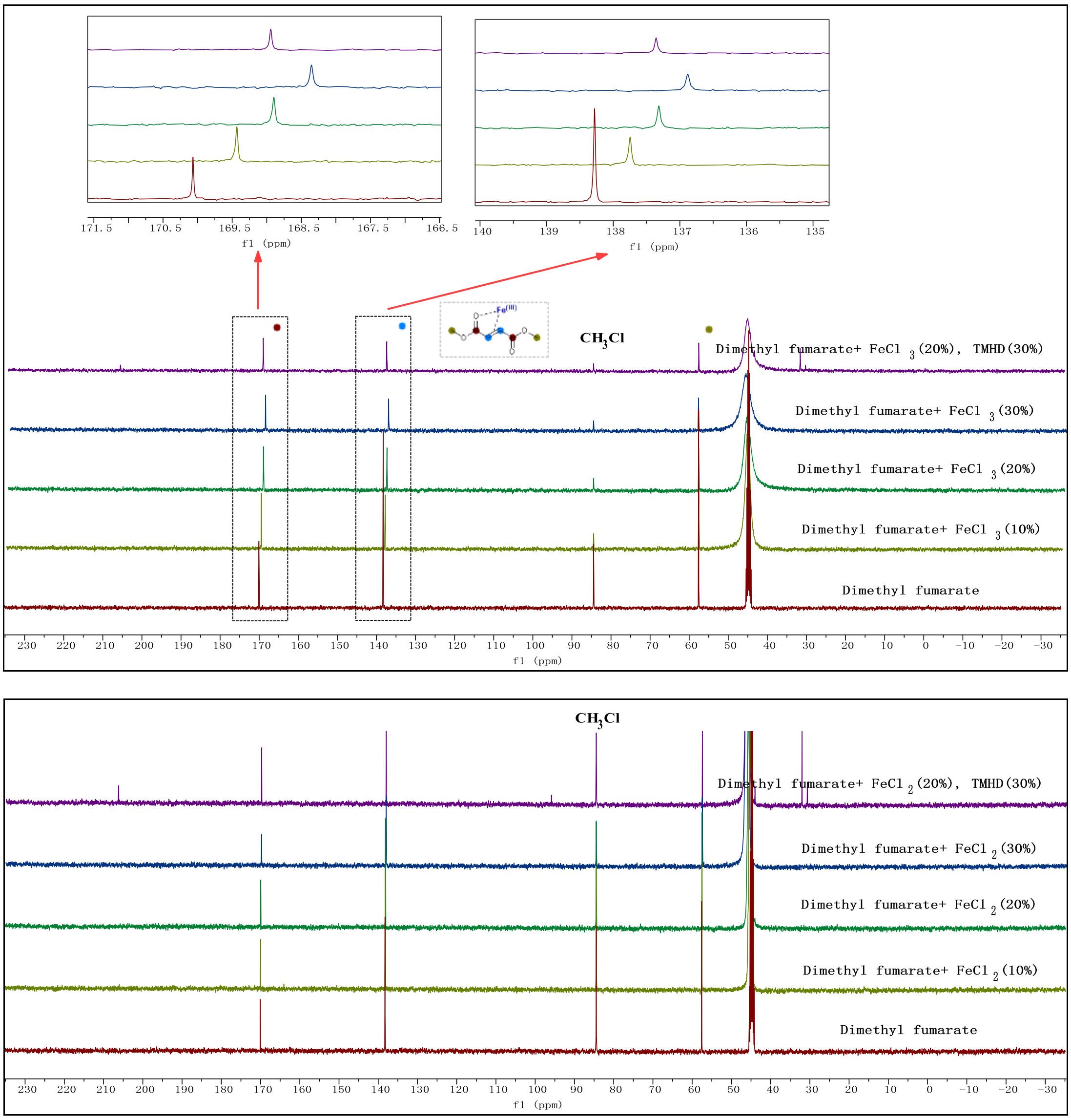

Supplementary Figure 8. $^{13}C\{^{1}H\}$ NMR (101 MHz, $CDCl_3$) of **c-1** with $FeCl_3$ or $FeCl_2$

(7) Stoichiometric reaction without electric current

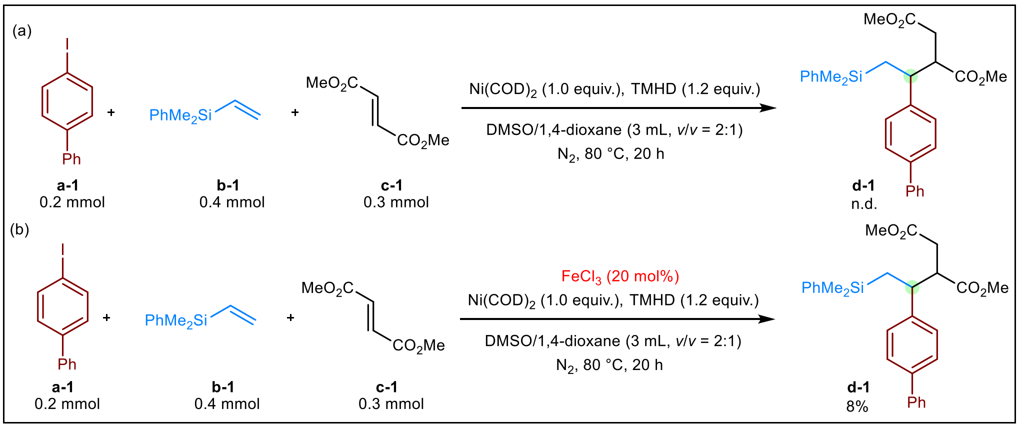

An oven-dried 8 mL reaction vial equipped with a magnetic stir bar was charged under nitrogen with the following components for two parallel trials:

(a) Substrates 4-iodobiphenyl (**a-1**, 0.2 mmol, 56.0 mg), dimethylphenylvinylsilane (**b-1**, 0.4 mmol, 64.8 mg), dimethyl fumarate (**c-1**, 0.3 mmol, 43.2 mg), followed by $Ni(cod)_2$ (55.0 mg, 1.0 equiv.) and TMHD (44.2 mg, 1.2 equiv.);

(b) Identical substrates as in Condition a, with additional $FeCl_3$ (6.5 mg, 20 mol%);

Both conditions utilized 3.0 mL of anhydrous DMSO:1,4-dioxane (2:1 v/v) as solvent. After assembly in a glovebox, the reaction vials were transferred to an external setup. The mixtures were stirred at 80 °C under a nitrogen atmosphere for 20 hours without applied current. Analysis revealed: **(a)** No detected of product of **d-1** were detected; **(b)** **d-1** was isolated in 8% yield. Adding 20 mol% $FeCl_3$ increased the yield to 8%, demonstrating that Fe(III) not only pre-activates metallic nickel (generating catalytically active Ni(II) via oxidative etching in the electrochemical system) but also exhibits inherent Lewis acid activity for **c-1** activation.

(8) Temperature control experiments

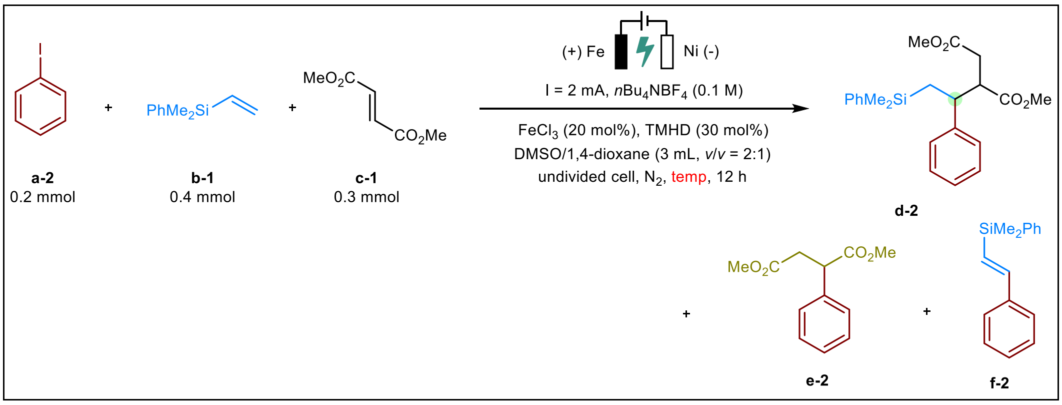

Temperature-dependent reaction optimization was performed in an 8 mL reaction vial equipped with a stir bar. An iron plate anode (10 × 10 × 0.3 mm) and a nickel plate cathode (10 × 10 × 0.15 mm) were immersed in the reaction mixture containing iodobenzene (**a-2**, 0.2 mmol, 40.8 mg), dimethylphenylvinylsilane (**b-1**, 0.4 mmol, 64.8 mg), dimethyl fumarate (**c-1**, 0.3 mmol, 43.2 mg), $FeCl_3$ (6.5 mg, 20 mol%) and TMHD (11.0 mg, 30 mol%) and electrolyte $n$$Bu_4NBF_4$ (98.9 mg, 0.1M). A total of 3.0 mL of

anhydrous solvent (DMSO:1,4-dioxane = 2:1, v/v) was added to the system. The system was subjected to constant-current electrolysis (2.0 mA) for 12 hours across a temperature gradient (30 °C, 40 °C, 50 °C, 60 °C, 70 °C, 80 °C) controlled by a thermostatic oil bath. Reaction progress at each temperature node was monitored by LC-MS analysis using decane as an internal standard, with product yields calculated via external calibration curves.

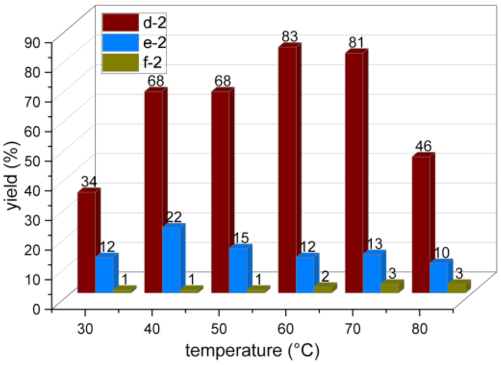

Supplementary Figure 9. Temperature control experiments

(9) EPR experiments

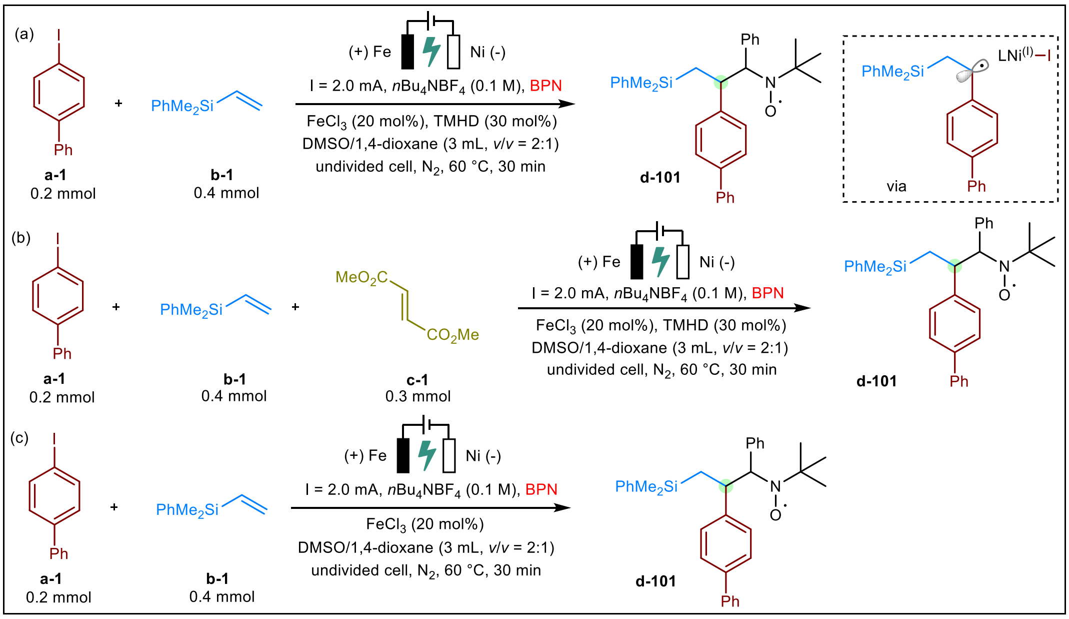

An oven-dried 8 mL reaction vial equipped with a magnetic stir bar and an iron plate anode (10 × 10 × 0.3 mm) and a nickel plate cathode (10 × 10 × 0.15 mm) was charged under nitrogen with the following components for two parallel trials:

(**a**) The reaction mixture containing 4-iodobiphenyl (**a-1**, 0.2 mmol, 56.0 mg), dimethylphenylvinylsilane (**b-1**, 0.4 mmol, 64.8 mg);

(b) The reaction mixture containing 4-iodobiphenyl (**a-1**, 0.2 mmol, 56.0 mg), dimethylphenylvinylsilane (**b-1**, 0.4 mmol, 64.8 mg), dimethyl fumarate (**c-1**, 0.3 mmol, 43.2 mg);

(**c**) The reaction mixture containing 4-iodobiphenyl (**a-1**, 0.2 mmol, 56.0 mg), dimethylphenylvinylsilane (**b-1**, 0.4 mmol, 64.8 mg), and without TMHD. The results provide evidence that the detection of the benzylic radical "m" is dependent on the ligand.

Then continue adding $FeCl_3$ (6.5 mg, 20 mol%) and TMHD (11.0 mg, 30 mol%), electrolyte *n*Bu_4NBF_4 (98.9 mg, 0.1M) and **N-benzylidene-2-methylpropan-2-amine oxide** (**PBN**, 0.2 mmol, 35.4 mg). A total of 3.0 mL of anhydrous solvent (DMSO:1,4-dioxane = 2:1, v/v) was added to the system. The electrochemical reaction proceeded under a constant current of 2.0 mA, maintained at 60 °C for 30 min. Specific test groups included:

EPR spectra was recorded at 298 K on EPR spectrometer operated at 9.8420 GHz. Typical spectrometer parameters are shown as follows, sweep width: ±100 G; center field set: 3510.00 G; conversion time: 60.00 ms; sweep time: 30.72 s; modulation amplitude: 1.0 G; modulation frequency: 100 kHz; PowerAtten:60.0 dB; microwave power: 5.024 mW. The EPR spectroscopy analysis revealed that alkyl radicals were successfully trapped in the reaction, with the detection of the PBN-alkyl radical adduct d-74 (g = 2.0072, a_N = 15.05377 G, a_H = 3.28446 G).

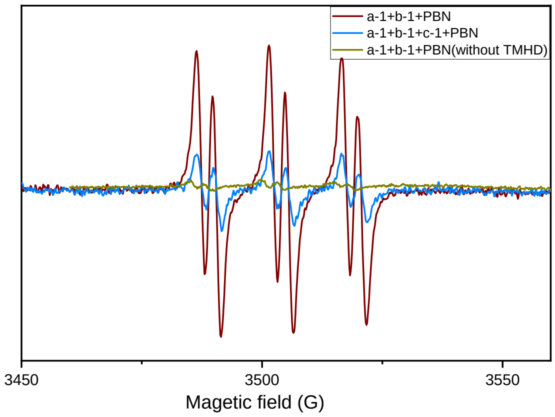

Supplementary Figure 10. EPR experiments

(10) Deuterium-labelled experiments

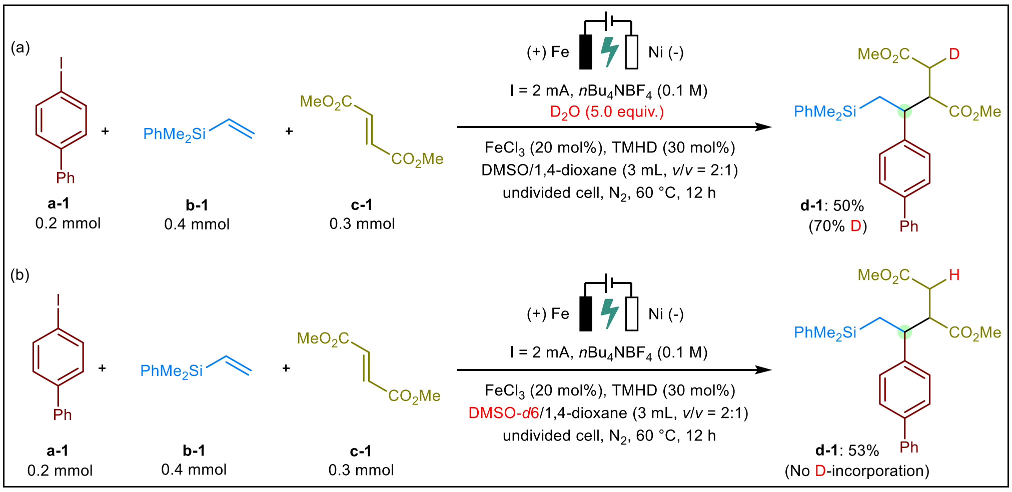

The deuterium-labelled experiments were performed in an 8 mL reaction vial equipped with a stir bar. An iron plate anode (10 × 10 × 0.3 mm) and a nickel plate cathode (10 × 10 × 0.15 mm) were immersed in the reaction mixture containing 4-iodobiphenyl (**a-1**, 0.2 mmol, 56.0 mg), dimethylphenylvinylsilane (**b-1**, 0.4 mmol, 64.8 mg), dimethyl fumarate (**c-1**, 0.3 mmol, 43.2 mg), $FeCl_3$ (6.5 mg, 20 mol%) and TMHD (11.0 g, 30 mol%) and electrolyte $n$$Bu_4NBF_4$ (98.9 mg, 0.1M). A total of 3.0 mL of anhydrous solvent (DMSO:1,4-dioxane = 2:1, v/v) was added to the system Two distinct additive systems were sequentially tested:

(a) D_2O (10.0 mg, 5.0 equiv.);

(b) DMSO-*d*6 instead of DMSO as the solvent.

All reagent handling and cell assembly were performed in a nitrogen-filled glovebox. The assembled reactor was then transferred to an external system, and constant-current electrolysis (2.0 mA) was carried out at 60 °C under a nitrogen atmosphere with vigorous stirring for 12 hours. Product analysis revealed: **(a) d-1** was formed in 50% yield (70% D-incorporation); **(b) d-1** was produced in 53% yield, and no D-incorporation.

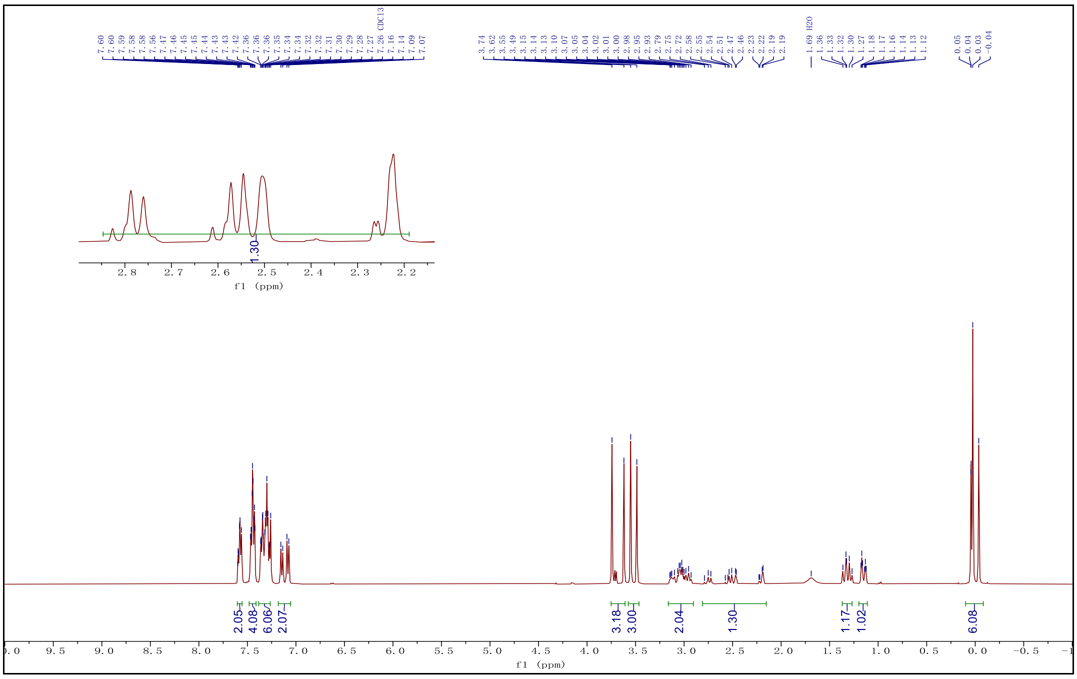

Supplementary Figure 11. ^{1}H NMR (400 MHz, $CDCl_3$) of **d-1** from condition **(a)**

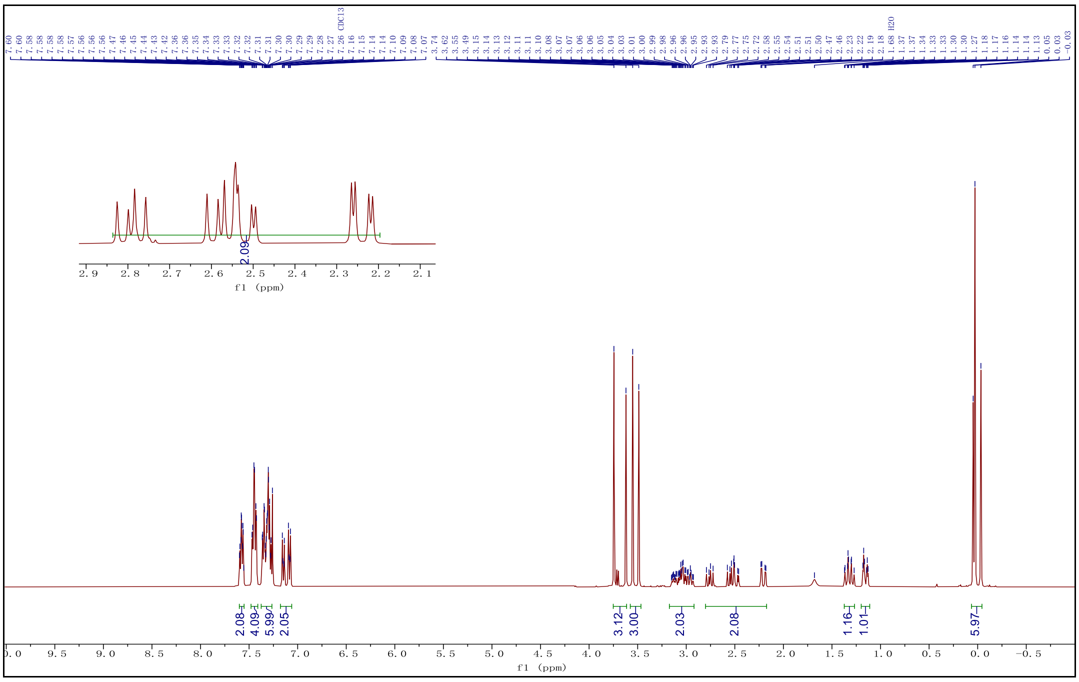

Supplementary Figure 12. ^{1}H NMR (400 MHz, $CDCl_3$) of **d-1** from condition **(b)**

(11) Necessity Control Experiment

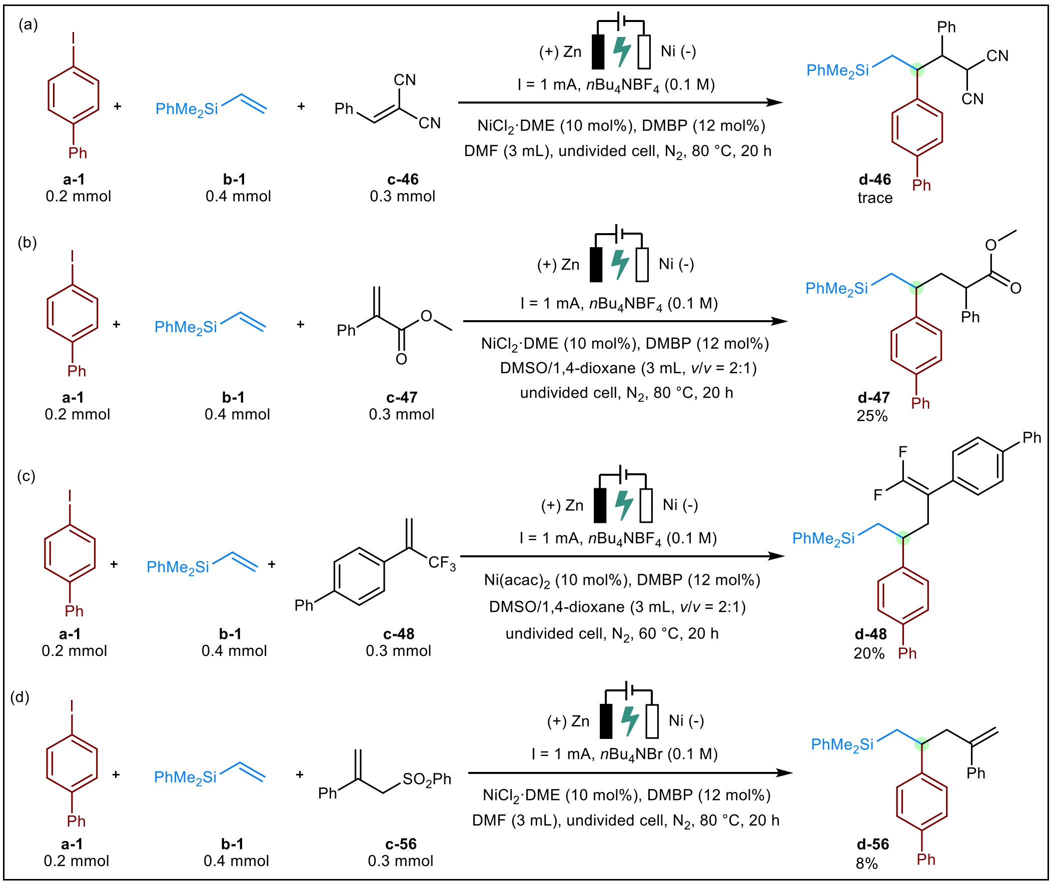

An oven-dried 8 mL reaction vial equipped with a magnetic stir bar, a zinc plate anode, and a nickel plate cathode was charged under nitrogen with the following components for two parallel trials:

(a) Substrates 4-iodobiphenyl (**a-1**, 0.2 mmol, 56.0 mg), dimethylphenylvinylsilane (**b-1**, 0.4 mmol, 64.8 mg), and benzalmalononitrile (**c-46**, 0.3 mmol, 46.2 mg), followed by $NiCl_2$·DME (4.4 mg, 10 mol%), DMBP (4.4 mg, 12 mol%), and 3.0 mL of anhydrous DMF. The mixture was stirred at 80 °C;

(b) Substrates **a-1** (0.2 mmol), **b-1** (0.4 mmol), methyl 2-phenylacrylate (**c-47**, 0.3 mmol, 48.6 mg), followed by $NiCl_2$·DME (4.4 mg, 10 mol%), DMBP (4.4 mg, 12 mol%), and 3.0 mL of anhydrous DMSO:1,4-dioxane (2:1 v/v). The mixture was stirred at 80 °C;

(c) Substrates **a-1** (0.2 mmol), **b-1** (0.4 mmol), (3,3,3-trifluoroprop-1-en-2-yl)benzene (**c-48**, 0.3 mmol, 74.4 mg), followed by $Ni(acac)_2$ (5.2 mg, 10 mol%), DMBP (4.4 mg, 6 mol%), and 3.0 mL anhydrous DMSO:1,4-dioxane (2:1 v/v). The mixture was stirred at 60 °C;

(d) Substrates **a-1** (0.2 mmol), **b-1** (0.4 mmol), [(2-phenyl-2-propenyl)sulfonyl]benzene (**c-56**, 0.3 mmol, 77.4 mg), followed by $NiCl_2$·DME (4.4 mg, 10 mol%), DMBP (4.4 mg, 12 mol%), and 3.0 mL anhydrous DMF. The mixture was stirred at 80 °C;

All assemblies were prepared in a glovebox before transferring to an external setup. Reactions proceeded under nitrogen atmosphere with constant current (1.0 mA) for 20 h. Analysis showed: **(a)** trace detection of **d-46**; **(b)** 25% amounts of **d-47** detected; **(c)** **d-48** obtained in 20% yield; **(d)** 8% amounts of **d-56** detected.

(12) General procedure for the gram-scale reaction

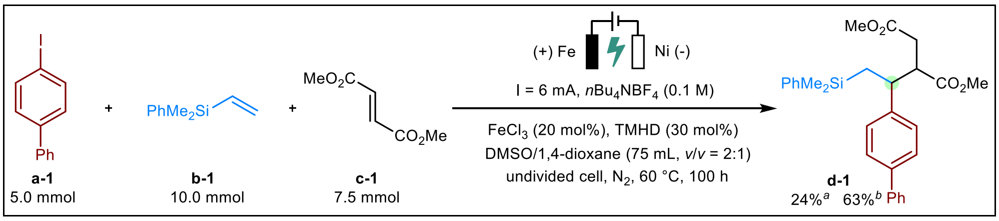

The large-scale reaction was performed in an oven-dried 100 mL solvent flask equipped with a stir bar. An iron plate anode (30 × 30 × 3 mm) and a nickel plate cathode (30 × 30 × 2 mm) were immersed in the reaction mixture containing 4-iodobiphenyl (**a-1**, 5.0 mmol, 1.4 g), dimethylphenylvinylsilane (**b-1**, 10.0 mmol, 1.6 g), dimethyl fumarate (**c-1**, 7.5 mmol, 1.1 g), and electrolyte $n$$Bu_4NBF_4$ (2.5 g, 0.1M). Two distinct additive systems were sequentially tested:

(a) $FeCl_3$ (162.2 mg, 20 mol%) and TMHD (276.2 mg, 30 mol%);

(b) $FeCl_3$ (162.2 mg, 20 mol%), TMHD (276.2 mg, 30 mol%) and $NiCl_2$·DME (54.9 mg, 5.0 mol%).

A total of 75.0 mL of anhydrous solvent (DMSO:1,4-dioxane = 2:1, v/v) was added to the system. All reagent handling and cell assembly were performed in a nitrogen-filled glovebox. The assembled reactor was then transferred to an external system, and constant-current electrolysis (6.0 mA) was carried out at 60 °C under a nitrogen atmosphere with vigorous stirring for 100 hours. Product analysis revealed: **(a)** **d-1** was obtained in 24% yield; **(b)** **d-1** was isolated in 63% yield.

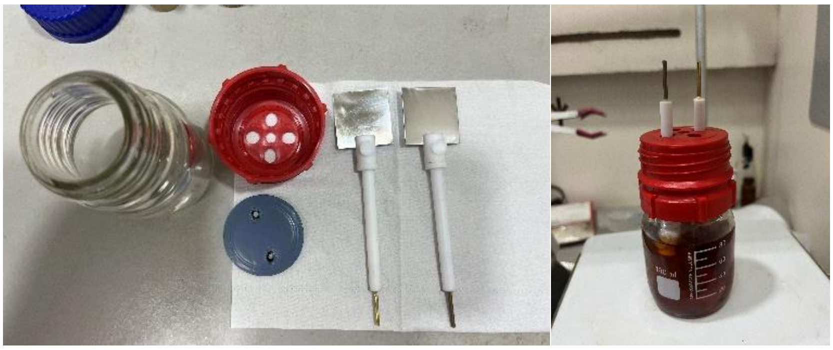

Supplementary Figure 13. Setup for gram-scale electrochemical reaction

(13) Synthesis of dimethyl 2-(1-([1,1'-biphenyl]-4-yl)ethyl)succinate from ethylene

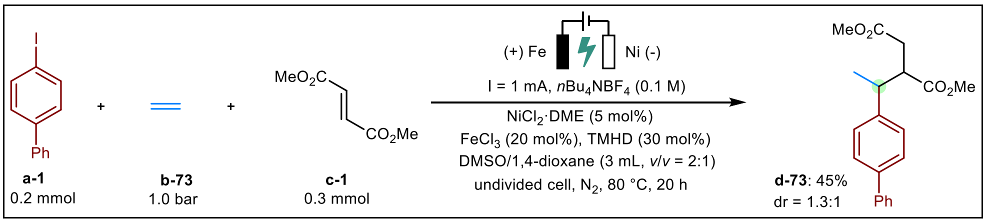

An oven-dried 8 mL reaction vial equipped with a magnetic stir bar, an iron anode, and a nickel cathode (electrode distance: 1.0 cm) was charged under nitrogen with substrate **a-1** (0.2 mmol, 56.0 mg), ethylene gas (1.0 bar), **c-1** (1.5 equiv., 0.3 mmol, 43.2 mg), *n*Bu_4NBF_4 (164.6 mg, 0.1 M), $FeCl_3$ (6.5 mg, 20 mol%), TMHD (11.0 mg, 30 mol%), $NiCl_2$·DME (2.2 mg, 5 mol%), and 5.0 mL of anhydrous DMSO:1,4-dioxane (2:1, v/v). All reagents were weighed and the reaction assembly was completed in a nitrogen-filled glovebox. The vial was transferred to an external setup (**Supplementary Figure 14**), and constant-current electrolysis (1.0 mA) was conducted at 80 °C under an argon atmosphere with vigorous stirring for 20 hours. Upon completion, the mixture was diluted with ethyl acetate (EtOAc) and washed three times with saturated ammonium chloride (NH_4Cl) solution (3 × equal volume). The combined organic layers were dried over anhydrous sodium sulfate (Na_2SO_4), concentrated under reduced pressure, and purified by silica gel column chromatography (gradient elution: PE/EA = 30:1 to 7:1, v/v) to yield product **d-73** as a colorless oil.

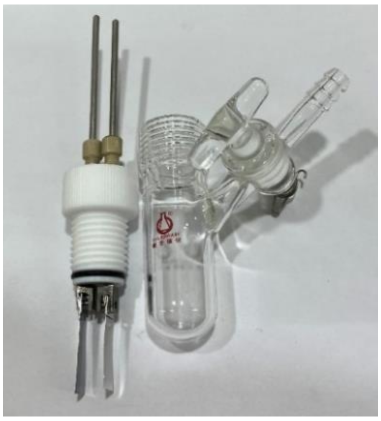

Supplementary Figure 14. Meteorological response device

(14) Controlled potential electrolysis

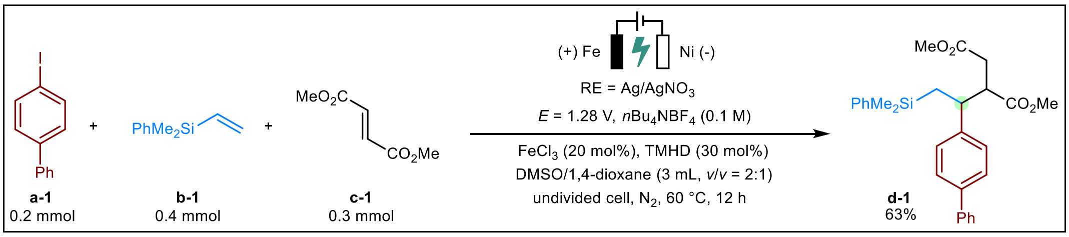

An oven-dried 10 mL three-necked flask with a magneton, a Fe anode, a Ni cathode and nonaqueous Ag/$AgNO_3$ reference electrode was added the substrate **a-1** (0.2 mmol, 1.0 equiv.), **b-1** (0.4 mmol, 2.0 equiv.) and **c-1** (0.3 mmol, 1.5 equiv.) and electrolyte *n*Bu_4NBF_4 (164.7 mg, 0.1M), followed by weighting $FeCl_3$ (6.5 mg, 20 mol%) and TMHD (11.0 mg, 30 mol%), 5.0 mL solvent (Extra Dry, DMSO:1,4-Dioxane=2:1). Weighing of all drugs and assembly of reaction units are done in a nitrogen-filled glove box. The flask was then removed from the glove box. The constant potential (-1.28 V) electrolysis was then performed at 60 °C under N_2 atmosphere with stirring for 12 h. Upon completion, the reaction mixture was diluted with EtOAc and washed with NH_4Cl saturated solution (3 x equal volume) for three times. The combined organic layer was dried over anhydrous Na_2SO_4, and the solvent was then removed under reduced pressure. The resulting mixture was purified by column chromatography on silica gel (eluted with PE / EtOAc = 50:1 to 3:1, v/v) to afford the desired product **d-1**.

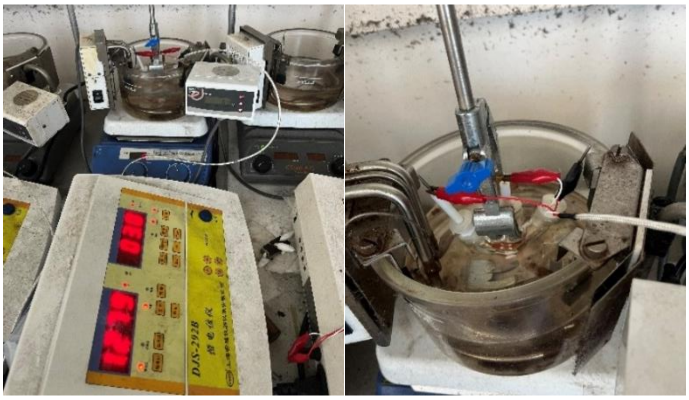

Supplementary Figure 15. Potentiostatic reaction setup

2. Characterization Data of the Products

dimethyl 2-(1-([1,1'-biphenyl]-4-yl)-2-(dimethyl(phenyl)silyl)ethyl)succinate (d-1)

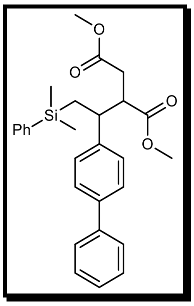

Following the **General procedure A**, **a-1** (56.0 mg, 0.2 mmol), **b-1** (64.9 mg, 0.4 mmol) and **c-1** (43.2 mg, 0.3 mmol) were used. Purification by column chromatography using silica with pentane/EtOAc (50:1 to 10:1, v/v) as eluent afforded **d-1** (73.7 mg, 80% yield) as a white solid.

^{1}H NMR (400 MHz, Chloroform-*d*) δ 7.62 – 7.56 (m, 2H), 7.45 (td, *J* = 8.0, 3.4 Hz, 4H), 7.32 (ddd, *J* = 15.2, 12.6, 6.7 Hz, 6H), 7.12 (dd, *J* = 25.6, 8.0 Hz, 2H), 3.69 (d, *J* = 51.0 Hz, 3H), 3.53 (d, *J* = 24.5 Hz, 3H), 3.18 – 2.91 (m, 2H), 2.82 – 2.17 (m, 2H), 1.34 (dd, *J* = 14.3, 12.1 Hz, 1H), 1.16 (dt, *J* = 14.6, 3.1 Hz, 1H), 0.09 – -0.06 (m, 6H). (*see spectrum*)

major: ^{13}C NMR (101 MHz, Chloroform-*d*) δ 174.88, 172.33, 141.04, 140.61, 139.80, 139.58, 138.58, 138.53, 133.36, 128.71, 128.68, 128.59, 127.59, 127.17, 127.04, 126.87, 77.30, 76.98, 76.66, 51.75, 51.60, 43.82, 34.91, 21.30, -2.49, -3.24. **minor**: **^{13}C NMR** (101 MHz, Chloroform-*d*) δ 174.07, 172.61, 140.98, 140.66, 139.80, 139.58, 138.58, 138.53, 133.36, 128.80, 128.71, 128.68, 127.64, 127.13, 126.87, 126.71, 77.30, 76.98, 76.66, 51.70, 51.52, 50.06, 43.13, 32.88, 18.07, -2.45, -3.22. (*see spectrum*)

HRMS (ESI) (m/z): $[M+H]^+$ calculated for $C_{28}H_{33}O_4Si^+$: 416.2143, found: 416.2140.

dimethyl 2-(2-(dimethyl(phenyl)silyl)-1-phenylethyl)succinate (d-2)

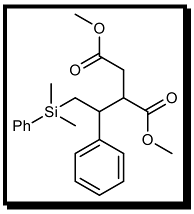

Following the **General procedure A**, **a-2** (40.8 mg, 0.2 mmol), **b-1** (64.9 mg, 0.4 mmol)

and **c-1** (43.2 mg, 0.3 mmol) were used. Purification by column chromatography using silica with pentane/EtOAc (50:1 to 10:1, v/v) as eluent afforded **d-2** (63.8 mg, 83% yield) as a colorless oil.

^{1}H NMR (400 MHz, Chloroform-*d*) δ 7.38 – 7.27 (m, 5H), 7.26 – 7.16 (m, 3H), 7.07 (ddd, *J* = 23.4, 8.1, 1.6 Hz, 2H), 3.66 (d, *J* = 46.5 Hz, 3H), 3.50 (d, *J* = 34.5 Hz, 3H), 3.11 – 2.85 (m, 2H), 2.77 – 2.10 (m, 2H), 1.30 (ddd, *J* = 14.6, 12.2, 3.6 Hz, 1H), 1.12 (dt, *J* = 14.5, 3.0 Hz, 1H), -0.12 (s, 6H). (see spectrum)

major: ^{13}C NMR (101 MHz, Chloroform-*d*) δ 175.04, 172.47, 142.20, 138.82, 133.52, 128.90, 128.63, 128.33, 127.77, 127.22, 51.86, 51.73, 50.77, 44.29, 43.63, 35.03, 21.36, -2.23, -3.39. **minor**: **^{13}C NMR** (101 MHz, Chloroform-*d*) δ 174.25, 172.77, 142.08, 138.75, 133.52, 128.99, 128.44, 128.29, 127.80, 127.05, 51.82, 51.61, 50.27, 44.29, 43.63, 33.03, 18.16, -2.21, -3.29. (see spectrum)

HRMS (ESI) (m/z): $[M+H]^+$ calculated for $C_{27}H_{25}N_2$: 385.1830, found: 385.1833.

dimethyl 2-(1-(4-(tert-butyl)phenyl)-2-(dimethyl(phenyl)silyl)ethyl)succinate (d-3)

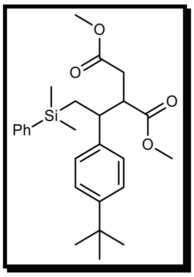

Following the **General procedure A**, **a-3** (52.0 mg, 0.2 mmol), **b-1** (64.9 mg, 0.4 mmol) and **c-1** (43.2 mg, 0.3 mmol) were used. Purification by column chromatography using silica with pentane/EtOAc (50:1 to 10:1, v/v) as eluent afforded **d-3** (63.5 mg, 72% yield) as a white solid.

^{1}H NMR (400 MHz, Chloroform-*d*) δ 7.35 – 7.25 (m, 5H), 7.21 (dd, *J* = 8.3, 1.9 Hz, 2H), 7.02 – 6.91 (m, 2H), 3.66 (d, *J* = 48.4 Hz, 3H), 3.50 (d, *J* = 29.8 Hz, 3H), 3.10 – 2.82 (m, 2H), 2.77 – 2.12 (m, 2H), 1.29 (s, 10H), 1.09 (dd, *J* = 14.6, 3.1 Hz, 1H), -0.12 (s, 6H). (see spectrum)

major: ^{13}C NMR (101 MHz, Chloroform-*d*) δ 175.15, 172.54, 150.02, 138.90, 138.79, 133.48, 128.76, 127.90, 127.66, 125.35, 51.77, 51.65, 50.77, 43.81, 35.14, 34.44, 31.42, 21.53, -2.52, -3.29. **minor**: **^{13}C NMR** (101 MHz, Chloroform-*d*) δ 174.32, 172.85,

149.81, 139.00, 138.90, 133.48, 128.86, 128.03, 127.70, 125.00, 51.73, 51.52, 50.17, 43.04, 34.41, 32.86, 31.42, 18.06, -2.46, -3.21. (*see spectrum*)

HRMS (ESI) (m/z): $[M+H]^+$ calculated for $C_{26}H_{37}O_4Si^+$: 441.2456, found: 441.2460.

dimethyl 2-(1-(4-butylphenyl)-2-(dimethyl(phenyl)silyl)ethyl)succinate (d-4)

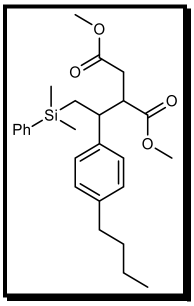

Following the **General procedure A**, **a-4** (52.0 mg, 0.2 mmol), **b-1** (64.9 mg, 0.4 mmol) and **c-1** (43.2 mg, 0.3 mmol) were used. Purification by column chromatography using silica with pentane/EtOAc (50:1 to 10:1, v/v) as eluent afforded **d-4** (54.6 mg, 62% yield) as a colorless oil.

^{1}H NMR (400 MHz, Chloroform-*d*) δ 7.41 – 7.30 (m, 5H), 7.09 – 6.94 (m, 4H), 3.70 (d, *J* = 45.2 Hz, 3H), 3.54 (d, *J* = 33.4 Hz, 3H), 3.13 – 2.86 (m, 2H), 2.80 – 2.51 (m, 3H), 2.51 – 2.15 (m, 1H), 1.66 – 1.57 (m, 2H), 1.43 – 1.27 (m, 3H), 1.14 (dt, *J* = 14.5, 2.9 Hz, 1H), 0.97 (td, *J* = 7.3, 2.3 Hz, 3H), -0.08 (s, 6H). (*see spectrum*)

major: ^{13}C NMR (101 MHz, Chloroform-*d*) δ 175.15, 172.55, 141.76, 139.24, 138.98, 133.51, 128.79, 128.57, 128.26, 128.13, 127.70, 51.79, 51.67, 50.81, 43.92, 35.25, 35.09, 33.66, 22.34, 21.49, 14.02 -2.31, -3.35. **minor**: **^{13}C NMR** (101 MHz, Chloroform-*d*) δ 174.34, 172.84, 141.55, 139.14, 138.90, 133.51, 128.90, 128.57, 128.26, 128.13, 127.74, 51.76, 51.54, 50.28, 43.23, 35.25, 33.66, 32.99, 22.30 18.19, 14.02, -2.30, -3.27. (*see spectrum*)

HRMS (ESI) (m/z): $[M+H]^+$ calculated for $C_{26}H_{37}O_4Si^+$: 441.2456, found: 441.2458.

dimethyl 2-(2-(dimethyl(phenyl)silyl)-1-(4-methoxyphenyl)ethyl)succinate (d-5)

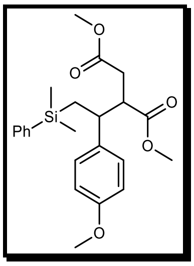

Following the **General procedure A**, **a-5** (46.8 mg, 0.2 mmol), **b-1** (64.9 mg, 0.4 mmol) and **c-1** (43.2 mg, 0.3 mmol) were used. Purification by column chromatography using silica with DCM/ethanol (50:1 to 10:1, v/v) as eluent afforded **d-5** (46.4 mg, 56% yield) as a colorless oil.

^{1}H NMR (400 MHz, Chloroform-*d*) δ 7.37 – 7.26 (m, 5H), 7.02 – 6.90 (m, 2H), 6.78 – 6.72 (m, 2H), 3.66 (d, *J* = 42.8 Hz, 3H), 3.50 (d, *J* = 29.4 Hz, 3H), 3.06 – 2.80 (m, 2H), 2.74 – 2.10 (m, 2H), 1.25 (dd, *J* = 14.6, 12.1 Hz, 1H), 1.10 (ddd, *J* = 14.5, 6.8, 2.9 Hz, 1H), -0.09 (s, 6H). (*see spectrum*)

major: ^{13}C NMR (101 MHz, Chloroform-*d*) δ 175.08, 172.53, 158.66, 138.91, 134.12, 133.50, 129.23, 128.92, 127.75, 127.71, 113.91, 55.26, 51.80, 51.69, 50.95, 43.45, 34.96, 21.49, -2.25, -3.24. **minor**: **^{13}C NMR** (101 MHz, Chloroform-*d*) δ 174.27, 172.78, 158.54, 138.85, 134.06, 133.50, 129.37, 128.92, 128.81, 127.75, 113.59, 55.26, 51.78, 51.59, 50.42, 42.84, 33.13, 18.49, -2.25, -3.20. (*see spectrum*)

HRMS (ESI) (m/z): $[M+H]^+$ calculated for $C_{23}H_{31}O_5Si^+$: 415.1935, found: 415.1935.

dimethyl 2-(2-(dimethyl(phenyl)silyl)-1-(4-(methylthio)phenyl)ethyl)succinate (d-6)

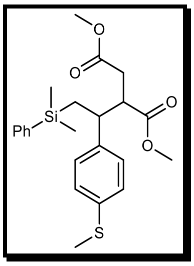

Following the **General procedure A**, **a-6** (50.0 mg, 0.2 mmol), **b-1** (64.9 mg, 0.4 mmol) and **c-1** (43.2 mg, 0.3 mmol) were used. Purification by column chromatography using silica with pentane/EtOAc (50:1 to 5:1, v/v) as eluent afforded **d-6** (52.5 mg, 61% yield) as a colorless oil.

¹H NMR (400 MHz, Chloroform-*d*) δ 7.36 – 7.26 (m, 5H), 7.13 – 7.06 (m, 2H), 7.02 – 6.90 (m, 2H), 3.66 (d, *J* = 43.7 Hz, 3H), 3.50 (d, *J* = 27.6 Hz, 3H), 3.07 – 2.82 (m, 2H), 2.74 – 2.10 (m, 5H), 1.27 – 1.21 (m, 1H), 1.15 – 1.07 (m, 1H), -0.06 (s, 6H). (*see spectrum*)

major: ¹³C NMR (101 MHz, Chloroform-*d*) δ 174.88, 172.39, 139.01, 138.68, 137.08, 133.47, 128.85, 128.79, 127.73, 126.78, 51.84, 51.71, 50.73, 43.74, 34.91, 21.32, 15.94, -2.28, -3.12. **minor**: **¹³C NMR** (101 MHz, Chloroform-*d*) δ 174.09, 172.66, 138.97, 138.63, 136.86, 133.47, 128.96, 128.91, 127.78, 126.78, 51.80, 51.63, 50.20, 43.11, 33.03, 18.23, 15.99, -2.28, -3.10. (*see spectrum*)

HRMS (ESI) (m/z): $[M+H]^+$ calculated for $C_{23}H_{31}O_4SSi^+$: 431.1707, found: 431.1709.

dimethyl 2-(2-(dimethyl(phenyl)silyl)-1-(4-(trifluoromethyl)phenyl)ethyl)succinate (d-7)

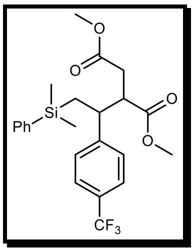

Following the **General procedure A**, **a-7** (54.4 mg, 0.2 mmol), **b-1** (64.9 mg, 0.4 mmol) and **c-1** (43.2 mg, 0.3 mmol) were used. Purification by column chromatography using silica with pentane/EtOAc (50:1 to 10:1, v/v) as eluent afforded **d-7** (43.4 mg, 48% yield) as a colorless oil.

¹H NMR (400 MHz, Chloroform-*d*) δ 7.45 (t, *J* = 7.4 Hz, 2H), 7.37 – 7.24 (m, 5H), 7.15 (dd, *J* = 23.4, 8.0 Hz, 2H), 3.70 (d, *J* = 44.9 Hz, 3H), 3.52 (d, *J* = 37.7 Hz, 3H), 3.18 – 2.97 (m, 2H), 2.78 – 2.09 (m, 2H), 1.31 (ddd, *J* = 14.9, 8.2, 2.8 Hz, 1H), 1.18 (ddd, *J* = 14.4, 11.4, 2.8 Hz, 1H), 0.10 – -0.02 (m, 6H). (*see spectrum*)

major: ¹³C NMR (101 MHz, Chloroform-*d*) δ 174.33, 171.93, 146.05, 137.94, 133.19, 128.83, 128.52, 127.63, 125.27(q, *J* = 3.74 Hz), 124.22(q, *J* = 317.85 Hz), 51.81, 51.63, 50.27, 43.88, 34.58, 21.04, -2.82, -3.12. **minor**: **¹³C NMR** (101 MHz, Chloroform-*d*) δ, 173.67, 172.24, 146.05, 137.91, 133.22, 128.94, 128.62 127.67, 124.96(q, *J* = 3.74

Hz), 124.16(q, J = 317.85 Hz), 51.73, 51.51, 49.87, 43.43, 33.06, 18.33, -2.75, -3.17. (see spectrum)

major: ^{19}F NMR (376 MHz, Chloroform-*d*) δ -62.61. **minor**: **^{19}F NMR** (376 MHz, Chloroform-*d*) δ -62.65. (see spectrum)

HRMS (ESI) (m/z): $[M+H]^+$ calculated for $C_{23}H_{28}F_3O_4Si^+$: 453.1703, found: 453.1697.

dimethyl 2-(2-(dimethyl(phenyl)silyl)-1-(4-(4,4,5,5-tetramethyl-1,3,2-dioxaborolan-2-yl)phenyl)ethyl)succinate (d-8)

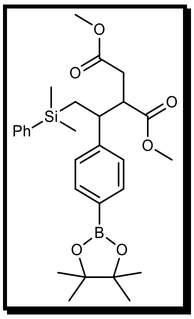

Following the **General procedure A**, **a-8** (66.0 mg, 0.2 mmol), **b-1** (64.9 mg, 0.4 mmol) and **c-1** (43.2 mg, 0.3 mmol) were used. Purification by column chromatography using silica with pentane/EtOAc (50:1 to 10:1, v/v) as eluent afforded **d-8** (61.2 mg, 60% yield) as a colorless oil.

^{1}H NMR (400 MHz, Chloroform-*d*) δ 7.70 – 7.63 (m, 2H), 7.37 – 7.27 (m, 5H), 7.13 – 7.01 (m, 2H), 3.65 (d, J = 45.3 Hz, 3H), 3.49 (d, J = 26.9 Hz, 3H), 3.13 – 2.86 (m, 2H), 2.74 – 2.05 (m, 2H), 1.35 (s, 12H), 1.31 – 1.26 (m, 1H), 1.11 (dt, J = 14.8, 3.1 Hz, 1H), -0.10 (s, 6H). (see spectrum)

major: ^{13}C NMR (101 MHz, Chloroform-*d*) δ 174.87, 172.40, 145.50, 138.75, 135.42, 135.08, 133.50, 128.86, 127.78, 127.74, 83.83, 51.81, 51.66, 50.65 44.37, 34.88, 24.95, 21.10, -2.11, -3.30. **minor**: **^{13}C NMR** (101 MHz, Chloroform-*d*) δ 174.02, 172.67, 145.39, 138.69, 135.42, 135.08, 133.50, 128.96, 127.85, 127.74, 83.80, 51.76, 51.63, 50.08, 43.66, 32.80, 24.95, 17.79, -2.14, -3.23. (see spectrum)

HRMS (ESI) (m/z): $[M+H]^+$ calculated for $C_{28}H_{40}BO_6Si^+$: 511.2682, found: 511.2686.

dimethyl 2-(2-(dimethyl(phenyl)silyl)-1-(4-fluorophenyl)ethyl)succinate (d-9)

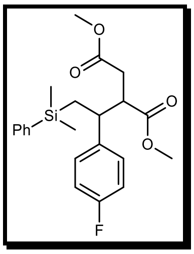

Following the **General procedure A**, **a-9** (44.4 mg, 0.2 mmol), **b-1** (64.9 mg, 0.4 mmol) and **c-1** (43.2 mg, 0.3 mmol) were used. Purification by column chromatography using silica with pentane/EtOAc (50:1 to 10:1, v/v) as eluent afforded **d-9** (45.9 mg, 57% yield) as a colorless oil.

^{1}H NMR (400 MHz, Chloroform-*d*) δ 7.37 – 7.26 (m, 5H), 7.02 (ddd, *J* = 8.7, 5.5, 2.6 Hz, 1H), 6.96 (ddd, *J* = 8.5, 5.4, 2.6 Hz, 1H), 6.89 (td, *J* = 8.6, 3.2 Hz, 2H), 3.66 (d, *J* = 40.2 Hz, 3H), 3.49 (d, *J* = 40.4 Hz, 3H), 3.06 – 2.85 (m, 2H), 2.74 – 2.08 (m, 2H), 1.23 (ddd, *J* = 14.9, 11.8, 4.5 Hz, 1H), 1.12 (td, *J* = 14.4, 13.9, 2.9 Hz, 1H), -0.06 (s, 6H). (*see spectrum*)

major: ^{13}C NMR (101 MHz, Chloroform-*d*) δ 174.80, 172.32, 161.89(d, *J* = 247.45, 1C), 138.48, 137.83(d, *J* = 3.0, 1C), 133.46, 129.76, 129.68, 128.94, 127.78, 115.50, 115.29, 51.91, 51.78, 50.76, 43.49, 34.81, 21.47, -2.33. **minor**: **^{13}C NMR** (101 MHz, Chloroform-*d*) δ174.12, 172.60, 161.82(d, *J* = 247.45, 1C), 138.44, 137.78(d, *J* = 3.0, 1C), 133.46, 129.88, 129.81, 129.04, 127.82, 115.16, 114.95, 51.88, 51.64, 50.35, 43.05, 33.31, 18.81, -3.17. (*see spectrum*)

major: ^{19}F NMR (376 MHz, Chloroform-d) δ -115.43 – -115.56 (m). **minor**: **^{19}F NMR** (376 MHz, Chloroform-d) δ -115.72 – -115.83 (m) (*see spectrum*)

HRMS (ESI) (m/z): $[M+H]^+$ calculated for $C_{22}H_{28}FO_4Si^+$: 403.1735, found: 403.1731.

dimethyl 2-(1-(4-chlorophenyl)-2-(dimethyl(phenyl)silyl)ethyl)succinate (d-10)

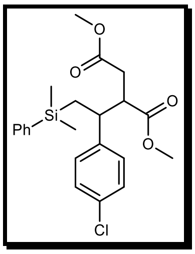

Following the **General procedure A**, **a-10** (47.7 mg, 0.2 mmol), **b-1** (64.9 mg, 0.4 mmol) and **c-1** (43.2 mg, 0.3 mmol) were used. Purification by column chromatography

using silica with pentane/EtOAc (50:1 to 10:1, v/v) as eluent afforded **d-10** (49.4 mg, 59% yield) as a colorless oil.

^{1}H NMR (400 MHz, Chloroform-*d*) δ 7.37 – 7.26 (m, 5H), 7.20 – 7.13 (m, 2H), 7.02 – 6.90 (m, 2H), 3.66 (d, *J* = 40.1 Hz, 3H), 3.50 (d, *J* = 34.9 Hz, 3H), 3.07 – 2.84 (m, 2H), 2.73 – 2.08 (m, 2H), 1.24 (ddd, *J* = 15.1, 11.7, 3.7 Hz, 1H), 1.13 (ddd, *J* = 14.3, 10.4, 2.9 Hz, 1H), 0.02 – -0.04 (m, 6H). (*see spectrum*)

major: ^{13}C NMR (101 MHz, Chloroform-*d*) δ 174.62, 172.21, 140.64, 138.34, 133.42, 132.85, 129.62, 128.91, 128.6, 128.36, 127.77, 51.88, 51.74, 50.59, 43.61, 34.76, 21.30, -3.09. **minor**: **^{13}C NMR** (101 MHz, Chloroform-*d*) δ 173.93, 172.48, 140.61, 138.31, 133.42, 132.71, 129.74, 129.02, 128.36, 127.81, 51.84, 51.63, 50.16, 43.14, 33.23, 18.58, -2.37. (*see spectrum*)

HRMS (ESI) (m/z): $[M+H]^+$ calculated for $C_{22}H_{28}ClO_4Si^+$: 419.1440, found: 419。1437.

dimethyl 2-(1-(4-bromophenyl)-2-(dimethyl(phenyl)silyl)ethyl)succinate (d-11)

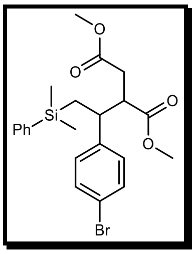

Following the **General procedure A**, **a-11** (56.6 mg, 0.2 mmol), **b-1** (64.9 mg, 0.4 mmol) and **c-1** (43.2 mg, 0.3 mmol) were used. Purification by column chromatography using silica with pentane/EtOAc (50:1 to 10:1, v/v) as eluent afforded **d-11** (51.0 mg, 55% yield) as a white solid.

^{1}H NMR (400 MHz, Chloroform-*d*) δ 7.26 – 7.14 (m, 7H), 6.87 – 6.70 (m, 2H), 3.56 (d, *J* = 41.5 Hz, 3H), 3.40 (d, *J* = 34.1 Hz, 3H), 2.94 – 2.73 (m, 2H), 2.63 – 1.98 (m, 2H), 1.13 (ddd, *J* = 15.8, 11.8, 3.8 Hz, 1H), 1.01 (ddd, *J* = 14.5, 9.6, 2.9 Hz, 1H), -0.09 (s, 6H). (*see spectrum*)

major: ^{13}C NMR (101 MHz, Chloroform-*d*) δ 174.67, 172.24, 141.15, 138.31, 133.44, 131.63, 130.01, 128.93, 127.80, 120.95, 51.94, 51.80, 50.53, 43.70, 34.78, 21.26, -2.38, -3.05. **minor**: **^{13}C NMR** (101 MHz, Chloroform-*d*) δ 173.96, 172.52, 141.11, 138.28,

133.44, 131.33, 130.12, 129.04, 127.84, 120.82, 51.89, 51.70, 50.09, 43.18, 33.17, 18.47, -2.35, -3.05. (see spectrum)

HRMS (ESI) (m/z): $[M+H]^+$ calculated for $C_{22}H_{28}BrO_4Si^+$: 463.0935, found: 463.0928.

dimethyl 2-(2-(dimethyl(phenyl)silyl)-1-(4-iodophenyl)ethyl)succinate (d-12)

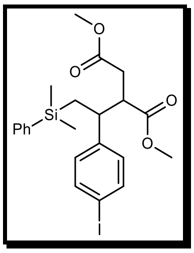

Following the **General procedure A**, **a-12** (66.0 mg, 0.2 mmol), **b-1** (64.9 mg, 0.4 mmol) and **c-1** (43.2 mg, 0.3 mmol) were used. Purification by column chromatography using silica with pentane/EtOAc (50:1 to 10:1, v/v) as eluent afforded **d-12** (54.1 mg, 53% yield) as a colorless oil.

^{1}H NMR (400 MHz, Chloroform-*d*) δ 7.54 – 7.46 (m, 2H), 7.36 – 7.24 (m, 6H), 6.87 – 6.67 (m, 2H), 3.66 (d, *J* = 41.4 Hz, 3H), 3.50 (d, *J* = 31.9 Hz, 3H), 3.00 – 2.82 (m, 2H), 2.72 – 2.43 (m, 1H), 2.43 – 2.08 (m, 1H), 1.23 (ddd, *J* = 15.3, 11.7, 3.3 Hz, 1H), 1.11 (ddd, *J* = 14.6, 8.0, 2.9 Hz, 1H), 0.03 – -0.03 (m, 6H). (see spectrum)

major: ^{13}C NMR (101 MHz, Chloroform-*d*) δ 174.64, 172.21, 141.81, 138.29, 137.57, 133.42, 130.29, 128.89, 127.80, 92.44, 92.31, 51.92, 51.78, 50.50, 43.80, 34.79, 21.23, -2.44, -3.01. **minor**: **^{13}C NMR** (101 MHz, Chloroform-*d*) δ173.92, 172.50, 141.78, 138.27, 137.28, 133.42, 130.41, 129.01, 127.83, 92.31, 51.87, 51.69, 50.05, 43.26, 33.14, 18.37, -2.39, -3.02. (see spectrum)

HRMS (ESI) (m/z): $[M+H]^+$ calculated for $C_{22}H_{28}IO_4Si^+$: 511.0796, found: 511.0786.

dimethyl 2-(1-(4-acetylphenyl)-2-(dimethyl(phenyl)silyl)ethyl)succinate (d-13)

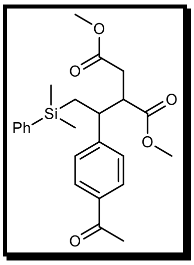

Following the **General procedure A**, **a-13** (49.2.0 mg, 0.2 mmol), **b-1** (64.9 mg, 0.4 mmol) and **c-1** (43.2 mg, 0.3 mmol) were used. Purification by column chromatography

using silica with pentane/EtOAc (20:1 to 5:1, v/v) as eluent afforded **d-13** (50.3 mg, 59% yield) as a white solid.

^{1}H NMR (400 MHz, Chloroform-*d*) δ 7.85 – 7.75 (m, 2H), 7.37 – 7.23 (m, 5H), 7.19 – 7.07 (m, 2H), 3.67 (d, *J* = 44.0 Hz, 3H), 3.50 (d, *J* = 37.3 Hz, 3H), 3.14 – 2.96 (m, 2H), 2.77 – 2.06 (m, 5H), 1.34 – 1.26 (m, 1H), 1.16 (ddd, *J* = 14.4, 10.0, 2.7 Hz, 1H), 0.02 – -0.04 (m, 6H). (see spectrum)

major: ^{13}C NMR (101 MHz, Chloroform-*d*) δ 197.77, 173.82, 172.42, 147.82, 138.22, 135.98, 133.44, 129.05, 128.69, 128.34, 127.82, 51.88, 51.69, 49.99, 43.70, 33.25, 26.63, 18.42, -2.39. **minor**: **^{13}C NMR** (101 MHz, Chloroform-*d*) δ 197.65, 174.50, 172.13, 147.82, 138.26, 136.13, 133.44, 128.93, 128.63, 128.57, 127.77, 51.95, 51.78, 50.39, 44.14, 33.25, 26.63, 21.09, -3.06. (see spectrum)

HRMS (ESI) (m/z): $[M+H]^+$ calculated for $C_{24}H_{31}O_5Si^+$: 427.1935, found: 427.1933.

dimethyl 2-(2-(dimethyl(phenyl)silyl)-1-(4-(methoxycarbonyl)phenyl)ethyl)succinate (d-14)

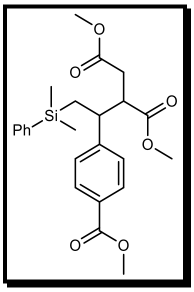

Following the **General procedure A**, **a-14** (52.4 mg, 0.2 mmol), **b-1** (64.9 mg, 0.4 mmol) and **c-1** (43.2 mg, 0.3 mmol) were used. Purification by column chromatography using silica with pentane/EtOAc (20:1 to 5:1, v/v) as eluent afforded **d-14** (39.8 mg, 45% yield) as a colorless oil.

^{1}H NMR (400 MHz, Chloroform-*d*) δ 7.77 (dd, *J* = 8.2, 3.7 Hz, 2H), 7.27 – 7.15 (m, 5H), 7.00 (dd, *J* = 23.4, 8.2 Hz, 2H), 3.79 (s, 3H), 3.54 (d, *J* = 42.1 Hz, 3H), 3.36 (d, *J* = 43.0 Hz, 3H), 3.01 – 2.81 (m, 2H), 2.64 – 1.93 (m, 2H), 1.20 – 1.14 (m, 1H), 1.04 (ddd, *J* = 14.3, 9.2, 2.8 Hz, 1H), -0.13 – -0.19 (m, 6H). (see spectrum)

major: ^{13}C NMR (101 MHz, Chloroform-*d*) δ 174.54, 172.15, 166.86, 147.67, 138.27, 133.46, 129.90, 129.11, 129.00, 128.39, 127.80, 52.15, 51.93, 51.77, 50.43, 44.18, 34.76, 21.10, -2.23, -3.20. **minor**: **^{13}C NMR** (101 MHz, Chloroform-*d*) δ 173.88,

172.45, 166.96, 147.64, 138.23, 133.46, 129.59, 129.00, 128.94, 128.50, 127.84, 52.13, 51.88, 51.67, 50.05, 43.76, 33.25, 18.41, -2.26, -3.17. (*see spectrum*)

HRMS (ESI) (m/z): $[M+H]^+$ calculated for $C_{24}H_{31}O_6Si^+$: 415.1884, found: 415.1894.

dimethyl 2-(1-(4-(tert-butoxycarbonyl)phenyl)-2-(dimethyl(phenyl)silyl)ethyl)succinate (d-15)

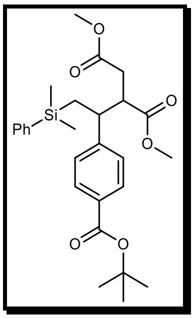

Following the **General procedure A**, **a-15** (60.8 mg, 0.2 mmol), **b-1** (64.9 mg, 0.4 mmol) and **c-1** (43.2 mg, 0.3 mmol) were used. Purification by column chromatography using silica with pentane/EtOAc (20:1 to 5:1, v/v) as eluent afforded **d-15** (51.4 mg, 53% yield) as a colorless oil.

^{1}H NMR (400 MHz, Chloroform-*d*) δ 7.83 (dd, *J* = 8.0, 4.7 Hz, 2H), 7.37 – 7.26 (m, 5H), 7.09 (dd, *J* = 24.1, 8.0 Hz, 2H), 3.66 (d, *J* = 44.4 Hz, 3H), 3.49 (d, *J* = 32.4 Hz, 3H), 3.13 – 2.92 (m, 2H), 2.74 – 2.04 (m, 2H), 1.60 (s, 9H), 1.29 (dd, *J* = 14.5, 11.8 Hz, 1H), 1.14 (ddd, *J* = 14.4, 6.1, 2.6 Hz, 1H), -0.06 (s, 6H). (*see spectrum*)

major: ^{13}C NMR (101 MHz, Chloroform-*d*) δ 174.60, 172.20, 165.54, 147.03, 138.37, 133.45, 130.97, 129.73, 128.95, 128.17, 127.78, 81.03, 51.91, 51.74, 50.50, 44.12, 34.73, 28.26, 21.11, -2.22, -3.14. **minor**: **^{13}C NMR** (101 MHz, Chloroform-*d*) δ 173.86, 172.48, 165.64, 146.97, 138.34, 133.45, 130.80, 129.43, 129.05, 128.17, 127.83, 80.99, 51.84, 51.69, 50.04, 43.62, 33.12, 28.26, 18.29, -2.22, -3.14. (*see spectrum*)

HRMS (ESI) (m/z): $[M+H]^+$ calculated for $C_{27}H_{37}O_6Si^+$: 485.2354, found: 485.2346.

dimethyl 2-(2-(dimethyl(phenyl)silyl)-1-(4-pivalamidophenyl)ethyl)succinate (d-16)

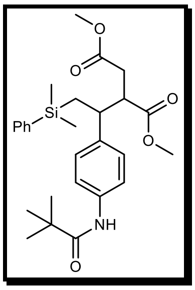

Following the **General procedure A**, **a-16** (60.6 mg, 0.2 mmol), **b-1** (64.9 mg, 0.4 mmol) and **c-1** (43.2 mg, 0.3 mmol) were used. Purification by column chromatography using silica with pentane/EtOAc (10:1 to 5:1, v/v) as eluent afforded **d-16** (43.5 mg, 45% yield) as a colorless oil.

^{1}H NMR (400 MHz, Chloroform-*d*) δ 7.36 – 7.27 (m, 5H), 7.23 (dd, *J* = 8.4, 3.8 Hz, 2H), 7.03 – 6.90 (m, 2H), 6.47 (s, 1H), 3.65 (d, *J* = 41.6 Hz, 3H), 3.50 (d, *J* = 27.7 Hz, 3H), 3.06 – 2.80 (m, 2H), 2.73 – 2.09 (m, 2H), 1.52 (s, 9H), 1.28 – 1.20 (m, 1H), 1.08 (dd, *J* = 14.6, 3.3 Hz, 1H), -0.09 (s, 6H). (*see spectrum*)

major: ^{13}C NMR (101 MHz, Chloroform-*d*) δ 174.99, 172.49, 152.75, 138.80, 137.38, 136.69, 133.51, 128.84, 128.81, 127.73, 118.49, 80.54, 51.82, 51.70, 50.83, 43.59, 34.91, 28.41, 21.35, -2.14, -3.27. **minor**: **^{13}C NMR** (101 MHz, Chloroform-*d*) δ 174.17, 172.75, 152.75, 138.75, 137.25, 136.59, 133.51, 128.95, 128.93, 127.78, 118.16, 80.58, 51.79, 51.63, 50.28, 42.92, 32.94, 28.41, 18.18, -2.15, -3.22. (*see spectrum*)

HRMS (ESI) (m/z): $[M+H]^+$ calculated for $C_{27}H_{38}NO_5Si^+$: 484.2514, found: 484.2524.

dimethyl 2-(2-(dimethyl(phenyl)silyl)-1-(4-(trimethylsilyl)phenyl)ethyl)succinate (d-17)

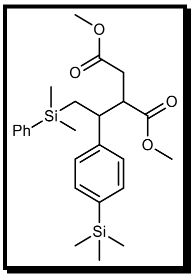

Following the **General procedure A**, **a-17** (45.8.0 mg, 0.2 mmol), **b-1** (64.9 mg, 0.4 mmol) and **c-1** (43.2 mg, 0.3 mmol) were used. Purification by column chromatography using silica with pentane/EtOAc (20:1 to 7:1, v/v) as eluent afforded **d-17** (41.1 mg, 45% yield) as a colorless oil.

^{1}H NMR (400 MHz, Chloroform-*d*) δ 7.36 – 7.25 (m, 7H), 7.02 (dd, *J* = 23.5, 7.9 Hz, 2H), 3.66 (d, *J* = 50.4 Hz, 3H), 3.50 (d, *J* = 25.2 Hz, 3H), 3.11 – 2.84 (m, 2H), 2.61 (ddd, *J* = 91.4, 16.7, 10.9 Hz, 1H), 2.28 (ddd, *J* = 112.4, 16.7, 3.8 Hz, 1H), 1.31 – 1.25 (m, 1H), 1.09 (ddd, *J* = 14.7, 3.2, 1.8 Hz, 1H), 0.24 (s, 9H), -0.10 (s, 6H). (*see spectrum*)

major: ^{13}C NMR (101 MHz, Chloroform-*d*) δ 175.06, 172.49, 142.55, 139.10, 138.86, 133.48, 133.46, 128.78, 127.73, 127.68, 51.83, 51.69, 50.67, 44.25, 35.07, 21.36, -1.04, -2.54, -3.21. **minor**: **^{13}C NMR** (101 MHz, Chloroform-*d*) δ 174.21, 172.80, 142.45, 138.86, 138.78, 133.52, 133.21, 128.90, 127.81, 127.68, 51.77, 51.60, 50.06, 43.44 32.77, 17.81, -1.04, -2.54, -3.21. (*see spectrum*)

HRMS (ESI) (m/z): $[M+H]^+$ calculated for $C_{25}H_{37}O_4Si_2^+$: 457.2225, found: 457.2233.

dimethyl 2-(1-(4-(4-butylcyclohexyl)phenyl)-2-(dimethyl(phenyl)silyl)ethyl)succinate (d-18)

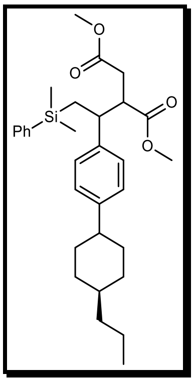

Following the **General procedure A**, **a-18** (68.5 mg, 0.2 mmol), **b-1** (64.9 mg, 0.4 mmol) and **c-1** (43.2 mg, 0.3 mmol) were used. Purification by column chromatography using silica with pentane/EtOAc (50:1 to 10:1, v/v) as eluent afforded **d-18** (68.0 mg, 65% yield) as a colorless oil.

^{1}H NMR (400 MHz, Chloroform-*d*) δ 7.36 – 7.24 (m, 5H), 7.04 (dd, *J* = 8.2, 2.3 Hz, 2H), 6.95 (dd, *J* = 22.0, 8.2 Hz, 2H), 3.66 (d, *J* = 47.4 Hz, 3H), 3.49 (d, *J* = 31.6 Hz, 3H), 3.09 – 2.82 (m, 2H), 2.76 – 2.46 (m, 1H), 2.46 – 2.12 (m, 2H), 1.86 (d, *J* = 12.6 Hz, 4H), 1.47 – 1.22 (m, 8H), 1.12 – 1.01 (m, 3H), 0.91 (t, *J* = 7.2 Hz, 3H), -0.13 (s, 6H). (*see spectrum*)

major: ^{13}C NMR (101 MHz, Chloroform-*d*) δ 175.16, 172.54, 146.80, 139.34, 138.99, 133.50, 128.78, 128.13, 127.68, 126.94, 51.78, 51.66, 50.79, 44.27, 43.94, 39.81, 37.11,

35.15, 34.43, 33.66, 21.54, 20.10, 14.48, -2.41, -3.35. **minor**: **^{13}C NMR** (101 MHz, Chloroform-*d*) δ 174.33, 172.85, 146.60, 139.23, 138.90, 133.50, 128.88, 128.26, 127.72, 126.60, 51.74, 51.53, 50.23, 44.27, 43.18, 39.81, 37.11, 34.46, 33.66, 32.89, 20.10, 18.10, 14.48, -2.37, -3.25. (<u>*see spectrum*</u>)

HRMS (ESI) (m/z): $[M+H]^+$ calculated for $C_{31}H_{45}O_4Si^+$: 509.3082, found: 509.3075.

dimethyl 2-(2-(dimethyl(phenyl)silyl)-1-(3,5-dimethylphenyl)ethyl)succinate (d-19)

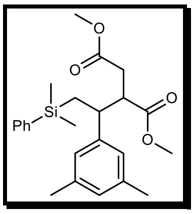

Following the **General procedure A**, **a-19** (46.4 mg, 0.2 mmol), **b-1** (64.9 mg, 0.4 mmol) and **c-1** (43.2 mg, 0.3 mmol) were used. Purification by column chromatography using silica with pentane/EtOAc (50:1 to 10:1, v/v) as eluent afforded **d-19** (54.4 mg, 66% yield) as a colorless oil.

^{1}H NMR (400 MHz, Chloroform-*d*) δ 7.36 – 7.26 (m, 5H), 6.79 (d, *J* = 5.9 Hz, 1H), 6.67 (s, 1H), 6.58 (s, 1H), 3.66 (d, *J* = 47.8 Hz, 3H), 3.53 (d, *J* = 18.7 Hz, 3H), 3.07 – 2.97 (m, 1H), 2.97 – 2.75 (m, 1H), 2.74 – 2.45 (m, 1H), 2.44 – 2.11 (m, 7H), 1.30 – 1.20 (m, 1H), 1.06 (ddd, *J* = 14.9, 6.9, 3.1 Hz, 1H), -0.09 (s, 6H). (<u>*see spectrum*</u>)

major: ^{13}C NMR (101 MHz, Chloroform-*d*) δ 175.23, 172.61, 141.93, 138.99, 137.86, 133.51, 128.77, 128.66, 127.59, 126.29, 51.77, 51.66, 50.67, 44.17, 35.18, 21.32, 17.54, -2.33, -3.20. **minor**: **^{13}C NMR** (101 MHz, Chloroform-*d*) δ 174.28, 172.90, 141.81, 138.97, 137.49, 133.51, 128.89, 128.49, 127.67, 126.14, 51.75, 51.60, 50.10, 43.20, 32.56, 21.32, 17.54, -2.33, -3.06. (<u>*see spectrum*</u>)

HRMS (ESI) (m/z): $[M+H]^+$ calculated for $C_{24}H_{33}O_4Si^+$: 413.2143, found: 413.2143.

dimethyl 2-(2-(dimethyl(phenyl)silyl)-1-(naphthalen-2-yl)ethyl)succinate (d-20)

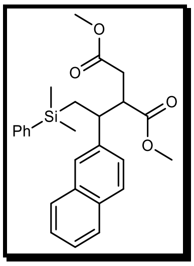

Following the **General procedure A**, **a-20** (50.8 mg, 0.2 mmol), **b-1** (64.9 mg, 0.4 mmol) and **c-1** (43.2 mg, 0.3 mmol) were used. Purification by column chromatography using silica with pentane/EtOAc (50:1 to 10:1, v/v) as eluent afforded **d-20** (37.4 mg, 43% yield) as a colorless oil.

^{1}H NMR (400 MHz, Chloroform-*d*) δ 7.78 (dt, *J* = 7.1, 2.7 Hz, 1H), 7.72 (dt, *J* = 9.9, 5.0 Hz, 2H), 7.48 – 7.39 (m, 3H), 7.34 – 7.18 (m, 6H), 3.66 (d, *J* = 65.1 Hz, 3H), 3.46 (d, *J* = 23.1 Hz, 3H), 3.31 – 3.02 (m, 2H), 2.81 – 2.09 (m, 2H), 1.41 (ddd, *J* = 14.7, 12.2, 9.3 Hz, 1H), 1.20 (dt, *J* = 14.8, 2.8 Hz, 1H), -0.08 (d, *J* = 18.0 Hz, 6H). (*see spectrum*)

major: ^{13}C NMR (101 MHz, Chloroform-*d*) δ 174.18, 172.70, 139.55, 138.66, 133.49, 133.22, 132.72, 128.96, 128.48, 127.80, 127.73, 127.65, 127.15, 126.60, 126.02, 125.75, 51.78, 51.63, 50.11, 43.66, 32.87, 17.92, -2.25, -3.08. **minor**: **^{13}C NMR** (101 MHz, Chloroform-*d*) δ 175.02, 172.38, 139.57, 138.64, 133.49, 133.32, 132.72, 128.83, 128.48, 128.00, 127.73, 127.61, 127.15, 126.60, 126.17, 125.69, 51.88, 51.64, 50.65, 44.46, 35.18, 21.30, -2.22 -3.19. (*see spectrum*)

HRMS (ESI) (m/z): $[M+H]^+$ calculated for $C_{26}H_{31}O_4Si^+$: 435.1986, found: 435.1994.

dimethyl 2-(2-(dimethyl(phenyl)silyl)-1-(9H-fluoren-3-yl)ethyl)succinate (d-21)

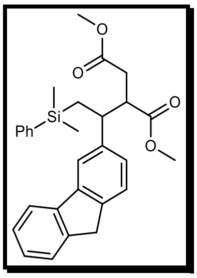

Following the **General procedure A**, **a-21** (58.4 mg, 0.2 mmol), **b-1** (64.9 mg, 0.4 mmol) and **c-1** (43.2 mg, 0.3 mmol) were used. Purification by column chromatography using silica with pentane/EtOAc (50:1 to 10:1, v/v) as eluent afforded **d-21** (42.5 mg, 45% yield) as a colorless oil.

^{1}H NMR (400 MHz, Chloroform-*d*) δ 7.70 – 7.64 (m, 1H), 7.56 (dd, *J* = 7.8, 4.0 Hz, 1H), 7.45 (d, *J* = 7.4 Hz, 1H), 7.31 – 7.06 (m, 8H), 7.00 (dd, *J* = 24.5, 7.9 Hz, 1H), 3.80 – 3.50 (m, 5H), 3.40 (d, *J* = 28.7 Hz, 3H), 3.12 – 2.82 (m, 2H), 2.74 – 2.06 (m, 2H),

1.33 – 1.22 (m, 1H), 1.09 (ddd, J = 14.5, 5.0, 3.1 Hz, 1H), -0.05 – -0.17 (m, 6H). (see spectrum)

major: ^{13}**C NMR** (101 MHz, Chloroform-d) δ 175.14, 172.77, 143.64, 143.33, 141.53, 140.87, 140.83, 138.82, 133.50, 128.77, 127.65, 127.19, 126.84, 126.69, 125.11, 125.08, 119.87, 119.85, 51.87, 51.70, 50.97, 44.48, 36.86, 35.20, 21.62, -2.24, -3.04.

minor: ^{13}**C NMR** (101 MHz, Chloroform-d) δ 174.32, 172.51, 143.40, 143.29, 141.64, 140.84, 140.64, 138.80, 133.50, 128.91, 127.73, 127.15, 126.82, 126.62, 125.11, 124.90, 119.85, 119.59, 51.82, 51.64, 50.48, 43.83, 36.86, 33.19, 18.48, -2.24, -3.00. (see spectrum)

HRMS (ESI) (m/z): $[M+H]^+$ calculated for $C_{29}H_{33}O_4Si^+$: 473.2143, found: 473.2141.

dimethyl 2-(2-(dimethyl(phenyl)silyl)-1-(9-phenyl-9H-carbazol-2-yl)ethyl)succinate (d-22)

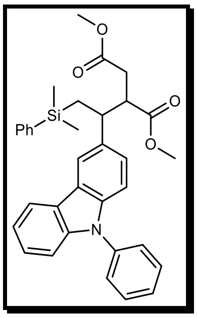

Following the **General procedure A**, **a-22** (73.8.0 mg, 0.2 mmol), **b-1** (64.9 mg, 0.4 mmol) and **c-1** (43.2 mg, 0.3 mmol) were used. Purification by column chromatography using silica with pentane/EtOAc (20:1 to 5:1, v/v) as eluent afforded **d-22** (49.5 mg, 45% yield) as a colorless oil.

^{1}**H NMR** (400 MHz, Chloroform-d) δ 8.16 – 8.06 (m, 1H), 7.79 (d, J = 35.6 Hz, 1H), 7.63 (dt, J = 14.9, 7.3 Hz, 4H), 7.53 – 7.41 (m, 3H), 7.41 – 7.26 (m, 7H), 7.15 (ddd, J = 30.6, 8.4, 1.8 Hz, 1H), 3.72 (d, J = 66.2 Hz, 3H), 3.53 (d, J = 14.5 Hz, 3H), 3.39 – 3.06 (m, 2H), 2.88 – 2.19 (m, 2H), 1.48 (ddd, J = 14.4, 12.2, 7.8 Hz, 1H), 1.26 (dt, J = 14.3, 2.7 Hz, 1H), 0.06 – -0.07 (m, 6H). (see spectrum)

major: ^{13}**C NMR** (101 MHz, Chloroform-d) δ 175.36, 172.63, 141.20, 140.13, 138.91, 137.78, 133.71, 133.56, 129.94, 128.74, 127.63, 127.48, 127.04, 126.08, 126.02, 123.42, 123.25, 120.36, 120.02, 119.99, 109.91, 109.88, 51.89, 51.67, 51.36, 44.46,

35.35, 22.07, -2.15, -3.09. **minor**: **^{13}C NMR** (101 MHz, Chloroform-*d*) δ 174.42, 172.90, 141.17, 140.04, 138.92, 137.83, 133.66, 133.56, 129.92, 128.89, 127.73, 127.43, 127.04, 126.52, 125.97 123.38, 123.22, 120.36, 120.10, 119.93, 109.88, 109.55, 51.80, 51.67, 50.75, 43.67, 33.10, 18.69, -2.15, -3.00. (*see spectrum*)

HRMS (ESI) (m/z): $[M+H]^+$ calculated for $C_{34}H_{36}NO_4Si^+$: 550.2408, found: 550.2401.

dimethyl 2-(1-(9H-carbazol-2-yl)-2-(dimethyl(phenyl)silyl)ethyl)succinate (d-23)

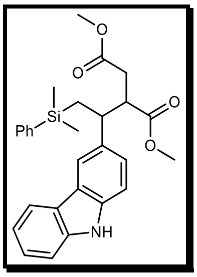

Following the **General procedure A**, **a-23** (58.6 mg, 0.2 mmol), **b-1** (64.9 mg, 0.4 mmol) and **c-1** (43.2 mg, 0.3 mmol) were used. Purification by column chromatography using silica with pentane/EtOAc (20:1 to 3:1, v/v) as eluent afforded **d-23** (35.0 mg, 37% yield) as a white solid.

^{1}H NMR (400 MHz, Chloroform-*d*) δ 7.93 (s, 1H), 7.88 – 7.83 (m, 1H), 7.53 (d, *J* = 33.7 Hz, 1H), 7.22 – 7.05 (m, 8H), 6.96 (dd, *J* = 31.1, 8.4 Hz, 1H), 3.52 (d, *J* = 65.1 Hz, 3H), 3.31 (d, *J* = 25.2 Hz, 3H), 3.14 – 2.85 (m, 2H), 2.69 – 1.99 (m, 2H), 1.26 (td, *J* = 13.0, 12.1, 7.1 Hz, 1H), 1.06 (dt, *J* = 14.6, 3.7 Hz, 1H), -0.16 – -0.33 (m, 6H). (*see spectrum*)

major: ^{13}C NMR (101 MHz, Chloroform-*d*) δ 175.25, 172.51, 139.76, 138.78, 138.59, 133.41, 132.99, 128.73, 127.56, 125.72, 125.81, 123.25, 123.04, 120.20, 119.87, 119.31, 110.56(2), 110.22, 51.64, 51.50, 50.66, 44.33, 35.19, 21.89, -2.25, -3.31. **minor**: **^{13}C NMR** (101 MHz, Chloroform-*d*) δ174.36, 172.78, 139.76, 138.78, 138.51, 133.39, 132.94, 128.60, 127.48, 126.22, 125.69, 123.16, 123.04, 120.20, 119.98, 119.24, 110.56, 110.22, 51.71, 51.46, 51.20, 43.60, 33.02, 18.60, -2.32, -3.44. (*see spectrum*)

HRMS (ESI) (m/z): $[M+H]^+$ calculated for $C_{28}H_{32}NO_4Si^+$: 474.2095, found: 474.2089.

dimethyl 2-(1-(benzofuran-5-yl)-2-(dimethyl(phenyl)silyl)ethyl)succinate (d-24)

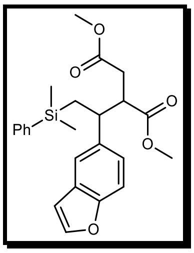

Following the **General procedure A**, **a-24** (39.4.0 mg, 0.2 mmol), **b-1** (64.9 mg, 0.4 mmol) and **c-1** (43.2 mg, 0.3 mmol) were used. Purification by column chromatography using silica with pentane/EtOAc (50:1 to 10:1, v/v) as eluent afforded **d-24** (26.3 mg, 31% yield) as a white solid.

^{1}H NMR (400 MHz, Chloroform-*d*) δ 7.59 (t, *J* = 2.5 Hz, 1H), 7.36 – 7.20 (m, 8H), 6.99 (ddd, *J* = 29.4, 8.5, 1.8 Hz, 1H), 6.70 – 6.63 (m, 1H), 3.66 (d, *J* = 53.1 Hz, 3H), 3.47 (d, *J* = 32.8 Hz, 3H), 3.21 – 2.94 (m, 2H), 2.77 – 2.09 (m, 2H), 1.33 (ddd, *J* = 14.4, 12.2, 2.1 Hz, 1H), 1.16 (ddd, *J* = 14.2, 6.2, 2.7 Hz, 1H), -0.09 (d, *J* = 28.2 Hz, 6H). (*see spectrum*)

major: ^{13}C NMR (101 MHz, Chloroform-*d*) δ 175.13, 172.75, 154.11, 145.38, 138.77, 136.65, 133.46, 128.80, 127.60, 127.57, 124.38, 120.83, 111.36, 106.61, 51.85, 51.67, 51.16, 44.22 35.13, 21.93, -2.35, -3.19. **minor**: **^{13}C NMR** (101 MHz, Chloroform-*d*) δ 174.28, 172.48, 154.17, 145.24, 138.75, 136.58, 133.46, 128.93, 127.69, 127.30, 124.77, 120.81, 111.02, 106.55, 51.79, 51.60, 50.62, 43.54, 33.12, 18.73, -2.35, -3.14. (*see spectrum*)

HRMS (ESI) (m/z): $[M+H]^+$ calculated for $C_{24}H_{29}O_5Si^+$:425.1779, found: 425.1776.

dimethyl 2-(1-([1,1'-biphenyl]-4-yl)-2-(methyldiphenylsilyl)ethyl)succinate (d-25)

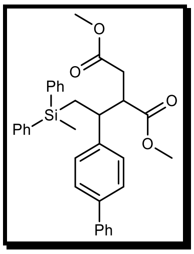

Following the **General procedure A**, **a-1** (56.0 mg, 0.2 mmol), **b-25** (89.4 mg, 0.4 mmol) and **c-1** (43.2 mg, 0.3 mmol) were used. Purification by column chromatography using silica with pentane/EtOAc (50:1 to 10:1, v/v) as eluent afforded **d-25** (64.8 mg, 62% yield) as a colorless oil.

^{1}H NMR (400 MHz, Chloroform-*d*) δ 7.61 (t, *J* = 7.5 Hz, 2H), 7.53 – 7.36 (m, 11H), 7.30 (dt, *J* = 24.4, 7.5 Hz, 4H), 7.10 (dd, *J* = 38.2, 7.9 Hz, 2H), 3.74 (d, *J* = 55.3 Hz, 3H), 3.57 (d, *J* = 21.4 Hz, 3H), 3.30 – 3.01 (m, 2H), 2.91 – 2.18 (m, 2H), 1.65 (qdd, *J* = 14.7, 10.3, 5.0 Hz, 2H), 0.21 (d, *J* = 13.0 Hz, 3H). (see spectrum)

major: ^{13}C NMR (101 MHz, Chloroform-*d*) δ 174.96 172.42, 140.93, 140.82, 139.97, 137.37, 136.22, 134.61, 134.18, 129.33, 128.96, 128.84, 128.79, 127.96, 127.74, 127.32, 127.17, 127.04, 51.94, 51.75, 50.69, 43.80, 35.16, 19.97, -4.36. **minor**: **^{13}C NMR** (101 MHz, Chloroform-*d*) δ 174.14, 172.72, 140.90, 140.86, 139.78, 137.21, 136.22, 134.58, 134.27, 129.39, 129.14, 128.92, 128.84, 127.96, 127.82, 127.29, 127.04, 126.86, 51.86, 51.69, 50.13, 43.06, 32.98, 16.59, -4.18. (see spectrum)

HRMS (ESI) (m/z): $[M+H]^+$ calculated for $C_{33}H_{35}O_4Si^+$: 523.2299, found: 523.2294.

dimethyl 2-(1-([1,1'-biphenyl]-4-yl)-2-(triphenylsilyl)ethyl)succinate (d-26)

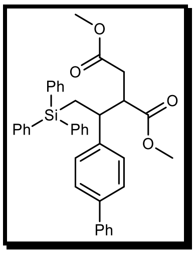

Following the **General procedure A**, **a-1** (56.0 mg, 0.2 mmol), **b-26** (114.6 mg, 0.4 mmol) and **c-1** (43.2 mg, 0.3 mmol) were used. Purification by column chromatography using silica with pentane/EtOAc (50:1 to 10:1, v/v) as eluent afforded **d-26** (29.2 mg, 25% yield) as a colorless oil.

^{1}H NMR (400 MHz, Chloroform-*d*) δ 7.50 (d, *J* = 8.2 Hz, 2H), 7.44 (td, *J* = 7.7, 2.4 Hz, 2H), 7.37 – 7.30 (m, 10H), 7.27 – 7.20 (m, 8H), 6.92 (dd, *J* = 32.1, 8.0 Hz, 2H), 3.67 (d, *J* = 46.7 Hz, 3H), 3.55 (d, *J* = 5.3 Hz, 3H), 3.33 (ddt, *J* = 66.4, 10.2, 4.1 Hz, 1H), 3.16 – 3.08 (m, 1H), 2.67 (ddd, *J* = 118.0, 16.7, 10.9 Hz, 1H), 2.33 (ddd, *J* = 94.5, 16.7, 3.8 Hz, 1H), 2.00 – 1.87 (m, 2H). (see spectrum)

major: ^{13}C NMR (101 MHz, Chloroform-*d*) δ 174.83, 172.39, 141.08, 140.53, 139.79, 135.70, 134.61, 129.36, 128.93, 128.79, 127.79, 127.17, 127.14, 127.02, 51.83, 51.74, 50.66, 43.50, 32.73, 19.20. **minor**: **^{13}C NMR** (101 MHz, Chloroform-*d*) δ174.05,

172.69, 141.01, 140.73, 139.56, 135.64, 134.43, 129.27, 128.93, 128.79, 127.79, 127.21, 127.02, 126.76, 51.94, 51.74, 50.01, 42.76, 34.98, 15.45. (*see spectrum*)

HRMS (ESI) (m/z): $[M+H]^+$ calculated for $C_{38}H_{37}O_4Si^+$: 585.2456, found: 585.2451.

dimethyl 2-(1-([1,1'-biphenyl]-4-yl)-2-(trimethylsilyl)ethyl)succinate (d-27)

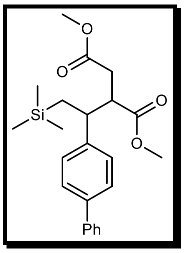

Following the **General procedure A**, **a-1** (56.0 mg, 0.2 mmol), **b-27** (40.1 mg, 0.4 mmol) and **c-1** (43.2 mg, 0.3 mmol) were used. Purification by column chromatography using silica with pentane/EtOAc (50:1 to 10:1, v/v) as eluent afforded **d-27** (51.0 mg, 64% yield) as a colorless oil.

^{1}H NMR (400 MHz, Chloroform-*d*) δ 7.59 (dd, *J* = 7.6, 1.9 Hz, 2H), 7.52 (dd, *J* = 8.3, 2.7 Hz, 2H), 7.43 (td, *J* = 7.6, 1.5 Hz, 2H), 7.33 (td, *J* = 7.2, 1.6 Hz, 1H), 7.22 (dd, *J* = 10.6, 8.2 Hz, 2H), 3.70 (d, *J* = 49.4 Hz, 3H), 3.55 (d, *J* = 13.6 Hz, 3H), 3.19 – 2.94 (m, 2H), 2.82 – 2.21 (m, 2H), 1.12 (ddd, *J* = 14.9, 12.2, 3.0 Hz, 1H), 0.87 (ddd, *J* = 14.2, 10.9, 2.9 Hz, 1H), -0.23 (d, *J* = 18.1 Hz, 9H). (*see spectrum*)

major: ^{13}C NMR (101 MHz, Chloroform-*d*) δ 175.13, 172.53, 141.68, 140.71, 139.95, 128.83, 128.81, 127.32, 127.26, 127.00, 51.85, 51.74, 50.85, 44.14, 35.06, 22.21, -1.23.

minor: **^{13}C NMR** (101 MHz, Chloroform-*d*) δ 174.34, 172.79, 141.57, 140.78 139.71, 128.81, 128.70, 127.32, 127.00, 126.88, 51.82, 51.66, 50.36, 43.48, 33.171, 18.86, -1.16. (*see spectrum*)

HRMS (ESI) (m/z): $[M+H]^+$ calculated for $C_{23}H_{31}O_4Si^+$: 399.1986, found: 399.1989.

dimethyl 2-(1-([1,1'-biphenyl]-4-yl)-2-(triethylsilyl)ethyl)succinate (d-28)

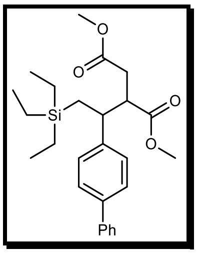

Following the **General procedure A**, **a-1** (56.0 mg, 0.2 mmol), **b-28** (56.9 mg, 0.4 mmol) and **c-1** (43.2 mg, 0.3 mmol) were used. Purification by column chromatography using silica with pentane/EtOAc (50:1 to 10:1, v/v) as eluent afforded **d-28** (44.8 mg, 51% yield) as a white solid.

^{1}H NMR (400 MHz, Chloroform-*d*) δ 7.59 (ddd, *J* = 8.0, 2.7, 1.3 Hz, 2H), 7.52 (d, *J* = 7.9 Hz, 2H), 7.43 (td, *J* = 7.6, 1.6 Hz, 2H), 7.33 (td, *J* = 7.2, 1.6 Hz, 1H), 7.23 (dd, *J* = 10.9, 7.4 Hz, 2H), 3.69 (d, *J* = 47.5 Hz, 3H), 3.55 (d, *J* = 10.4 Hz, 3H), 3.22 – 2.94 (m, 2H), 2.84 – 2.18 (m, 2H), 1.15 – 1.05 (m, 1H), 0.88 (ddd, *J* = 14.5, 6.2, 2.6 Hz, 1H), 0.79 (dt, *J* = 10.8, 7.9 Hz, 9H), 0.35 – 0.15 (m, 6H). (see spectrum)

major: ^{13}C NMR (101 MHz, Chloroform-*d*) δ 175.05, 172.52, 141.91, 140.76, 139.96, 128.83, 128.62, 127.31, 127.19, 127.00, 51.82, 51.73, 51.04, 43.84, 35.09, 16.81, 7.25, 3.36. **minor**: **^{13}C NMR** (101 MHz, Chloroform-*d*) δ 174.32, 172.83, 141.78, 140.79, 139.74, 128.82, 128.71, 127.27, 127.00, 126.84, 51.82, 51.66, 50.46, 43.15, 32.89, 13.28, 7.28, 3.43. (see spectrum)

HRMS (ESI) (m/z): $[M+H]^+$ calculated for $C_{26}H_{37}O_4Si^+$: 441.2456, found: 441.2459.

dimethyl 2-(1-([1,1'-biphenyl]-4-yl)-2-(diethyl(methyl)silyl)ethyl)succinate (d-29)

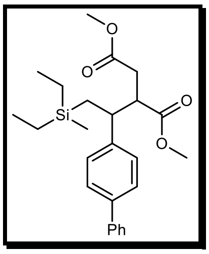

Following the **General procedure A**, **a-1** (56.0 mg, 0.2 mmol), **b-29** (51.3 mg, 0.4 mmol) and **c-1** (43.2 mg, 0.3 mmol) were used. Purification by column chromatography using silica with pentane/EtOAc (50:1 to 10:1, v/v) as eluent afforded **d-29** (44.4 mg, 52% yield) as a colorless oil.

^{1}H NMR (400 MHz, Chloroform-*d*) δ 7.59 (dd, *J* = 7.2, 1.6 Hz, 2H), 7.52 (dd, *J* = 8.2, 1.7 Hz, 2H), 7.43 (t, *J* = 7.6 Hz, 2H), 7.33 (td, *J* = 7.2, 1.6 Hz, 1H), 7.23 (dd, *J* = 11.6, 8.2 Hz, 2H), 3.69 (d, *J* = 48.3 Hz, 3H), 3.55 (d, *J* = 10.9 Hz, 3H), 3.24 – 2.93 (m, 2H), 2.84 – 2.18 (m, 2H), 1.16 – 1.04 (m, 1H), 0.92 – 0.75 (m, 7H), 0.42 – 0.14 (m, 4H), -0.32 (d, *J* = 13.1 Hz, 3H). (see spectrum)

major: ^{13}**C NMR** (101 MHz, Chloroform-*d*) δ 175.07, 172.52, 141.84, 140.74, 139.95, 128.83, 128.65, 127.31, 127.21, 127.00, 51.82, 51.73, 50.97, 43.93, 35.06, 18.51, 7.25, 5.22, 5.07, -5.84. **minor**: ^{13}**C NMR** (101 MHz, Chloroform-*d*) δ 174.32, 172.81, 141.71, 140.78, 139.72, 128.81, 128.75, 127.27, 127.00, 126.86, 51.82, 51.66, 50.42, 43.24, 32.96, 15.01, 7.22, 5.27, 5.14, -5.71. (*see spectrum*)

HRMS (ESI) (m/z): $[M+H]^+$ calculated for $C_{25}H_{35}O_4Si^+$: 427.2299, found: 427.2305.

dimethyl 2-(1-([1,1'-biphenyl]-4-yl)-2-((4-methoxyphenyl)dimethylsilyl)ethyl)succinate (d-30)

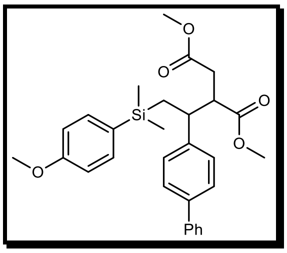

Following the **General procedure A**, **a-1** (56.0 mg, 0.2 mmol), **b-30** (76.9 mg, 0.4 mmol) and **c-1** (43.2 mg, 0.3 mmol) were used. Purification by column chromatography using silica with pentane/EtOAc (40:1 to 10:1, v/v) as eluent afforded **d-30** (45.1 mg, 46% yield) as a colorless oil.

^{1}**H NMR** (400 MHz, Chloroform-*d*) δ 7.58 (ddd, *J* = 7.9, 6.1, 1.3 Hz, 2H), 7.48 – 7.42 (m, 4H), 7.37 – 7.31 (m, 1H), 7.28 – 7.24 (m, 1H), 7.24 – 7.19 (m, 1H), 7.14 (d, *J* = 8.2 Hz, 1H), 7.07 (d, *J* = 8.2 Hz, 1H), 6.87 – 6.80 (m, 2H), 3.78 – 3.62 (m, 6H), 3.52 (d, *J* = 23.1 Hz, 3H), 3.14 – 2.89 (m, 2H), 2.81 – 2.17 (m, 2H), 1.31 (ddd, *J* = 14.1, 12.3, 1.3 Hz, 1H), 1.12 (ddd, *J* = 14.6, 5.8, 3.0 Hz, 1H), 0.05 – -0.06 (m, 6H). (*see spectrum*)

major: ^{13}**C NMR** (101 MHz, Chloroform-*d*) 175.08, 172.50, 160.26, 141.23, 140.81, 139.89, 134.92, 129.48, 128.88, 128.83, 127.31, 127.12, 127.00, 113.48, 55.00, 51.90, 51.75, 50.74, 44.02, 35.06, 33.10, 21.67, -2.21, -2.84. **minor**: ^{13}**C NMR** (101 MHz, Chloroform-*d*) 174.27, 172.78, 160.36, 141.17, 140.76, 139.67, 134.94, 129.43, 128.83, 128.75, 127.27, 127.00, 126.79, 113.53, 55.04, 51.85, 51.67, 50.23, 43.36, 35.06, 33.10, 18.50, -2.17, -2.82. (*see spectrum*)

HRMS (ESI) (m/z): $[M+H]^+$ calculated for $C_{29}H_{35}O_5Si^+$: 491.2248, found: 491.2247.

dimethyl 2-(1-([1,1'-biphenyl]-4-yl)-2-(4,4,5,5-tetramethyl-1,3,2-dioxaborolan-2-

yl)ethyl)succinate (d-31)

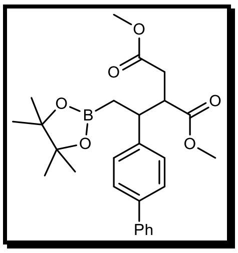

Following the **General procedure A**, **a-1** (56.0 mg, 0.2 mmol), **b-31** (61.6 mg, 0.4 mmol) and **c-1** (43.2 mg, 0.3 mmol) were used. Purification by column chromatography using silica with pentane/EtOAc (30:1 to 5:1, v/v) as eluent afforded **d-31** (48.9 mg, 54% yield) as a colorless oil.

^{1}H NMR (400 MHz, Chloroform-*d*) δ 7.57 (ddd, *J* = 8.0, 4.4, 1.4 Hz, 2H), 7.54 – 7.49 (m, 2H), 7.46 – 7.41 (m, 2H), 7.34 (td, *J* = 7.2, 1.7 Hz, 1H), 7.29 – 7.25 (m, 2H), 3.70 (d, *J* = 49.4 Hz, 3H), 3.58 (d, *J* = 2.6 Hz, 3H), 3.39 – 3.02 (m, 2H), 2.71 (ddd, *J* = 53.6, 16.8, 11.0 Hz, 1H), 2.38 (ddd, *J* = 74.3, 16.8, 3.6 Hz, 1H), 1.33 (ddd, *J* = 15.3, 11.2, 3.7 Hz, 1H), 1.19 (ddd, *J* = 19.7, 15.3, 5.1 Hz, 1H), 1.08 – 0.97 (m, 12H). (*see spectrum*)

major: ^{13}C NMR (101 MHz, Chloroform-*d*) δ 175.02, 172.59, 141.98, 140.95, 139.79, 128.83, 128.64, 127.25, 127.11, 127.01, 83.19, 51.87, 51.76, 49.33, 43.39, 35.04, 24.67, 24.45. **minor**: **^{13}C NMR** (101 MHz, Chloroform-*d*) δ 174.29, 172.76, 141.98, 140.95, 139.56, 128.81, 128.57, 127.21, 127.01, 126.84, 83.28, 51.84, 51.72, 48.78, 42.63, 33.29, 24.71, 24.40. (*see spectrum*)

HRMS (ESI) (m/z): $[M+H]^+$ calculated for $C_{26}H_{34}BO_6^+$: 453.2443, found: 453.2451.

dimethyl 2-(1-([1,1'-biphenyl]-4-yl)-2-cyclohexylethyl)succinate (d-32)

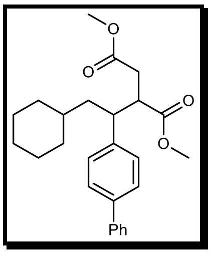

Following the **General procedure B**, **a-1** (56.0 mg, 0.2 mmol), **b-32** (66.1 mg, 0.6 mmol) and **c-1** (43.2 mg, 0.3 mmol) were used. Purification by column chromatography using silica with pentane/EtOAc (50:1 to 10:1, v/v) as eluent afforded **d-32** (40.0 mg, 49% yield) as a colorless oil.

^{1}H NMR (400 MHz, Chloroform-*d*) δ 7.62 – 7.58 (m, 2H), 7.54 (dd, *J* = 8.3, 1.8 Hz, 2H), 7.43 (td, *J* = 7.6, 1.5 Hz, 2H), 7.33 (td, *J* = 7.2, 1.4 Hz, 1H), 7.24 – 7.17 (m, 2H), 3.69 (d, *J* = 49.1 Hz, 3H), 3.56 (d, *J* = 7.7 Hz, 3H), 3.21 – 2.93 (m, 2H), 2.66 (ddd, *J* = 69.8, 16.8, 10.6 Hz, 1H), 2.34 (ddd, *J* = 95.9, 17.0, 3.7 Hz, 1H), 1.80 – 1.51 (m, 6H), 1.41 (dddd, *J* = 19.2, 13.0, 9.2, 3.5 Hz, 1H), 1.15 – 0.87 (m, 5H), 0.86 – 0.74 (m, 1H). (*see spectrum*)

major: ^{13}C NMR (101 MHz, Chloroform-*d*) δ 175.19, 172.56, 140.73, 140.66, 139.70, 128.84, 128.61, 127.36, 127.31, 127.02, 51.91, 51.77, 48.09, 44.65, 41.56, 35.11, 34.56, 34.47, 32.17, 26.59, 26.18, 25.97. **minor**: **^{13}C NMR** (101 MHz, Chloroform-*d*) δ 174.28, 172.78, 140.81, 140.50, 139.56, 128.83, 128.79, 127.26, 127.06, 127.02, 51.86, 51.74, 48.03, 43.80, 38.62, 34.65, 34.51, 33.14, 32.17, 26.59, 26.23, 25.97. (*see spectrum*)

HRMS (ESI) (m/z): $[M+H]^+$ calculated for $C_{26}H_{33}O_4^+$: 409.2373, found: 409.2372.

dimethyl 2-(1-([1,1'-biphenyl]-4-yl)pentyl)succinate (d-33)

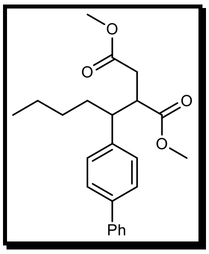

Following the **General procedure B**, **a-1** (56.0 mg, 0.2 mmol), **b-33** (42.1 mg, 0.6 mmol) and **c-1** (43.2 mg, 0.3 mmol) were used Purification by column chromatography using silica with pentane/EtOAc (50:1 to 10:1, v/v) as eluent afforded **d-33** (28.7 mg, 39% yield) as a colorless oil.

^{1}H NMR (400 MHz, Chloroform-*d*) δ 7.63 – 7.56 (m, 2H), 7.53 (dd, *J* = 8.2, 2.5 Hz, 2H), 7.43 (td, *J* = 7.7, 1.6 Hz, 2H), 7.33 (td, *J* = 7.2, 1.5 Hz, 1H), 7.20 (dd, *J* = 10.9, 8.1 Hz, 2H), 3.70 (d, *J* = 50.5 Hz, 3H), 3.56 (d, *J* = 7.5 Hz, 3H), 3.17 – 2.54 (m, 3H), 2.36 (ddd, *J* = 99.4, 16.8, 3.9 Hz, 1H), 1.83 – 1.01 (m, 7H), 0.82 (dt, *J* = 14.6, 7.3 Hz, 3H). (*see spectrum*)

major: ^{13}C NMR (101 MHz, Chloroform-*d*) δ 175.34, 172.56, 140.75, 140.59, 139.80, 128.84, 128.61, 127.38, 127.30, 127.03, 51.94, 51.78, 47.78, 47.73, 35.20, 33.60, 29.57,

22.57, 13.95. **minor**: **^{13}C NMR** (101 MHz, Chloroform-*d*) δ 174.27, 172.75, 140.83, 140.41, 139.64, 128.82, 128.78, 127.26, 127.07, 127.03, 51.87, 51.74, 47.80, 47.01, 33.35, 30.79, 29.78, 22.63, 13.97. (*see spectrum*)

HRMS (ESI) (m/z): $[M+H]^+$ calculated for $C_{23}H_{29}O_4^+$: 369.2060, found: 369.2059.

dimethyl 2-(1-([1,1'-biphenyl]-4-yl)hexyl)succinate (d-34)

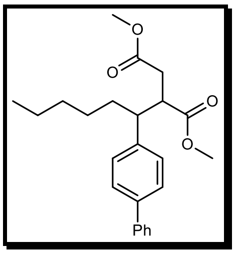

Following the **General procedure B**, **a-1** (56.0 mg, 0.2 mmol), **b-34** (50.5 mg, 0.6 mmol) and **c-1** (43.2 mg, 0.3 mmol) were used. Purification by column chromatography using silica with pentane/EtOAc (50:1 to 10:1, v/v) as eluent afforded **d-34** (36.7 mg, 48% yield) as a colorless oil.

^{1}H NMR (400 MHz, Chloroform-*d*) δ 7.61 – 7.57 (m, 2H), 7.54 (dd, *J* = 8.4, 2.4 Hz, 2H), 7.43 (td, *J* = 7.6, 1.6 Hz, 2H), 7.36 – 7.31 (m, 1H), 7.25 – 7.15 (m, 2H), 3.70 (d, *J* = 50.7 Hz, 3H), 3.56 (d, *J* = 7.2 Hz, 3H), 3.18 – 2.54 (m, 3H), 2.36 (ddd, *J* = 98.4, 16.8, 3.9 Hz, 1H), 1.78 – 1.59 (m, 2H), 1.32 – 1.02 (m, 6H), 0.83 (dtd, *J* = 10.6, 7.4, 3.2 Hz, 3H). (*see spectrum*)

major: ^{13}C NMR (101 MHz, Chloroform-*d*) δ 175.34, 172.56, 140.75, 140.60, 139.79, 128.84, 128.61, 127.37, 127.30, 127.03, 51.94, 51.77, 47.83, 47.78, 35.21, 33.86, 31.72, 27.08, 22.50, 14.09. **minor**: **^{13}C NMR** (101 MHz, Chloroform-*d*) δ 174.26, 172.75, 140.83, 140.42, 139.63, 128.82, 128.78, 127.26, 127.06, 127.03, 51.87, 51.74, 47.73, 47.05, 33.35, 31.78, 31.05, 27.29, 22.50, 14.09. (*see spectrum*)

HRMS (ESI) (m/z): $[M+H]^+$ calculated for $C_{24}H_{31}O_4^+$: 383.2217, found: 383.2209.

dimethyl 2-(1-([1,1'-biphenyl]-4-yl)heptyl)succinate (d-35)

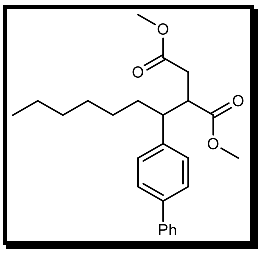

Following the **General procedure B**, **a-1** (56.0 mg, 0.2 mmol), **b-35** (58.9 mg, 0.6 mmol) and **c-1** (43.2 mg, 0.3 mmol) were used. Purification by column chromatography using silica with pentane/EtOAc (50:1 to 10:1, v/v) as eluent afforded **d-35** (40.4 mg, 51% yield) as a white solid.

^{1}H NMR (400 MHz, Chloroform-*d*) δ 7.59 (d, *J* = 7.2 Hz, 2H), 7.54 (dd, *J* = 8.2, 2.3 Hz, 2H), 7.44 (t, *J* = 7.6 Hz, 2H), 7.33 (td, *J* = 7.2, 1.6 Hz, 1H), 7.20 (dd, *J* = 11.1, 8.1 Hz, 2H), 3.70 (d, *J* = 51.2 Hz, 3H), 3.56 (d, *J* = 7.0 Hz, 3H), 3.17 – 2.54 (m, 3H), 2.36 (ddd, *J* = 98.9, 16.8, 3.9 Hz, 1H), 1.81 – 1.56 (m, 2H), 1.31 – 1.12 (m, 7H), 1.07 (p, *J* = 7.0 Hz, 1H), 0.84 (q, *J* = 7.0 Hz, 3H). (see spectrum)

major: ^{13}C NMR (101 MHz, Chloroform-*d*) δ 175.35, 172.56, 140.76, 140.60, 139.79, 128.84, 128.61, 127.31, 127.07, 127.03, 51.95, 51.78, 47.84, 47.78, 35.21, 33.92, 31.68, 29.20, 27.38, 22.64, 14.12. **minor**: **^{13}C NMR** (101 MHz, Chloroform-*d*) δ174.28, 172.76, 140.84, 140.42, 139.63, 128.82, 128.78, 127.38, 127.26, 127.03, 51.88, 47.78, 47.73, 47.05, 33.35, 31.68, 31.09, 29.25, 27.57, 22.67, 14.12. (see spectrum)

HRMS (ESI) (m/z): $[M+H]^+$ calculated for $C_{25}H_{33}O_4^+$: 397.2373, found: 397.2366.

dimethyl 2-(1-([1,1'-biphenyl]-4-yl)octyl)succinate (d-36)

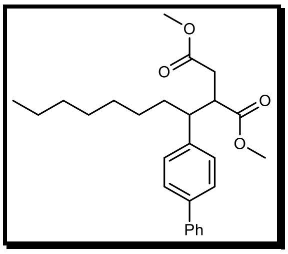

Following the **General procedure B**, **a-1** (56.0 mg, 0.2 mmol), **b-36** (67.3 mg, 0.6 mmol) and **c-1** (43.2 mg, 0.3 mmol) were used. Purification by column chromatography using silica with pentane/EtOAc (50:1 to 10:1, v/v) as eluent afforded **d-36** (32.0 mg, 39% yield) as a white solid.

^{1}H NMR (400 MHz, Chloroform-*d*) δ 7.59 (d, *J* = 7.2 Hz, 2H), 7.54 (dd, *J* = 8.2, 2.4 Hz, 2H), 7.43 (td, *J* = 7.6, 1.6 Hz, 2H), 7.33 (td, *J* = 7.2, 1.5 Hz, 1H), 7.20 (dd, *J* = 11.0, 8.2 Hz, 2H), 3.70 (d, *J* = 50.8 Hz, 3H), 3.56 (d, *J* = 7.2 Hz, 3H), 3.17 – 3.03 (m, 1H), 3.02 – 2.55 (m, 2H), 2.36 (ddd, *J* = 98.7, 16.8, 3.9 Hz, 1H), 1.77 – 1.58 (m, 2H), 1.29 – 1.14 (m, 9H), 1.08 (h, *J* = 7.0, 6.3 Hz, 1H), 0.85 (td, *J* = 7.0, 5.5 Hz, 3H). (see spectrum)

major: ^{13}**C NMR** (101 MHz, Chloroform-*d*) δ 175.34, 172.56, 140.76, 140.60, 139.79, 128.84, 128.61, 127.37, 127.30, 127.03, 51.93, 51.77, 47.83, 47.78, 35.20, 33.90, 31.83, 29.48, 29.14, 27.40, 22.66, 14.13. **minor**: ^{13}**C NMR** (101 MHz, Chloroform-*d*) δ 174.27, 172.75, 140.84, 140.42, 139.63, 128.82, 128.78, 127.25, 127.06, 127.03, 51.87, 51.74, 47.73, 47.05, 33.35, 31.86, 31.08, 29.54, 29.14, 27.61, 22.68, 14.15. (*see spectrum*)

HRMS (ESI) (m/z): $[M+H]^+$ calculated for $C_{26}H_{35}O_4^+$: 411.2530, found: 411.2535.

dimethyl 2-(1-([1,1'-biphenyl]-4-yl)decyl)succinate (d-37)

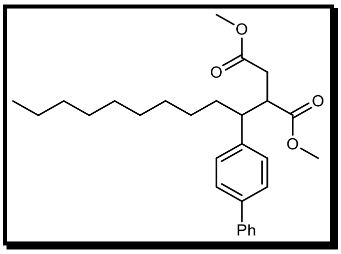

Following the **General procedure B**, **a-1** (56.0 mg, 0.2 mmol), **b-37** (84.2 mg, 0.6 mmol) and **c-1** (43.2 mg, 0.3 mmol) were used. Purification by column chromatography using silica with pentane/EtOAc (50:1 to 10:1, v/v) as eluent afforded **d-37** (44.7 mg, 51% yield) as a white solid.

^{1}**H NMR** (400 MHz, Chloroform-*d*) δ 7.61 – 7.57 (m, 2H), 7.54 (dd, *J* = 8.2, 2.4 Hz, 2H), 7.43 (td, *J* = 7.6, 1.6 Hz, 2H), 7.33 (td, *J* = 7.2, 1.5 Hz, 1H), 7.24 – 7.15 (m, 2H), 3.70 (d, *J* = 50.8 Hz, 3H), 3.56 (d, *J* = 7.4 Hz, 3H), 3.17 – 2.55 (m, 3H), 2.36 (ddd, *J* = 98.8, 16.8, 3.9 Hz, 1H), 1.76 – 1.60 (m, 2H), 1.28 – 1.05 (m, 14H), 0.86 (td, *J* = 6.9, 3.2 Hz, 3H). (*see spectrum*)

major: ^{13}**C NMR** (101 MHz, Chloroform-*d*) δ 175.34, 172.56, 140.75, 140.60, 139.79, 128.83, 128.61, 127.37, 127.30, 127.03, 51.93, 51.77, 47.83, 47.78, 35.20, 33.90, 31.93, 29.57, 29.51, 29.32, 27.40, 22.73, 14.17. **minor**: ^{13}**C NMR** (101 MHz, Chloroform-*d*) δ 174.27, 172.75, 140.84, 140.42, 139.63, 128.81, 128.78, 127.25, 127.06, 127.03, 51.86, 51.74, 47.73, 47.05, 33.35, 31.93, 31.08, 29.60, 29.46, 29.34, 27.59, 22.73, 14.17. (*see spectrum*)

HRMS (ESI) (m/z): $[M+H]^+$ calculated for $C_{28}H_{39}O_4^+$:439.2843, found: 439.2845.

dimethyl 2-(1-([1,1'-biphenyl]-4-yl)-3-(trimethylsilyl)propyl)succinate (d-38)

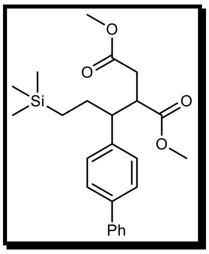

Following the **General procedure B**, **a-1** (56.0 mg, 0.2 mmol), **b-38** (68.6 mg, 0.6 mmol) and **c-1** (43.2 mg, 0.3 mmol) were used. Purification by column chromatography using silica with pentane/EtOAc (50:1 to 10:1, v/v) as eluent afforded **d-38** (28.9 mg, 35% yield) as a colorless oil.

^{1}H NMR (400 MHz, Chloroform-*d*) δ 7.59 (d, *J* = 7.4 Hz, 2H), 7.54 (dd, *J* = 8.2, 2.7 Hz, 2H), 7.47 – 7.40 (m, 2H), 7.33 (td, *J* = 7.5, 1.7 Hz, 1H), 7.18 (t, *J* = 7.6 Hz, 2H), 3.70 (d, *J* = 45.5 Hz, 3H), 3.56 (d, *J* = 10.5 Hz, 3H), 3.21 – 3.05 (m, 1H), 2.96 – 2.54 (m, 2H), 2.36 (ddd, *J* = 92.9, 16.9, 3.9 Hz, 1H), 1.80 – 1.69 (m, 1H), 1.64 (q, *J* = 8.1 Hz, 2H), 0.43 – 0.25 (m, 2H), -0.00 – -0.12 (m, 9H). (*see spectrum*)

major: ^{13}C NMR (101 MHz, Chloroform-*d*) δ 175.36, 172.59, 140.73, 140.37 139.76, 128.83, 128.81, 127.34, 127.30, 127.02, 51.87, 51.78, 50.78, 47.34, 35.06, 28.13, 14.18, -1.80. **minor**: **^{13}C NMR** (101 MHz, Chloroform-*d*) δ 174.20, 172.74, 140.82, 140.27, 139.62, 128.92, 128.80, 127.25, 127.06, 127.02, 51.87, 51.70, 50.30, 47.30, 33.51, 25.50, 14.18, -1.74. (*see spectrum*)

HRMS (ESI) (m/z): $[M+H]^+$ calculated for $C_{24}H_{33}O_4Si^+$: 413.2133, found: 413.2139.

dimethyl 2-(1-([1,1'-biphenyl]-4-yl)-4-phenylbutyl)succinate (d-39)

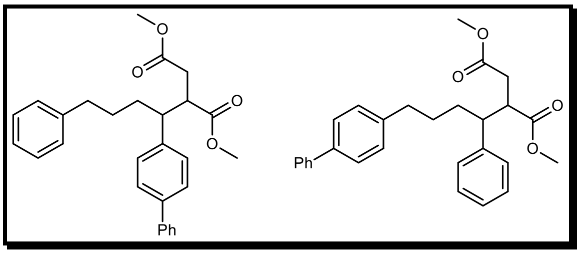

Following the **General procedure B**, **a-1** (56.0 mg, 0.2 mmol), **b-39** (79.2 mg, 0.6 mmol) and **c-1** (43.2 mg, 0.3 mmol) were used. Purification by column chromatography using silica with pentane/EtOAc (50:1 to 10:1, v/v) as eluent afforded **d-39** (48.2 mg, 56% yield) as a colorless oil.

^{1}H NMR (400 MHz, Chloroform-*d*) δ 7.65 – 7.57 (m, 2H), 7.56 – 7.48 (m, 2H), 7.47 – 7.27 (m, 4H), 7.23 (d, *J* = 7.6 Hz, 1H), 7.21 – 7.04 (m, 5H), 3.69 (d, *J* = 36.7 Hz, 3H), 3.56 (d, *J* = 14.1 Hz, 3H), 3.17 – 2.81 (m, 2H), 2.80 – 2.50 (m, 3H), 2.49 – 2.14 (m, 1H), 1.81 – 1.65 (m, 2H), 1.55 – 1.38 (m, 2H). (*see spectrum*)

major: ^{13}C NMR (101 MHz, Chloroform-*d*) δ 175.26, 172.50, 142.15, 140.72, 140.26, 140.06, 128.86, 128.78, 128.45, 128.32, 127.47, 127.35, 127.05, 125.80, 51.95, 51.80, 47.74, 47.71, 35.68, 35.23, 33.44, 29.11. **minor**: **^{13}C NMR** (101 MHz, Chloroform-*d*) δ 174.16, 172.68, 142.10, 140.81, 139.94, 139.75, 128.84, 128.61, 128.45, 128.36, 127.29, 127.13, 127.05, 125.85, 51.89, 51.77, 47.68, 46.94, 35.68, 33.36, 30.60, 29.28. (*see spectrum*)

HRMS (ESI) (m/z): $[M+H]^+$ calculated for $C_{28}H_{31}O_4^+$: 431.2217, found: 431.2212.

dimethyl 2-(1-([1,1'-biphenyl]-4-yl)-5-oxohexyl)succinate (d-40)

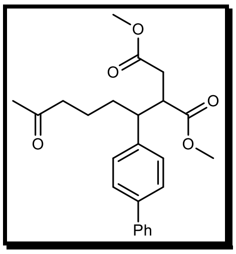

Following the **General procedure B**, **a-1** (56.0 mg, 0.2 mmol), **b-40** (58.8 mg, 0.6 mmol) and **c-1** (43.2 mg, 0.3 mmol) were used. Purification by column chromatography using silica with pentane/EtOAc (50:1 to 5:1, v/v) as eluent afforded **d-40** (34.8 mg, 44% yield) as a colorless oil.

^{1}H NMR (400 MHz, Chloroform-*d*) δ 7.60 – 7.50 (m, 4H), 7.43 (t, *J* = 7.4 Hz, 2H), 7.37 – 7.31 (m, 1H), 7.20 (dd, *J* = 10.8, 8.0 Hz, 2H), 3.69 (d, *J* = 48.9 Hz, 3H), 3.56 (d, *J* = 3.4 Hz, 3H), 3.16 – 2.78 (m, 2H), 2.66 (ddd, *J* = 64.0, 16.8, 10.9 Hz, 1H), 2.49 – 2.20 (m, 3H), 2.07 (d, *J* = 12.3 Hz, 3H), 1.79 – 1.57 (m, 2H), 1.50 – 1.34 (m, 2H). (*see spectrum*)

major: ^{13}C NMR (101 MHz, Chloroform-*d*) δ208.63, 175.10, 172.41, 140.67, 139.91, 139.72, 128.85, 128.58, 127.54, 127.35, 127.05, 52.01, 51.80, 47.73, 47.67, 43.38, 33.27, 29.95, 29.88, 21.66. **minor**: **^{13}C NMR** (101 MHz, Chloroform-*d*) δ 208.61,

174.04, 172.61, 140.76, 140.09, 139.89, 128.82, 128.75, 127.30, 127.20, 127.05, 51.89, 51.80, 47.60, 46.97, 43.36, 35.19, 30.55, 29.76, 21.87. (see spectrum)

HRMS (ESI) (m/z): $[M+H]^+$ calculated for $C_{24}H_{29}O_5^+$: 397.2010, found: 397.2015.

6-ethyl 1,2-dimethyl 3-([1,1'-biphenyl]-4-yl)hexane-1,2,6-tricarboxylate (d-41)

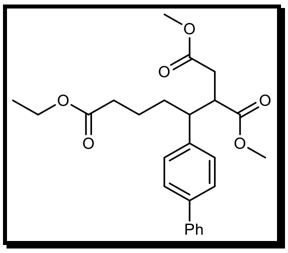

Following the **General procedure B**, **a-1** (56.0 mg, 0.2 mmol), **b-41** (76.8 mg, 0.6 mmol) and **c-1** (43.2 mg, 0.3 mmol) were used. Purification by column chromatography using silica with pentane/EtOAc (50:1 to 10:1, v/v) as eluent afforded **d-41** (44.3 mg, 52% yield) as a colorless oil.

^{1}H NMR (400 MHz, Chloroform-*d*) δ7.55 (dd, *J* = 16.4, 7.2 Hz, 4H), 7.43 (t, *J* = 7.6 Hz, 2H), 7.34 (d, *J* = 7.5 Hz, 1H), 7.20 (t, *J* = 9.3 Hz, 2H), 4.08 (dt, *J* = 14.5, 7.2 Hz, 2H), 3.69 (d, *J* = 48.6 Hz, 3H), 3.56 (d, *J* = 4.0 Hz, 3H), 3.16 – 2.80 (m, 2H), 2.67 (ddd, *J* = 65.1, 16.9, 10.9 Hz, 1H), 2.50 – 2.19 (m, 3H), 1.86 – 1.64 (m, 2H), 1.57 – 1.39 (m, 2H), 1.22 (q, *J* = 7.7 Hz, 3H). (see spectrum)

major: ^{13}C NMR (101 MHz, Chloroform-*d*) δ 175.06, 173.35, 172.41, 140.68, 140.07, 139.88, 128.84, 128.57, 127.54, 127.35, 127.04, 60.32, 51.80, 47.73, 47.63, 47.60, 35.16, 33.28, 22.89, 14.27. **minor**: **^{13}C NMR** (101 MHz, Chloroform-*d*) δ 174.03, 173.38, 172.60, 140.76, 139.88, 139.69, 128.83, 128.74, 127.30, 127.20, 127.04, 60.32, 51.99, 51.89, 51.80, 46.85, 34.08, 30.55, 23.05, 14.30. (see spectrum)

HRMS (ESI) (m/z): $[M+H]^+$ calculated for $C_{25}H_{31}O_6^+$: 427.2115, found: 427.2122.

dimethyl 2-(1-([1,1'-biphenyl]-4-yl)-4-chlorobutyl)succinate (d-42)

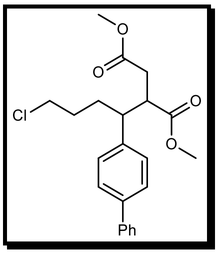

Following the **General procedure B**, **a-1** (56.0 mg, 0.2 mmol), **b-42** (54.0 mg, 0.6 mmol) and **c-1** (43.2 mg, 0.3 mmol) were used. Purification by column chromatography using silica with pentane/EtOAc (50:1 to 10:1, v/v) as eluent afforded **d-42** (25.6 mg, 33% yield) as a colorless oil.

^{1}H NMR (400 MHz, Chloroform-*d*) δ 7.60 – 7.52 (m, 4H), 7.44 (t, *J* = 7.4 Hz, 2H), 7.37 – 7.32 (m, 1H), 7.20 (t, *J* = 8.4 Hz, 2H), 3.71 (d, *J* = 52.2 Hz, 3H), 3.57 (d, *J* = 4.2 Hz, 3H), 3.47 (dt, *J* = 22.8, 6.4 Hz, 2H), 3.19 – 2.81 (m, 2H), 2.68 (ddd, *J* = 68.0, 16.8, 10.8 Hz, 1H), 2.37 (ddd, *J* = 104.6, 16.8, 4.0 Hz, 1H), 1.94 – 1.78 (m, 2H), 1.70 – 1.53 (m, 3H). (*see spectrum*)

major: ^{13}C NMR (101 MHz, Chloroform-*d*) δ 173.79, 172.20, 140.42, 140.06, 139.54, 128.71, 128.37, 127.46, 127.26, 126.88, 51.92, 51.67, 47.54, 47.09, 44.63, 35.07, 31.01, 30.27. **minor**: **^{13}C NMR** (101 MHz, Chloroform-*d*) δ 174.87, 172.40, 140.51, 139.86, 139.29, 128.69, 128.55, 127.21, 127.12, 126.88, 51.77, 51.69, 47.46, 46.21, 44.66, 33.17, 30.32, 28.23. (*see spectrum*)

HRMS (ESI) (m/z): $[M+H]^+$ calculated for $C_{22}H_{26}ClO_4^+$: 389.1514, found: 389.1510.

dimethyl 2-(1-([1,1'-biphenyl]-4-yl)-5-(4,4,5,5-tetramethyl-1,3,2-dioxaborolan-2-yl)pentyl)succinate (d-43)

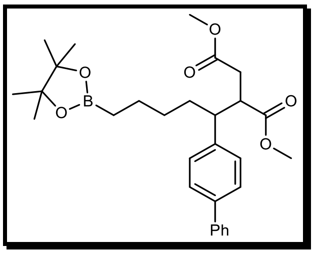

Following the **General procedure B**, **a-1** (56.0 mg, 0.2 mmol), **b-44** (117.6 mg, 0.6 mmol) and **c-1** (43.2 mg, 0.3 mmol) were used. Purification by column chromatography using silica with pentane/EtOAc (50:1 to 10:1, v/v) as eluent afforded **d-43** (47.4 mg, 48% yield) as a colorless oil.

^{1}H NMR (400 MHz, Chloroform-*d*) δ 7.57 (d, *J* = 8.6 Hz, 2H), 7.51 (dd, *J* = 8.2, 2.6 Hz, 2H), 7.45 – 7.40 (m, 2H), 7.33 (td, *J* = 7.2, 1.6 Hz, 1H), 7.19 (dd, *J* = 10.4, 8.1 Hz, 2H), 3.69 (d, *J* = 49.5 Hz, 3H), 3.55 (d, *J* = 10.5 Hz, 3H), 3.14 – 2.77 (m, 2H), 2.77 – 2.53 (m, 1H), 2.35 (ddd, *J* = 102.2, 16.8, 3.8 Hz, 1H), 1.74 (ddd, *J* = 16.3, 8.2, 3.7 Hz,

1H), 1.65 – 1.56 (m, 1H), 1.47 – 1.25 (m, 3H), 1.17 (d, J = 7.0 Hz, 12H), 1.07 (q, J = 7.4, 6.3 Hz, 1H), 0.69 (dt, J = 15.3, 7.7 Hz, 2H). (see spectrum)

major: ^{13}C NMR (101 MHz, Chloroform-*d*) δ 175.33, 172.54, 140.81, 140.61, 139.77, 128.79, 128.62, 127.36, 127.25, 127.01, 82.90, 51.93, 51.75, 47.78, 47.74, 35.20, 33.79, 30.08, 24.79, 24.02. **minor**: **^{13}C NMR** (101 MHz, Chloroform-*d*) δ 174.26, 172.73, 140.89, 140.41, 139.60, 128.81, 128.62, 127.36, 127.20, 127.02, 82.93, 51.84, 51.71, 47.82, 47.01, 33.41, 31.02, 30.23, 24.79, 24.02. (see spectrum)

HRMS (ESI) (m/z): $[M+H]^+$ calculated for $C_{29}H_{40}BO_6^+$: 495.2912, found: 495.2919.

dimethyl 2-(1-([1,1'-biphenyl]-4-yl)-3-(diethoxyphosphoryl)propyl)succinate (d-44)

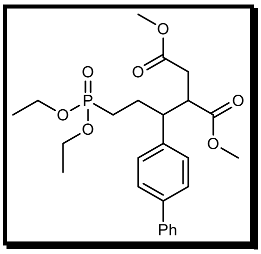

Following the **General procedure B**, **a-1** (56.0 mg, 0.2 mmol), **b-43** (106.8 mg, 0.6 mmol) and **c-1** (43.2 mg, 0.3 mmol) were used. Purification by column chromatography using silica with pentane/EtOAc (50:1 to 30:1, v/v) as eluent afforded **d-44** (40.0 mg, 42% yield) as a colorless oil.

^{1}H NMR (400 MHz, Chloroform-*d*) δ 7.61 – 7.50 (m, 4H), 7.43 (td, J = 7.7, 2.2 Hz, 2H), 7.37 – 7.30 (m, 1H), 7.18 (dd, J = 8.1, 3.7 Hz, 2H), 4.04 (dddd, J = 15.4, 14.0, 7.2, 4.0 Hz, 4H), 3.70 (d, J = 49.2 Hz, 3H), 3.55 (d, J = 9.9 Hz, 3H), 3.18 – 3.05 (m, 1H), 3.03 – 2.83 (m, 1H), 2.67 (ddd, J = 65.8, 16.8, 10.8 Hz, 1H), 2.37 (ddd, J = 108.3, 16.8, 4.0 Hz, 1H), 2.10 – 1.90 (m, 2H), 1.63 – 1.45 (m, 2H), 1.32 – 1.25 (m, 6H). (see spectrum)

major: ^{13}C NMR (101 MHz, Chloroform-*d*) δ 174.83, 173.73, 172.43, 172.27, 140.62, 140.53, 140.42, 140.20, 138.83, 138.66, 128.88, 128.86, 128.77, 128.64, 127.72, 127.46, 127.40, 127.36, 127.04, 61.64 (J = 6.2 Hz), 52.08, 51.84, 48.19 (J = 18.3 Hz), 47.35, 35.17, 26.79 (J = 3.3 Hz), 23.93 (J = 142.4 Hz), 16.49 (J = 1.9 Hz). **minor**: **^{13}C NMR** (101 MHz, Chloroform-*d*) δ 174.83, 173.73, 172.43, 172.27, 140.62, 140.53,

140.42, 140.20, 138.83, 138.66, 128.88, 128.86, 128.77, 128.64, 127.72, 127.46, 127.40, 127.36, 127.04, 61.71 (J = 6.6 Hz), 51.94, 51.84, 47.68 (J = 17.9 Hz), 47.35, 33.56, 24.49 (J = 3.7 Hz), 23.70 (J = 142.4 Hz), 16.49 (J = 9.6 Hz). (*see spectrum*)

major: ^{31}P NMR (162 MHz, Chloroform-*d*) δ 31.54. **minor**: **^{31}P NMR** (162 MHz, Chloroform-*d*) δ 31.62. (*see spectrum*)

HRMS (ESI) (m/z): $[M+H]^+$ calculated for $C_{25}H_{34}O_7P^+$: 477.2037, found: 477.2039.

dimethyl 2-([1,1'-biphenyl]-4-yl(cyclohexyl)methyl)succinate (d-45)

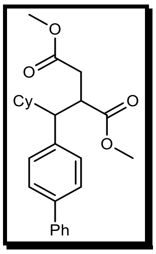

Following the **General procedure B**, **a-1** (56.0 mg, 0.2 mmol), **b-45** (57.6 mg, 0.6 mmol) and **c-1** (43.2 mg, 0.3 mmol) were used. Purification by column chromatography using silica with pentane/EtOAc (50:1 to 10:1, v/v) as eluent afforded **d-45** (23.7 mg, 30% yield) as a colorless oil.

^{1}H NMR (400 MHz, Chloroform-*d*) δ 7.58 (d, J = 8.2 Hz, 2H), 7.50 (dd, J = 8.1, 5.6 Hz, 2H), 7.43 (td, J = 7.7, 2.1 Hz, 2H), 7.36 – 7.30 (m, 1H), 7.11 (dd, J = 8.3, 6.6 Hz, 2H), 3.69 (d, J = 18.0 Hz, 3H), 3.59 (d, J = 17.3 Hz, 3H), 3.52 – 3.43 (m, 1H), 2.89 – 2.57 (m, 2H), 2.39 (ddd, J = 56.2, 16.8, 4.0 Hz, 1H), 2.14 – 1.83 (m, 1H), 1.81 – 1.50 (m, 6H), 1.19 – 0.99 (m, 2H), 0.96 – 0.79 (m, 2H). (*see spectrum*)

major: ^{13}C NMR (101 MHz, Chloroform-*d*) δ 175.34, 172.88, 140.76, 139.65, 138.26, 129.60, 128.81, 127.26, 127.02, 126.89, 53.17, 51.86, 51.83, 43.49, 39.85, 33.78, 31.89, 29.29, 26.53, 26.52, 26.36. **minor**: **^{13}C NMR** (101 MHz, Chloroform-*d*) δ 174.04, 172.72, 140.86, 139.52, 139.17, 129.58, 128.78, 127.19, 127.02, 126.70, 54.30, 51.86, 51.62, 42.72, 38.94, 35.10, 31.86, 30.81, 26.60, 26.49, 26.40. (*see spectrum*)

HRMS (ESI) (m/z): $[M+H]^+$ calculated for $C_{25}H_{31}O_4^+$: 395.2217, found: 395.2211.

2-(2-([1,1'-biphenyl]-4-yl)-3-(dimethyl(phenyl)silyl)-1-phenylpropyl)malononitrile (d-46)

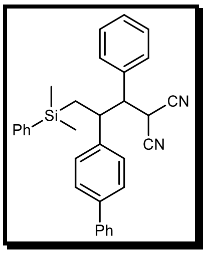

Following the **General procedure B**, **a-1** (56.0 mg, 0.2 mmol), **b-1** (64.9 mg, 0.4 mmol) and **c-46** (46.3 mg, 0.3 mmol) were used. Purification by column chromatography using silica with pentane/EtOAc (100:1 to 20:1, v/v) as eluent afforded **d-46** (45.2 mg, 48% yield) as a white solid.

major: ^{1}H NMR (400 MHz, Chloroform-*d*) δ 7.59 (dd, *J* = 17.3, 7.5 Hz, 4H), 7.48 (q, *J* = 7.1, 6.4 Hz, 5H), 7.41 (dd, *J* = 16.5, 7.6 Hz, 3H), 7.30 (dq, *J* = 15.2, 7.3 Hz, 6H), 7.20 (d, *J* = 6.2 Hz, 2H), 3.57 (d, *J* = 3.7 Hz, 1H), 3.36 – 3.21 (m, 2H), 1.09 – 0.97 (m, 2H), -0.07 (d, *J* = 4.7 Hz, 6H). (*see spectrum*)

^{13}C NMR (101 MHz, Chloroform-*d*) δ 141.16, 140.30, 140.21, 137.97, 135.69, 133.44, 129.41, 129.22, 129.08, 128.98, 128.85, 128.16, 127.81, 127.70, 127.10, 112.32, 111.42, 77.43, 77.11, 76.79, 55.29, 43.45, 28.69, 21.66, -2.48, -3.36. (*see spectrum*)

minor: **^{1}H NMR** (400 MHz, Chloroform-*d*) δ 7.54 (dd, *J* = 7.5, 1.7 Hz, 2H), 7.43 (t, *J* = 7.6 Hz, 2H), 7.38 – 7.28 (m, 8H), 7.28 – 7.21 (m, 4H), 6.98 – 6.90 (m, 2H), 6.84 (d, *J* = 8.0 Hz, 2H), 4.09 (d, *J* = 8.3 Hz, 1H), 3.57 – 3.34 (m, 2H), 1.31 – 1.22 (m, 2H), 0.09 (s, 6H). (*see spectrum*)

^{13}C NMR (101 MHz, Chloroform-*d*) δ 7.54 (dd, *J* = 7.5, 1.7 Hz, 2H), 7.43 (t, *J* = 7.6 Hz, 2H), 7.38 – 7.28 (m, 8H), 7.28 – 7.21 (m, 4H), 6.98 – 6.90 (m, 2H), 6.84 (d, *J* = 8.0 Hz, 2H), 4.09 (d, *J* = 8.3 Hz, 1H), 3.57 – 3.34 (m, 2H), 1.31 – 1.22 (m, 2H), 0.09 (s, 6H). (*see spectrum*)

HRMS (ESI) (m/z): $[M+H]^+$ calculated for $C_{32}H_{31}N_2Si^+$: 471.2251, found: 471.2258.

methyl 4-([1,1'-biphenyl]-4-yl)-5-(dimethyl(phenyl)silyl)-2-phenylpentanoate (d-47)

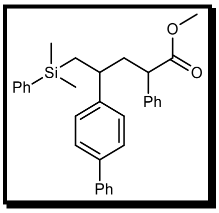

Following the **General procedure C**, **a-1** (56.0 mg, 0.2 mmol), **b-1** (64.9 mg, 0.4 mmol) and **c-47** (48.6 mg, 0.3 mmol) were used. Purification by column chromatography using silica with pentane/EtOAc (100:1 to 20:1, v/v) as eluent afforded **d-47** (46.0 mg, 48 % yield) as a colorless oil.

major: ^{1}H NMR (400 MHz, Chloroform-*d*) δ 7.60 (d, *J* = 8.1 Hz, 2H), 7.50 – 7.42 (m, 4H), 7.37 – 7.26 (m, 9H), 7.12 (dd, *J* = 41.5, 7.8 Hz, 4H), 3.49 (s, 3H), 3.31 (dd, *J* = 9.7, 5.5 Hz, 1H), 2.49 (dq, *J* = 10.5, 5.4 Hz, 1H), 2.36 (ddd, *J* = 13.3, 10.2, 5.5 Hz, 1H), 2.22 (ddd, *J* = 14.0, 9.7, 4.6 Hz, 1H), 1.25 – 1.13 (m, 2H), 0.05 (s, 6H). (see spectrum)

^{13}C NMR (101 MHz, Chloroform-*d*) δ 174.48, 144.91, 141.09, 139.22, 138.60, 133.52, 128.80, 128.75, 128.63, 128.39, 128.22, 127.70, 127.32, 127.12, 127.09, 127.02, 51.92, 49.50, 43.26, 38.83, 25.12, -2.30, -2.82. (see spectrum)

minor: **^{1}H NMR** (400 MHz, Chloroform-*d*) δ 7.60 (d, *J* = 7.8 Hz, 2H), 7.51 – 7.37 (m, 7H), 7.37 – 7.28 (m, 5H), 7.24 – 7.19 (m, 2H), 7.12 (dd, *J* = 15.0, 7.9 Hz, 4H), 3.65 (s, 3H), 3.36 (dd, *J* = 10.6, 4.7 Hz, 1H), 2.72 (dp, *J* = 10.4, 5.2 Hz, 1H), 2.51 (td, *J* = 12.1, 10.3, 4.8 Hz, 1H), 1.99 – 1.86 (m, 1H), 1.28 – 1.17 (m, 2H), 0.10 (dd, *J* = 26.8, 1.9 Hz, 6H). (see spectrum)

^{13}C NMR (101 MHz, Chloroform-*d*) δ 174.35, 145.18, 141.07, 139.45, 139.21, 133.58, 129.40, 128.78, 128.75, 128.65, 127.98, 127.76, 127.71, 127.16, 127.11, 127.02, 51.97, 49.55, 44.44, 39.78, 24.67, -2.29, -2.84. (see spectrum)

HRMS (ESI) (m/z): $[M+H]^+$ calculated for $C_{32}H_{35}O_2Si^+$: 479.2401, found: 479.2397.

(2,4-di([1,1'-biphenyl]-4-yl)-5,5-difluoropent-4-en-1-yl)dimethyl(phenyl)silane (d-48)

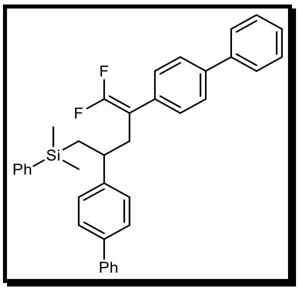

Following the **General procedure C**, **a-1** (56.0 mg, 0.2 mmol), **b-1** (64.9 mg, 0.4 mmol) and **c-48** (74.5 mg, 0.3 mmol) were used. Purification by column chromatography using silica with pentane as eluent afforded **d-48** (76.2 mg, 70% yield) as a white solid.

^{1}H NMR (400 MHz, Chloroform-*d*) δ 7.71 – 7.57 (m, 6H), 7.55 – 7.29 (m, 14H), 7.25 (d, *J* = 7.9 Hz, 2H), 7.12 (d, *J* = 7.8 Hz, 2H), 2.87 – 2.67 (m, 3H), 1.35 – 1.24 (m, 2H), 0.07 (d, *J* = 3.5 Hz, 6H). (*see spectrum*)

^{13}C NMR (101 MHz, Chloroform-*d*) δ 154.2824 (dd, *J* = 292.1 Hz, 288.2 Hz), 140.70, 139.96, 139.19, 139.15, 133.55, 132.44 (t, *J* = 3.8 Hz), 128.92, 128.88, 128.85, 128.82, 128.80, 128.06, 127.76, 127.49, 127.33, 127.12, 127.08, 127.06, 126.90, 91.14 (dd, *J* = 21.9 Hz, 13.3 Hz), 39.58, 38.43, 23.01, -2.47 (d, *J* = 92.2 Hz). (*see spectrum*)

^{19}F NMR (376 MHz, Chloroform-d) δ -90.54 (d, *J* = 41.4 Hz), -91.32 (d, *J* = 41.4 Hz) (*see spectrum*)

HRMS (ESI) (m/z): $[M+H]^+$ calculated for $C_{37}H_{35}F_2Si^+$: 545.2471, found: 545.2480.

(2-([1,1'-biphenyl]-4-yl)-4-(4-(tert-butyl)phenyl)-5,5-difluoropent-4-en-1-yl)dimethyl(phenyl)silane (d-49)

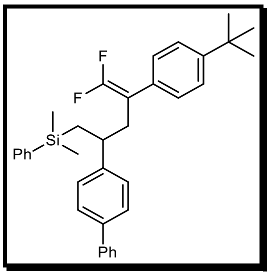

Following the **General procedure C**, **a-1** (56.0 mg, 0.2 mmol), **b-1** (64.9 mg, 0.4 mmol) and **c-49** (68.5 mg, 0.3 mmol) were used. Purification by column chromatography using silica with pentane as eluent afforded **d-49** (57.7 mg, 55% yield) as a colorless oil.

^{1}H NMR (400 MHz, Chloroform-*d*) δ 7.60 (d, *J* = 7.2 Hz, 2H), 7.45 (t, *J* = 7.8 Hz, 4H), 7.37 – 7.26 (m, 8H), 7.08 (dd, *J* = 9.5, 7.8 Hz, 4H), 2.69 (d, *J* = 10.4 Hz, 3H), 1.36 (s, 9H), 1.30 – 1.25 (m, 1H), 1.20 (dd, *J* = 14.8, 9.5 Hz, 1H). (*see spectrum*)

^{13}C NMR (101 MHz, Chloroform-*d*) δ154.24 (dd, *J* = 291.2 Hz, 287.4 Hz), 150.10, 144.99, 141.15, 139.22, 139.11, 133.54, 130.36 (t, *J* = 3.9 Hz), 128.76, 128.74, 128.09, 128.06, 128.03, 127.70, 127.08, 127.03, 126.84, 125.30, 91.07 (dd, *J* = 21.4 Hz, 13.4 Hz), 39.48, 38.59, 34.58, 31.39, 22.77, -2.52 (d, *J* = 93.7 Hz). (*see spectrum*)

^{19}F NMR (376 MHz, Chloroform-d) δ -91.28 (d, *J* = 44.0 Hz), -91.96 (d, *J* = 43.0 Hz). (*see spectrum*)

HRMS (ESI) (m/z): $[M+H]^+$ calculated for $C_{35}H_{39}F_2Si^+$: 525.2784, found: 525.2775.

(2-([1,1'-biphenyl]-4-yl)-5,5-difluoro-4-(4-fluorophenyl)pent-4-en-1-yl)dimethyl(phenyl)silane (d-50)

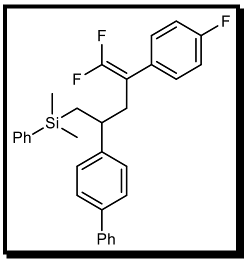

Following the **General procedure C**, **a-1** (56.0 mg, 0.2 mmol), **b-1** (64.9 mg, 0.4 mmol) and **c-50** (57.0 mg, 0.3 mmol) were used. Purification by column chromatography using silica with pentane as eluent afforded **d-50** (50.5 mg, 52% yield) as a white solid.

^{1}H NMR (400 MHz, Chloroform-*d*) δ 7.63 – 7.56 (m, 2H), 7.44 (ddd, *J* = 10.3, 5.8, 1.6 Hz, 4H), 7.37 – 7.26 (m, 6H), 7.09 – 6.93 (m, 6H), 2.64 (s, 3H), 1.29 – 1.16 (m, 2H), 0.02 (s, 6H). (*see spectrum*)

^{13}C NMR (101 MHz, Chloroform-*d*) δ 161.84 (d, *J* = 247.8 Hz), 154.15 (dd, *J* = 292.3 Hz, 288.5 Hz), 154.13, 151.29, 144.66, 141.06, 139.26, 139.04, 133.52, 130.14 (dt, *J* = 8.0 Hz, 2.8 Hz), 129.33 (dd, *J* = 7.4 Hz, 3.4 Hz), 129.35, 129.30, 129.27, 128.86, 128.80, 127.97, 127.76, 127.14, 127.03, 126.92, 115.38 (d, *J* = 21.6 Hz), 115.27, 90.60 (dd, *J* = 22.4 Hz, 13.9 Hz), 39.44, 38.53, 23.17, -2.54 (d, *J* = 89.7 Hz). (*see spectrum*)

^{19}F NMR (376 MHz, Chloroform-d) δ -91.16 (d, *J* = 42.5 Hz), -91.96 (d, *J* = 42.5 Hz), -114.75 (q, *J* = 7.1 Hz). (*see spectrum*)

HRMS (ESI) (m/z): $[M+H]^+$ calculated for $C_{31}H_{30}F_3Si^+$: 487.2063, found: 487.2066.

(2-([1,1'-biphenyl]-4-yl)-4-(4-chlorophenyl)-5,5-difluoropent-4-en-1-yl)dimethyl(phenyl)silane (d-51)

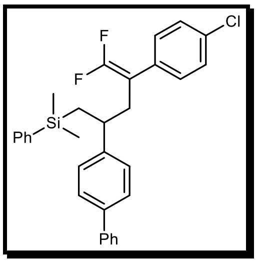

Following the **General procedure C**, **a-1** (56.0 mg, 0.2 mmol), **b-1** (64.9 mg, 0.4 mmol) and **c-51** (62.0 mg, 0.3 mmol) were used. Purification by column chromatography using silica with pentane as eluent afforded **d-51** (48.2 mg, 48% yield) as a colorless oil.

^{1}H NMR (400 MHz, Chloroform-*d*) δ 7.64 – 7.59 (m, 2H), 7.46 (td, *J* = 7.8, 2.6 Hz, 4H), 7.39 – 7.26 (m, 8H), 7.03 (td, *J* = 6.1, 2.9 Hz, 4H), 2.66 (t, *J* = 2.8 Hz, 3H), 1.30 – 1.18 (m, 2H), 0.04 (s, 6H). (*see spectrum*)

^{13}C NMR (101 MHz, Chloroform-*d*) δ 154.18 (dd, *J* = 292.4 Hz, 288.5 Hz), 154.19, 154.16, 151.30, 144.58, 141.06, 139.32, 139.00, 133.54, 133.02, 131.94 (t, *J* = 3.9 Hz), 129.80 (t, *J* = 3.2 Hz), 128.90, 128.82, 128.64, 127.97, 127.80, 127.17, 127.06, 126.95, 90.66 (dd, *J* = 22.5 Hz, 13.3 Hz), 39.51, 38.30, 23.21, -2.1 (d, *J* = 91.0 Hz). (*see spectrum*)

^{19}F NMR (376 MHz, Chloroform-d) δ -90.27 (d, *J* = 40.5 Hz), -91.11 (d, *J* = 40.1 Hz). (*see spectrum*)

HRMS (ESI) (m/z): $[M+H]^+$ calculated for $C_{31}H_{30}ClF_2Si^+$: 503.1768, found: 503.1760.

(2-([1,1'-biphenyl]-4-yl)-4-(4-bromophenyl)-5,5-difluoropent-4-en-1-yl)dimethyl(phenyl)silane (d-52)

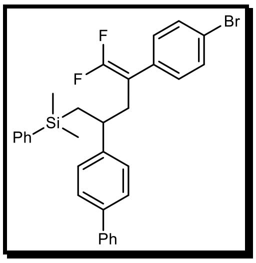

Following the **General procedure C**, **a-1** (56.0 mg, 0.2 mmol), **b-1** (64.9 mg, 0.4 mmol) and **c-52** (75.3 mg, 0.3 mmol) were used. Purification by column chromatography using silica with pentane as eluent afforded **d-52** (21.9 mg, 20% yield) as a white solid.

^{1}H NMR (400 MHz, Chloroform-*d*) δ 7.59 (d, *J* = 7.7 Hz, 2H), 7.48 – 7.39 (m, 6H), 7.37 – 7.28 (m, 6H), 7.02 (d, *J* = 7.9 Hz, 2H), 6.95 (d, *J* = 8.1 Hz, 2H), 2.64 (d, *J* = 3.7 Hz, 3H), 1.22 (q, *J* = 5.5, 4.1 Hz, 2H), 0.03 (s, 6H). (*see spectrum*)

^{13}C NMR (101 MHz, Chloroform-*d*) δ 154.10 (dd, *J* = 292.2 Hz, 288.7 Hz), 144.54, 141.04, 139.31, 138.97, 133.51, 132.44 (dd, *J* = 4.7 Hz, 3.1 Hz), 131.57, 130.10 (t, *J* =

3.2 Hz), 128.88, 128.79, 127.95, 127.79, 127.15, 127.05, 126.93, 121.15, 90.71 (dd, *J* = 22.7 Hz, 13.1 Hz), 39.52, 38.24, 23.22, -2.52 (d, *J* = 90.4 Hz). (see spectrum)

^{19}F NMR (376 MHz, Chloroform-d) δ -90.18 (d, *J* = 40.2 Hz), -91.06 (d, *J* = 40.2 Hz). (see spectrum)

HRMS (ESI) (m/z): $[M+H]^+$ calculated for $C_{31}H_{30}BrF_2Si^+$: 547.1263, found: 547.1254.

(2-([1,1'-biphenyl]-4-yl)-5,5-difluoro-4-(4-methoxyphenyl)pent-4-en-1-yl)dimethyl(phenyl)silane (d-53)

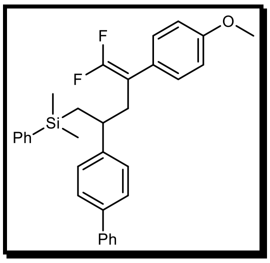

Following the **General procedure C**, **a-1** (56.0 mg, 0.2 mmol), **b-1** (64.9 mg, 0.4 mmol) and **c-53** (60.6 mg, 0.3 mmol) were used. Purification by column chromatography using silica with pentane/EtOAc (100:1 to 20:1, v/v) as eluent afforded **d-53** (38.9 mg, 39% yield) as a colorless oil.

^{1}H NMR (400 MHz, Chloroform-*d*) δ 7.62 – 7.59 (m, 2H), 7.49 – 7.42 (m, 8H), 7.39 – 7.29 (m, 7H), 7.08 – 7.03 (m, 4H), 6.96 – 6.91 (m, 2H), 5.09 (s, 2H), 2.66 (pd, *J* = 6.5, 4.1, 3.7 Hz, 3H), 1.32 – 1.21 (m, 2H), 0.01 (d, *J* = 8.2 Hz, 6H). (see spectrum)

^{13}C NMR (101 MHz, Chloroform-*d*) δ 158.66, 154.09 (dd, *J* = 290.3 Hz, 287.3 Hz), 144.95, 141.14, 139.22, 139.12, 133.54, 129.61 (t, *J* = 3.1 Hz), 128.78, 128.02, 127.73, 127.09, 127.03, 126.86, 113.87, 90.79 (dd, *J* = 21.7 Hz, 13.7 Hz), 55.32, 39.40, 38.60, 22.93, -2.51 (d, *J* = 86.6 Hz). (see spectrum)

^{19}F NMR (376 MHz, Chloroform-d) δ -92.09 (d, *J* = 45.0 Hz), -92.75 (t, *J* = 45.0 Hz) (see spectrum)

HRMS (ESI) (m/z): $[M+H]^+$ calculated for $C_{32}H_{33}F_2OSi^+$: 499.2263, found: 499.2268.

(2-([1,1'-biphenyl]-4-yl)-4-(4-(benzyloxy)phenyl)-5,5-difluoropent-4-en-1-yl)dimethyl(phenyl)silane (d-54)

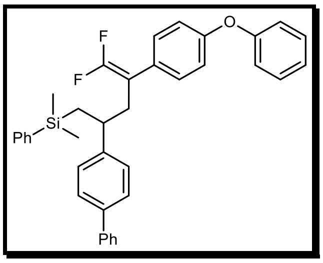

Following the **General procedure C**, **a-1** (56.0 mg, 0.2 mmol), **b-1** (64.9 mg, 0.4 mmol) and **c-54** (79.3.2 mg, 0.3 mmol) were used. Purification by column chromatography using silica with pentane/EtOAc (100:1 to 20:1, v/v) as eluent afforded **d-54** (67.3 mg, 60% yield) as a colorless oil.

^{1}H NMR (400 MHz, Chloroform-*d*) δ 7.58 (d, *J* = 7.4 Hz, 2H), 7.43 (t, *J* = 7.9 Hz, 4H), 7.37 – 7.27 (m, 8H), 7.13 (t, *J* = 7.4 Hz, 1H), 7.08 – 6.99 (m, 6H), 6.93 (d, *J* = 8.7 Hz, 2H), 2.66 (tt, *J* = 9.2, 5.3 Hz, 3H), 1.26 (td, *J* = 11.2, 10.1, 5.4 Hz, 2H), 0.01 (d, *J* = 3.6 Hz, 6H). (*see spectrum*)

^{13}C NMR (101 MHz, Chloroform-*d*) [1] δ 157.00, 156.36, 154.15 (dd, *J* = 291.3 Hz, 287.8 Hz), 144.79, 141.07, 139.17, 139.11, 133.52, 129.85, 129.82 (t, *J* = 2.7 Hz), 128.81, 128.76, 128.00, 127.73, 127.09, 127.01, 126.86, 123.52, 119.13, 118.56, 90.78 (dd, *J* = 21.8 Hz, 13.5 Hz), 39.52, 38.54, 23.05, -2.54(d, *J* = 89.7 Hz). (*see spectrum*)

^{19}F NMR (376 MHz, Chloroform-d) δ -91.97 (d, *J* = 43.7 Hz), -92.11 (d, *J* = 43.3 Hz) (*see spectrum*)

HRMS (ESI) (m/z): $[M+H]^+$ calculated for $C_{37}H_{35}F_2OSi^+$: 561.2420, found: 561.2427.

Methyl 2-acetamido-2-((diphenylmethylene)amino)-3-(pyridin-2-yl)propanoate (d-55)

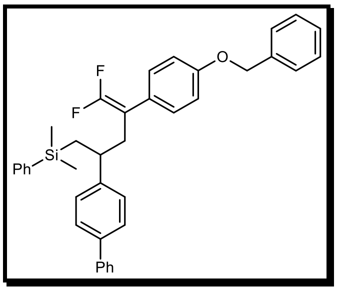

Following the **General procedure C**, **a-1** (56.0 mg, 0.2 mmol), **b-1** (64.9 mg, 0.4 mmol) and **c-55** (83.5 mg, 0.3 mmol) were used. Purification by column chromatography using

silica with pentane/EtOAc (100:1 to 20:1, v/v) as eluent afforded **d-55** (63.2 mg, 55% yield) as a colorless oil.

^{1}H NMR (400 MHz, Chloroform-*d*) δ 7.62 – 7.59 (m, 2H), 7.49 – 7.42 (m, 8H), 7.39 – 7.29 (m, 7H), 7.08 – 7.03 (m, 4H), 6.96 – 6.91 (m, 2H), 5.09 (s, 2H), 2.66 (pd, *J* = 6.5, 4.1, 3.7 Hz, 3H), 1.32 – 1.21 (m, 2H), 0.01 (d, *J* = 8.2 Hz, 6H). (*see spectrum*)

^{13}C NMR (101 MHz, Chloroform-*d*) δ 157.90, 154.57 (dd, *J* = 290.3 Hz, 275.5 Hz), 144.94, 141.14, 139.22, 139.12, 137.00, 133.55, 129.64 (t, *J* = 3.1 Hz), 128.79, 128.73, 128.15, 128.03, 127.74, 127.63, 127.10, 127.04, 126.87, 114.78, 90.79 (dd, *J* = 21.8 Hz, 13.7 Hz), 70.11, 39.42, 38.58, 22.94, -2.49 (d, *J* = 87.7 Hz). (*see spectrum*)

^{19}F NMR (376 MHz, Chloroform-d) δ -91.96 (d, *J* = 44.4 Hz), -91.62 (d, *J* = 44.7 Hz). (*see spectrum*)

HRMS (ESI) (m/z): $[M+H]^+$ calculated for $C_{38}H_{37}F_2OSi^+$: 575.2576, found: 575.2578.

(2-([1,1'-biphenyl]-4-yl)-4-phenylpent-4-en-1-yl)dimethyl(phenyl)silane (d-56)

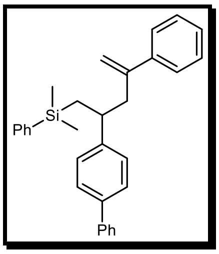

Following the **General procedure C**, **a-1** (56.0 mg, 0.2 mmol), **b-1** (64.9 mg, 0.4 mmol) and **c-56** (77.5 mg, 0.3 mmol) were used. Purification by column chromatography using silica with pentane as eluent afforded **d-56** (36.3 mg, 42% yield) as a colorless oil.

^{1}H NMR (400 MHz, Chloroform-*d*) δ 7.64 – 7.57 (m, 2H), 7.45 (dt, *J* = 7.9, 3.6 Hz, 4H), 7.37 – 7.24 (m, 12H), 7.08 (d, *J* = 8.1 Hz, 2H), 5.06 (dd, *J* = 106.4, 1.6 Hz, 2H), 2.92 – 2.72 (m, 3H), 1.35 (dd, *J* = 13.8, 2.7 Hz, 1H), 1.23 – 1.12 (m, 1H), -0.00 (d, *J* = 6.5 Hz, 6H). (*see spectrum*)

^{13}C NMR (101 MHz, Chloroform-*d*) δ 147.09, 145.85, 141.20, 141.15, 139.42, 138.85, 133.55, 128.77, 128.70, 128.34, 128.05, 127.70, 127.36, 127.03, 126.85, 126.54, 114.70, 47.11, 39.80, 22.74, -2.00, -2.93. (*see spectrum*)

HRMS (ESI) (m/z): $[M+H]^+$ calculated for $C_{31}H_{33}Si^+$: 433.2346, found: 433.2351.

4-([1,1'-biphenyl]-4-yl)-5-(dimethyl(phenyl)silyl)-2-methylenepentanenitrile (d-57)

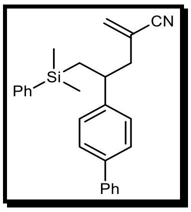

Following the **General procedure C**, **a1** (56.0 mg, 0.2 mmol), **b1** (64.9 mg, 0.4 mmol) and **c-57** (62.2 mg, 0.3 mmol) were used. Purification by column chromatography using silica with pentane/EtOAc (20:1 to 5:1, v/v) as eluent afforded **d-57** (32.8 mg, 43% yield) as a colorless oil.

^{1}H NMR (400 MHz, Chloroform-*d*) δ 7.59 (dt, *J* = 7.4, 2.2 Hz, 2H), 7.49 – 7.29 (m, 10H), 7.16 (dd, *J* = 8.2, 2.7 Hz, 2H), 5.72 (d, *J* = 2.7 Hz, 1H), 5.43 (d, *J* = 2.7 Hz, 1H), 3.05 (ddt, *J* = 12.5, 9.4, 4.5 Hz, 1H), 2.58 – 2.43 (m, 2H), 1.25 (tt, *J* = 7.2, 4.2 Hz, 2H), 0.16 (d, *J* = 2.7 Hz, 3H), 0.05 (d, *J* = 2.7 Hz, 3H). (*see spectrum*)

^{13}C NMR (101 MHz, Chloroform-*d*) δ 143.37, 140.85, 139.55, 138.86, 133.53, 132.15, 128.95, 128.81, 128.02, 127.83, 127.23, 127.14, 127.01, 121.50, 118.67, 45.77, 40.30, 23.41, -2.36, -2.95. (*see spectrum*)

HRMS (ESI) (m/z): $[M+H]^+$ calculated for $C_{26}H_{28}NSi^+$: 382.1986, found: 382.1991.

methyl 4-([1,1'-biphenyl]-4-yl)-5-(dimethyl(phenyl)silyl)-2-methylenepentanoate (d-58)

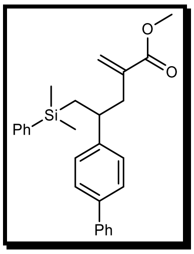

Following the **General procedure C**, **a-1** (56.0 mg, 0.2 mmol), **b-1** (64.9 mg, 0.4 mmol) and **c-58** (72.1 mg, 0.3 mmol) were used. Purification by column chromatography using silica with pentane/EtOAc (50:1 to 10:1, v/v) as eluent afforded **d-58** (42.2 mg, 51% yield) as a colorless oil.

^{1}H NMR (400 MHz, Chloroform-*d*) δ 7.59 (d, *J* = 7.6 Hz, 2H), 7.47 – 7.39 (m, 6H), 7.35 – 7.29 (m, 4H), 7.13 (d, *J* = 8.1 Hz, 2H), 6.03 (d, *J* = 1.6 Hz, 1H), 5.26 (d, *J* = 1.6 Hz, 1H), 3.68 (s, 3H), 2.98 (td, *J* = 9.1, 4.2 Hz, 1H), 2.70 – 2.51 (m, 2H), 1.32 – 1.20 (m, 2H), 0.14 (s, 3H), 0.06 (s, 3H). (*see spectrum*)

^{13}**C NMR** (101 MHz, Chloroform-*d*) δ 167.76, 145.17, 141.10, 139.46, 138.97, 138.68, 133.58, 128.77, 128.18, 127.72, 127.07, 126.99, 126.97, 126.87, 51.80, 43.37, 40.73, 23.43, -2.22, -2.83. (see spectrum)

HRMS (ESI) (m/z): [M+H]$^+$ calculated for $C_{27}H_{31}O_2Si^+$: 415.2088, found: 415.2079.

dimethyl 2-(2-(dimethyl(phenyl)silyl)-1-(4-((((1S,2S,5R)-2-isopropyl-5-methylcyclohexyl)oxy)carbonyl)phenyl)ethyl)succinate (d-59)

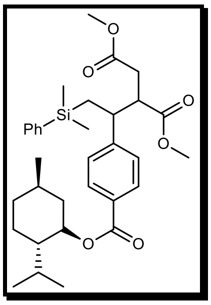

Following the **General procedure A**, **a-59** (77.3.0 mg, 0.2 mmol), **b-1** (64.9 mg, 0.4 mmol) and **c-1** (43.2 mg, 0.3 mmol) were used. Purification by column chromatography using silica with pentane/EtOAc (30:1 to 5:1, v/v) as eluent afforded **d-59** (53.3 mg, 47% yield) as a colorless oil.

^{1}**H NMR** (400 MHz, Chloroform-*d*) δ 7.43 (d, *J* = 8.0 Hz, 2H), 7.37 – 7.27 (m, 5H), 7.11 (dd, *J* = 27.1, 8.0 Hz, 2H), 3.97 (dtd, *J* = 11.1, 7.2, 6.5, 3.0 Hz, 1H), 3.64 (d, *J* = 42.9 Hz, 3H), 3.51 (d, *J* = 20.0 Hz, 4H), 3.15 – 2.88 (m, 2H), 2.56 (ddd, *J* = 88.2, 16.7, 10.7 Hz, 1H), 2.37 – 1.69 (m, 10H), 1.63 – 1.20 (m, 8H), 1.19 – 0.90 (m, 7H), 0.05 – -0.13 (m, 6H). (see spectrum)

major: ^{13}**C NMR** (101 MHz, Chloroform-*d*) δ 174.46, 172.14, 171.74, 154.29, 145.49, 135.77, 135.77, 133.44, 129.12, 128.51, 127.90, 127.07, 58.50, 51.93, 51.78, 50.39, 49.70, 43.93, 34.59, 32.71, 32.52, 30.76, 26.32, 25.43, 25.26, 24.61, 21.03, -1.83, -3.20.

minor: ^{13}**C NMR** (101 MHz, Chloroform-*d*) δ 173.72, 172.44, 171.95, 154.29, 145.33, 138.24, 135.88, 133.44, 129.19, 128.65, 127.90, 126.73, 58.44, 51.86, 51.75, 49.85, 49.70, 43.27, 34.59, 32.71, 32.56, 30.88, 26.34, 25.45, 25.26, 24.61, 17.87 -1.95, -3.13. (see spectrum)

HRMS (ESI) (m/z): [M+H]$^+$ calculated for $C_{33}H_{47}O_6Si^+$: 567.3136, found: 567.3140.

dimethyl 2-(2-(dimethyl(phenyl)silyl)-1-(4-((((3aS,5S,5aR,8aR,8bS)-2,2,7,7-

tetramethyltetrahydro-5H-bis([1,3]dioxolo)[4,5-b:4',5'-d]pyran-5-yl)methoxy)carbonyl)phenyl)ethyl)succinate (d-60)

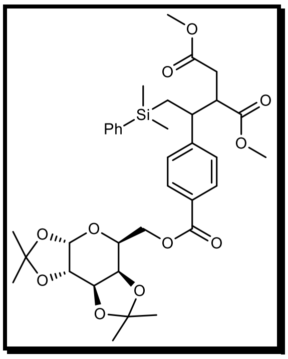

Following the **General procedure A**, **a-60** (98.1 mg, 0.2 mmol), **b-1** (64.9 mg, 0.4 mmol) and **c-1** (43.2 mg, 0.3 mmol) were used. Purification by column chromatography using silica with pentane/EtOAc (30:1 to 5:1, v/v) as eluent afforded **d-60** (69.8 mg, 52% yield) as a colorless oil.

^{1}H NMR (400 MHz, Chloroform-*d*) δ 7.93 (dt, *J* = 8.4, 2.3 Hz, 2H), 7.29 (dd, *J* = 7.9, 5.0 Hz, 5H), 7.19 – 7.04 (m, 2H), 4.72 – 4.61 (m, 2H), 4.46 (dt, *J* = 4.2, 2.5 Hz, 1H), 4.34 – 4.24 (m, 2H), 4.00 – 3.77 (m, 2H), 3.66 (d, *J* = 39.1 Hz, 3H), 3.46 (d, *J* = 53.8 Hz, 3H), 3.00 (dt, *J* = 16.2, 10.8 Hz, 2H), 2.74 – 2.04 (m, 2H), 1.55 (s, 3H), 1.47 (s, 3H), 1.41 – 1.21 (m, 8H), 1.15 (ddd, *J* = 14.4, 11.1, 2.7 Hz, 1H), -0.00 – -0.13 (m, 6H). (see spectrum)

major: ^{13}C NMR (101 MHz, Chloroform-*d*) δ 173.90, 172.14, 165.69, 165.70, 147.95, 138.21, 133.45, 130.10, 129.06, 128.86, 128.42, 127.83, 109.25, 108.91, 101.73, 70.86, 70.61, 65.38, 61.41, 51.95, 51.80, 50.04, 44.18, 34.75, 33.32, 26.60, 25.97, 25.60, 24.09, -2.15, -3.23. **minor**: **^{13}C NMR** (101 MHz, Chloroform-*d*) δ 174.51 172.45, 165.77, 165.70, 147.93, 138.17, 133.45, 129.79, 129.15, 128.67, 128.54, 127.86, 109.25, 108.91, 101.73, 70.58, 70.16, 65.27, 61.41 51.91, 51.66, 50.39, 43.81, 34.75, 33.32, 26.60, 25.97, 25.57, 24.09, 18.49, -2.21, -3.26. (see spectrum)

HRMS (ESI) (m/z): $[M+H]^+$ calculated for $C_{35}H_{47}O_{11}Si^+$: 671.2882, found: 671.2888.

dimethyl 2-(2-(dimethyl(phenyl)silyl)-1-(4-((((3aS,8aR,8bS)-2,2,7,7-tetramethyltetrahydro-3aH-bis([1,3]dioxolo)[4,5-b:4',5'-d]pyran-3a-yl)methoxy)carbonyl)phenyl)ethyl)succinate (d-61)

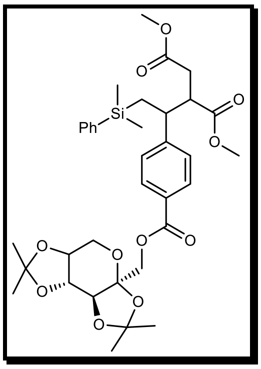

Following the **General procedure A**, **a-61** (98.1 mg, 0.2 mmol), **b-1** (64.9 mg, 0.4 mmol) and **c-1** (43.2 mg, 0.3 mmol) were used. Purification by column chromatography using silica with pentane/EtOAc (30:1 to 5:1, v/v) as eluent afforded **d-61** (72.2 mg, 54% yield) as a colorless oil.

^{1}H NMR (400 MHz, Chloroform-*d*) δ 7.91 – 7.87 (m, 2H), 7.36 – 7.22 (m, 6H), 7.10 (dd, *J* = 23.9, 8.1 Hz, 2H), 5.57 (d, *J* = 4.9 Hz, 1H), 4.66 (dd, *J* = 7.9, 2.5 Hz, 1H), 4.55 – 4.47 (m, 1H), 4.41 (dd, *J* = 11.6, 7.5 Hz, 1H), 4.34 (td, *J* = 7.8, 2.2 Hz, 2H), 4.18 (ddd, *J* = 7.1, 4.8, 1.8 Hz, 1H), 3.65 (d, *J* = 43.1 Hz, 3H), 3.48 (d, *J* = 40.5 Hz, 3H), 3.13 – 2.92 (m, 2H), 2.74 – 2.04 (m, 2H), 1.52 (d, *J* = 1.8 Hz, 3H), 1.48 (s, 3H), 1.38 – 1.23 (m, 8H), 1.14 (ddd, *J* = 14.3, 7.4, 2.7 Hz, 1H), -0.07 (s, 6H). (see spectrum)

major: ^{13}C NMR (101 MHz, Chloroform-*d*) δ 174.56, 172.17, 166.27, 147.75, 138.26, 133.44, 130.06, 130.03, 129.00, 128.81, 128.37, 127.84, 109.77, 108.88, 96.40, 71.22, 70.79, 70.59, 66.23, 63.98, 51.88, 51.78, 50.01, 44.19, 34.76, 26.11, 26.04, 25.05, 24.57, 21.11, -2.27 -3.15. **minor**: **^{13}C NMR** (101 MHz, Chloroform-*d*) δ 173.88, 172.46, 166.18, 147.70, 138.22, 133.44, 130.06, 129.73, 129.10, 128.81, 128.47, 127.80, 109.77, 108.88, 96.40, 71.22, 70.79, 70.59, 66.23, 63.98, 51.94, 51.70, 50.44, 43.71, 33.15, 26.11, 26.04, 25.05, 24.57, 18.31, -2.27 -3.15. (see spectrum)

HRMS (ESI) (m/z): [M+H]$^+$ calculated for $C_{35}H_{47}O_{11}Si^+$: 671.2882, found: 671.2886.

dimethyl 2-(2-(dimethyl(phenyl)silyl)-1-(4-((((3aS,4R,6R,6aS)-6-((R)-2,2-dimethyl-1,3-dioxolan-4-yl)-2,2-dimethyltetrahydrofuro[3,4-d][1,3]dioxol-4-yl)oxy)carbonyl)phenyl)ethyl)succinate (d-62)

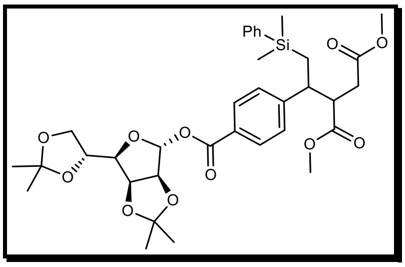

Following the **General procedure A**, **a-62** (98.0 mg, 0.2 mmol), **b-1** (64.9 mg, 0.4 mmol) and **c-1** (43.2 mg, 0.3 mmol) were used. Purification by column chromatography using silica with pentane/EtOAc (30:1 to 5:1, v/v) as eluent afforded **d-62** (93.9 mg, 70% yield) as a colorless oil.

^{1}H NMR (400 MHz, Chloroform-*d*) δ 7.84 (dd, *J* = 8.0, 4.5 Hz, 2H), 7.33 – 7.25 (m, 5H), 7.12 (dd, *J* = 23.4, 8.0 Hz, 2H), 6.35 (s, 1H), 4.94 (dd, *J* = 6.0, 3.6 Hz, 1H), 4.86 (d, *J* = 5.8 Hz, 1H), 4.48 – 4.41 (m, 1H), 4.10 (dtd, *J* = 13.2, 8.6, 4.1 Hz, 3H), 3.65 (d, *J* = 43.6 Hz, 3H), 3.49 (d, *J* = 35.5 Hz, 3H), 3.12 – 2.93 (m, 2H), 2.73 – 2.04 (m, 2H), 1.47 (s, 3H), 1.38 (d, *J* = 3.6 Hz, 6H), 1.32 – 1.25 (m, 1H), 1.20 – 1.11 (m, 1H), -0.02 (dd, *J* = 11.1, 5.9 Hz, 6H). (*see spectrum*)

major: ^{13}C NMR (101 MHz, Chloroform-*d*) δ 174.45, 172.10, 164.66, 148.37, 138.19, 133.44, 130.13, 128.99, 128.48, 128.40, 127.80, 113.43, 109.43, 101.58, 85.29, 82.63, 79.44, 72.98, 66.91, 51.95, 51.78, 50.40, 44.17, 34.69, 27.05, 26.03, 25.16, 24.74, 21.04, -2.23, -3.07. **minor**: **^{13}C NMR** (101 MHz, Chloroform-*d*) δ 173.77, 172.38, 164.76, 148.33, 138.15, 133.44, 129.82, 129.10, 128.60, 128.23, 127.84, 113.43, 109.43, 101.58, 85.29, 82.63, 79.44, 72.98, 66.91, 51.88, 51.73, 49.99, 43.75, 33.24, 27.05, 26.03, 25.16, 24.74, 18.42, -2.27, -3.07. (*see spectrum*)

HRMS (ESI) (m/z): $[M+H]^+$ calculated for $C_{35}H_{47}O_{11}Si^+$: 671.2882, found: 671.2879.

dimethyl 2-(2-(dimethyl(phenyl)silyl)-1-(4-((((5R,5aR,8aR,9R)-8-oxo-9-(3,4,5-trimethoxyphenyl)-5,5a,6,8,8a,9-hexahydrofuro[3',4':6,7]naphtho[2,3-d][1,3]dioxol-5-yl)oxy)carbonyl)phenyl)ethyl)succinate (d-63)

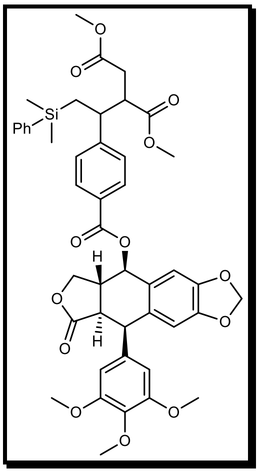

Following the **General procedure A**, **a d-63** (128.9 mg, 0.2 mmol), **b-1** (64.9 mg, 0.4 mmol) and **c-1** (43.2 mg, 0.3 mmol) were used. Purification by column chromatography using silica with pentane/EtOAc (20:1 to 5:1, v/v) as eluent afforded **d-63** as eluent afforded **52** (87.4 mg, 53% yield) as a colorless oil.

^{1}H NMR (400 MHz, Chloroform-*d*) δ 7.87 (dt, *J* = 8.7, 4.5 Hz, 2H), 7.35 – 7.23 (m, 5H), 7.15 (ddd, *J* = 23.0, 8.3, 4.2 Hz, 2H), 6.87 (d, *J* = 4.4 Hz, 1H), 6.59 (s, 1H), 6.45 (s, 2H), 6.10 (d, *J* = 7.9 Hz, 1H), 6.00 (d, *J* = 9.4 Hz, 2H), 4.65 (d, *J* = 3.7 Hz, 1H), 4.49 – 4.42 (m, 1H), 4.32 (t, *J* = 9.4 Hz, 1H), 3.82 – 3.77 (m, 9H), 3.73 – 3.61 (m, 3H), 3.56 – 3.46 (m, 3H), 3.15 – 2.94 (m, 4H), 2.75 – 2.04 (m, 2H), 1.30 (t, *J* = 12.9 Hz, 1H), 1.22 – 1.14 (m, 1H), 0.03 (d, *J* = 4.0 Hz, 6H). (*see spectrum*)

major: ^{13}C NMR (101 MHz, Chloroform-*d*) δ 174.36, 173.74, 172.06, 166.53, 152.73, 148.65, 148.30, 147.79, 138.17, 137.25, 134.93, 133.44, 132.58, 129.65, 128.98, 128.82, 128.46, 127.97, 127.78, 109.87, 108.19, 107.20, 101.71, 74.22, 71.59, 60.81, 56.19, 51.99, 51.82, 50.38, 45.71, 44.13, 43.84, 38.93, 34.61, 21.00, -2.31, -2.92. **minor**: **^{13}C NMR** (101 MHz, Chloroform-*d*) δ 173.74, 173.68, 172.34, 166.63, 152.73, 148.61, 148.30, 147.79, 138.13, 137.25, 134.93, 133.44, 132.58, 129.94, 129.09, 128.68, 128.46, 128.12, 127.98, 127.96, 127.83, 109.87, 108.19, 107.20, 101.71, 74.22, 71.59, 60.81, 56.19, 51.91, 51.77, 49.95, 45.71, 43.84, 43.70, 38.93, 33.22, 18.40, -2.39, -2.97. (*see spectrum*)

HRMS (ESI) (m/z): $[M+H]^+$ calculated for $C_{45}H_{49}O_{13}Si^+$: 825.2937, found: 825.2937.

dimethyl 2-(2-(dimethyl(phenyl)silyl)-1-(4-(((1,7,7-trimethylbicyclo[2.2.1]heptan-

2-yl)oxy)carbonyl)phenyl)ethyl)succinate (d-64)

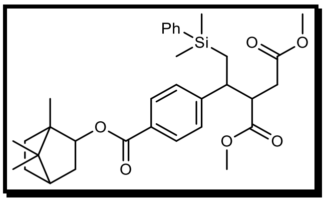

Following the **General procedure A**, **a-64** (76.5 mg, 0.2 mmol), **b-1** (64.9 mg, 0.4 mmol) and **c-1** (43.2 mg, 0.3 mmol) were used. Purification by column chromatography using silica with pentane/EtOAc (20:1 to 5:1, v/v) as eluent afforded **d-64** (65.5 mg, 58% yield) as a colorless oil.

^{1}H NMR (400 MHz, Chloroform-*d*) δ 7.88 (t, *J* = 7.3 Hz, 2H), 7.29 (dt, *J* = 14.1, 4.6 Hz, 5H), 7.11 (dd, *J* = 24.5, 8.3 Hz, 2H), 5.11 (dd, *J* = 9.7, 3.0 Hz, 1H), 3.66 (d, *J* = 46.7 Hz, 3H), 3.50 (d, *J* = 28.7 Hz, 3H), 3.15 – 2.95 (m, 2H), 2.74 – 2.06 (m, 4H), 1.93 – 1.38 (m, 4H), 1.31 (dt, *J* = 15.1, 11.5 Hz, 2H), 1.14 (ddd, *J* = 14.0, 8.5, 3.1 Hz, 2H), 0.95 (d, *J* = 19.6 Hz, 9H), 0.01 (s, 6H). (*see spectrum*)

major: ^{13}C NMR (101 MHz, Chloroform-*d*) δ 174.57, 172.18, 166.57, 147.33, 138.29, 133.44, 129.80, 129.65, 128.93, 128.32, 127.77, 80.55, 51.94, 51.77, 50.01, 49.15, 47.95, 45.06, 44.16, 36.98, 34.74, 28.17, 27.46, 21.15, 19.80, 18.99, 13.70, -2.38, -3.02.

minor: **^{13}C NMR** (101 MHz, Chloroform-*d*) δ173.83, 172.46, 166.67, 147.38, 138.33, 133.44, 129.80, 129.65, 129.04, 128.44, 127.82, 80.55, 51.86, 51.72, 50.48, 49.15, 47.95, 45.06, 43.65, 36.98, 33.14, 28.17, 27.46, 19.80, 18.99, 18.33, 13.70, -2.36, -3.02. (*see spectrum*)

HRMS (ESI) (m/z): $[M+H]^+$ calculated for $C_{33}H_{45}O_6Si^+$: 565.2980, found: 565.2971.

dimethyl 2-(2-(dimethyl(phenyl)silyl)-1-(4-(((1,3,3-trimethylbicyclo[2.2.1]heptan-2-yl)oxy)carbonyl)phenyl)ethyl)succinate (d-65)

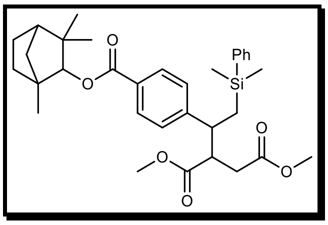

Following the **General procedure A**, **a-65** (76.9 mg, 0.2 mmol), **b-1** (64.9 mg, 0.4 mmol) and **c-1** (43.2 mg, 0.3 mmol) were used. Purification by column chromatography

using silica with pentane/EtOAc (20:1 to 5:1, v/v) as eluent afforded **d-65** (63.3 mg, 56% yield) as a colorless oil.

^{1}H NMR (400 MHz, Chloroform-*d*) δ 7.92 – 7.83 (m, 2H), 7.34 – 7.21 (m, 5H), 7.11 (dd, *J* = 24.8, 8.1 Hz, 2H), 4.60 (d, *J* = 1.9 Hz, 1H), 3.66 (d, *J* = 48.1 Hz, 3H), 3.50 (d, *J* = 28.1 Hz, 3H), 3.15 – 2.95 (m, 2H), 2.74 – 2.07 (m, 2H), 1.92 (ddd, *J* = 12.9, 10.1, 6.5 Hz, 1H), 1.83 – 1.76 (m, 2H), 1.69 – 1.65 (m, 1H), 1.57 – 1.48 (m, 1H), 1.27 (td, *J* = 10.8, 9.2, 3.7 Hz, 2H), 1.21 – 1.11 (m, 8H), 0.85 (d, *J* = 2.8 Hz, 3H), 0.04 – -0.03 (m, 6H). (*see spectrum*)

major: ^{13}C NMR (101 MHz, Chloroform-*d*) δ 174.60, 172.19, 166.66, 147.35, 138.30, 133.43, 129.77, 129.43, 128.90, 128.36, 127.74, 86.72, 51.95, 51.77, 50.49, 48.69, 48.48, 44.18, 41.53, 39.90, 34.79, 29.82, 26.95, 25.99, 21.20, 20.40, 19.59, -2.55, -2.95, -2.99. **minor**: **^{13}C NMR** (101 MHz, Chloroform-*d*) δ 173.86, 172.48, 166.76, 138.30, 138.27, 133.40, 129.59, 129.48, 129.02, 128.48, 127.79, 86.68, 51.86, 51.71, 49.98, 48.69, 48.48, 43.62, 41.53, 39.90, 33.05, 29.82, 26.95, 25.99, 20.40, 19.59, 18.24, -2.48, -2.95. (*see spectrum*)

HRMS (ESI) (m/z): $[M+H]^+$ calculated for $C_{33}H_{45}O_6Si^+$: 565.2980, found: 565.2972.

dimethyl 2-(2-(dimethyl(phenyl)silyl)-1-(4-(((3,7-dimethyloct-6-en-1-yl)oxy)carbonyl)phenyl)ethyl)succinate (d-66)

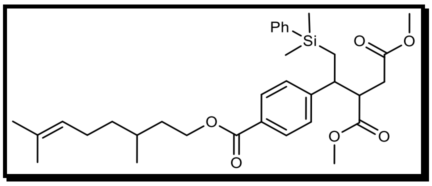

Following the **General procedure A**, **a-66** (77.3 mg, 0.2 mmol), **b-1** (64.9 mg, 0.4 mmol) and **c-1** (43.2 mg, 0.3 mmol) were used. Purification by column chromatography using silica with pentane/EtOAc (30:1 to 5:1, v/v) as eluent afforded **d-66** (70.3 mg, 62% yield) as a colorless oil.

^{1}H NMR (400 MHz, Chloroform-*d*) δ 7.88 (dd, *J* = 8.1, 4.5 Hz, 2H), 7.38 – 7.26 (m, 5H), 7.11 (dd, *J* = 23.6, 8.2 Hz, 2H), 5.15 – 5.06 (m, 1H), 4.35 (td, *J* = 6.7, 3.8 Hz, 2H), 3.66 (d, *J* = 44.4 Hz, 3H), 3.48 (d, *J* = 38.6 Hz, 3H), 3.12 – 2.92 (m, 2H), 2.74 – 1.94 (m, 4H), 1.87 – 1.11 (m, 15H), 0.98 (d, *J* = 6.5 Hz, 3H), -0.07 (s, 6H). (*see spectrum*)

major: ^{13}**C NMR** (101 MHz, Chloroform-*d*) δ 174.56, 172.16, 166.42, 147.51, 138.28, 133.45, 131.44, 129.85, 129.47, 129.08, 128.33, 127.79, 124.63, 63.54, 51.93, 51.76, 50.44, 44.17, 37.05, 35.56, 34.75, 33.17, 29.60, 25.46, 21.11, 19.58, 17.73, -2.26, -3.16.

minor: ^{13}**C NMR** (101 MHz, Chloroform-*d*) δ173.87, 172.46, 166.52, 147.46, 138.25, 133.45, 131.44, 129.54, 129.29, 128.98, 128.45, 127.83, 124.63, 63.54, 51.86, 51.68, 50.02, 43.70, 37.05, 35.56, 34.75, 33.17, 29.60, 25.79, 19.58, 18.35, 17.73, -2.26, -3.16.

(*see spectrum*)

HRMS (ESI) (m/z): $[M+H]^+$ calculated for $C_{33}H_{47}O_6Si^+$: 567.3136, found: 567.3133.

dimethyl 2-(2-(dimethyl(phenyl)silyl)-1-(4-((((R)-2,5,7,8-tetramethyl-2-((4R,8R)-4,8,12-trimethyltridecyl)chroman-6-yl)oxy)carbonyl)phenyl)ethyl)succinate (d-67)

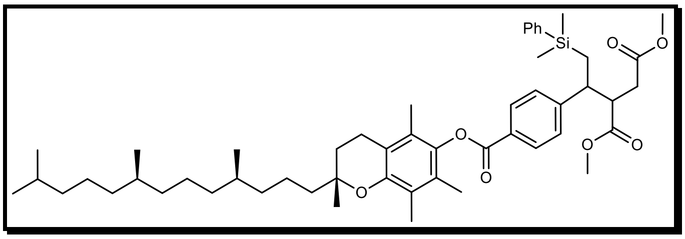

Following the **General procedure A**, **a-67** (132.1 mg, 0.2 mmol), **b-1** (64.9 mg, 0.4 mmol) and **c-1** (43.2 mg, 0.3 mmol) were used. Purification by column chromatography using silica with pentane/EtOAc (20:1 to 3:1, v/v) as eluent afforded **d-67** (94.2 mg, 56% yield) as a colorless oil.

^{1}**H NMR** (400 MHz, Chloroform-*d*) δ 8.06 (dd, *J* = 9.8, 8.1 Hz, 2H), 7.36 – 7.26 (m, 5H), 7.19 (dd, *J* = 25.9, 8.1 Hz, 2H), 3.70 (d, *J* = 49.3 Hz, 3H), 3.54 (d, *J* = 23.8 Hz, 3H), 3.25 – 2.98 (m, 2H), 2.79 – 2.50 (m, 3H), 2.49 – 2.13 (m, 4H), 2.08 (s, 3H), 2.04 (s, 3H), 1.83 (dh, *J* = 19.8, 6.7 Hz, 2H), 1.63 – 1.38 (m, 7H), 1.36 – 1.25 (m, 11H), 1.23 – 1.06 (m, 8H), 0.88 (t, *J* = 6.6 Hz, 12H), 0.07 (dd, *J* = 9.8, 5.1 Hz, 6H). (*see spectrum*)

major: ^{13}**C NMR** (101 MHz, Chloroform-*d*) δ 174.60, 172.21, 164.97, 149.56, 148.06, 140.66, 138.25, 133.44, 130.40 128.97, 128.58, 128.51, 127.78, 126.97, 125.20, 123.22, 117.57, 75.17, 51.92, 51.84, 50.48, 44.26, 39.46, 37.54, 37.48, 37.37, 34.79, 32.87, 31.29, 31.08, 28.07, 24.91, 24.54, 24.31, 23.80, 22.83, 22.73, 21.21, 21.14, 20.73, 19.86, 19.79, 19.74, 13.18, 12.33, 11.96, -2.59, -2.84. **minor**: ^{13}**C NMR** (101 MHz, Chloroform-*d*) δ173.85, 172.52, 165.07, 149.56, 148.04, 140.66, 138.21, 133.47,

130.13, 129.09, 128.71, 128.34, 127.83, 126.97, 125.20, 123.22, 117.57, 75.17, 52.02, 51.79, 49.96, 43.68, 39.46, 37.54, 37.48, 37.37, 33.04, 32.87, 28.07, 24.91, 24.54, 24.31, 23.80, 22.83, 22.73, 21.14, 20.73, 19.86, 19.79, 19.74, 18.23, 13.18, 12.33, 11.96, -2.49, -2.91. (<u>see spectrum</u>)

HRMS (ESI) (m/z): $[M+H]^+$ calculated for $C_{52}H_{77}O_7Si^+$: 841.5433, found: 841.5425.

dimethyl 2-(2-(dimethyl(phenyl)silyl)-1-(4-((((7R,11R,E)-3,7,11,15-tetramethylhexadec-2-en-1-yl)oxy)carbonyl)phenyl)ethyl)succinate (d-68)

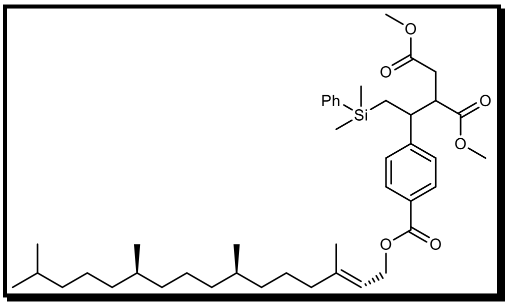

Following the **General procedure A**, **a-68** (105.3 mg, 0.2 mmol), **b-1** (64.9 mg, 0.4 mmol) and **c-1** (43.2 mg, 0.3 mmol) were used. Purification by column chromatography using silica with pentane/EtOAc (30:1 to 5:1, v/v) as eluent afforded **d-68** (80.6 mg, 57% yield) as a colorless oil.

^{1}H NMR (400 MHz, Chloroform-*d*) δ 7.90 (dd, *J* = 8.2, 4.4 Hz, 2H), 7.36 – 7.26 (m, 5H), 7.11 (dd, *J* = 24.4, 8.3 Hz, 2H), 5.50 – 5.42 (m, 1H), 4.83 (d, *J* = 7.1 Hz, 2H), 3.66 (d, *J* = 43.5 Hz, 3H), 3.53 (s, 3H), 3.12 – 2.92 (m, 2H), 2.75 – 2.01 (m, 4H), 1.76 (d, *J* = 1.3 Hz, 3H), 1.57 – 1.25 (m, 13H), 1.20 – 1.00 (m, 8H), 0.88 – 0.83 (m, 12H), 0.13 – -0.20 (m, 6H). (<u>see spectrum</u>)

major: ^{13}C NMR (101 MHz, Chloroform-*d*) δ 174.59, 172.19, 166.55, 147.49, 142.99, 138.29, 133.46, 129.94, 129.501, 128.99, 128.31, 127.80, 118.13, 62.00, 51.95, 51.78, 50.45, 44.19, 39.98, 39.43, 37.49, 37.41, 37.35, 36.74, 33.17, 32.86, 32.74, 28.04, 25.13, 24.87, 24.53, 22.80, 22.70, 21.11, 19.83, 19.80, 16.56, -2.20, -3.18. **minor**: **^{13}C NMR** (101 MHz, Chloroform-*d*) δ173.90, 172.49, 166.55, 147.45, 142.92, 138.26, 133.46, 129.63, 129.31, 129.10, 128.42, 127.85, 118.16, 62.00, 51.89, 51.70, 50.04, 43.71, 39.98, 39.43, 37.49, 37.41, 37.35, 36.74, 34.76, 33.17, 32.86, 32.74, 28.04, 25.13, 24.87, 24.53, 22.80, 22.70, 19.83, 19.80, 18.34, 16.56, -2.22, -3.19. (<u>see spectrum</u>)

HRMS (ESI) (m/z): $[M+H]^+$ calculated for $C_{43}H_{67}O_6Si^+$: 707.4701, found: 707.4705.

dimethyl (*E*)-2-(1-(4-((4-(3,5-dimethoxystyryl)phenoxy)carbonyl)phenyl)-2-(dimethyl(phenyl)silyl)ethyl)succinate (d-69)

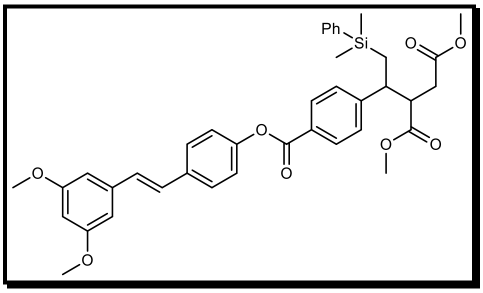

Following the **General procedure A**, **a-69** (97.3 mg, 0.2 mmol), **b-1** (64.9 mg, 0.4 mmol) and **c-1** (43.2 mg, 0.3 mmol) were used. Purification by column chromatography using silica with pentane/EtOAc (20:1 to 5:1, v/v) as eluent afforded **d-69** (69.4 mg, 52% yield) as a white solid.

^{1}H NMR (400 MHz, Chloroform-*d*) δ 8.05 (ddt, *J* = 6.7, 4.9, 1.8 Hz, 2H), 7.57 (d, *J* = 7.7 Hz, 2H), 7.38 – 7.29 (m, 5H), 7.26 – 7.20 (m, 3H), 7.17 (d, *J* = 7.7 Hz, 1H), 7.07 (q, *J* = 16.2 Hz, 2H), 6.69 (t, *J* = 2.0 Hz, 2H), 6.42 (q, *J* = 1.9 Hz, 1H), 3.84 (s, 6H), 3.68 (d, *J* = 43.5 Hz, 3H), 3.52 (d, *J* = 34.2 Hz, 3H), 3.19 – 2.99 (m, 2H), 2.79 – 2.09 (m, 2H), 1.37 – 1.29 (m, 1H), 1.20 (t, *J* = 12.2 Hz, 1H), 0.08 – -0.03 (m, 6H). (*see spectrum*)

major: ^{13}C NMR (101 MHz, Chloroform-*d*) δ 174.49, 172.14, 164.88, 161.08, 150.47, 148.52, 139.27, 138.21, 135.10, 130.50, 129.04 (2), 128.62, 128.43, 128.28, 127.88, 127.64, 122.01, 104.68, 100.17, 55.46, 51.99, 51.83, 50.41, 44.23, 34.71, 21.09, -2.25, -3.05. **minor**: **^{13}C NMR** (101 MHz, Chloroform-*d*) δ 173.82, 172.43, 164.97, 161.08, 150.49, 148.52, 139.27, 138.18, 133.48, 130.21, 129.14, 129.04, 128.74, 128.28 (2), 127.84, 127.64, 122.01, 104.68, 100.17, 55.46, 51.92, 51.76, 50.04, 43.83, 33.30, 18.49, -2.25, -3.05. (*see spectrum*)

HRMS (ESI) (m/z): $[M+H]^+$ calculated for $C_{39}H_{43}O_8Si^+$: 667.2722, found: 667.2727.

dimethyl 2-(2-(dimethyl(phenyl)silyl)-1-(4-((((3*S*,8*S*,9*S*,10*R*,13*R*,14*S*,17*R*)-10,13-dimethyl-17-((*R*)-6-methylheptan-2-yl)-2,3,4,7,8,9,10,11,12,13,14,15,16,17-tetradecahydro-1H-cyclopenta[a]phenanthren-3-yl)oxy)carbonyl)phenyl)ethyl)succinate (d-70)

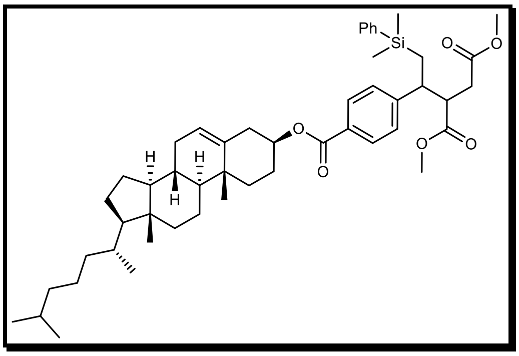

Following the **General procedure A**, **a-70** (123.3 mg, 0.2 mmol), **b-1** (64.9 mg, 0.4 mmol) and **c-1** (43.2 mg, 0.3 mmol) were used. Purification by column chromatography using silica with pentane/EtOAc (20:1 to 5:1, v/v) as eluent afforded **d-70** (82.9 mg, 52% yield) as a white solid.

^{1}H NMR (400 MHz, Chloroform-*d*) δ 7.89 (dd, *J* = 8.1, 4.9 Hz, 2H), 7.35 – 7.26 (m, 5H), 7.11 (dd, *J* = 24.3, 8.1 Hz, 2H), 5.47 – 5.38 (m, 1H), 4.85 (dtd, *J* = 11.9, 8.4, 4.5 Hz, 1H), 3.66 (d, *J* = 44.6 Hz, 3H), 3.49 (d, *J* = 36.2 Hz, 3H), 3.13 – 2.91 (m, 2H), 2.75 – 1.67 (m, 11H), 1.64 – 1.45 (m, 6H), 1.36 (dt, *J* = 11.8, 7.6 Hz, 3H), 1.29 – 1.21 (m, 3H), 1.20 – 1.09 (m, 6H), 1.09 – 0.96 (m, 7H), 0.93 (d, *J* = 6.5 Hz, 3H), 0.87 (dd, *J* = 6.6, 1.8 Hz, 6H), 0.70 (s, 3H), 0.07 – -0.14 (m, 7H). (see spectrum)

major: ^{13}C NMR (101 MHz, Chloroform-*d*) δ 174.58, 172.18, 165.74, 147.41, 139.71, 138.31, 133.45, 129.87, 129.62, 129.08, 128.27, 127.80, 122.88, 122.86, 74.64, 56.76, 56.20, 51.87, 51.77, 50.46, 50.11, 44.17, 42.39, 39.81, 39.58, 38.30, 37.11, 36.72, 36.25, 35.87, 34.75, 32.01, 31.95, 28.31, 28.08, 27.96, 24.37, 23.90, 22.90, 22.64, 21.12, 19.46 (2), 18.80, 18.34, 11.94, -2.23, -3.14. **minor**: **^{13}C NMR** (101 MHz, Chloroform-*d*) δ 173.87, 172.47, 165.85, 147.36, 139.73, 138.28, 133.45, 129.80, 129.47, 128.97, 128.39, 127.84, 122.88, 122.86, 74.64, 56.76, 56.20, 51.93, 51.70, 50.11, 50.03, 43.69, 42.39, 39.81, 39.58, 38.30, 37.11, 36.72, 36.25, 35.87, 33.16, 32.01, 31.95, 28.31, 28.08, 27.96, 24.37, 23.90, 22.90, 22.64, 21.12, 19.46, 18.80, 18.34, 11.94, -2.23, -3.14. (see spectrum)

HRMS (ESI) (m/z): $[M+H]^+$ calculated for $C_{50}H_{73}O_6Si^+$: 797.5171, found: 797.5165.

dimethyl 2-(2-(dimethyl(phenyl)silyl)-1-(4-((((10*S*,13*R*,14*R*,17*R*)-4,4,10,13,14-pentamethyl-17-((*R*)-6-methylhept-5-en-2-yl)-2,3,4,5,6,7,10,11,12,13,14,15,16,17-

tetradecahydro-1H-cyclopenta[a]phenanthren-3-yl)oxy)carbonyl)phenyl)ethyl)succinate (d-71)

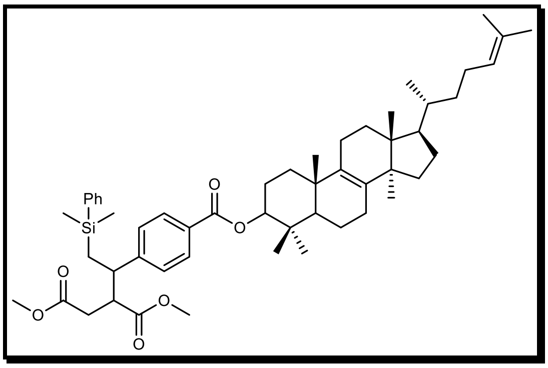

Following the **General procedure A**, **a-71** (131.3 mg, 0.2 mmol), **b-1** (64.9 mg, 0.4 mmol) and **c-1** (43.2 mg, 0.3 mmol) were used. Purification by column chromatography using silica with pentane/EtOAc (20:1 to 5:1, v/v) as eluent afforded **d-71** (92.1 mg, 55% yield) as a white solid.

^{1}H NMR (400 MHz, Chloroform-*d*) δ 7.87 (t, *J* = 7.4 Hz, 2H), 7.34 – 7.23 (m, 5H), 7.11 (dd, *J* = 24.6, 7.9 Hz, 2H), 5.16 – 5.08 (m, 1H), 4.74 (dd, *J* = 11.6, 4.4 Hz, 1H), 3.67 (d, *J* = 46.0 Hz, 3H), 3.50 (d, *J* = 30.6 Hz, 3H), 3.17 – 2.94 (m, 2H), 2.76 – 2.46 (m, 1H), 2.46 – 2.01 (m, 6H), 1.98 – 1.91 (m, 1H), 1.89 – 1.82 (m, 2H), 1.79 – 1.72 (m, 3H), 1.71 (s, 3H), 1.62 (s, 3H), 1.56 (d, *J* = 12.0 Hz, 1H), 1.53 – 1.47 (m, 1H), 1.38 (q, *J* = 14.2, 13.6 Hz, 4H), 1.26 (t, *J* = 14.4 Hz, 3H), 1.20 – 1.12 (m, 3H), 1.06 (d, *J* = 6.3 Hz, 6H), 0.98 (s, 3H), 0.94 – 0.87 (m, 9H), 0.72 (s, 3H), 0.08 – -0.07 (m, 6H). (*see spectrum*)

major: ^{13}C NMR (101 MHz, Chloroform-*d*) δ 174.60, 172.48, 166.05, 147.31, 138.32, 134.63, 134.31, 133.43, 130.98, 129.94, 129.81, 128.93, 128.31, 127.76, 125.33, 81.62, 51.94, 51.77, 50.66, 50.57, 50.50, 50.47, 50.46, 49.90, 44.56, 44.16, 39.59, 38.29, 37.02, 36.55, 36.42, 36.33, 35.35, 34.76, 31.05, 30.91, 28.28, 28.17, 28.07, 26.46, 25.79, 24.99, 24.34, 24.17, 22.90, 22.61, 21.16, 21.11, 19.28, 18.79, 18.71, 18.23, 17.70, 16.90, 15.84, -2.35, -3.02. **minor**: **^{13}C NMR** (101 MHz, Chloroform-*d*) δ 173.85, 172.19, 166.16, 147.28, 138.30, 134.63, 134.31, 133.43, 130.98, 129.77, 129.52, 129.05, 128.43, 127.81, 125.33, 81.62, 51.86, 51.71, 50.66, 50.57, 50.47, 50.46, 50.01, 49.90, 44.56, 43.64, 39.59, 38.29, 37.02, 36.55, 36.42, 36.33, 35.35, 33.10, 31.05, 30.91, 28.28, 28.17,

28.07, 26.46, 25.79, 24.99, 24.34, 24.17, 22.90, 22.61, 21.16, 21.11, 19.28, 18.79, 18.71, 18.23 (2), 17.70, 16.90, 15.84, -2.43, -3.02. (<u>see spectrum</u>)

HRMS (ESI) (m/z): $[M+H]^+$ calculated for $C_{53}H_{77}O_6Si^+$:837.5484, found: 837.5483.

dimethyl 2-(2-(dimethyl(phenyl)silyl)-1-(4-((((3*S*,8*S*,9*S*,10*R*,13*R*,14*S*,17*R*)-17-((2*R*,5*S*,*E*)-5-ethyl-6-methylhept-3-en-2-yl)-10,13-dimethyl-2,3,4,7,8,9,10,11,12,13,14,15,16,17-tetradecahydro-1H-cyclopenta[a]phenanthren-3-yl)oxy)carbonyl)phenyl)ethyl)succinate (d-72)

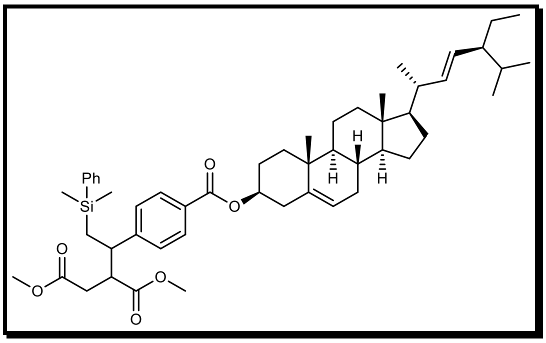

Following the **General procedure A**, **a-72** (128.5 mg, 0.2 mmol), **b-1** (64.9 mg, 0.4 mmol) and **c-1** (43.2 mg, 0.3 mmol) were used. Purification by column chromatography using silica with pentane/EtOAc (20:1 to 5:1, v/v) as eluent afforded **d-72** (100.3 mg, 61% yield) as a white solid.

^{1}H NMR (400 MHz, Chloroform-*d*) δ 7.92 – 7.85 (m, 2H), 7.36 – 7.25 (m, 5H), 7.11 (dd, *J* = 24.0, 7.9 Hz, 2H), 5.42 (d, *J* = 5.1 Hz, 1H), 5.16 (dd, *J* = 15.1, 8.5 Hz, 1H), 5.02 (dd, *J* = 15.2, 8.4 Hz, 1H), 4.85 (dtd, *J* = 12.2, 8.4, 4.4 Hz, 1H), 3.66 (d, *J* = 44.0 Hz, 3H), 3.48 (d, *J* = 36.0 Hz, 3H), 3.12 – 2.92 (m, 2H), 2.74 – 2.45 (m, 3H), 2.00 (td, *J* = 32.3, 30.8, 14.1 Hz, 6H), 1.76 – 1.68 (m, 2H), 1.50 (qd, *J* = 18.2, 17.2, 5.6 Hz, 8H), 1.32 – 1.12 (m, 8H), 1.08 (s, 3H), 1.04 (t, *J* = 6.0 Hz, 5H), 0.83 (dd, *J* = 20.2, 6.3 Hz, 9H), 0.71 (s, 3H), -0.06 (s, 6H). (<u>see spectrum</u>)

major: ^{13}C NMR (101 MHz, Chloroform-*d*) δ 174.56, 172.17, 165.75, 147.42, 139.73, 138.39, 138.32, 138.29, 133.45, 129.63, 129.57, 129.36, 128.97, 128.28, 127.80, 122.87, 74.65, 56.87, 56.01, 51.86, 51.75, 51.30, 50.47, 50.14, 44.17, 43.70, 42.29, 40.57, 39.71, 38.30, 37.11, 36.74, 33.18, 32.00, 31.95, 28.99, 27.96, 25.48, 24.44, 21.30, 21.16, 21.11 (2), 19.45, 19.06, 12.32, 12.13, -2.23, -3.14. **minor**: **^{13}C NMR** (101 MHz,

Chloroform-*d*) δ 173.86, 172.46, 165.85, 147.37, 139.71, 138.39, 138.32, 138.29, 133.45, 129.87, 129.80, 129.36, 129.07, 128.39, 127.84, 122.84, 74.65, 56.87, 56.01, 51.92, 51.69, 51.30, 50.14, 50.04, 44.17, 43.70, 42.29, 40.57, 39.71, 38.30, 37.11, 36.74, 34.75, 32.00, 31.95, 28.99, 27.96, 25.48, 24.44, 21.30, 21.16, 21.11, 19.45, 19.06, 18.36, 12.32, 12.13, -2.23, -3.14. (see spectrum)

HRMS (ESI) (m/z): $[M+H]^+$ calculated for $C_{52}H_{75}O_6Si^+$: 823.5327, found: 823.5324.

dimethyl 2-(1-([1,1'-biphenyl]-4-yl)ethyl)succinate (d-73)

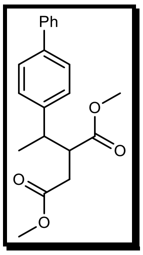

Following the **General procedure B**, **a-1** (56.0 mg, 0.2 mmol), **b-73** (1.0 bar) and **c-1** (43.2 mg, 0.3 mmol) were used. Purification by column chromatography using silica with pentane/EtOAc (30:1 to 7:1, v/v) as eluent afforded **d-73** (29.3 mg, 45% yield) as a white solid.

^{1}H NMR (400 MHz, Chloroform-*d*) δ 7.60 – 7.52 (m, 4H), 7.44 (t, *J* = 7.5 Hz, 2H), 7.37 – 7.32 (m, 1H), 7.30 – 7.22 (m, 2H), 3.70 (dd, *J* = 55.4, 1.3 Hz, 3H), 3.59 (dd, *J* = 7.8, 1.3 Hz, 3H), 3.33 – 2.92 (m, 2H), 2.82 – 2.60 (m, 1H), 2.33 (ddd, *J* = 45.2, 16.9, 3.7 Hz, 1H), 1.32 (d, *J* = 6.9 Hz, 3H). (see spectrum)

major: ^{13}C NMR (101 MHz, Chloroform-*d*) δ 175.17, 172.46, 142.60, 140.78, 139.94, 128.84, 127.89, 127.50, 127.32, 127.06, 51.91, 51.85, 48.40, 42.14, 35.39, 20.33.

minor: **^{13}C NMR** (101 MHz, Chloroform-*d*) δ 174.30, 172.73, 142.22, 140.82, 139.72, 128.84, 127.99, 127.29, 127.16, 127.06, 51.85, 51.78, 48.14, 40.43, 32.32, 16.42. (see spectrum)

HRMS (ESI) (m/z): $[M+H]^+$ calculated for $C_{20}H_{23}O_4^+$: 327.1591, found: 327.1595.

dimethyl 2-(1-([1,1'-biphenyl]-4-yl)-4-((2-(4-isobutylphenyl)propanoyl)oxy)butyl)succinate (d-74)

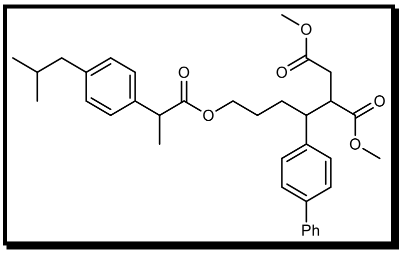

Following the **General procedure B**, **a-1** (56.0 mg, 0.2 mmol), **b-74** (156.1 mg, 0.6 mmol) and **c-1** (43.2 mg, 0.3 mmol) were used. Purification by column chromatography using silica with pentane/EtOAc (20:1 to 5:1, v/v) as eluent afforded **d-74** (48.0 mg, 43% yield) as a white solid.

^{1}H NMR (400 MHz, Chloroform-*d*) δ 7.62 (d, *J* = 7.3 Hz, 2H), 7.56 (dd, *J* = 8.0, 5.6 Hz, 2H), 7.48 (td, *J* = 7.6, 1.8 Hz, 2H), 7.38 (td, *J* = 7.2, 1.9 Hz, 1H), 7.27 – 7.20 (m, 2H), 7.19 – 7.11 (m, 4H), 4.12 – 3.98 (m, 2H), 3.81 – 3.66 (m, 4H), 3.63 – 3.55 (m, 3H), 3.17 – 3.02 (m, 1H), 3.02 – 2.80 (m, 1H), 2.79 – 2.57 (m, 1H), 2.49 – 2.22 (m, 3H), 1.87 (dq, *J* = 13.8, 6.7 Hz, 1H), 1.78 – 1.64 (m, 2H), 1.54 – 1.38 (m, 5H), 0.93 (d, *J* = 6.6 Hz, 6H). (see spectrum)

major: ^{13}C NMR (101 MHz, Chloroform-*d*) δ 174.99, 174.74, 172.38, 140.64 140.61, 140.10, 139.79, 137.92, 129.40, 129.38, 128.87, 128.55, 127.40, 127.24, 127.20, 127.04, 64.32, 51.96, 51.78, 47.72, 47.40, 45.19, 45.09, 35.08, 30.23, 30.17, 26.63, 22.46, 18.58. **minor**: **^{13}C NMR** (101 MHz, Chloroform-*d*) δ 174.76, 173.97, 172.55, 140.73, 140.57, 139.90, 139.62, 137.83, 129.40, 129.38, 128.84, 128.70, 127.52, 127.34, 127.24, 127.04, 64.24, 51.87, 51.78, 47.63, 46.64, 45.28, 45.09, 33.33, 30.23, 27.47, 26.80, 22.46, 18.51. (see spectrum)

HRMS (ESI) (m/z): $[M+H]^+$ calculated for $C_{35}H_{43}O_6^+$: 559.3054, found: 559.3054.

dimethyl 2-(1-([1,1'-biphenyl]-4-yl)-4-((2-(3-benzoylphenyl)propanoyl)oxy)butyl)succinate (d-75)

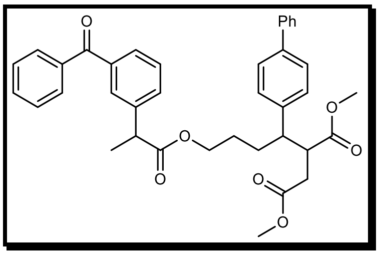

Following the **General procedure B**, **a-1** (56.0 mg, 0.2 mmol), **b-75** (184.9 mg, 0.6 mmol) and **c-1** (43.2 mg, 0.3 mmol) were used. Purification by column chromatography using silica with pentane/EtOAc (20:1 to 3:1, v/v) as eluent afforded **d-75** (58.2 mg, 48% yield) as a white solid.

^{1}H NMR (400 MHz, Chloroform-*d*) δ 7.82 – 7.73 (m, 3H), 7.68 (dd, *J* = 8.0, 3.7 Hz, 1H), 7.59 – 7.39 (m, 12H), 7.33 (dd, *J* = 8.6, 6.4 Hz, 1H), 7.14 (ddd, *J* = 8.2, 5.7, 2.5 Hz, 2H), 4.02 (dt, *J* = 22.1, 6.1 Hz, 2H), 3.78 (ddd, *J* = 11.6, 5.7, 2.7 Hz, 1H), 3.67 (d, *J* = 42.6 Hz, 3H), 3.58 – 3.51 (m, 3H), 3.06 (ddt, *J* = 24.6, 8.8, 4.7 Hz, 1H), 2.98 – 2.77 (m, 1H), 2.73 – 2.53 (m, 1H), 2.31 (ddt, *J* = 78.2, 16.7, 3.8 Hz, 1H), 1.68 (tt, *J* = 24.0, 14.2 Hz, 2H), 1.52 (t, *J* = 7.6 Hz, 4H), 1.44 – 1.38 (m, 1H). (*see spectrum*)

major: ^{13}C NMR (101 MHz, Chloroform-*d*) δ 196.45, 174.95, 174.05, 172.33, 141.04, 140.57, 140.12, 139.93, 139.73, 137.98, 137.96, 137.56, 132.57, 131.60, 130.13, 129.24, 129.11, 128.87, 128.61, 128.54, 128.38, 127.55, 127.41, 127.02, 64.60, 51.97, 51.78, 47.69, 47.56, 45.50, 35.12, 33.41, 30.16, 26.61, 18.54. **minor**: **^{13}C NMR** (101 MHz, Chloroform-*d*) δ 196.43, 174.05, 173.90, 172.49, 141.48, 140.66, 140.12, 139.93, 139.56, 137.98, 137.96, 137.56, 132.57, 131.55, 130.13, 129.31, 129.16, 128.84, 128.68, 128.63, 128.38, 127.35, 127.21, 127.02, 64.60, 51.86, 51.78, 47.38, 46.68, 45.46, 35.12, 33.41, 27.58, 26.80, 18.50. (*see spectrum*)

HRMS (ESI) (m/z): $[M+H]^+$ calculated for $C_{38}H_{39}O_7^+$: 607.2690, found: 607.2699.

dimethyl 2-(1-([1,1'-biphenyl]-4-yl)-4-((2-(2-fluoro-[1,1'-biphenyl]-4-yl)propanoyl)oxy)butyl)succinate (d-76)

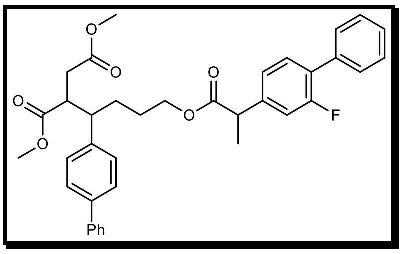

Following the **General procedure B**, **a-1** (56.0 mg, 0.2 mmol), **b-76** (178.9 mg, 0.6 mmol) and **c-1** (43.2 mg, 0.3 mmol) were used. Purification by column chromatography using silica with pentane/EtOAc (20:1 to 5:1, v/v) as eluent afforded **d-76** (47.7 mg, 40% yield) as a colorless oil.

^{1}H NMR (400 MHz, Chloroform-*d*) δ 7.57 – 7.48 (m, 6H), 7.43 – 7.30 (m, 7H), 7.17 – 7.06 (m, 4H), 4.12 – 3.98 (m, 2H), 3.75 – 3.58 (m, 4H), 3.57 – 3.51 (m, 3H), 3.13 – 2.99 (m, 1H), 2.88 (dtd, *J* = 58.2, 10.5, 9.9, 5.2 Hz, 1H), 2.74 – 2.52 (m, 1H), 2.44 – 2.17 (m, 1H), 1.80 – 1.62 (m, 2H), 1.54 – 1.36 (m, 5H). (*see spectrum*)

major: ^{13}C NMR (101 MHz, Chloroform-*d*) δ 174.99, 173.97, 172.34,159.76 (*J* = 249.42 Hz), 141.95 (*J* = 7.27 Hz), 140.58, 140.14, 139.76, 135.52, 130.87 (*J* = 8.34 Hz), 129.02 (*J* = 4.03 Hz), 128.88, 128.53 (2), 127.97 (*J* = 13.64 Hz), 127.74, 127.56, 127.41, 127.03, 123.65 (*J* = 3.68 Hz), 115.31 (*J* = 23.88 Hz), 64.66, 51.97, 51.79, 47.73, 47.40, 45.12, 35.15, 30.22, 26.63, 18.36. **minor**: **^{13}C NMR** (101 MHz, Chloroform-*d*) δ 173.97, 172.50, 172.50, 159.76 (*J* = 249.51 Hz), 141.97 (*J* = 7.46 Hz), 140.66, 139.95, 139.76, 135.52, 130.93 (*J* = 6.81 Hz), 129.02 (*J* = 4.03 Hz), 128.85, 128.68, 128.53, 127.95 (*J* = 13.64 Hz), 127.74, 127.35, 127.22, 127.03, 123.62 (*J* = 3.68 Hz), 115.38 (*J* = 23.76 Hz), 64.61, 51.86, 51.79, 47.59, 46.65, 45.16, 33.33, 27.58, 26.83, 18.45. (*see spectrum*)

major: ^{19}F NMR (376 MHz, Chloroform-*d*) δ -117.48 (t, *J* = 10.0 Hz). **minor**: **^{19}F NMR** (376 MHz, Chloroform-*d*) δ -117.40 (t, *J* = 10.0 Hz). (*see spectrum*)

HRMS (ESI) (m/z): $[M+H]^+$ calculated for $C_{37}H_{38}FO_6^+$: 597.2647, found: 597.2651.

dimethyl 2-(1-([1,1'-biphenyl]-4-yl)-4-(((S)-2-(6-methoxynaphthalen-2-yl)propanoyl)oxy)butyl)succinate (d-77)

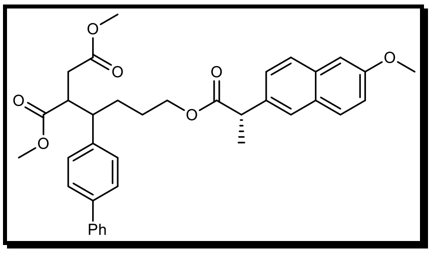

Following the **General procedure B**, **a-1** (56.0 mg, 0.2 mmol), **b-77** (170.5 mg, 0.6 mmol) and **c-1** (43.2 mg, 0.3 mmol) were used. Purification by column chromatography using silica with pentane/EtOAc (20:1 to 5:1, v/v) as eluent afforded **d-77** (47.7 mg, 41% yield) as a white solid.

^{1}H NMR (400 MHz, Chloroform-*d*) δ 7.75 – 7.65 (m, 3H), 7.55 (d, *J* = 7.7 Hz, 2H), 7.42 (p, *J* = 7.0, 6.6 Hz, 5H), 7.37 – 7.32 (m, 1H), 7.13 (td, *J* = 7.6, 6.3, 2.7 Hz, 2H),

7.06 – 6.95 (m, 2H), 4.13 – 3.94 (m, 2H), 3.90 (d, *J* = 4.0 Hz, 3H), 3.83 (dt, *J* = 11.9, 5.7 Hz, 1H), 3.72 – 3.63 (m, 3H), 3.58 – 3.50 (m, 3H), 3.06 – 2.72 (m, 2H), 2.69 – 2.48 (m, 1H), 2.34 – 2.11 (m, 1H), 1.57 (t, *J* = 7.8 Hz, 5H), 1.39 (ddd, *J* = 25.4, 11.7, 6.5 Hz, 2H). (*see spectrum*)

major: ^{13}**C NMR** (101 MHz, Chloroform-*d*) δ 174.98, 174.66, 172.39, 157.75, 140.62, 140.00, 130.69, 135.79, 133.78, 129.35, 129.01, 128.85, 128.47, 127.45, 127.37, 127.31, 127.02, 126.38, 126.11, 119.11, 105.68, 64.40, 55.36, 51.92, 51.77, 47.67, 47.35, 45.64, 34.98, 30.17, 26.67, 18.50. **minor**: ^{13}**C NMR** (101 MHz, Chloroform-*d*) δ 174.66, 173.94, 172.53, 157.75, 140.70, 139.79, 139.55, 135.91, 133.78, 129.35, 129.01, 128.82, 128.61, 127.31, 127.23, 127.12, 127.02, 126.24, 125.96, 119.14, 105.71, 64.32, 55.33, 51.86, 51.75, 47.63, 46.59, 45.51, 33.18, 27.42, 26.85, 18.42. (*see spectrum*)

HRMS (ESI) (m/z): $[M+H]^+$ calculated for $C_{36}H_{39}O_7^+$: 583.2690, found: 583.2686.

dimethyl 2-(1-([1,1'-biphenyl]-4-yl)-4-(2-(11-oxo-6,11-dihydrodibenzo[b,e]oxepin-2-yl)acetoxy)butyl)succinate (d-78)

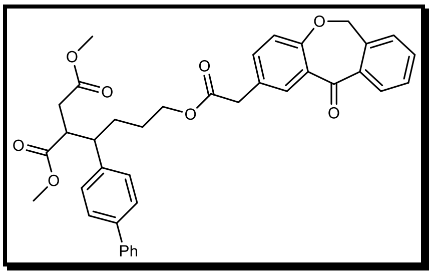

Following the **General procedure B**, **a-1** (56.0 mg, 0.2 mmol), **b-78** (193.3 mg, 0.6 mmol) and **c-1** (43.2 mg, 0.3 mmol) were used. Purification by column chromatography using silica with pentane/EtOAc (20:1 to 5:1, v/v) as eluent afforded **d-78** (53.3 mg, 43% yield) as a white solid.

^{1}**H NMR** (400 MHz, Chloroform-*d*) δ 8.12 (dd, *J* = 10.0, 2.4 Hz, 1H), 7.89 (dd, *J* = 7.7, 1.4 Hz, 1H), 7.59 – 7.50 (m, 5H), 7.47 – 7.38 (m, 4H), 7.36 – 7.31 (m, 2H), 7.20 – 7.14 (m, 2H), 7.03 (dd, *J* = 8.4, 3.4 Hz, 1H), 5.17 (s, 2H), 4.04 (dt, *J* = 21.5, 6.5 Hz, 2H), 3.75 – 3.59 (m, 5H), 3.55 (d, *J* = 8.8 Hz, 3H), 3.16 – 3.02 (m, 1H), 3.01 – 2.79 (m, 1H), 2.65 (ddd, *J* = 58.4, 16.8, 10.8 Hz, 1H), 2.34 (ddd, *J* = 92.6, 16.8, 3.9 Hz, 1H), 1.81 – 1.67 (m, 2H), 1.56 – 1.39 (m, 2H). (*see spectrum*)

major: ^{13}**C NMR** (101 MHz, Chloroform-*d*) δ 190.85, 174.99, 172.35, 171.42, 160.55, 140.61, 140.50, 140.13, 139.75, 136.38, 135.66, 132.84, 132.50, 129.59, 129.33, 128.85, 128.56, 127.90, 127.87, 127.57, 127.38, 127.04, 125.21, 121.14, 73.70, 64.67, 52.00, 51.78, 47.70, 47.41, 40.28, 35.14, 30.21, 26.60. **minor**: ^{13}**C NMR** (101 MHz, Chloroform-*d*) δ 190.82, 173.93, 172.52, 171.42, 160.57, 140.69, 140.48, 139.94, 139.56, 136.38, 135.66, 132.84, 132.50, 129.59, 129.33, 128.83, 128.71, 127.92, 127.87, 127.32, 127.22, 127.04, 125.23, 121.16, 73.70, 64.67, 51.87, 51.78, 47.58, 46.72, 40.36, 33.44, 27.69, 26.80. (*see spectrum*)

HRMS (ESI) (m/z): $[M+H]^+$ calculated for $C_{38}H_{37}O_8^+$: 621.2483, found: 621.2475.

dimethyl 2-(1-([1,1'-biphenyl]-4-yl)-4-((2-(10-oxo-10,11-dihydrodibenzo[b,f]thiepin-2-yl)propanoyl)oxy)butyl)succinate (d-79)

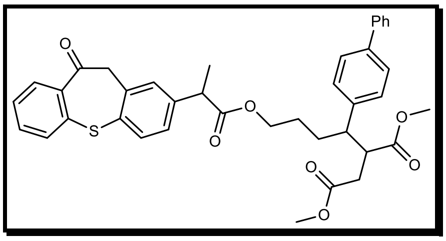

Following the **General procedure B**, **a-1** (56.0 mg, 0.2 mmol), **b-79** (211.3 mg, 0.6 mmol) and **c-1** (43.2 mg, 0.3 mmol) were used. Purification by column chromatography using silica with pentane/EtOAc (20:1 to 5:1, v/v) as eluent afforded **d-79** (53.3 mg, 41% yield) as a colorless oil.

^{1}**H NMR** (400 MHz, Chloroform-*d*) δ 8.19 (dd, *J* = 7.9, 1.6 Hz, 1H), 7.59 (td, *J* = 7.3, 3.0 Hz, 4H), 7.51 (dd, *J* = 8.1, 6.0 Hz, 2H), 7.43 (dd, *J* = 9.8, 7.6 Hz, 3H), 7.39 – 7.33 (m, 2H), 7.32 – 7.28 (m, 1H), 7.17 – 7.06 (m, 3H), 4.35 (d, *J* = 2.5 Hz, 2H), 4.07 – 3.90 (m, 2H), 3.73 – 3.64 (m, 4H), 3.59 – 3.50 (m, 3H), 3.12 – 2.99 (m, 1H), 2.97 – 2.75 (m, 1H), 2.73 – 2.52 (m, 1H), 2.30 (ddt, *J* = 82.6, 16.8, 3.6 Hz, 1H), 1.74 – 1.59 (m, 2H), 1.49 – 1.34 (m, 5H). (*see spectrum*)

major: ^{13}**C NMR** (101 MHz, Chloroform-*d*) δ 191.37, 174.97, 173.90, 172.34, 142.82, 140.59, 140.25, 140.11, 139.75, 138.00, 136.21, 133.28, 132.58, 131.60, 131.56, 130.90, 128.87, 128.67, 128.52, 127.54, 127.40, 127.04, 126.91, 126.41, 64.63, 51.96, 51.79, 51.10, 47.67, 47.37, 45.24, 35.17, 30.16, 26.75, 18.55. **minor**: ^{13}**C NMR** (101

MHz, Chloroform-*d*) δ 191.37, 173.90 (2), 172.49, 142.78, 140.68, 140.25, 139.93, 139.55, 138.02, 136.21, 133.31, 132.58, 131.60, 131.55, 130.90, 128.84, 128.71, 128.67, 127.54, 127.40, 127.04, 126.91, 126.41, 64.58, 51.90, 51.76, 51.10, 47.50, 46.66, 45.26, 33.42, 27.60, 26.75, 18.50. (<u>*see spectrum*</u>)

HRMS (ESI) (m/z): $[M+H]^+$ calculated for $C_{39}H_{39}O_7S^+$: 651.2411, found: 651.2404.

dimethyl 2-(1-([1,1'-biphenyl]-4-yl)-4-((2-(4-(4-chlorobenzoyl)phenoxy)-2-methylpropanoyl)oxy)butyl)succinate (d-80)

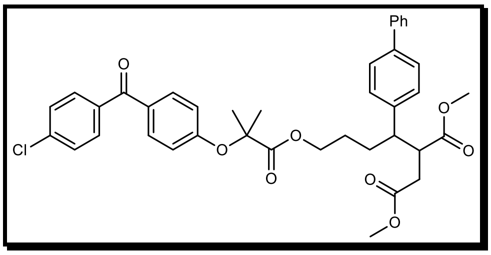

Following the **General procedure B**, **a-1** (56.0 mg, 0.2 mmol), **b-80** (223.3 mg, 0.6 mmol) and **c-1** (43.2 mg, 0.3 mmol) were used. Purification by column chromatography using silica with pentane/EtOAc (20:1 to 5:1, v/v) as eluent afforded **d-80** (64.3 mg, 48% yield) as a white solid.

^{1}H NMR (400 MHz, Chloroform-*d*) δ 7.71 (ddt, *J* = 16.5, 8.6, 2.5 Hz, 4H), 7.53 (dd, *J* = 15.0, 7.3 Hz, 4H), 7.45 – 7.39 (m, 4H), 7.33 (t, *J* = 7.5 Hz, 1H), 7.13 (dt, *J* = 8.2, 2.1 Hz, 2H), 6.84 (td, *J* = 8.7, 2.0 Hz, 2H), 4.10 (dt, *J* = 19.9, 6.5 Hz, 2H), 3.66 (d, *J* = 47.4 Hz, 3H), 3.54 (d, *J* = 9.6 Hz, 3H), 3.13 – 2.97 (m, 1H), 2.86 (ddd, *J* = 66.3, 13.1, 7.4 Hz, 1H), 2.59 (ddd, *J* = 39.3, 16.8, 10.8 Hz, 1H), 2.39 – 2.16 (m, 1H), 1.66 (d, *J* = 9.7 Hz, 8H), 1.50 (q, *J* = 7.3 Hz, 1H), 1.40 (p, *J* = 7.3 Hz, 1H). (<u>*see spectrum*</u>)

major: ^{13}C NMR (101 MHz, Chloroform-*d*) δ 194.19, 174.91, 173.67, 172.25, 159.66, 140.48, 140.18, 139.60, 138.41, 136.41, 132.11, 131.24, 130.45, 128.87, 128.65, 128.60, 127.55, 127.43, 126.99, 117.33, 79.43, 65.36, 51.97, 51.78, 47.64, 47.31, 35.16, 30.18, 26.47, 25.54, 25.37. **minor**: **^{13}C NMR** (101 MHz, Chloroform-*d*) δ 194.14, 173.79, 173.76, 172.41, 159.69, 140.57, 139.98, 139.38, 138.41, 136.39, 132.13, 131.24, 130.45, 128.84, 128.60, 128.50, 127.37, 127.21, 126.99, 117.23, 79.43, 65.36, 51.86, 51.78, 47.44, 46.58, 33.29, 27.51, 26.69, 25.59, 25.37. (<u>*see spectrum*</u>)

HRMS (ESI) (m/z): $[M+H]^+$ calculated for $C_{39}H_{40}ClO_8^+$: 671.2406, found: 671.2403.

dimethyl 2-([1,1'-biphenyl]-4-yl)succinate (e-1)

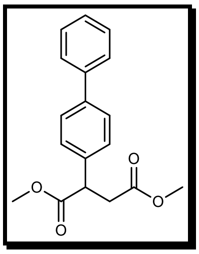

Following the **General procedure A**, **a-1** (56.0 mg, 0.2 mmol), **b-1** (64.9 mg, 0.4 mmol) and **c-1** (43.2 mg, 0.3 mmol) were used. Purification by column chromatography using silica with pentane/EtOAc (20:1 to 3:1, v/v) as eluent afforded **e-1** (7.2 mg, 12% yield) as a white solid.

^{1}H NMR (400 MHz, Chloroform-*d*) δ 7.60 – 7.53 (m, 4H), 7.44 (t, *J* = 7.7 Hz, 2H), 7.36 (d, *J* = 8.4 Hz, 3H), 4.15 (dd, *J* = 10.0, 5.3 Hz, 1H), 3.70 (d, *J* = 5.9 Hz, 6H), 3.26 (dd, *J* = 17.0, 10.0 Hz, 1H), 2.72 (dd, *J* = 17.0, 5.3 Hz, 1H). (*see spectrum*)

^{13}C NMR (101 MHz, Chloroform-*d*) δ 173.51, 172.07, 140.73, 140.58, 136.71, 128.87, 128.23, 127.69, 127.50, 127.13, 52.52, 52.00, 46.82, 37.66. (*see spectrum*)

HRMS (ESI) (m/z): $[M+H]^+$ calculated for $C_{18}H_{19}O_4^+$: 299.1278, found: 299.1276.

2-(2-(dimethyl(phenyl)silyl)-1-phenylethyl)quinoxaline (d-81)

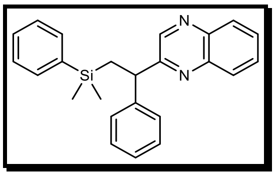

Following the **General procedure D**, **a-2** (40.8 mg, 0.2 mmol), **b-1** (64.9 mg, 0.4 mmol) and **c-81** (39.0 mg, 0.3 mmol) were used. Purification by column chromatography using silica with pentane/EtOAc (20:1 to 3:1, v/v) as eluent afforded **d-81** (53.0 mg, 72% yield) as a colorless oil.

^{1}H NMR (400 MHz, Chloroform-*d*) δ 8.59 (s, 1H), 8.07 (dd, J = 8.3, 1.6 Hz, 1H), 8.00 (dd, J = 8.1, 1.6 Hz, 1H), 7.71 (dddd, J = 21.3, 8.4, 6.9, 1.6 Hz, 2H), 7.37 (ddd, J = 8.1, 4.0, 1.4 Hz, 4H), 7.29 – 7.15 (m, 7H), 4.43 (dd, J = 8.6, 7.2 Hz, 1H), 2.08 (dd, J = 14.7, 8.6 Hz, 1H), 1.75 (dd, J = 14.7, 7.2 Hz, 1H), 0.12 (d, J = 13.3 Hz, 6H). (*see spectrum*)

^{13}C NMR (101 MHz, Chloroform-*d*) δ 159.47, 145.85, 144.14, 141.83, 141.02, 138.62, 133.55, 129.91, 129.21, 129.15, 129.02, 128.86, 128.78, 128.02, 127.72, 126.92, 47.99, 22.21, -2.27, -2.83. (*see spectrum*)

HRMS (ESI) (m/z): $[M+H]^+$ calculated for $C_{24}H_{25}N_2Si^+$: 369.1982, found: 369.1990.

2-(2-(dimethyl(phenyl)silyl)-1-(p-tolyl)ethyl)quinoxaline (d-82)

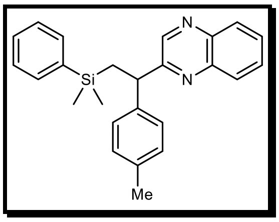

Following the **General procedure D**, **a-25** (43.6 mg, 0.2 mmol), **b-1** (64.9 mg, 0.4 mmol) and **c-81** (39.0 mg, 0.3 mmol) were used. Purification by column chromatography using silica with pentane/EtOAc (20:1 to 3:1, v/v) as eluent afforded **d-82** (42.0 mg, 55% yield) as a colorless oil.

^{1}H NMR (400 MHz, Chloroform-*d*) δ 8.57 (s, 1H), 8.01 (ddd, *J* = 27.2, 8.3, 1.2 Hz, 2H), 7.74 – 7.69 (m, 1H), 7.66 (td, *J* = 7.6, 6.9, 1.6 Hz, 1H), 7.40 – 7.32 (m, 2H), 7.27 – 7.18 (m, 5H), 7.05 (d, *J* = 7.8 Hz, 2H), 4.43 – 4.34 (m, 1H), 2.27 (s, 3H), 2.05 (dd, *J* = 14.7, 8.6 Hz, 1H), 1.72 (dd, *J* = 14.7, 7.2 Hz, 1H), 0.11 (d, *J* = 12.5 Hz, 6H). (*see spectrum*)

^{13}C NMR (101 MHz, Chloroform-*d*) δ 159.71, 145.89, 141.82, 141.14, 141.03, 138.71, 136.50, 133.55, 129.84, 129.44, 129.20, 129.06, 129.02, 128.81, 127.87, 127.69, 77.43, 77.11, 76.79, 47.60, 22.17, 21.06, -2.26, -2.78. (*see spectrum*)

HRMS (ESI) (m/z): $[M+H]^+$ calculated for $C_{25}H_{27}N_2Si^+$: 383.1938, found: 383.1932.

2-(1-(4-(tert-butyl)phenyl)-2-(dimethyl(phenyl)silyl)ethyl)quinoxaline (d-83)

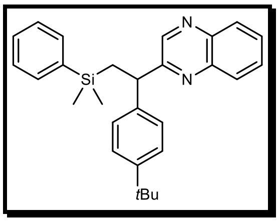

Following the **General procedure D**, **a-3** (52.0 mg, 0.2 mmol), **b-1** (64.9 mg, 0.4 mmol) and **c-81** (39.0 mg, 0.3 mmol) were used. Purification by column chromatography using

silica with pentane/EtOAc (20:1 to 10:1, v/v) as eluent afforded **d-83** (49.2 mg, 58% yield) as a colorless oil.

^{1}H NMR (400 MHz, Chloroform-*d*) δ 8.59 (s, 1H), 8.05 (dd, *J* = 8.3, 1.6 Hz, 1H), 7.98 (dd, *J* = 8.2, 1.6 Hz, 1H), 7.71 (ddd, *J* = 8.4, 6.9, 1.6 Hz, 1H), 7.66 (ddd, *J* = 8.3, 6.9, 1.6 Hz, 1H), 7.37 – 7.32 (m, 2H), 7.29 – 7.24 (m, 4H), 7.24 – 7.16 (m, 3H), 4.40 (dd, *J* = 8.9, 6.8 Hz, 1H), 2.07 (dd, *J* = 14.7, 8.9 Hz, 1H), 1.72 (dd, *J* = 14.7, 6.8 Hz, 1H), 1.25 (s, 9H), 0.11 (d, *J* = 11.8 Hz, 6H). (see spectrum)

^{13}C NMR (101 MHz, Chloroform-*d*) δ 159.68, 149.74, 146.01, 141.87, 141.12, 141.06, 138.72, 133.54, 129.82, 129.23, 129.04, 128.80, 127.68, 127.57, 125.63, 47.55, 34.45, 31.40, 22.19, -2.39, -2.75. (see spectrum)

HRMS (ESI) (m/z): $[M+H]^+$ calculated for $C_{28}H_{33}N_2Si^+$: 425.2408, found: 425.2412.

2-(1-([1,1'-biphenyl]-4-yl)-2-(dimethyl(phenyl)silyl)ethyl)quinoxaline (d-84)

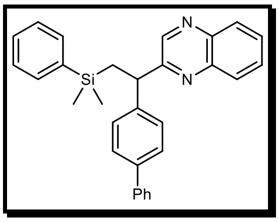

Following the **General procedure D**, **a-1** (56.0 mg, 0.2 mmol), **b-1** (64.9 mg, 0.4 mmol) and **c-81** (39.0 mg, 0.3 mmol) were used. Purification by column chromatography using silica with pentane/EtOAc (20:1 to 10:1, v/v) as eluent afforded **d-84** (46.2 mg, 52% yield) as a colorless oil.

^{1}H NMR (400 MHz, Chloroform-*d*) δ 8.67 (s, 1H), 8.12 (d, *J* = 8.2 Hz, 1H), 8.04 (d, *J* = 8.2 Hz, 1H), 7.80 – 7.70 (m, 2H), 7.57 (d, *J* = 8.1 Hz, 2H), 7.52 (d, *J* = 8.0 Hz, 2H), 7.44 (dd, *J* = 17.6, 8.4 Hz, 6H), 7.38 – 7.33 (m, 1H), 7.27 (d, *J* = 6.8 Hz, 3H), 4.50 (t, *J* = 7.9 Hz, 1H), 2.15 (dd, *J* = 14.6, 8.6 Hz, 1H), 1.83 (dd, *J* = 14.7, 7.1 Hz, 1H), 0.19 (dd, *J* = 9.5, 1.2 Hz, 6H). (see spectrum)

^{13}C NMR (101 MHz, Chloroform-*d*) δ 159.38, 145.94, 143.28, 141.96, 141.20, 140.83, 139.79, 138.59, 133.55, 129.87, 129.28, 129.12, 128.85, 128.79, 128.41, 127.72, 127.46, 127.27, 127.08, 47.71, 22.32, -2.32, -2.68. (see spectrum)

HRMS (ESI) (m/z): $[M+H]^+$ calculated for $C_{30}H_{29}N_2Si^+$: 445.2095, found: 445.2102.

2-(2-(dimethyl(phenyl)silyl)-1-(4-fluorophenyl)ethyl)quinoxaline (d-85)

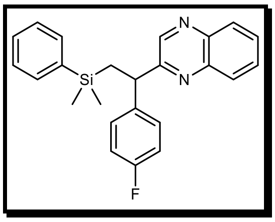

Following the **General procedure D**, **a-9** (44.4 mg, 0.2 mmol), **b-1** (64.9 mg, 0.4 mmol) and **c-81** (39.0 mg, 0.3 mmol) were used. Purification by column chromatography using silica with pentane/EtOAc (20:1 to 10:1, v/v) as eluent afforded **d-85** (48.6 mg, 63% yield) as a colorless oil.

^{1}H NMR (400 MHz, Chloroform-*d*) δ 8.04 – 8.00 (m, 2H), 8.00 – 7.96 (m, 2H), 7.56 (t, *J* = 7.4 Hz, 1H), 7.51 (t, *J* = 7.3 Hz, 1H), 7.43 (dt, *J* = 15.1, 7.7 Hz, 4H), 7.34 (dd, *J* = 8.5, 5.4 Hz, 2H), 7.00 (t, *J* = 8.6 Hz, 2H), 5.33 (dd, *J* = 9.8, 3.9 Hz, 1H), 4.17 (dd, *J* = 18.0, 9.8 Hz, 1H), 3.31 (dd, *J* = 18.0, 4.0 Hz, 1H). (*see spectrum*)

^{13}C NMR (101 MHz, Chloroform-*d*) δ 163.01, 160.57, 159.28, 145.74, 141.91, 141.18, 139.90, 139.87, 138.42, 133.53, 129.95, 129.54, 129.46, 129.25, 129.21, 129.13, 128.93, 127.75, 115.62, 115.41, 47.24, 22.58, -2.31, -2.76. (*see spectrum*)

^{19}F NMR (376 MHz, Chloroform-*d*) δ -115.90 – -116.14 (m). (*see spectrum*)

HRMS (ESI) (m/z): $[M+H]^+$ calculated for $C_{24}H_{24}FN_2Si^+$: 387.1687, found: 387.1681.

2-(1-(4-chlorophenyl)-2-(dimethyl(phenyl)silyl)ethyl)quinoxaline (d-86)

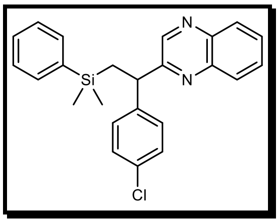

Following the **General procedure D**, **a-10** (47.7 mg, 0.2 mmol), **b-1** (64.9 mg, 0.4 mmol) and **c-81** (39.0 mg, 0.3 mmol) were used. Purification by column chromatography using silica with pentane/EtOAc (20:1 to 10:1, v/v) as eluent afforded **d-86** (45.0 mg, 56% yield) as a colorless oil.

^{1}H NMR (400 MHz, Chloroform-*d*) δ 8.54 (s, 1H), 8.02 (ddd, *J* = 20.0, 8.1, 1.7 Hz, 2H), 7.77 – 7.64 (m, 2H), 7.34 (dd, *J* = 7.8, 1.7 Hz, 2H), 7.30 – 7.15 (m, 7H), 4.37 (t, *J*

= 7.9 Hz, 1H), 2.04 (dd, *J* = 14.7, 8.5 Hz, 1H), 1.70 (dd, *J* = 14.7, 7.3 Hz, 1H), 0.12 (d, *J* = 8.4 Hz, 6H). (see spectrum)

^{13}C NMR (101 MHz, Chloroform-*d*) δ 158.94, 145.63, 142.66, 141.89, 141.19, 138.31, 133.50, 132.68, 130.01, 129.36, 129.28, 129.23, 129.12, 128.93, 128.83, 127.76, 47.36, 22.46, -2.33, -2.72. (see spectrum)

HRMS (ESI) (m/z): $[M+H]^+$ calculated for $C_{24}H_{24}ClN_2Si^+$: 403.1392, found: 403.1396.

2-(2-(dimethyl(phenyl)silyl)-1-(4-methoxyphenyl)ethyl)quinoxaline (d-87)

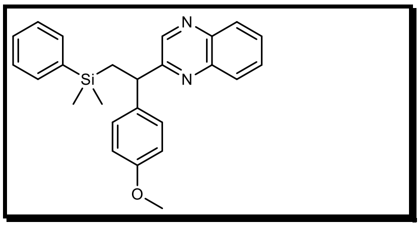

Following the **General procedure D**, **a-5** (46.8 mg, 0.2 mmol), **b-1** (64.9 mg, 0.4 mmol) and **c-81** (39.0 mg, 0.3 mmol) were used. Purification by column chromatography using silica with pentane/EtOAc (20:1 to 10:1, v/v) as eluent afforded **d-87** (37.4 mg, 47% yield) as a colorless oil.

^{1}H NMR (400 MHz, Chloroform-*d*) δ 8.57 (s, 1H), 8.01 (ddd, *J* = 26.1, 8.2, 1.6 Hz, 2H), 7.68 (dddd, *J* = 21.5, 8.3, 6.9, 1.6 Hz, 2H), 7.40 – 7.31 (m, 2H), 7.29 – 7.18 (m, 5H), 6.81 – 6.73 (m, 2H), 4.37 (t, *J* = 7.9 Hz, 1H), 3.74 (s, 3H), 2.01 (dd, *J* = 14.7, 8.2 Hz, 1H), 1.73 (dd, *J* = 14.7, 7.6 Hz, 1H), 0.12 (d, *J* = 10.3 Hz, 6H). (see spectrum)

^{13}C NMR (101 MHz, Chloroform-*d*) δ 159.86, 158.52, 145.93, 141.85, 141.07, 138.72, 136.20, 133.56, 129.83, 129.27, 129.21, 129.16, 129.07, 129.02, 128.84, 127.71, 114.25, 114.12, 55.31, 47.19, 22.28, -2.24, -2.77. (see spectrum)

HRMS (ESI) (m/z): $[M+H]^+$ calculated for $C_{25}H_{27}N_2OSi^+$: 399.1887, found: 399.1891.

methyl 4-(2-(dimethyl(phenyl)silyl)-1-(quinoxalin-2-yl)ethyl)benzoate (d-88)

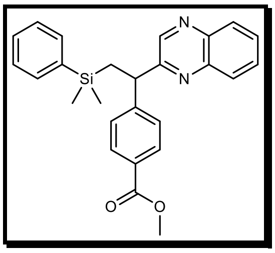

Following the **General procedure D**, **a-14** (52.4 mg, 0.2 mmol), **b-1** (64.9 mg, 0.4 mmol) and **c-81** (39.0 mg, 0.3 mmol) were used. Purification by column chromatography using silica with pentane/EtOAc (20:1 to 10:1, v/v) as eluent afforded **d-88** (54.5 mg, 64% yield) as a colorless oil.

^{1}H NMR (400 MHz, Chloroform-*d*) δ 8.56 (s, 1H), 8.06 (dd, *J* = 8.2, 1.1 Hz, 1H), 8.00 (dd, *J* = 8.2, 1.2 Hz, 1H), 7.92 (d, *J* = 8.3 Hz, 2H), 7.72 (dddd, *J* = 20.3, 8.4, 6.9, 1.6 Hz, 2H), 7.43 (d, *J* = 8.4 Hz, 2H), 7.35 (dd, *J* = 7.7, 1.7 Hz, 2H), 7.28 – 7.19 (m, 3H), 4.45 (dd, *J* = 8.6, 7.1 Hz, 1H), 3.88 (s, 3H), 2.10 (dd, *J* = 14.7, 8.6 Hz, 1H), 1.74 (dd, *J* = 14.7, 7.1 Hz, 1H), 0.12 (d, *J* = 8.7 Hz, 6H). (see spectrum)

^{13}C NMR (101 MHz, Chloroform-*d*) δ 166.93, 158.57, 149.44, 145.64, 141.94, 141.22, 138.24, 133.51, 130.08, 130.04, 129.35, 129.27, 129.13, 128.97, 128.74, 128.07, 127.77, 52.14, 47.98, 22.36, -2.29, -2.76. (see spectrum)

HRMS (ESI) (m/z): $[M+H]^+$ calculated for $C_{26}H_{27}N_2O_2Si^+$: 427.1836, found: 427.1832.

2-(2-(dimethyl(phenyl)silyl)-1-(4-(4-propylcyclohexyl)phenyl)ethyl)quinoxaline (d-89)

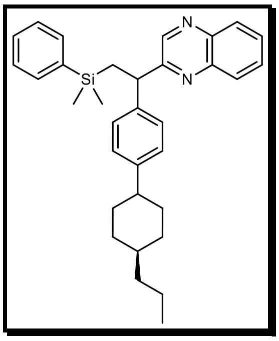

Following the **General procedure D**, **a-18** (68.5 mg, 0.2 mmol), **b-1** (64.9 mg, 0.4 mmol) and **c-81** (39.0 mg, 0.3 mmol) were used. Purification by column chromatography using silica with pentane/EtOAc (20:1 to 10:1, v/v) as eluent afforded **d-89** (47.2 mg, 48% yield) as a colorless oil.

^{1}H NMR (400 MHz, Chloroform-*d*) δ 8.58 (s, 1H), 8.05 (dd, *J* = 8.3, 1.6 Hz, 1H), 7.97 (dd, *J* = 8.2, 1.6 Hz, 1H), 7.71 (ddd, *J* = 8.4, 6.9, 1.7 Hz, 1H), 7.66 (ddd, *J* = 8.4, 6.9, 1.6 Hz, 1H), 7.38 – 7.31 (m, 2H), 7.28 – 7.17 (m, 5H), 7.08 (d, *J* = 8.0 Hz, 2H), 4.39 (dd, *J* = 8.9, 6.9 Hz, 1H), 2.39 (td, *J* = 12.2, 6.1 Hz, 1H), 2.06 (dd, *J* = 14.7, 8.9 Hz,

1H), 1.82 (d, *J* = 10.5 Hz, 4H), 1.72 (dd, *J* = 14.7, 6.9 Hz, 1H), 1.43 – 1.25 (m, 5H), 1.21 – 1.16 (m, 2H), 1.05 – 0.96 (m, 2H), 0.88 (t, *J* = 7.3 Hz, 3H), 0.10 (d, *J* = 11.9 Hz, 6H). (*see spectrum*)

^{13}C NMR (101 MHz, Chloroform-*d*) δ 159.69, 146.55, 146.01, 141.85, 141.49, 141.04, 138.72, 133.54, 129.81, 129.22, 129.03, 128.79, 127.82, 127.67, 127.18, 77.43, 77.11, 76.80, 44.24, 39.79, 37.05, 34.37, 33.62, 22.20, 20.10, 14.50, -2.35, -2.79. (*see spectrum*)

HRMS (ESI) (m/z): $[M+H]^+$ calculated for $C_{33}H_{41}N_2Si^+$: 493.3034, found: 493.3030.

2-(2-(dimethyl(phenyl)silyl)-1-(3,5-dimethylphenyl)ethyl)quinoxaline (d-90)

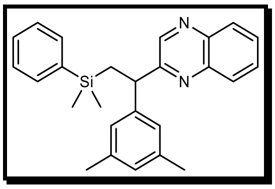

Following the **General procedure D**, **a-19** (46.4 mg, 0.2 mmol), **b-1** (64.9 mg, 0.4 mmol) and **c-81** (39.0 mg, 0.3 mmol) were used. Purification by column chromatography using silica with pentane/EtOAc (20:1 to 10:1, v/v) as eluent afforded **d-90** (36.4 mg, 46% yield) as a colorless oil.

^{1}H NMR (400 MHz, Chloroform-*d*) δ 8.60 (s, 1H), 8.09 (d, *J* = 8.2 Hz, 1H), 8.01 (d, *J* = 8.3 Hz, 1H), 7.72 (dt, *J* = 21.7, 7.3 Hz, 2H), 7.38 – 7.33 (m, 2H), 7.22 (q, *J* = 6.7 Hz, 3H), 6.94 (s, 2H), 6.80 (s, 1H), 4.43 – 4.31 (m, 1H), 2.24 (s, 6H), 0.14 (d, *J* = 7.0 Hz, 6H). (*see spectrum*)

^{13}C NMR (101 MHz, Chloroform-*d*) δ 159.61, 145.78, 143.86, 141.67, 140.80, 138.71, 138.26, 133.53, 129.93, 129.16, 129.13, 128.89, 128.78, 128.56, 127.63, 125.78, 47.84, 21.98, 21.34, -2.32, -2.75. (*see spectrum*)

HRMS (ESI) (m/z): $[M+ Na]^+$ calculated for $C_{26}H_{29}N_2Si^+$: 397.2095, found: 397.2090.

2-(2-(dimethyl(phenyl)silyl)-1-(naphthalen-2-yl)ethyl)quinoxaline (d-91)

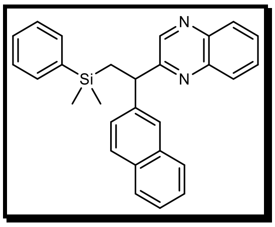

Following the **General procedure D**, **a-20** (50.8 mg, 0.2 mmol), **b-1** (64.9 mg, 0.4 mmol) and **c-81** (39.0 mg, 0.3 mmol) were used. Purification by column chromatography using silica with pentane/EtOAc (20:1 to 10:1, v/v) as eluent afforded **d-91** (31.8 mg, 38% yield) as a colorless oil.

^{1}H NMR (400 MHz, Chloroform-*d*) δ 8.62 (s, 1H), 8.04 (ddd, *J* = 40.6, 8.3, 1.6 Hz, 2H), 7.71 (ddd, *J* = 25.6, 11.9, 5.0 Hz, 6H), 7.49 – 7.40 (m, 3H), 7.39 – 7.34 (m, 2H), 7.25 – 7.17 (m, 3H), 4.59 (dd, *J* = 8.6, 7.1 Hz, 1H), 2.16 (dd, *J* = 14.7, 8.6 Hz, 1H), 1.84 (dd, *J* = 14.7, 7.1 Hz, 1H), 0.13 (d, *J* = 4.1 Hz, 6H). (see spectrum)

^{13}C NMR (101 MHz, Chloroform-*d*) δ 159.32, 145.92, 141.84, 141.53, 141.06, 138.57, 133.56, 132.47, 129.94, 129.23, 129.19, 129.07, 128.87, 128.55, 127.83, 127.71, 127.66, 126.39, 126.30, 126.21, 125.80, 48.12, 22.04, -2.23, -2.70. (see spectrum)

HRMS (ESI) (m/z): $[M+H]^+$ calculated for $C_{28}H_{27}N_2Si^+$: 419.1938, found: 419.1935.

2-(2-(diethyl(methyl)silyl)-1-phenylethyl)quinoxaline (d-92)

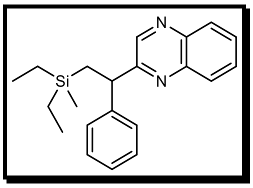

Following the **General procedure D**, **a-2** (40.8 mg, 0.2 mmol), **b-29** (51.3 mg, 0.4 mmol) and **c-81** (39.0 mg, 0.3 mmol) were used. Purification by column chromatography using silica with pentane/EtOAc (20:1 to 10:1, v/v) as eluent afforded **d-92** (45.4 mg, 68% yield) as a colorless oil.

^{1}H NMR (400 MHz, Chloroform-*d*) δ 8.74 (s, 1H), 8.08 (ddd, *J* = 29.8, 8.2, 1.6 Hz, 2H), 7.72 (dddd, *J* = 21.8, 8.4, 7.0, 1.6 Hz, 2H), 7.48 – 7.39 (m, 2H), 7.29 (t, *J* = 7.7 Hz, 2H), 7.19 (t, *J* = 7.3 Hz, 1H), 4.47 (t, *J* = 7.9 Hz, 1H), 1.80 (dd, *J* = 14.7, 8.1 Hz, 1H), 1.54 (dd, *J* = 14.7, 7.7 Hz, 1H), 0.85 (t, *J* = 7.9 Hz, 6H), 0.44 – 0.27 (m, 4H), -0.23 (s, 3H). (see spectrum)

^{13}C NMR (101 MHz, Chloroform-*d*) δ 159.97, 145.85, 144.44, 141.90, 141.09, 129.98, 129.23, 129.17, 129.07, 128.78, 128.00, 126.89, 48.09, 19.35, 7.34, 5.37, 5.35, -5.63. (see spectrum)

HRMS (ESI) (m/z): $[M+H]^+$ calculated for $C_{21}H_{27}N_2Si^+$: 335.1938, found: 335.1942.

2-(1-phenyl-2-(triethylsilyl)ethyl)quinoxaline (d-93)

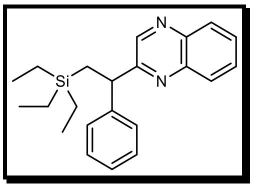

Following the **General procedure D**, **a-1** (56.0 mg, 0.2 mmol), **b-28** (56.9 mg, 0.4 mmol) and **c-81** (39.0 mg, 0.3 mmol) were used. Purification by column chromatography using silica with pentane/EtOAc (20:1 to 10:1, v/v) as eluent afforded **d-93** (43.2 mg, 62% yield) as a colorless oil.

^{1}H NMR (400 MHz, Chloroform-*d*) δ 8.74 (s, 1H), 8.07 (dd, *J* = 29.5, 8.2 Hz, 2H), 7.78 – 7.65 (m, 2H), 7.43 (d, *J* = 7.3 Hz, 2H), 7.28 (t, *J* = 7.6 Hz, 2H), 7.18 (t, *J* = 7.3 Hz, 1H), 4.46 (t, *J* = 7.8 Hz, 1H), 1.84 (dd, *J* = 14.7, 8.2 Hz, 1H), 1.51 (dd, *J* = 14.7, 7.3 Hz, 1H), 0.84 (t, *J* = 7.9 Hz, 9H), 0.41 – 0.30 (m, 6H). (*see spectrum*)

^{13}C NMR (101 MHz, Chloroform-*d*) δ 159.95, 145.92, 144.67, 141.93, 141.14, 129.94, 129.27, 129.13, 129.09, 128.77, 127.96, 126.86, 47.99, 17.76, 7.39, 3.58. (*see spectrum*)

HRMS (ESI) (m/z): $[M+H]^+$ calculated for $C_{22}H_{30}N_2Si^+$: 349.2095, found: 349.2090.

2-(2-(methyldiphenylsilyl)-1-phenylethyl)quinoxaline (d-94)

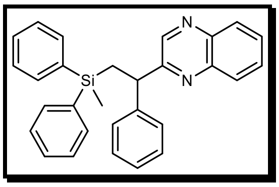

Following the **General procedure D**, **a-2** (40.8 mg, 0.2 mmol), **b-25** (89.4 mg, 0.4 mmol) and **c-81** (39.0 mg, 0.3 mmol) were used. Purification by column chromatography using silica with pentane/EtOAc (20:1 to 10:1, v/v) as eluent afforded **d-94** (55.9 mg, 65% yield) as a colorless oil.

^{1}H NMR (400 MHz, Chloroform-*d*) δ 8.52 (s, 1H), 8.04 (ddd, *J* = 25.3, 8.2, 1.7 Hz, 2H), 7.73 (dtd, *J* = 13.5, 7.0, 5.4 Hz, 2H), 7.51 – 7.42 (m, 4H), 7.35 (dd, *J* = 20.7, 6.3 Hz, 4H), 7.28 (dd, *J* = 13.2, 5.6 Hz, 5H), 7.21 (dd, *J* = 13.9, 6.7 Hz, 2H), 4.51 (dd, *J* = 9.3, 6.1 Hz, 1H), 2.55 (dd, *J* = 14.8, 9.3 Hz, 1H), 2.06 (dd, *J* = 14.8, 6.1 Hz, 1H), 0.35 (s, 3H). (*see spectrum*)

^{13}**C NMR** (101 MHz, Chloroform-*d*) δ 159.04, 145.93, 144.34, 141.84, 141.02, 136.95, 136.43, 134.47, 134.45, 129.79, 129.27, 129.21, 129.10, 129.01, 128.79, 127.99, 127.85, 127.75, 126.91, 47.72, 20.64, -3.93. (*see spectrum*)

HRMS (ESI) (m/z): $[M+H]^+$ calculated for $C_{29}H_{27}N_2Si^+$:431.1938, found: 431.1933.

2-(2-cyclohexyl-1-phenylethyl)quinoxaline (d-95)

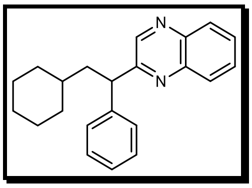

Following the **General procedure D**, **a-2** (40.8 mg, 0.2 mmol), **b-32** (66.1 mg, 0.6 mmol) and **c-81** (39.0 mg, 0.3 mmol) were used. Purification by column chromatography using silica with pentane/EtOAc (20:1 to 10:1, v/v) as eluent afforded **d-95** (32.9 mg, 52% yield) as a colorless oil.

^{1}**H NMR** (400 MHz, Chloroform-*d*) δ 8.72 (s, 1H), 8.09 (ddd, *J* = 29.2, 8.2, 1.6 Hz, 2H), 7.72 (dddd, *J* = 20.4, 8.4, 6.9, 1.6 Hz, 2H), 7.40 (d, *J* = 7.3 Hz, 2H), 7.30 (t, *J* = 7.5 Hz, 2H), 7.20 (t, *J* = 7.3 Hz, 1H), 4.47 (t, *J* = 7.8 Hz, 1H), 2.30 (dt, *J* = 14.4, 7.4 Hz, 1H), 2.11 (ddd, *J* = 14.0, 7.9, 6.4 Hz, 1H), 1.85 – 1.77 (m, 2H), 1.73 – 1.55 (m, 3H), 1.25 – 0.90 (m, 6H). (*see spectrum*)

^{13}**C NMR** (101 MHz, Chloroform-*d*) δ 159.03, 145.96, 142.60, 142.11, 141.11, 129.96, 129.33, 129.22, 129.07, 128.81, 128.27, 126.86, 76.78, 49.01, 42.21, 35.13, 33.54, 33.32, 26.60, 26.21, 26.18. (*see spectrum*)

HRMS (ESI) (m/z): $[M+H]^+$ calculated for $C_{22}H_{25}N_2^+$: 317.2012, found: 317.2017.

2-(1-phenyloctyl)quinoxaline (d-96)

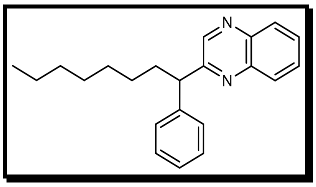

Following the **General procedure D**, **a-2** (40.8 mg, 0.2 mmol), **b-36** (67.3 mg, 0.6 mmol) and **c-81** (39.0 mg, 0.3 mmol) were used. Purification by column chromatography using silica with pentane/EtOAc (20:1 to 3:1, v/v) as eluent afforded **d-96** (31.8 mg, 50% yield) as a colorless oil.

^{1}H NMR (400 MHz, Chloroform-*d*) δ 8.70 (s, 1H), 8.08 (ddd, *J* = 28.3, 8.2, 1.6 Hz, 2H), 7.72 (dddd, *J* = 20.5, 8.4, 6.9, 1.6 Hz, 2H), 7.39 (d, *J* = 7.3 Hz, 2H), 7.30 (t, *J* = 7.6 Hz, 2H), 7.21 (t, *J* = 7.3 Hz, 1H), 4.29 (t, *J* = 7.7 Hz, 1H), 2.46 – 2.33 (m, 1H), 2.27 – 2.15 (m, 1H), 1.36 – 1.19 (m, 10H), 0.85 (t, *J* = 6.8 Hz, 3H). (see spectrum)

^{13}C NMR (101 MHz, Chloroform-*d*) δ 158.93, 146.03, 142.49, 142.15, 141.23, 129.91, 129.35, 129.16, 129.14, 128.78, 128.26, 126.88, 52.20, 34.57, 31.86, 29.60, 29.19, 27.92, 22.69, 14.15. (see spectrum)

HRMS (ESI) (m/z): $[M+H]^+$ calculated for $C_{22}H_{27}N_2^+$: 319.2169, found: 319.2173.

2-(1,4-diphenylbutyl)quinoxaline (d-97)

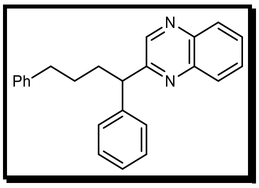

Following the **General procedure D**, **a-2** (40.8 mg, 0.2 mmol), **b-39** (79.2 mg, 0.6 mmol) and **c-81** (39.0 mg, 0.3 mmol) were used. Purification by column chromatography using silica with pentane/EtOAc (20:1 to 10:1, v/v) as eluent afforded **d-97** (28.4 mg, 42% yield) as a colorless oil.

^{1}H NMR (400 MHz, Chloroform-*d*) δ 8.66 (s, 1H), 8.07 (ddd, *J* = 26.6, 8.2, 1.6 Hz, 2H), 7.78 – 7.66 (m, 2H), 7.36 (d, *J* = 7.3 Hz, 2H), 7.31 – 7.11 (m, 8H), 4.31 (t, *J* = 7.7 Hz, 1H), 2.68 (t, *J* = 7.7 Hz, 2H), 2.51 – 2.38 (m, 1H), 2.33 – 2.21 (m, 1H), 1.66 (qq, *J* = 9.6, 6.6, 4.7 Hz, 2H). (see spectrum)

^{13}C NMR (101 MHz, Chloroform-*d*) δ 158.63, 146.00, 142.24, 142.12, 141.23, 129.98, 129.35, 129.25, 129.15, 128.85, 128.46, 128.37, 128.27, 127.00, 125.84, 52.04, 35.90, 34.14, 29.67. (see spectrum)

HRMS (ESI) (m/z): $[M+H]^+$ calculated for $C_{24}H_{23}N_2^+$: 339.1856, found: 339.1853.

2-(2-(dimethyl(phenyl)silyl)-1-phenylethyl)-3-methylquinoxaline (d-98)

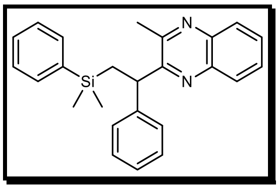

Following the **General procedure D**, **a-2** (40.8 mg, 0.2 mmol), **b-1** (64.9 mg, 0.4 mmol) and **c-98** (43.2 mg, 0.3 mmol) were used. Purification by column chromatography using silica with pentane/EtOAc (20:1 to 10:1, v/v) as eluent afforded **d-98** (32.1 mg, 42% yield) as a pink oil.

^{1}H NMR (400 MHz, Chloroform-*d*) δ 8.08 (d, *J* = 9.6 Hz, 1H), 7.95 (d, *J* = 8.8 Hz, 1H), 7.68 (tt, *J* = 7.1, 5.2 Hz, 2H), 7.32 (d, *J* = 7.0 Hz, 2H), 7.28 (d, *J* = 7.0 Hz, 1H), 7.24 – 7.19 (m, 6H), 7.16 – 7.11 (m, 1H), 4.37 (dd, *J* = 9.8, 5.5 Hz, 1H), 2.44 (s, 3H), 2.28 (dd, *J* = 14.6, 9.9 Hz, 1H), 1.59 (dd, *J* = 14.6, 5.5 Hz, 1H), 0.13 (s, 3H), 0.07 (s, 3H). (*see spectrum*)

^{13}C NMR (101 MHz, Chloroform-*d*) δ 157.89, 153.09, 144.30, 140.99, 138.90, 133.49, 129.14, 129.07, 128.86, 128.80, 128.63, 128.12, 128.04, 127.70, 126.59, 46.04, 23.96, 22.73, -2.26, -2.31. (*see spectrum*)

HRMS (ESI) (m/z): $[M+H]^+$ calculated for $C_{25}H_{27}N_2Si^+$: 383.1938, found: 383.1940.

(3S,8S,9S,10R,13R,14S,17R)-10,13-dimethyl-17-((R)-6-methylheptan-2-yl)-2,3,4,7,8,9,10,11,12,13,14,15,16,17-tetradecahydro-1H-cyclopenta[a]phenanthren-3-yl 4-(2-(dimethyl(phenyl)silyl)-1-(quinoxalin-2-yl)ethyl)benzoate (d-99)

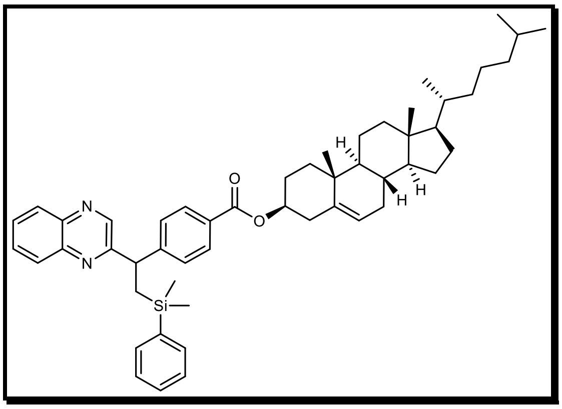

Following the **General procedure D**, **a-70** (123.3 mg, 0.2 mmol), **b-1** (64.9 mg, 0.4 mmol) and **c-81** (39.0 mg, 0.3 mmol) were used. Purification by column chromatography using silica with pentane/EtOAc (20:1 to 5:1, v/v) as eluent afforded **d-99** (81.2 mg, 52% yield) as a colorless oil.

^{1}H NMR (400 MHz, Chloroform-*d*) δ 8.54 (s, 1H), 8.02 (ddd, *J* = 23.6, 8.2, 1.7 Hz, 2H), 7.92 (d, *J* = 8.1 Hz, 2H), 7.75 – 7.65 (m, 2H), 7.41 (d, *J* = 8.1 Hz, 2H), 7.37 – 7.31

(m, 2H), 7.27 – 7.18 (m, 3H), 5.39 (d, *J* = 5.0 Hz, 1H), 4.82 (dtd, *J* = 12.0, 8.3, 4.4 Hz, 1H), 4.44 (t, *J* = 7.8 Hz, 1H), 2.42 (d, *J* = 8.2 Hz, 2H), 2.09 (dd, *J* = 14.7, 8.5 Hz, 1H), 2.04 – 1.93 (m, 3H), 1.91 – 1.77 (m, 2H), 1.76 – 1.64 (m, 2H), 1.61 – 1.42 (m, 6H), 1.35 (dd, *J* = 13.9, 7.3 Hz, 3H), 1.25 (t, *J* = 7.1 Hz, 1H), 1.13 (ddt, *J* = 18.9, 13.5, 7.8 Hz, 6H), 1.05 – 0.95 (m, 6H), 0.91 (d, *J* = 6.5 Hz, 3H), 0.88 – 0.83 (m, 6H), 0.67 (s, 3H), 0.12 (d, *J* = 9.0 Hz, 6H). (see spectrum)

^{13}C NMR (101 MHz, Chloroform-*d*) δ 165.79, 158.64, 149.20, 145.62, 141.93, 141.21, 139.71, 138.27, 133.51, 130.05, 130.01, 129.45, 129.31, 129.25, 129.12, 128.95, 127.97, 127.77, 122.84, 74.58, 56.75, 56.19, 50.09, 47.98, 42.38, 39.79, 39.59, 38.26, 37.08, 36.70, 36.25, 35.87, 31.99, 31.93, 28.31, 28.09, 27.93, 24.36, 23.90, 22.91, 22.65, 22.32, 21.11, 19.45, 18.80, 11.93, -2.26, -2.73. (see spectrum)

HRMS (ESI) (m/z): $[M+H]^+$ calculated for $C_{52}H_{69}N_2O_2Si^+$: 781.5123, found: 781.5125.

((3aS,8aR,8bS)-2,2,7,7-tetramethyltetrahydro-3aH-bis([1,3]dioxolo)[4,5-b:4',5'-d]pyran-3a-yl)methyl 4-(2-(dimethyl(phenyl)silyl)-1-(quinoxalin-2-yl)ethyl)benzoate (d-100)

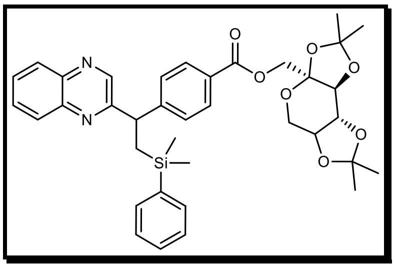

Following the **General procedure D**, **a-61** (98.1 mg, 0.2 mmol), **b-1** (64.9 mg, 0.4 mmol) and **c-81** (39.0 mg, 0.3 mmol) were used. Purification by column chromatography using silica with pentane/EtOAc (20:1 to 5:1, v/v) as eluent afforded **d-100** (73.2 mg, 56% yield) as a white solid.

^{1}H NMR (400 MHz, Chloroform-*d*) δ 8.56 (s, 1H), 8.03 (ddd, *J* = 24.2, 8.2, 1.6 Hz, 2H), 7.94 (d, *J* = 8.1 Hz, 2H), 7.78 – 7.66 (m, 2H), 7.43 (d, *J* = 8.2 Hz, 2H), 7.39 – 7.32 (m, 2H), 7.28 – 7.19 (m, 3H), 5.56 (d, *J* = 5.0 Hz, 1H), 4.64 (dd, *J* = 7.9, 2.4 Hz, 1H), 4.53 – 4.43 (m, 2H), 4.40 (dd, *J* = 11.7, 7.4 Hz, 1H), 4.32 (ddd, *J* = 14.6, 6.5, 2.2 Hz, 2H), 4.15 (ddd, *J* = 7.3, 5.1, 1.9 Hz, 1H), 2.14 – 2.04 (m, 1H), 1.74 (dd, *J* = 14.7, 7.1

Hz, 1H), 1.48 (d, *J* = 9.2 Hz, 6H), 1.33 (d, *J* = 9.5 Hz, 6H), 0.13 (d, *J* = 10.0 Hz, 6H). (*see spectrum*)

^{13}C NMR (101 MHz, Chloroform-*d*) δ 166.39, 158.72, 149.68, 145.76, 142.09, 141.36, 138.39, 133.67, 130.38, 130.20, 129.52, 129.42, 129.26, 129.13, 128.81, 128.21, 127.93, 109.91, 109.03, 96.54, 71.34, 70.94, 70.74, 66.35, 64.04, 48.14, 26.25, 26.20, 25.21, 24.71, 22.51, -2.13, -2.58. (*see spectrum*)

HRMS (ESI) (m/z): $[M+H]^+$ calculated for $C_{37}H_{43}N_2O_7Si^+$: 655.2834, found: 655.2841.

3. NMR Spectra for the Products

^{1}H NMR spectra of compound **a-59** in $CDCl_3$ (400 MHz): (*see procedure*)

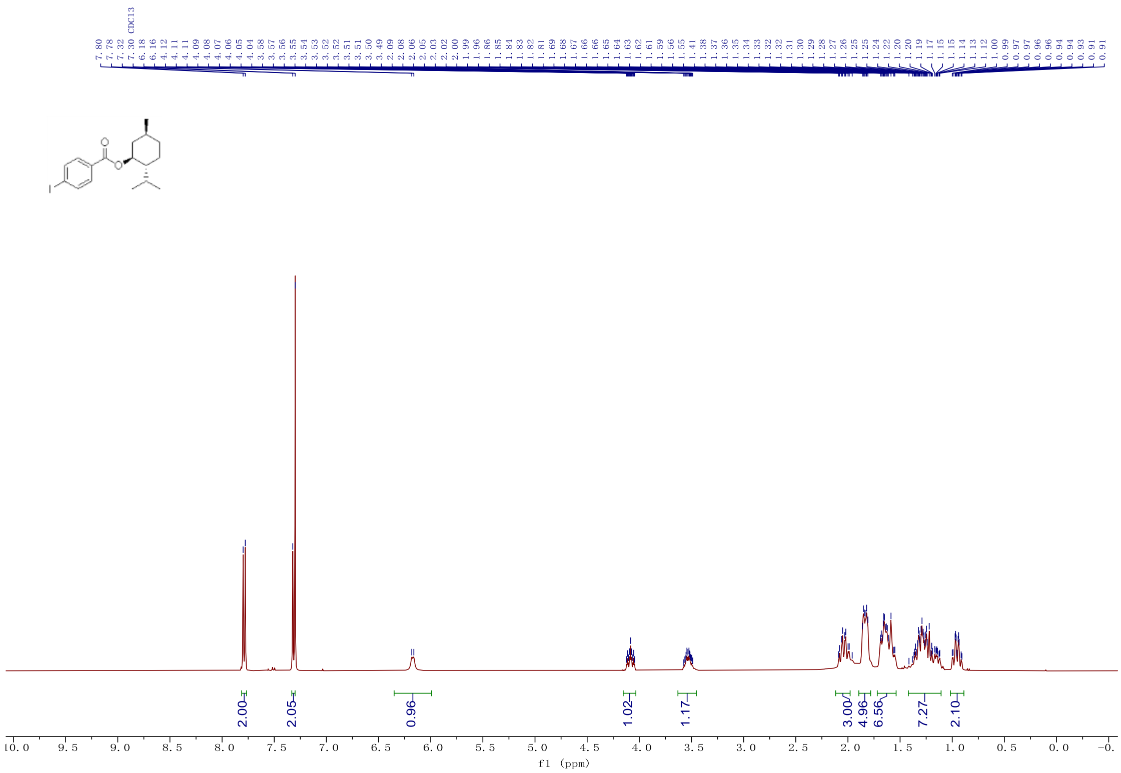

^{13}C NMR spectra of compound **a-59** in $CDCl_3$ (101 MHz): (*see procedure*)

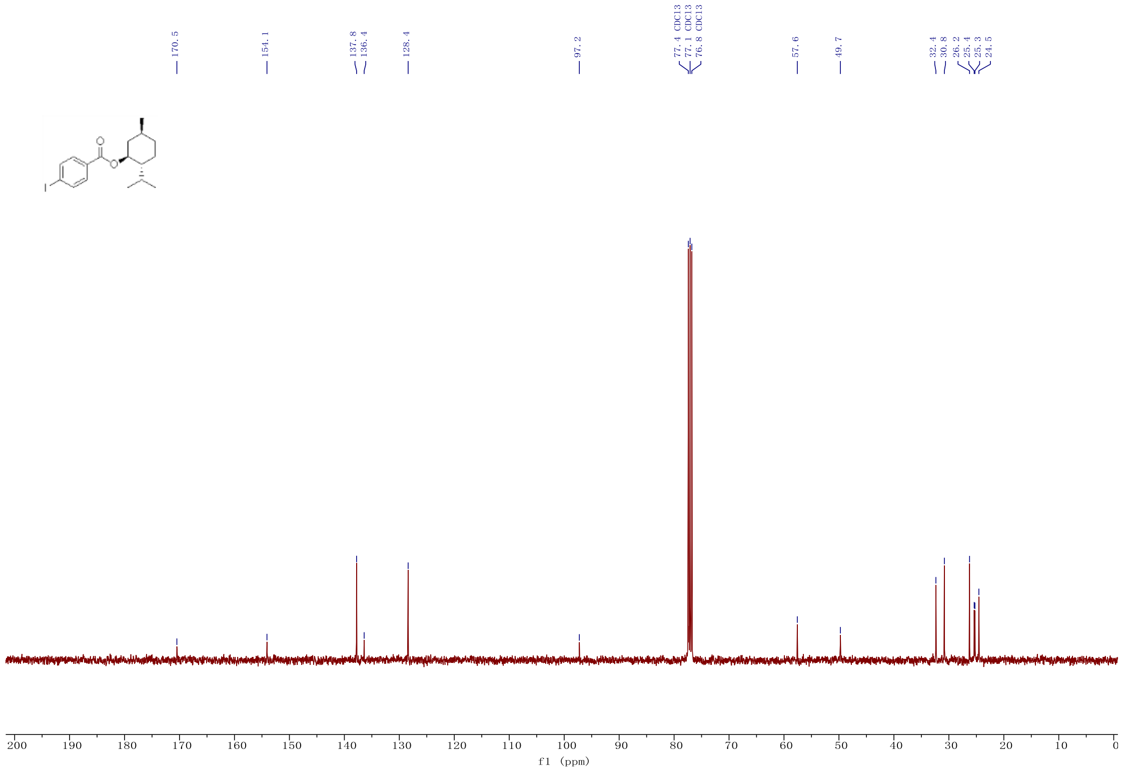

^{1}H NMR spectra of compound **a-60** in $CDCl_3$ (400 MHz): (*see procedure*)

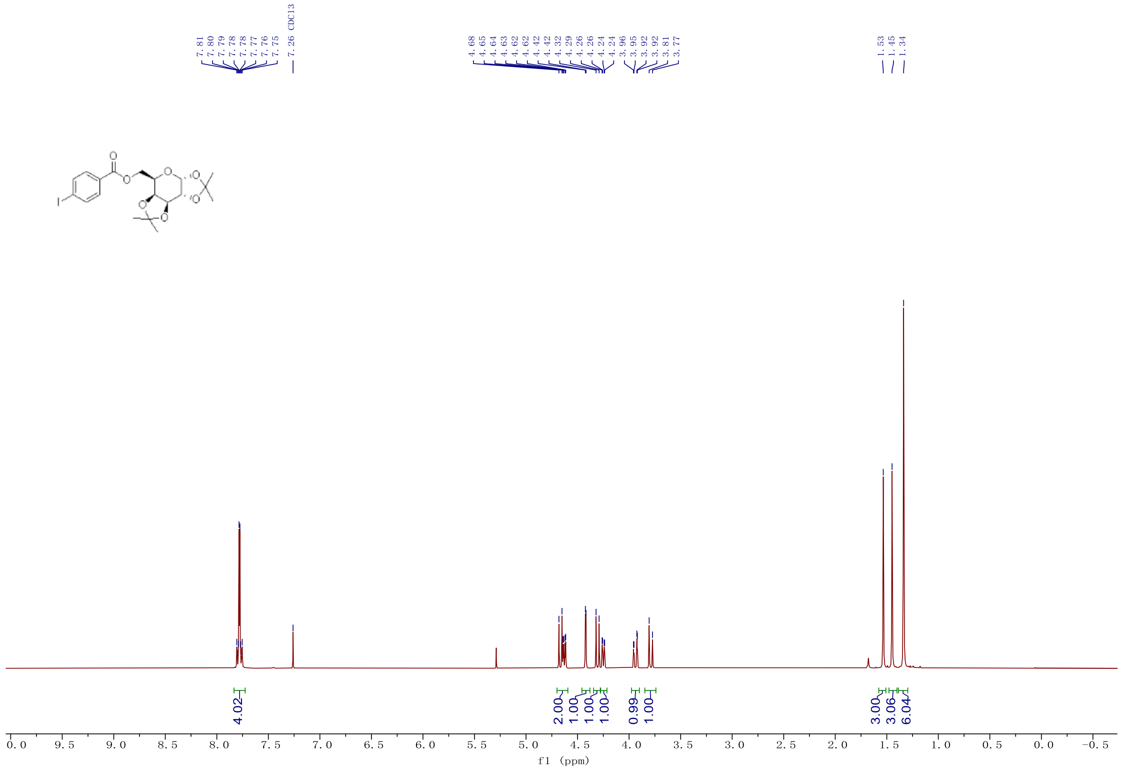

^{13}C NMR spectra of compound **a-60** in $CDCl_3$ (101 MHz): (*see procedure*)

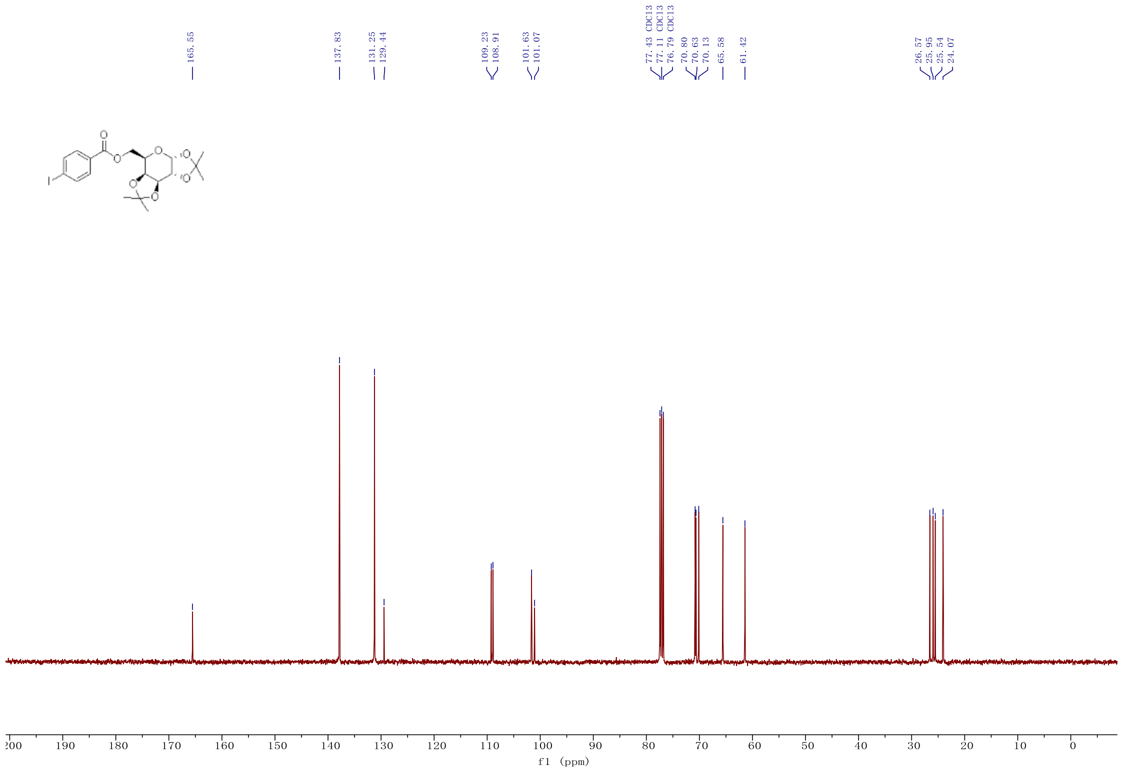

^{1}H NMR spectra of compound **a-61** in $CDCl_3$ (400 MHz): (*see procedure*)

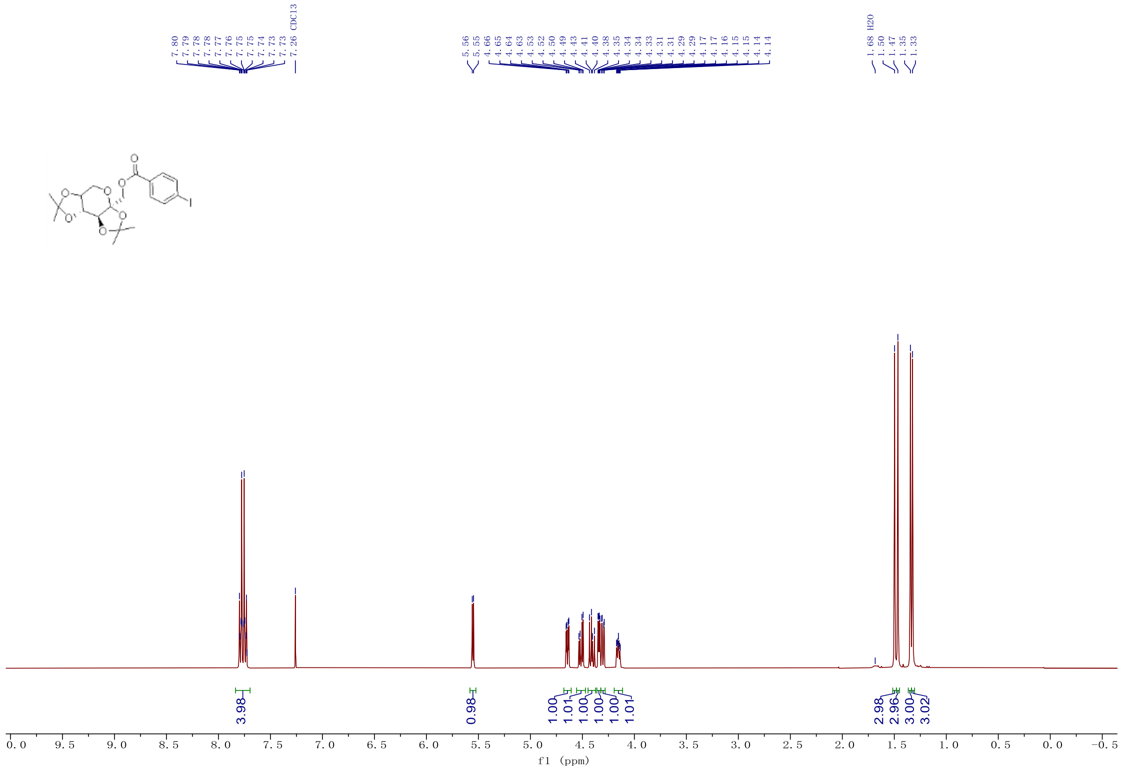

^{13}C NMR spectra of compound **a-61** in $CDCl_3$ (101 MHz): (*see procedure*)

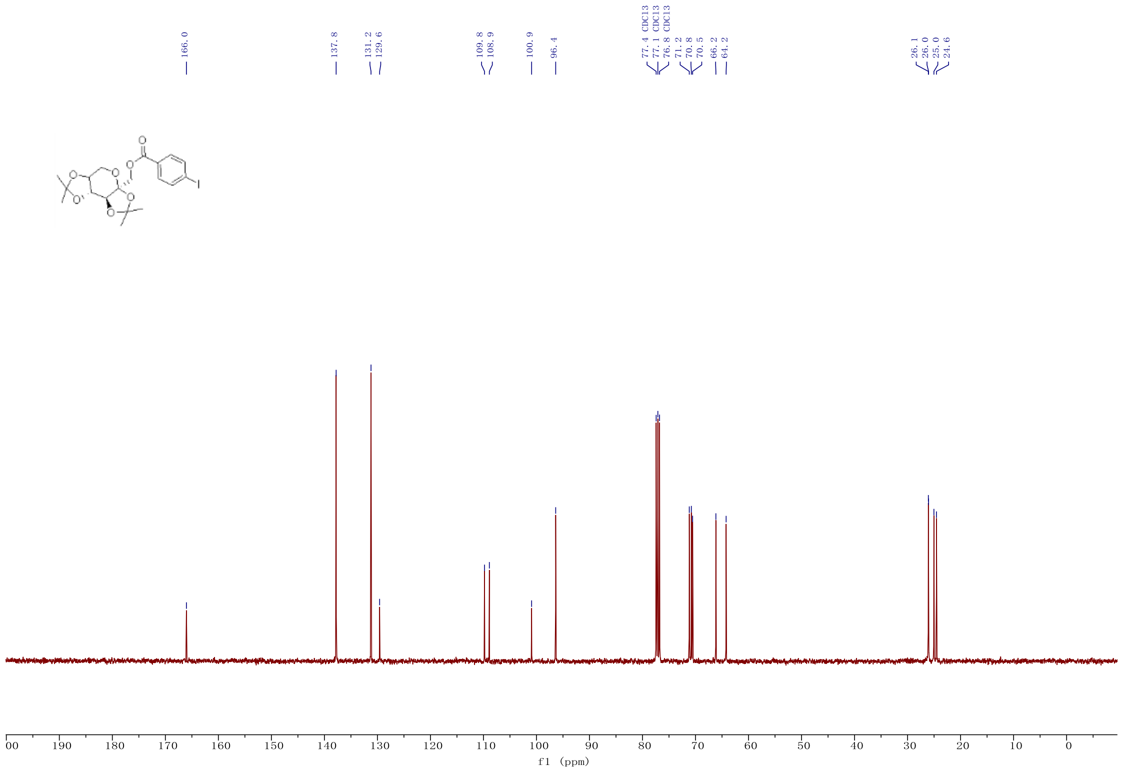

^{1}H NMR spectra of compound **a-62** in $CDCl_3$ (400 MHz): (*see procedure*)

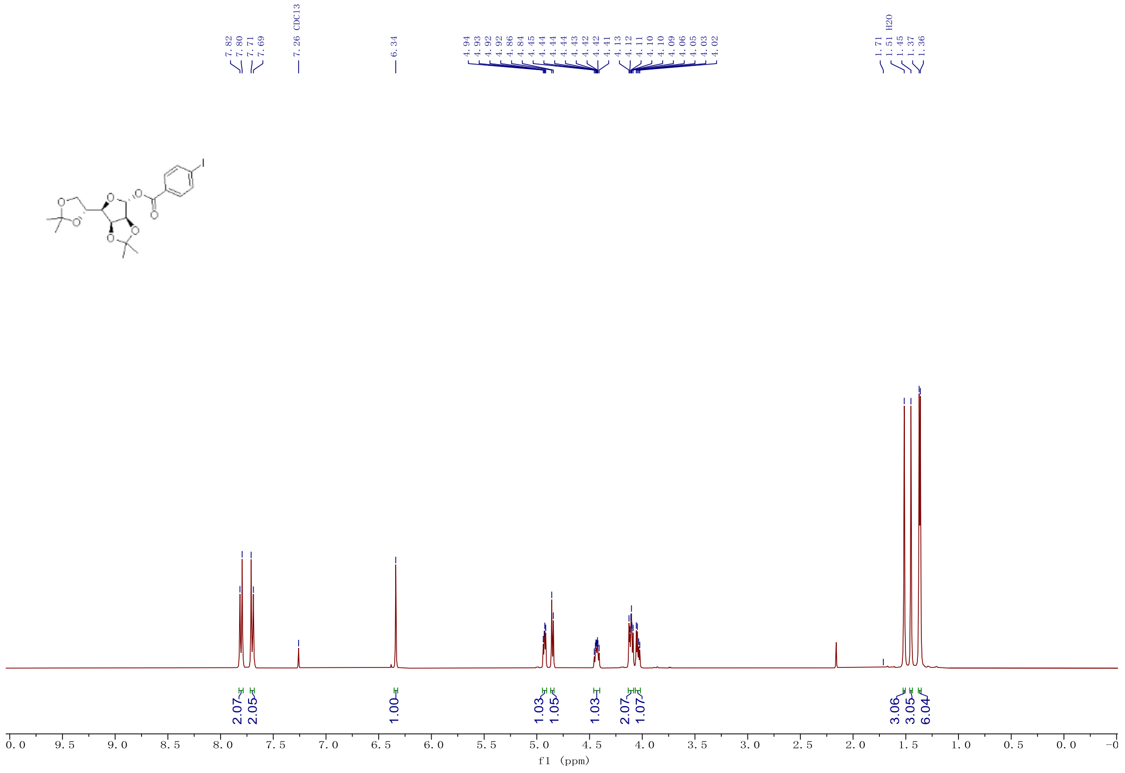

^{13}C NMR spectra of compound **a-62** in $CDCl_3$ (101 MHz): (*see procedure*)

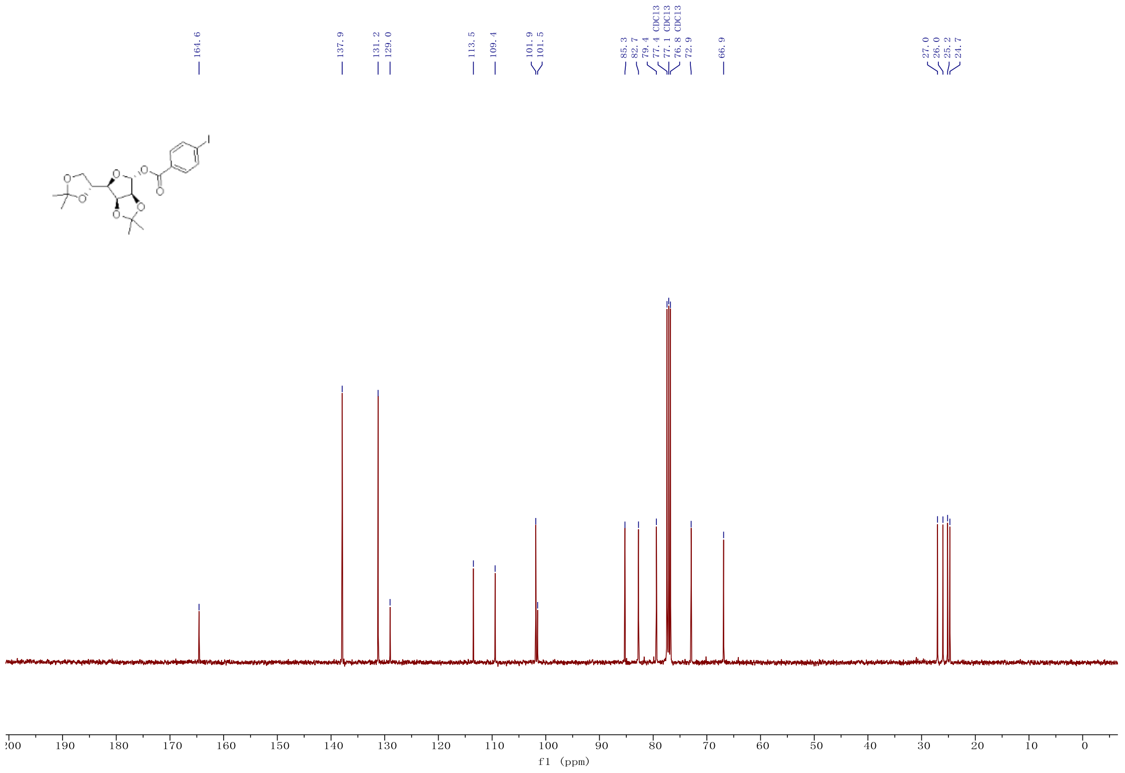

^{1}H NMR spectra of compound **a-63** in $CDCl_3$ (400 MHz): (*see procedure*)

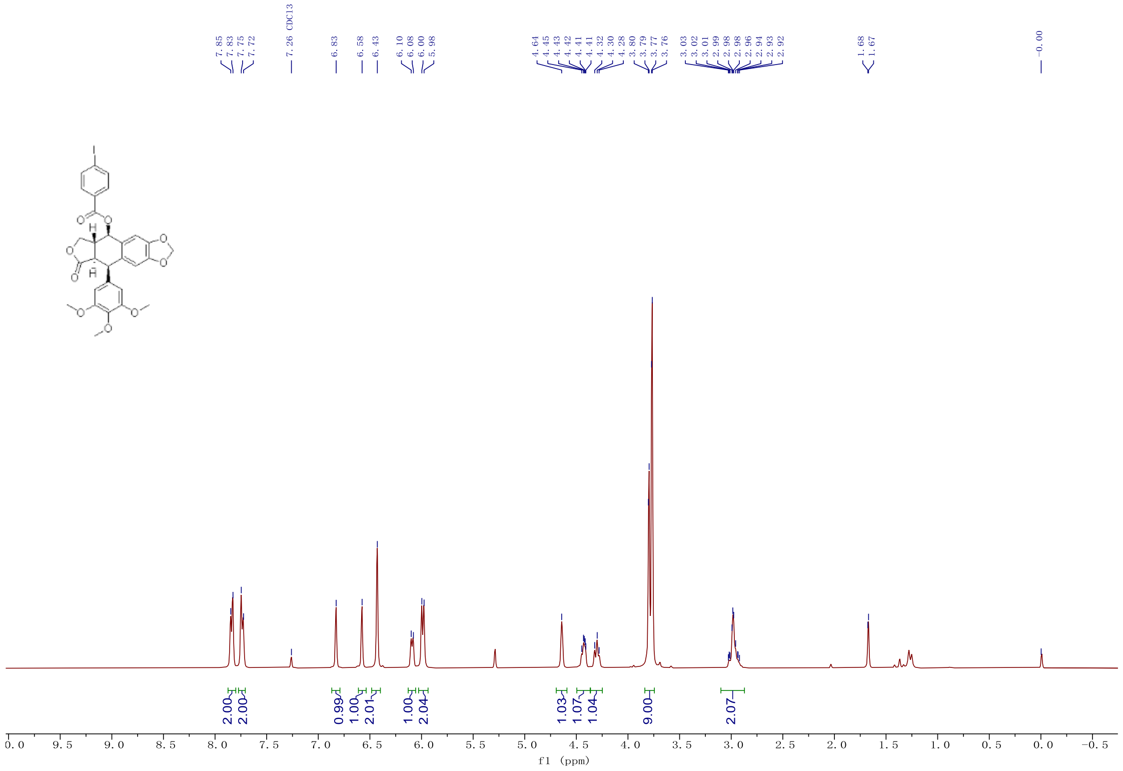

^{13}C NMR spectra of compound **a-63** in $CDCl_3$ (101 MHz): (*see procedure*)

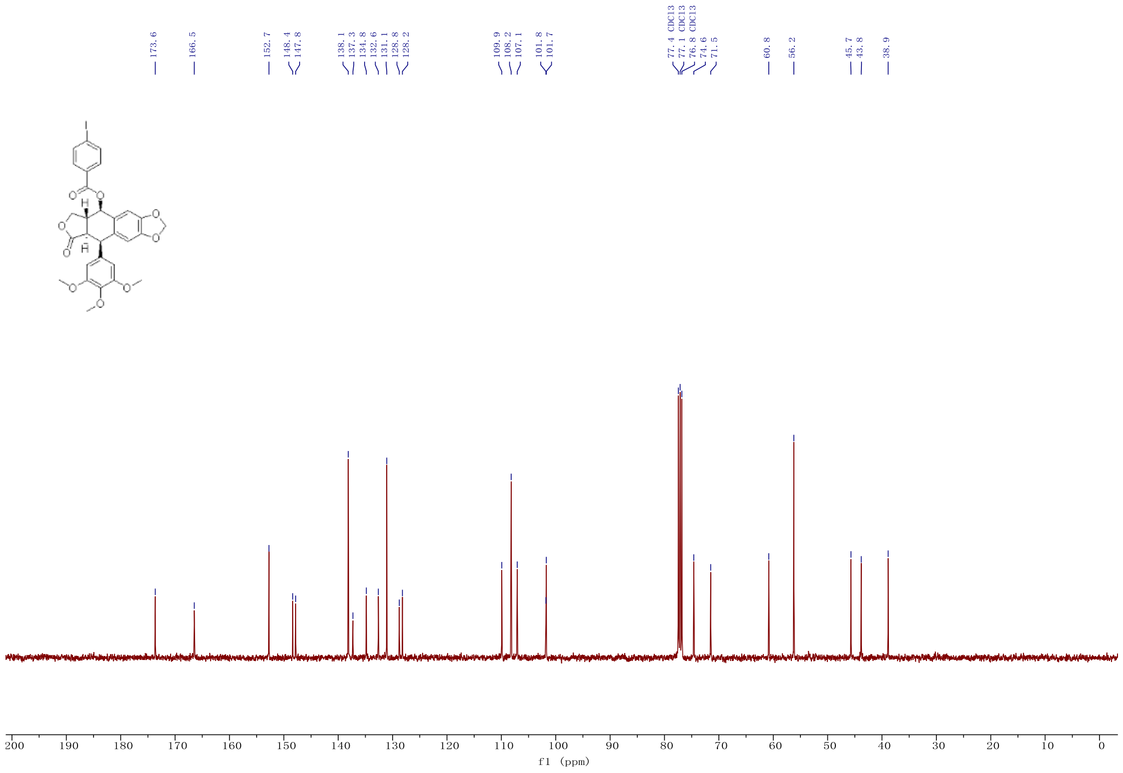

^{1}H NMR spectra of compound **a-64** in $CDCl_3$ (400 MHz): (*see procedure*)

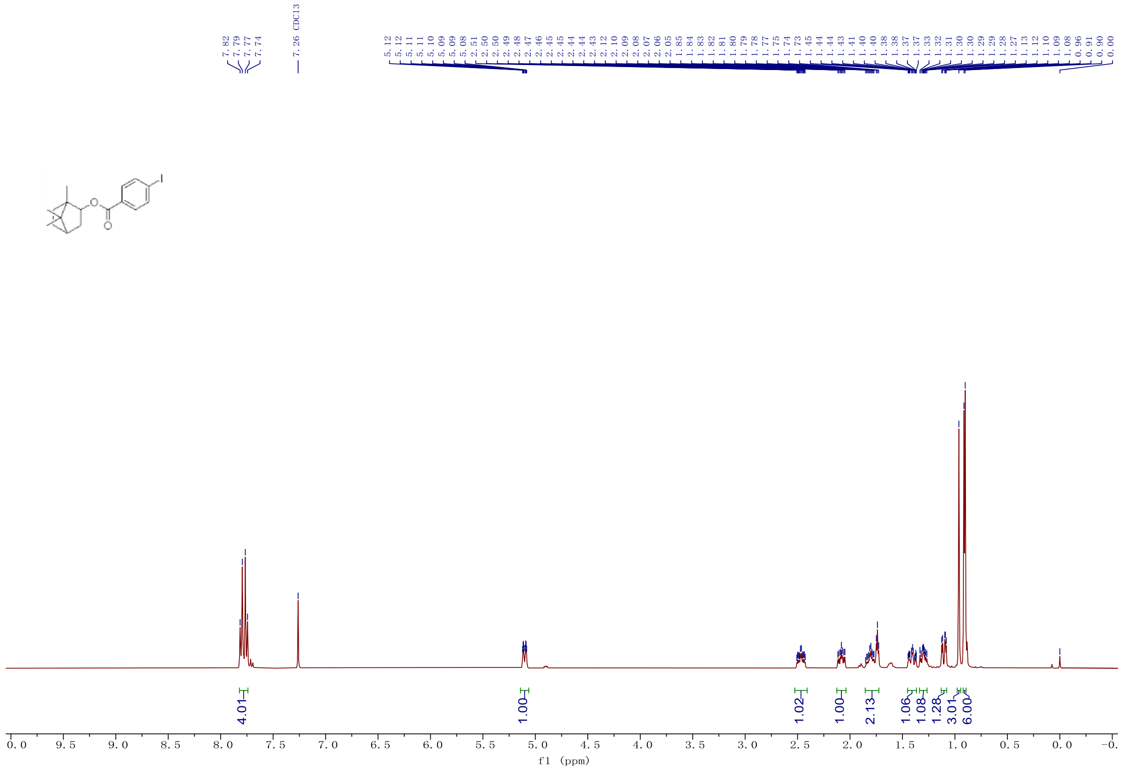

^{13}C NMR spectra of compound **a-64** in $CDCl_3$ (101 MHz): (*see procedure*)

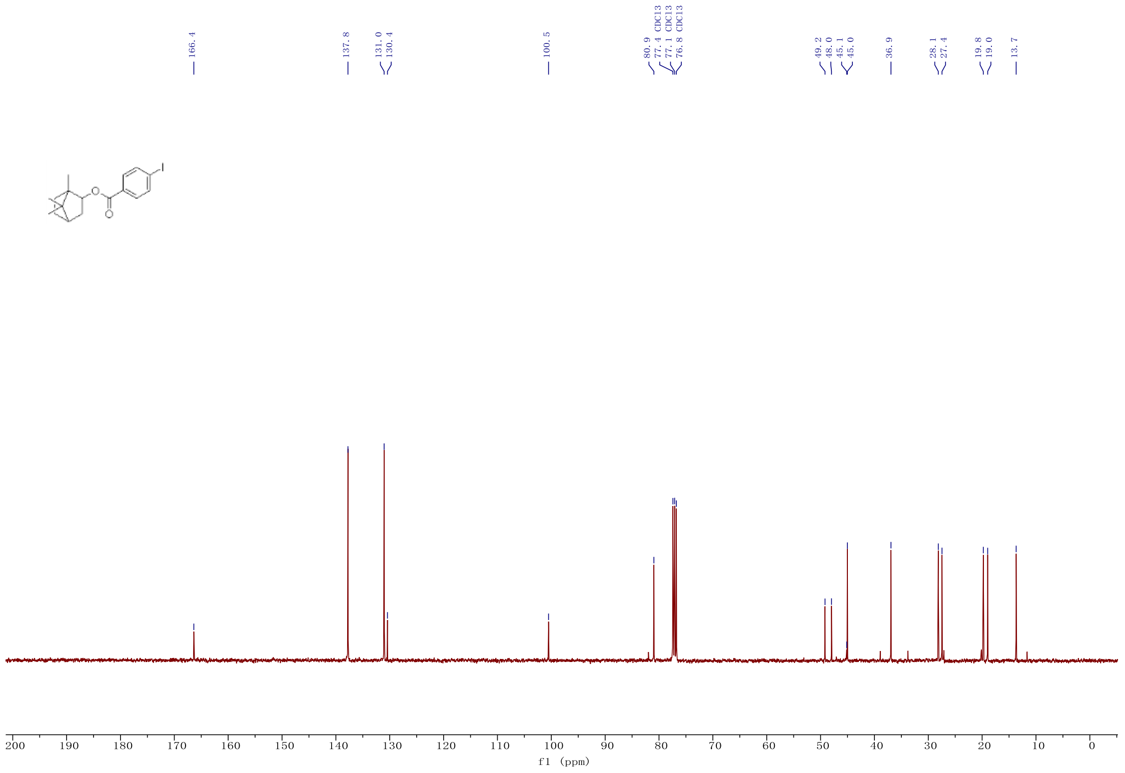

^{1}H NMR spectra of compound **a-65** in $CDCl_3$ (400 MHz): (*see procedure*)

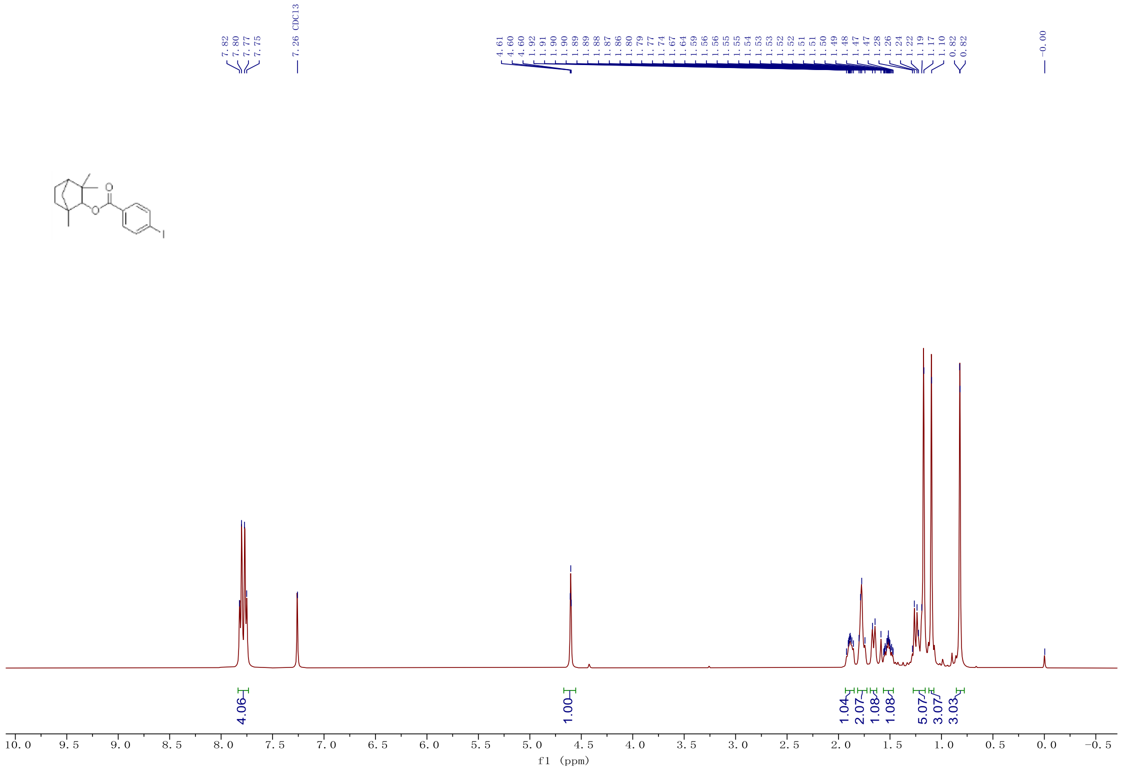

^{13}C NMR spectra of compound **a-65** in $CDCl_3$ (101 MHz): (*see procedure*)

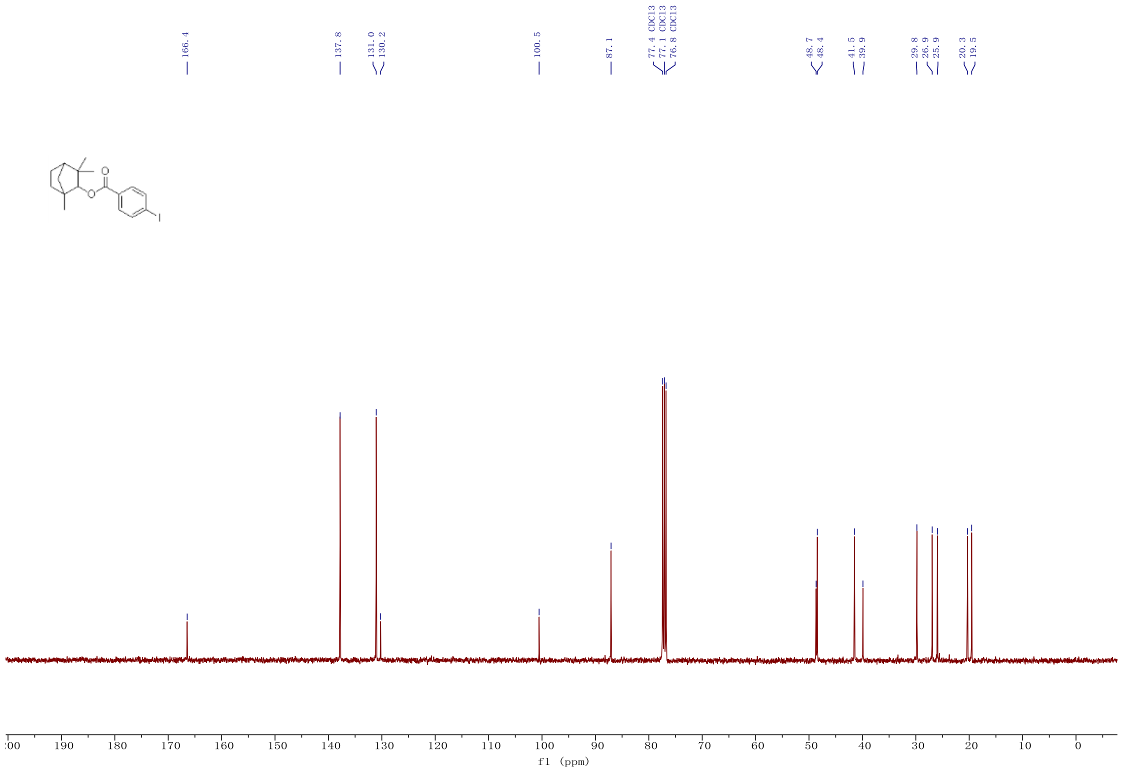

^{1}H NMR spectra of compound **a-66** in $CDCl_3$ (400 MHz): (*see procedure*)

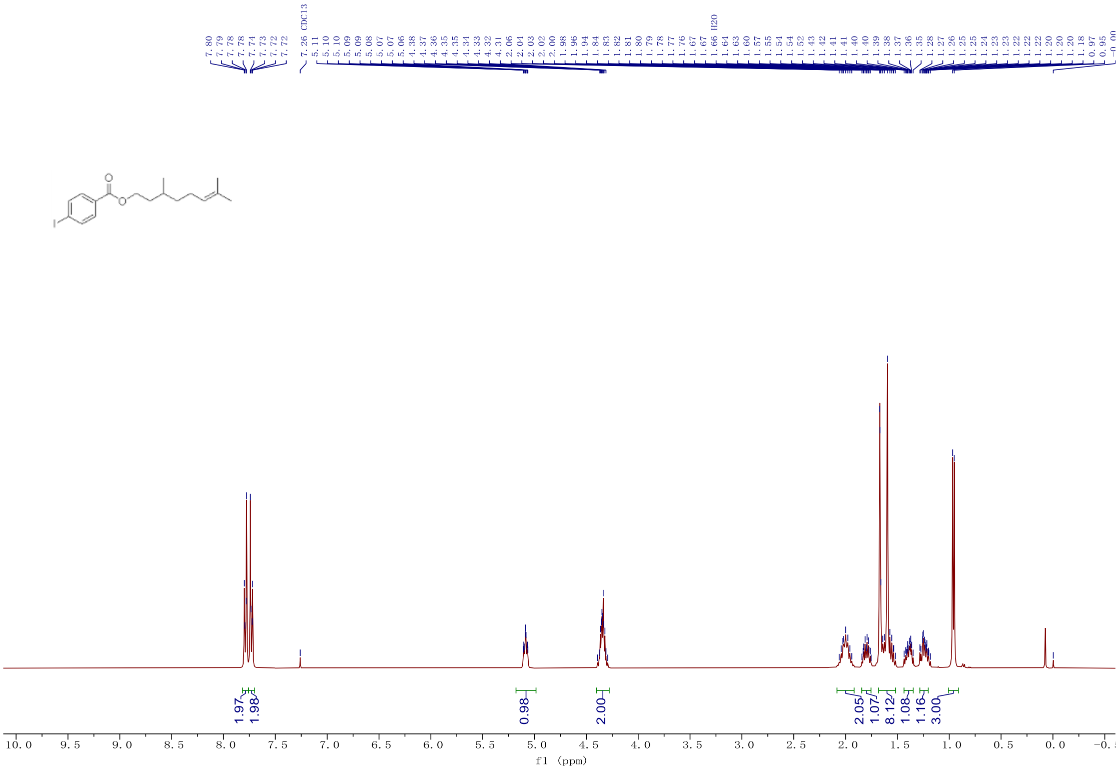

^{13}C NMR spectra of compound **a-66** in $CDCl_3$ (101 MHz): (*see procedure*)

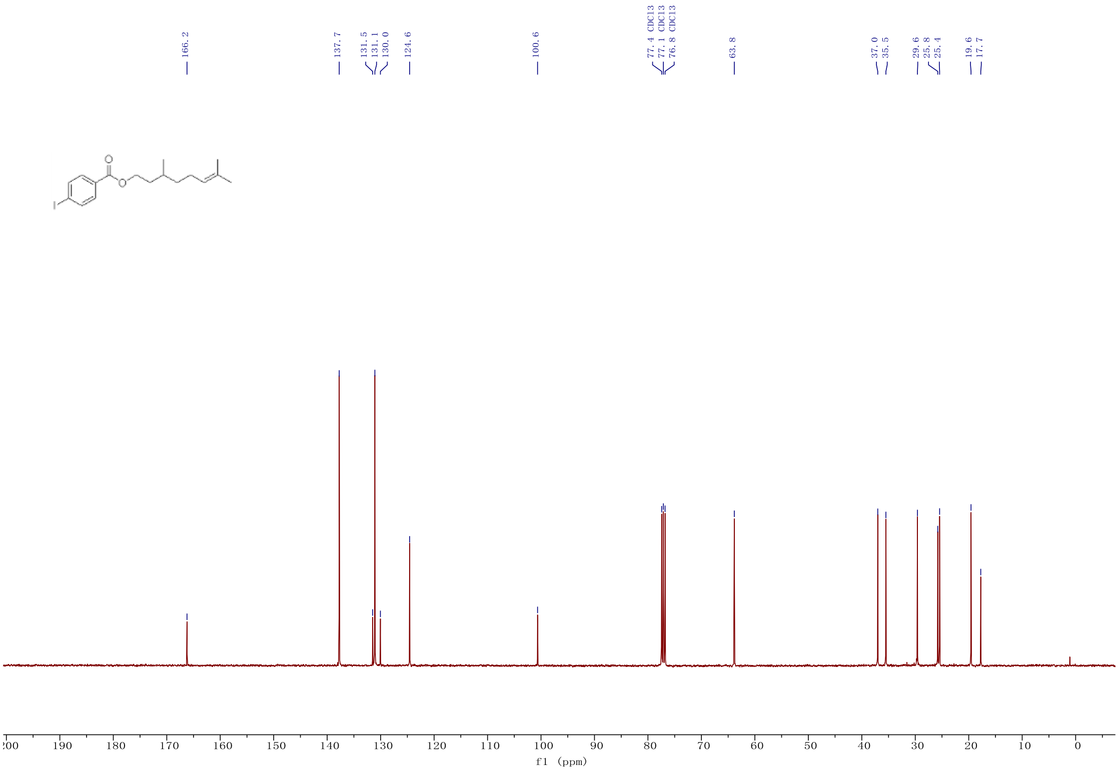

^{1}H NMR spectra of compound **a-67** in $CDCl_3$ (400 MHz): (<u>*see procedure*</u>)

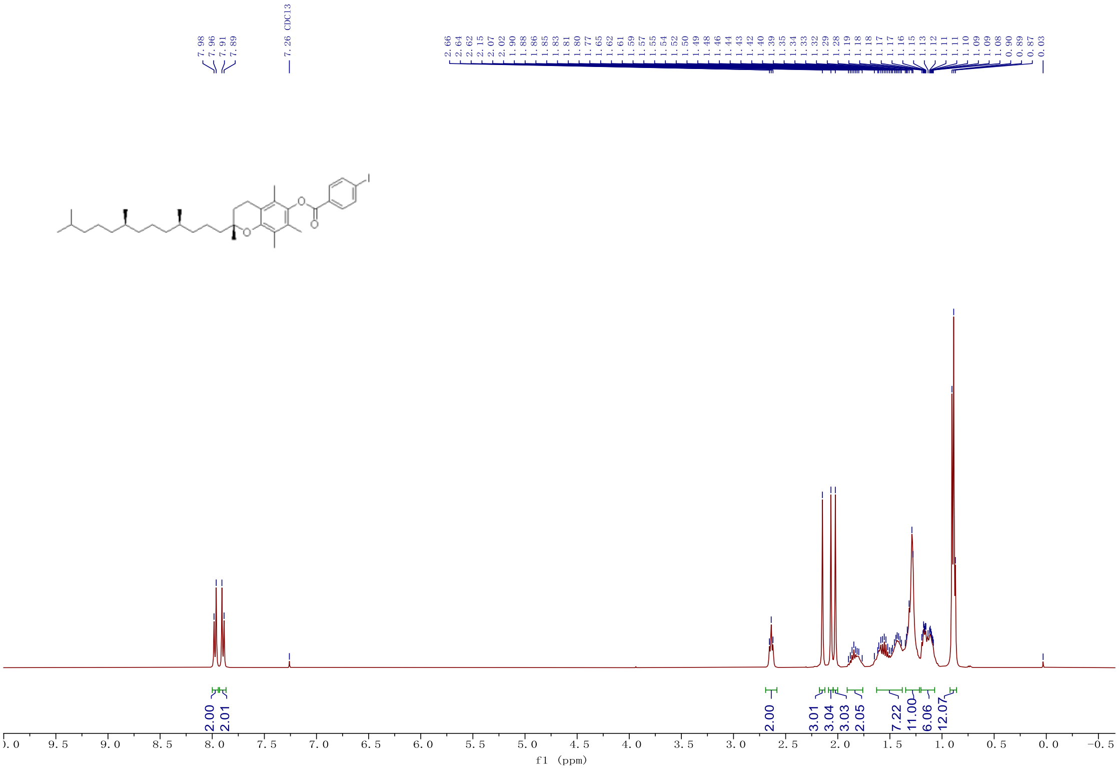

^{13}C NMR spectra of compound **a-67** in $CDCl_3$ (101 MHz): (<u>*see procedure*</u>)

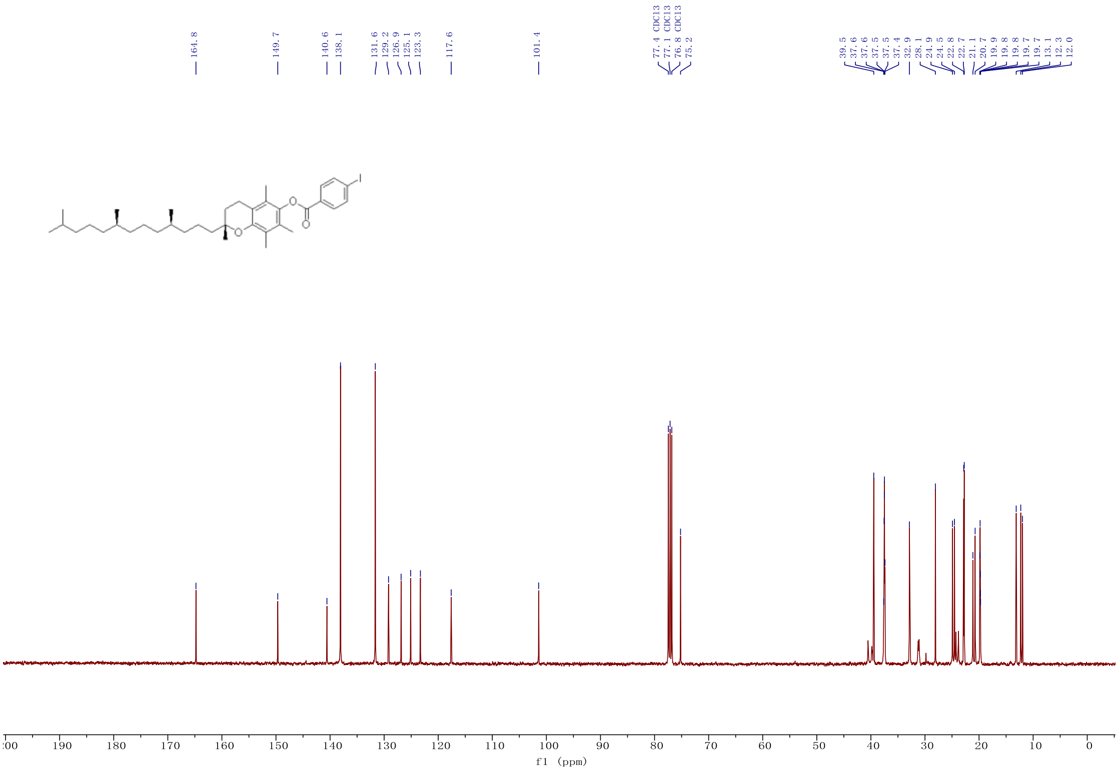

^{1}H NMR spectra of compound **a-68** in $CDCl_3$ (400 MHz): (*see procedure*)

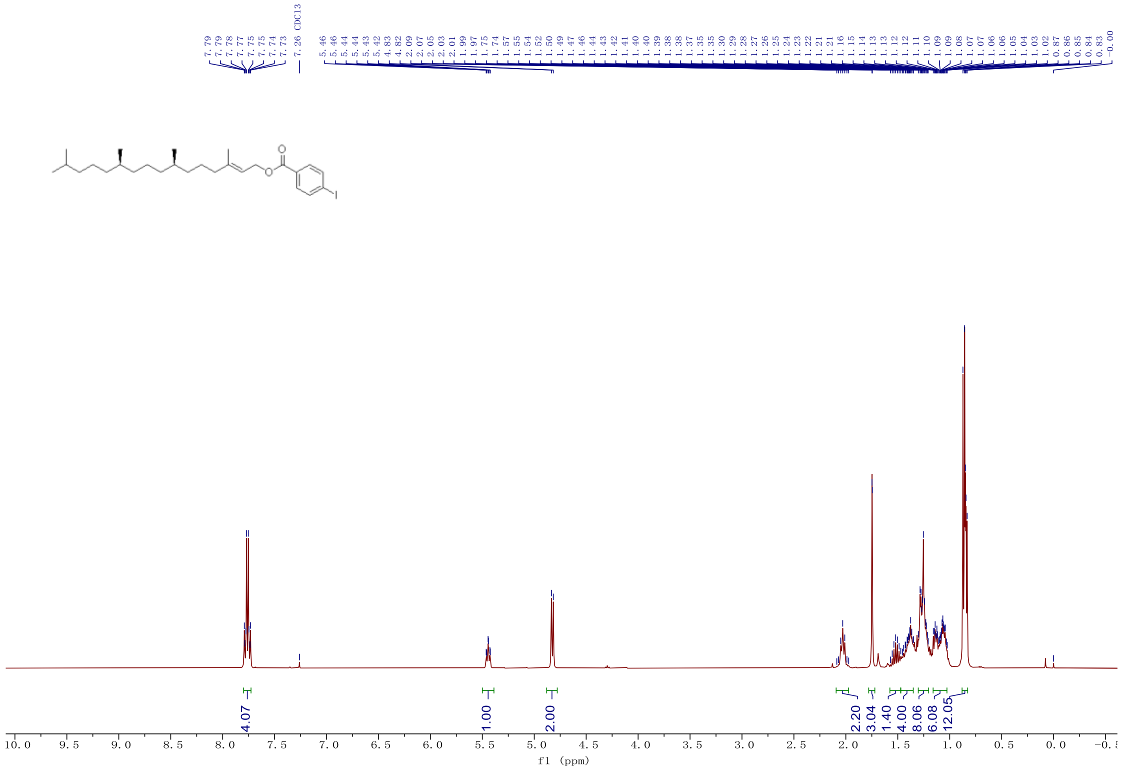

^{13}C NMR spectra of compound **a-68** in $CDCl_3$ (101 MHz): (*see procedure*)

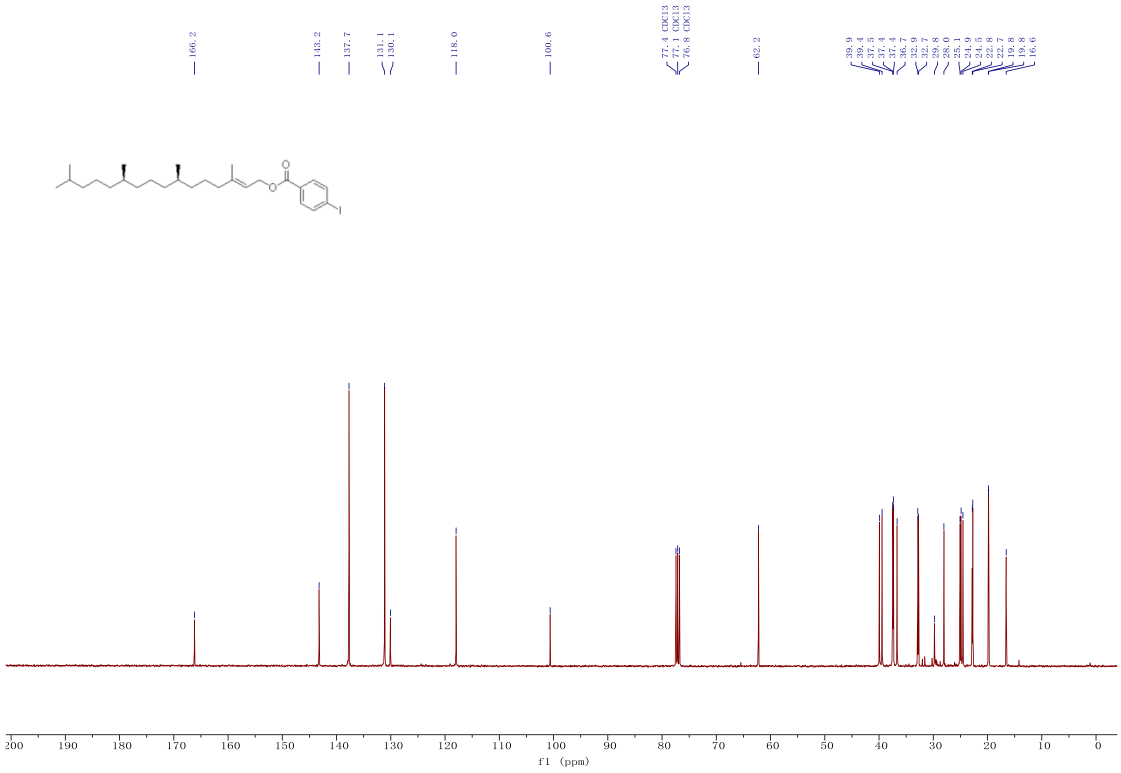

^{1}H NMR spectra of compound **a-69** in $CDCl_3$ (400 MHz): (*see procedure*)

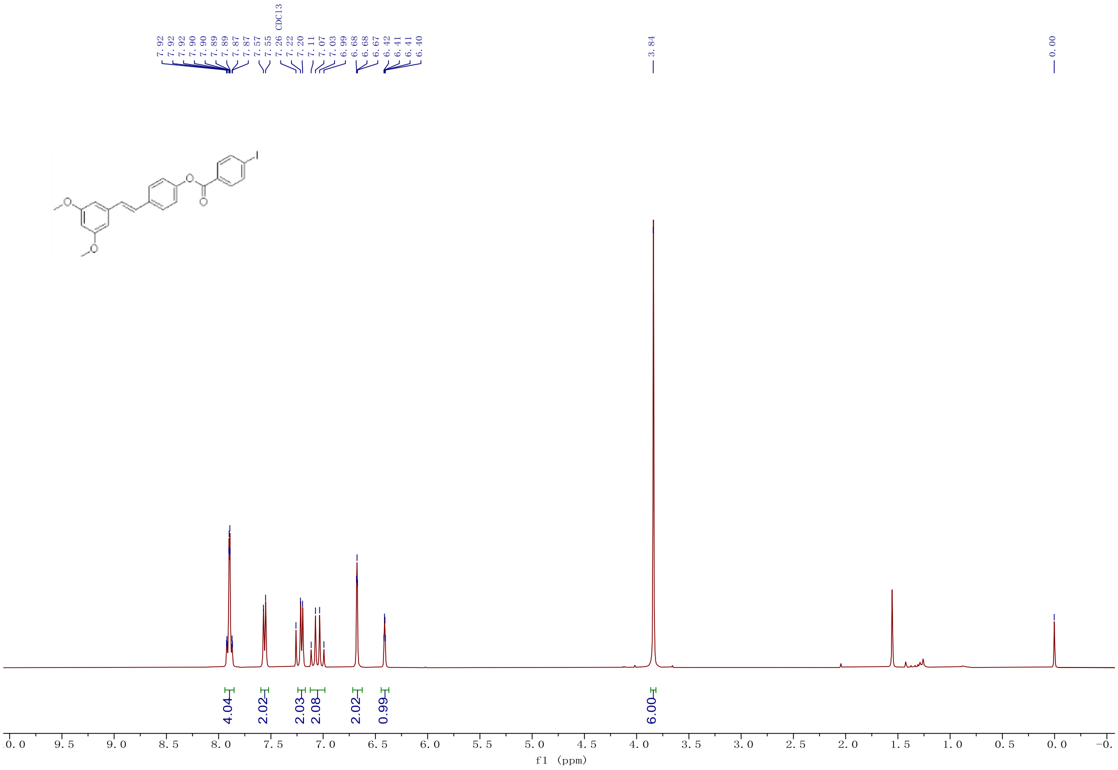

^{13}C NMR spectra of compound **a-69** in $CDCl_3$ (101 MHz): (*see procedure*)

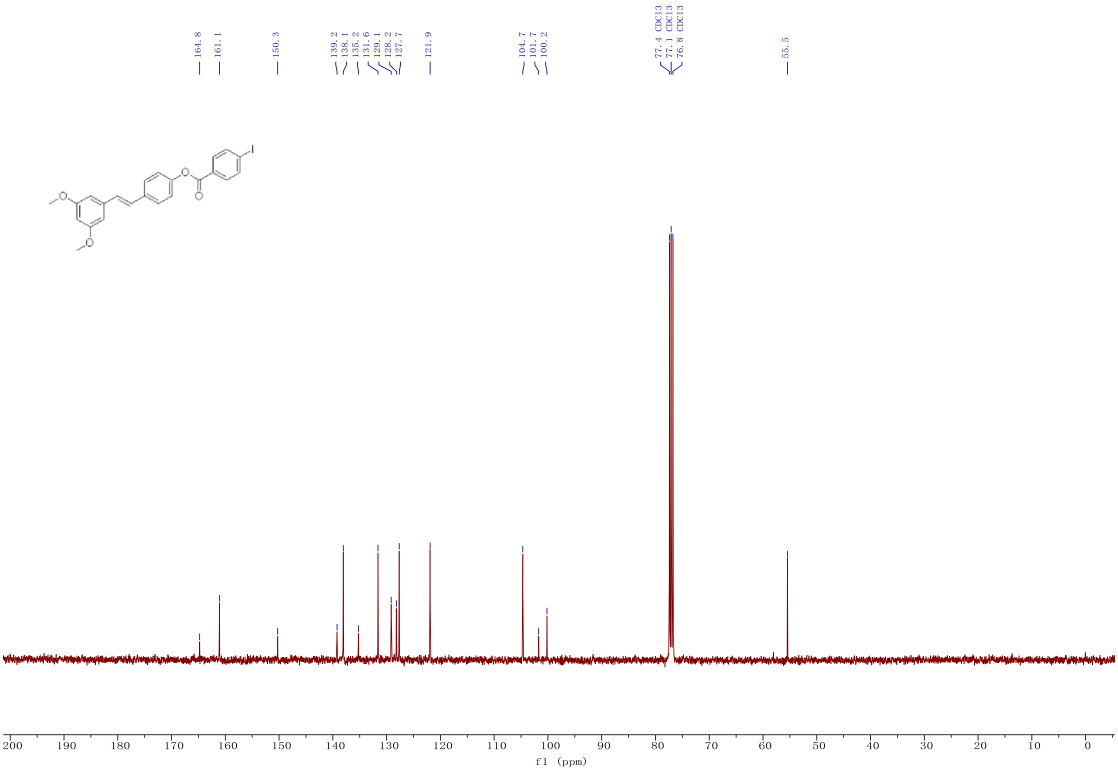

^{1}H NMR spectra of compound **a-70** in $CDCl_3$ (400 MHz): (*see procedure*)

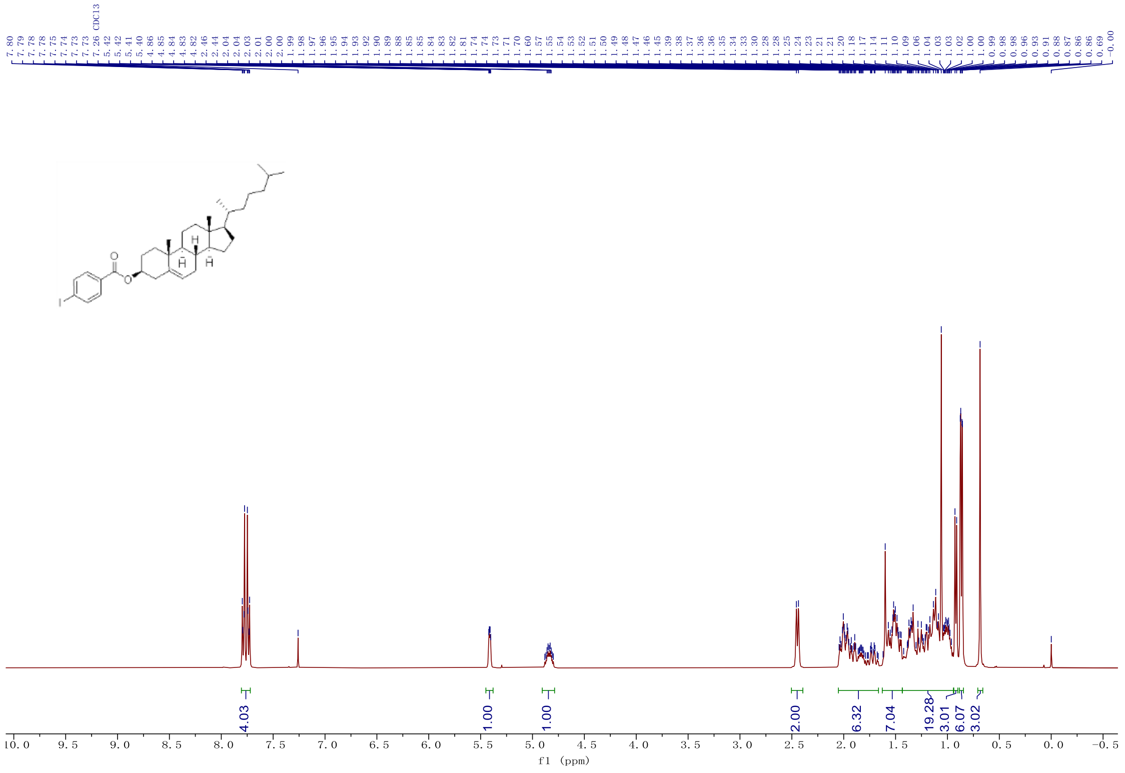

^{13}C NMR spectra of compound **a-70** in $CDCl_3$ (101 MHz): (*see procedure*)

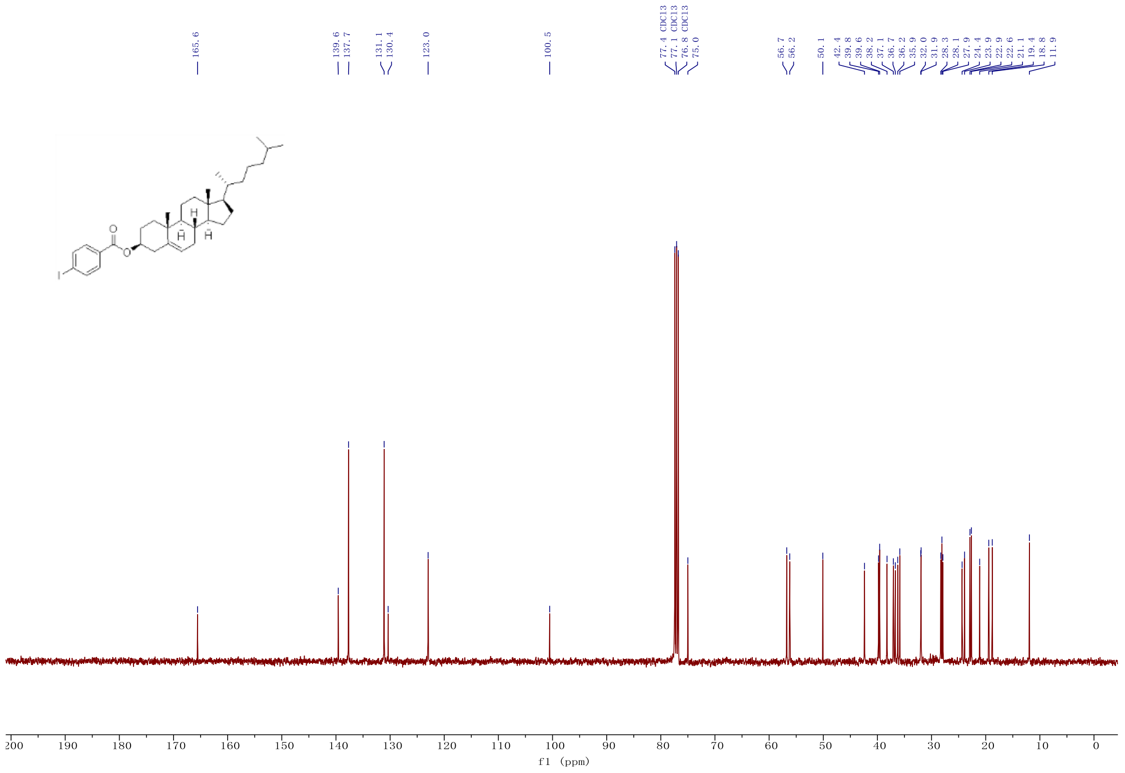

^{1}H NMR spectra of compound **a-71** in $CDCl_3$ (400 MHz): (*see procedure*)

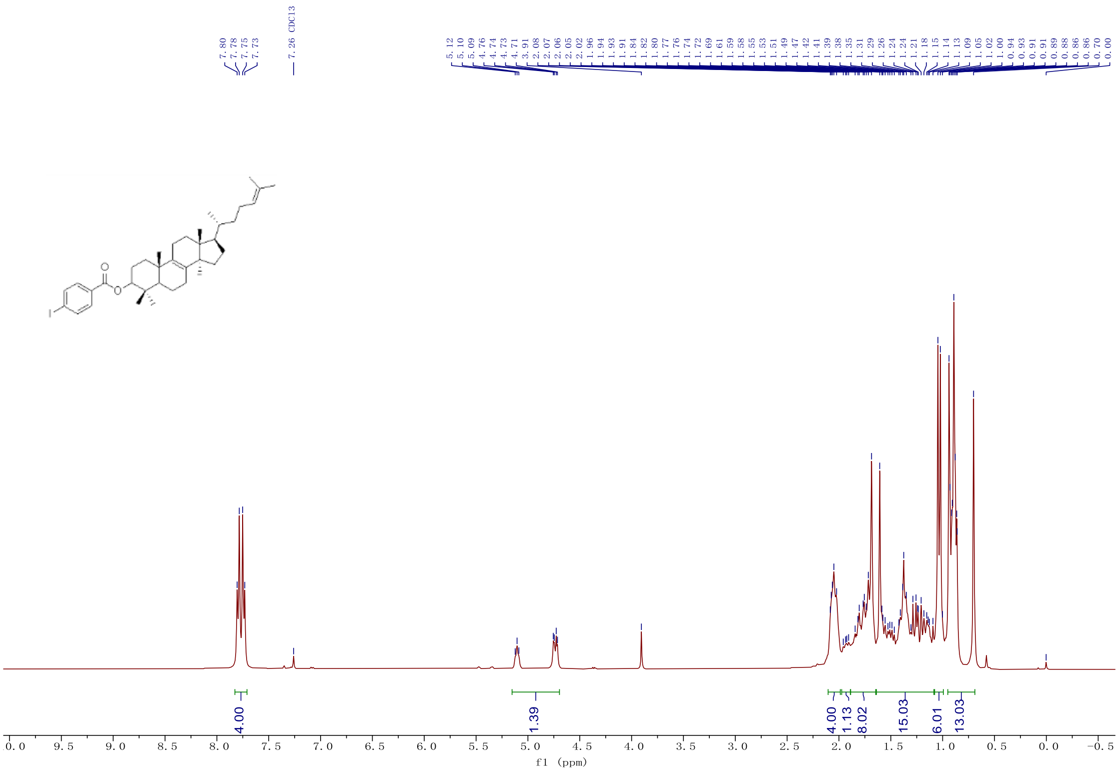

^{13}C NMR spectra of compound **a-71** in $CDCl_3$ (101 MHz): (*see procedure*)

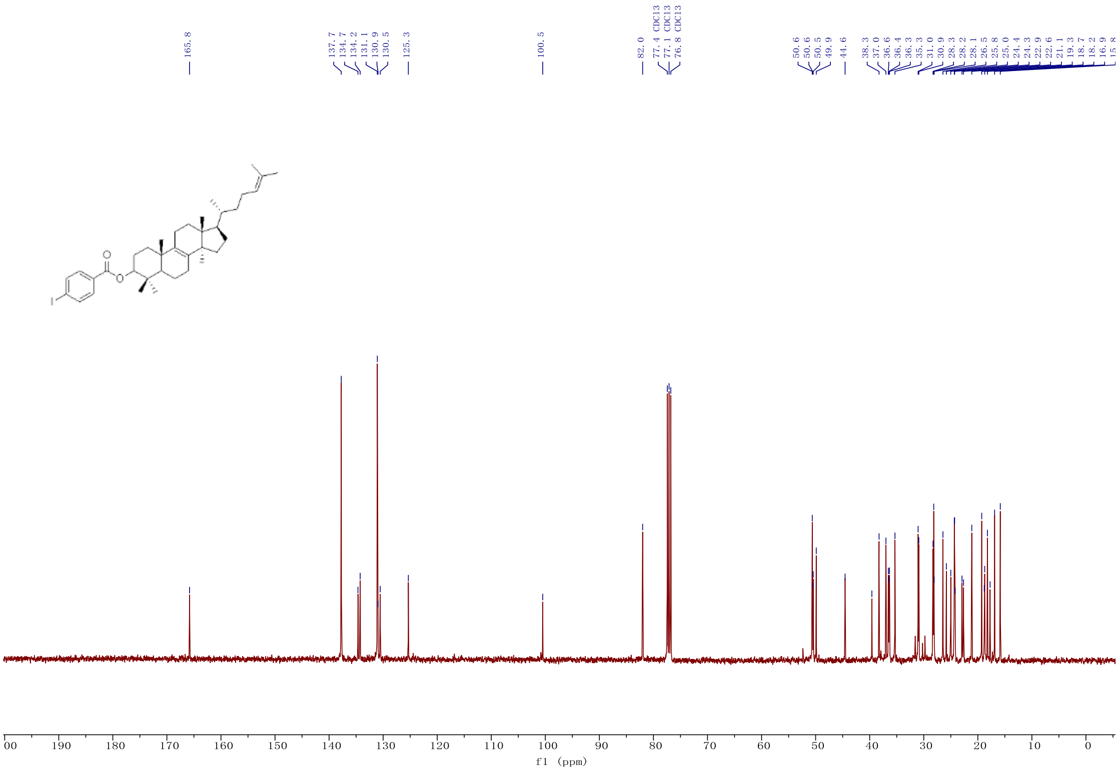

^{1}H NMR spectra of compound **a-72** in $CDCl_3$ (400 MHz): (*see procedure*)

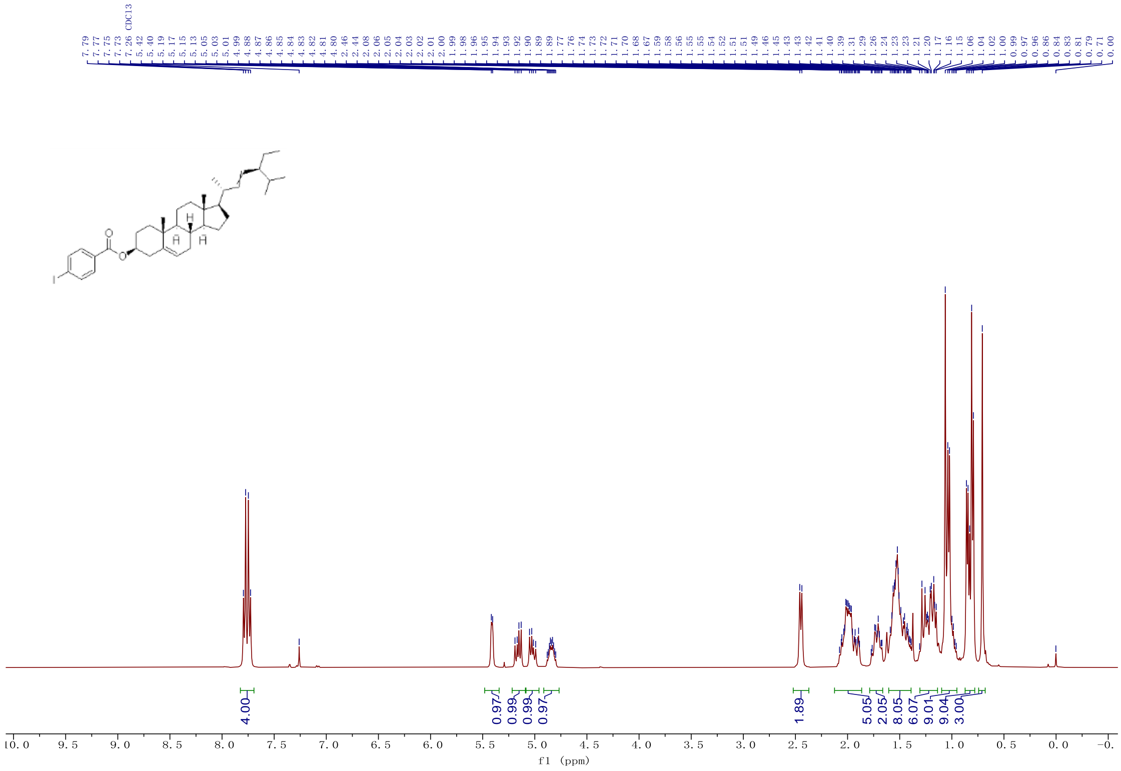

^{13}C NMR spectra of compound **a-72** in $CDCl_3$ (101 MHz): (*see procedure*)

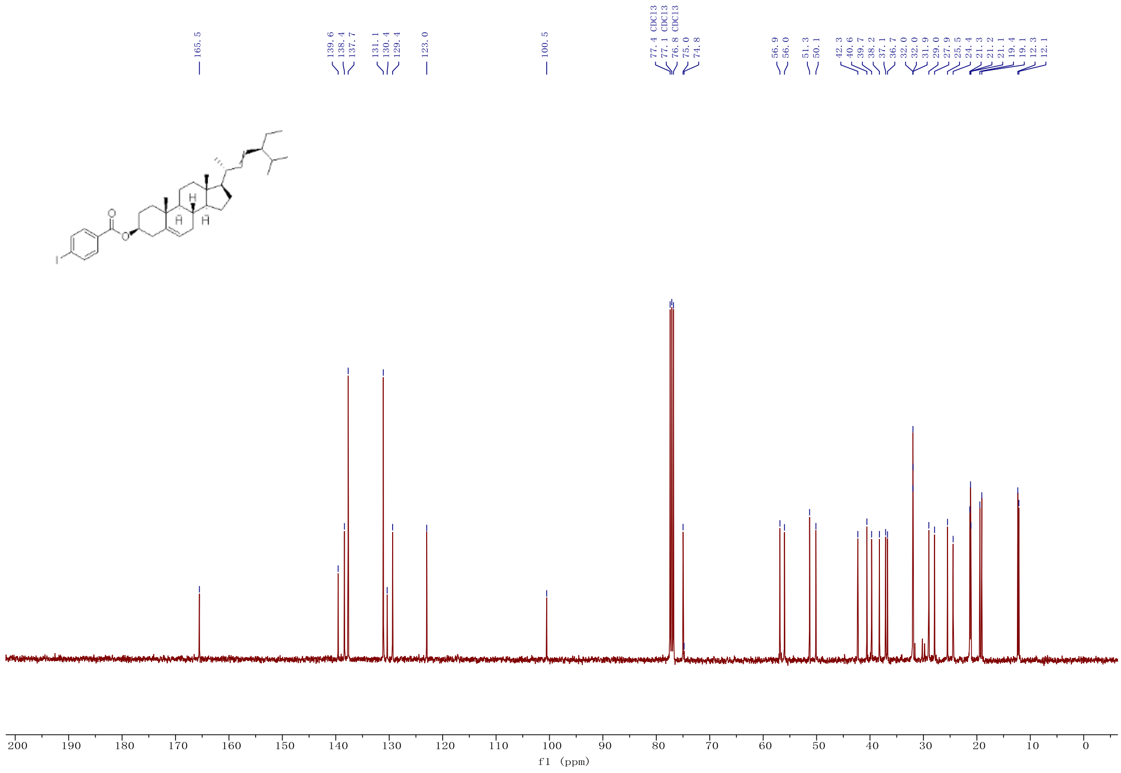

^{1}H NMR spectra of compound **b-74** in $CDCl_3$ (400 MHz): (*see procedure*)

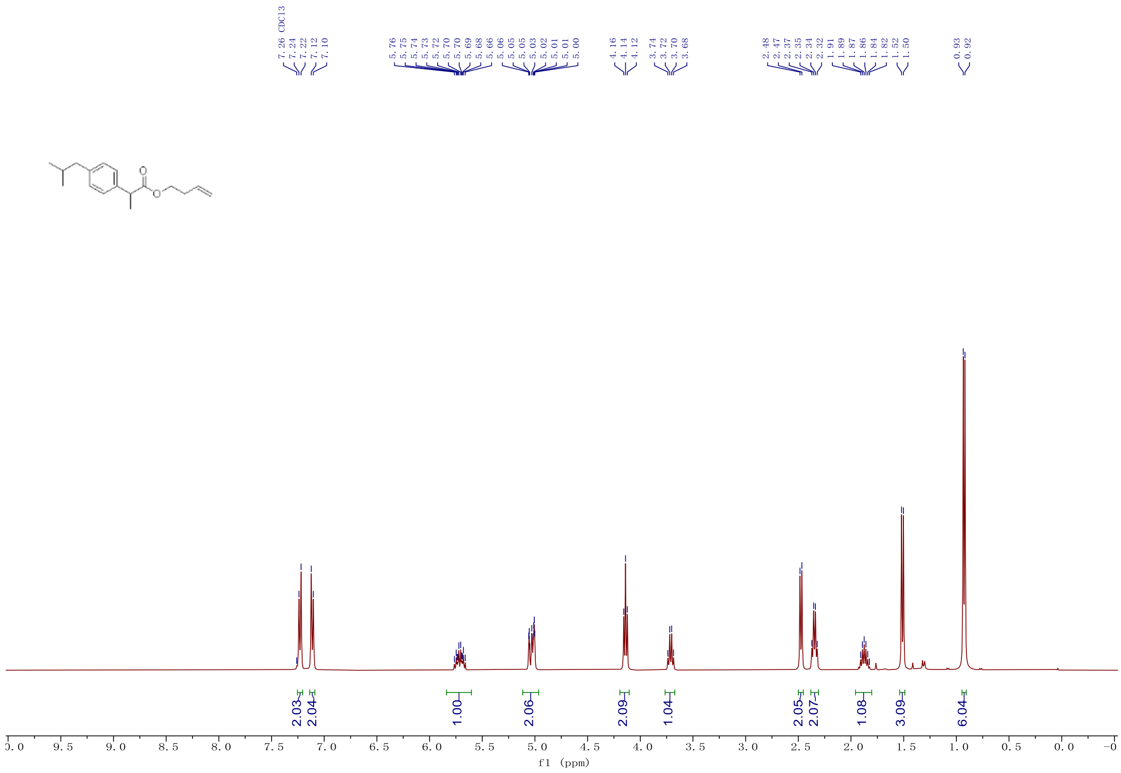

^{13}C NMR spectra of compound **b-74** in $CDCl_3$ (101 MHz): (*see procedure*)

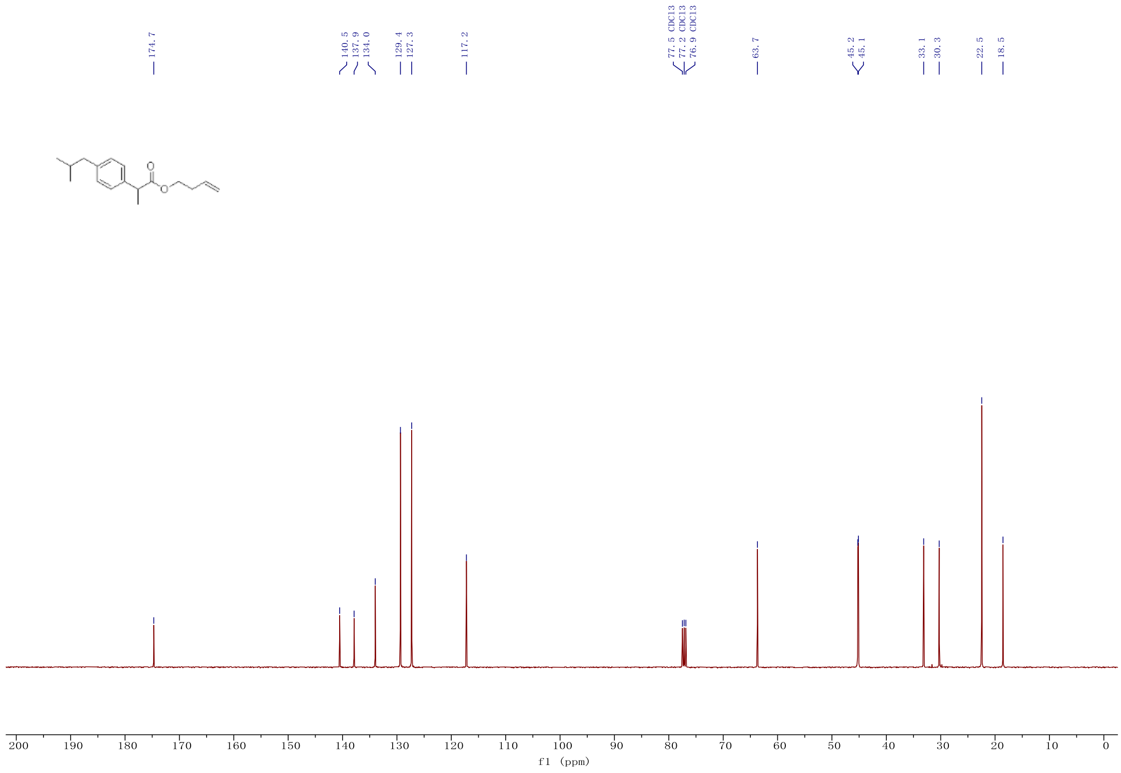

^{1}H NMR spectra of compound **b-75** in $CDCl_3$ (400 MHz): (*see procedure*)

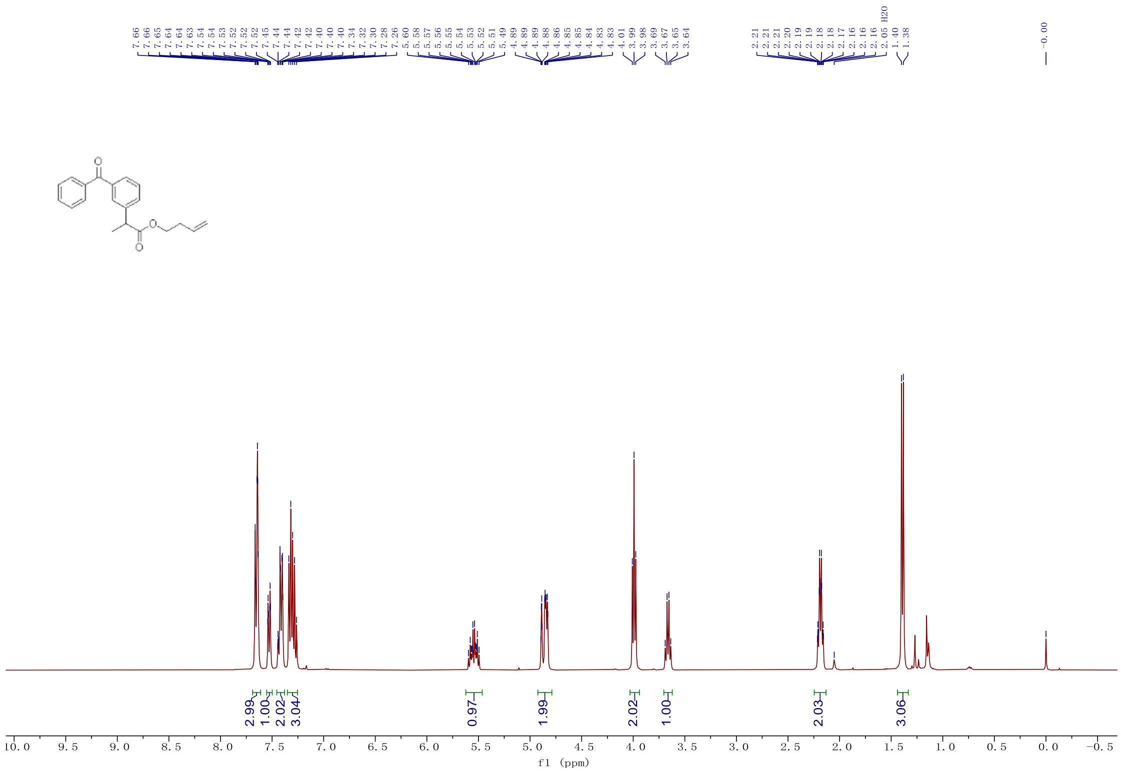

^{13}C NMR spectra of compound **b-75** in $CDCl_3$ (101 MHz): (*see procedure*)

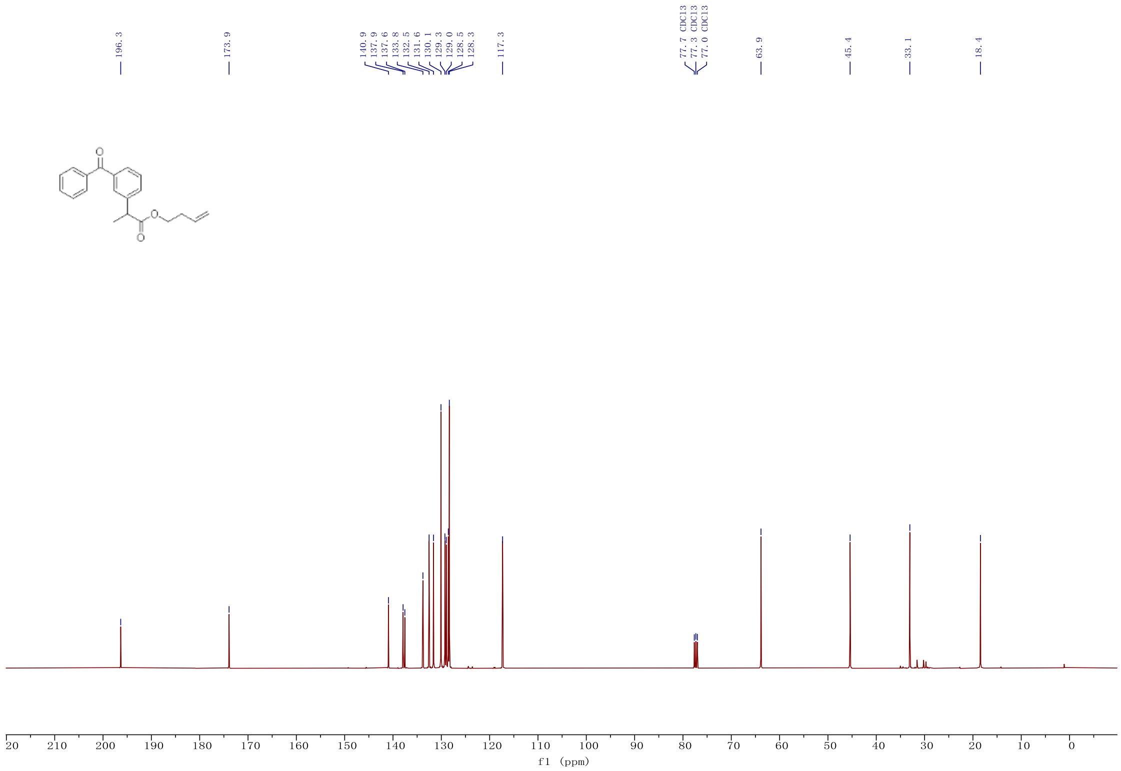

^{1}H NMR spectra of compound **b-76** in $CDCl_3$ (400 MHz): (see procedure)

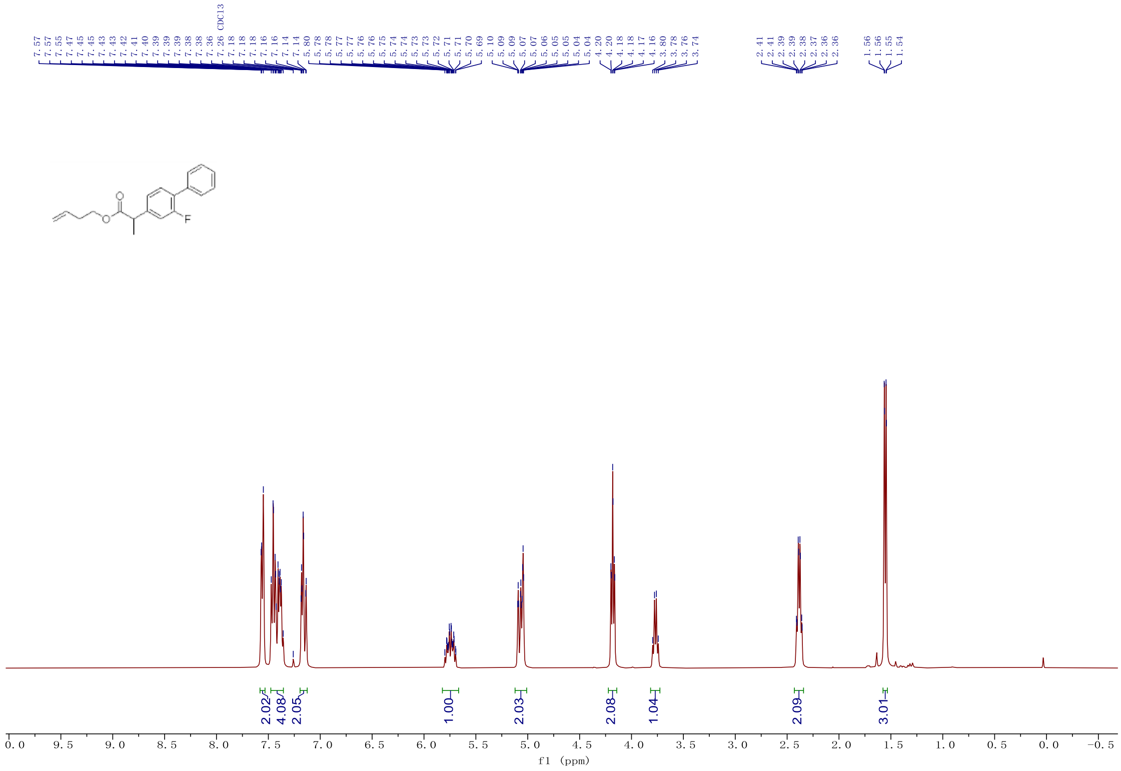

^{13}C NMR spectra of compound **b-76** in $CDCl_3$ (101 MHz): (see procedure)

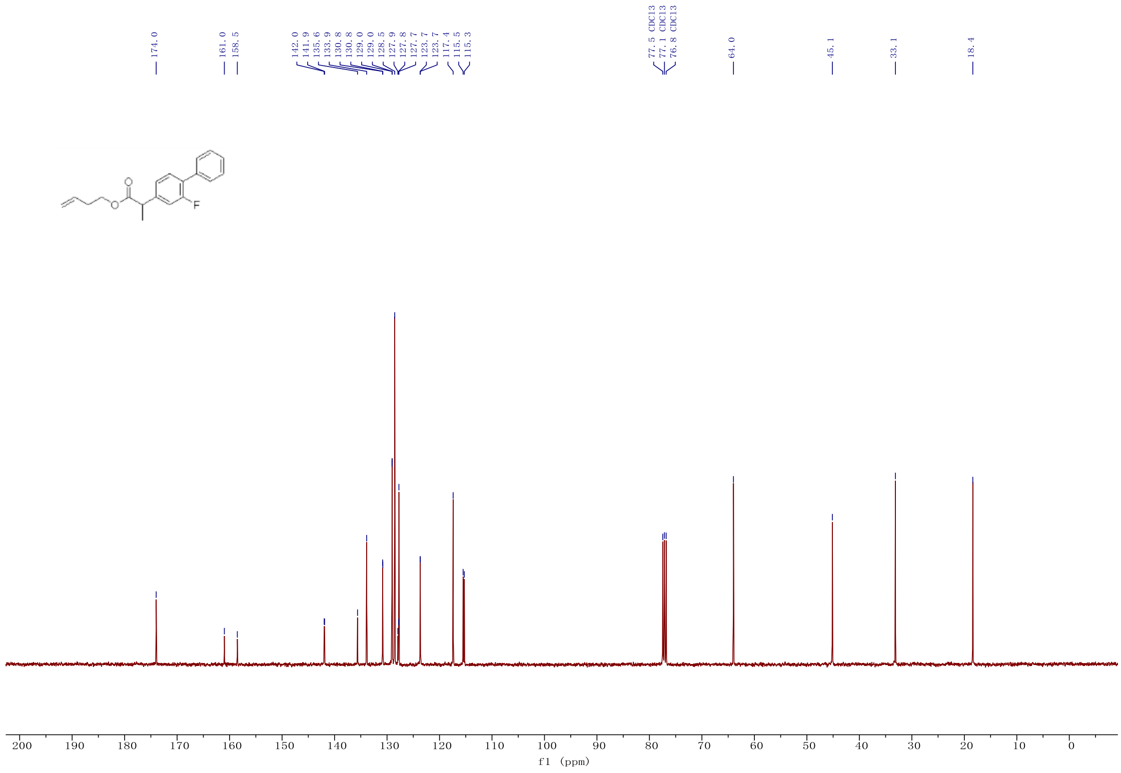

^{19}F NMR spectra of compound **b-76** in $CDCl_3$ (376 MHz): (*see procedure*)

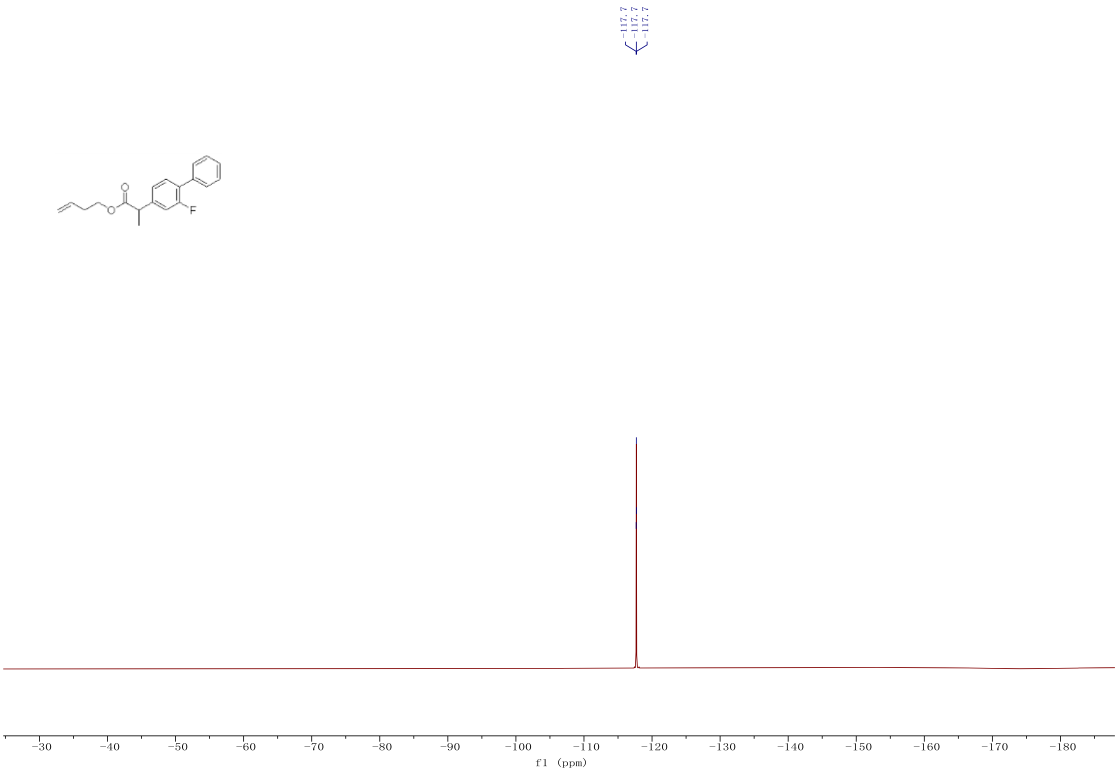

^{1}H NMR spectra of compound **b-77** in $CDCl_3$ (400 MHz): (*see procedure*)

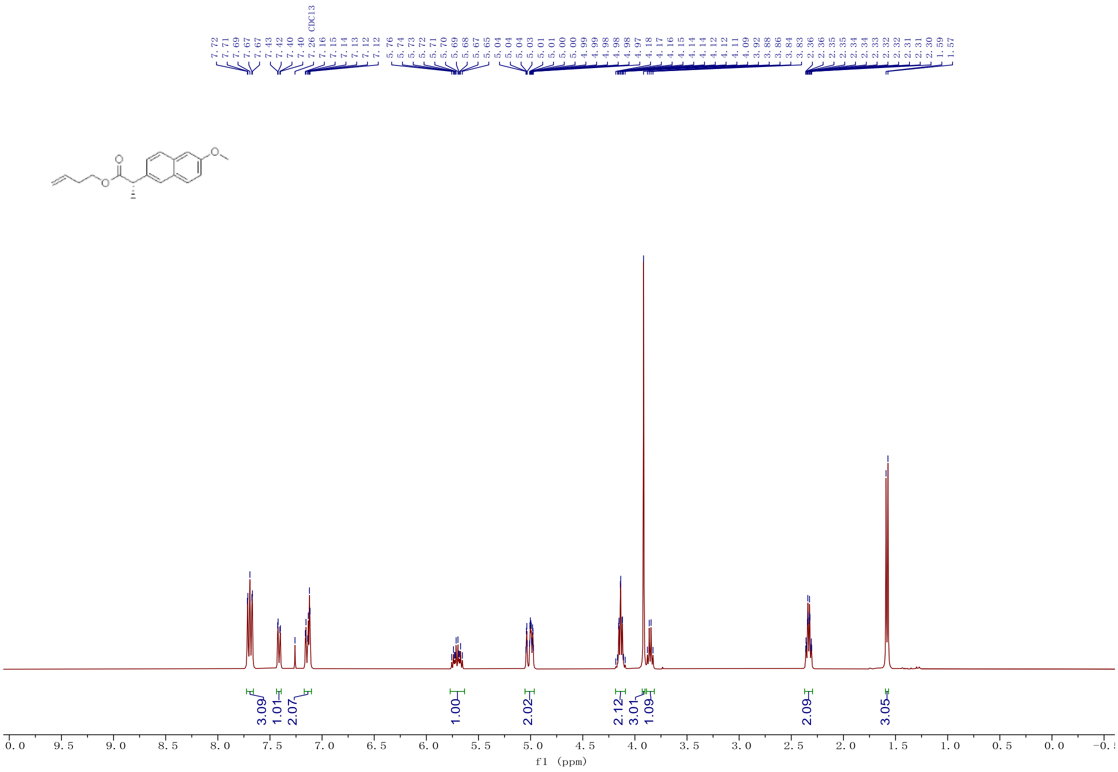

^{13}C NMR spectra of compound **b-77** in $CDCl_3$ (101 MHz): (*see procedure*)

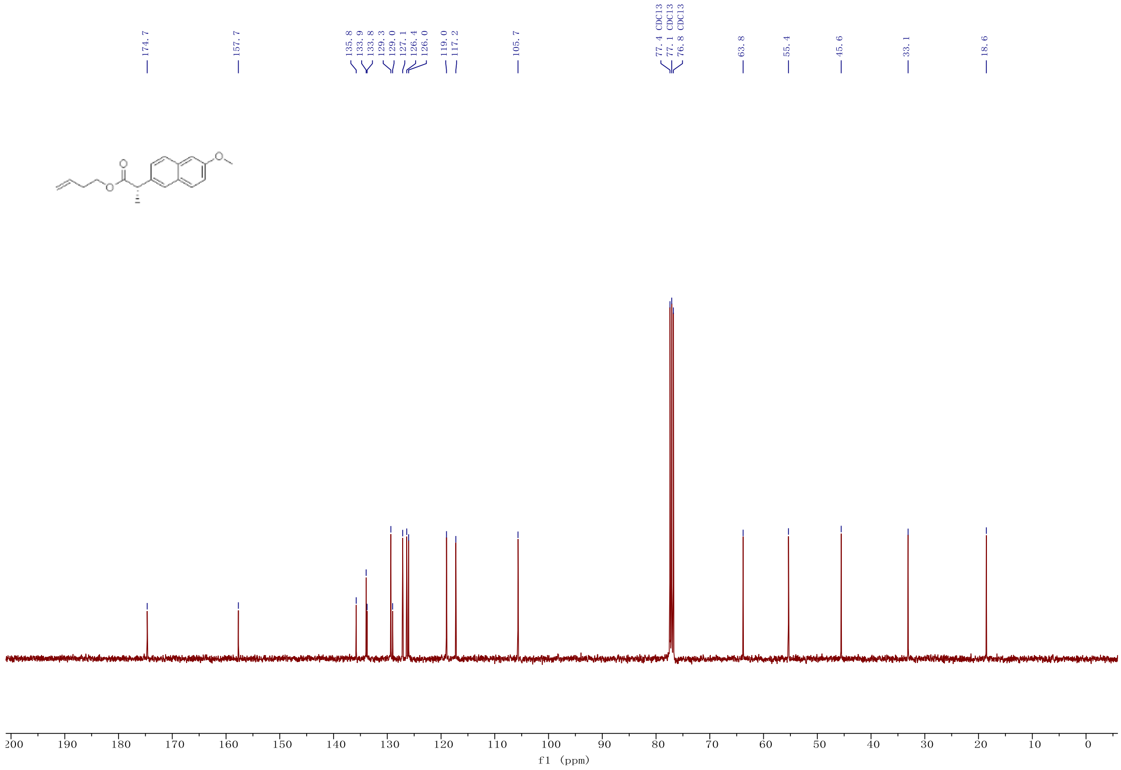

^{1}H NMR spectra of compound **b-78** in $CDCl_3$ (400 MHz): (*see procedure*)

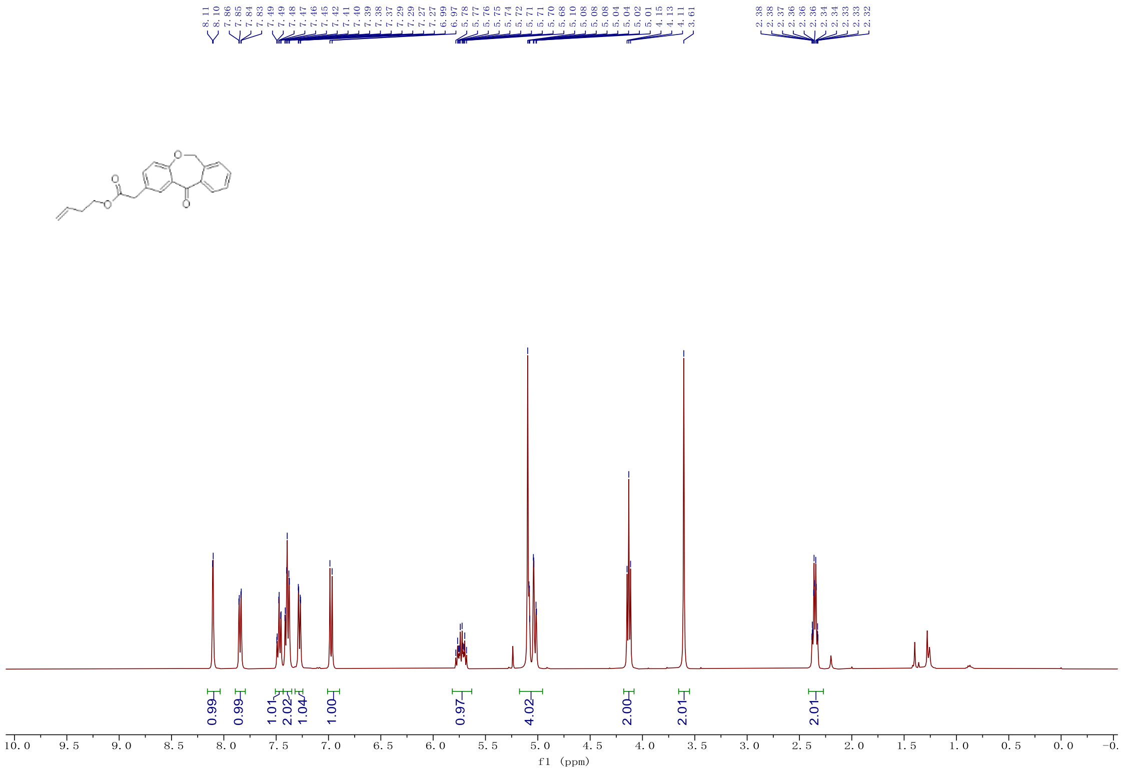

^{13}C NMR spectra of compound **b-78** in $CDCl_3$ (101 MHz): (*see procedure*)

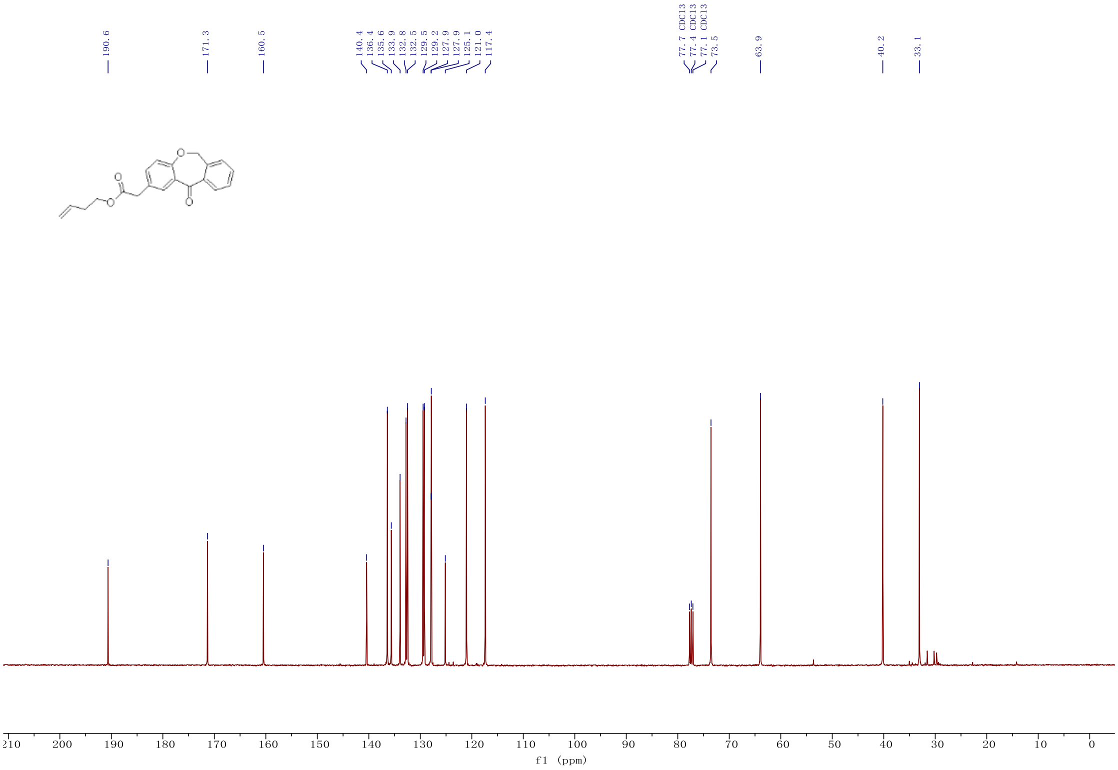

^{1}H NMR spectra of compound **b-79** in $CDCl_3$ (400 MHz): (*see procedure*)

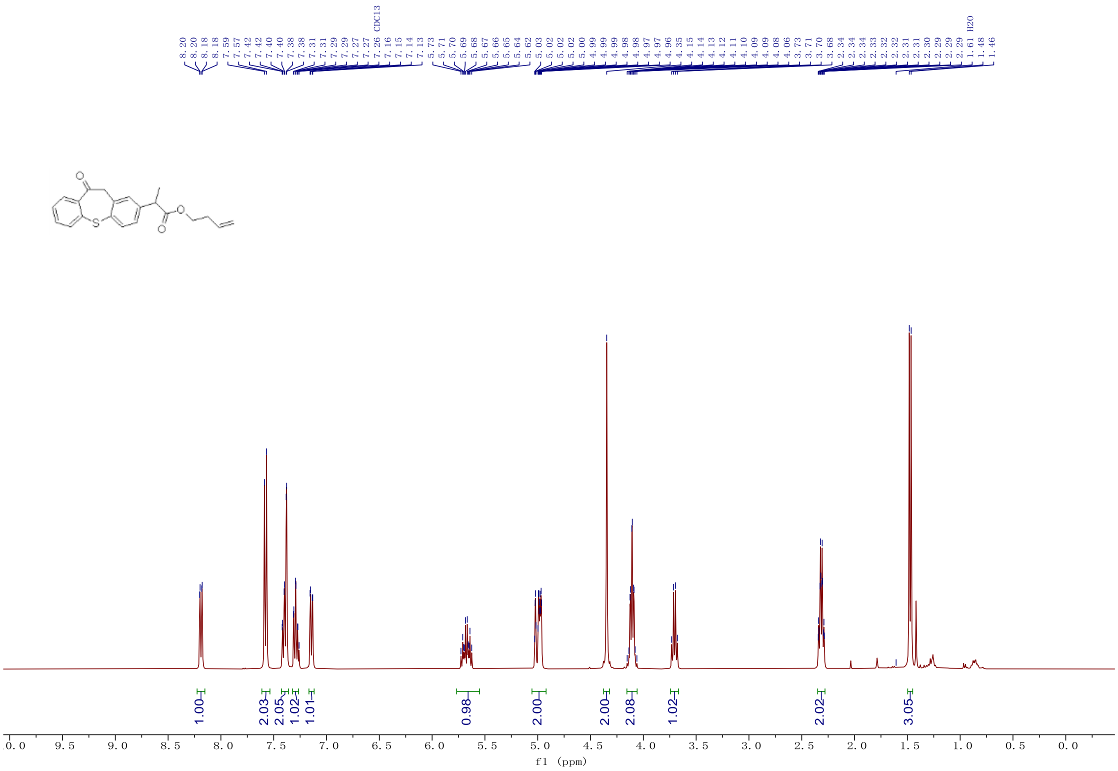

^{13}C NMR spectra of compound **b-79** in $CDCl_3$ (101 MHz): (*see procedure*)

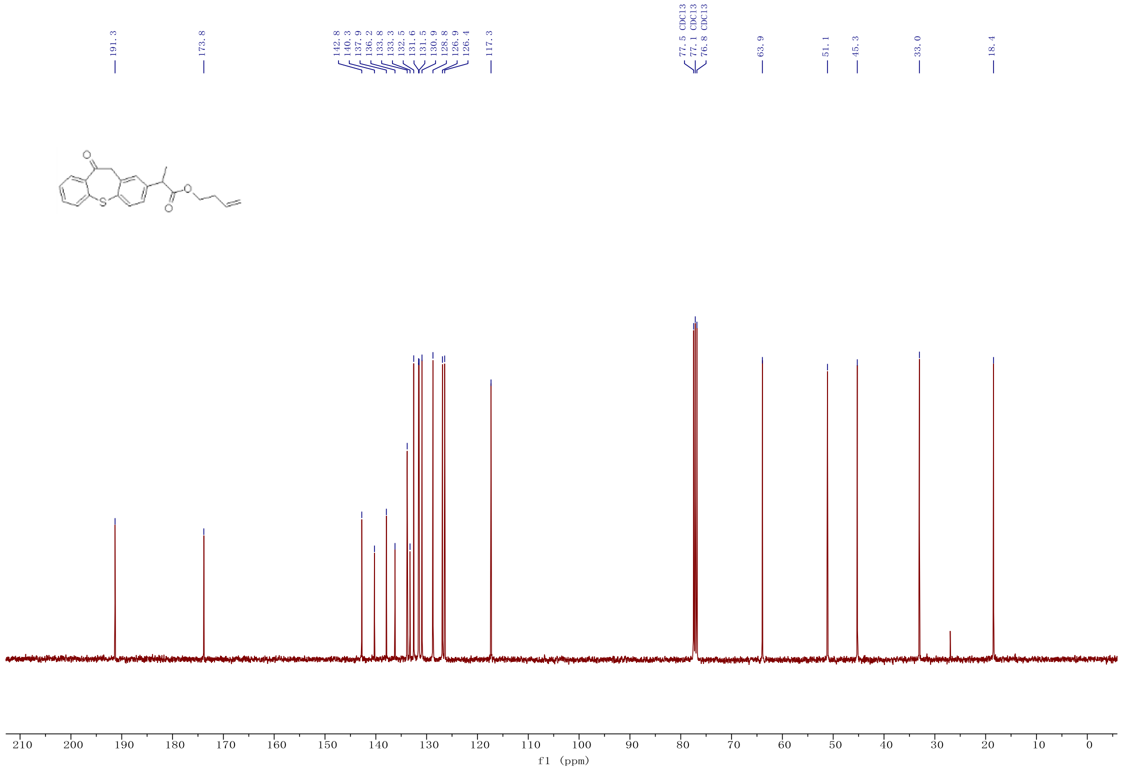

^{1}H NMR spectra of compound **b-80** in $CDCl_3$ (400 MHz): (*see procedure*)

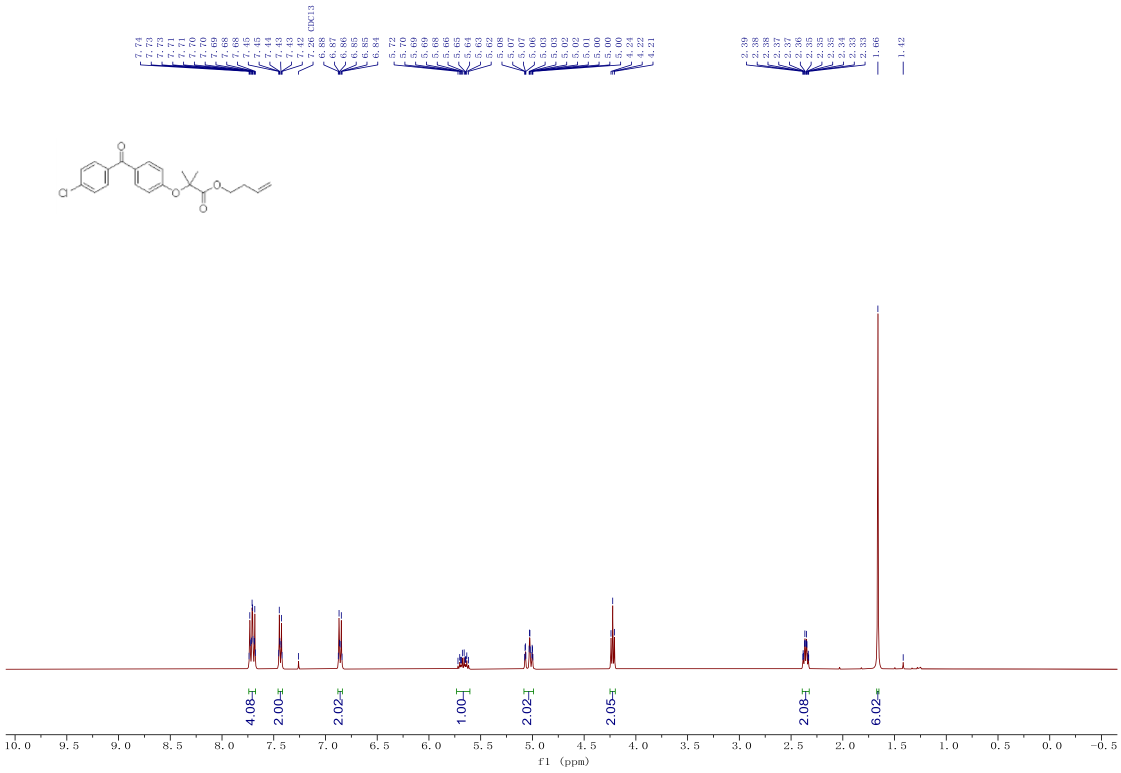

^{13}C NMR spectra of compound **b-80** in $CDCl_3$ (101 MHz): (*see procedure*)

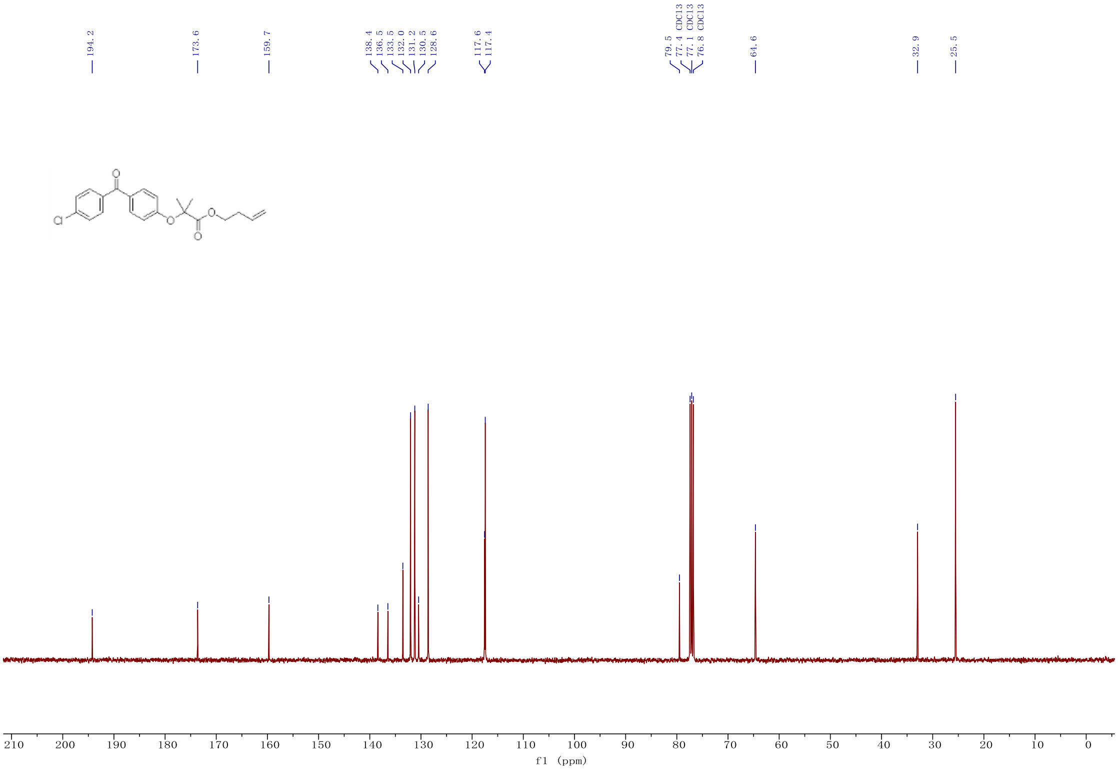

^{1}H NMR spectra of compound **d-1** in $CDCl_3$ (400 MHz): (*see procedure*)

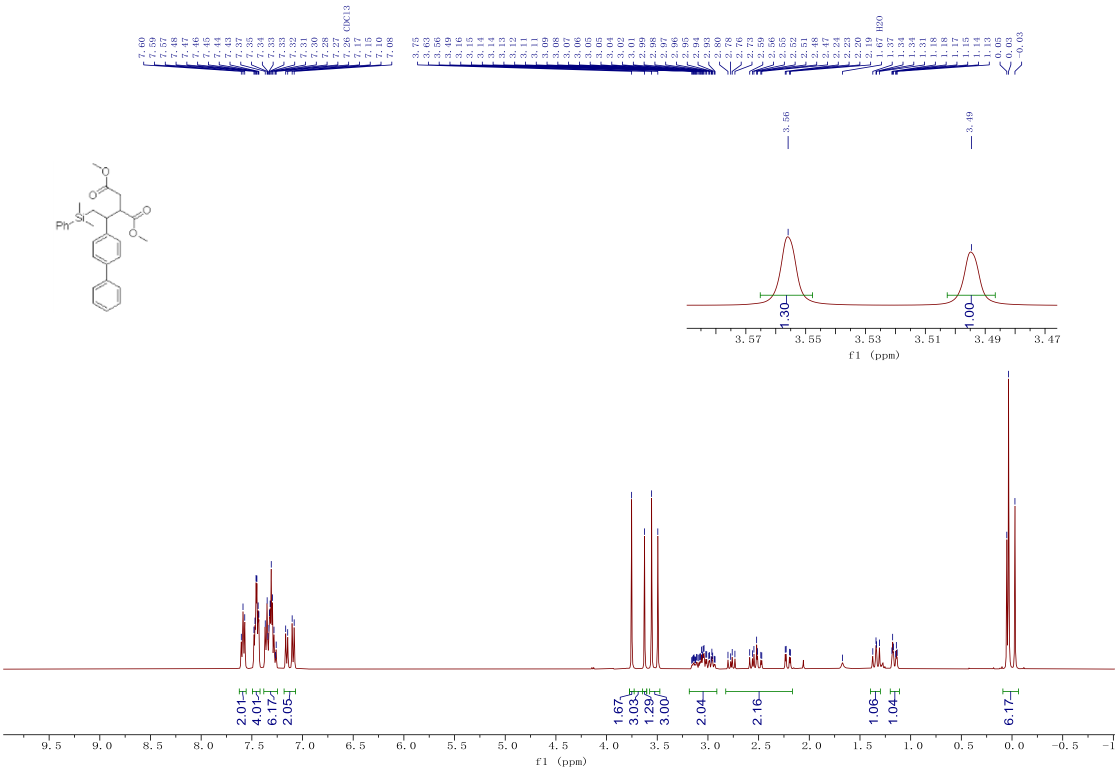

^{13}C NMR spectra of compound **d-1** in $CDCl_3$ (101 MHz): (*see procedure*)

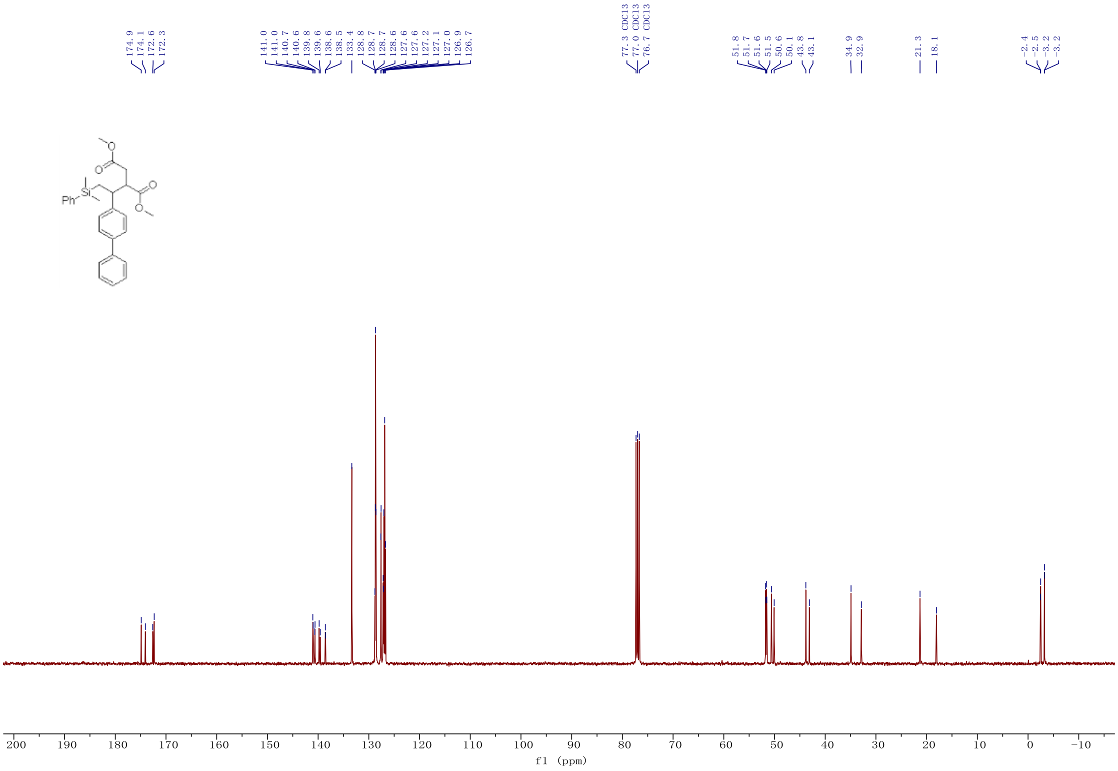

^{1}H NMR spectra of compound **d-2** in $CDCl_3$ (400 MHz): (*see procedure*)

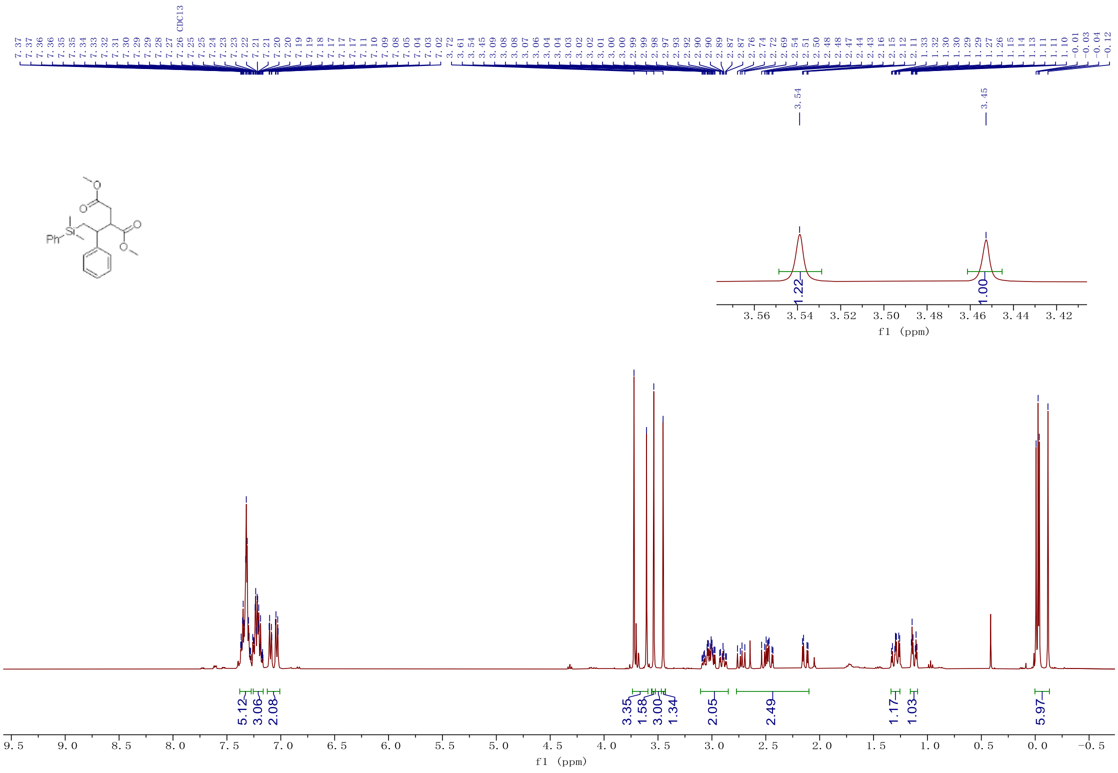

^{13}C NMR spectra of compound **d-2** in $CDCl_3$ (101 MHz): (see procedure)

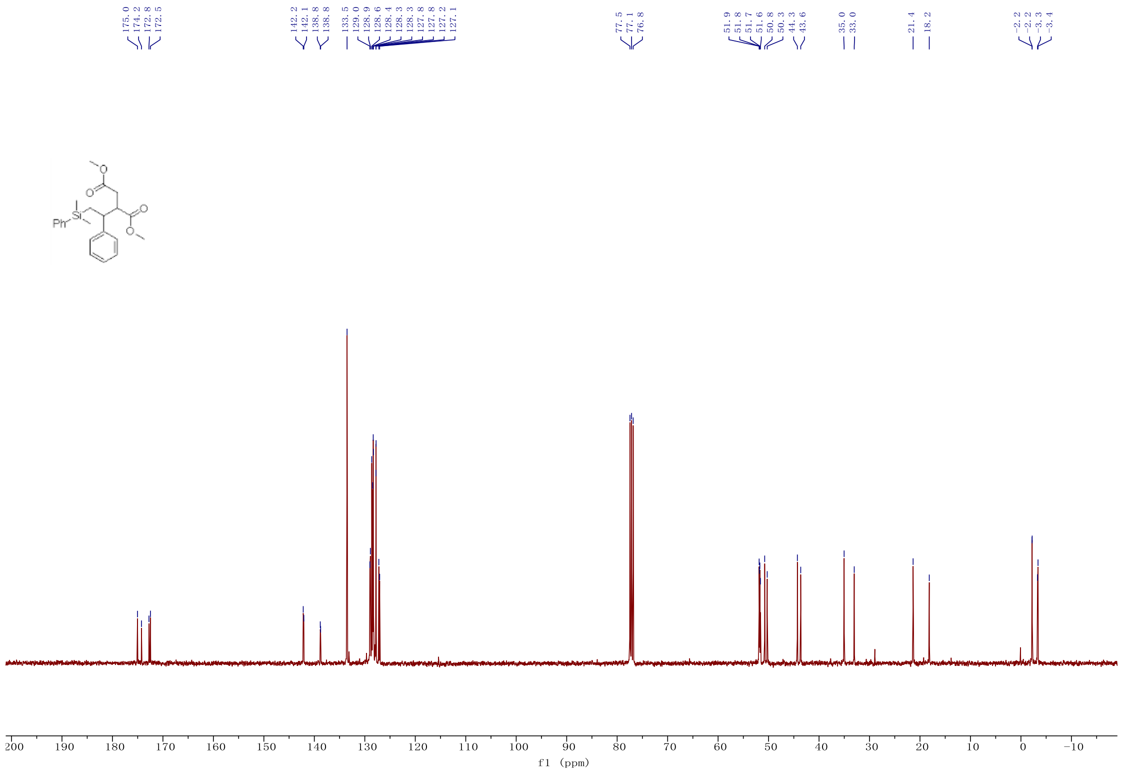

^{1}H NMR spectra of compound **d-3** in $CDCl_3$ (400 MHz): (see procedure)

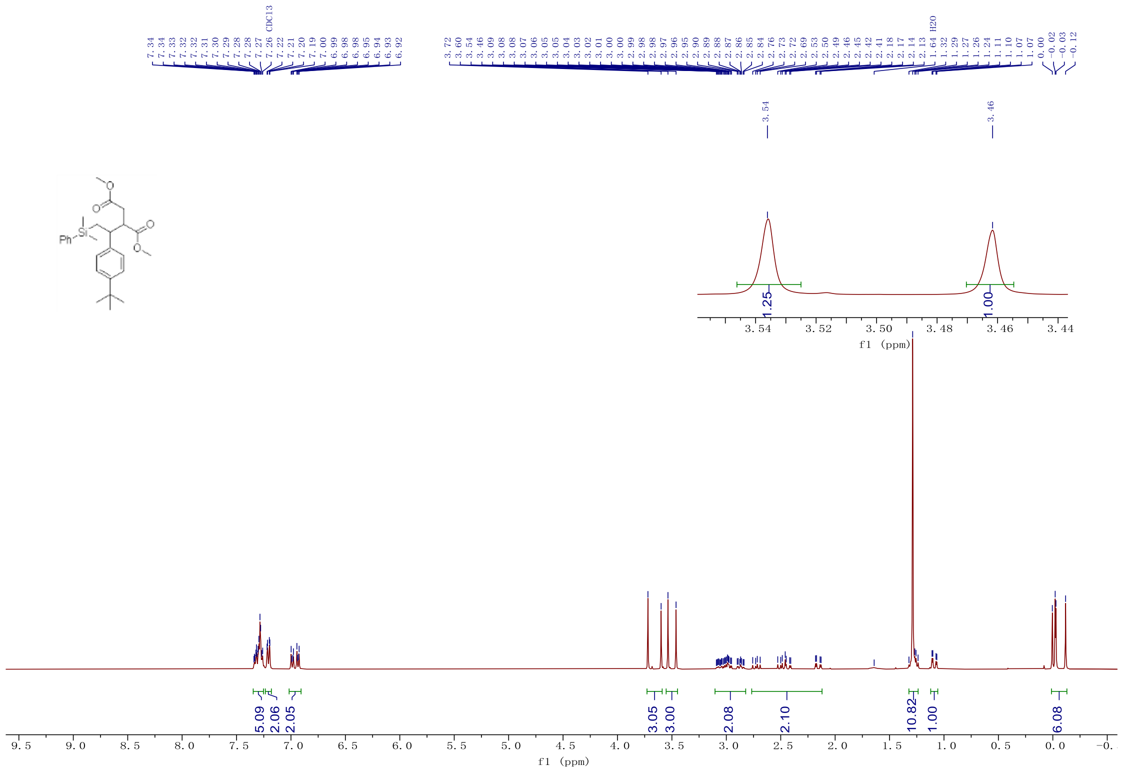

^{13}C NMR spectra of compound **d-3** in $CDCl_3$ (101 MHz): (<u>*see procedure*</u>)

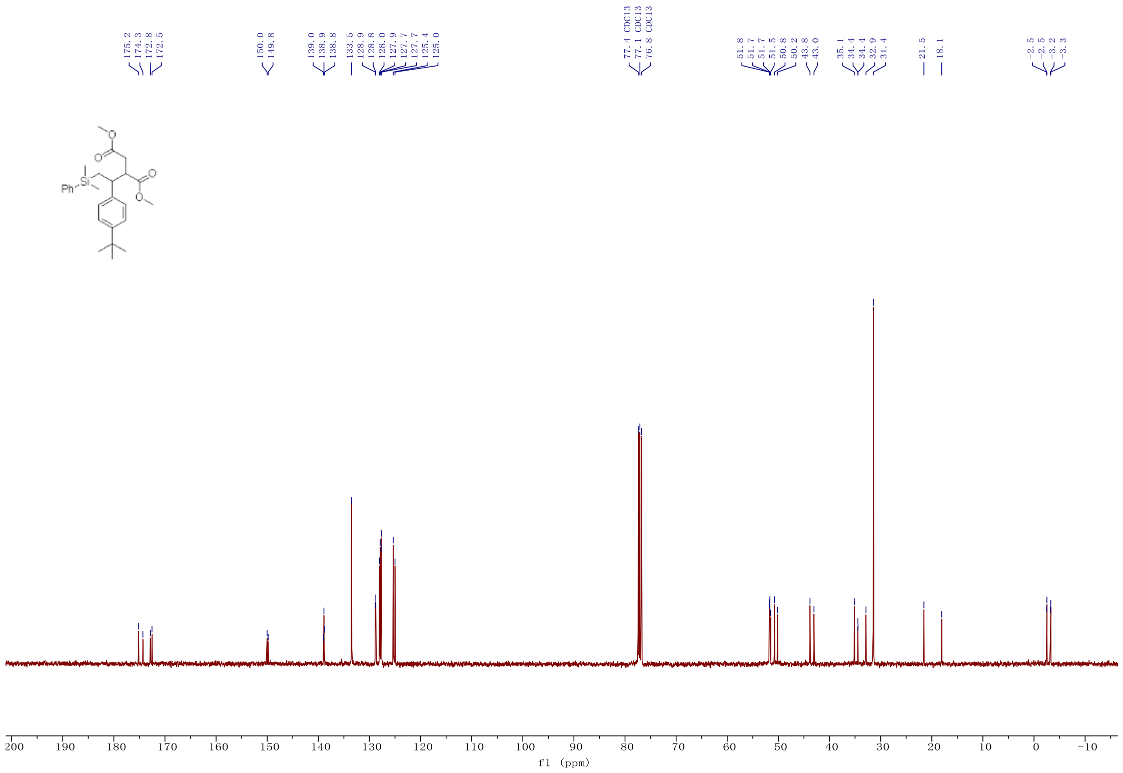

^{1}H NMR spectra of compound **d-4** in $CDCl_3$ (400 MHz): (<u>*see procedure*</u>)

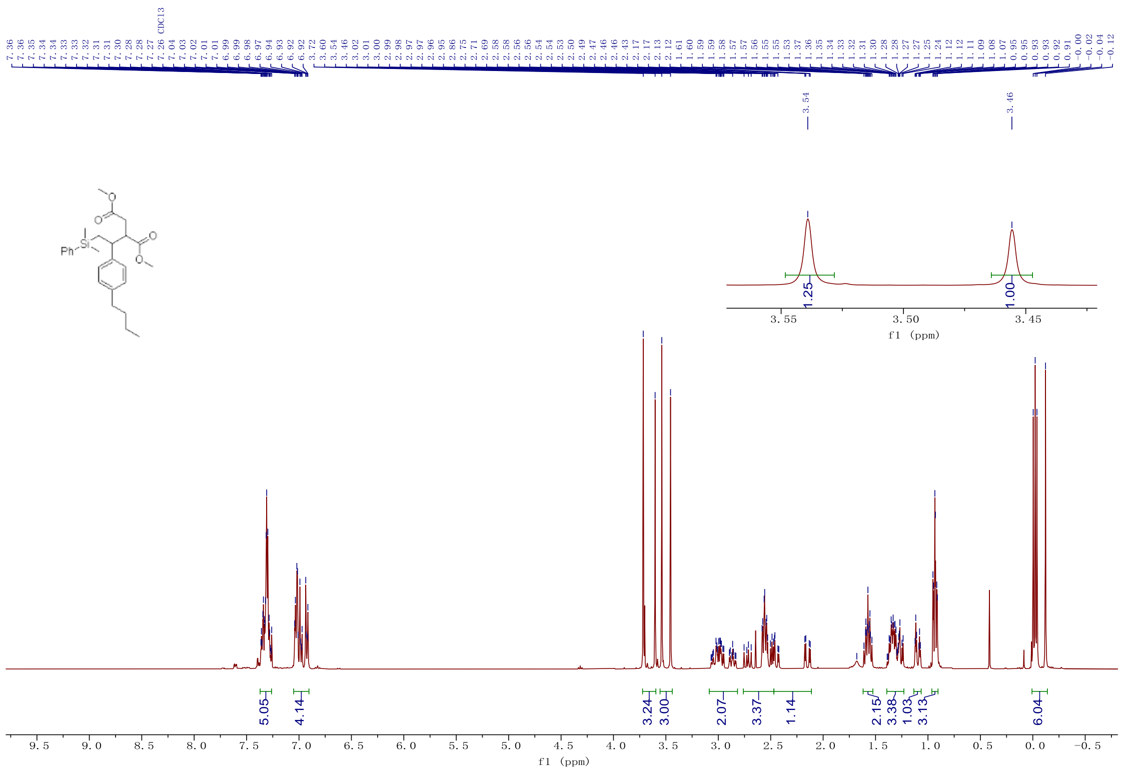

^{13}C NMR spectra of compound **d-4** in $CDCl_3$ (101 MHz): (see procedure)

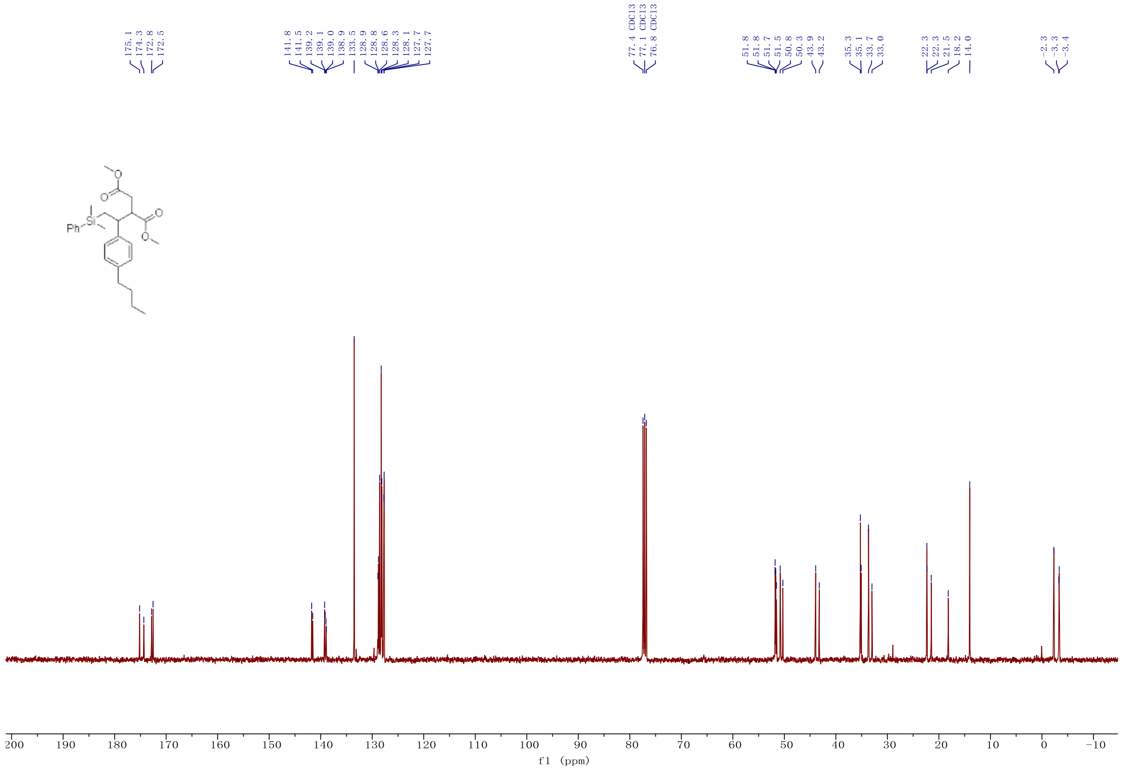

^{1}H NMR spectra of compound **d-5** in $CDCl_3$ (400 MHz): (see procedure)

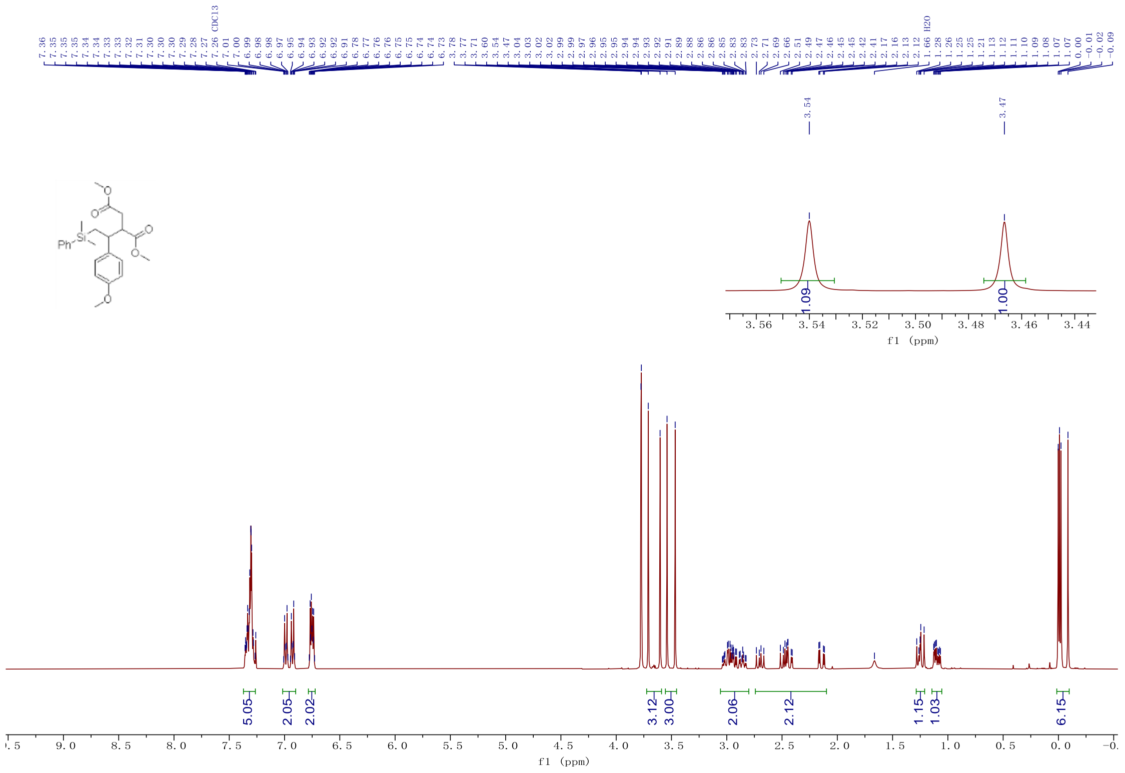

^{13}C NMR spectra of compound **d-5** in $CDCl_3$ (101 MHz): (<u>*see procedure*</u>)

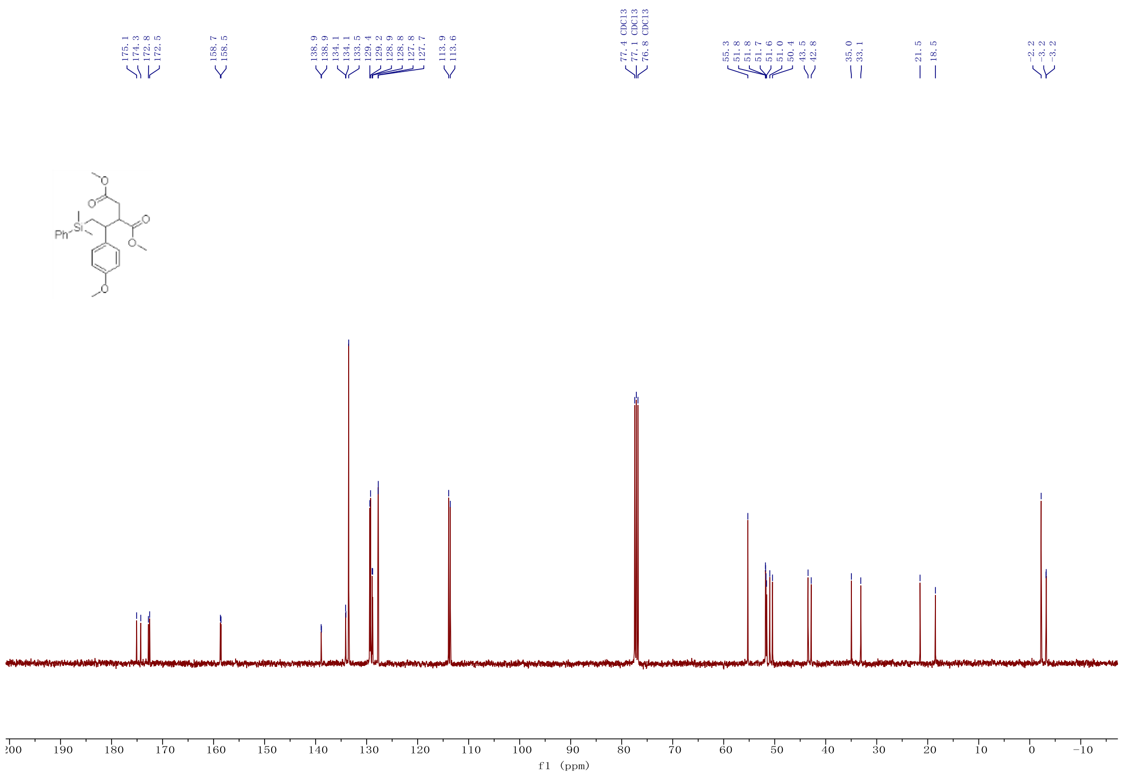

^{1}H NMR spectra of compound **d-6** in $CDCl_3$ (400 MHz): (<u>*see procedure*</u>)

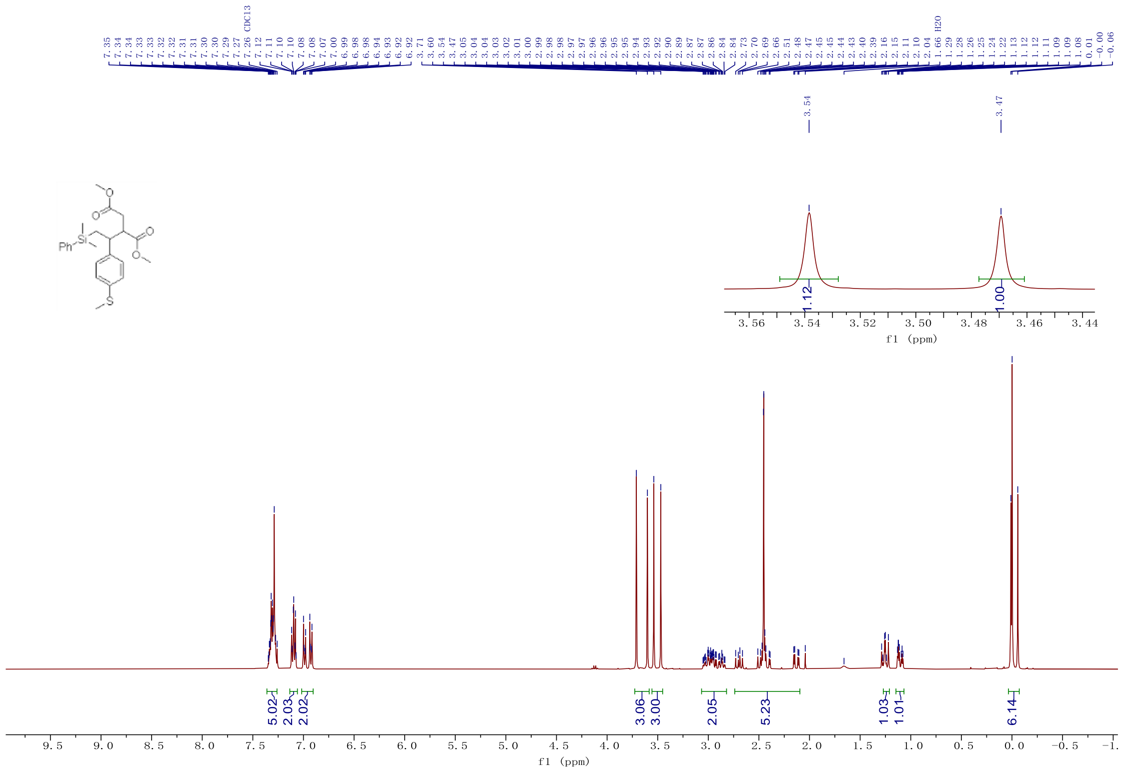

^{13}C NMR spectra of compound **d-6** in $CDCl_3$ (101 MHz): (see procedure)

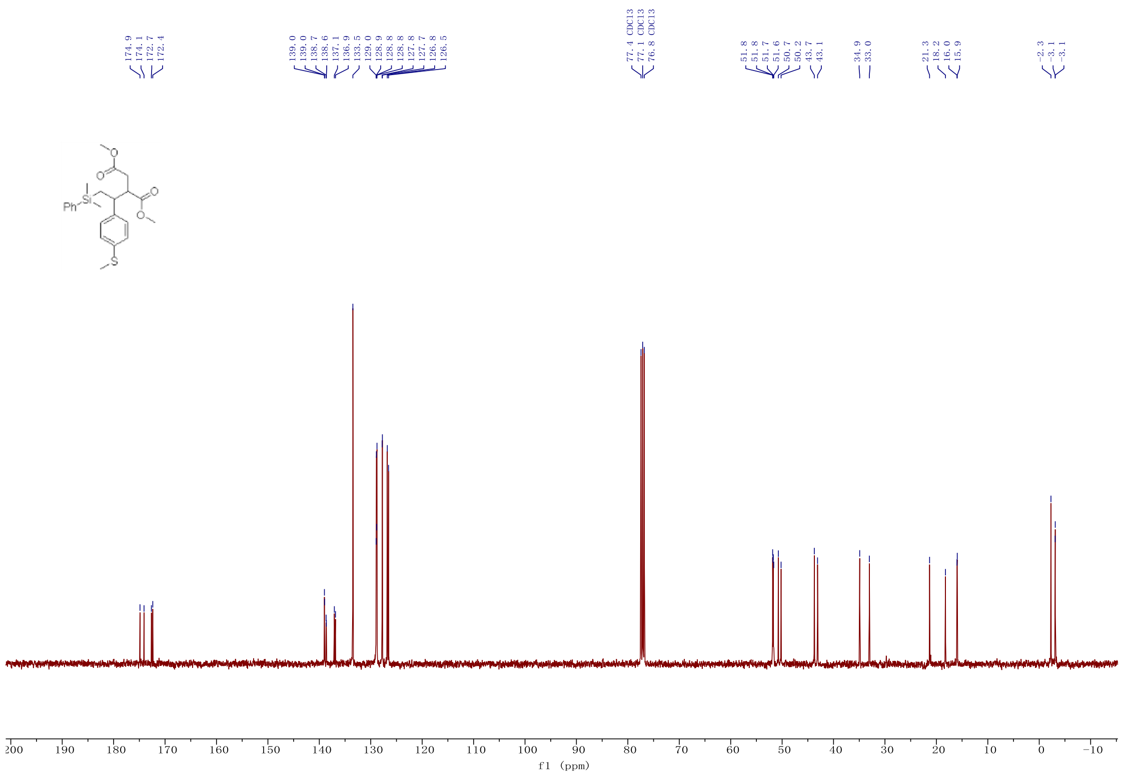

^{1}H NMR spectra of compound **d-7** in $CDCl_3$ (400 MHz): (see procedure)

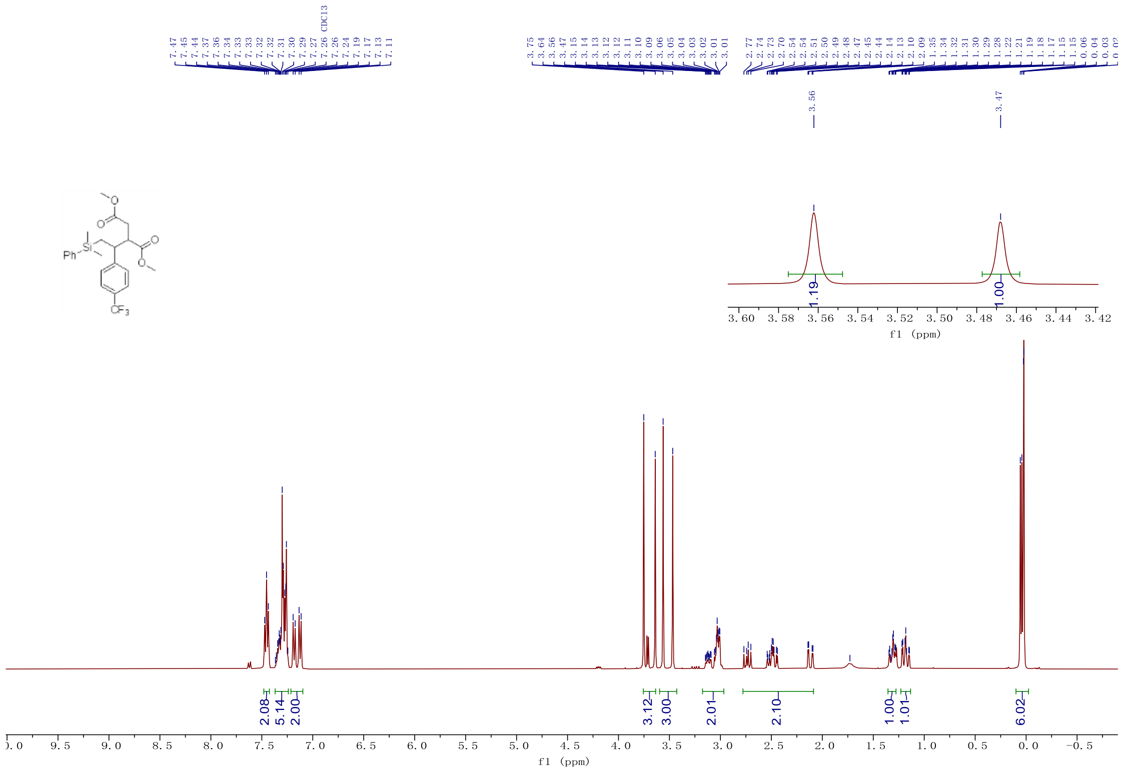

^{13}C NMR spectra of compound **d-7** in $CDCl_3$ (101 MHz): (*see procedure*)

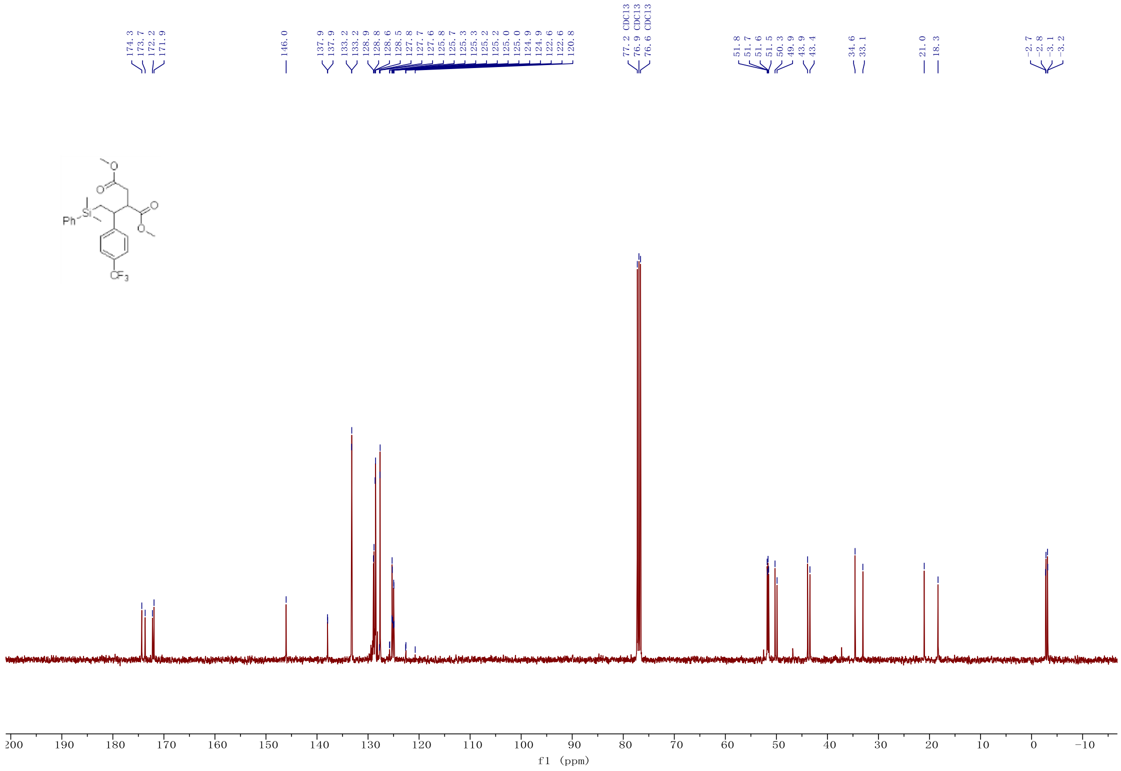

^{19}F NMR spectra of compound **d-7** in $CDCl_3$ (376 MHz): (*see procedure*)

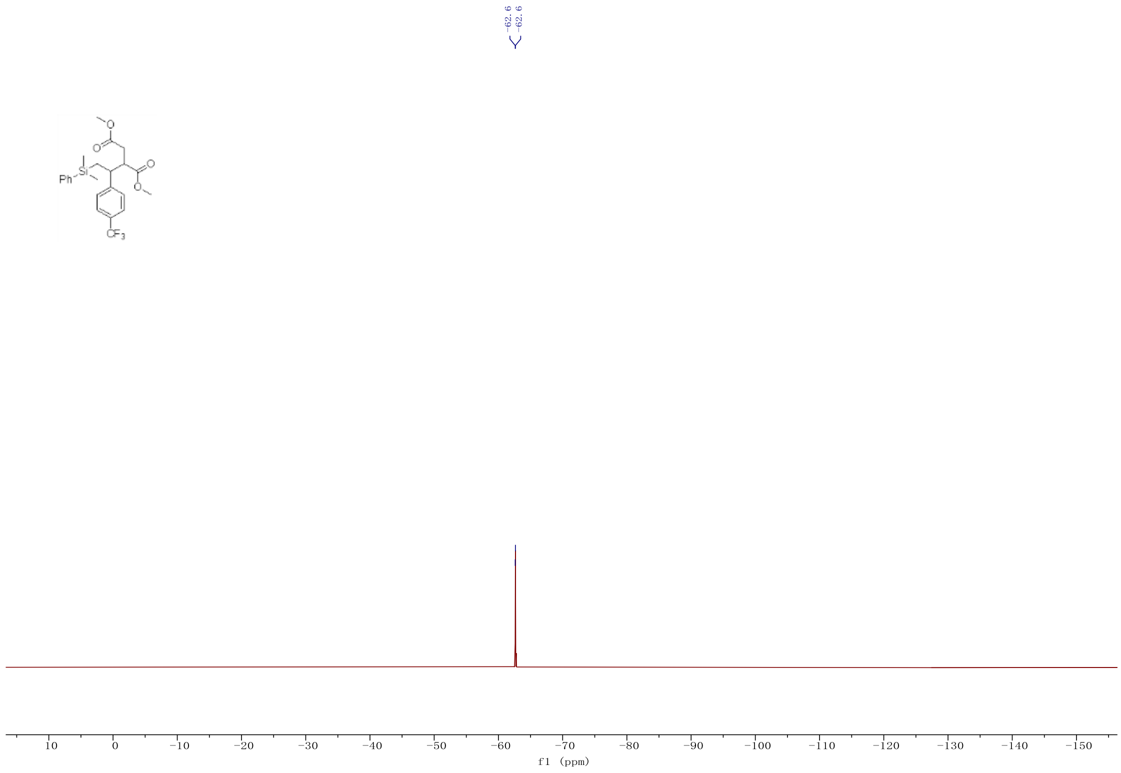

^{1}H NMR spectra of compound **d-8** in $CDCl_3$ (400 MHz): (*see procedure*)

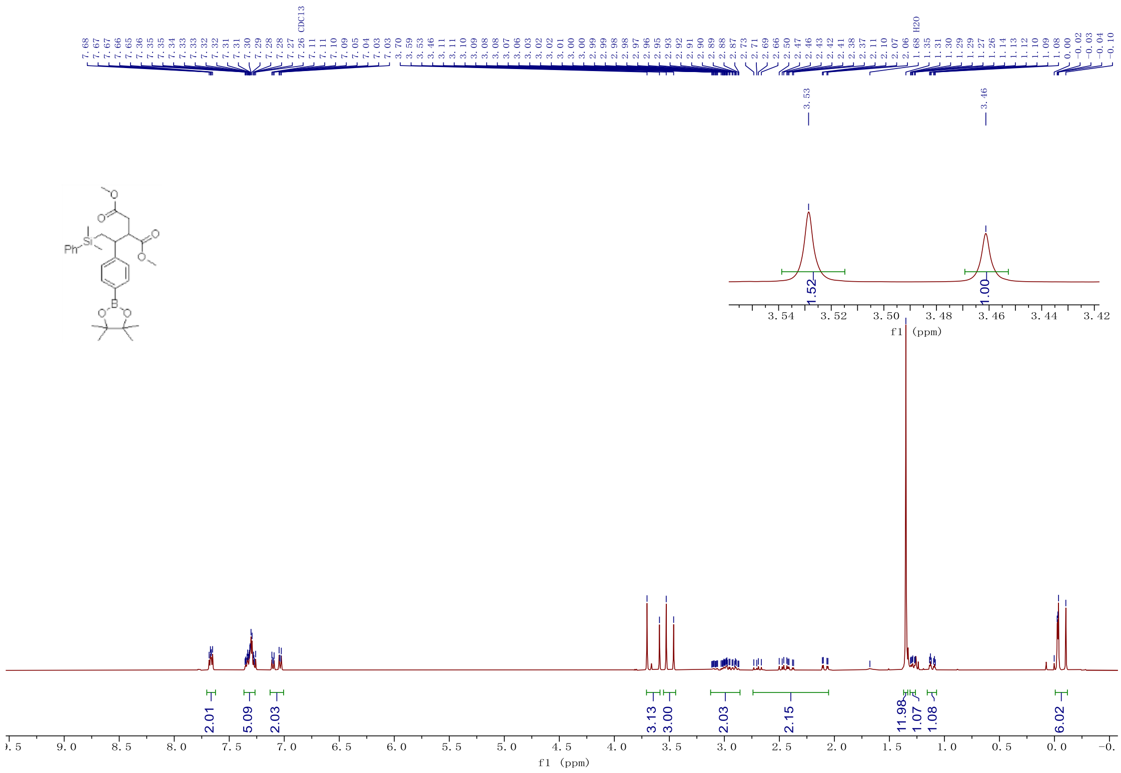

^{13}C NMR spectra of compound **d-8** in $CDCl_3$ (101 MHz): (*see procedure*)

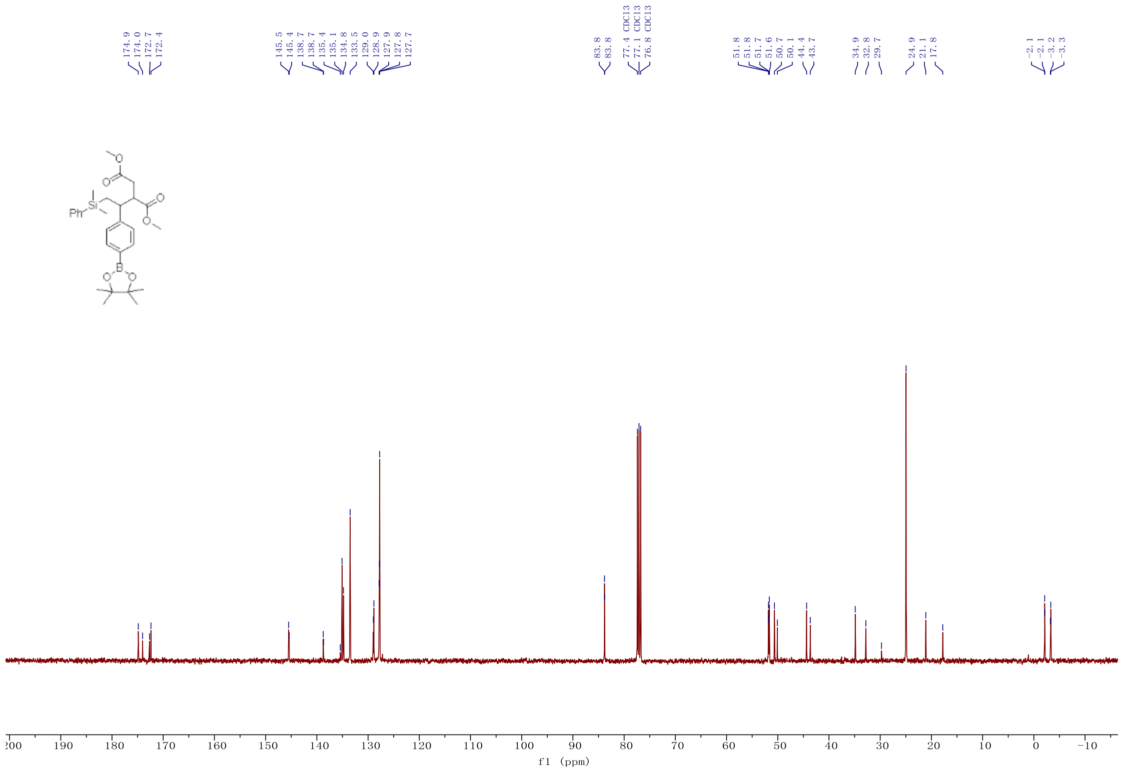

^{1}H NMR spectra of compound **d-9** in $CDCl_3$ (400 MHz): (*see procedure*)

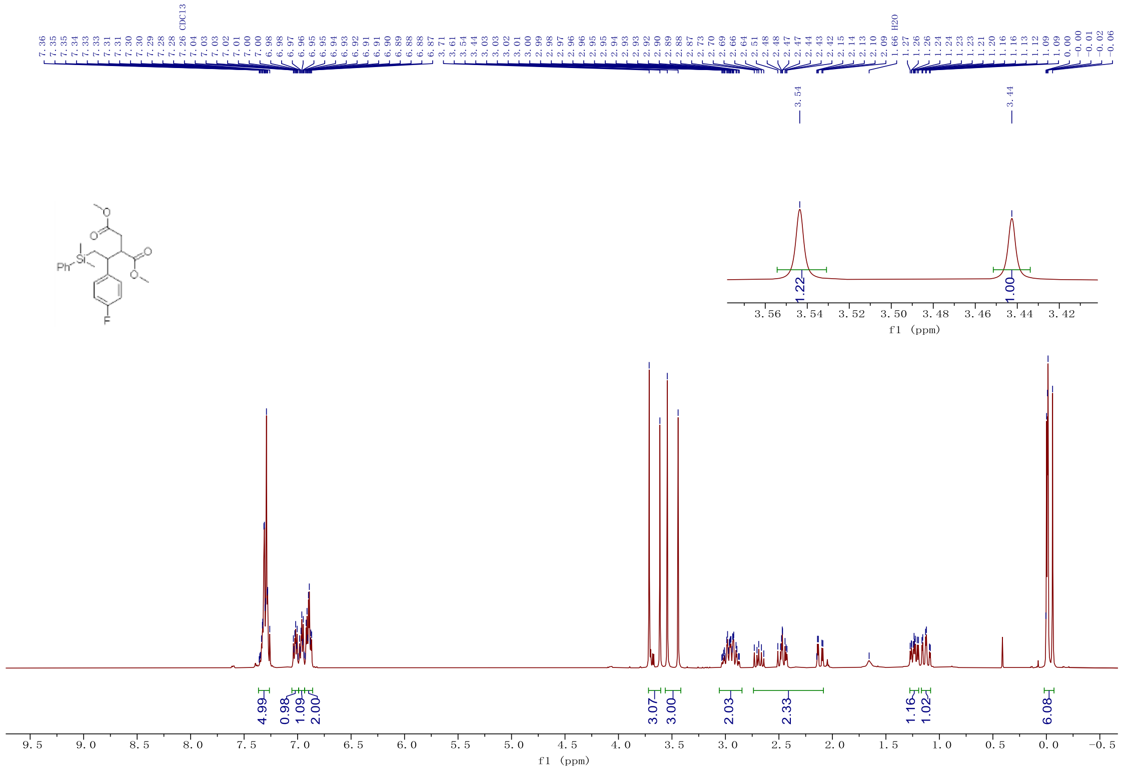

^{13}C NMR spectra of compound **d-9** in $CDCl_3$ (101 MHz): (*see procedure*)

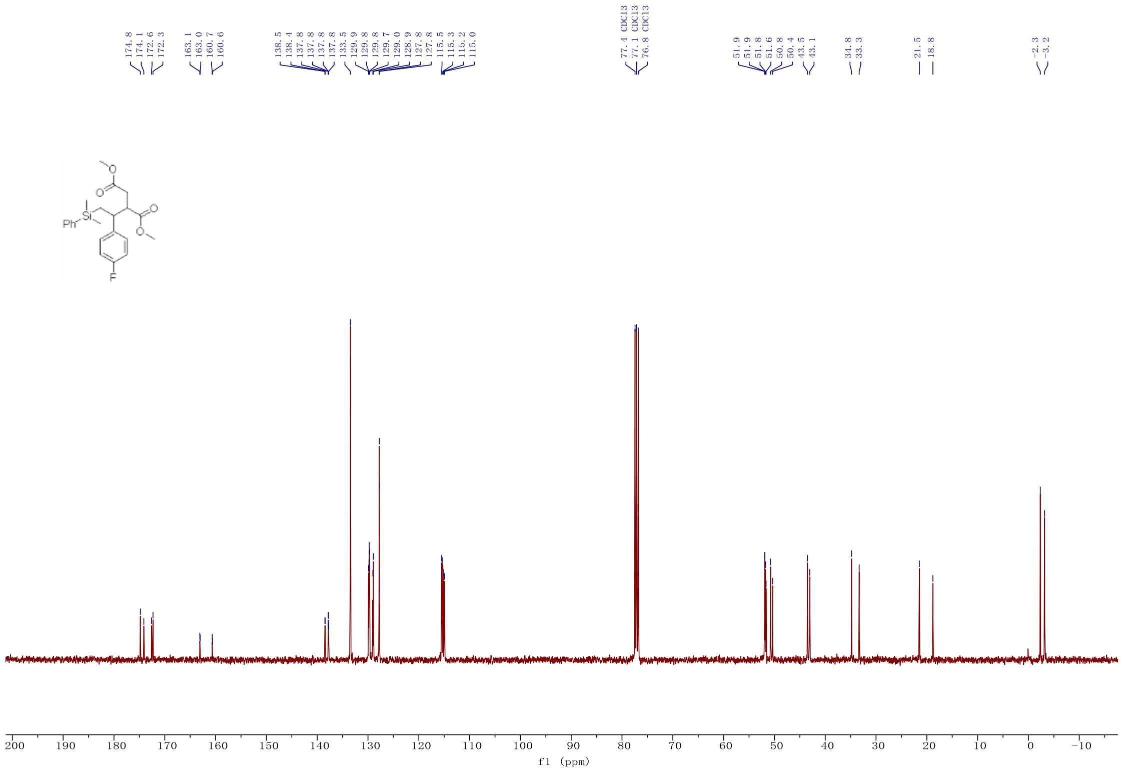

^{19}F NMR spectra of compound **d-9** in $CDCl_3$ (101 MHz): (*see procedure*)

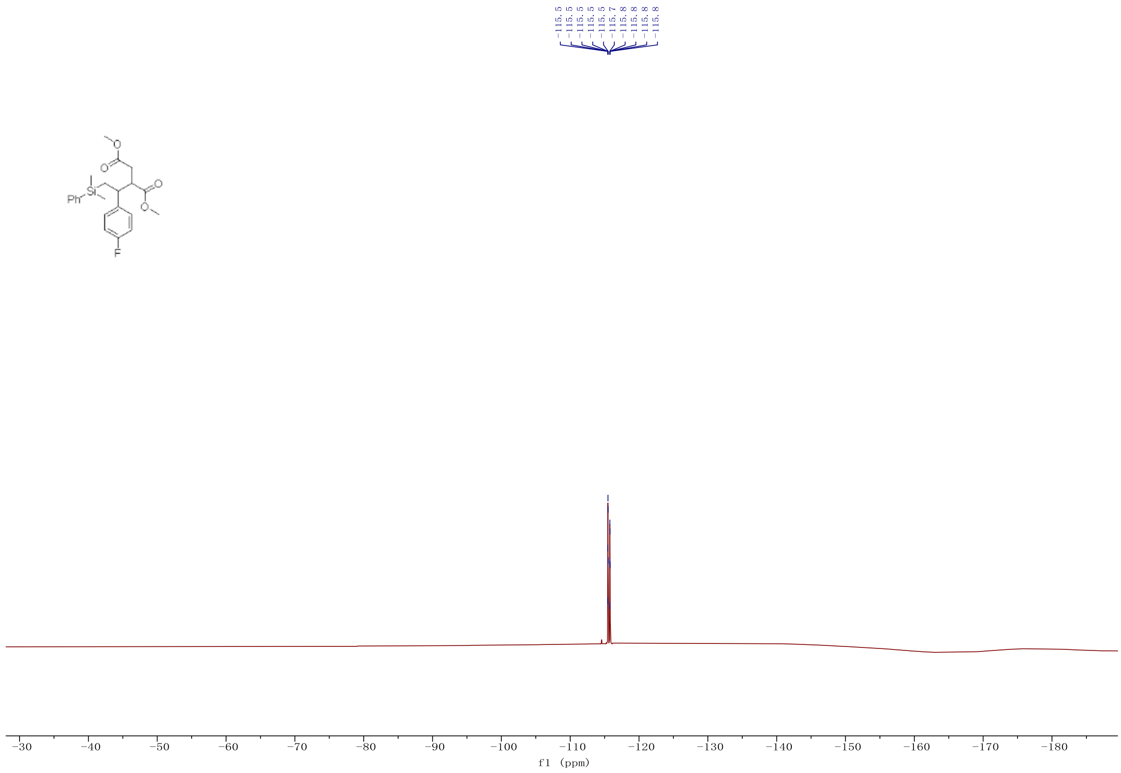

^{1}H NMR spectra of compound **d-10** in $CDCl_3$ (400 MHz): (*see procedure*)

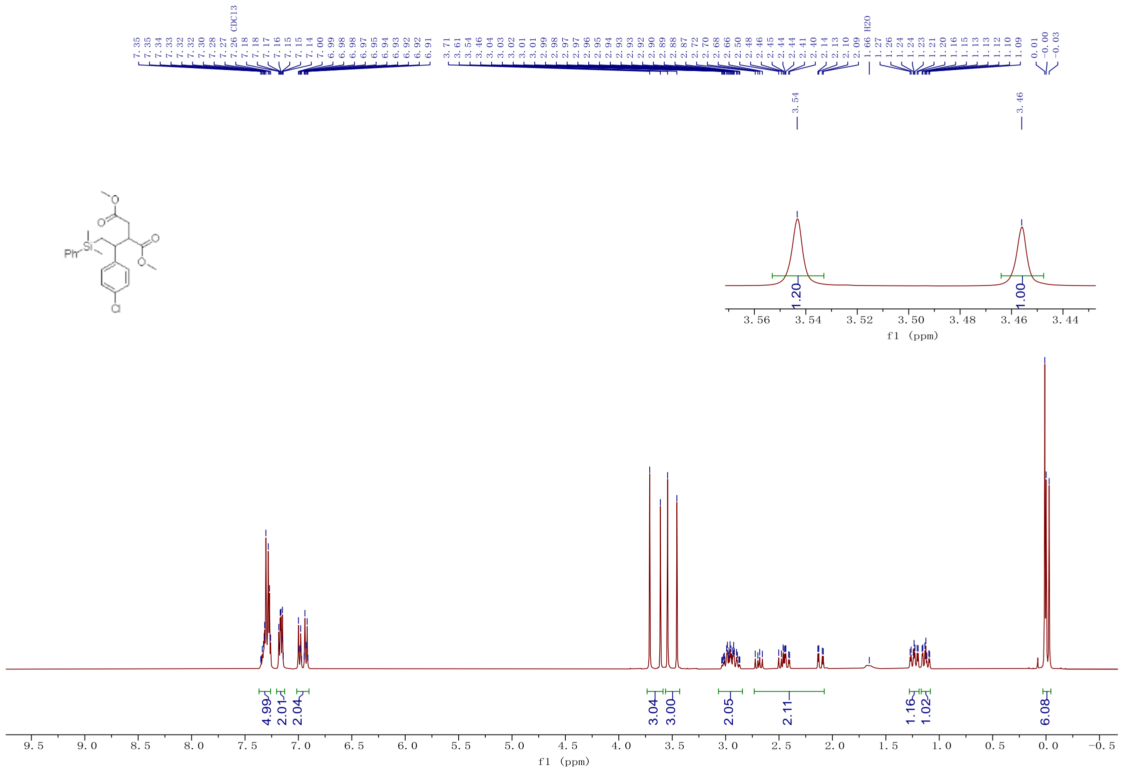

^{13}C NMR spectra of compound **d-10** in $CDCl_3$ (101 MHz): (*see procedure*)

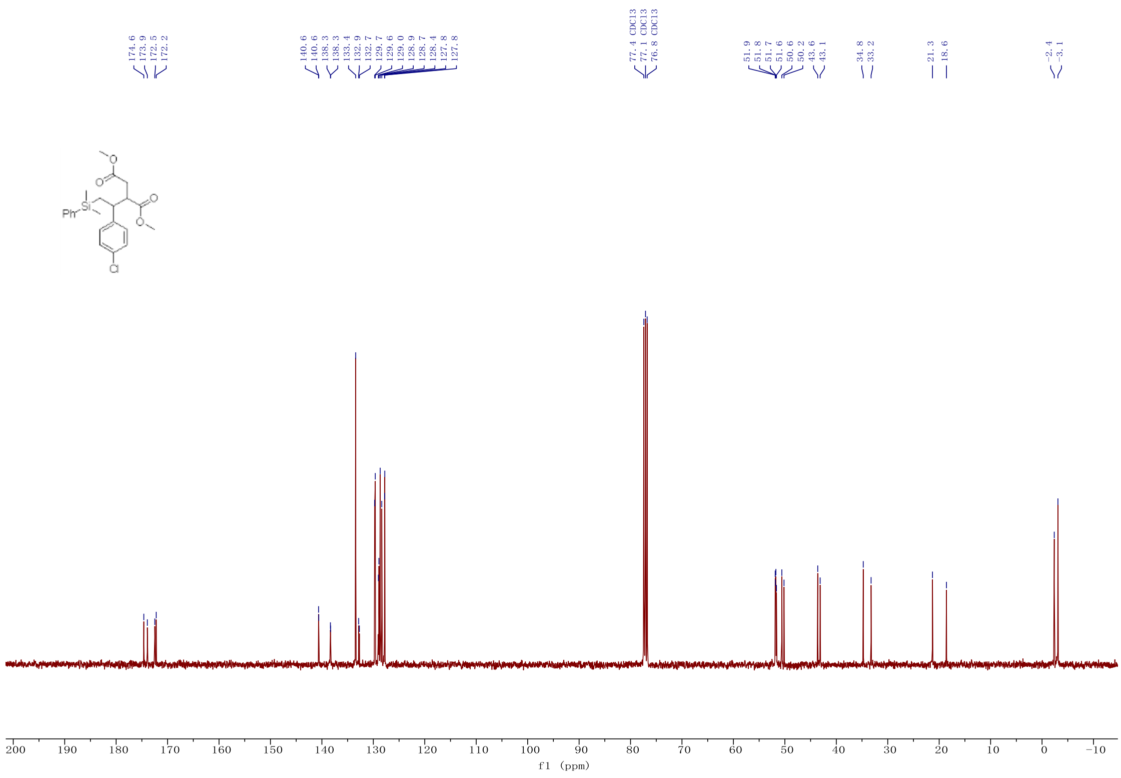

^{1}H NMR spectra of compound **d-11** in $CDCl_3$ (400 MHz): (*see procedure*)

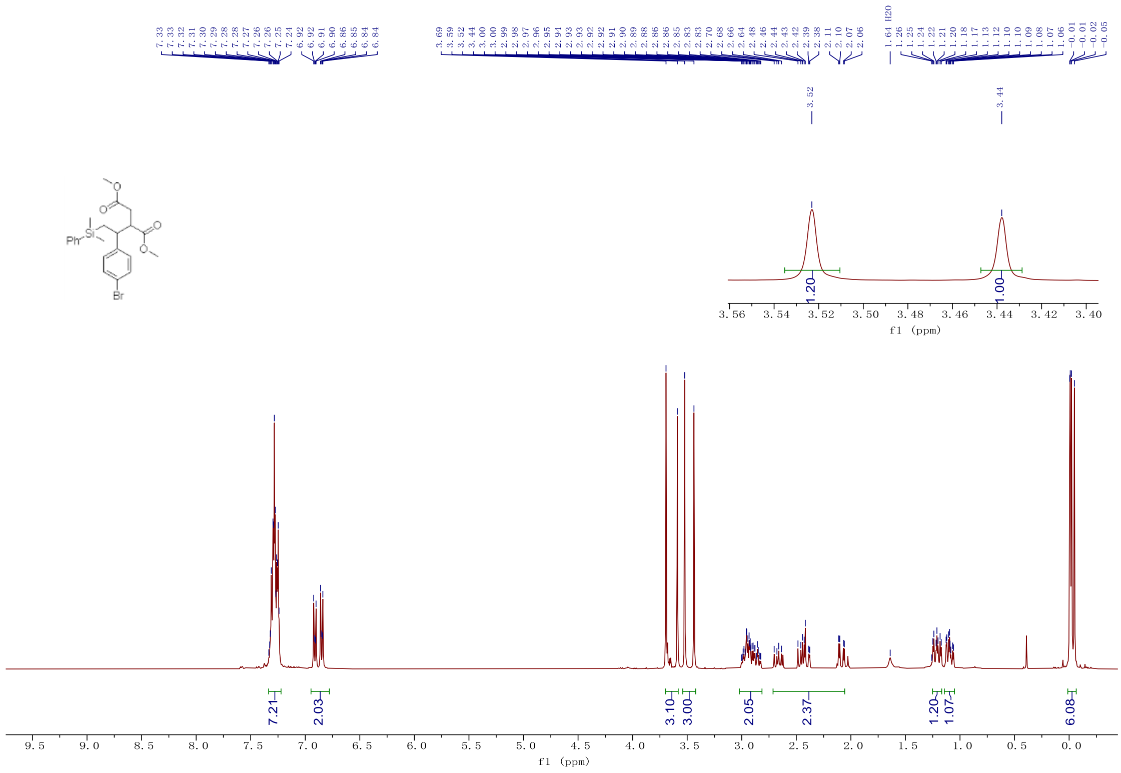

^{13}C NMR spectra of compound **d-11** in $CDCl_3$ (101 MHz): (*see procedure*)

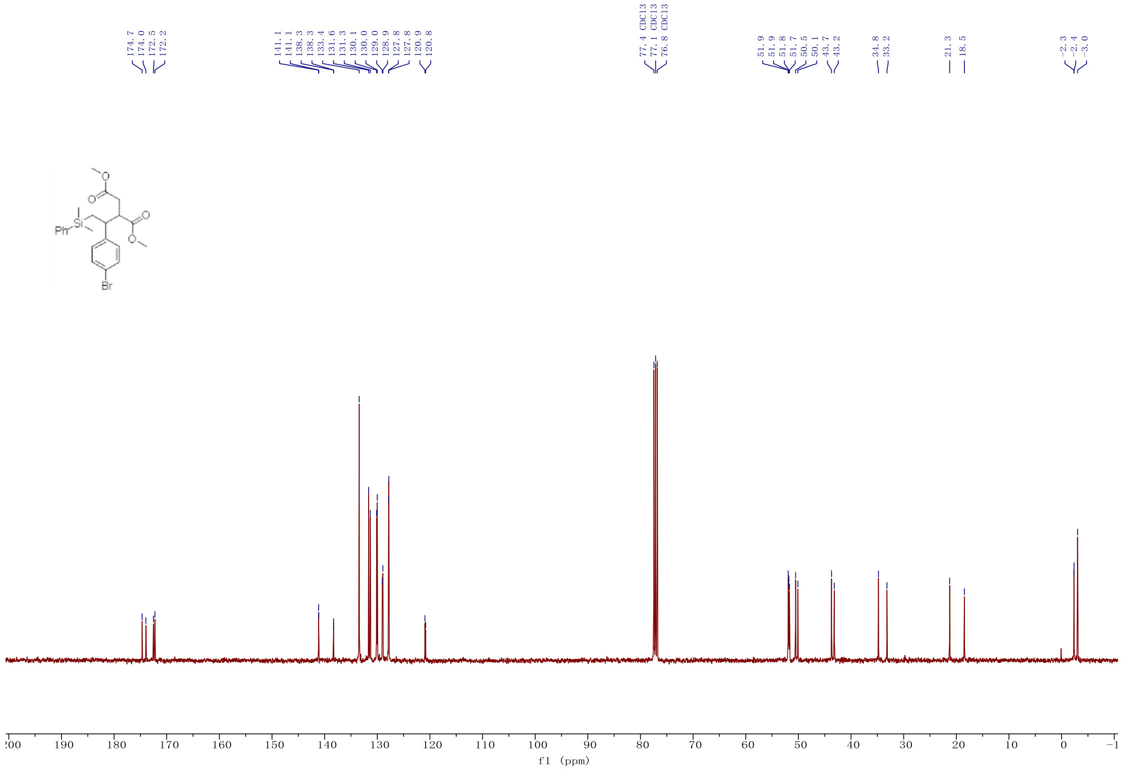

^{1}H NMR spectra of compound **d-12** in $CDCl_3$ (400 MHz): (*see procedure*)

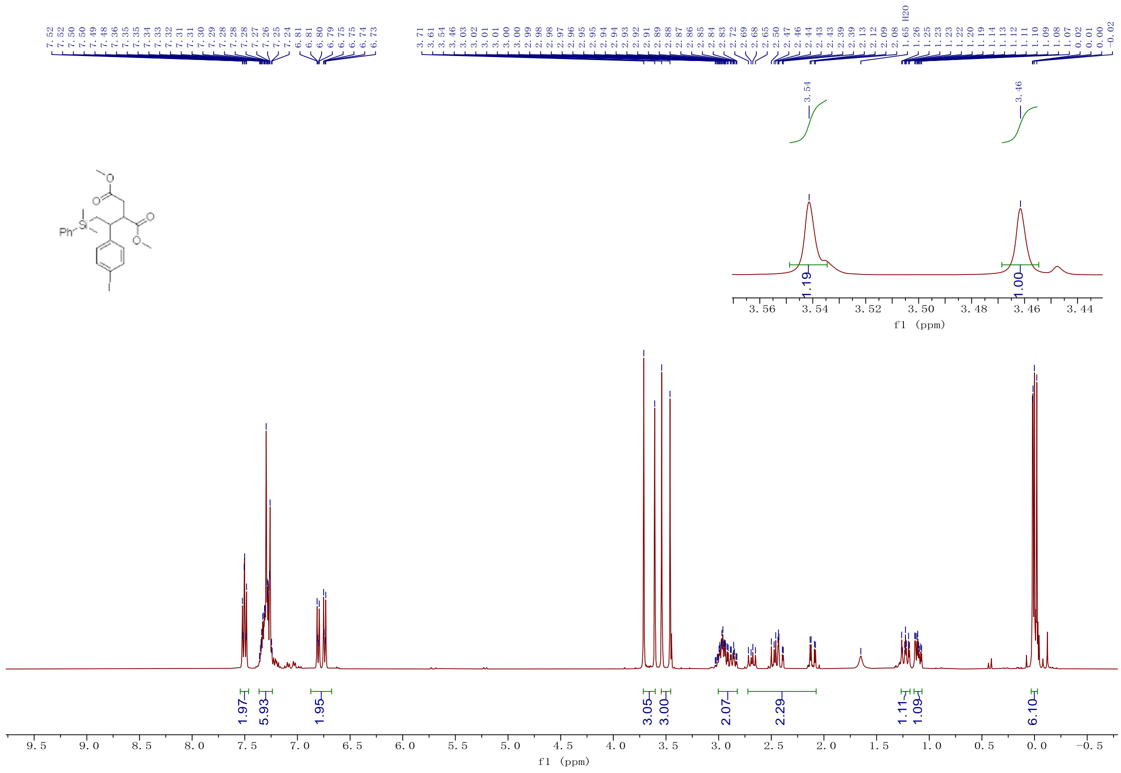

^{13}C NMR spectra of compound **d-12** in $CDCl_3$ (101 MHz): (<u>*see procedure*</u>)

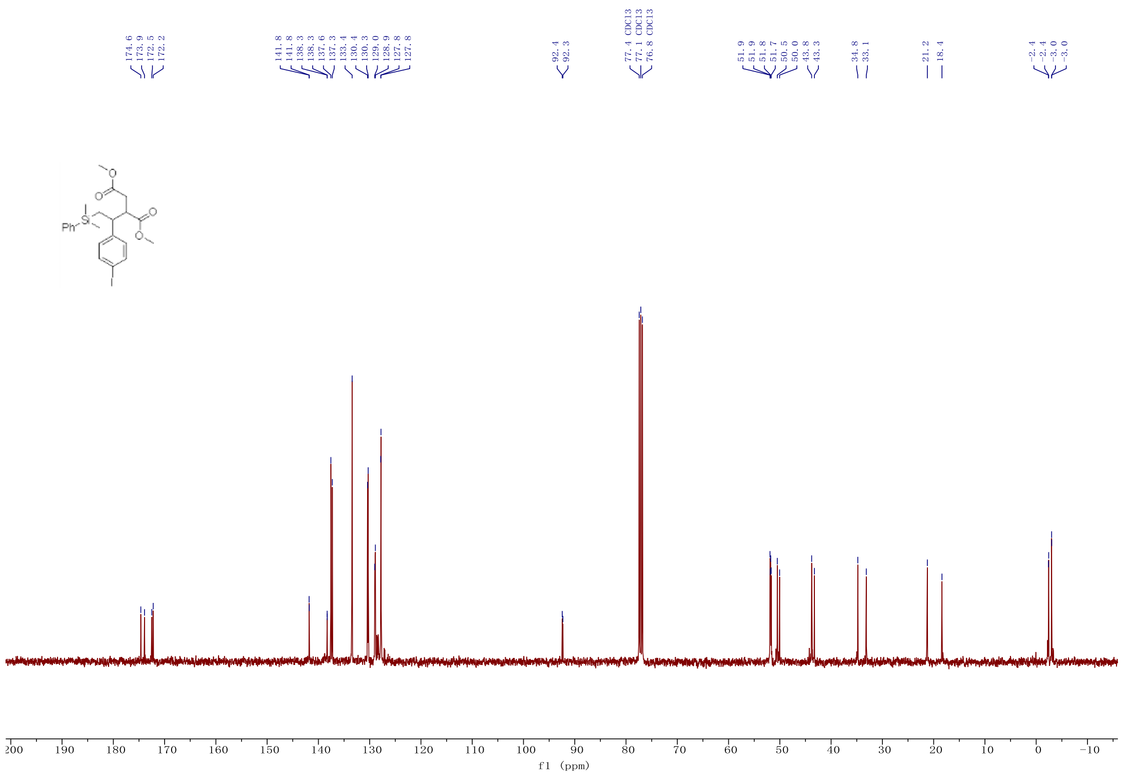

^{1}H NMR spectra of compound **d-13** in $CDCl_3$ (400 MHz): (<u>*see procedure*</u>)

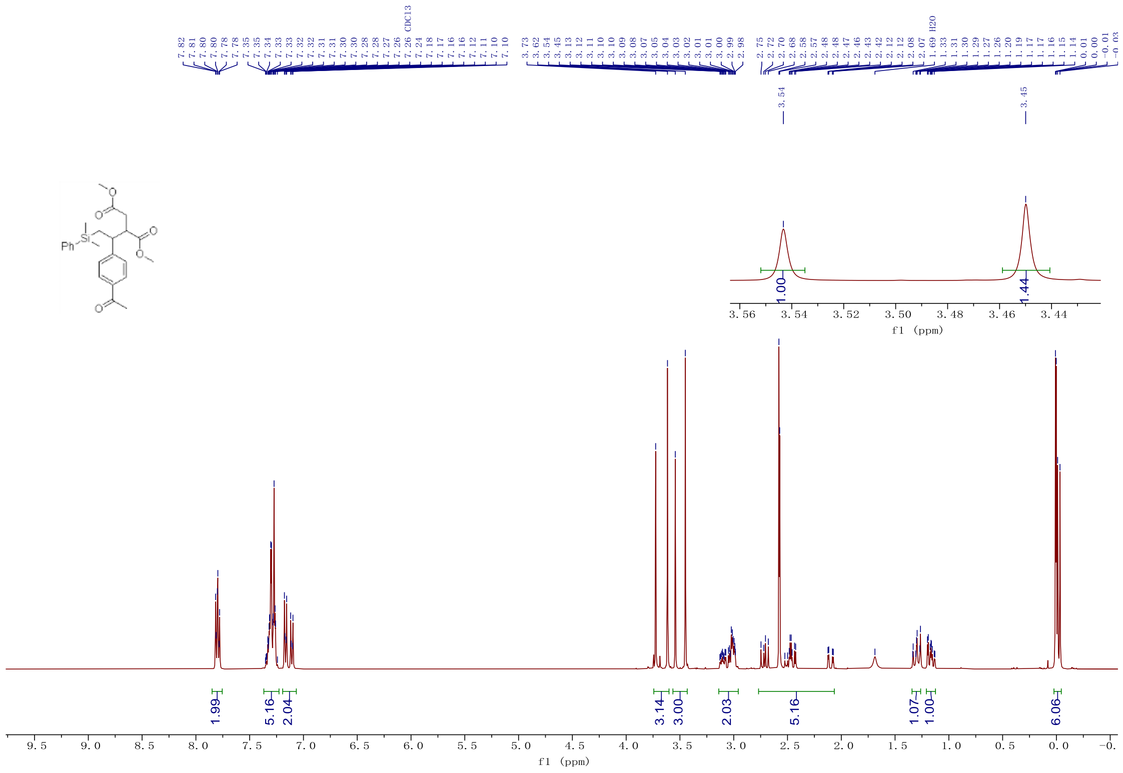

^{13}C NMR spectra of compound **d-13** in $CDCl_3$ (101 MHz): (see procedure)

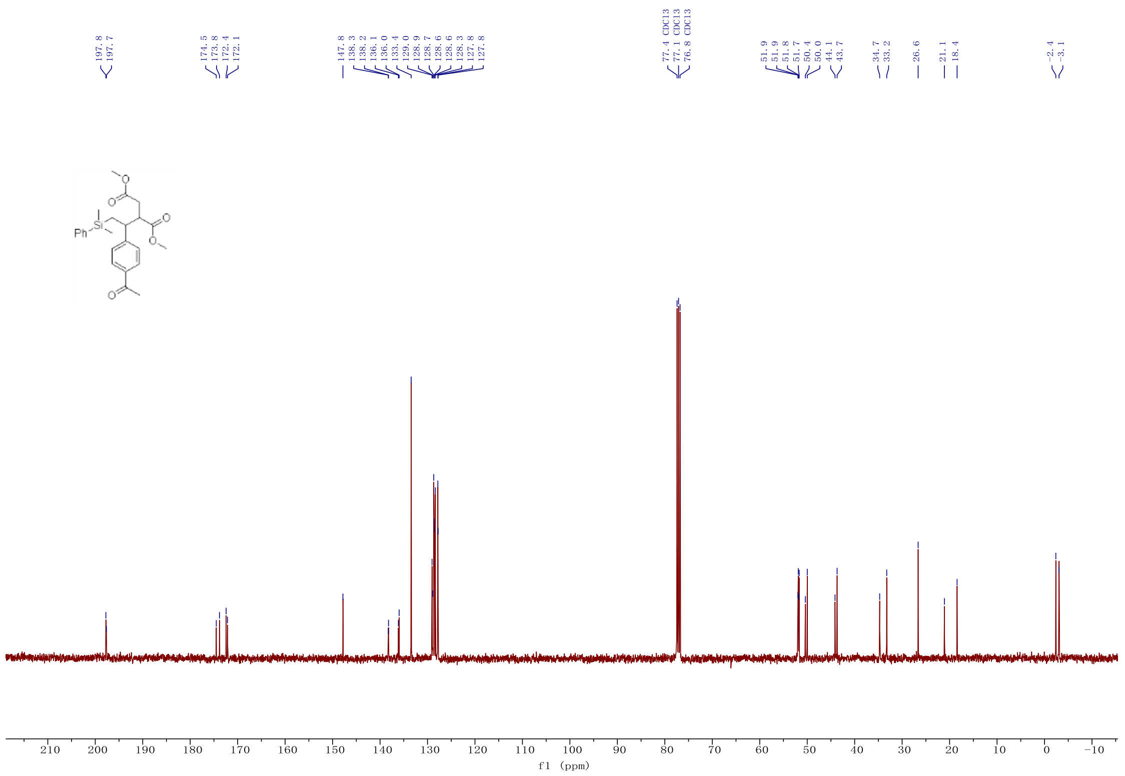

^{1}H NMR spectra of compound **d-14** in $CDCl_3$ (400 MHz): (see procedure)

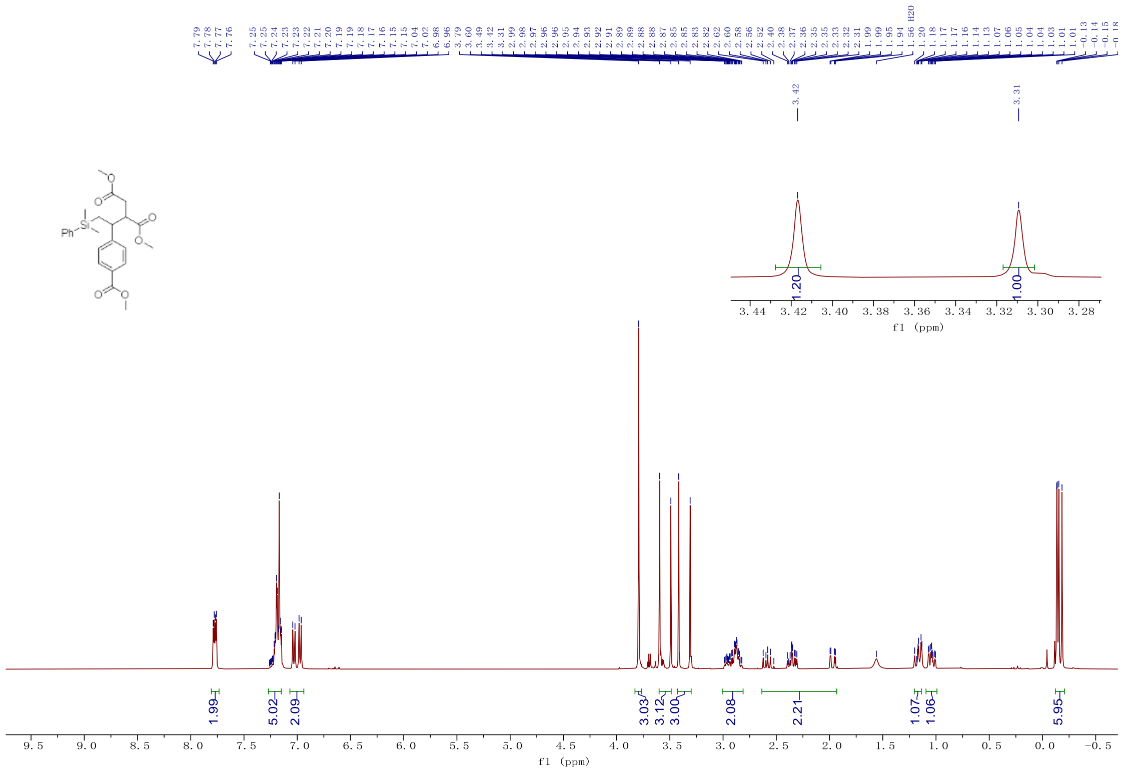

^{13}C NMR spectra of compound **d-14** in $CDCl_3$ (101 MHz): (*see procedure*)

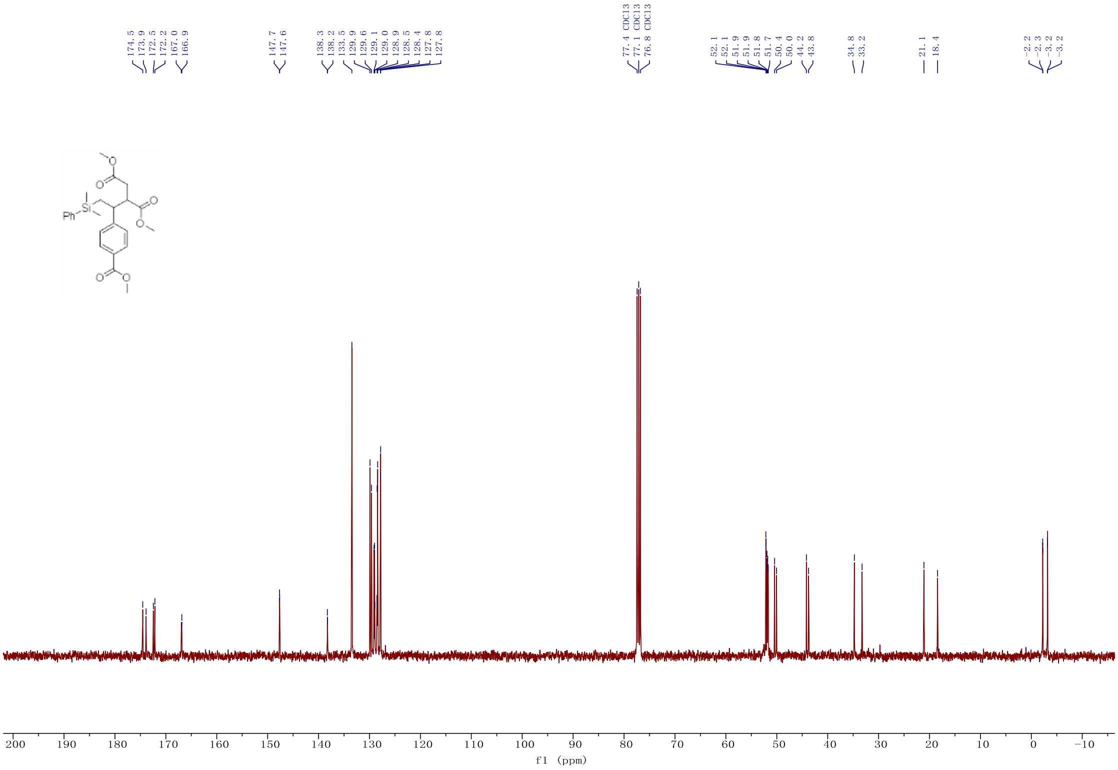

^{1}H NMR spectra of compound **d-15** in $CDCl_3$ (400 MHz): (*see procedure*)

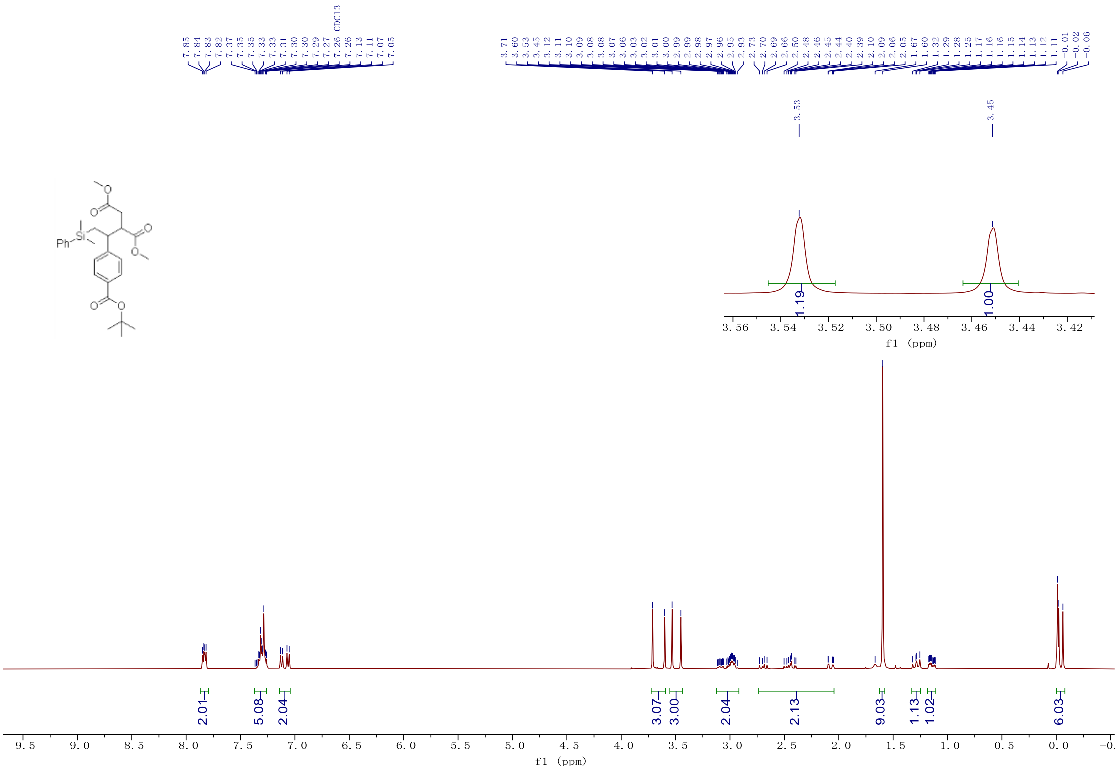

^{13}C NMR spectra of compound **d-15** in $CDCl_3$ (101 MHz): (*see procedure*)

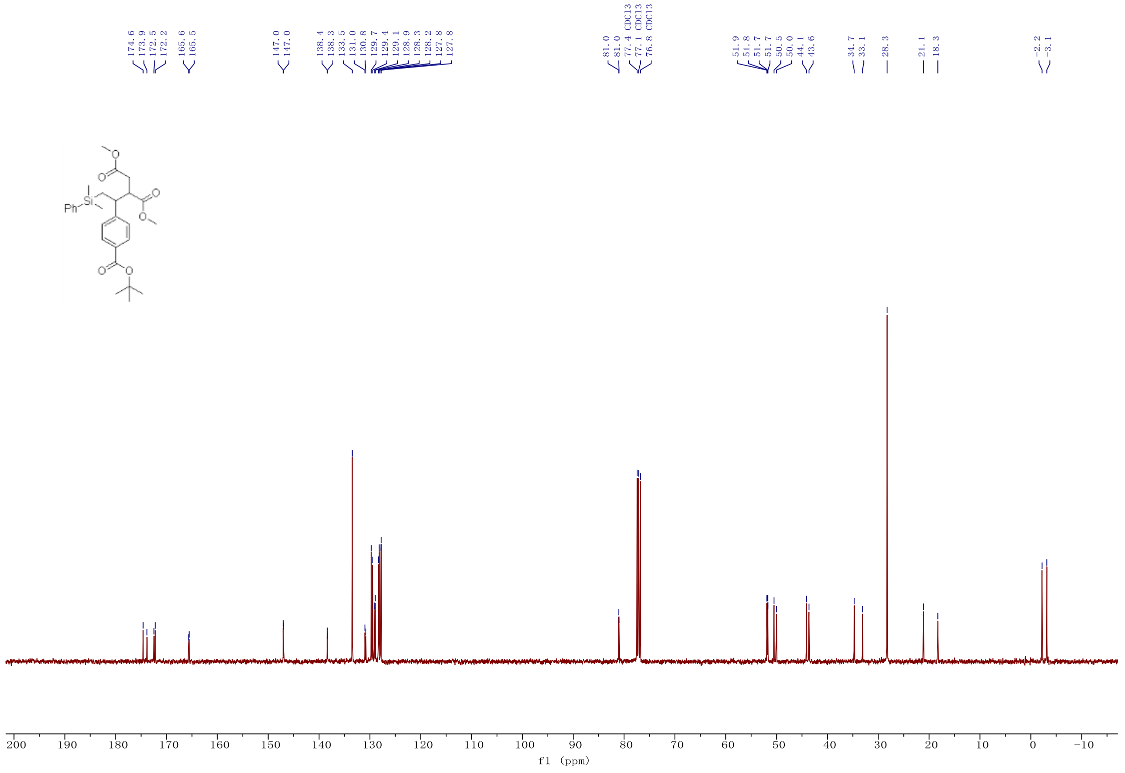

^{1}H NMR spectra of compound **d-16** in $CDCl_3$ (400 MHz): (*see procedure*)

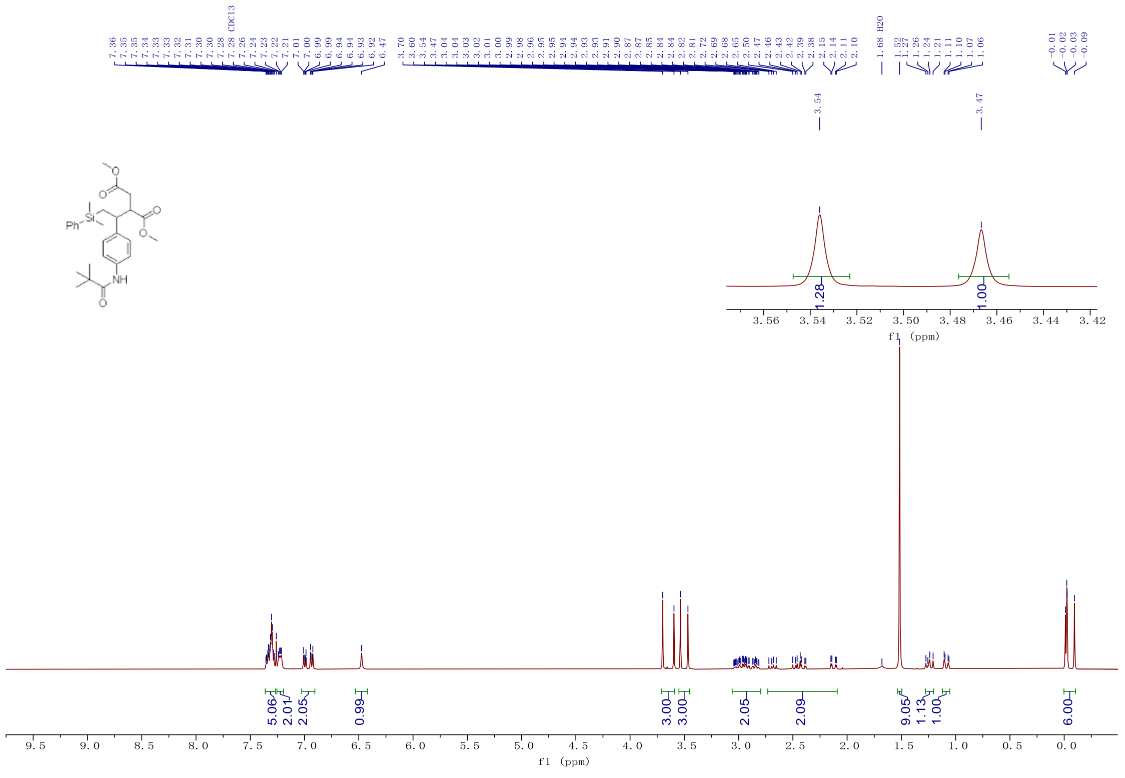

^{13}C NMR spectra of compound **d-16** in $CDCl_3$ (101 MHz): (<u>*see procedure*</u>)

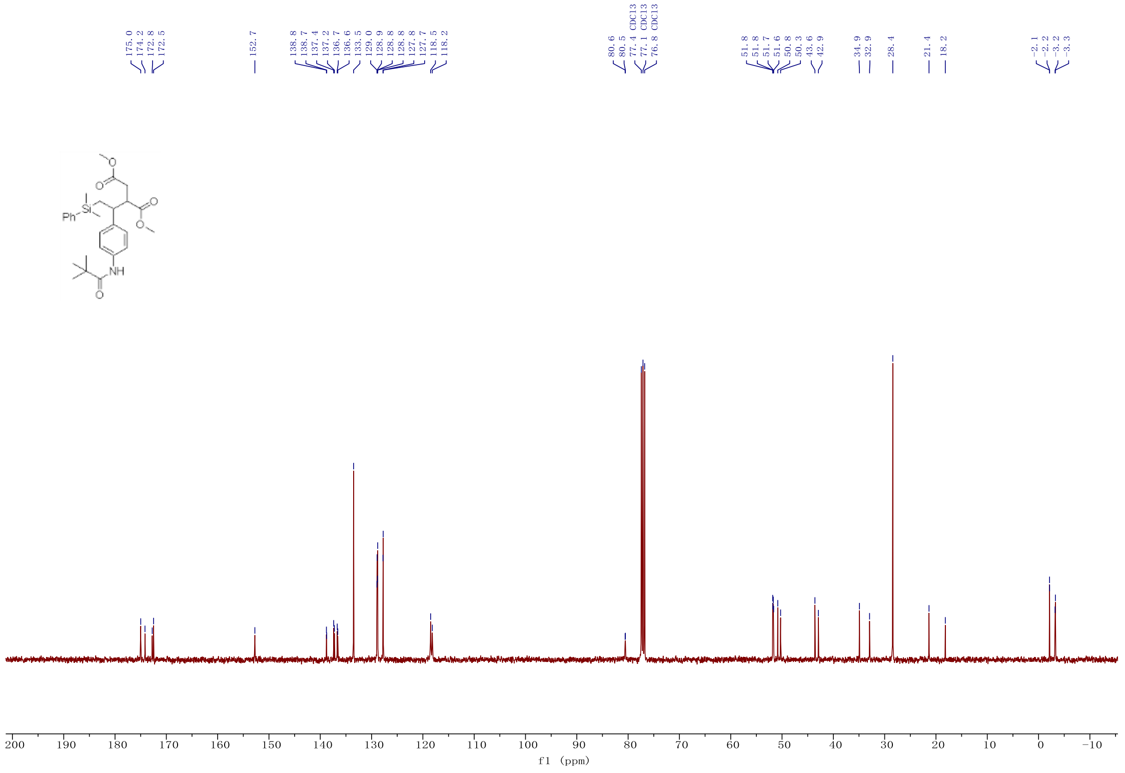

^{1}H NMR spectra of compound **d-17** in $CDCl_3$ (400 MHz): (<u>*see procedure*</u>)

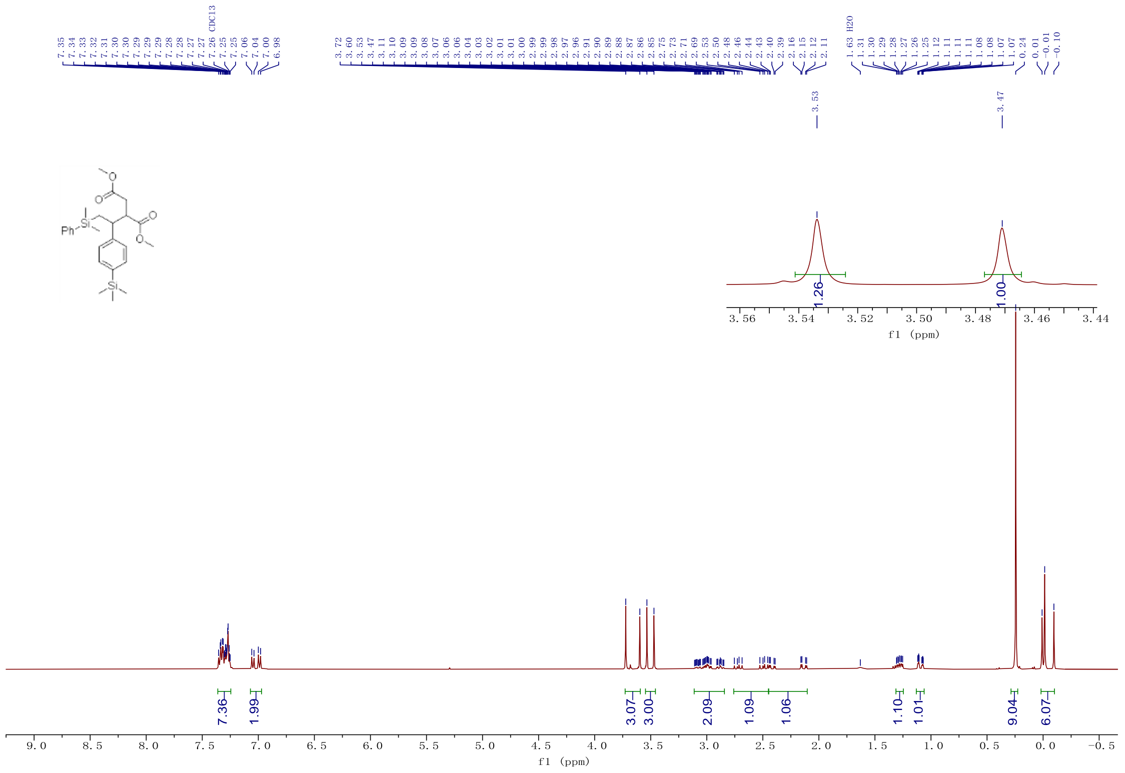

^{13}C NMR spectra of compound **d-17** in $CDCl_3$ (101 MHz): (*see procedure*)

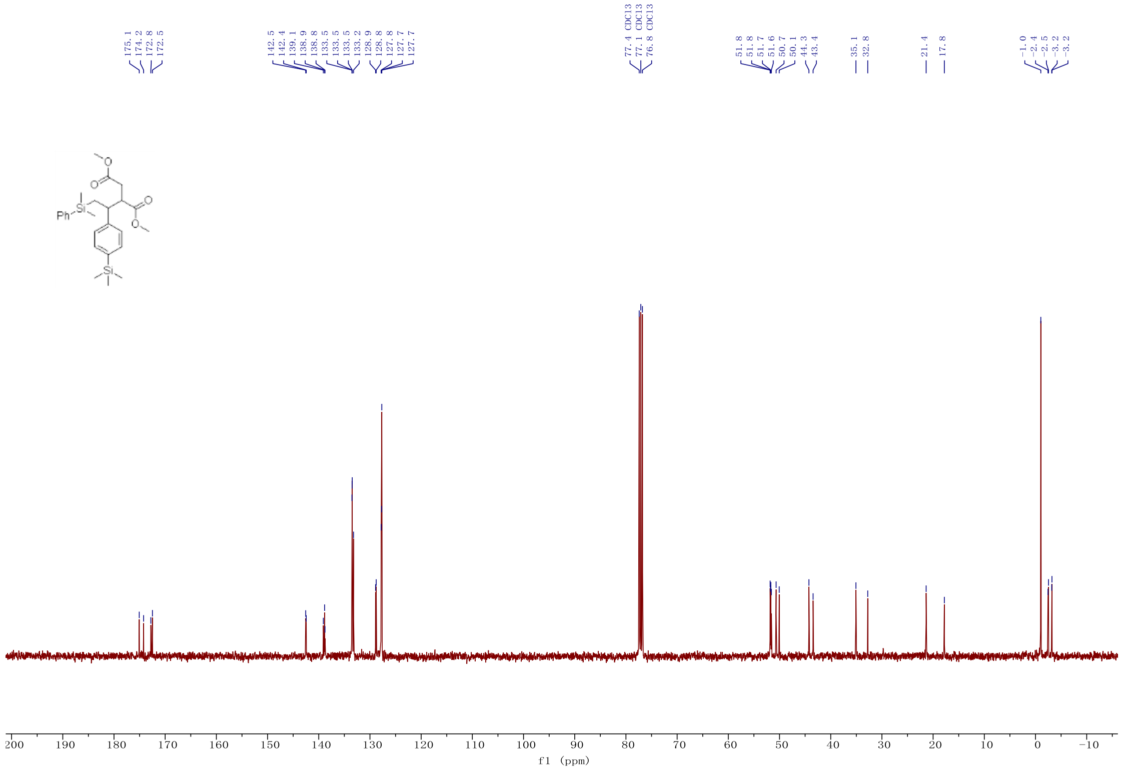

^{1}H NMR spectra of compound **d-18** in $CDCl_3$ (400 MHz): (*see procedure*)

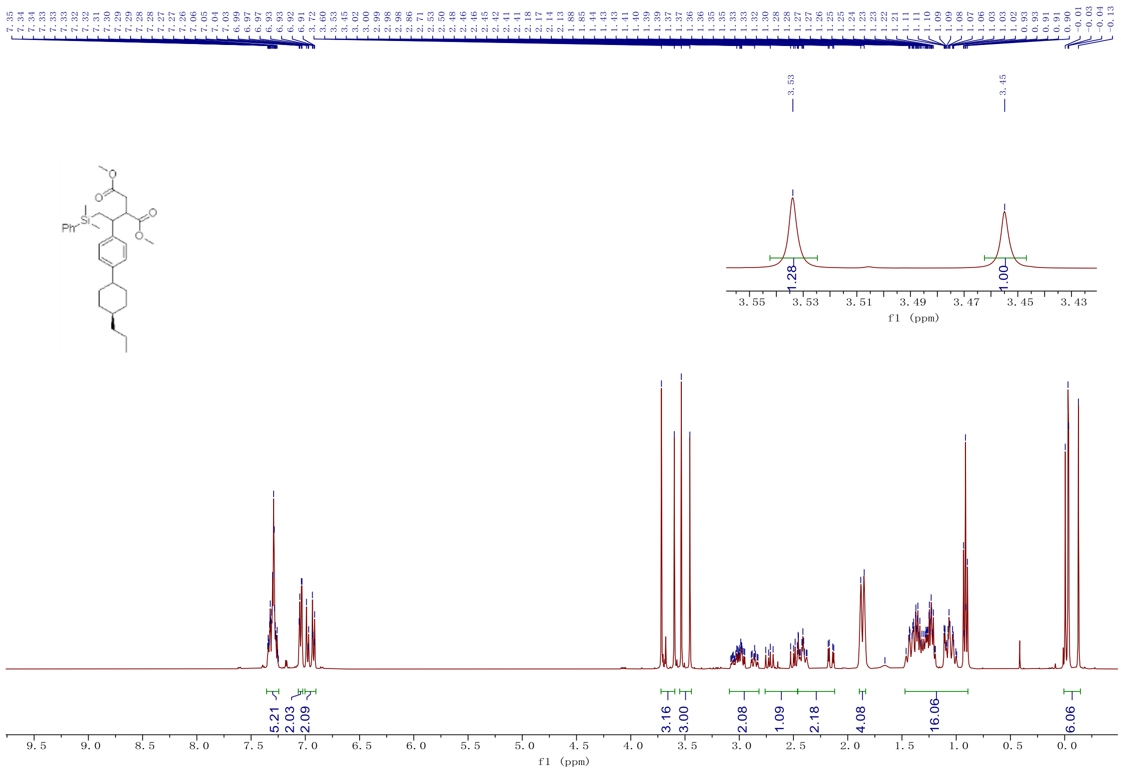

^{13}C NMR spectra of compound **d-18** in $CDCl_3$ (101 MHz): (<u>*see procedure*</u>)

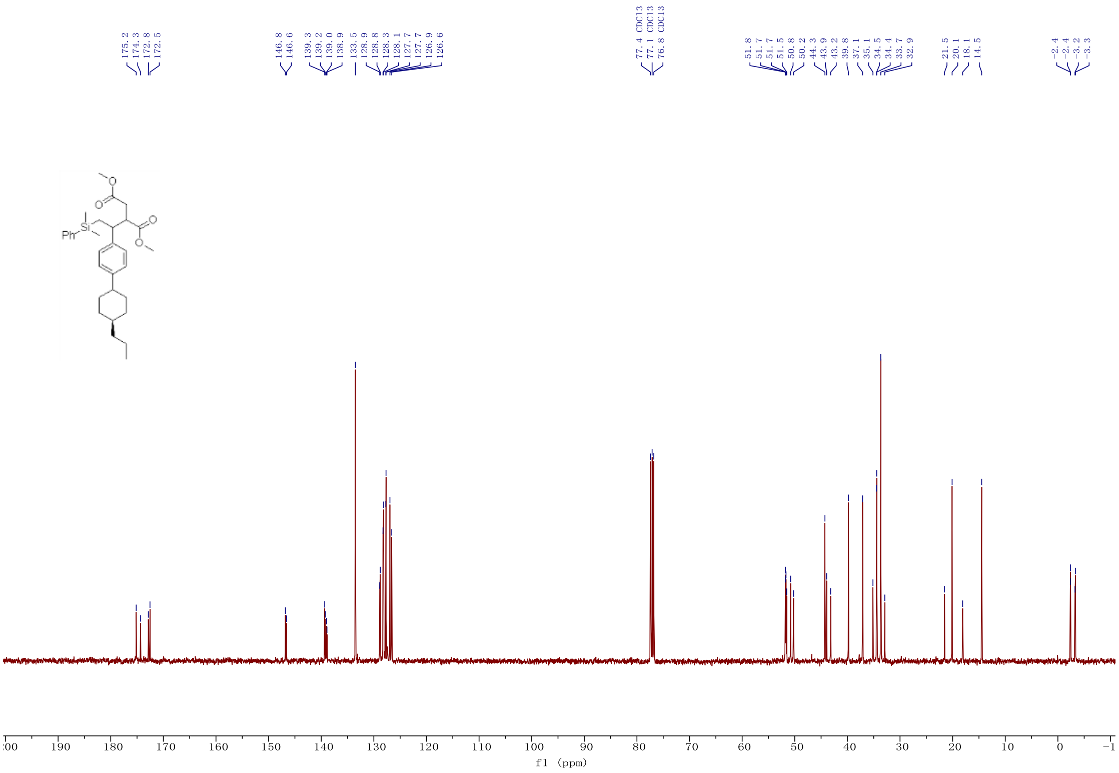

^{1}H NMR spectra of compound **d-19** in $CDCl_3$ (400 MHz): (<u>*see procedure*</u>)

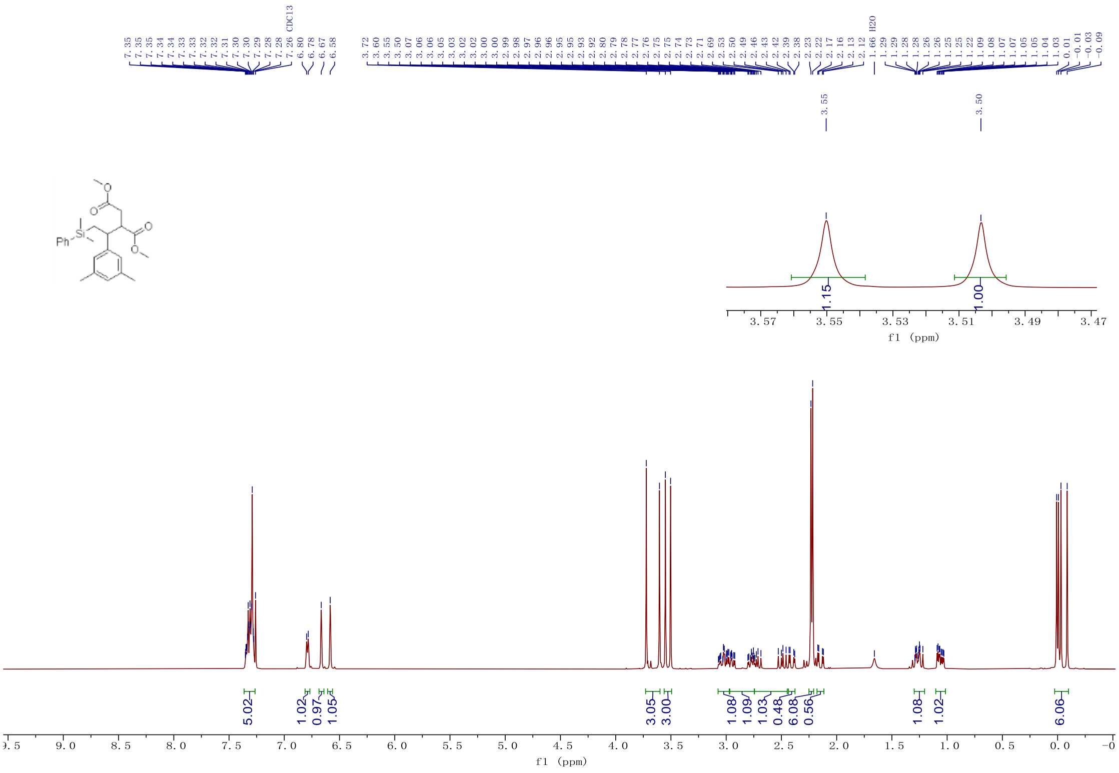

^{13}C NMR spectra of compound **d-19** in $CDCl_3$ (101 MHz): (*see procedure*)

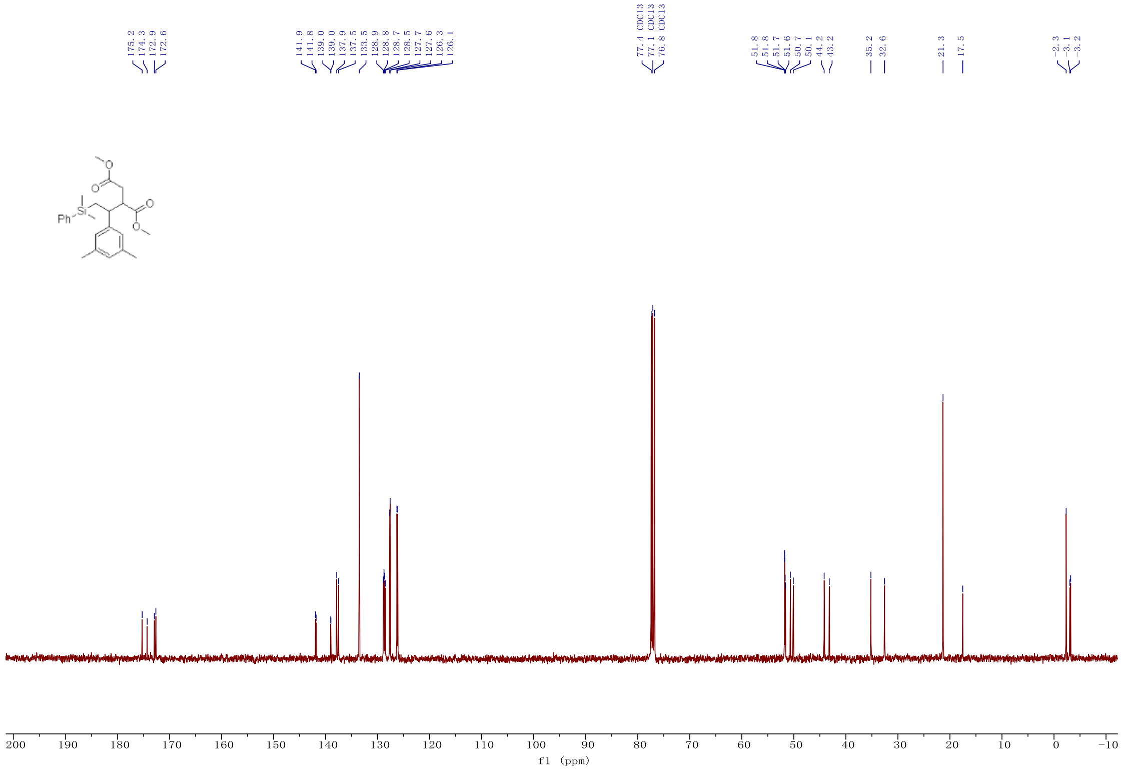

^{1}H NMR spectra of compound **d-20** in $CDCl_3$ (400 MHz): (*see procedure*)

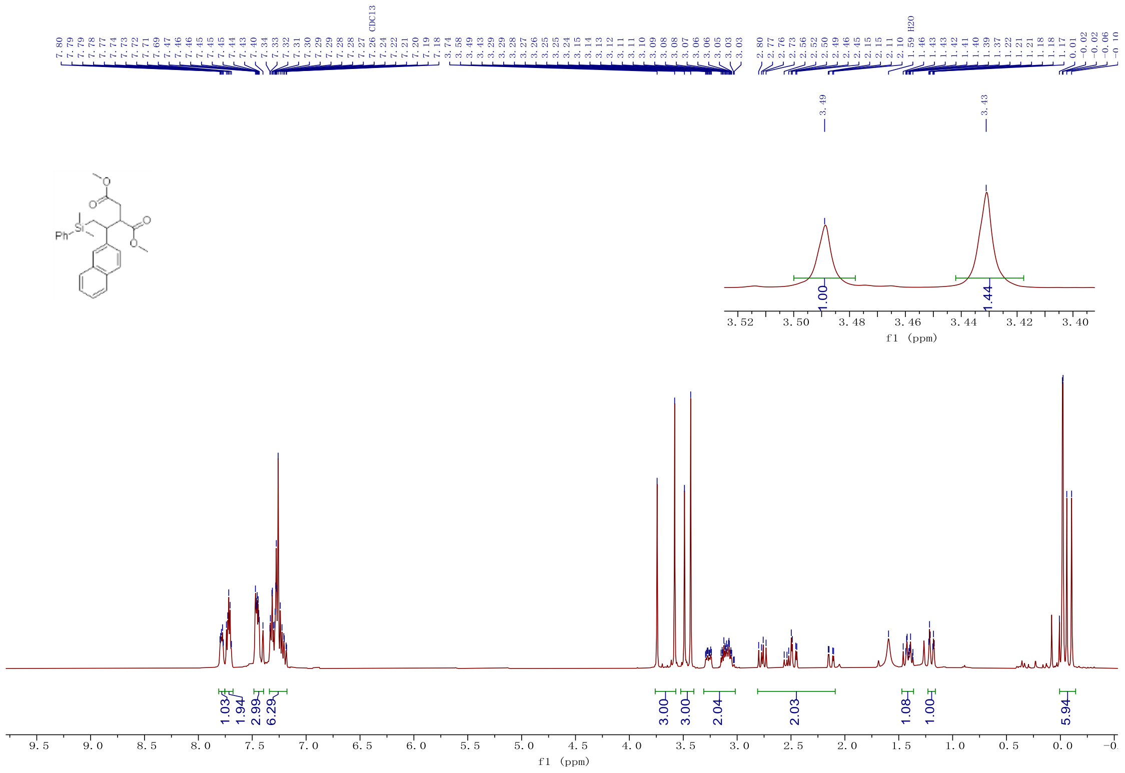

^{13}C NMR spectra of compound **d-20** in $CDCl_3$ (101 MHz): (see procedure)

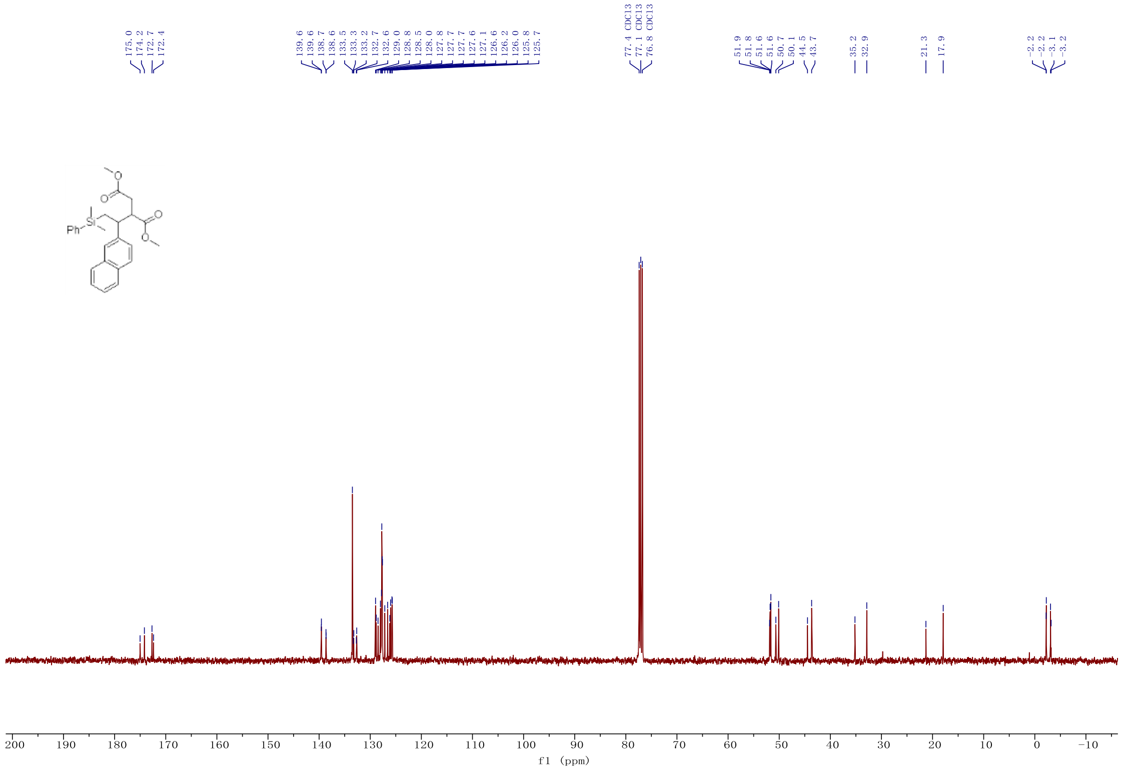

^{1}H NMR spectra of compound **d-21** in $CDCl_3$ (400 MHz): (see procedure)

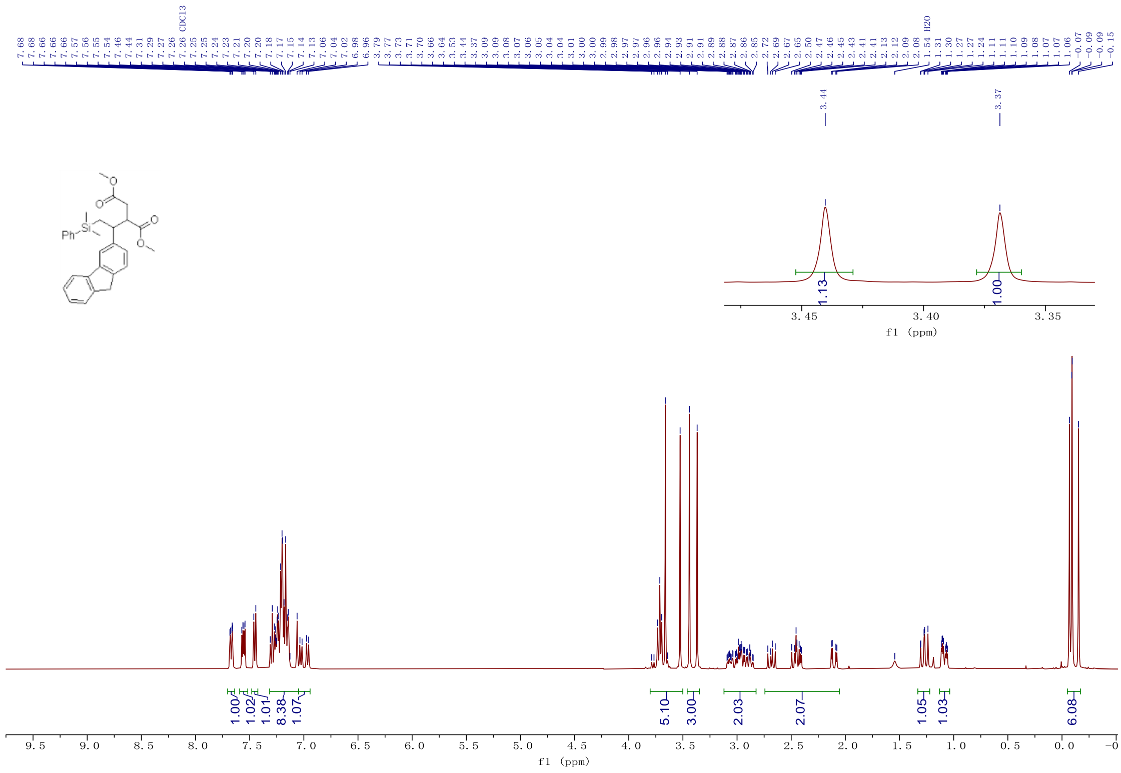

^{13}C NMR spectra of compound **d-21** in $CDCl_3$ (101 MHz): (*see procedure*)

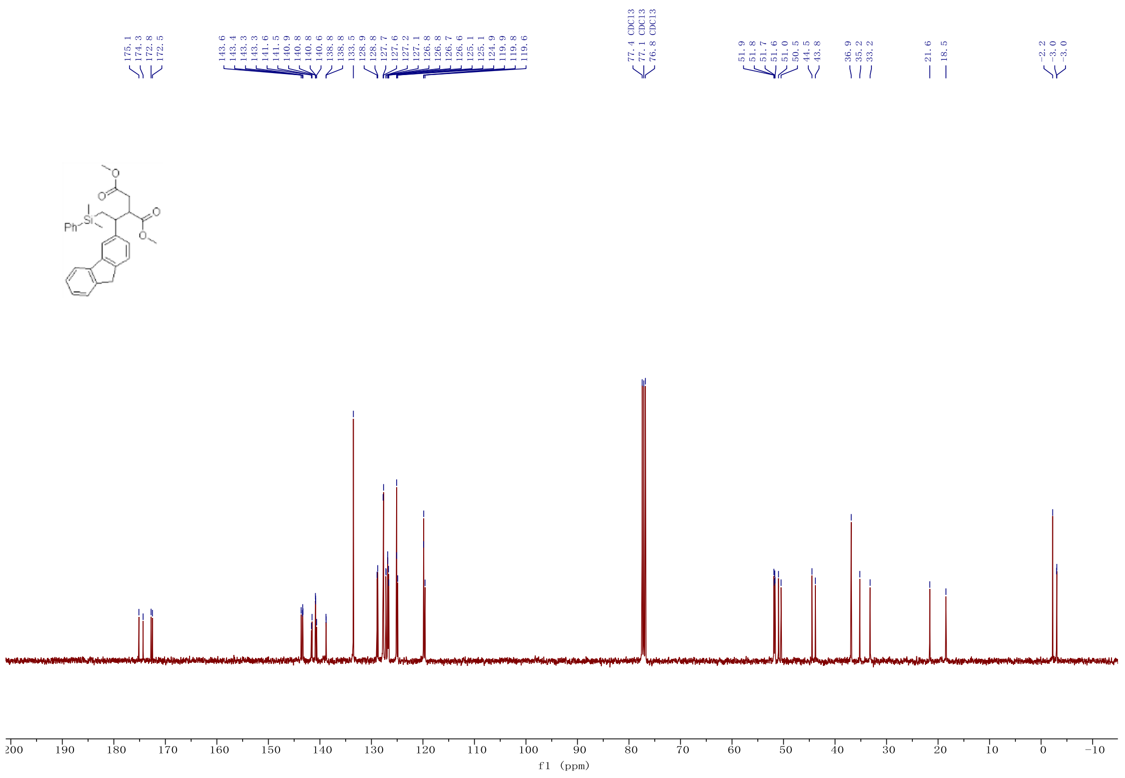

^{1}H NMR spectra of compound **d-22** in $CDCl_3$ (400 MHz): (*see procedure*)

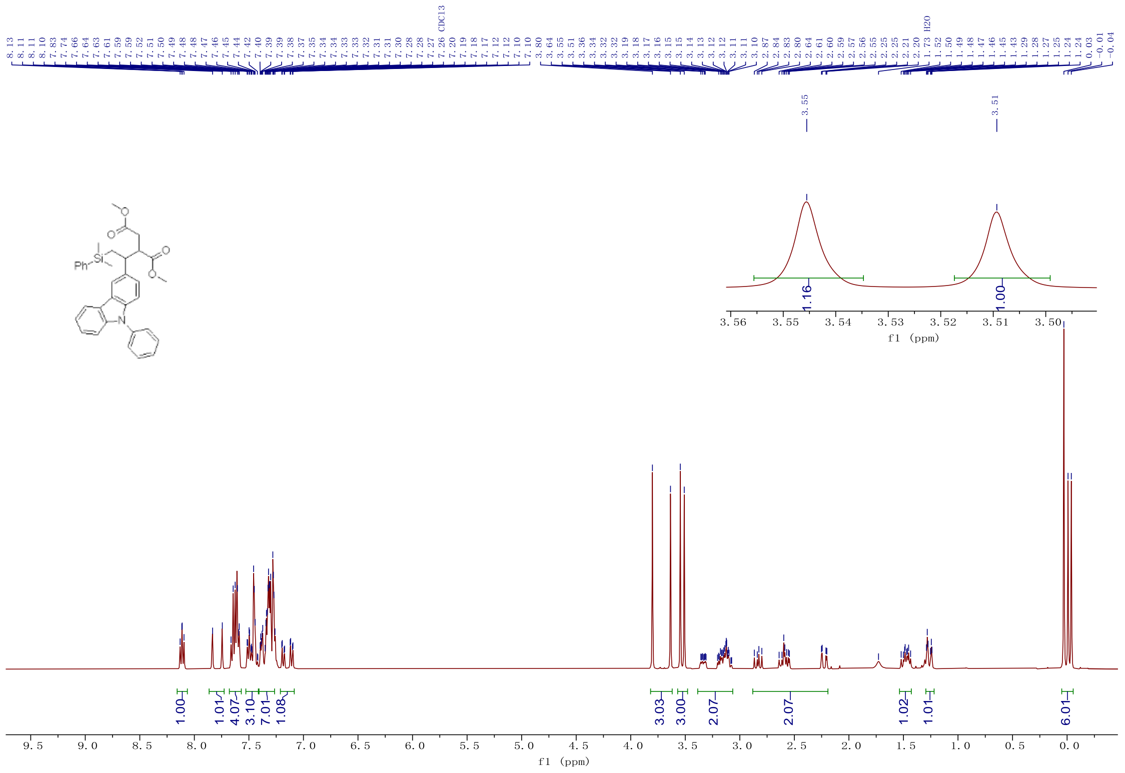

^{13}C NMR spectra of compound **d-22** in $CDCl_3$ (101 MHz): (*see procedure*)

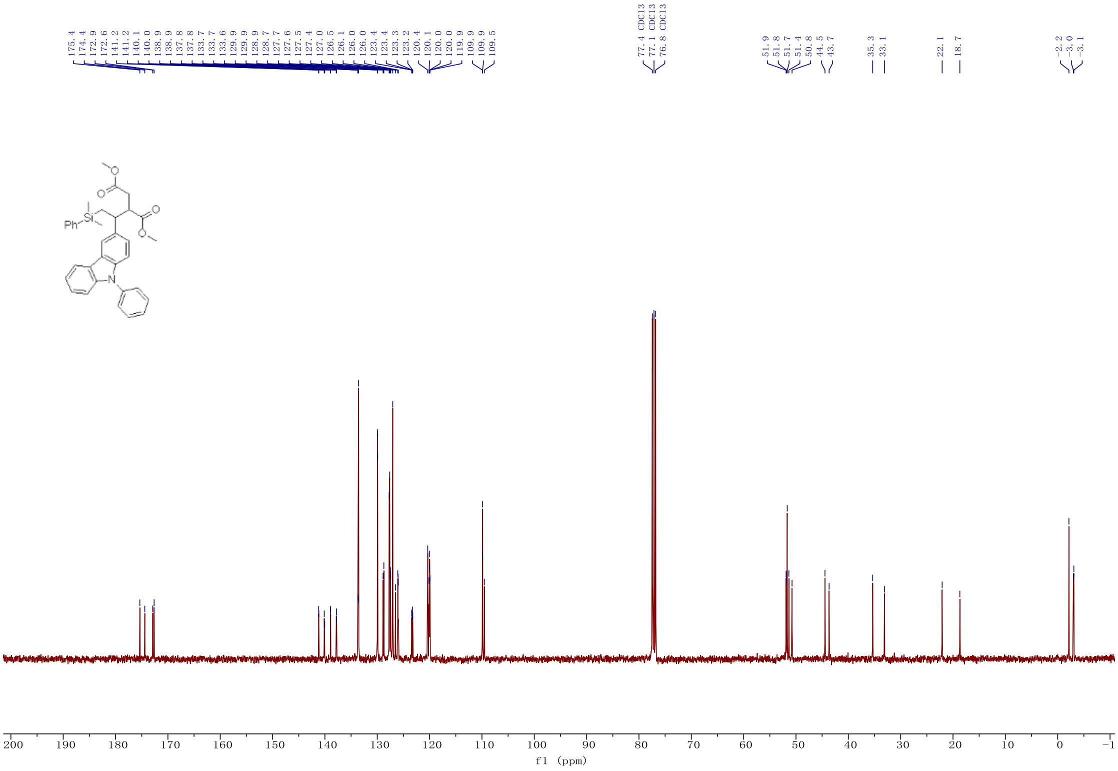

^{1}H NMR spectra of compound **d-23** in $CDCl_3$ (400 MHz): (*see procedure*)

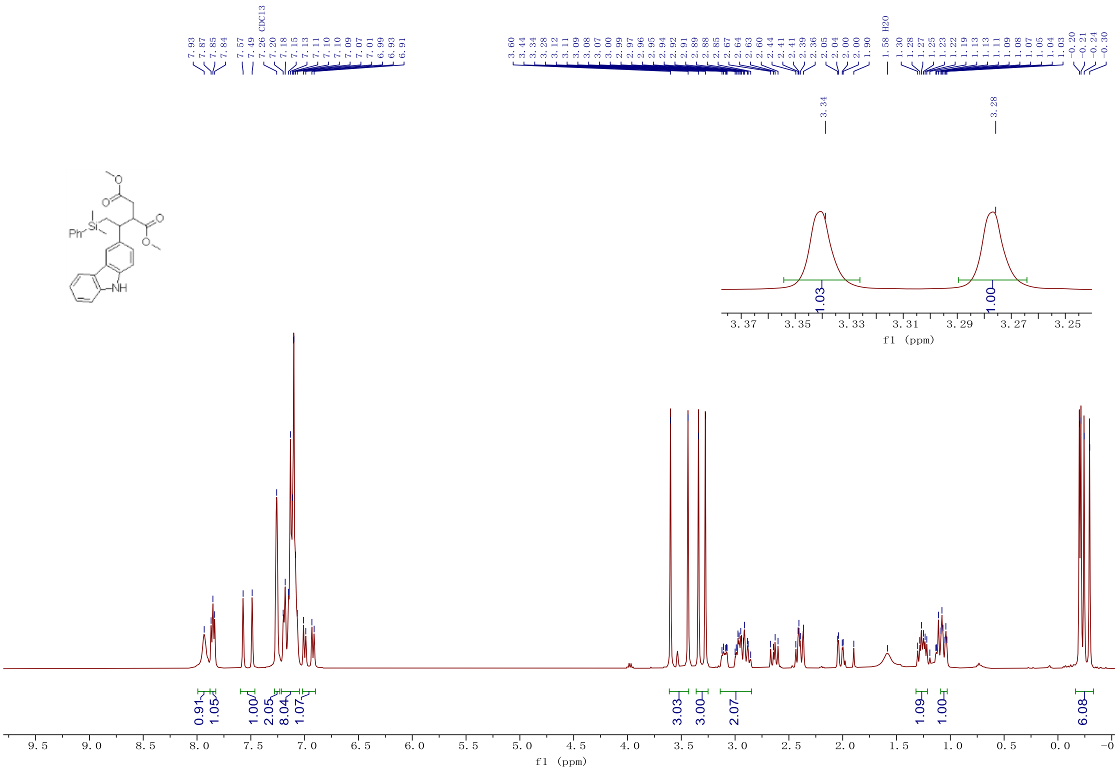

^{13}C NMR spectra of compound **d-23** in $CDCl_3$ (101 MHz): (*see procedure*)

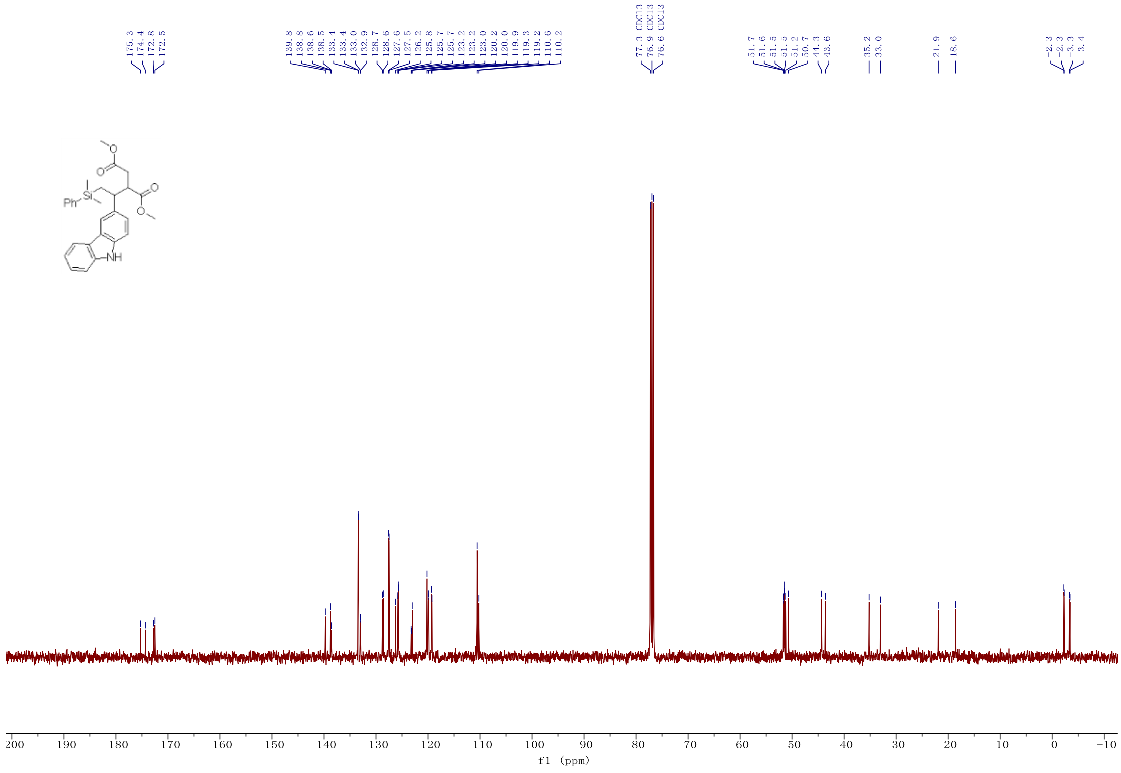

^{1}H NMR spectra of compound **d-24** in $CDCl_3$ (400 MHz): (*see procedure*)

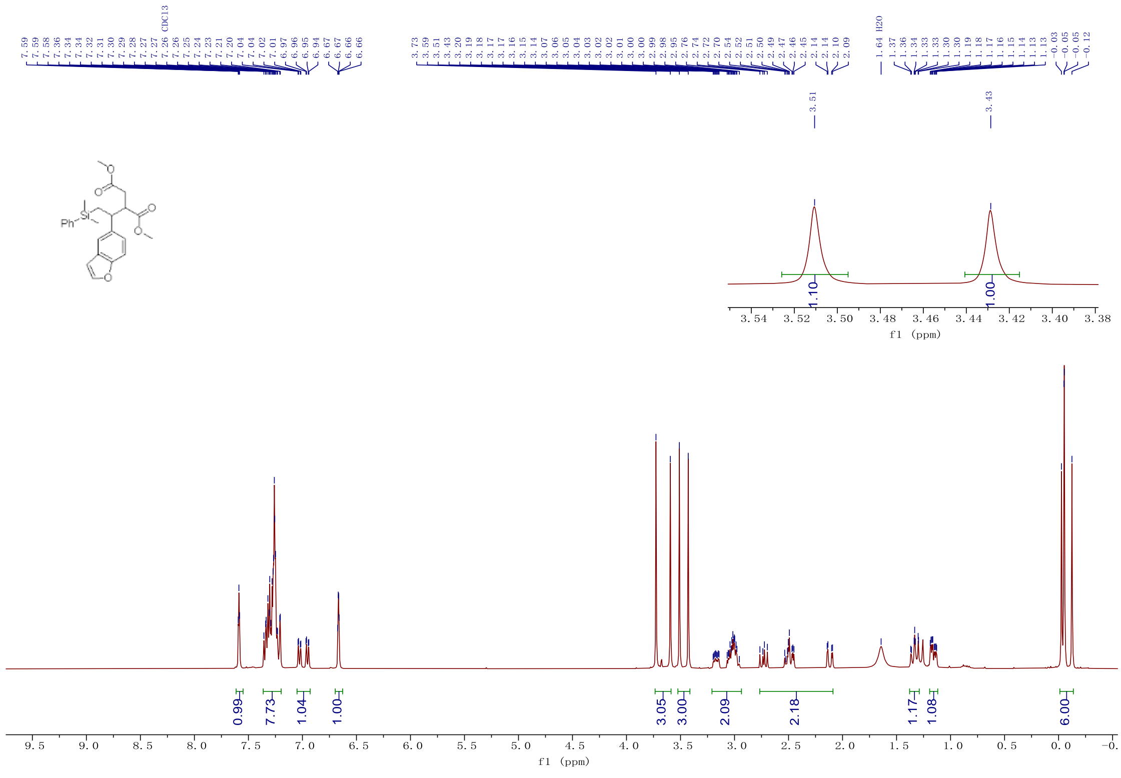

^{13}C NMR spectra of compound **d-24** in $CDCl_3$ (101 MHz): (*see procedure*)

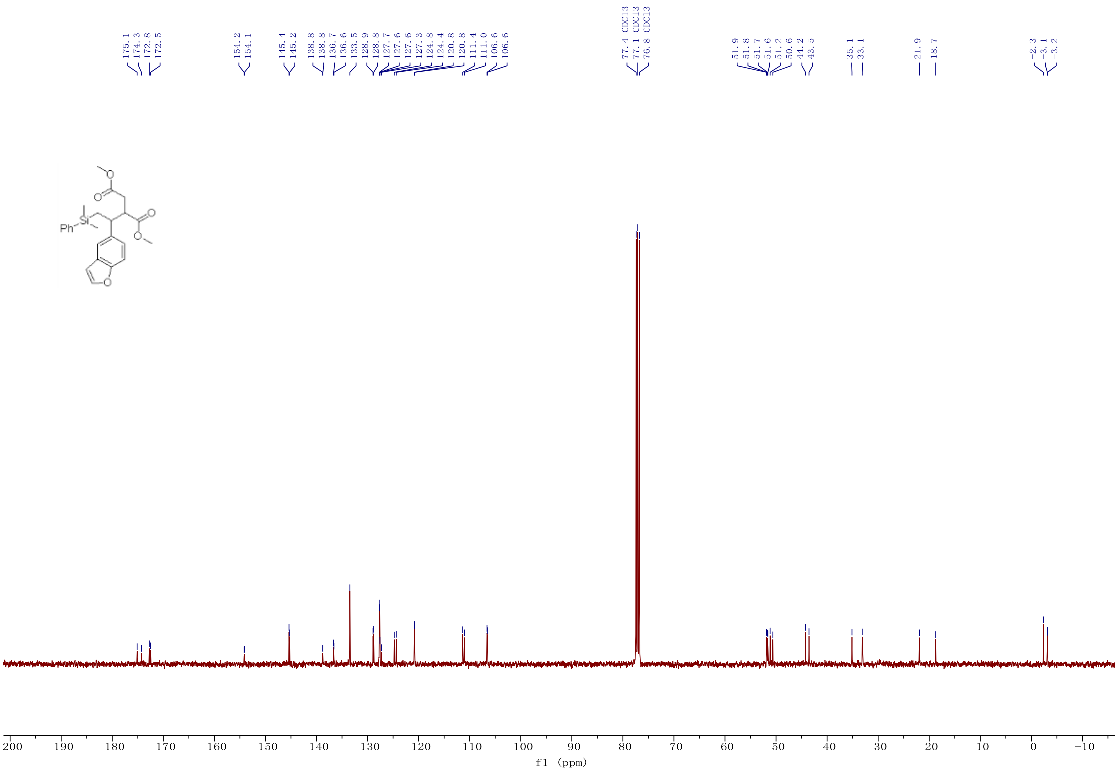

^{1}H NMR spectra of compound **d-25** in $CDCl_3$ (400 MHz): (*see procedure*)

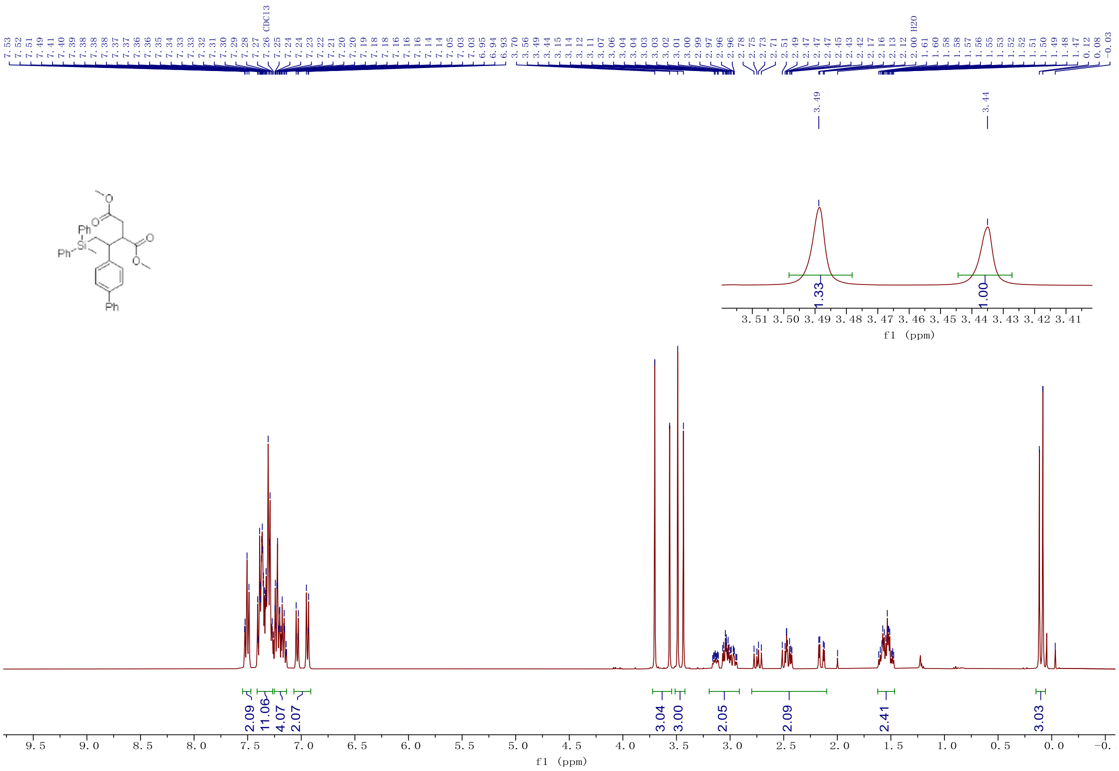

^{13}C NMR spectra of compound **d-25** in $CDCl_3$ (101 MHz): (*see procedure*)

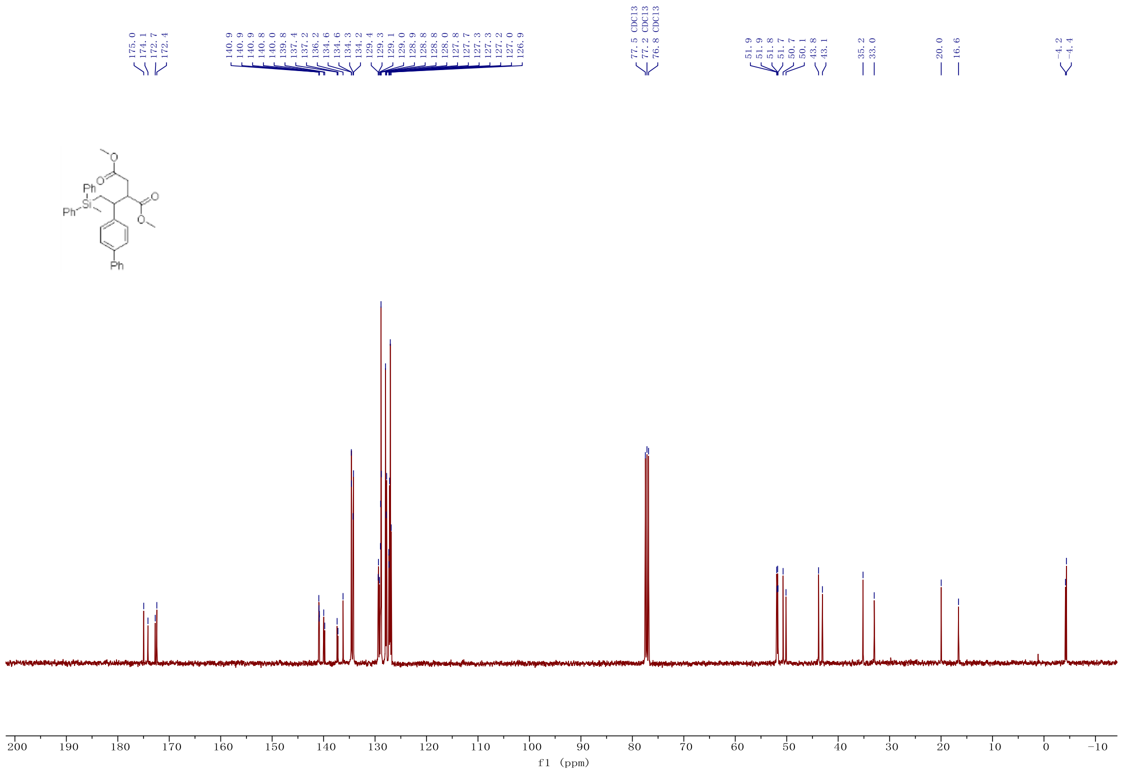

^{1}H NMR spectra of compound **d-26** in $CDCl_3$ (400 MHz): (*see procedure*)

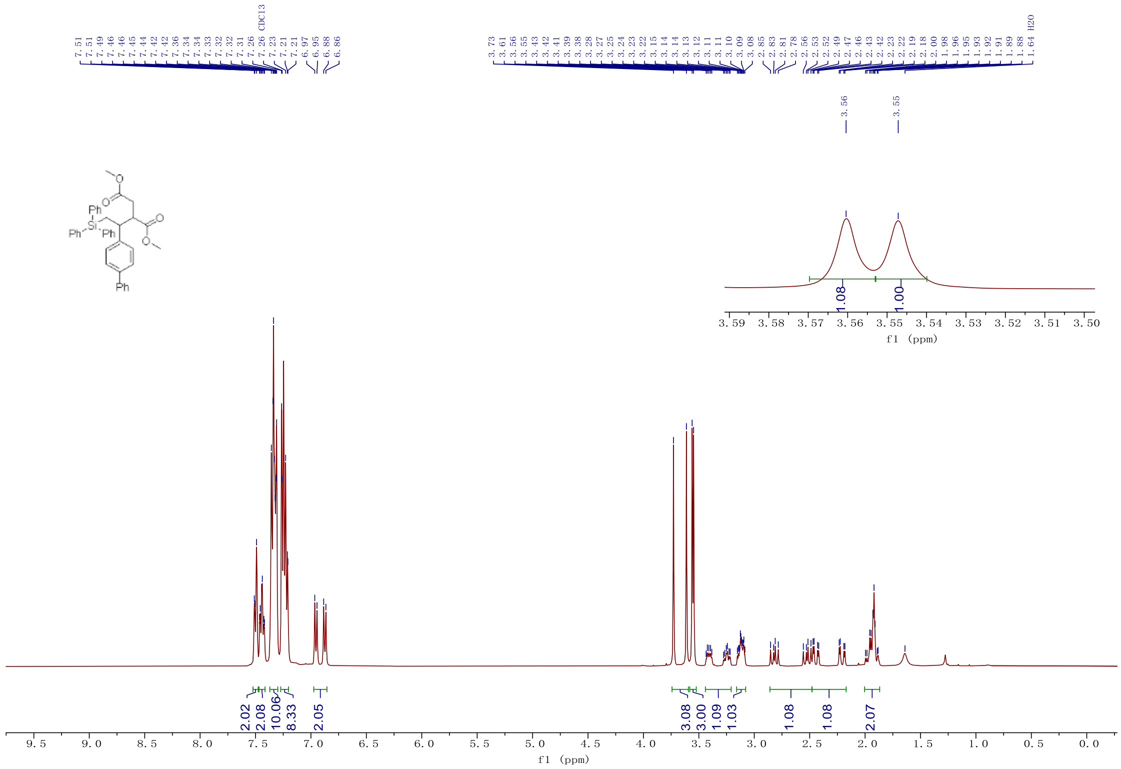

^{13}C NMR spectra of compound **d-26** in $CDCl_3$ (101 MHz): (<u>*see procedure*</u>)

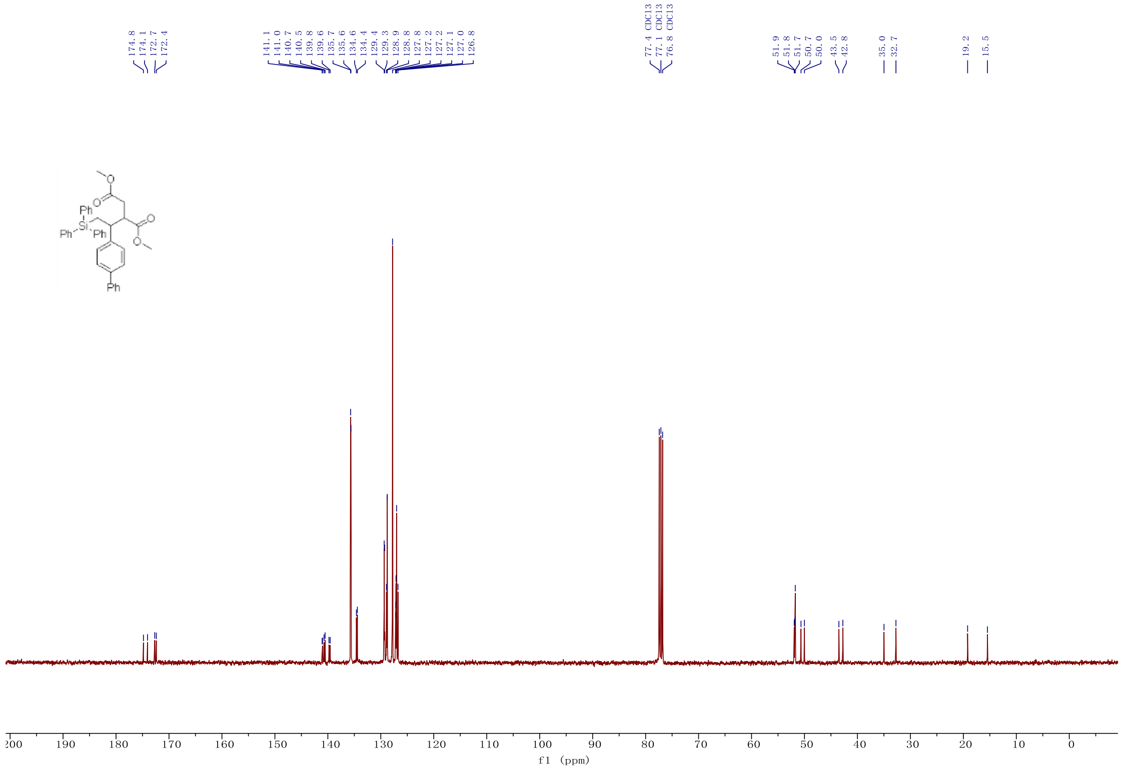

^{1}H NMR spectra of compound **d-27** in $CDCl_3$ (400 MHz): (<u>*see procedure*</u>)

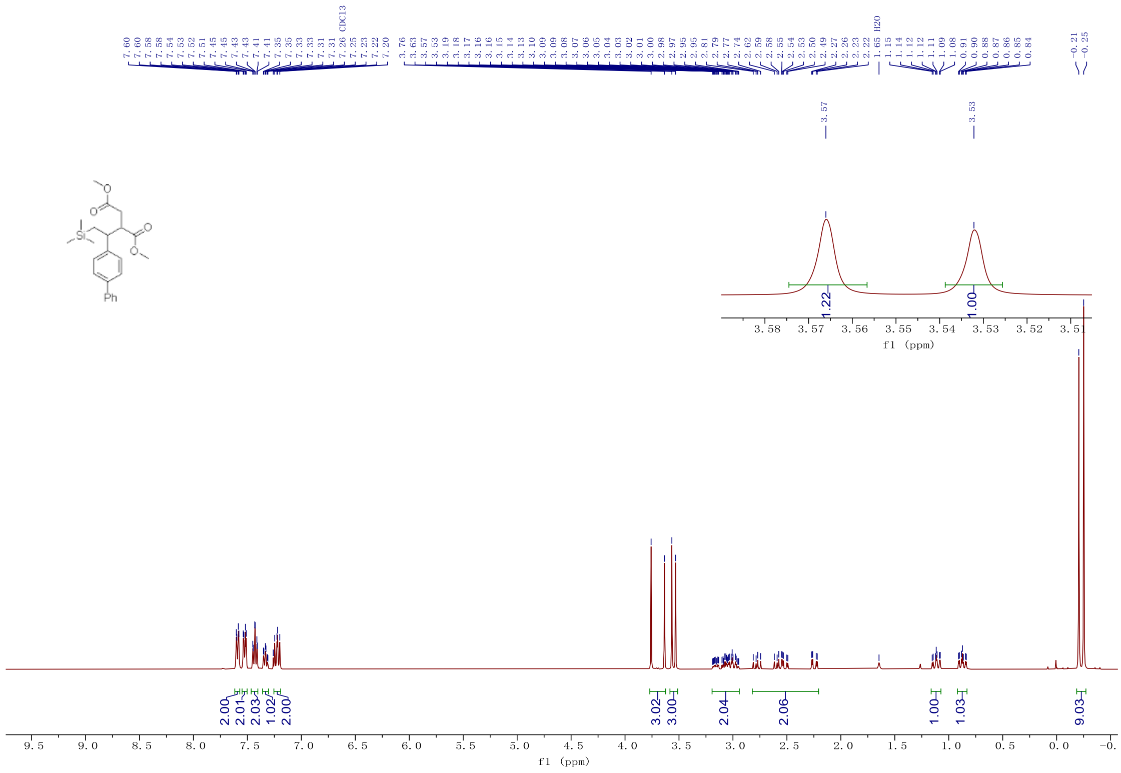

^{13}C NMR spectra of compound **d-27** in $CDCl_3$ (101 MHz): (see procedure)

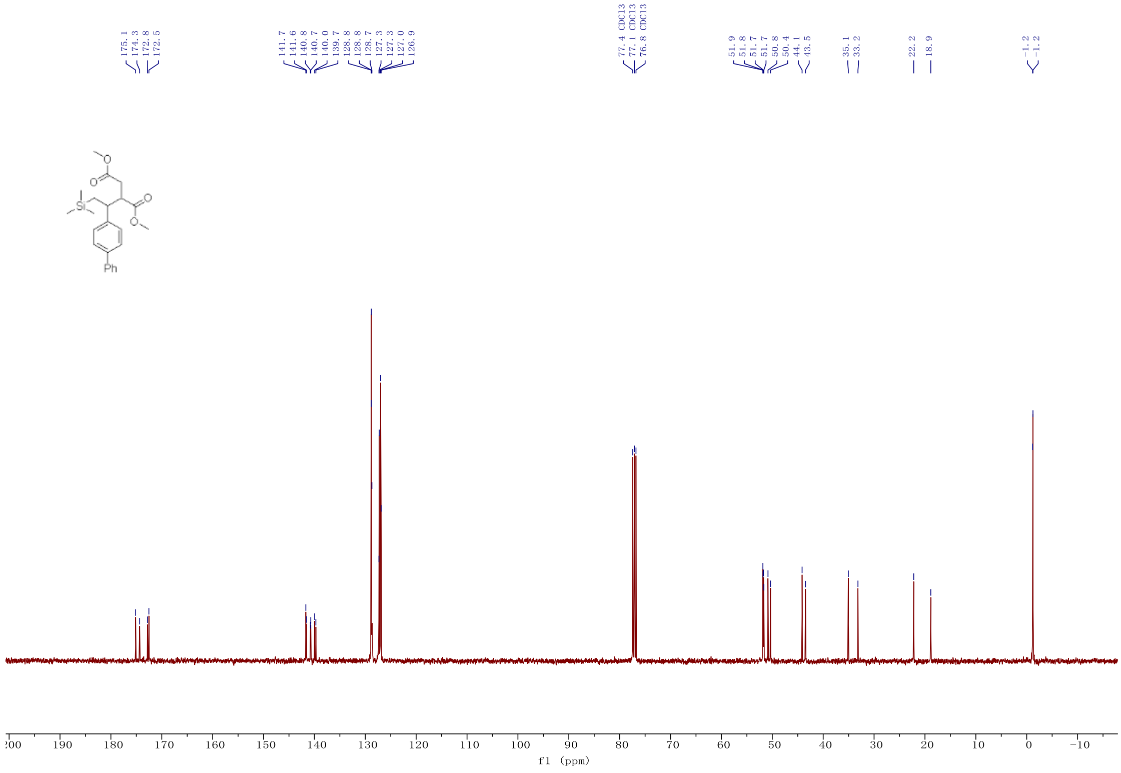

^{1}H NMR spectra of compound **d-28** in $CDCl_3$ (400 MHz): (see procedure)

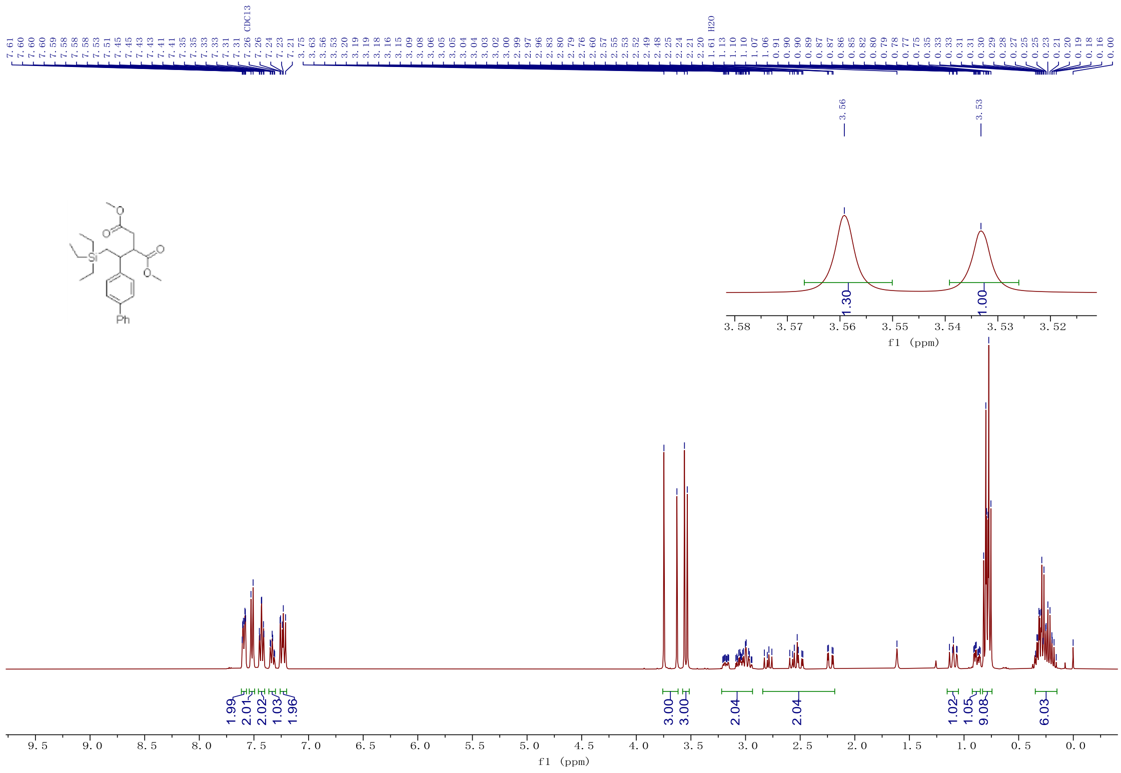

^{13}C NMR spectra of compound **d-28** in $CDCl_3$ (101 MHz): (*see procedure*)

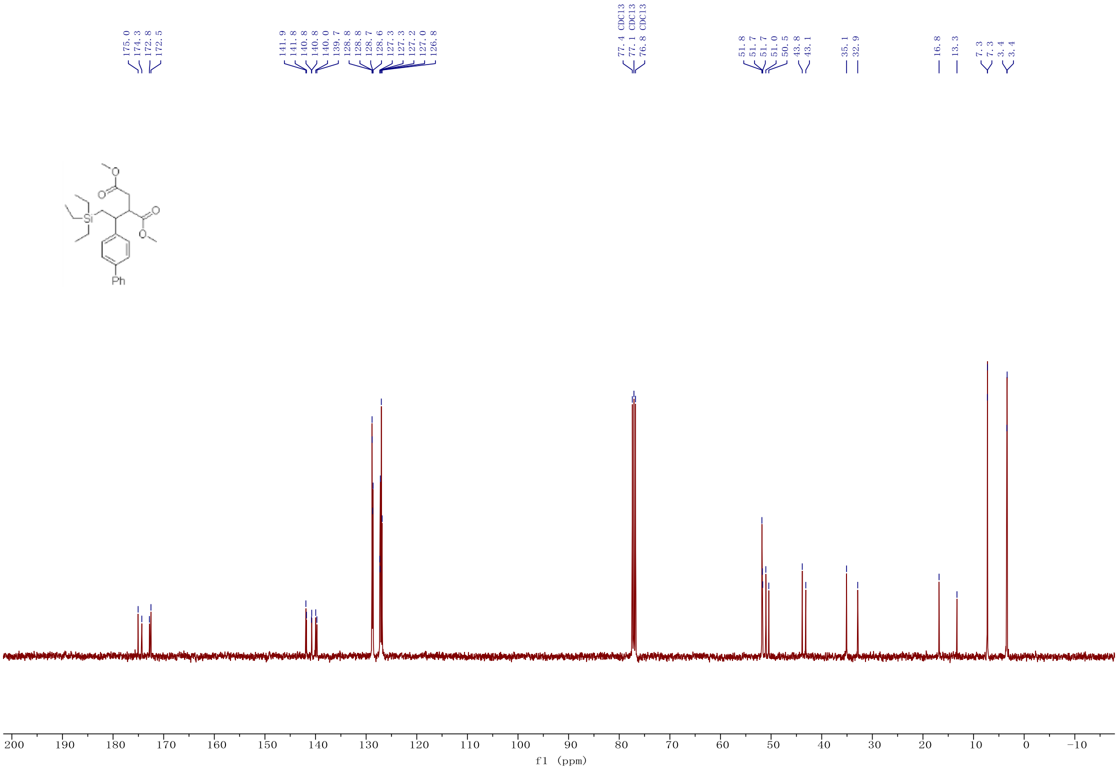

^{1}H NMR spectra of compound **d-29** in $CDCl_3$ (400 MHz): (*see procedure*)

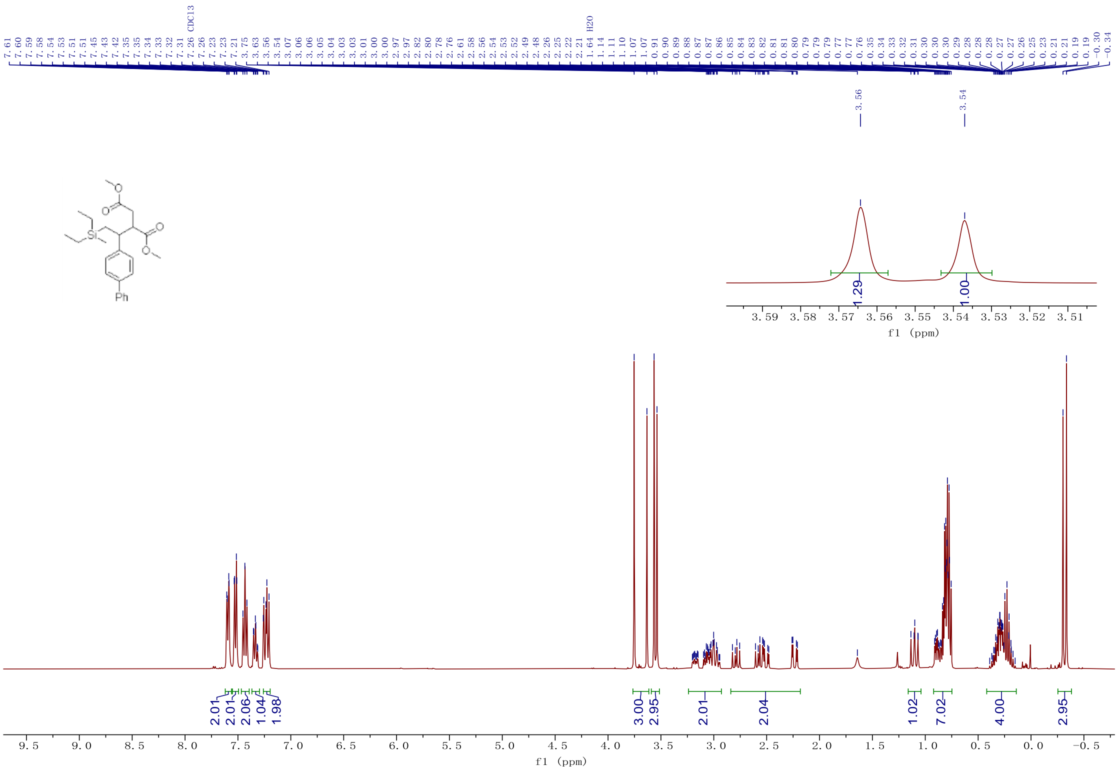

^{13}C NMR spectra of compound **d-29** in $CDCl_3$ (101 MHz): (see procedure)

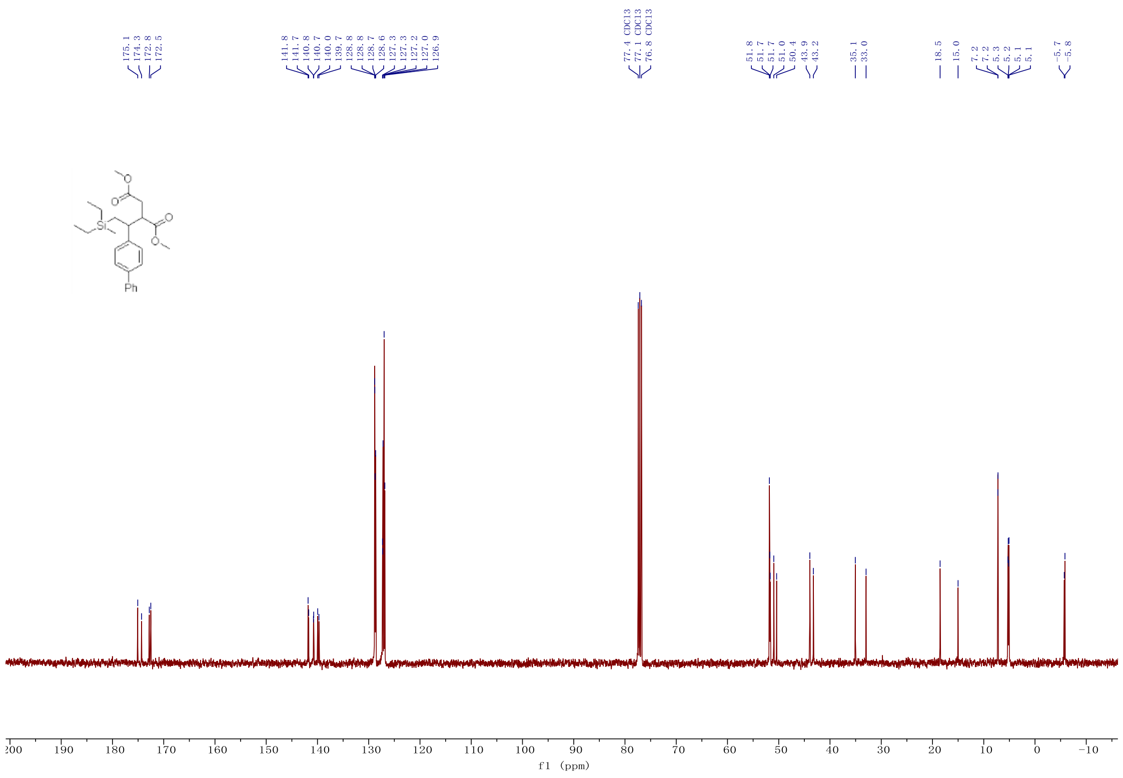

^{1}H NMR spectra of compound **d-30** in $CDCl_3$ (400 MHz): (see procedure)

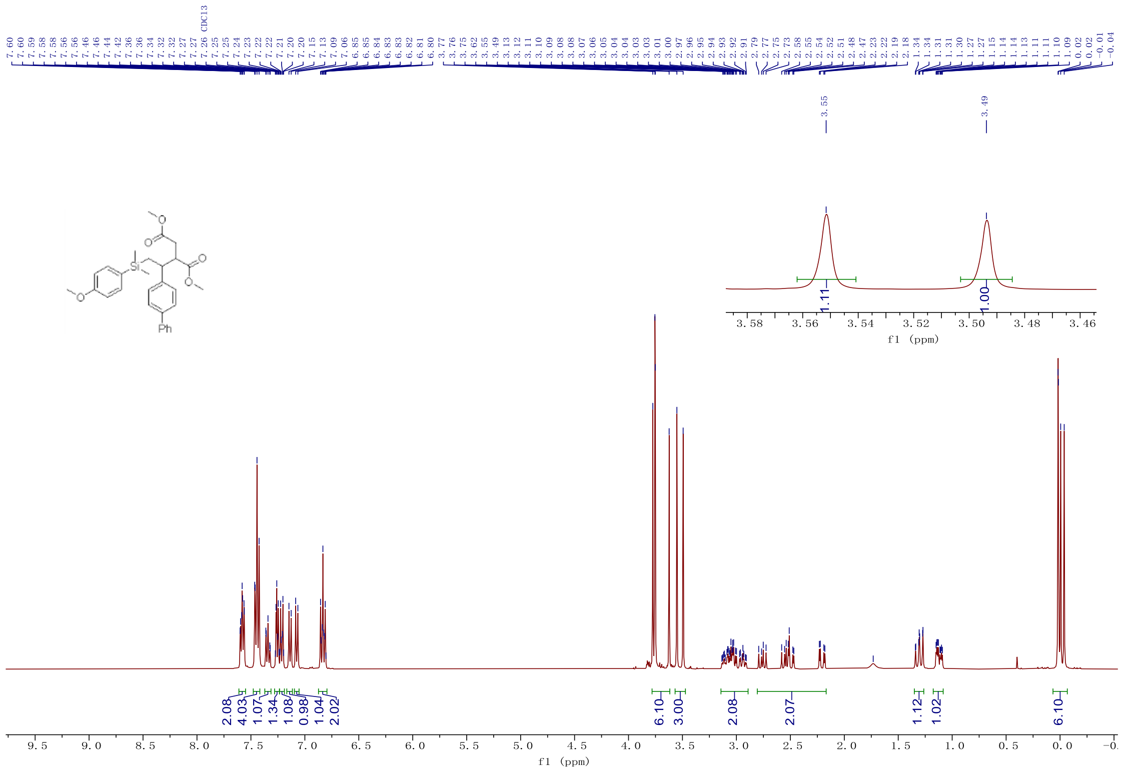

^{13}C NMR spectra of compound **d-30** in $CDCl_3$ (101 MHz): (*see procedure*)

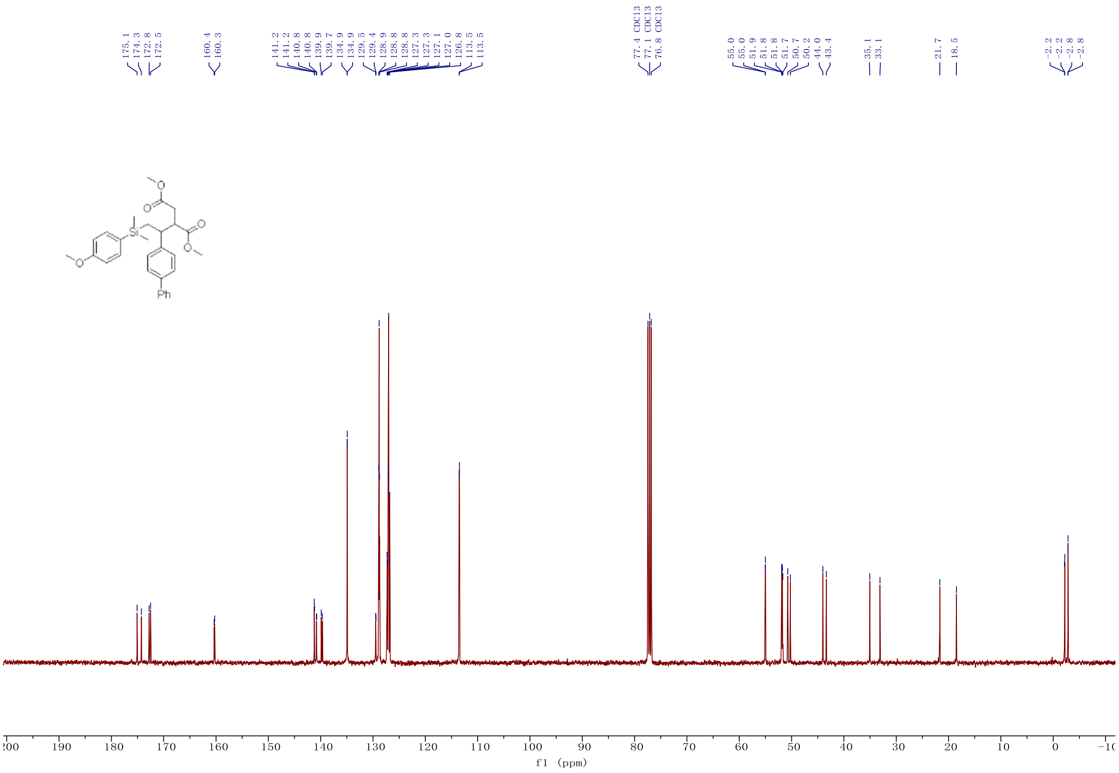

^{1}H NMR spectra of compound **d-31** in $CDCl_3$ (400 MHz): (*see procedure*)

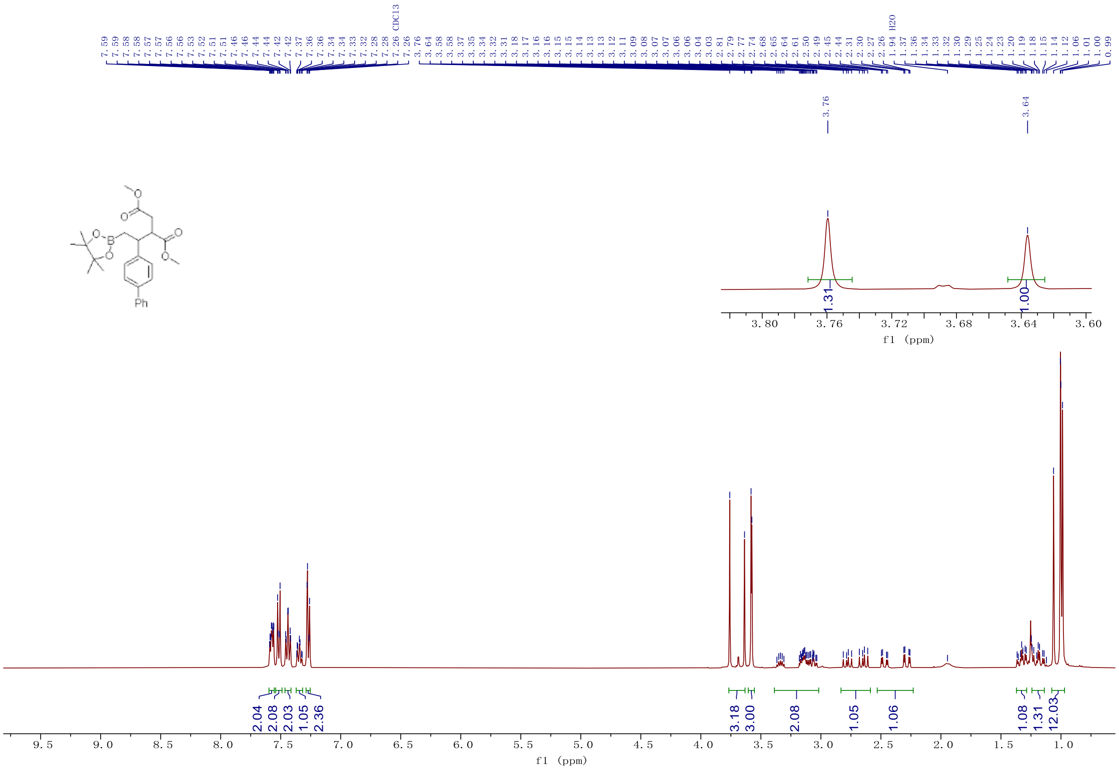

^{13}C NMR spectra of compound **d-31** in $CDCl_3$ (101 MHz): (*see procedure*)

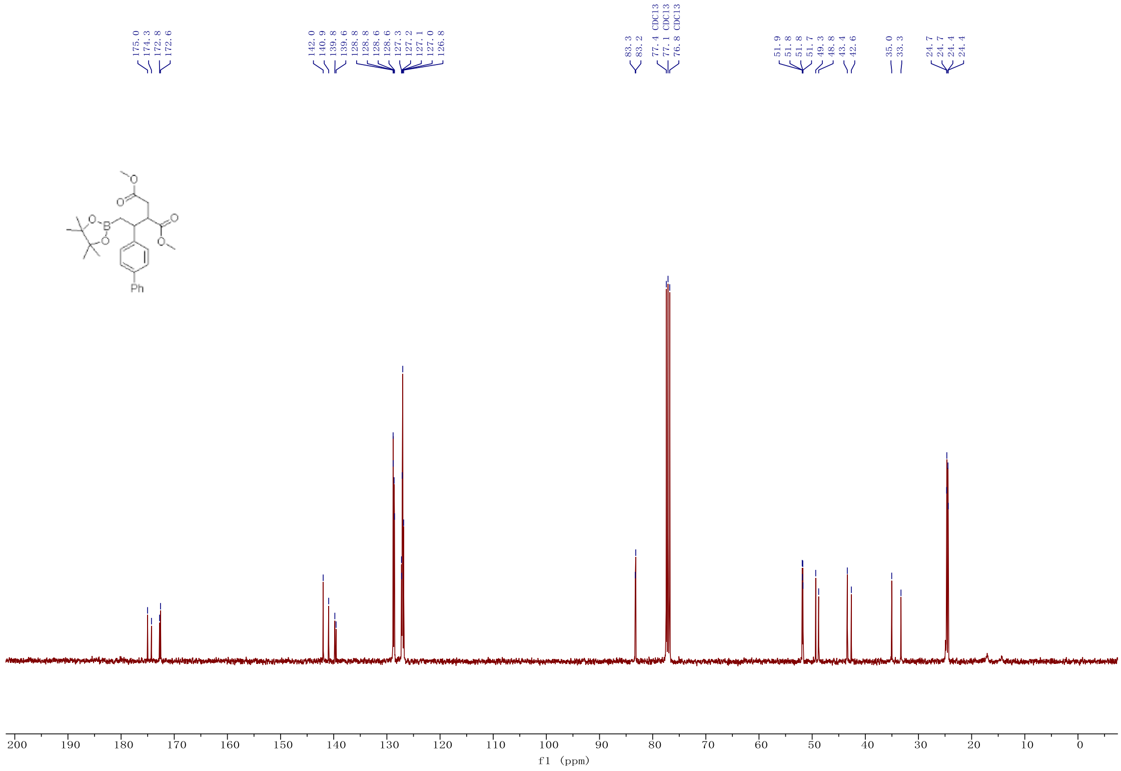

^{1}H NMR spectra of compound **d-32** in $CDCl_3$ (400 MHz): (*see procedure*)

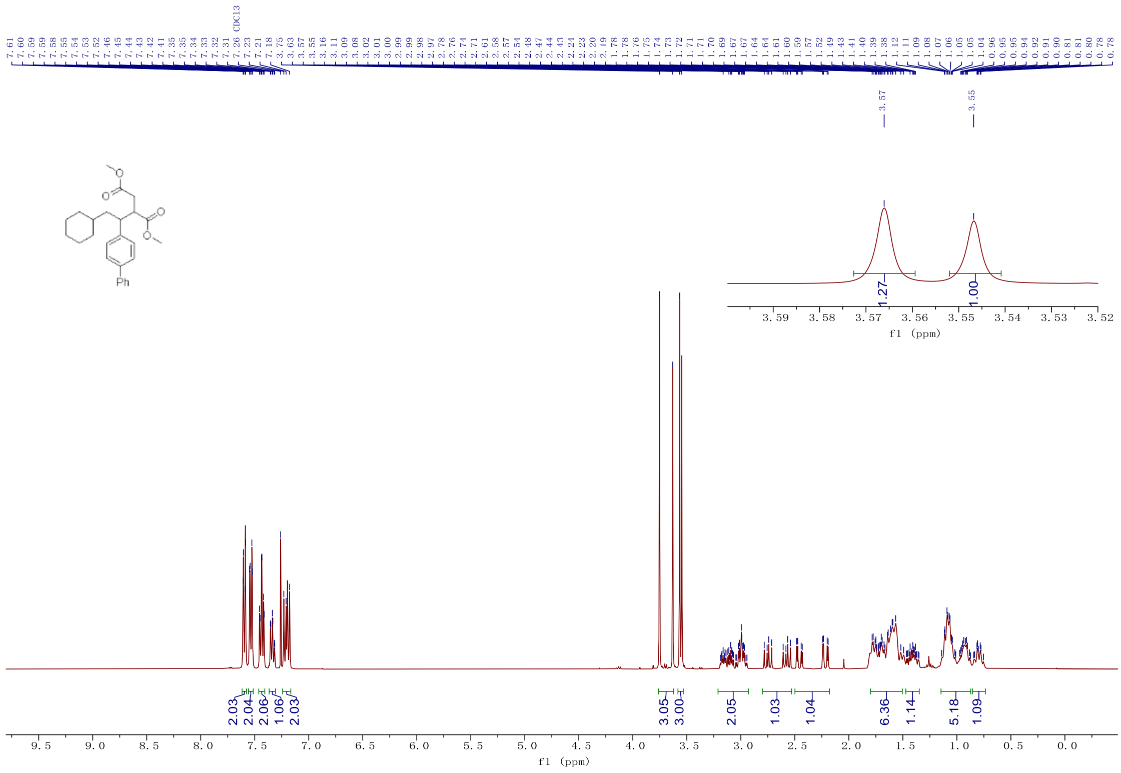

^{13}C NMR spectra of compound **d-32** in $CDCl_3$ (101 MHz): (*see procedure*)

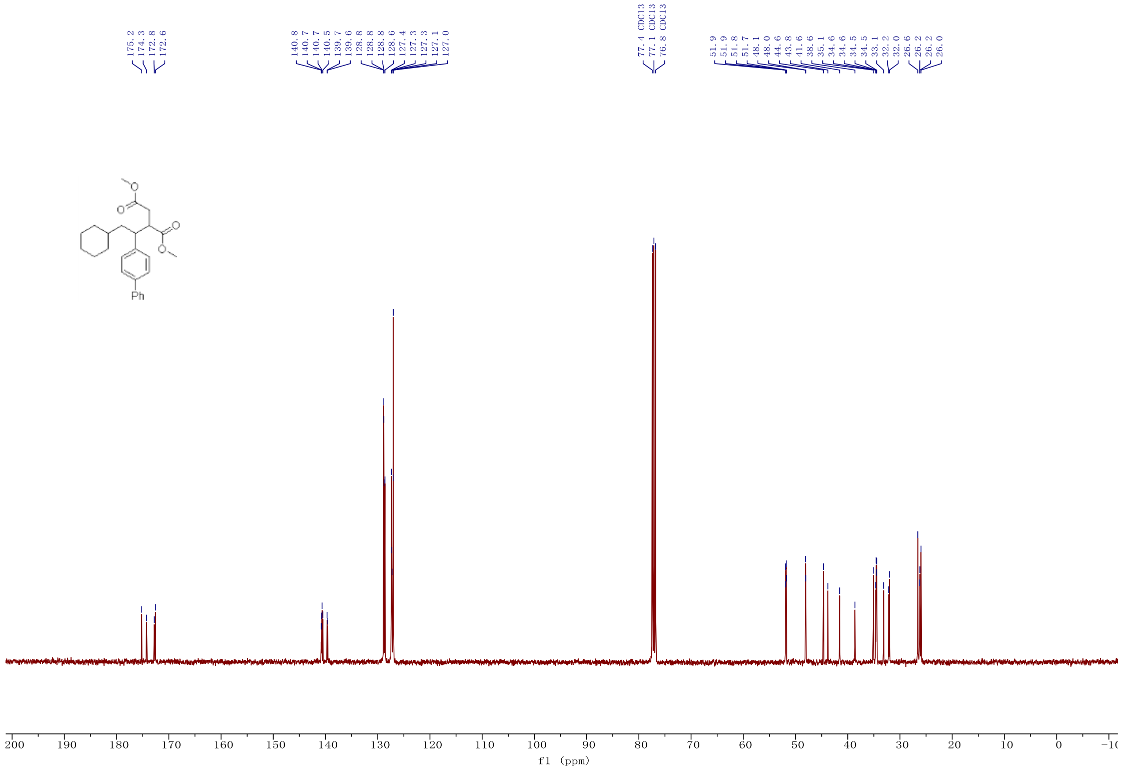

^{1}H NMR spectra of compound **d-33** in $CDCl_3$ (400 MHz): (*see procedure*)

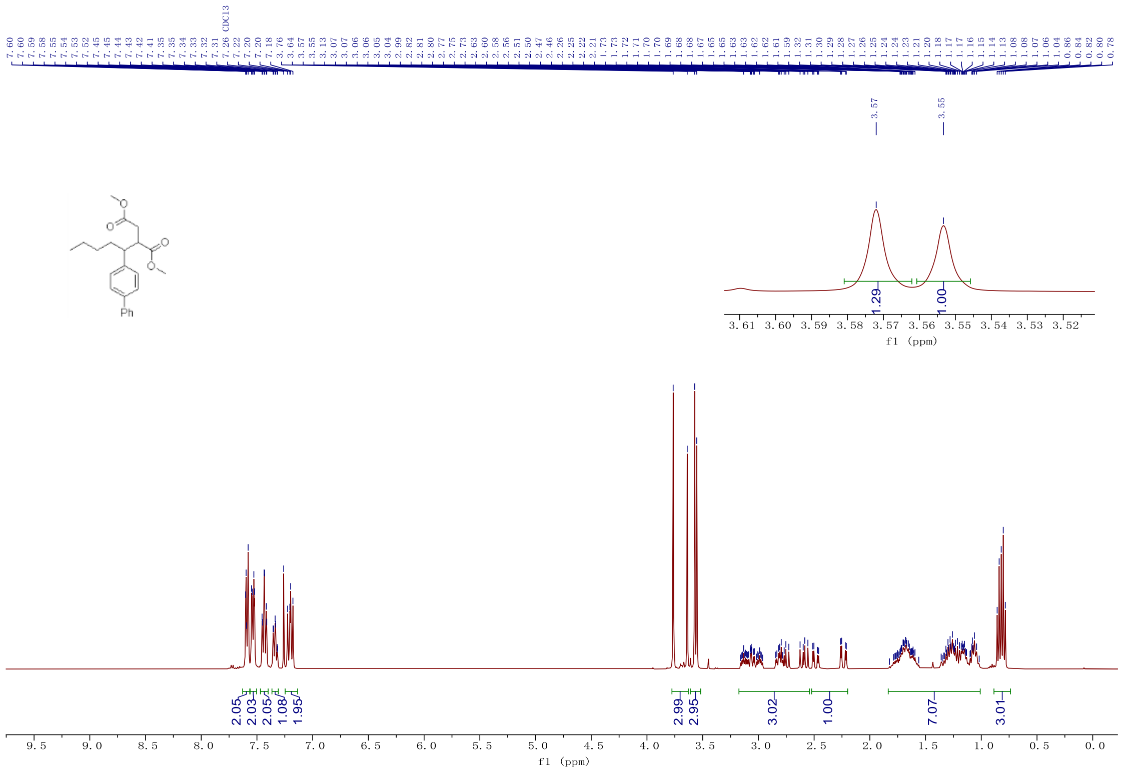

^{13}C NMR spectra of compound **d-33** in $CDCl_3$ (101 MHz): (*see procedure*)

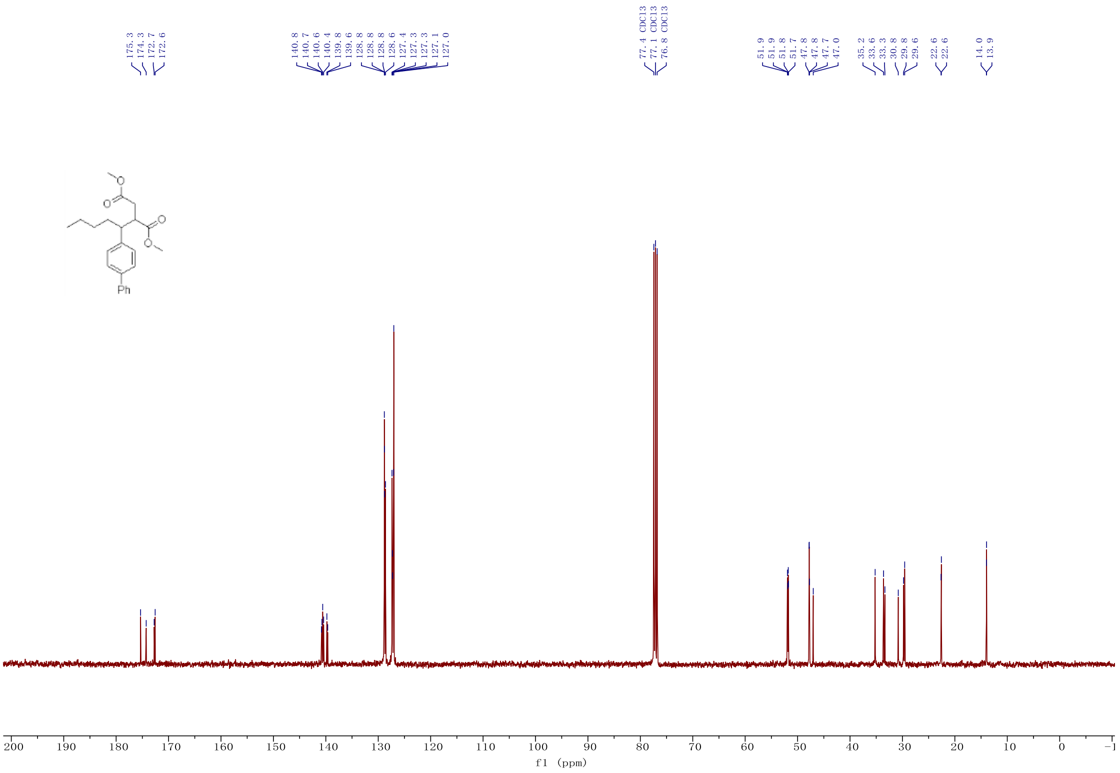

^{1}H NMR spectra of compound **d-34** in $CDCl_3$ (400 MHz): (*see procedure*)

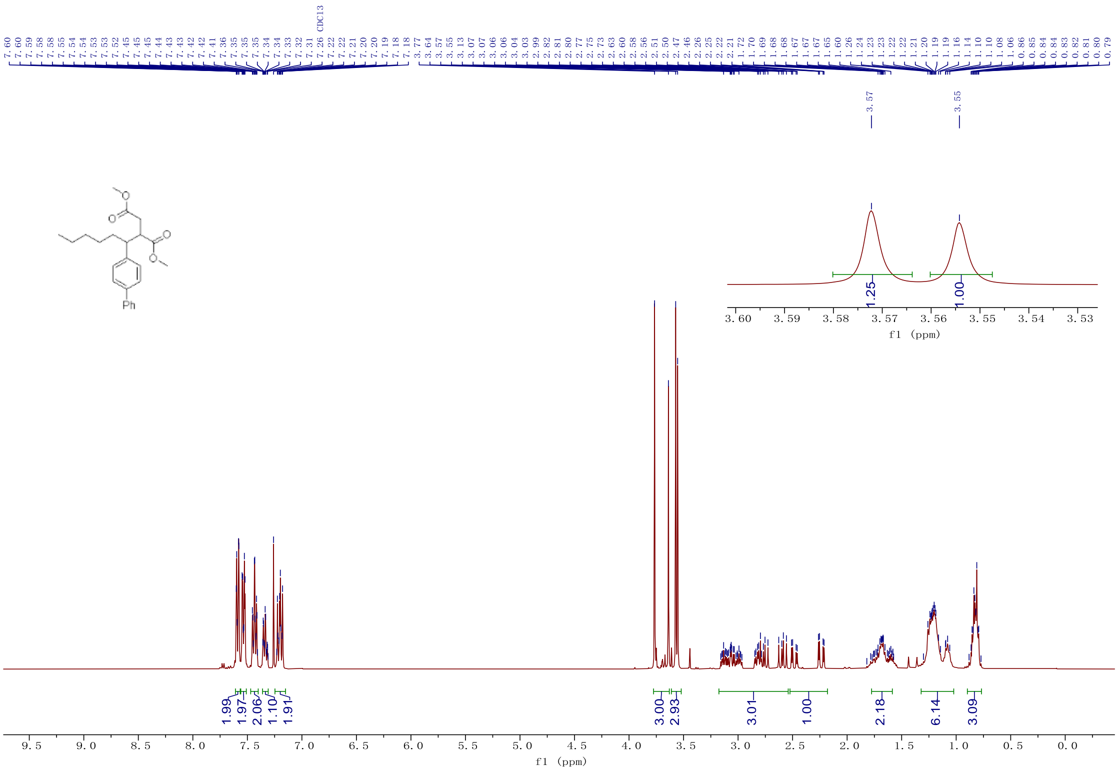

^{13}C NMR spectra of compound **d-34** in $CDCl_3$ (101 MHz): (*see procedure*)

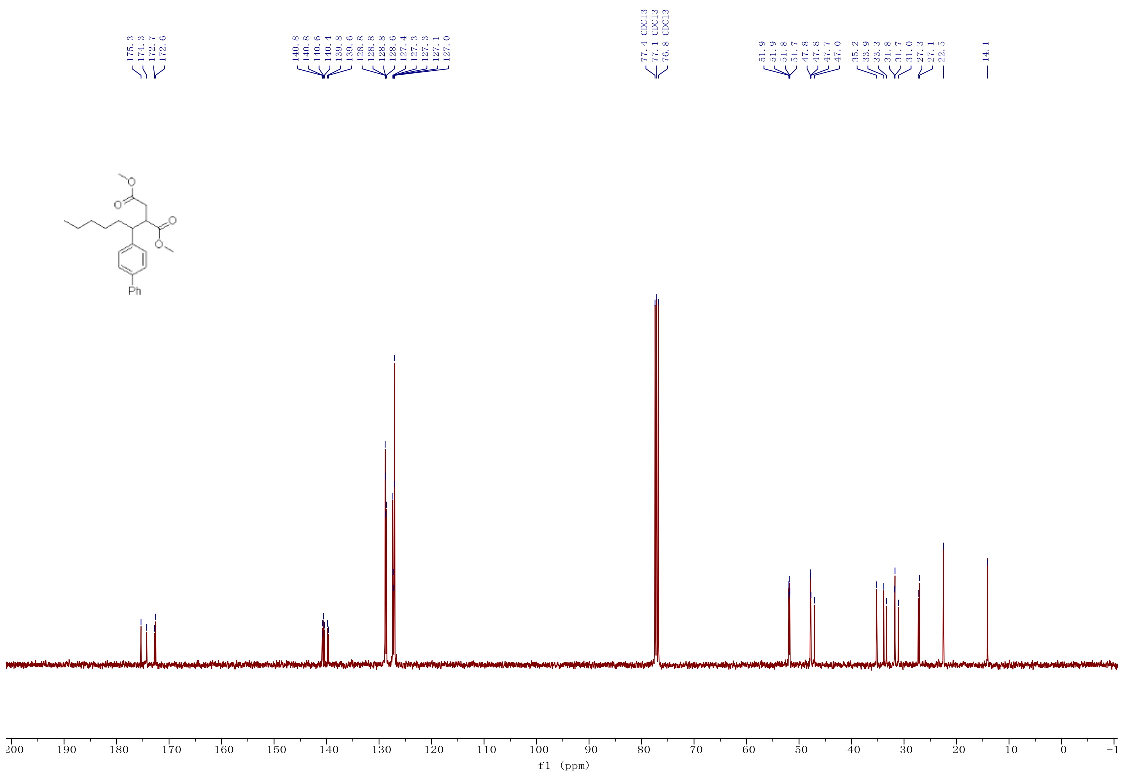

^{1}H NMR spectra of compound **d-35** in $CDCl_3$ (400 MHz): (*see procedure*)

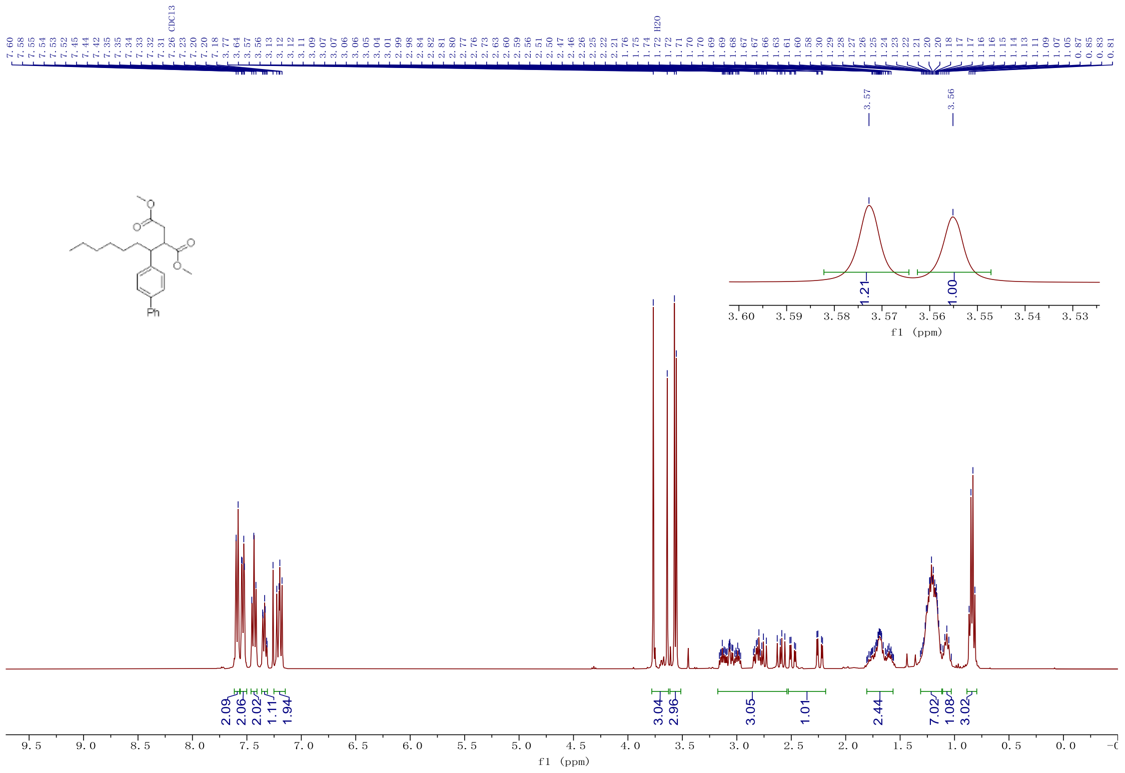

^{13}C NMR spectra of compound **d-35** in $CDCl_3$ (101 MHz): (<u>*see procedure*</u>)

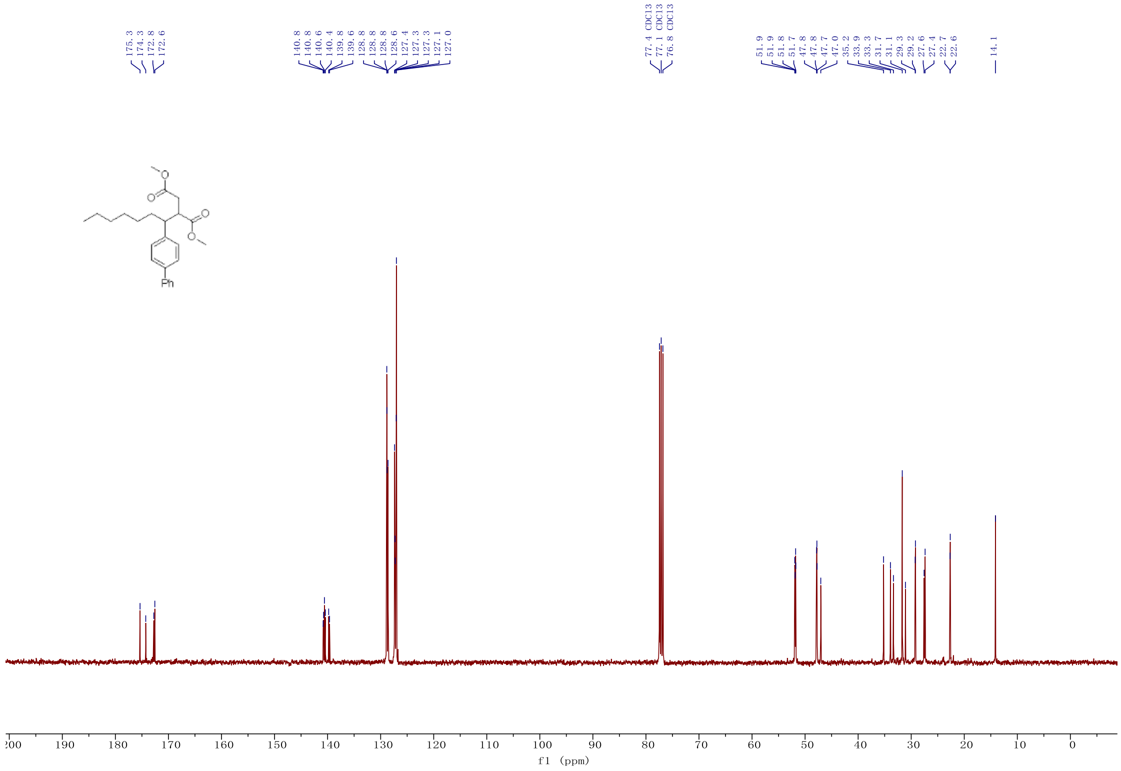

^{1}H NMR spectra of compound **d-36** in $CDCl_3$ (400 MHz): (<u>*see procedure*</u>)

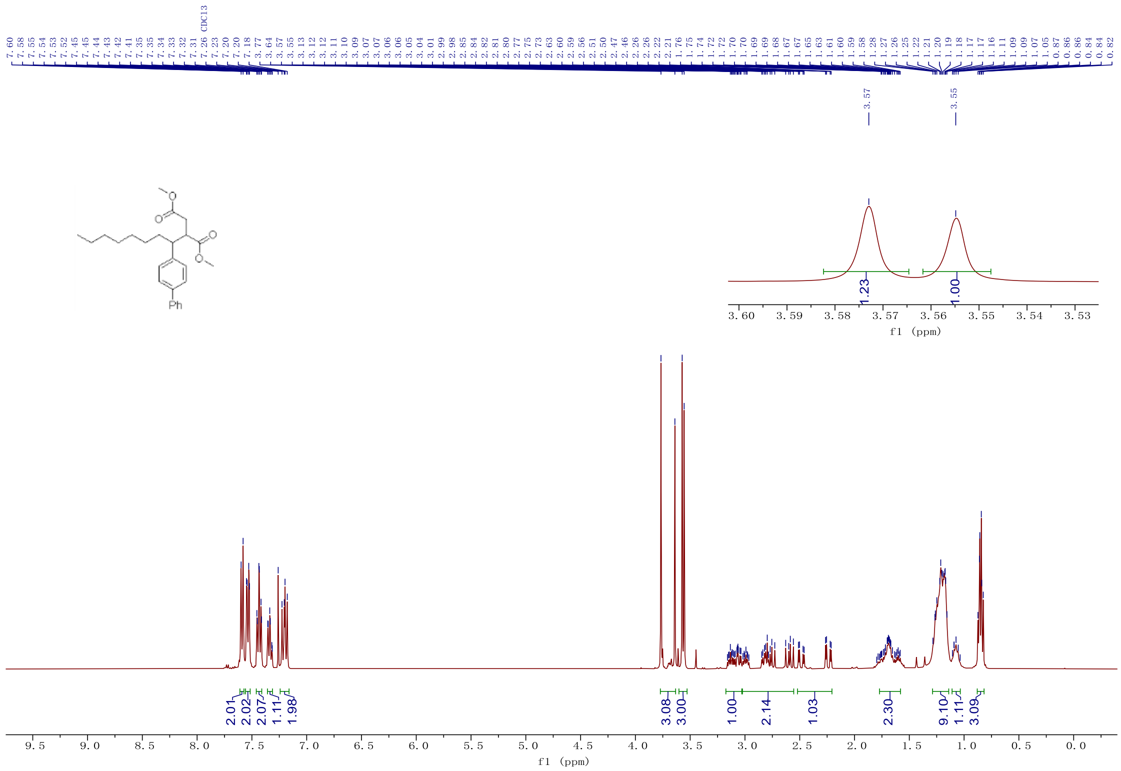

^{13}C NMR spectra of compound **d-36** in $CDCl_3$ (101 MHz): (*see procedure*)

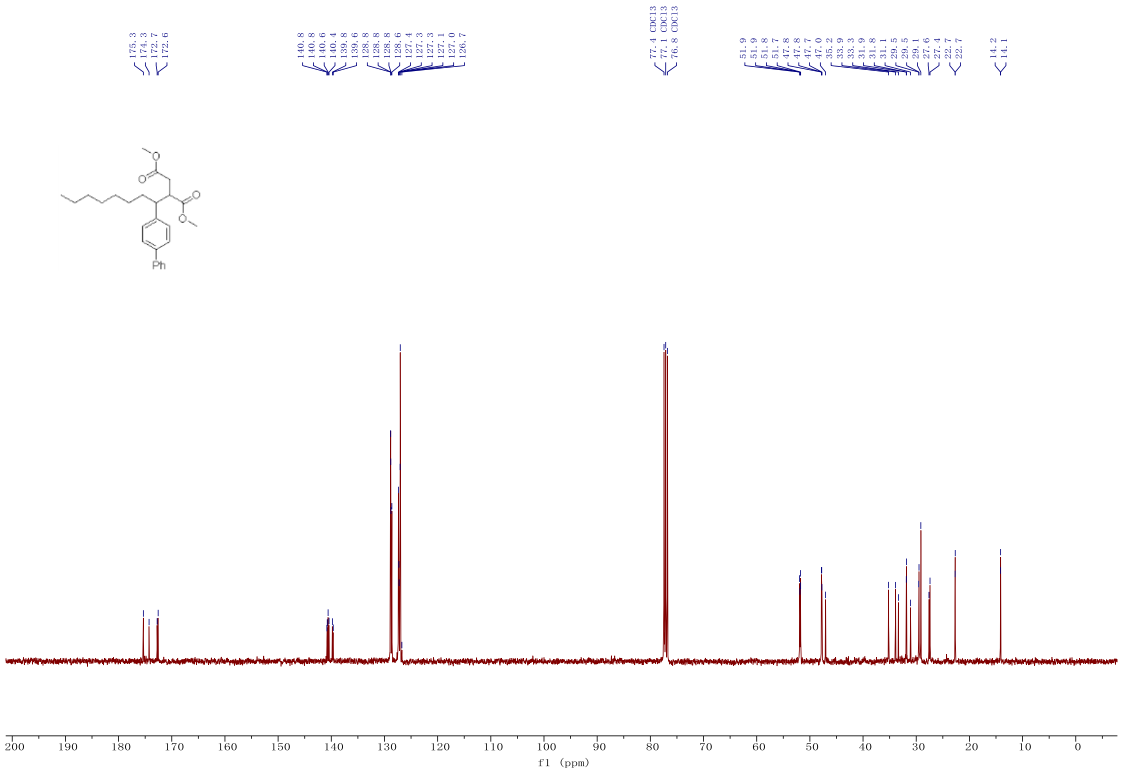

^{1}H NMR spectra of compound **d-37** in $CDCl_3$ (400 MHz): (*see procedure*)

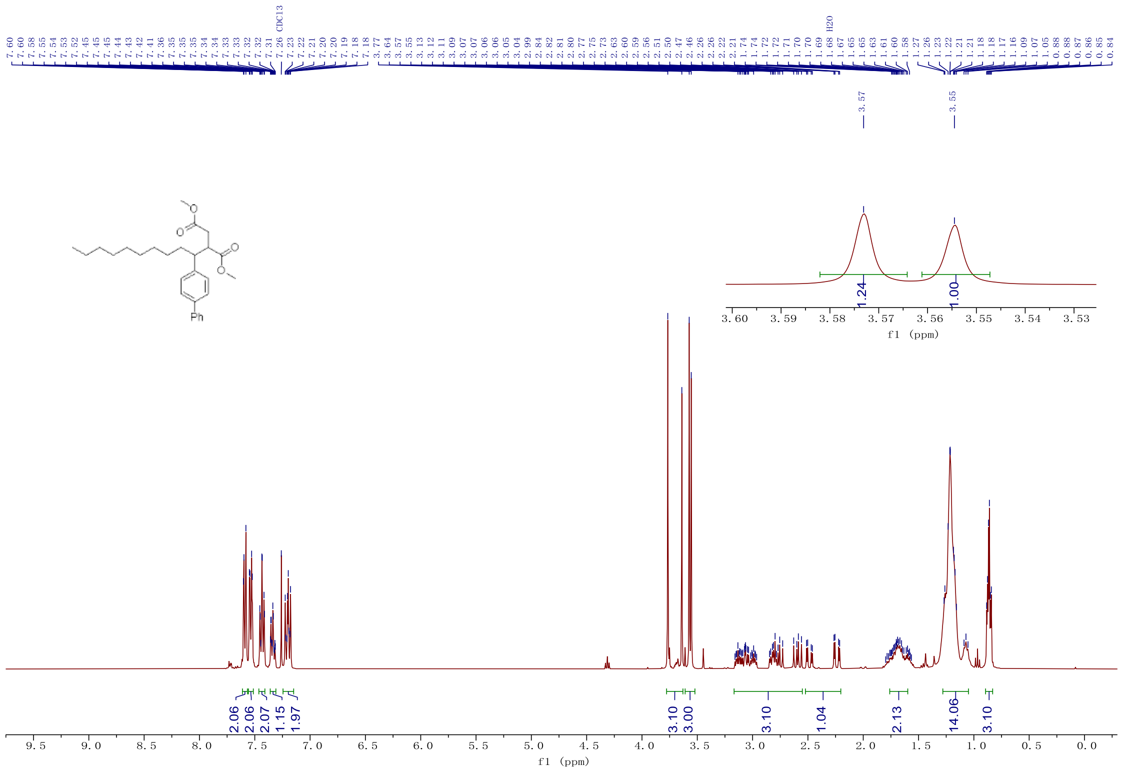

^{13}C NMR spectra of compound **d-37** in $CDCl_3$ (101 MHz): (*see procedure*)

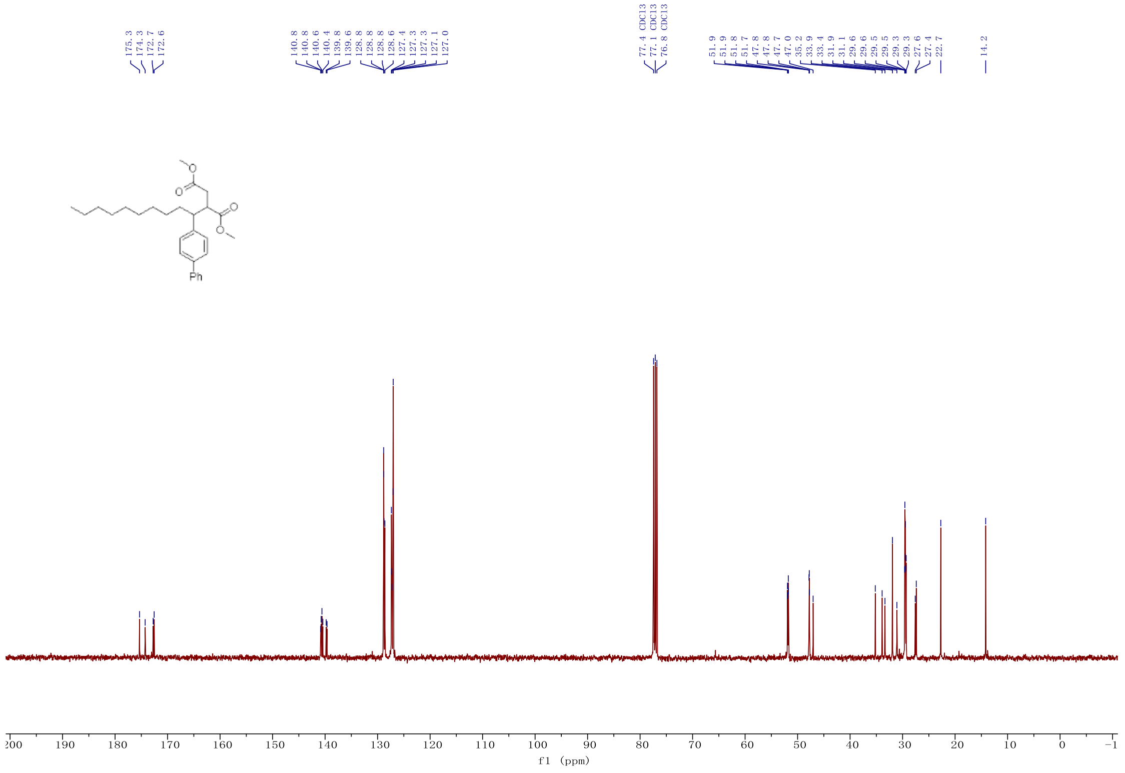

^{1}H NMR spectra of compound **d-38** in $CDCl_3$ (400 MHz): (*see procedure*)

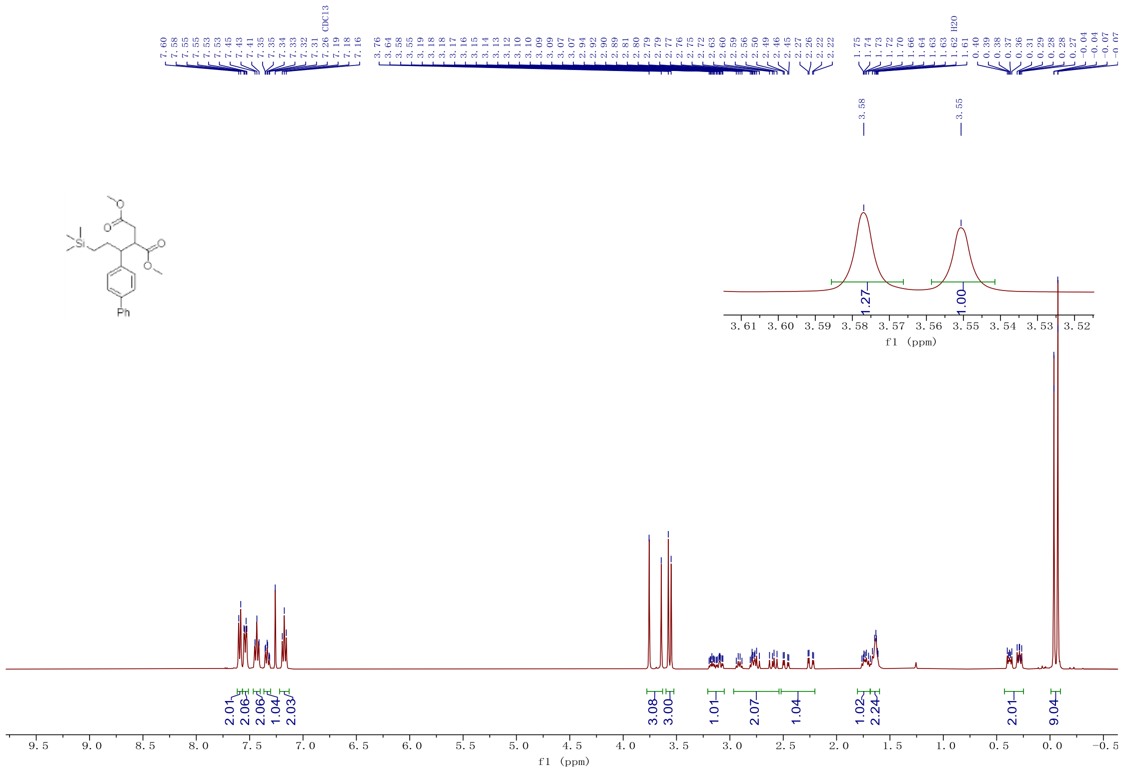

^{13}C NMR spectra of compound **d-38** in $CDCl_3$ (101 MHz): (*see procedure*)

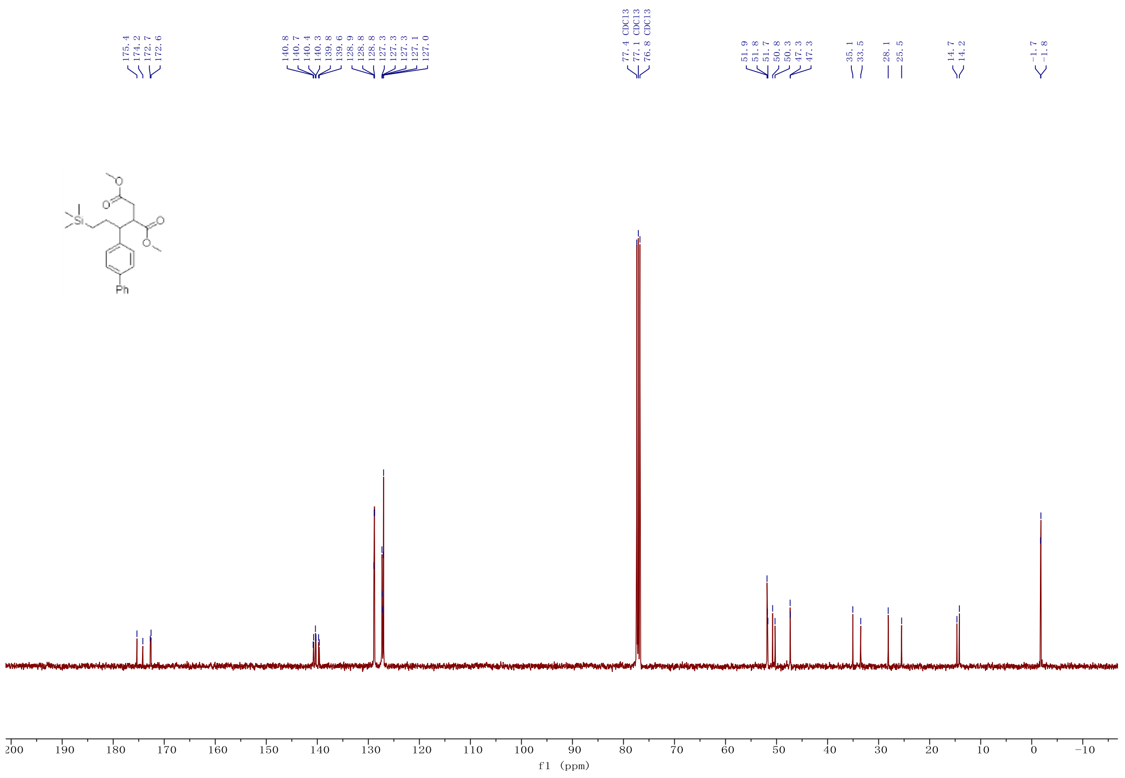

^{1}H NMR spectra of compound **d-39** in $CDCl_3$ (400 MHz): (*see procedure*)

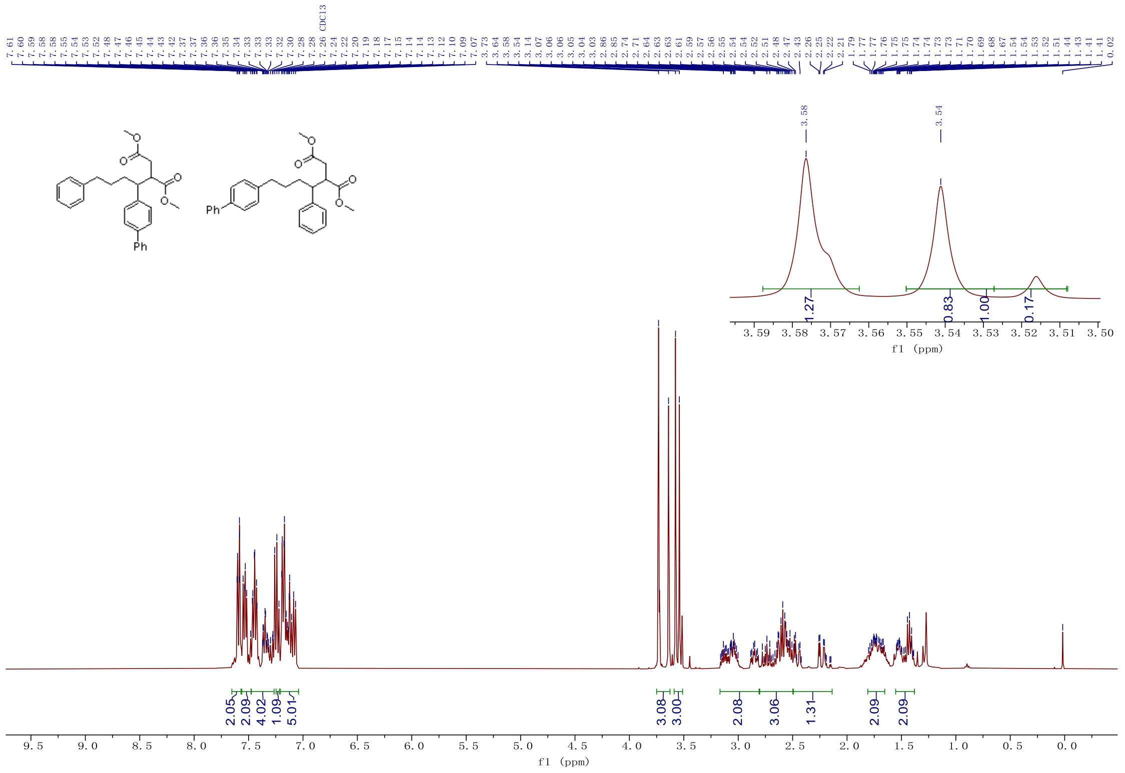

^{13}C NMR spectra of compound **d-39** in $CDCl_3$ (101 MHz): (*see procedure*)

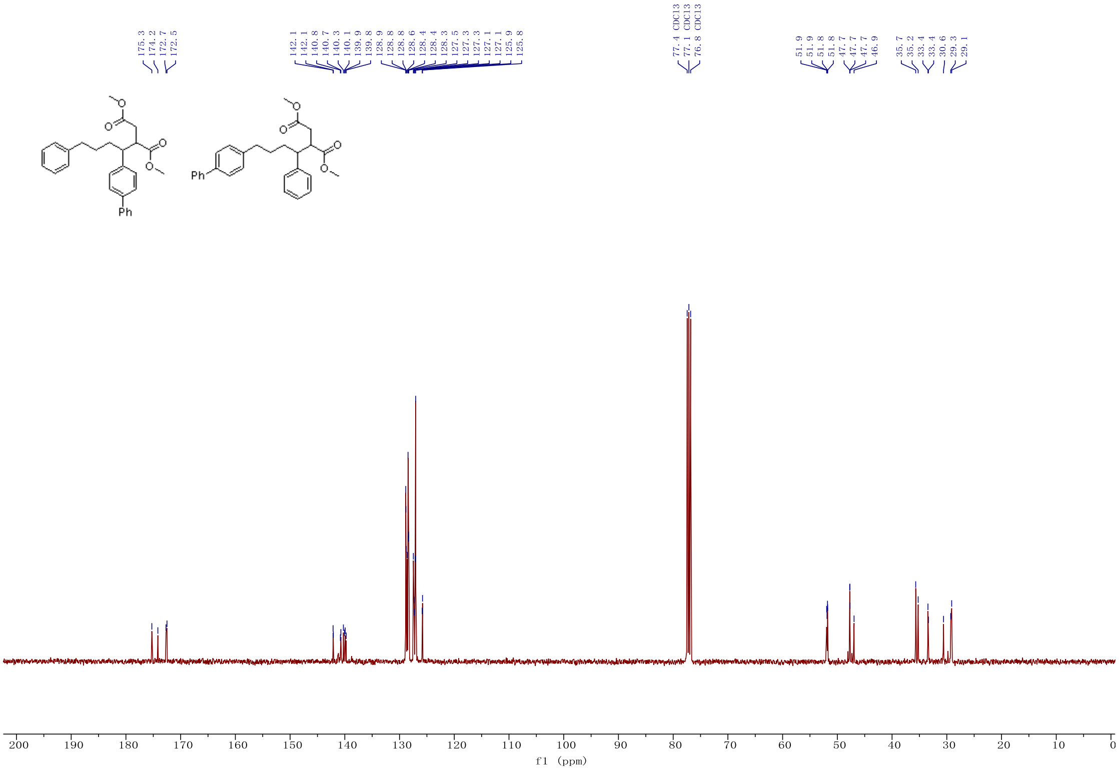

^{1}H NMR spectra of compound **d-40** in $CDCl_3$ (400 MHz): (*see procedure*)

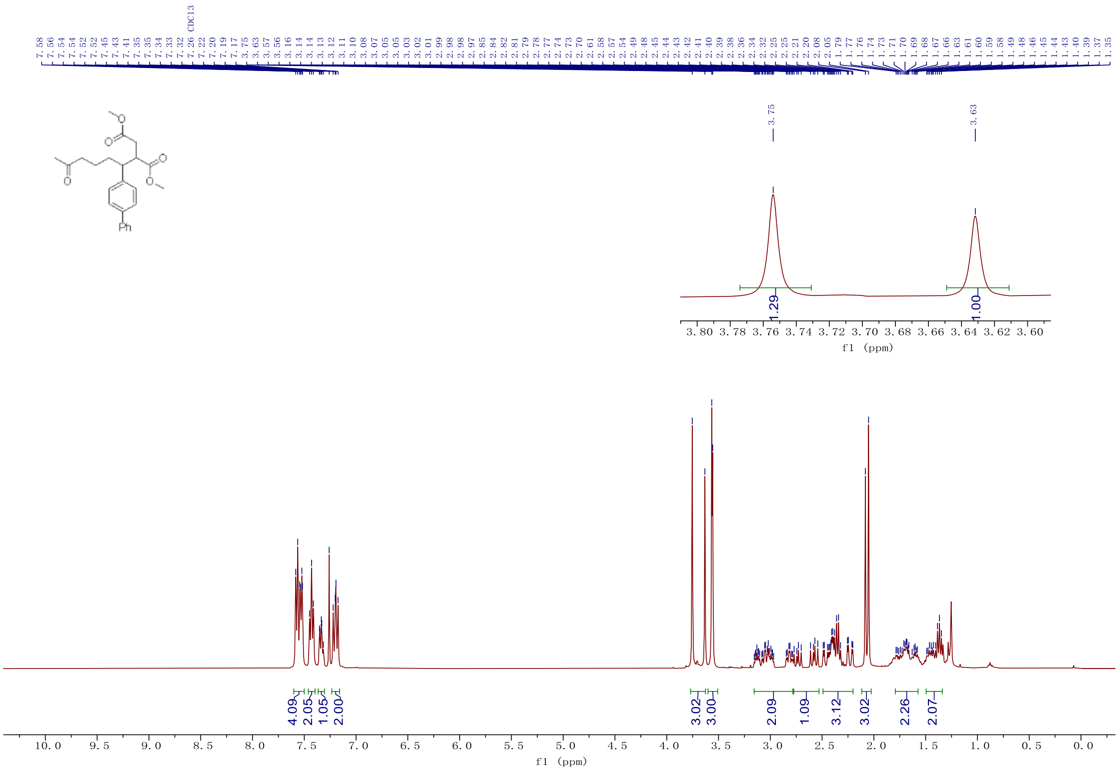

^{13}C NMR spectra of compound **d-40** in $CDCl_3$ (101 MHz): (*see procedure*)

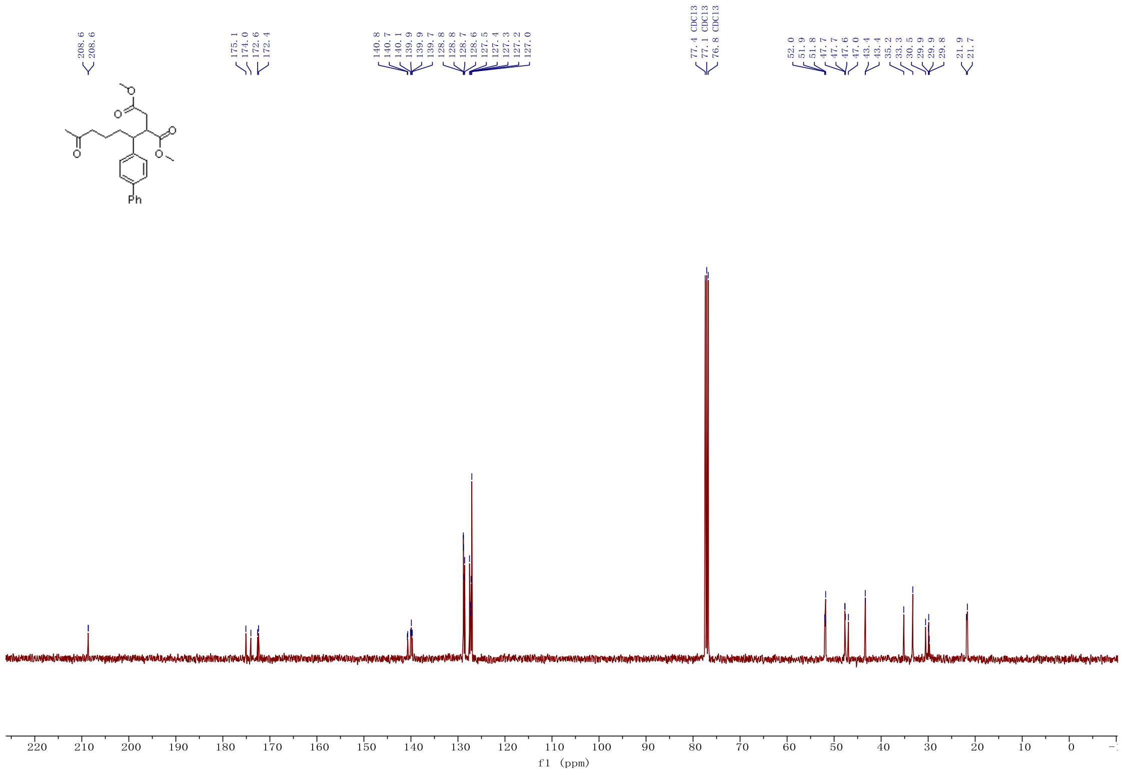

^{1}H NMR spectra of compound **d-41** in $CDCl_3$ (400 MHz): (*see procedure*)

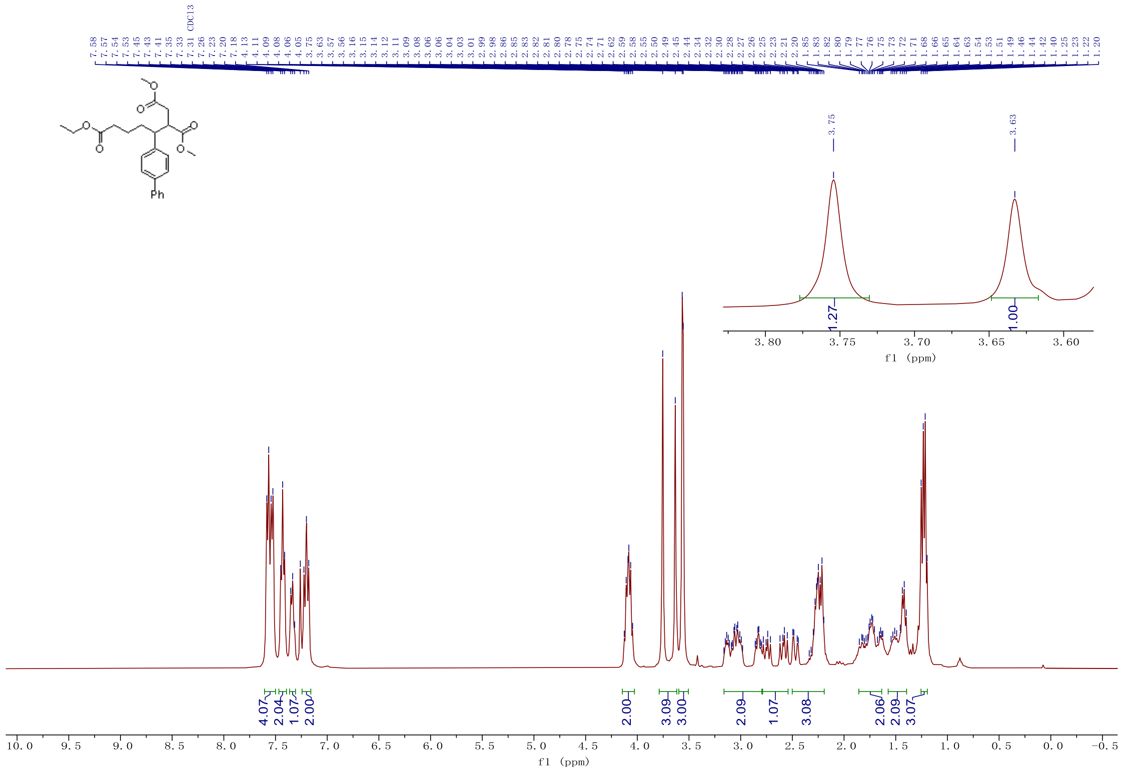

^{13}C NMR spectra of compound **d-41** in $CDCl_3$ (101 MHz): (*see procedure*)

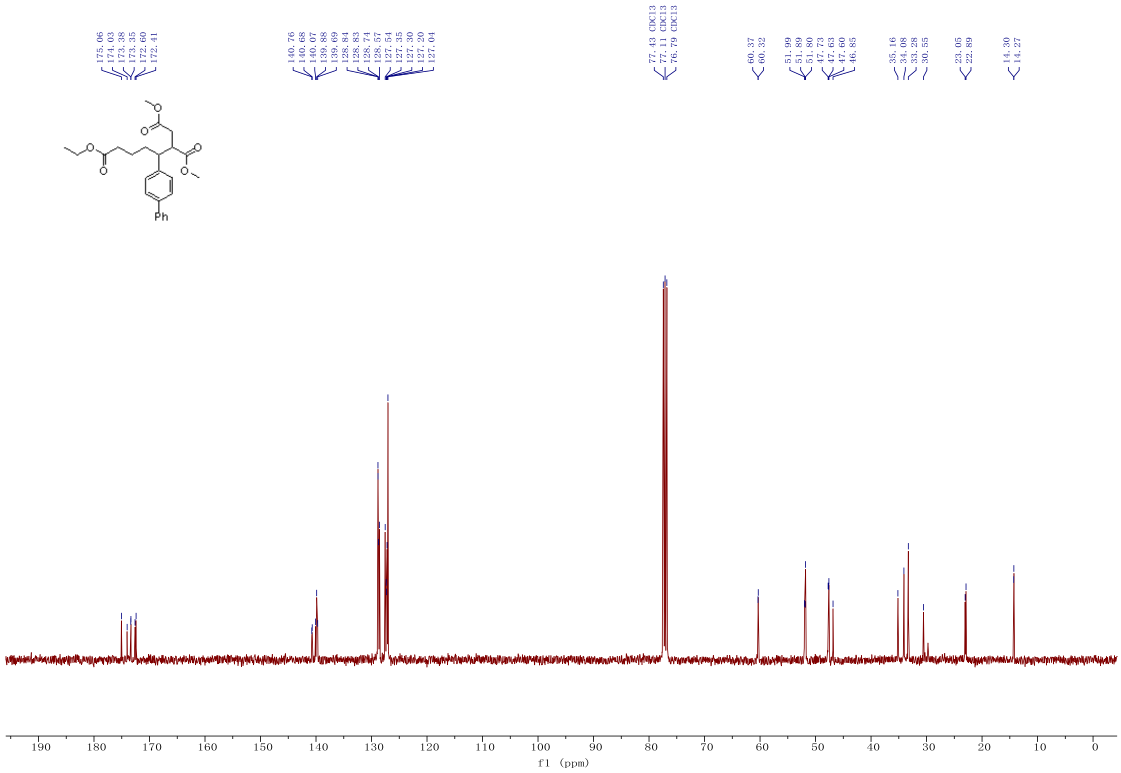

^{1}H NMR spectra of compound **d-42** in $CDCl_3$ (400 MHz): (*see procedure*)

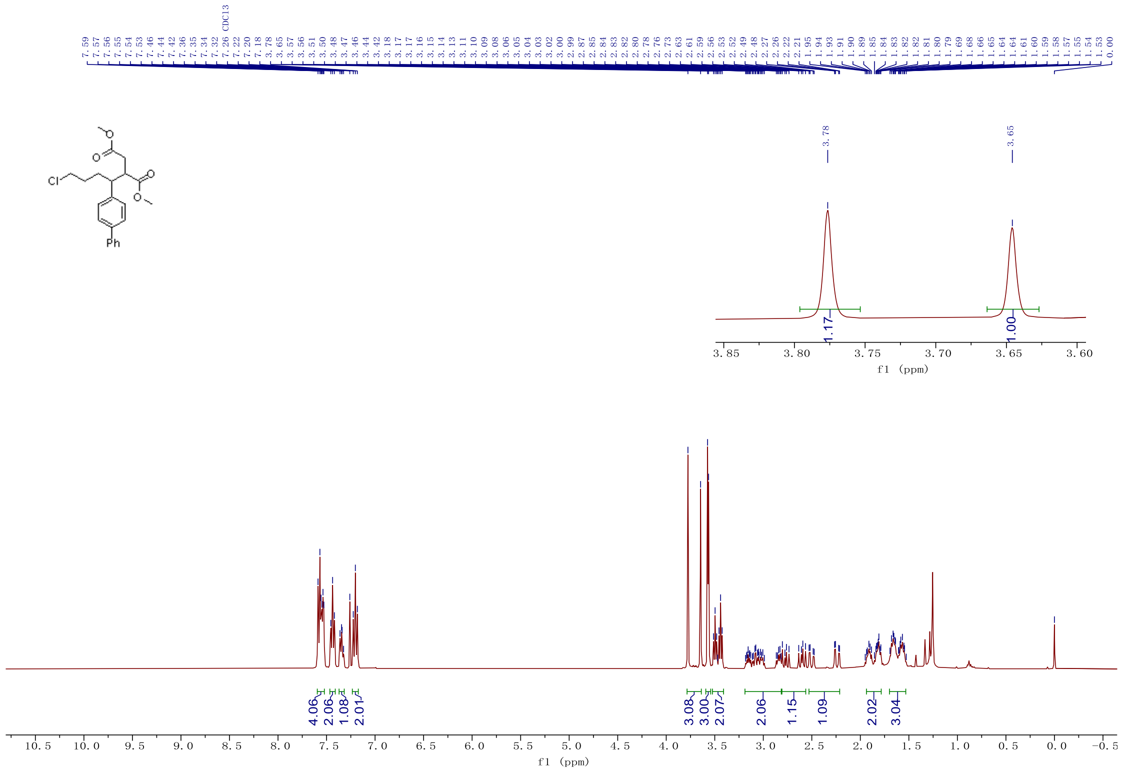

^{13}C NMR spectra of compound **d-42** in $CDCl_3$ (101 MHz): (*see procedure*)

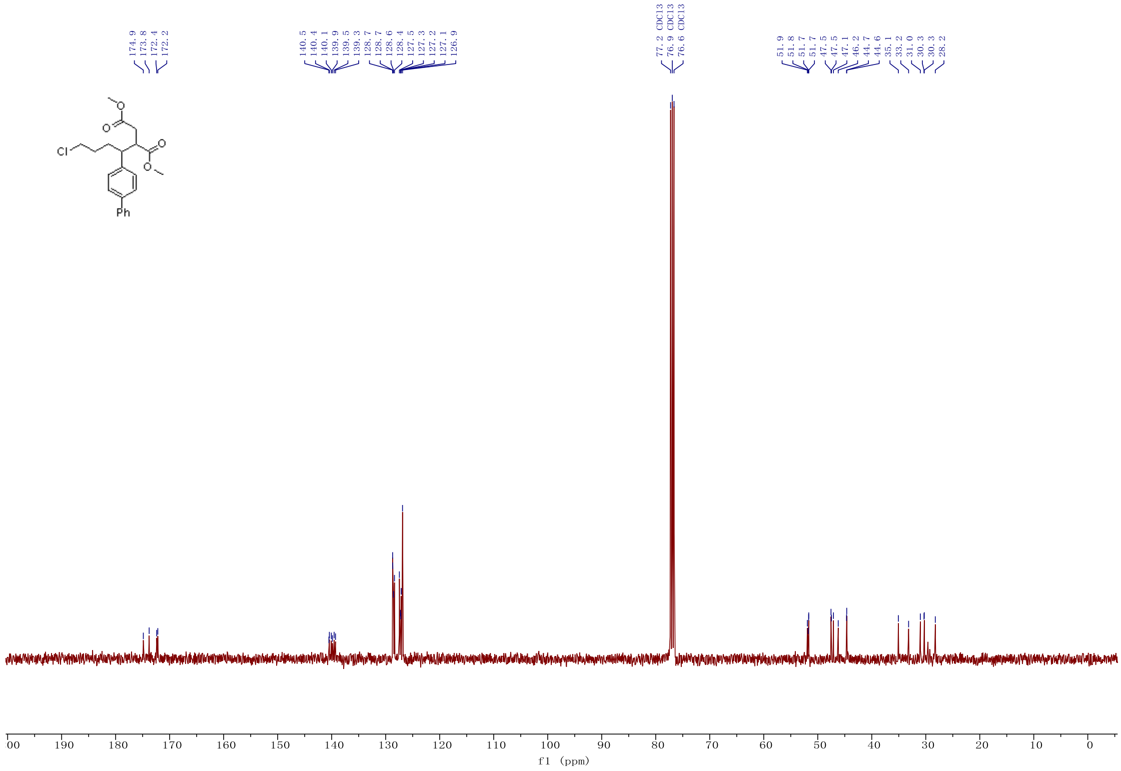

^{1}H NMR spectra of compound **d-43** in $CDCl_3$ (400 MHz): (*see procedure*)

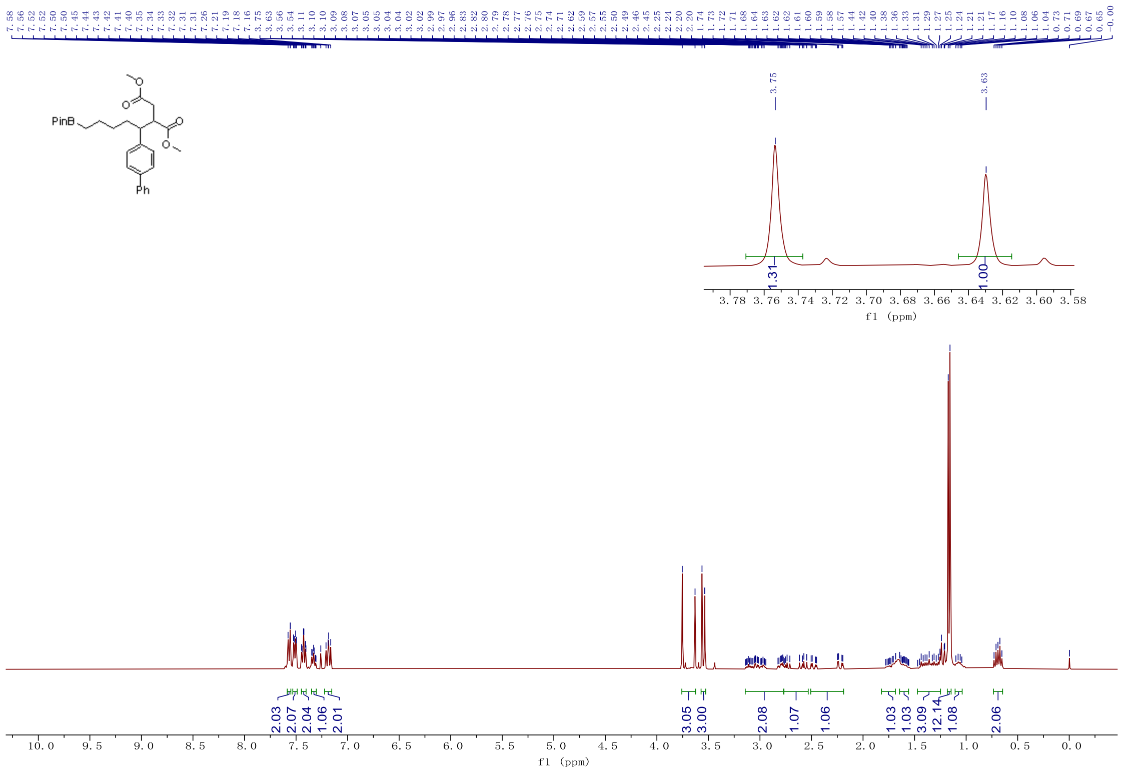

^{13}C NMR spectra of compound **d-43** in $CDCl_3$ (101 MHz): (*see procedure*)

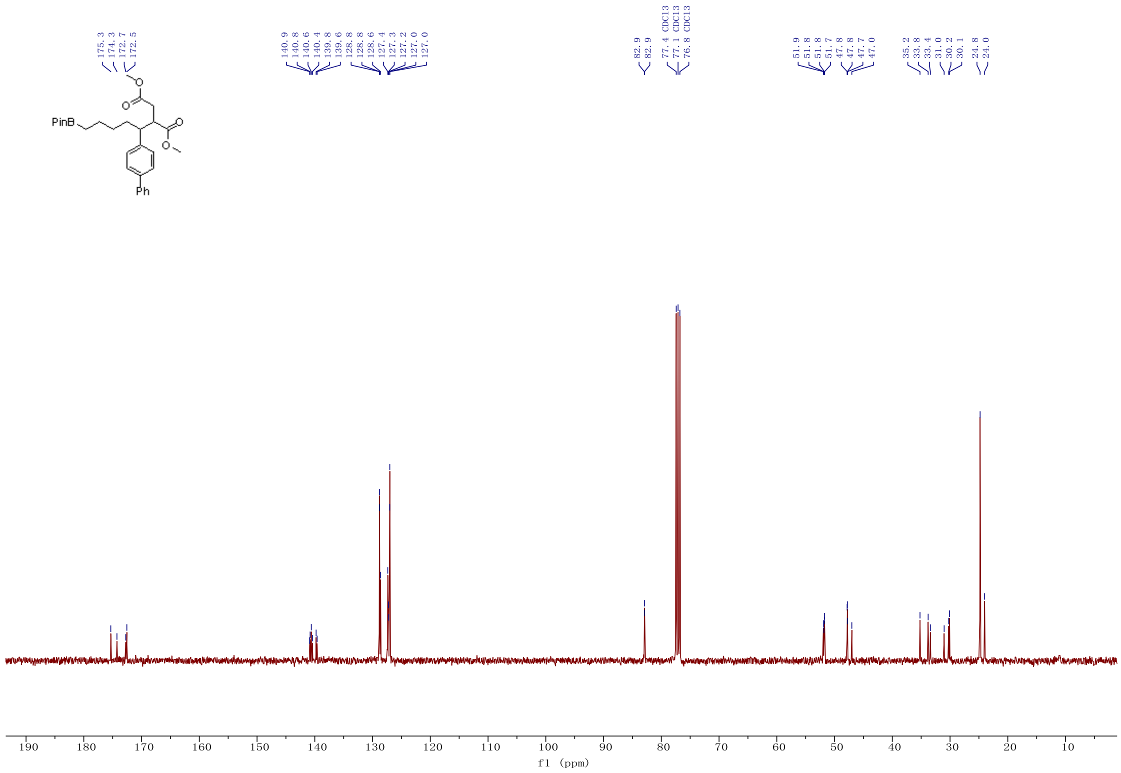

^{1}H NMR spectra of compound **d-44** in $CDCl_3$ (400 MHz): (*see procedure*)

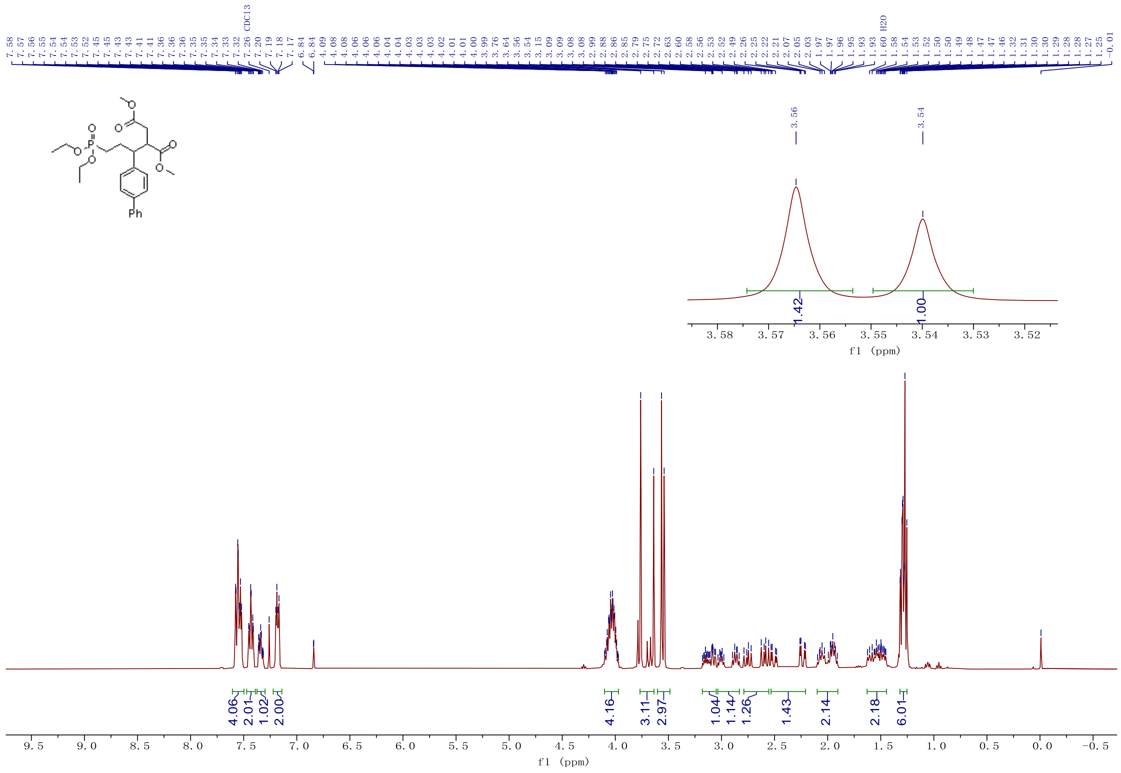

^{13}C NMR spectra of compound **d-44** in $CDCl_3$ (101 MHz): (*see procedure*)

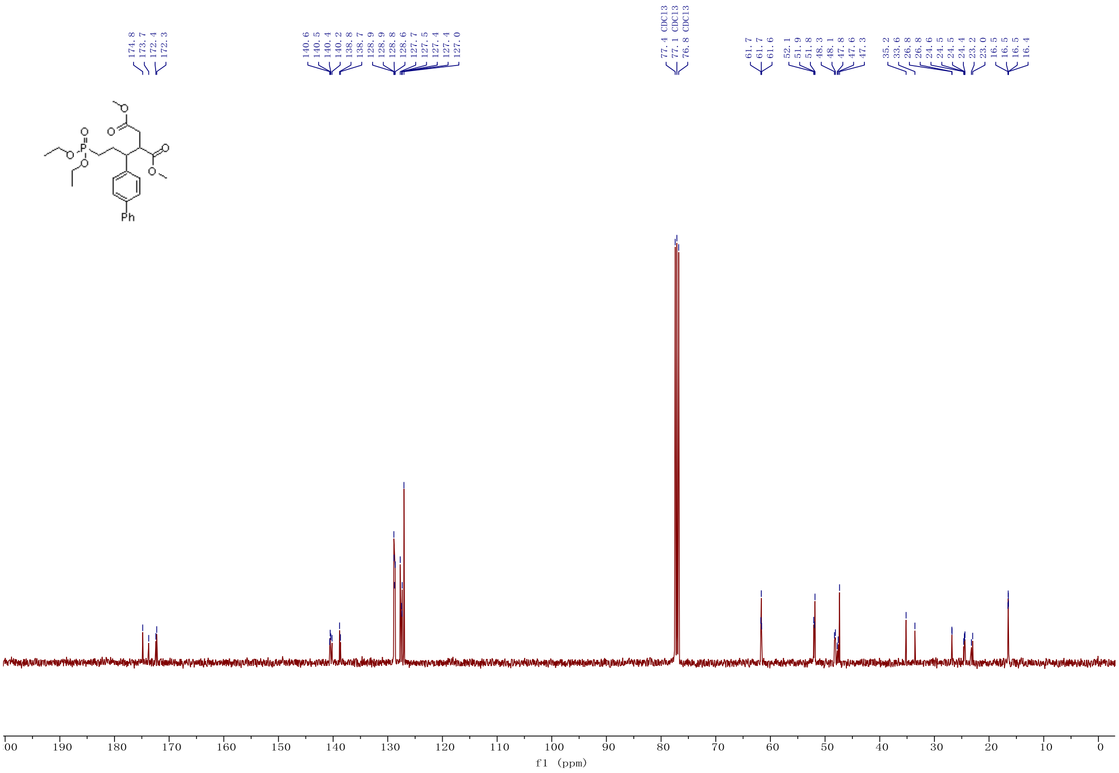

^{31}P NMR spectra of compound **d-44** in $CDCl_3$ (101 MHz): (*see procedure*)

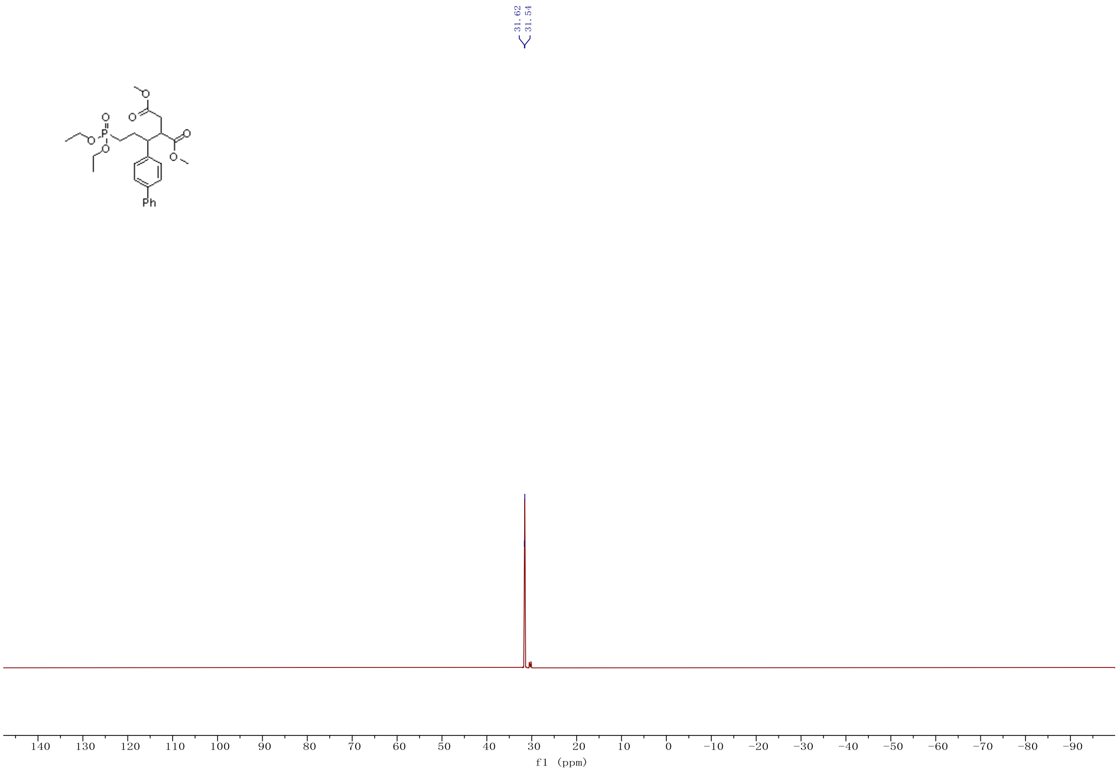

^{1}H NMR spectra of compound **d-45** in $CDCl_3$ (400 MHz): (*see procedure*)

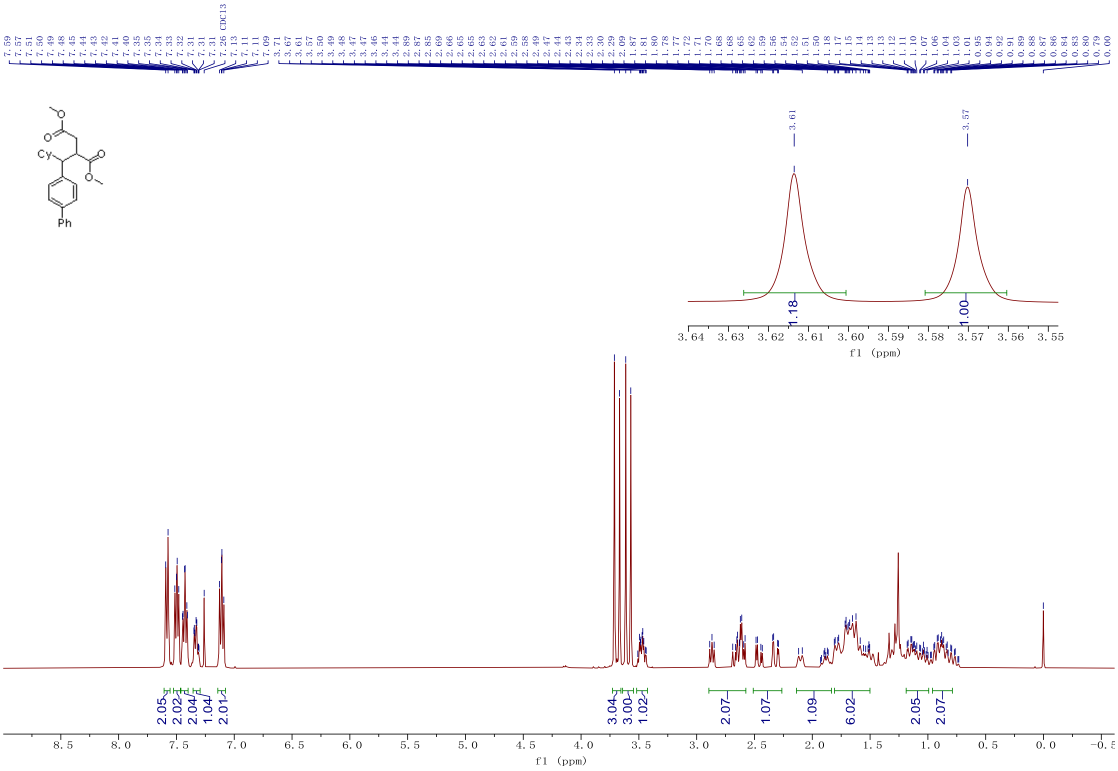

^{13}C NMR spectra of compound **d-45** in $CDCl_3$ (101 MHz): (*see procedure*)

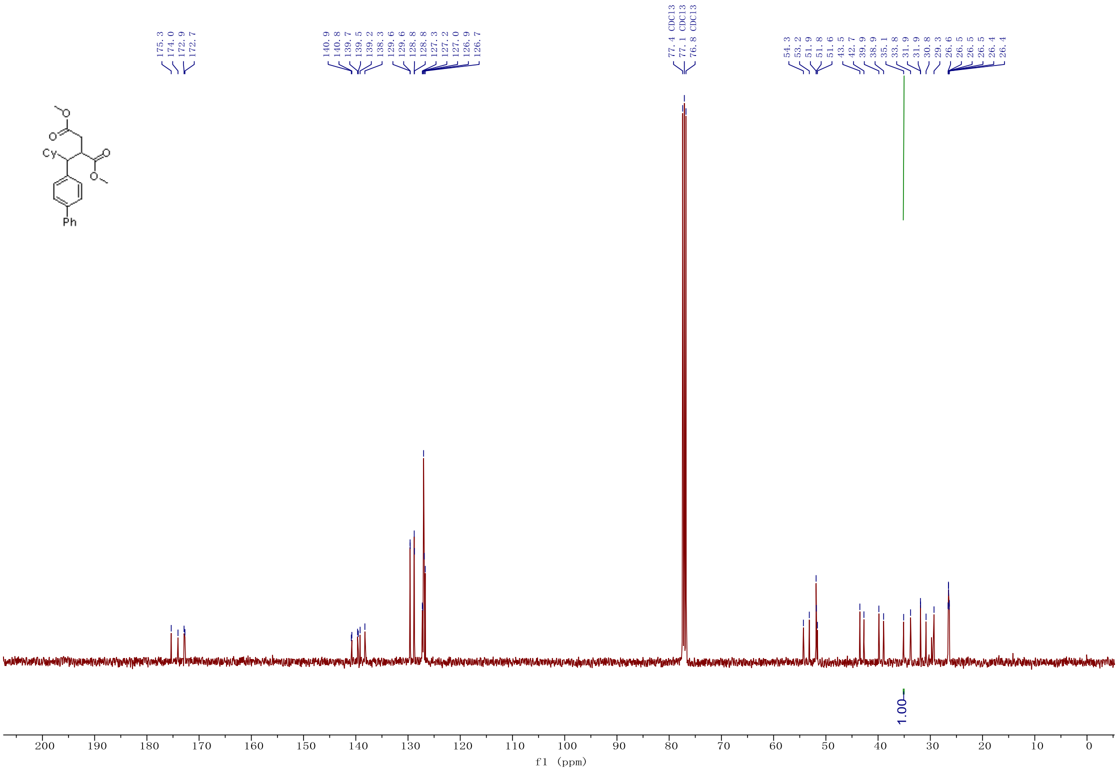

^{1}H NMR spectra of compound **d-46** in $CDCl_3$ (400 MHz): (<u>see procedure</u>)

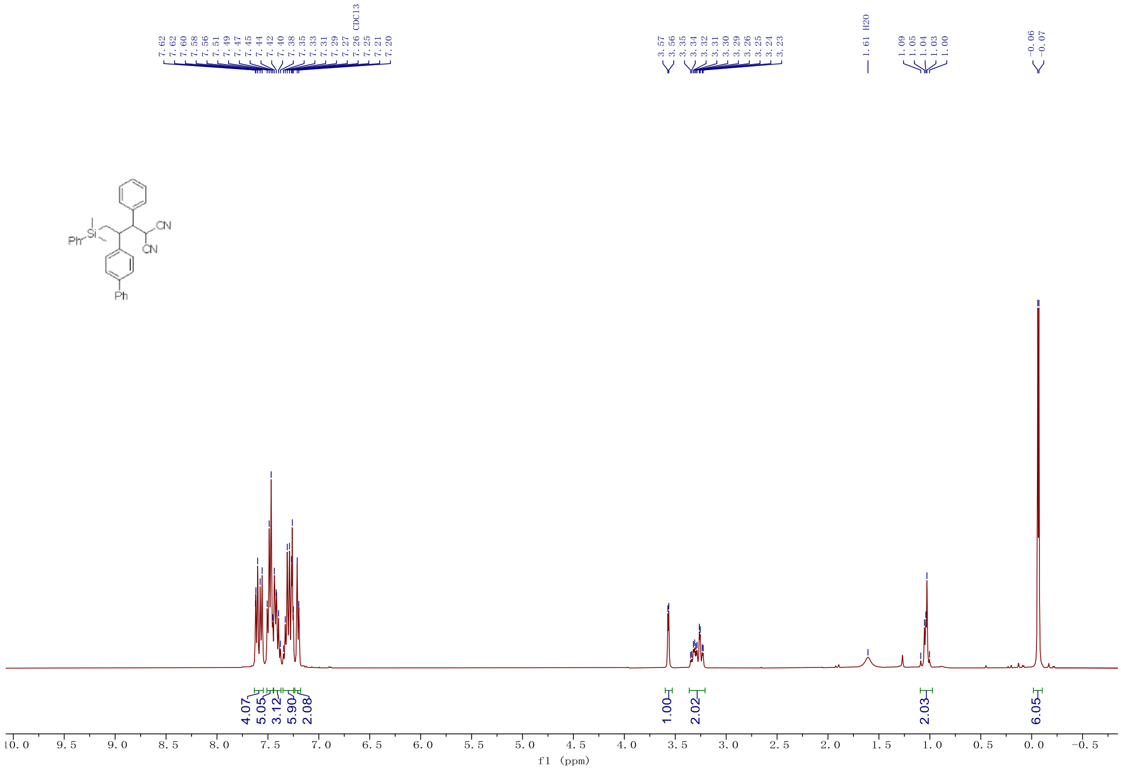

^{13}C NMR spectra of compound **d-46** in $CDCl_3$ (101 MHz): (<u>see procedure</u>)

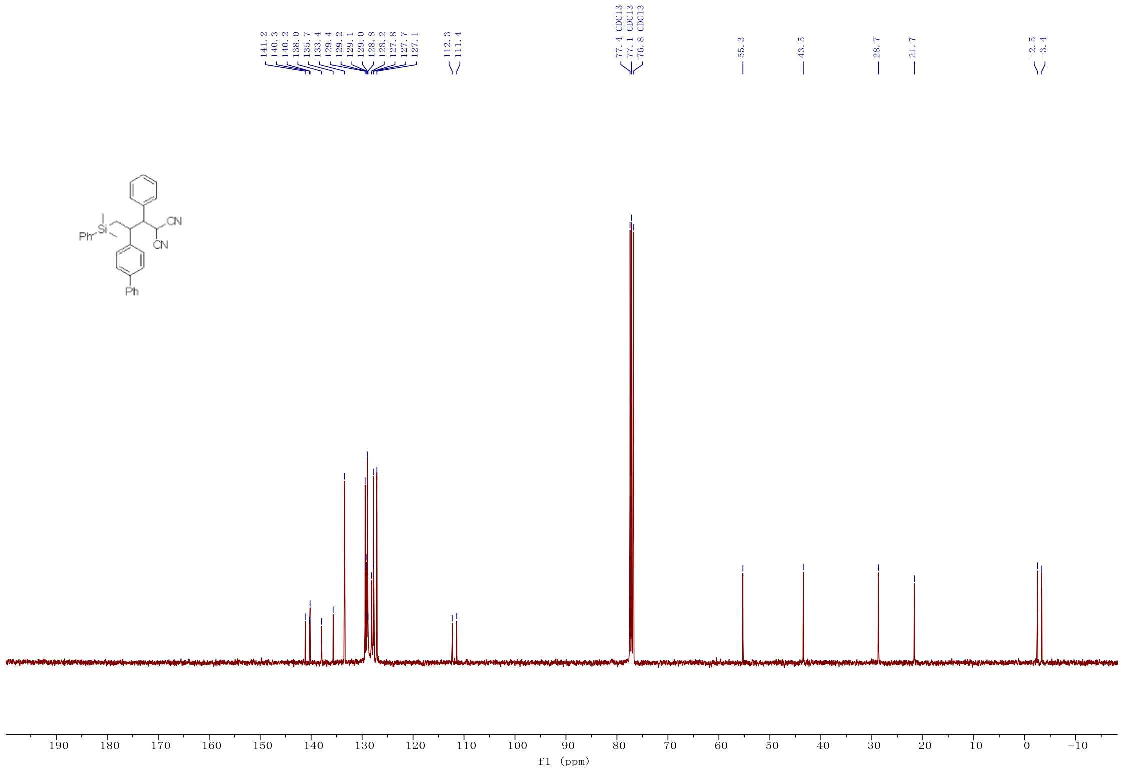

^{1}H NMR spectra of compound **d-46’** in $CDCl_3$ (400 MHz): (*see procedure*)

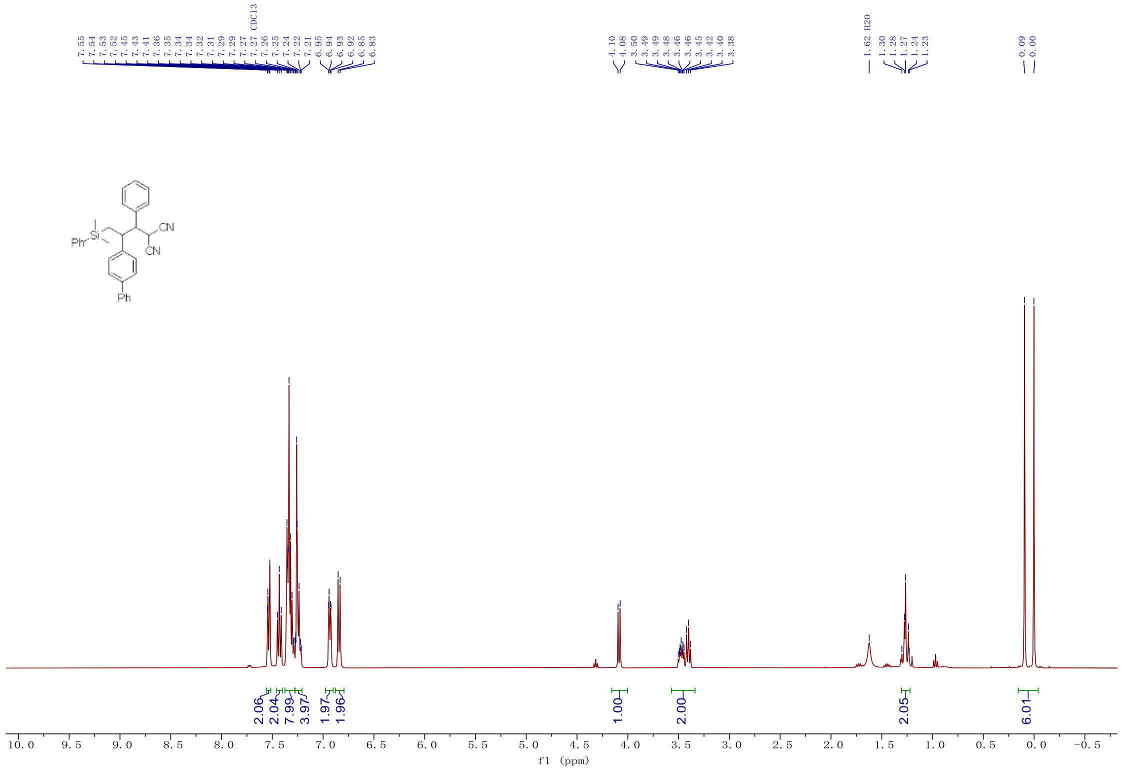

^{13}C NMR spectra of compound **d-46’** in $CDCl_3$ (101 MHz): (*see procedure*)

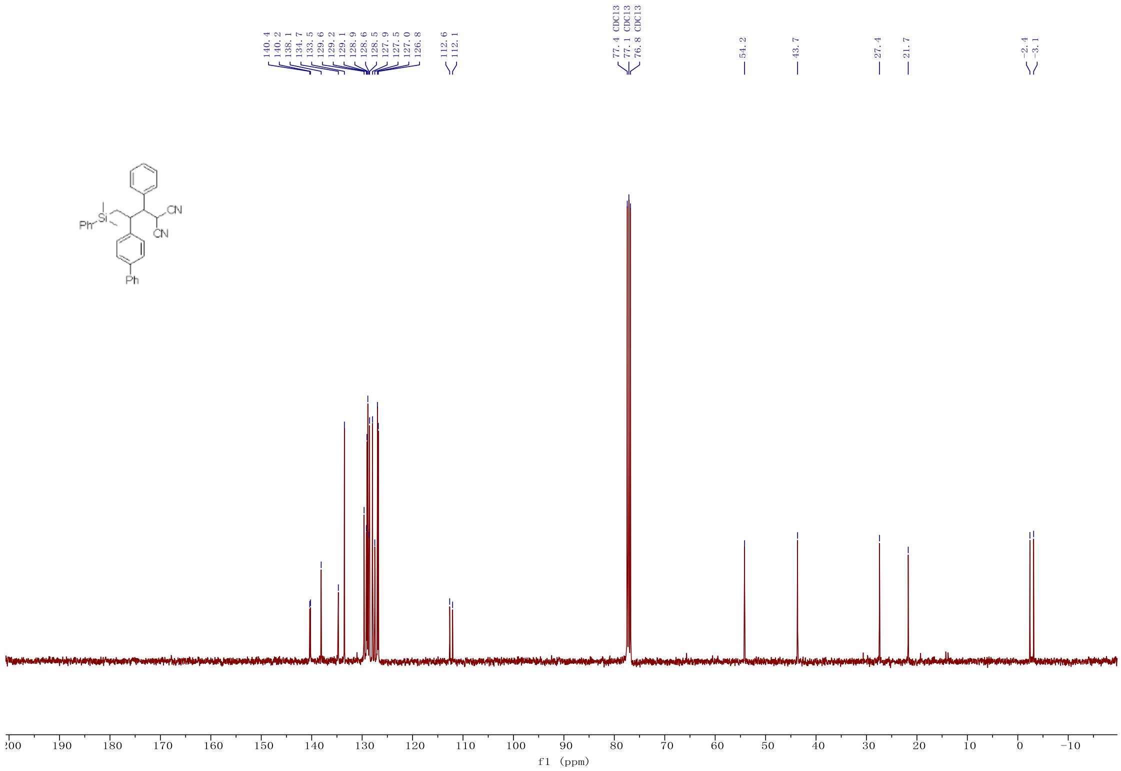

^{1}H NMR spectra of compound **d-47** in $CDCl_3$ (400 MHz): (see procedure)

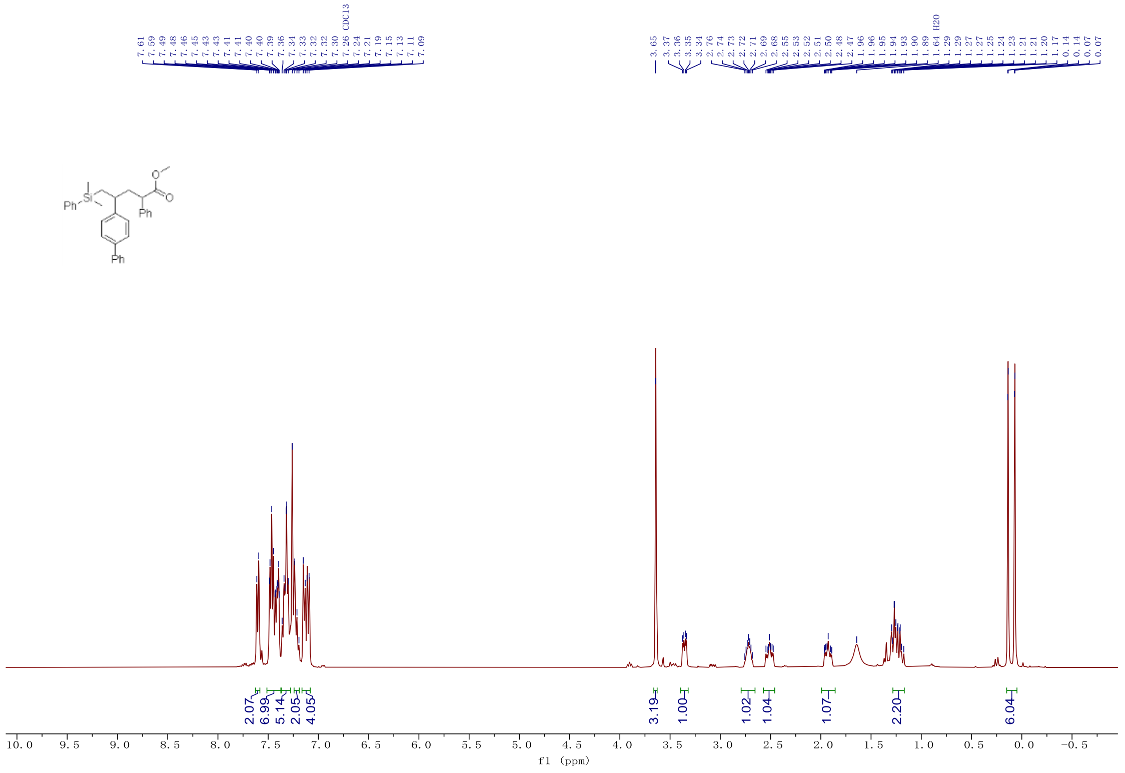

^{13}C NMR spectra of compound **d-47** in $CDCl_3$ (101 MHz): (see procedure)

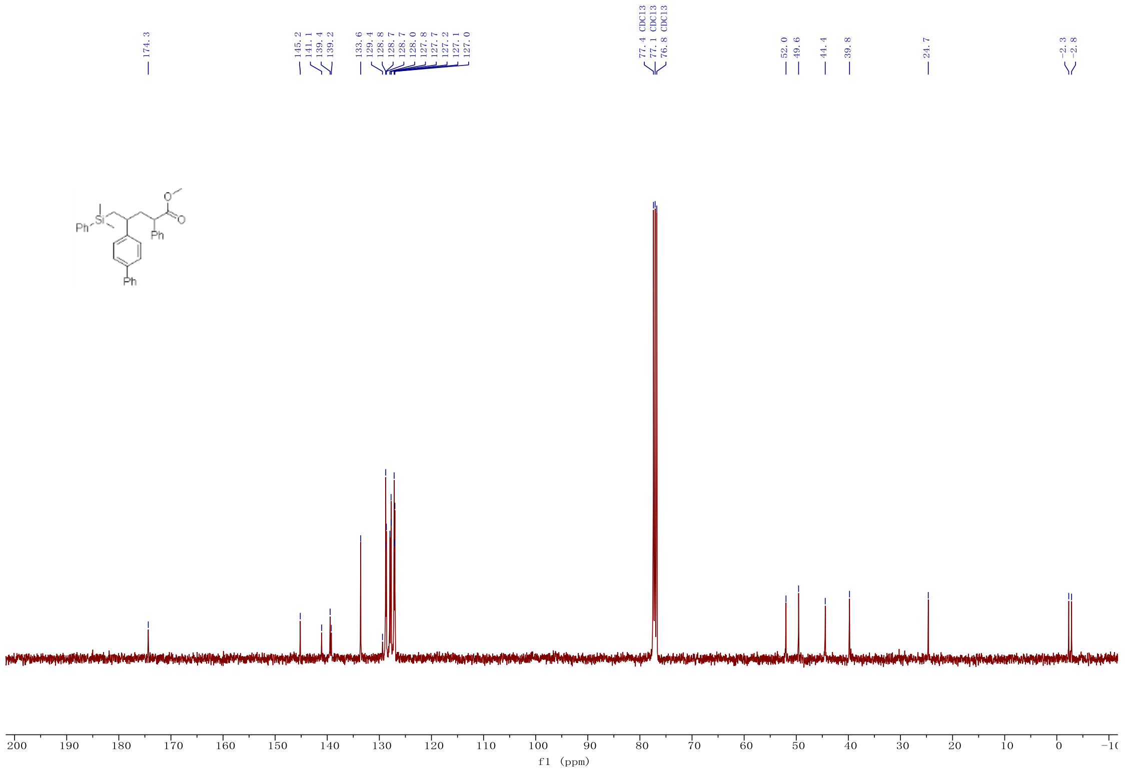

^{1}H NMR spectra of compound **d-47’** in $CDCl_3$ (400 MHz): (*see procedure*)

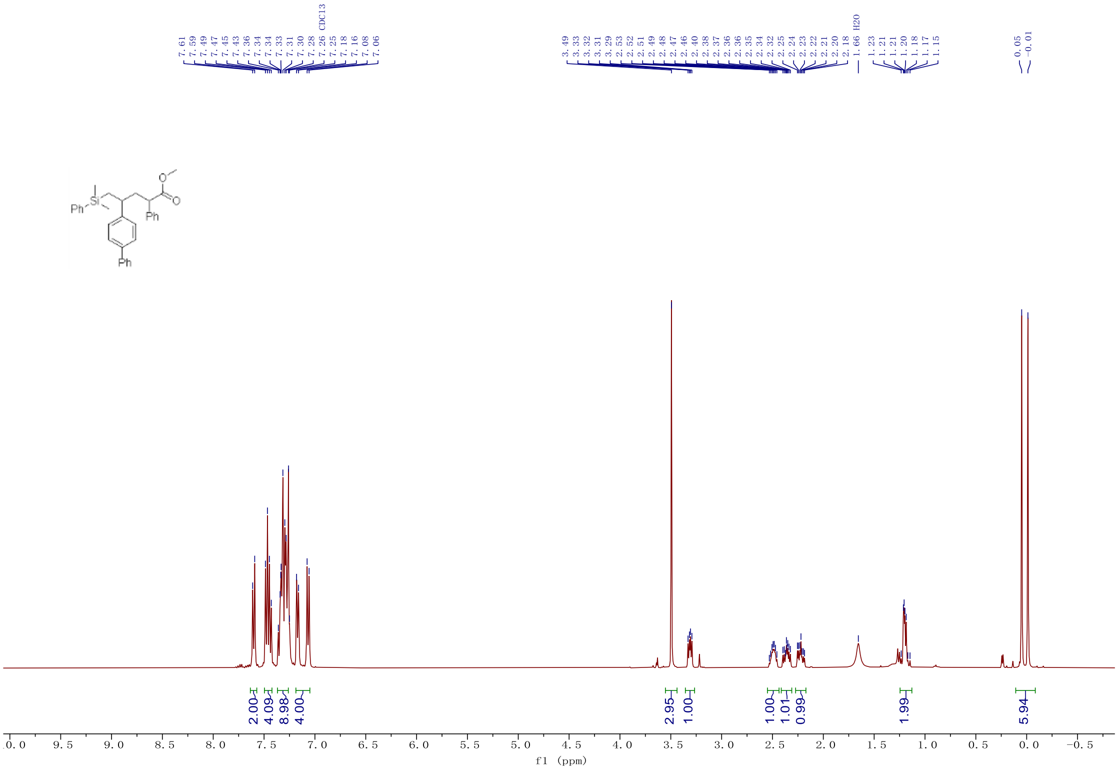

^{13}C NMR spectra of compound **d-47’** in $CDCl_3$ (101 MHz): (*see procedure*)

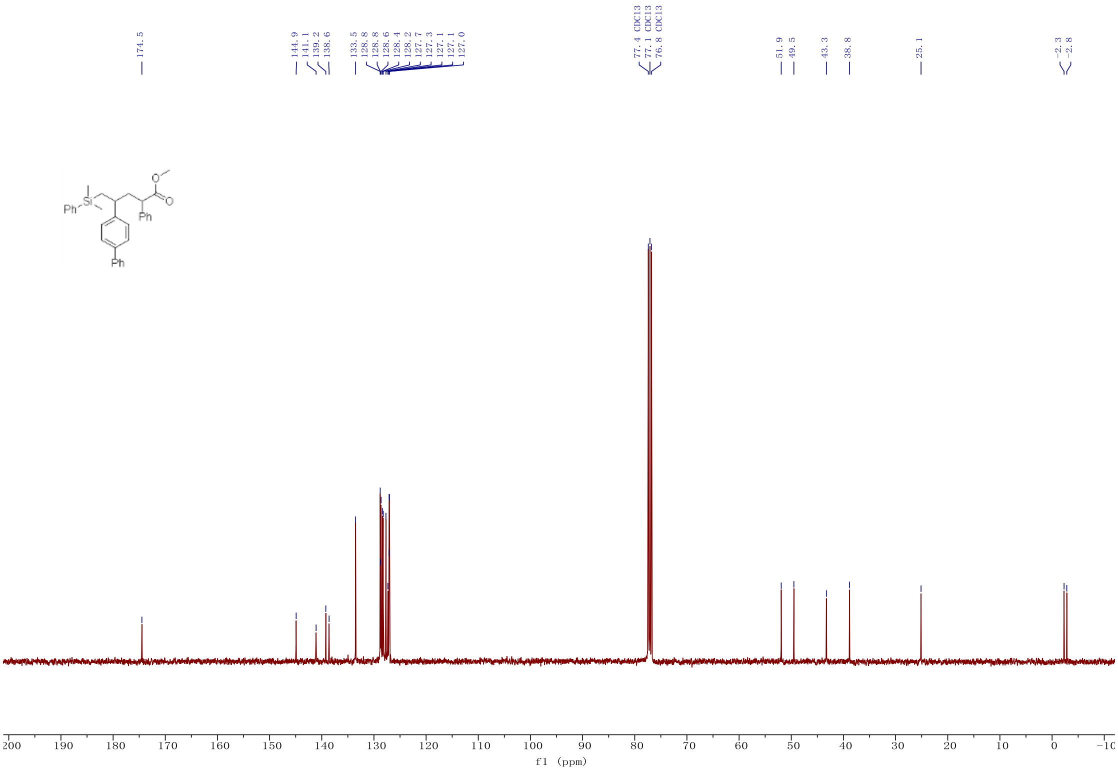

^{1}H NMR spectra of compound **d-48** in $CDCl_3$ (400 MHz): (*see procedure*)

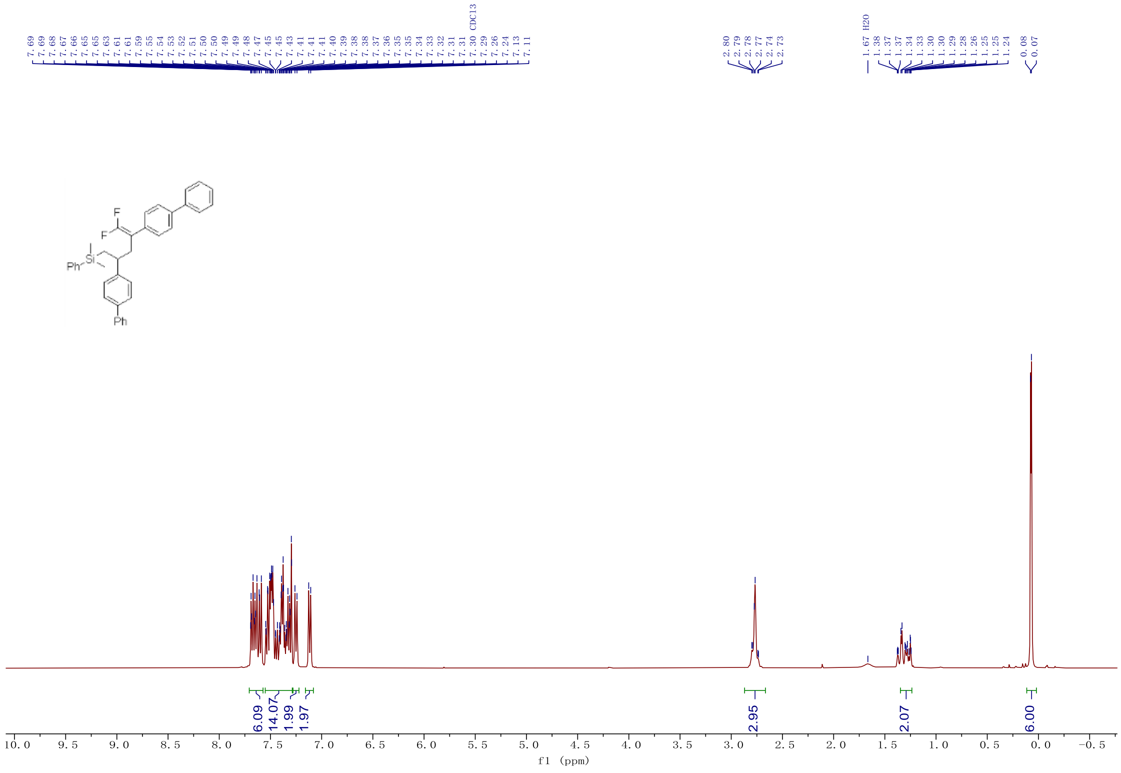

^{13}C NMR spectra of compound **d-48** in $CDCl_3$ (101 MHz): (*see procedure*)

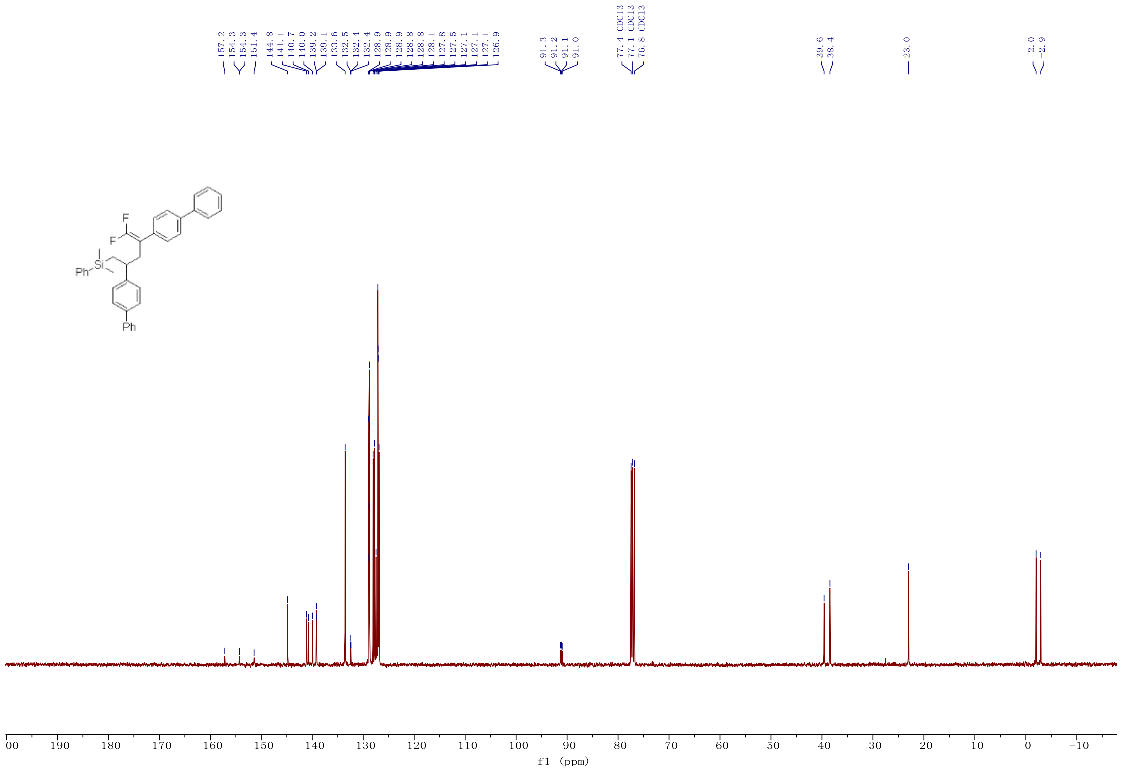

^{19}F NMR spectra of compound **d-48** in $CDCl_3$ (376 MHz): (*see procedure*)

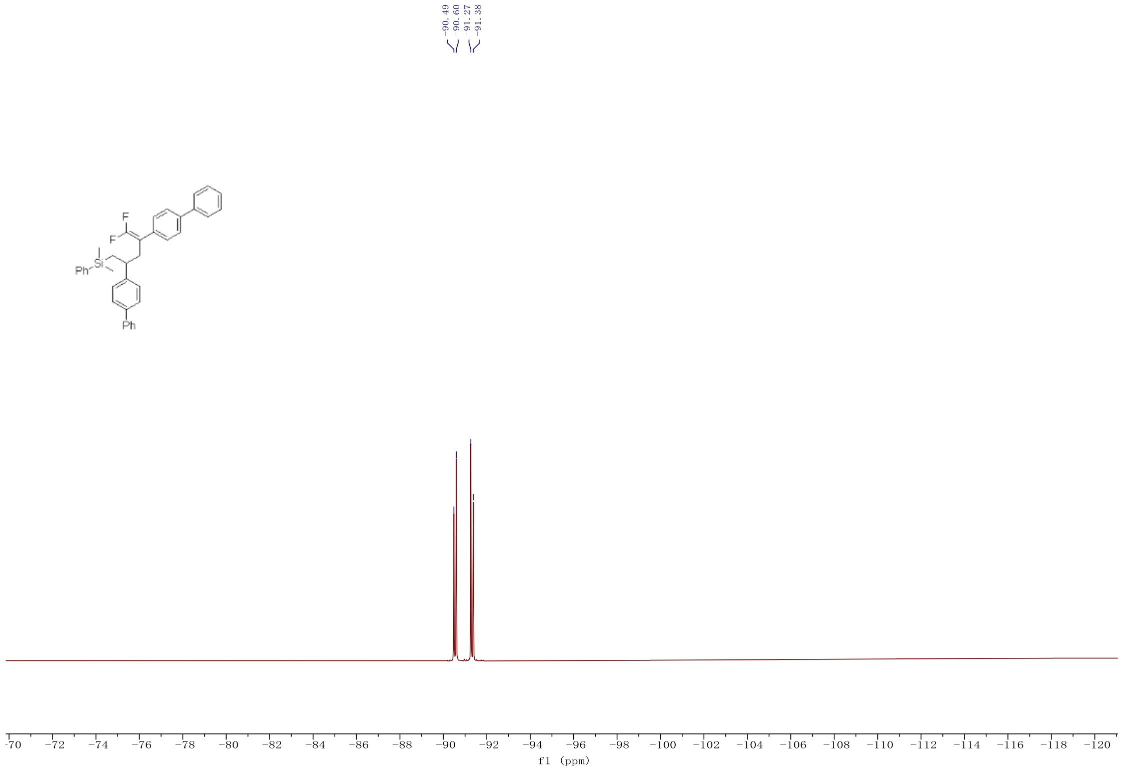

^{1}H NMR spectra of compound **d-49** in $CDCl_3$ (400 MHz): (*see procedure*)

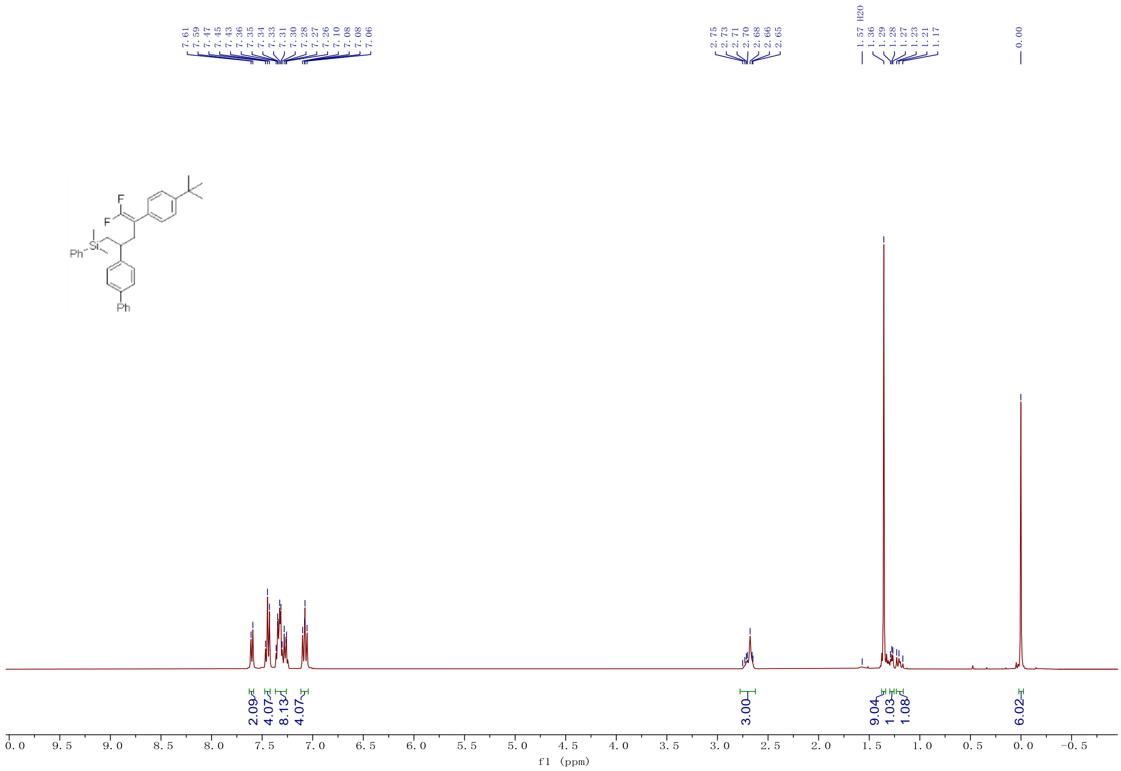

^{13}C NMR spectra of compound **d-49** in $CDCl_3$ (101 MHz): (see procedure)

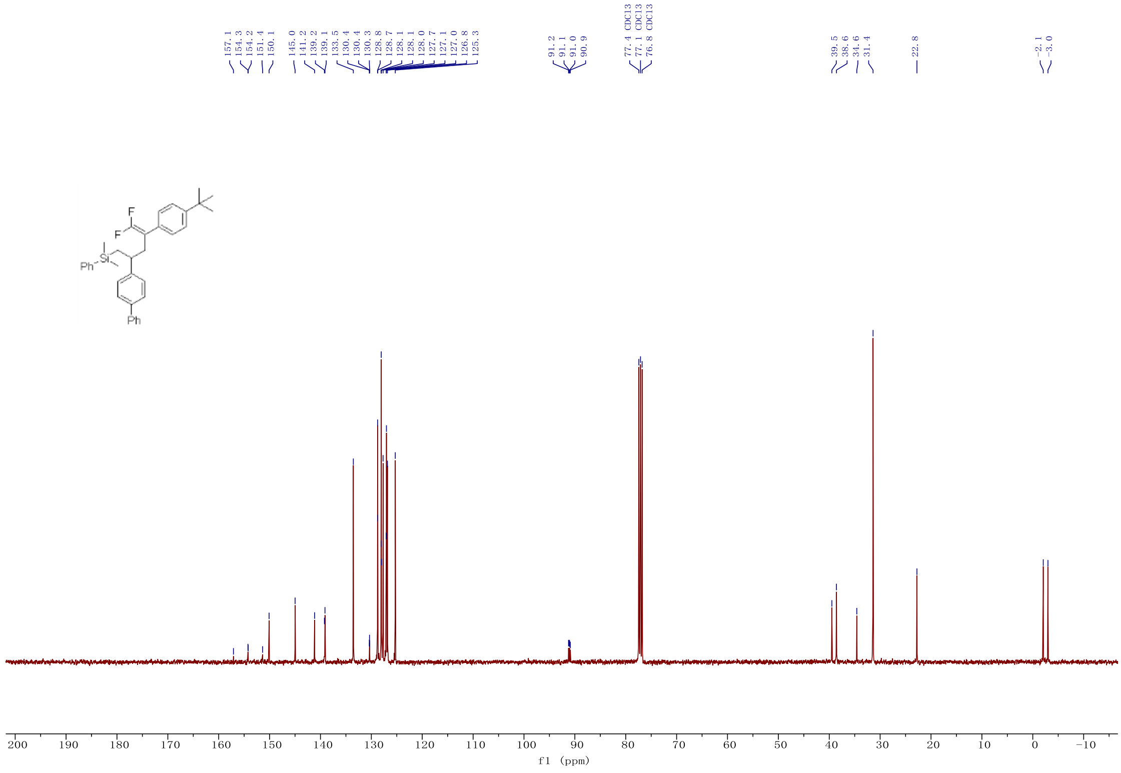

^{19}F NMR spectra of compound **d-49** in $CDCl_3$ (376 MHz): (see procedure)

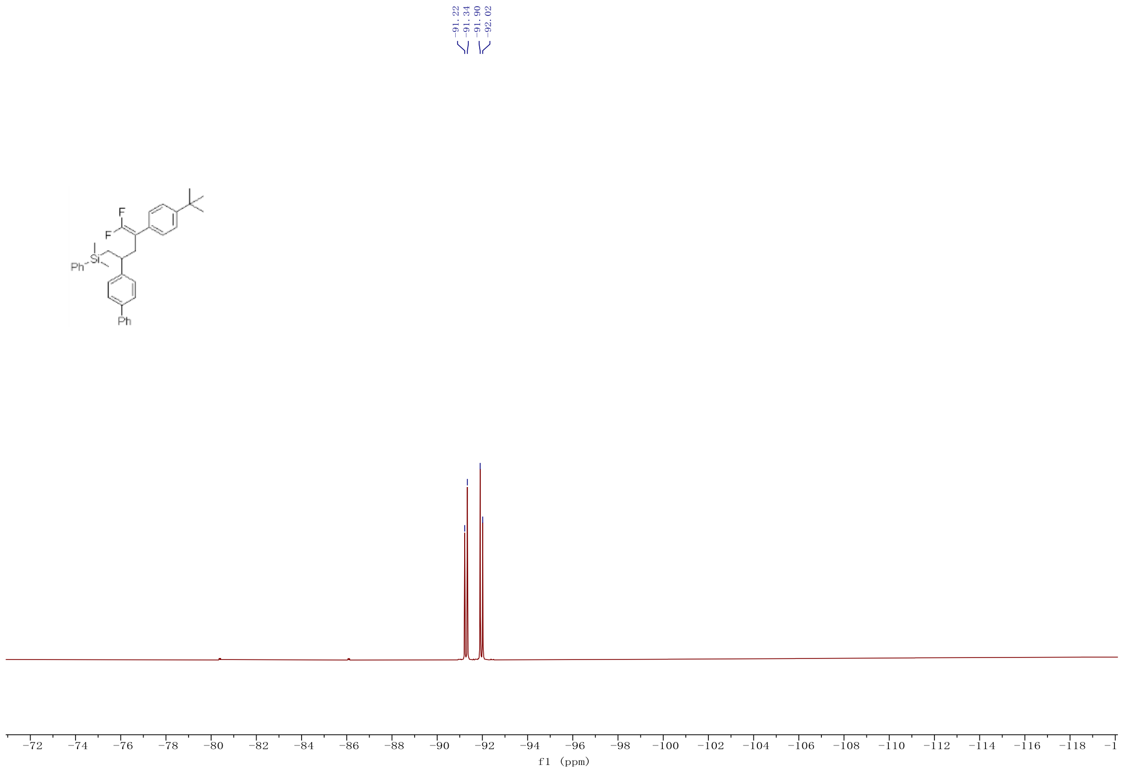

^{1}H NMR spectra of compound **d-50** in $CDCl_3$ (400 MHz): (*see procedure*)

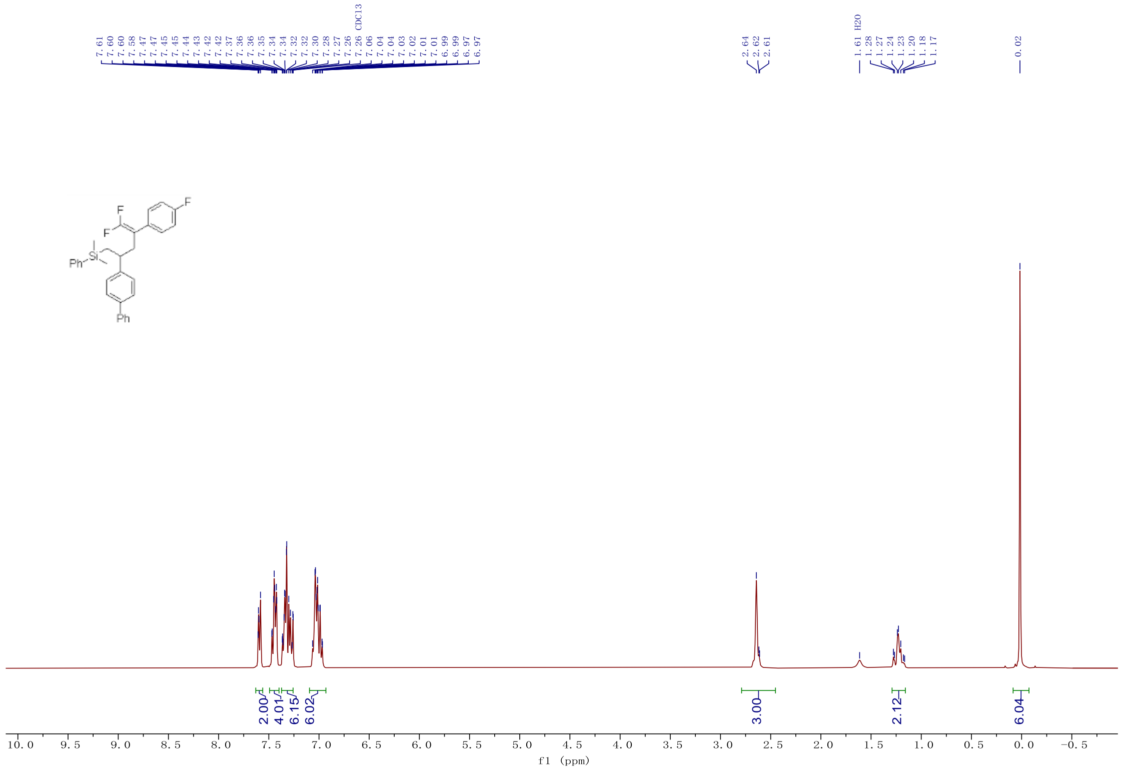

^{13}C NMR spectra of compound **d-50** in $CDCl_3$ (101 MHz): (*see procedure*)

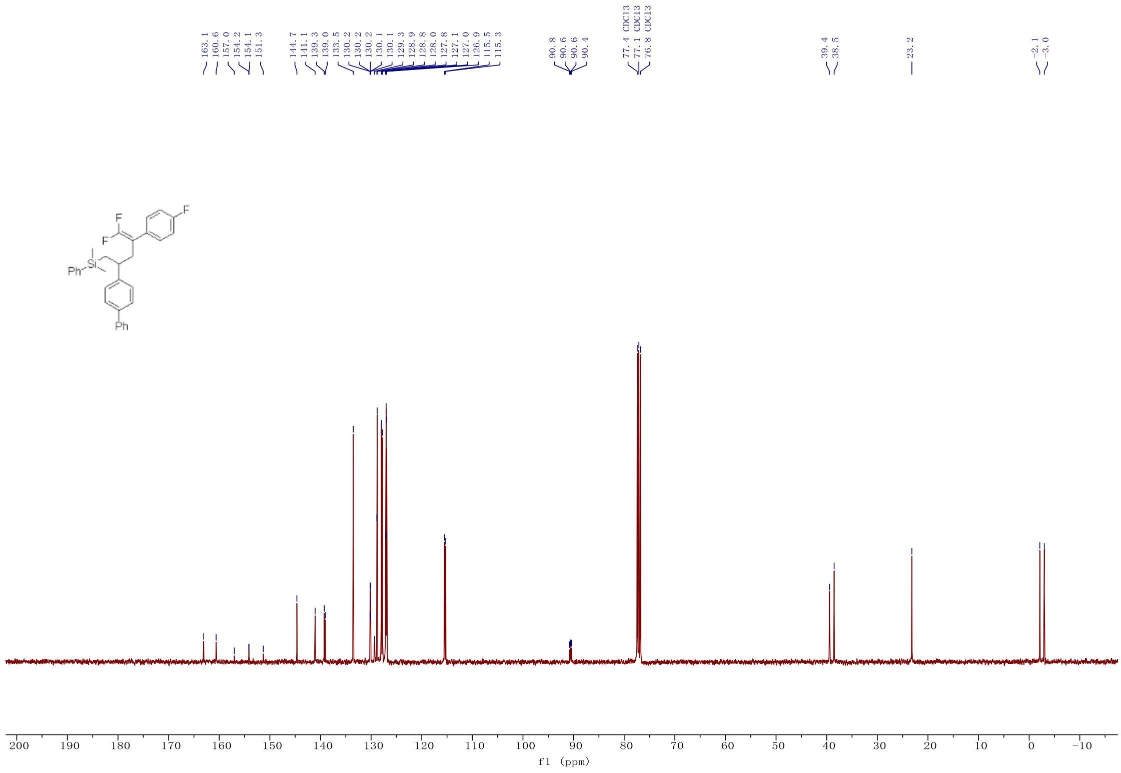

^{19}F NMR spectra of compound **d-50** in $CDCl_3$ (376 MHz): (*see procedure*)

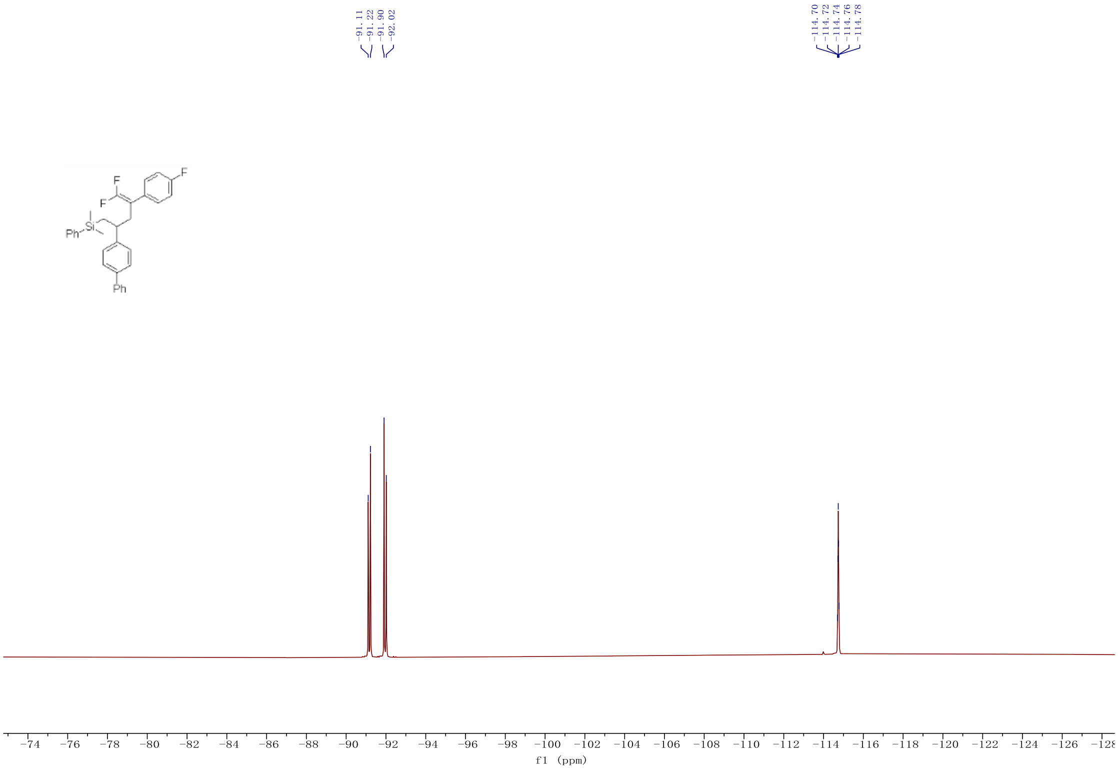

^{1}H NMR spectra of compound **d-51** in $CDCl_3$ (400 MHz): (*see procedure*)

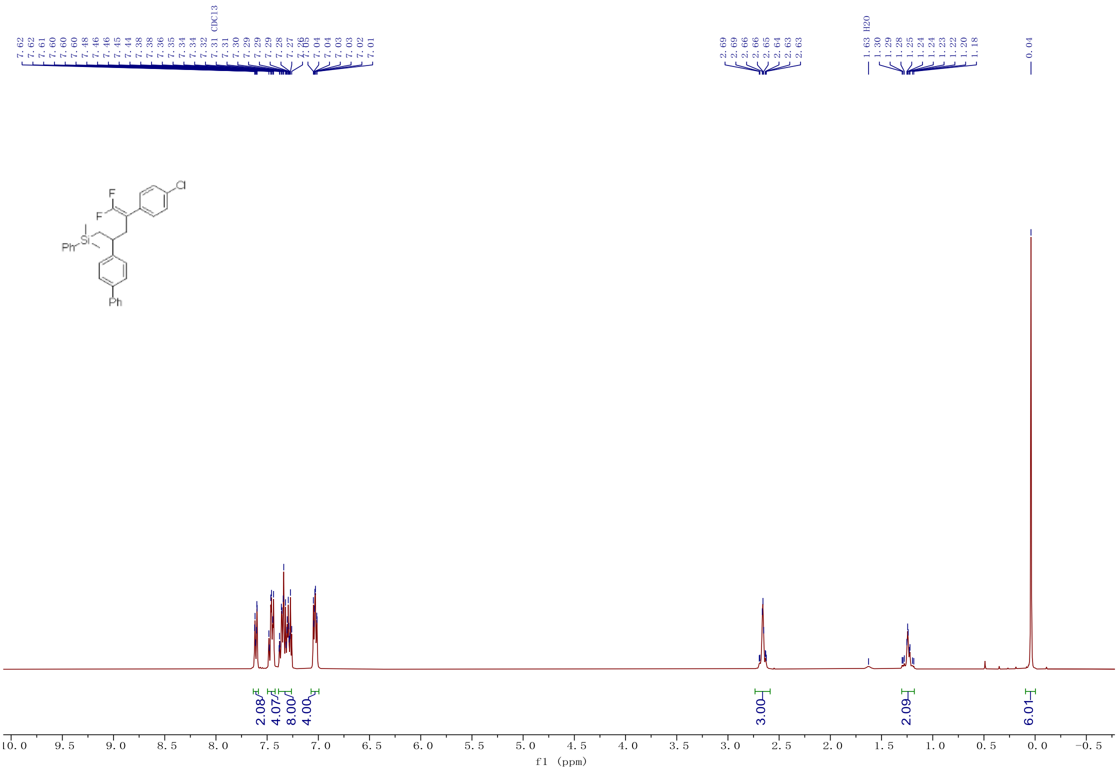

^{13}C NMR spectra of compound **d-51** in $CDCl_3$ (101 MHz): (*see procedure*)

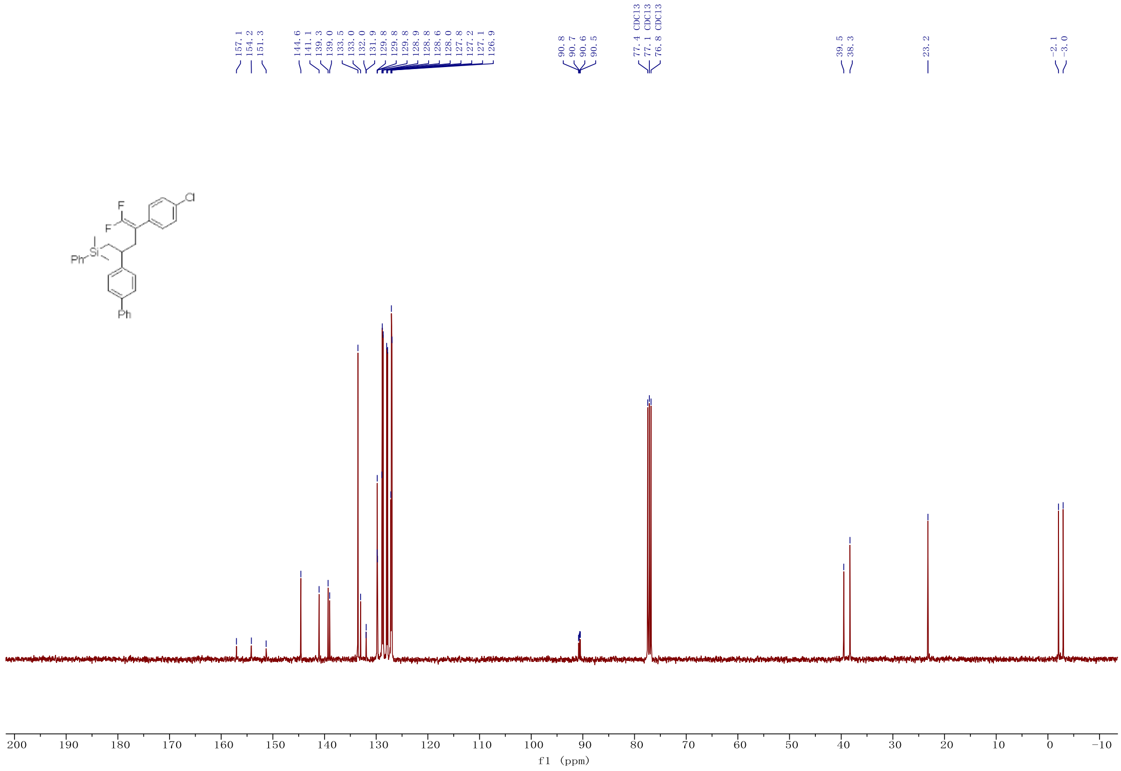

^{19}F NMR spectra of compound **d-51** in $CDCl_3$ (376 MHz): (*see procedure*)

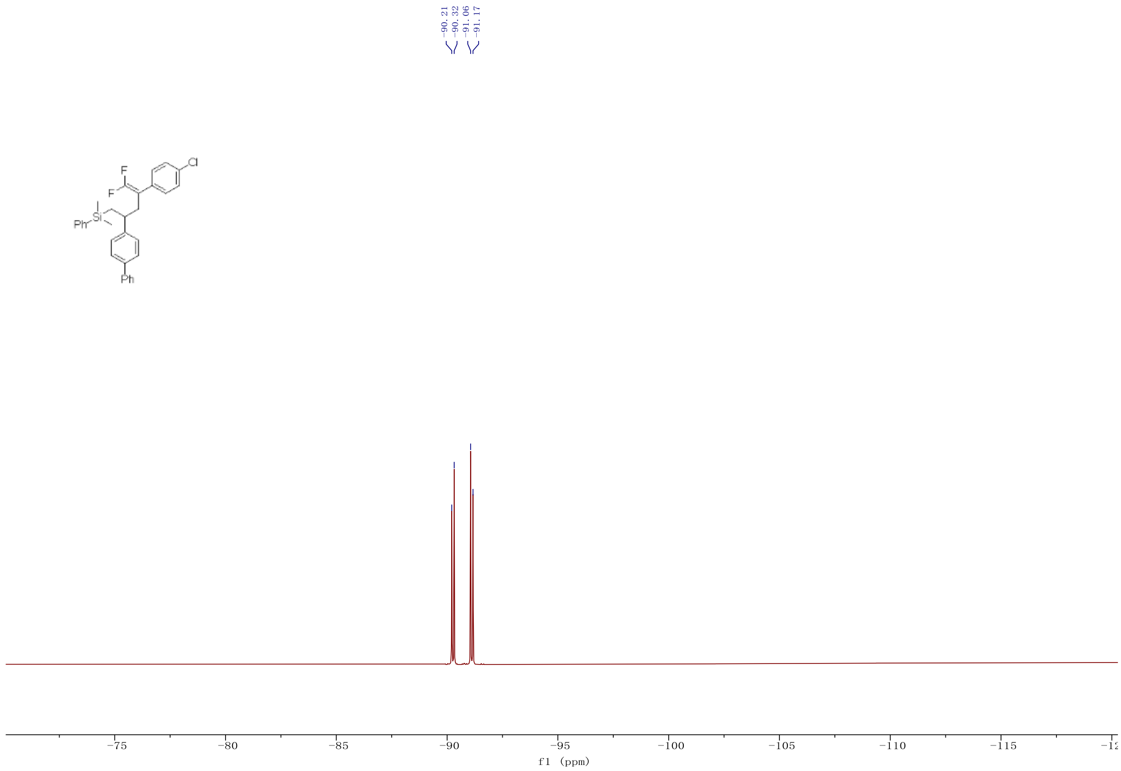

^{1}H NMR spectra of compound **d-52** in $CDCl_3$ (400 MHz): (*see procedure*)

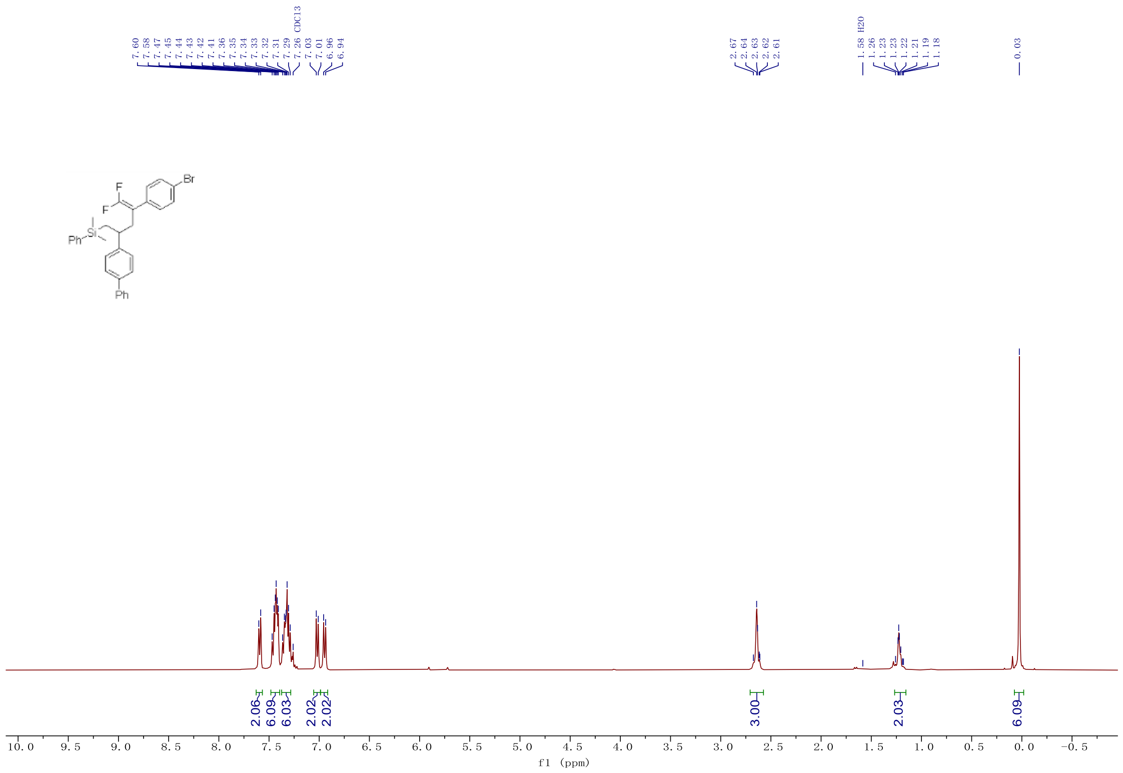

^{13}C NMR spectra of compound **d-52** in $CDCl_3$ (101 MHz): (*see procedure*)

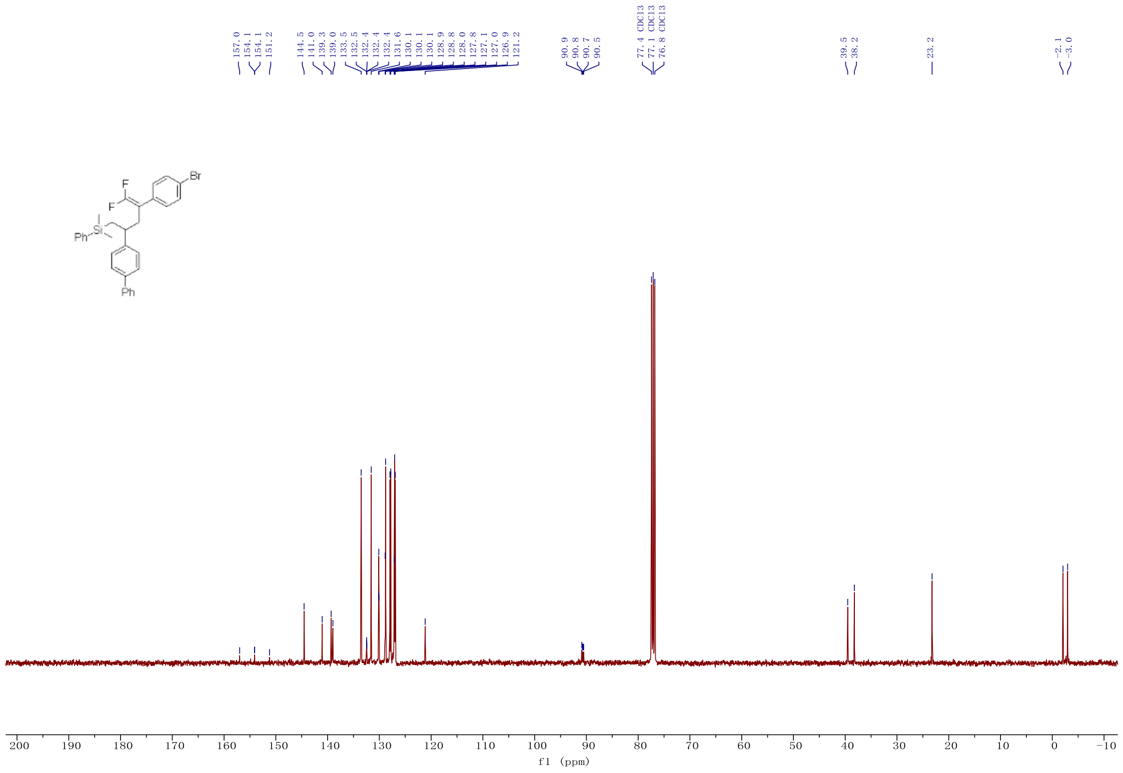

^{19}F NMR spectra of compound **d-52** in $CDCl_3$ (376 MHz): (*see procedure*)

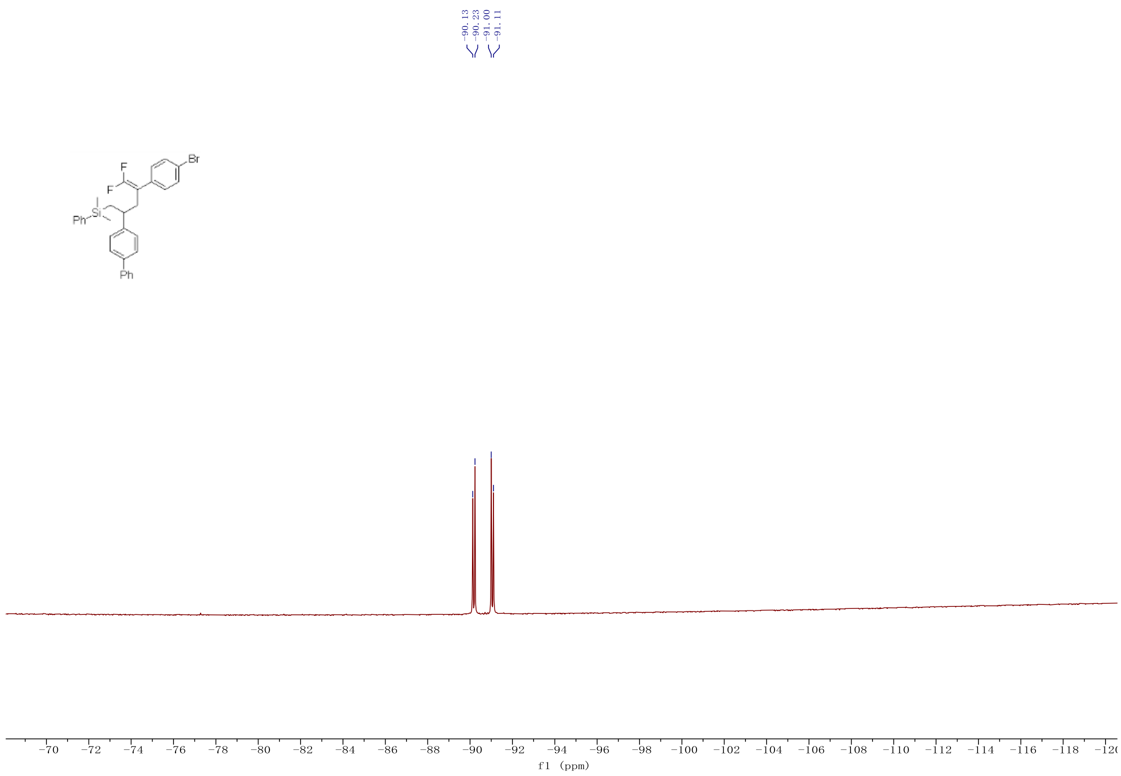

^{1}H NMR spectra of compound **d-53** in $CDCl_3$ (400 MHz): (*see procedure*)

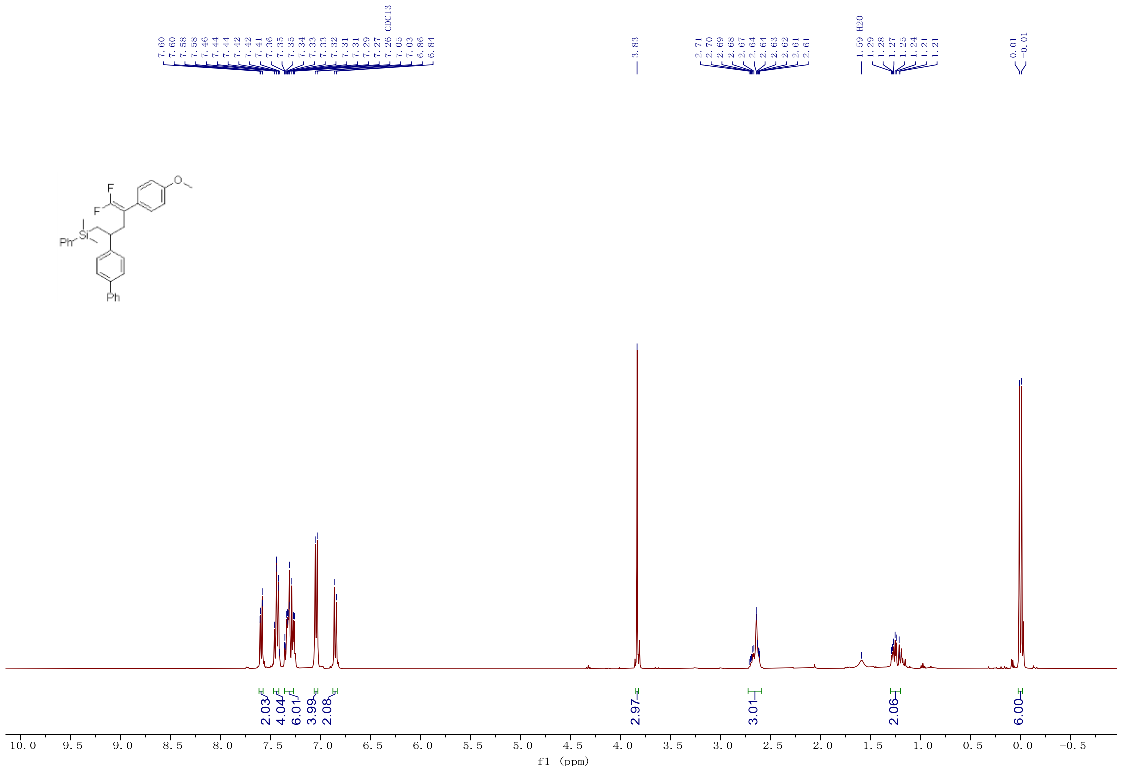

^{13}C NMR spectra of compound **d-53** in $CDCl_3$ (101 MHz): (*see procedure*)

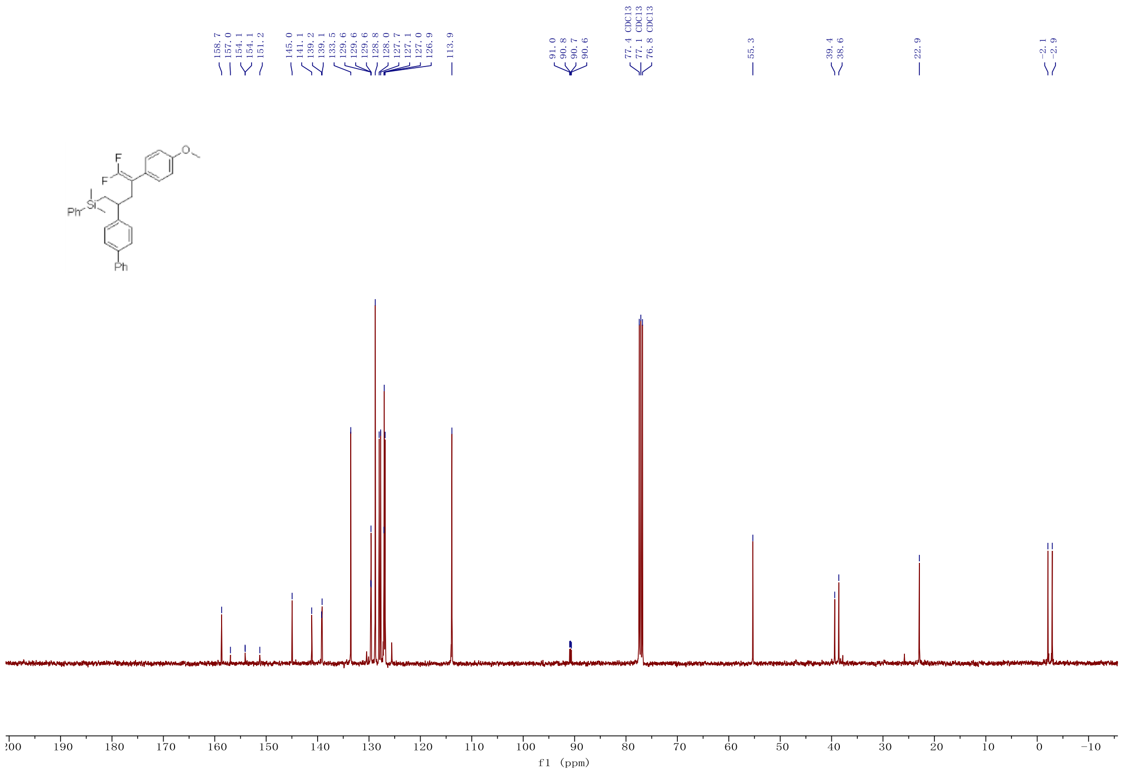

^{19}F NMR spectra of compound **d-53** in $CDCl_3$ (376 MHz): (*see procedure*)

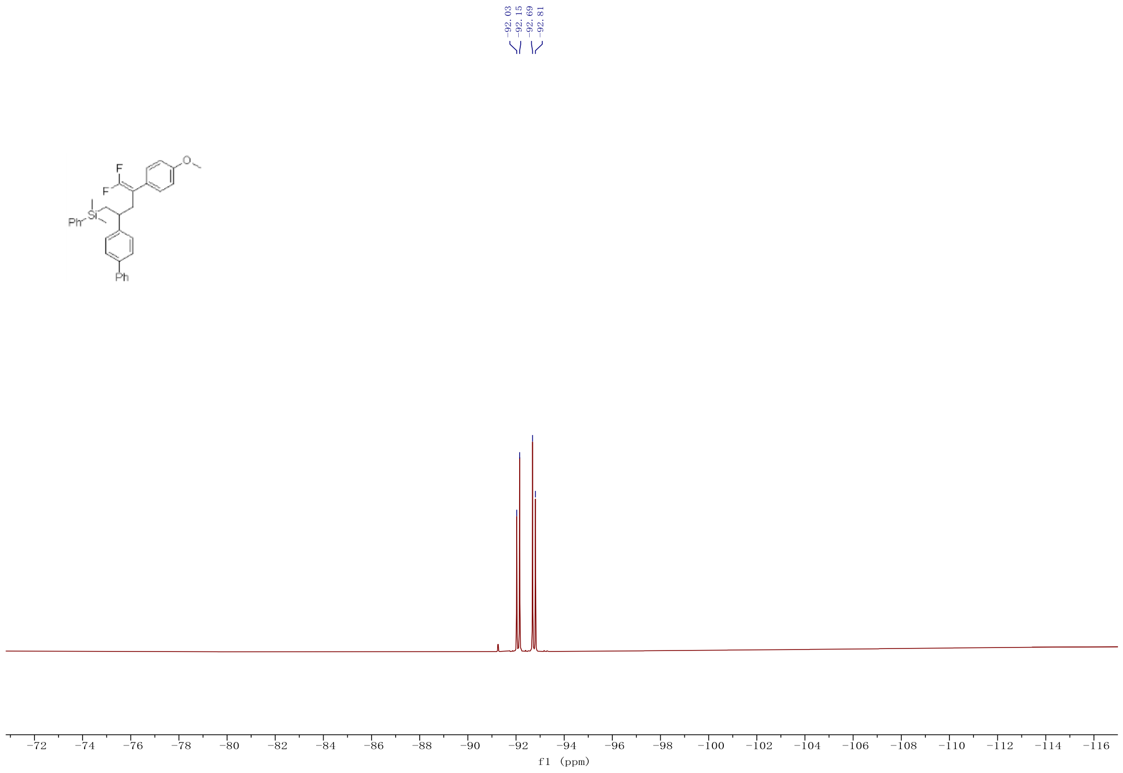

^{1}H NMR spectra of compound **d-54** in $CDCl_3$ (400 MHz): (see procedure)

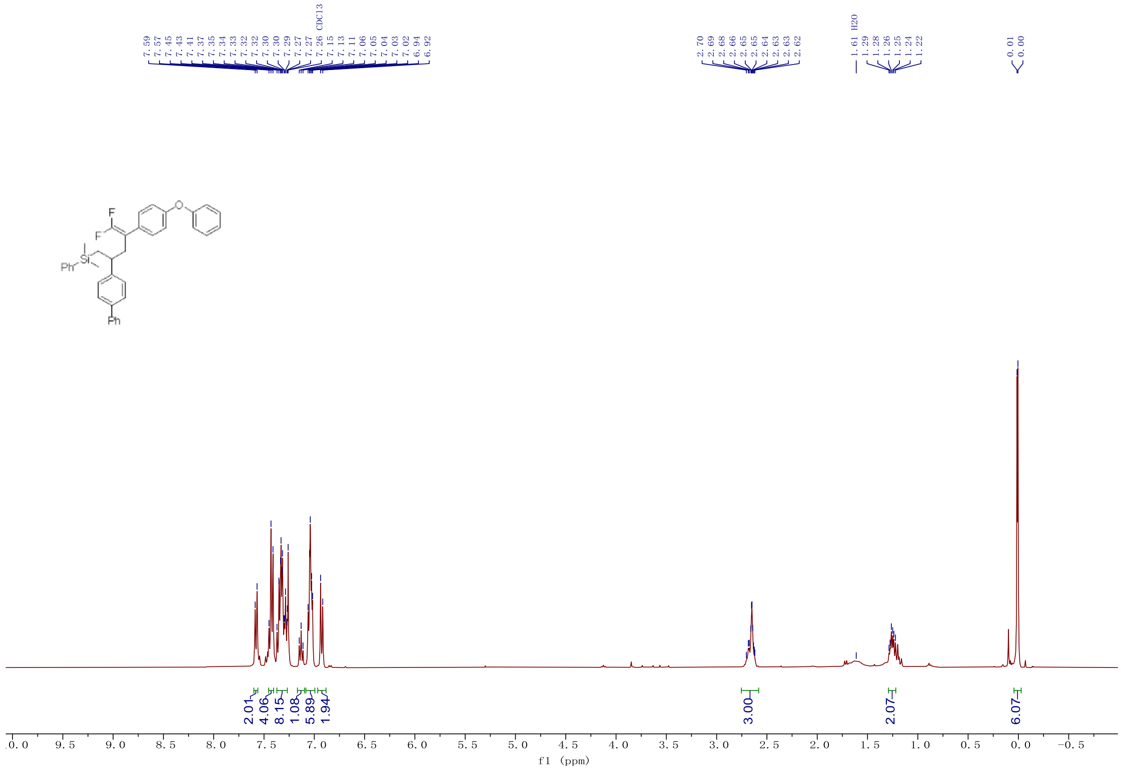

^{13}C NMR spectra of compound **d-54** in $CDCl_3$ (101 MHz): (see procedure)

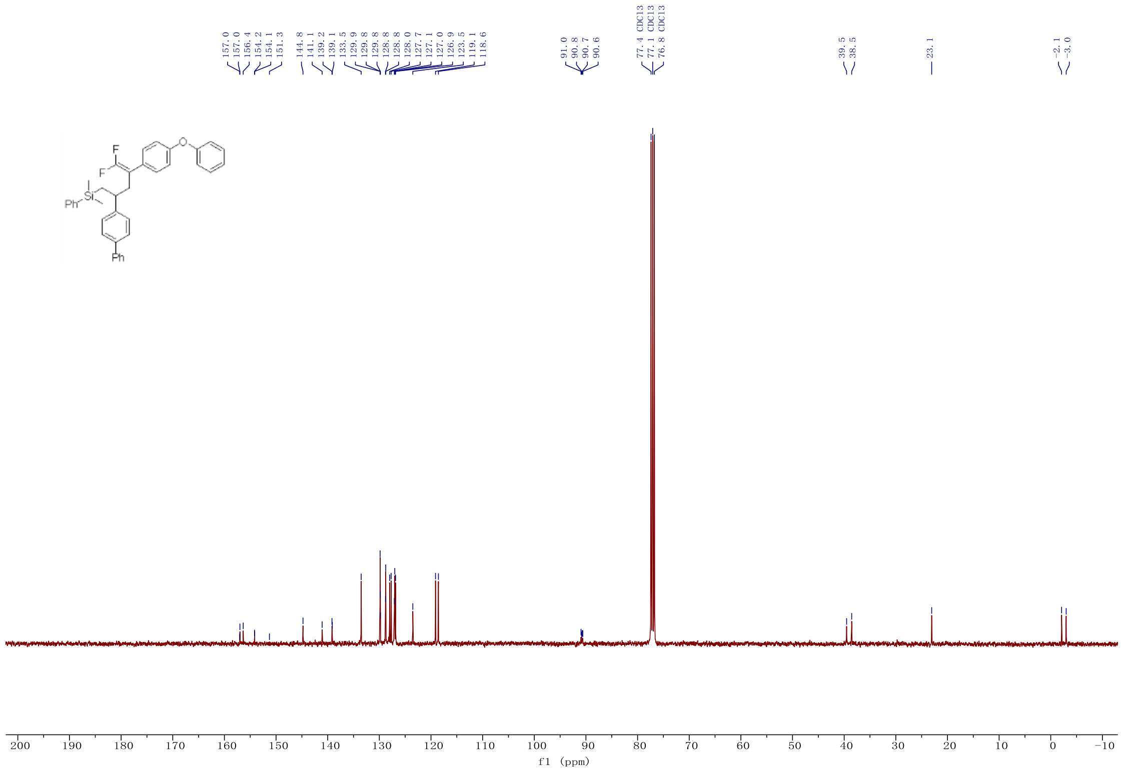

^{19}F NMR spectra of compound **d-54** in $CDCl_3$ (376 MHz): (*see procedure*)

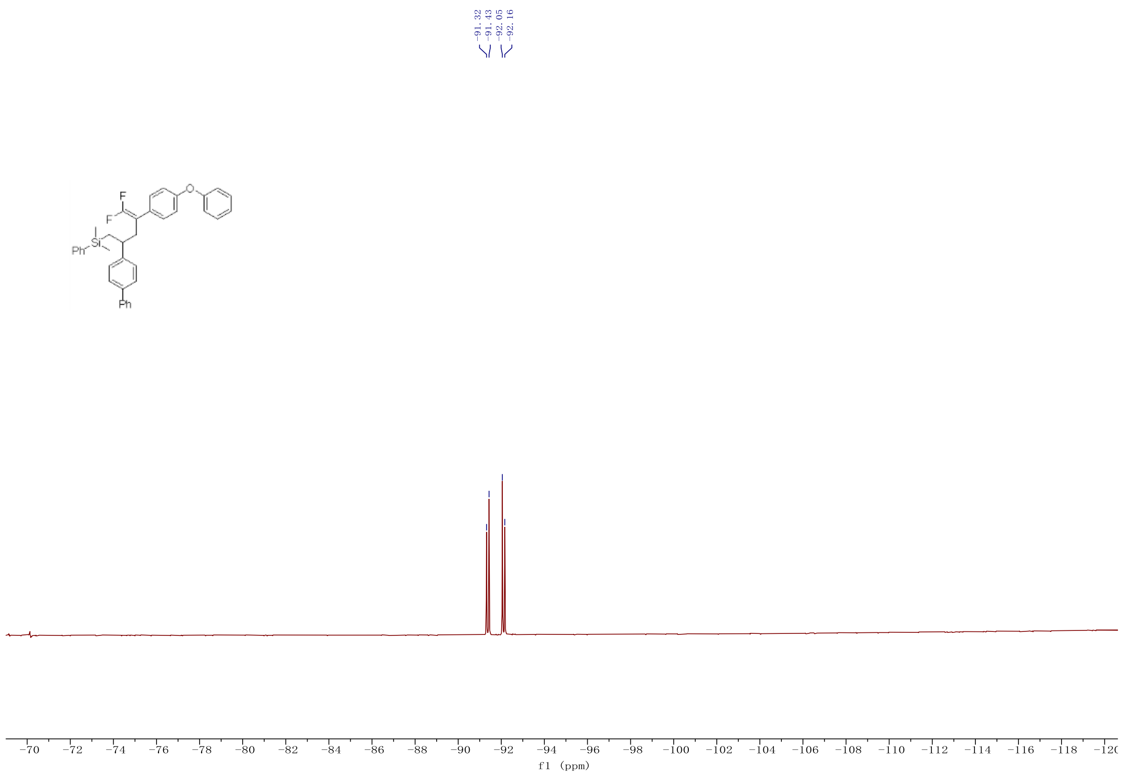

^{1}H NMR spectra of compound **d-55** in $CDCl_3$ (400 MHz): (*see procedure*)

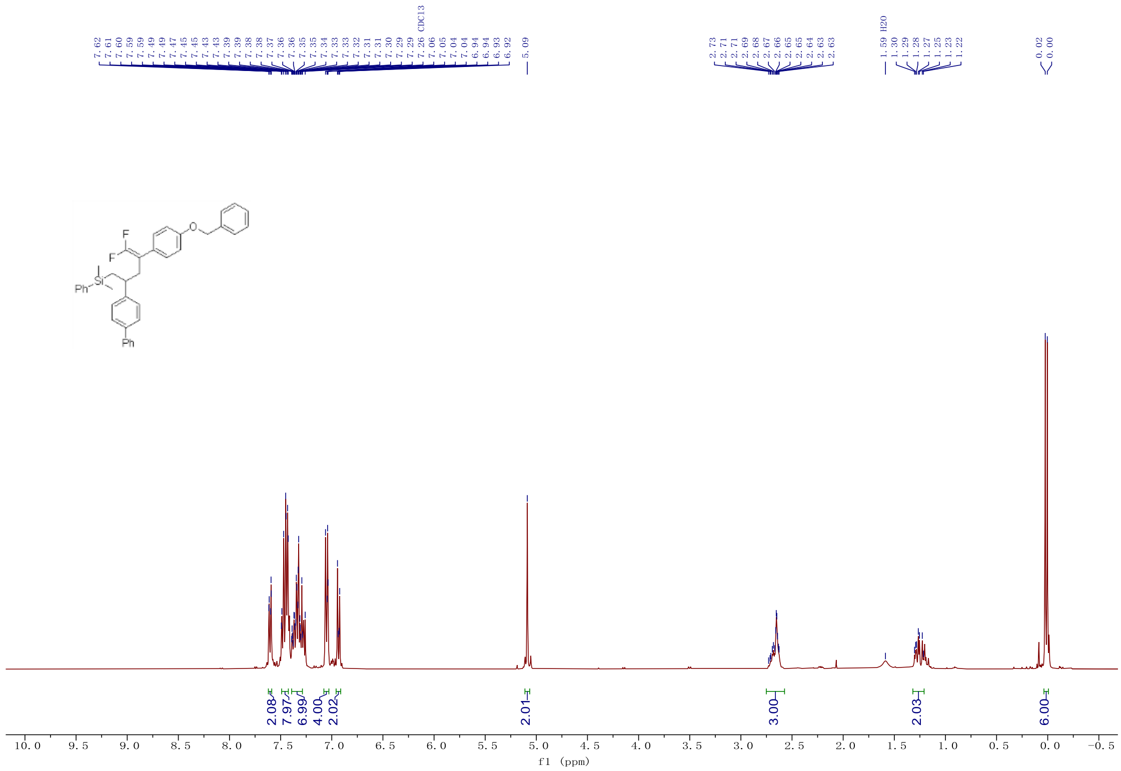

^{13}C NMR spectra of compound **d-55** in $CDCl_3$ (101 MHz): (*see procedure*)

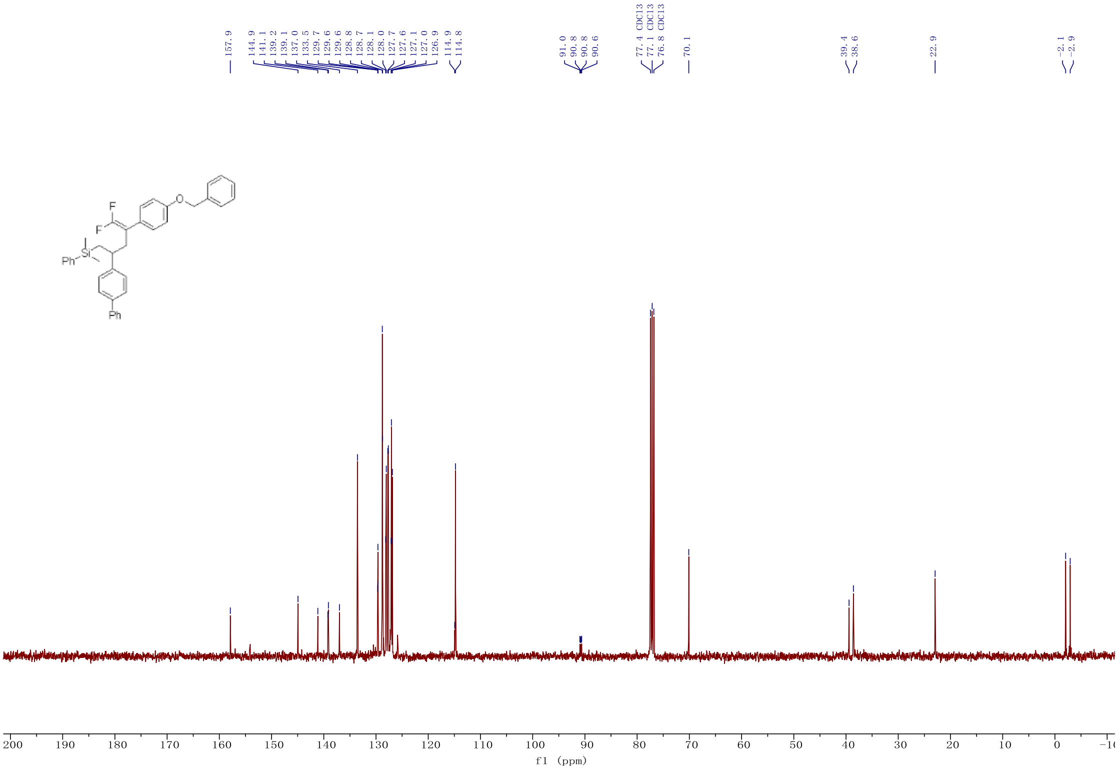

^{19}F NMR spectra of compound **d-55** in $CDCl_3$ (376 MHz): (*see procedure*)

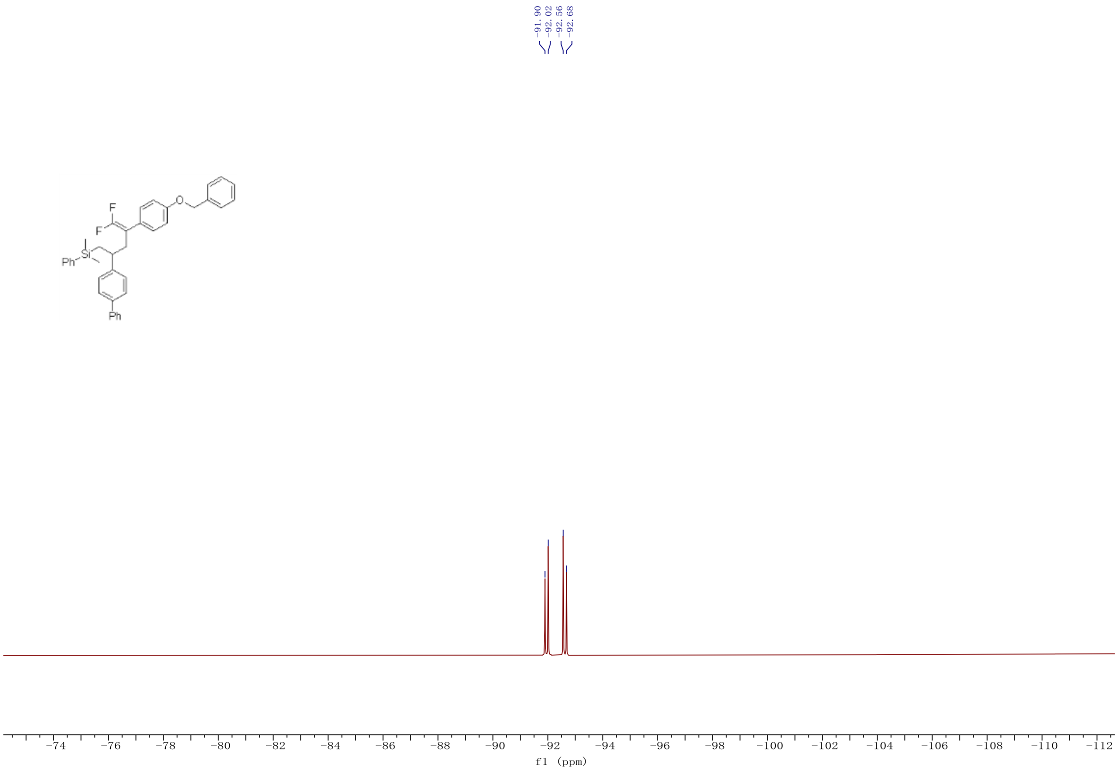

^{1}H NMR spectra of compound **d-56** in $CDCl_3$ (400 MHz): (*see procedure*)

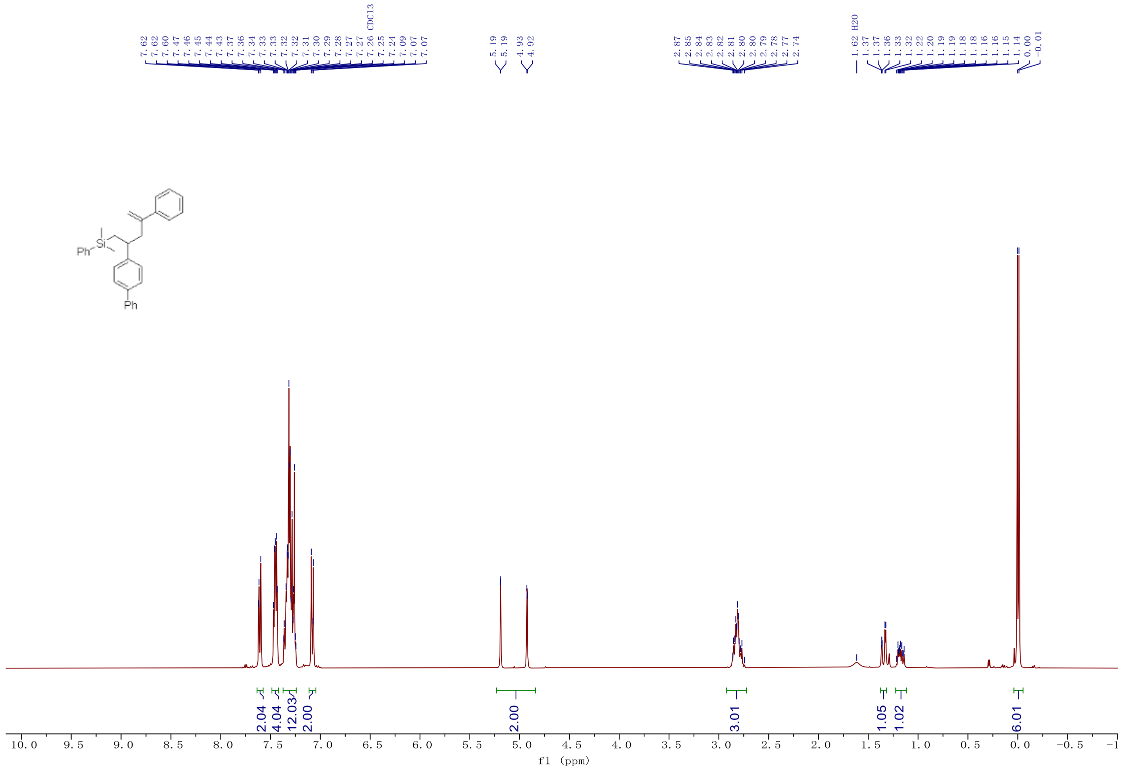

^{13}C NMR spectra of compound **d-56** in $CDCl_3$ (101 MHz): (*see procedure*)

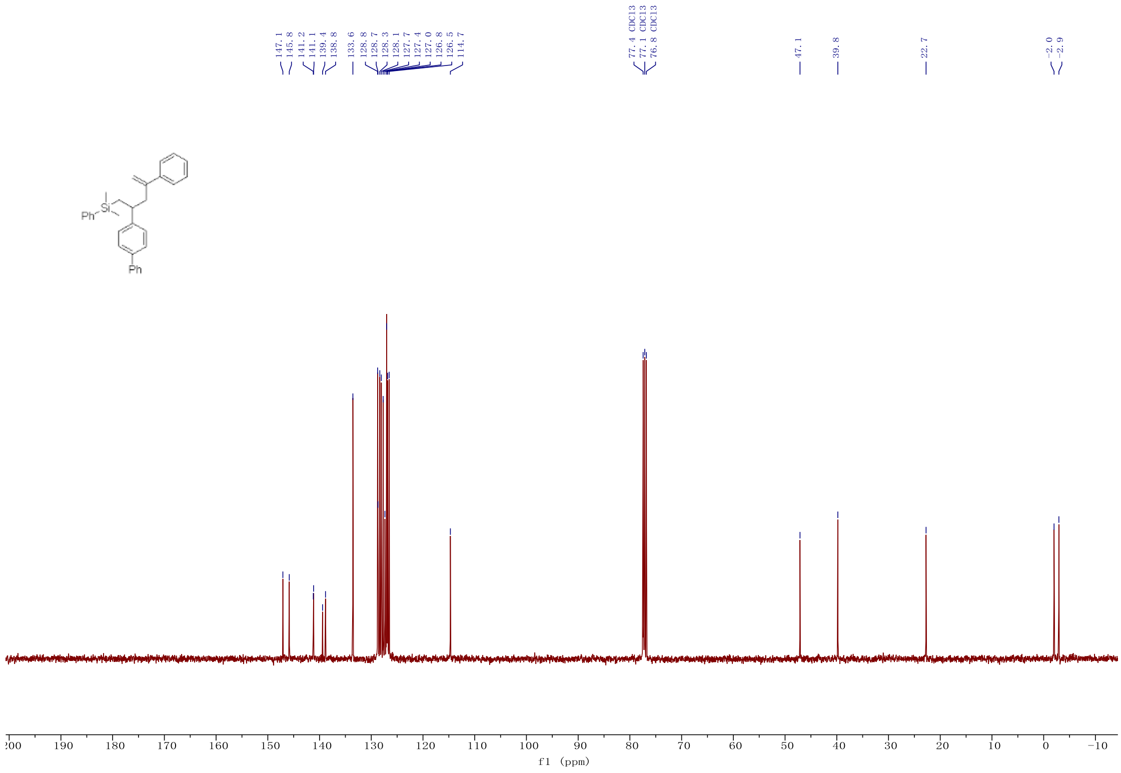

^{1}H NMR spectra of compound **d-57** in $CDCl_3$ (400 MHz): (*see procedure*)

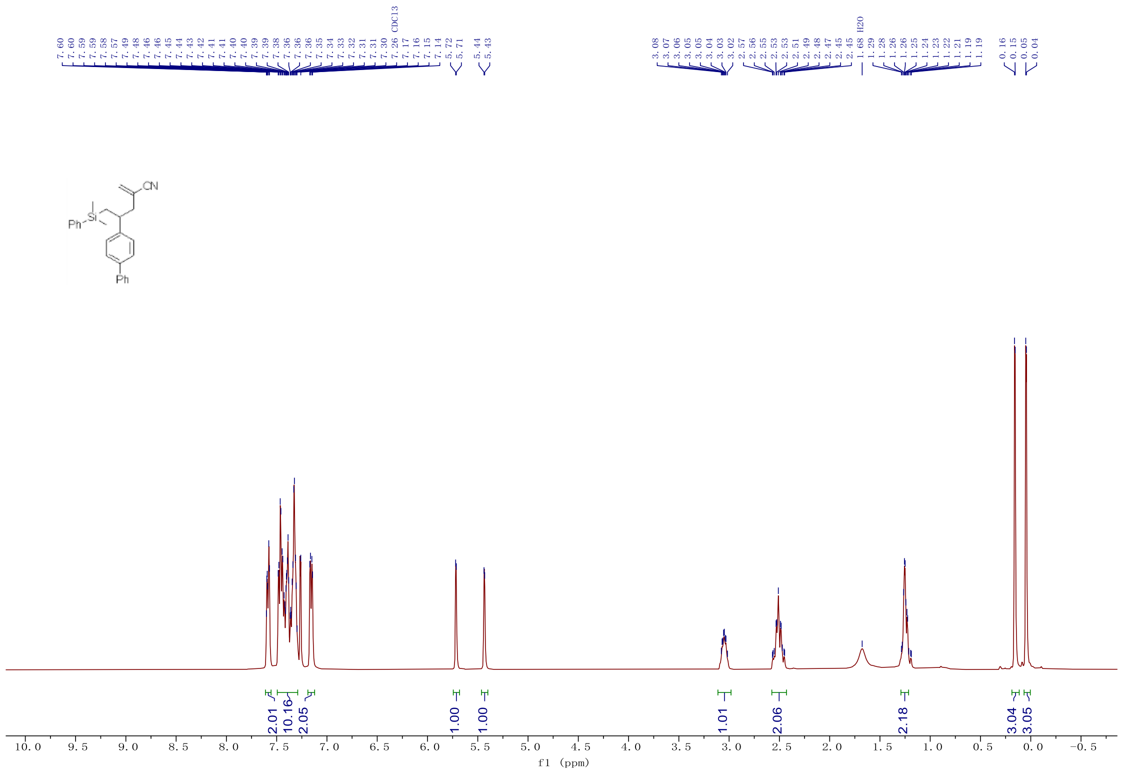

^{13}C NMR spectra of compound **d-57** in $CDCl_3$ (101 MHz): (*see procedure*)

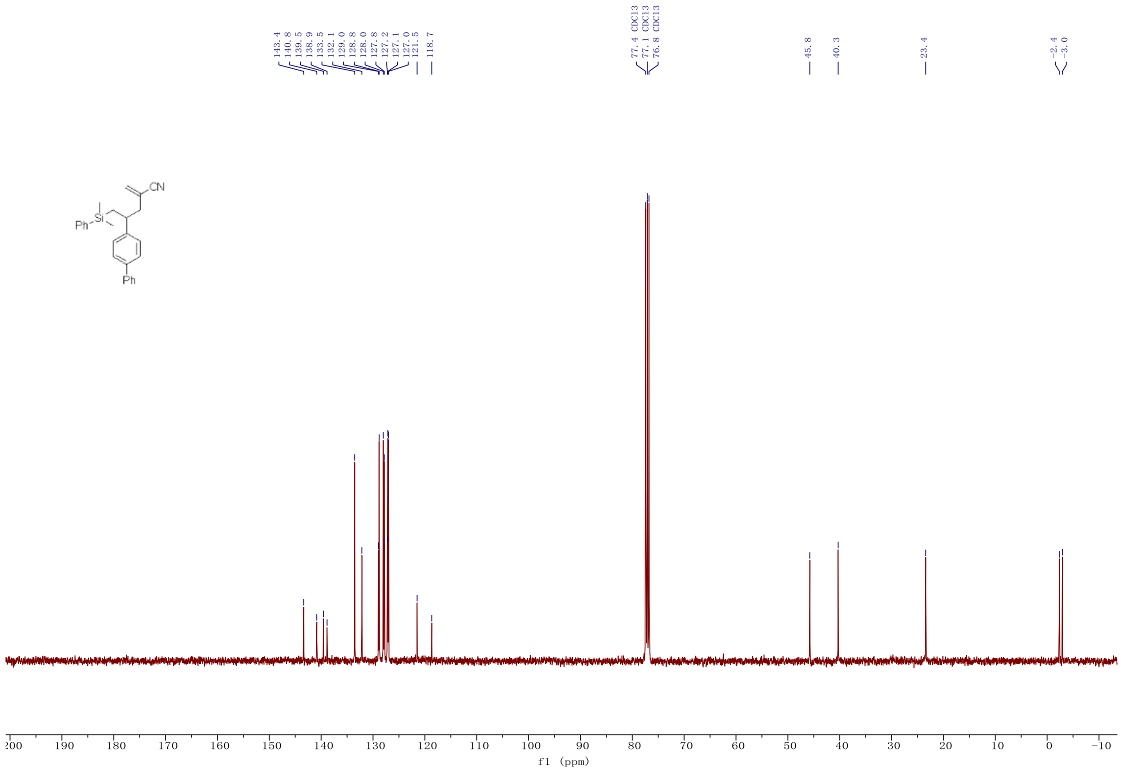

^{1}H NMR spectra of compound **d-58** in $CDCl_3$ (400 MHz): (see procedure)

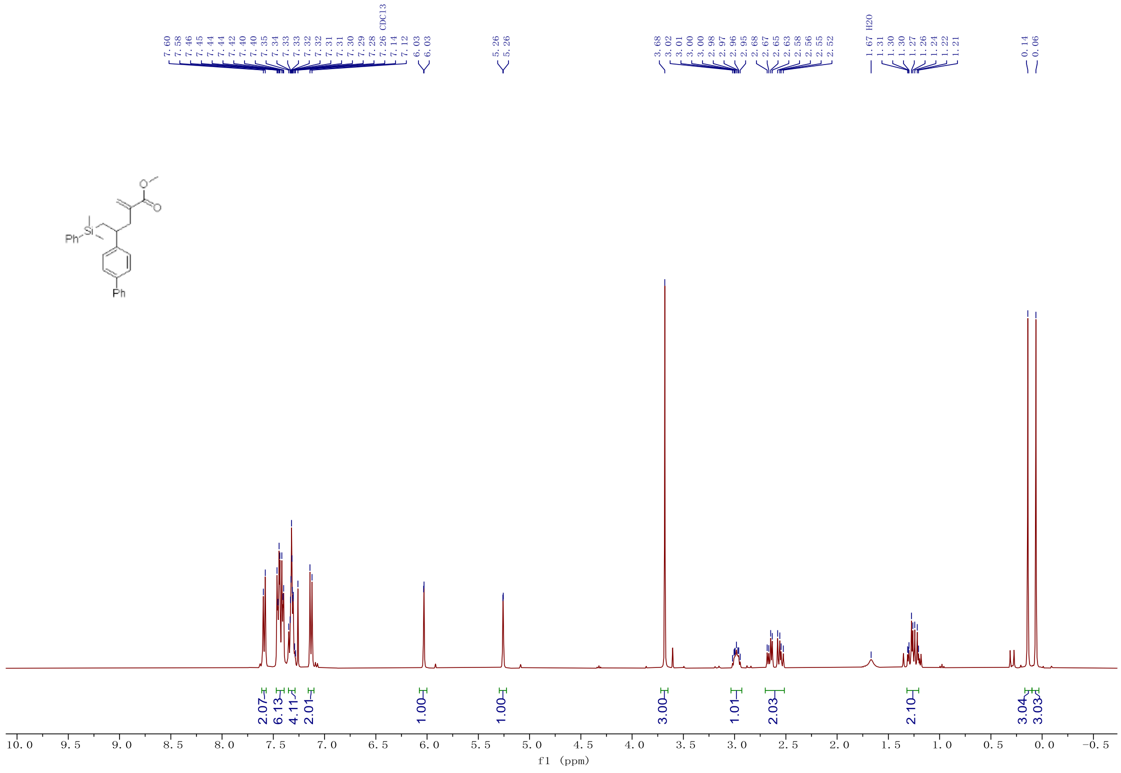

^{13}C NMR spectra of compound **d-58** in $CDCl_3$ (101 MHz): (see procedure)

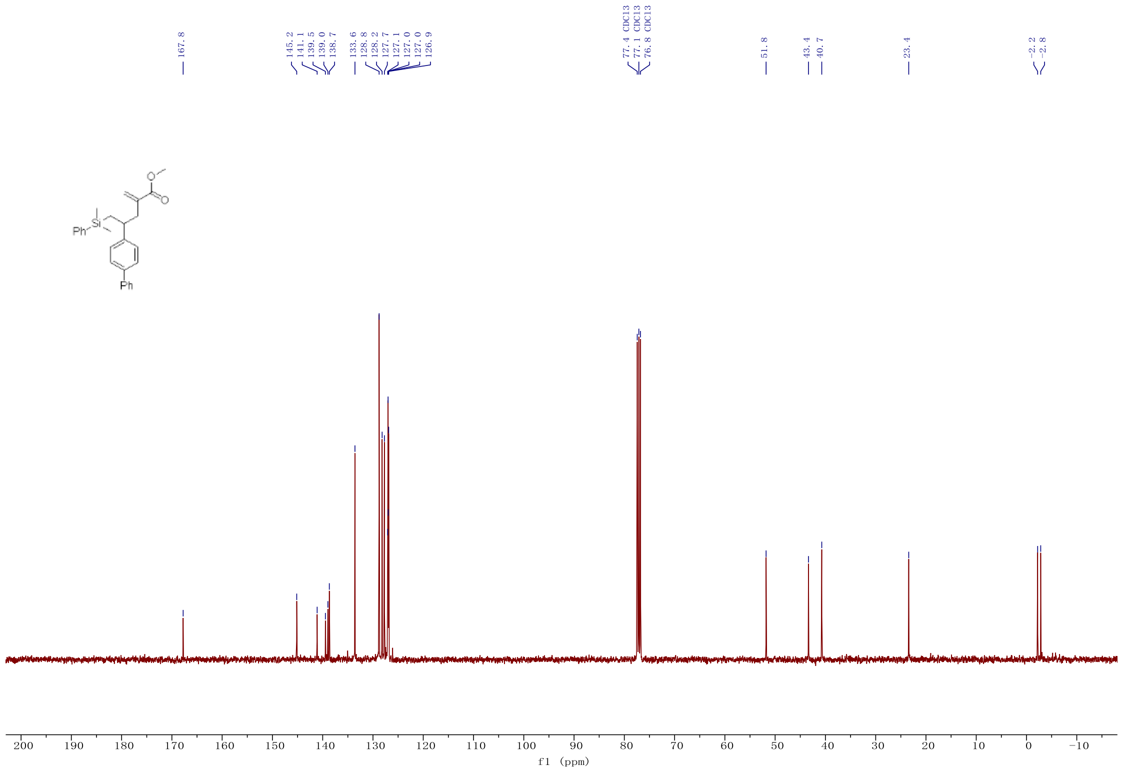

^{1}H NMR spectra of compound **d-59** in $CDCl_3$ (400 MHz): (see procedure)

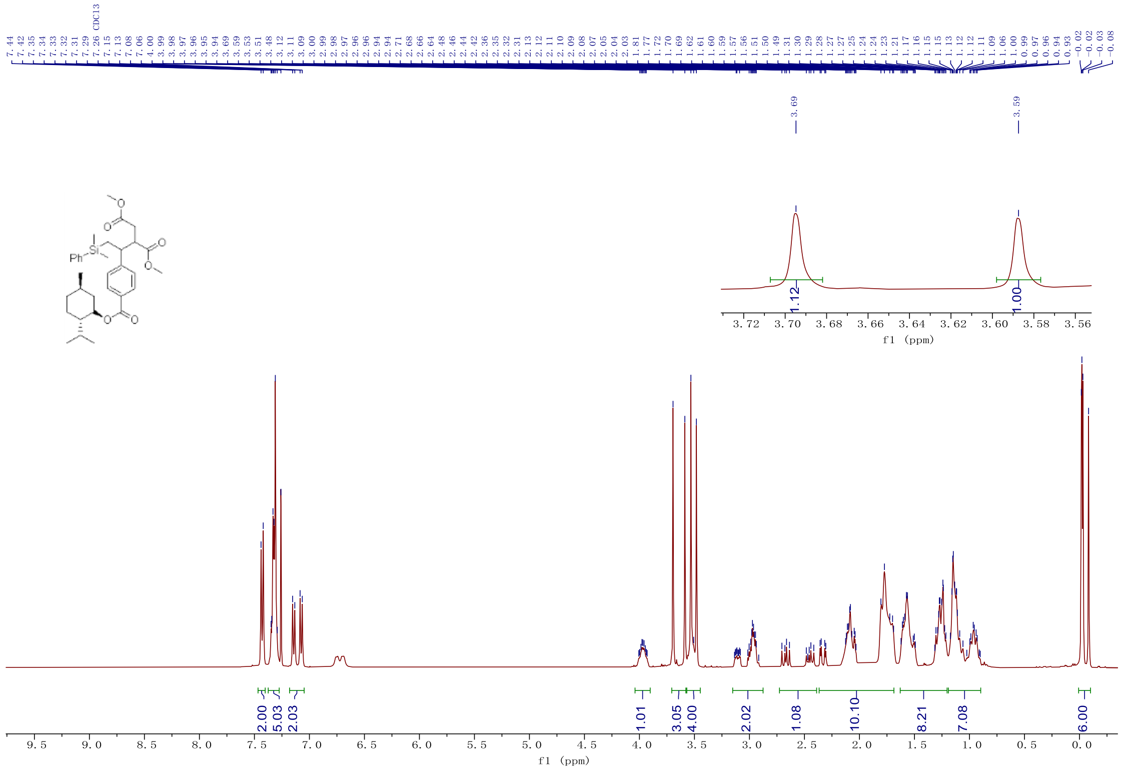

^{13}C NMR spectra of compound **d-59** in $CDCl_3$ (101 MHz): (see procedure)

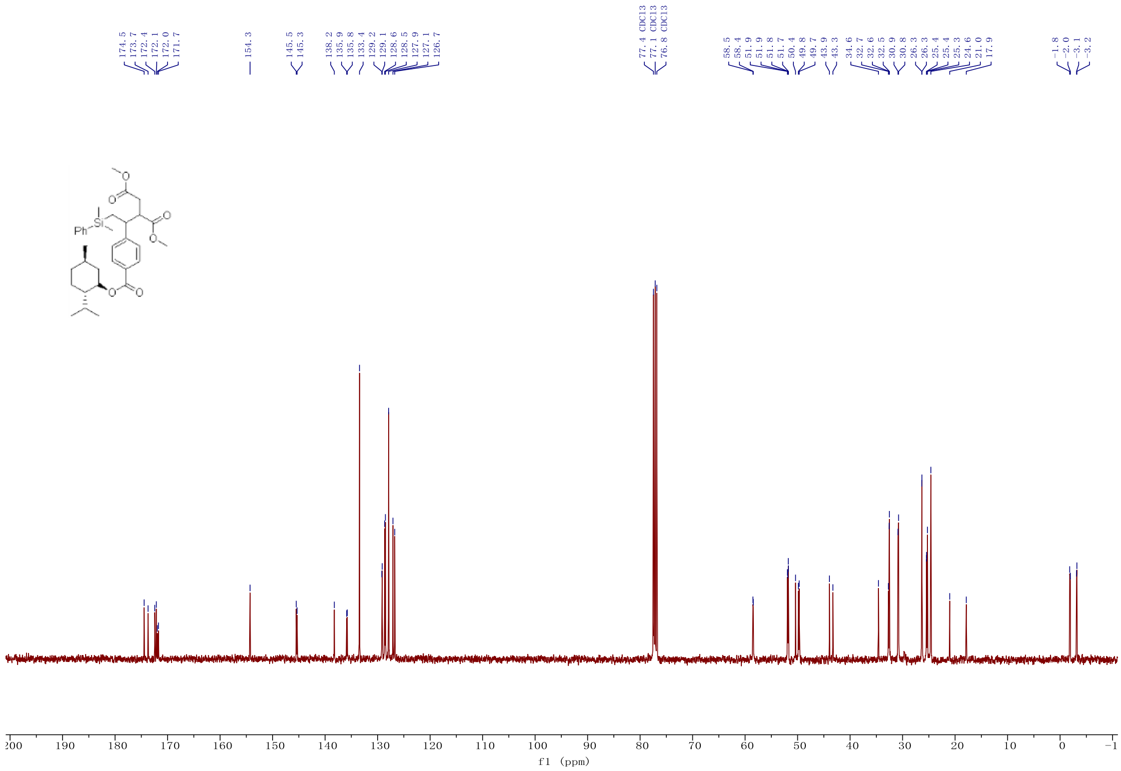

^{1}H NMR spectra of compound **d-60** in $CDCl_3$ (400 MHz): (*see procedure*)

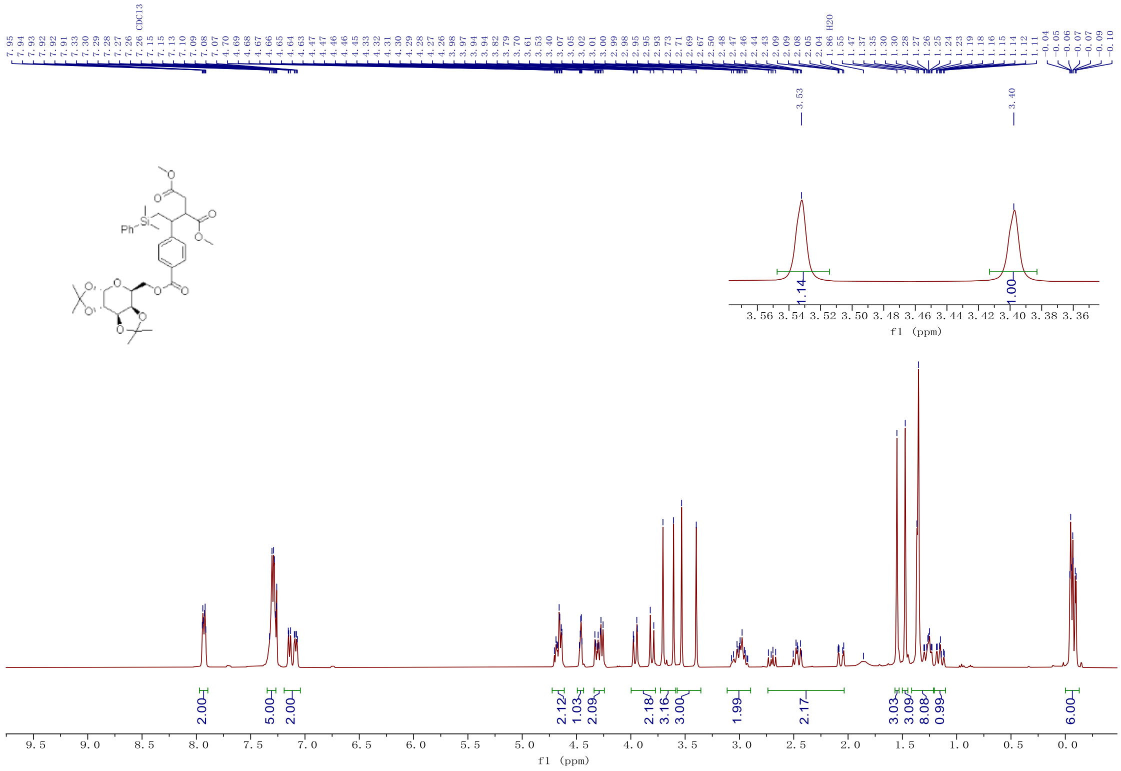

^{13}C NMR spectra of compound **d-60** in $CDCl_3$ (101 MHz): (*see procedure*)

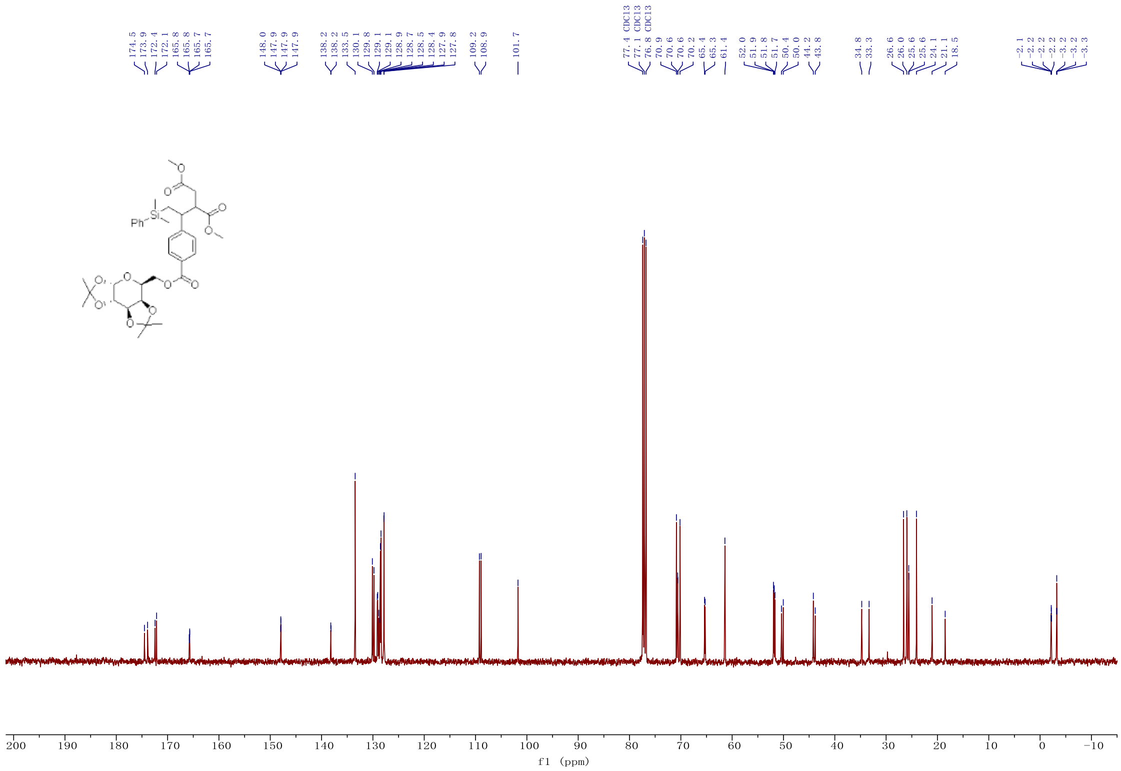

^{1}H NMR spectra of compound **d-61** in $CDCl_3$ (400 MHz): (see procedure)

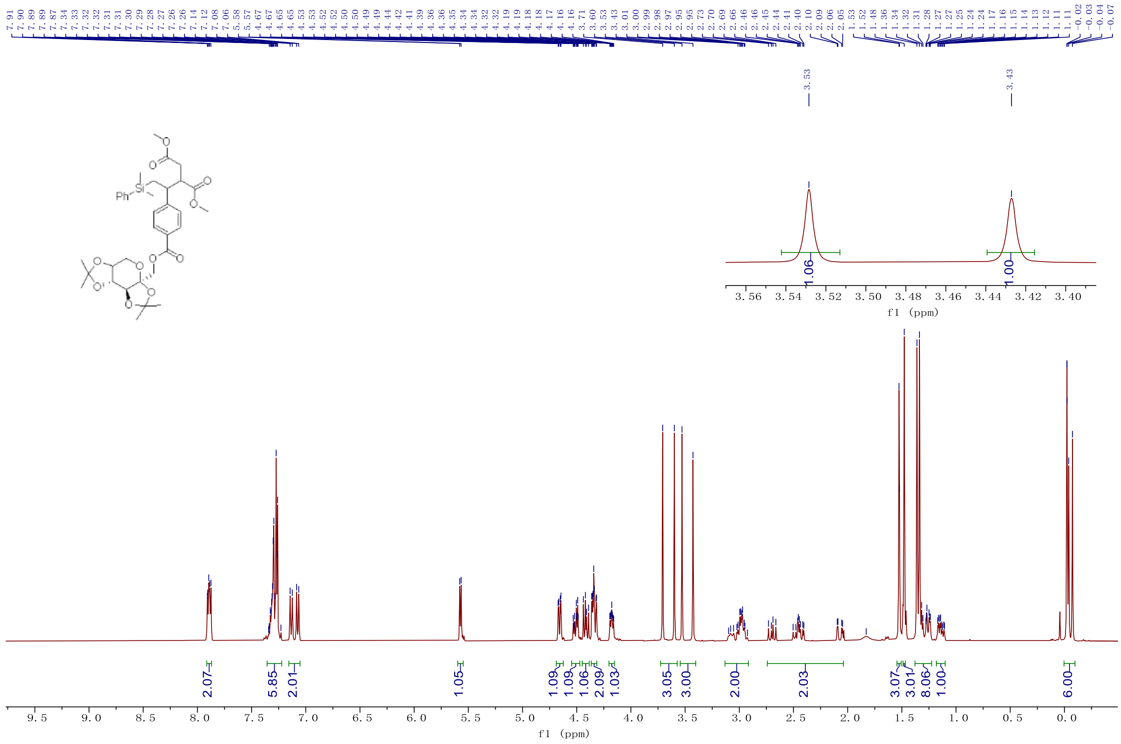

^{13}C NMR spectra of compound **d-61** in $CDCl_3$ (101 MHz): (see procedure)

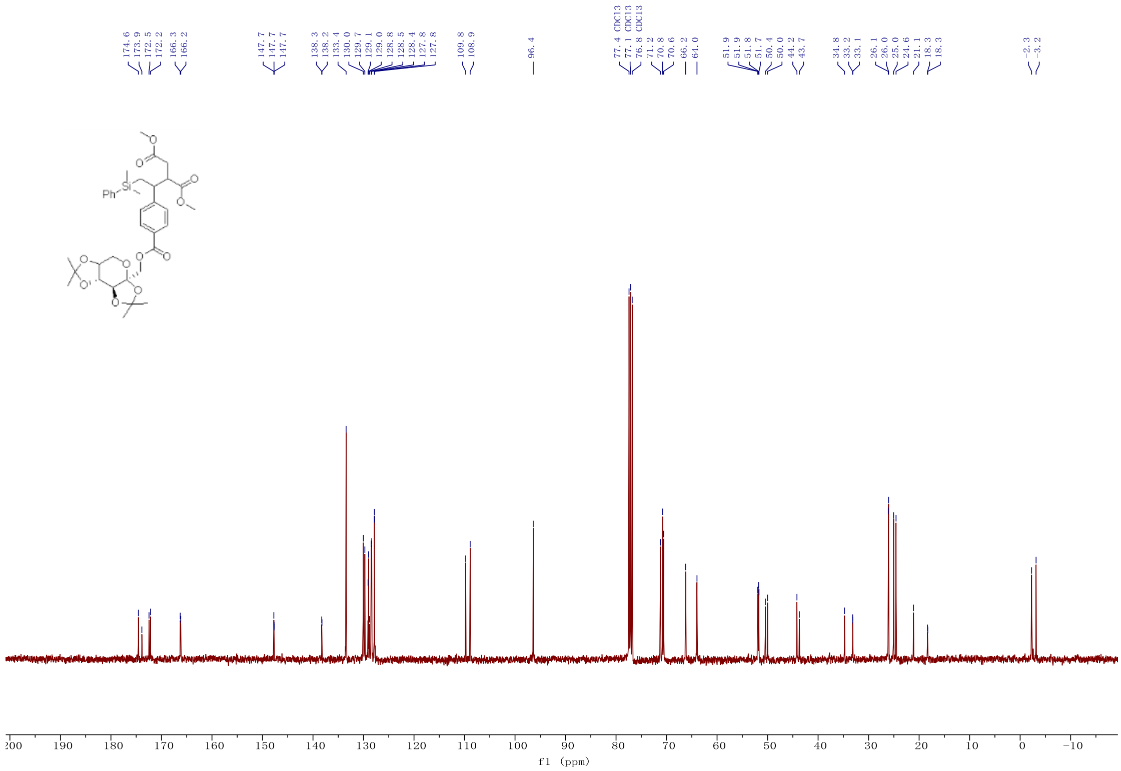

^{1}H NMR spectra of compound **d-62** in $CDCl_3$ (400 MHz): (*see procedure*)

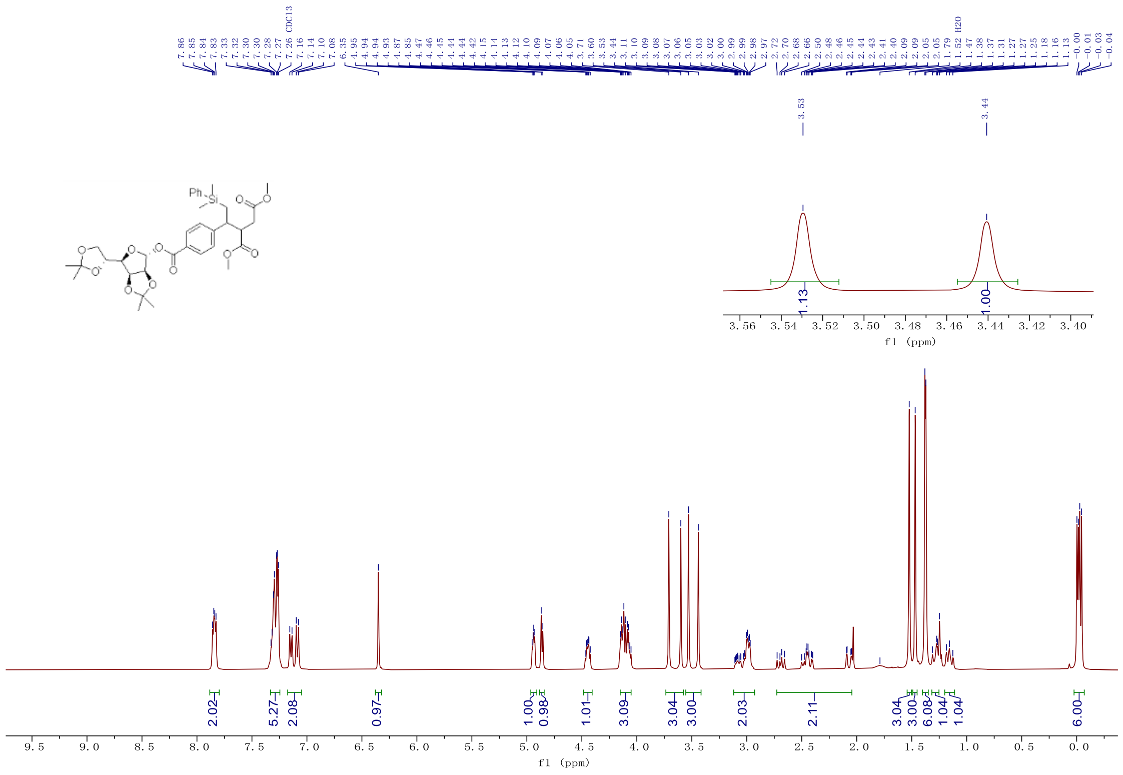

^{13}C NMR spectra of compound **d-62** in $CDCl_3$ (101 MHz): (*see procedure*)

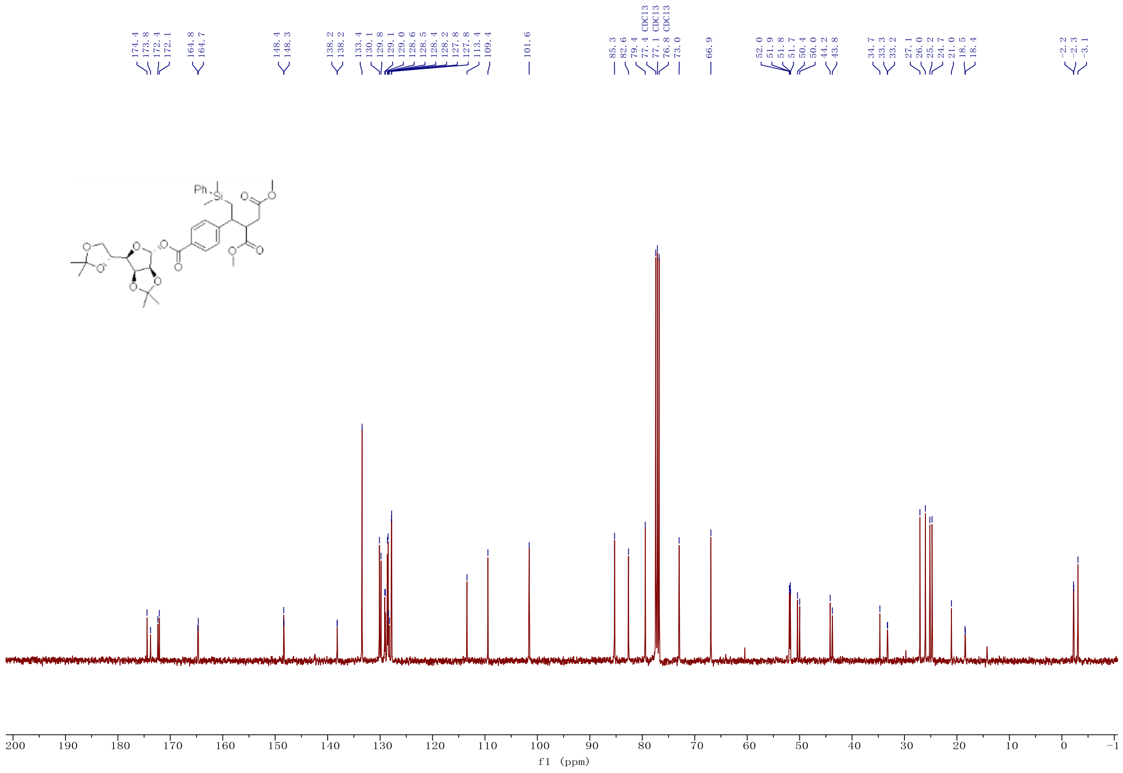

^{1}H NMR spectra of compound **d-63** in $CDCl_3$ (400 MHz): (<u>*see procedure*</u>)

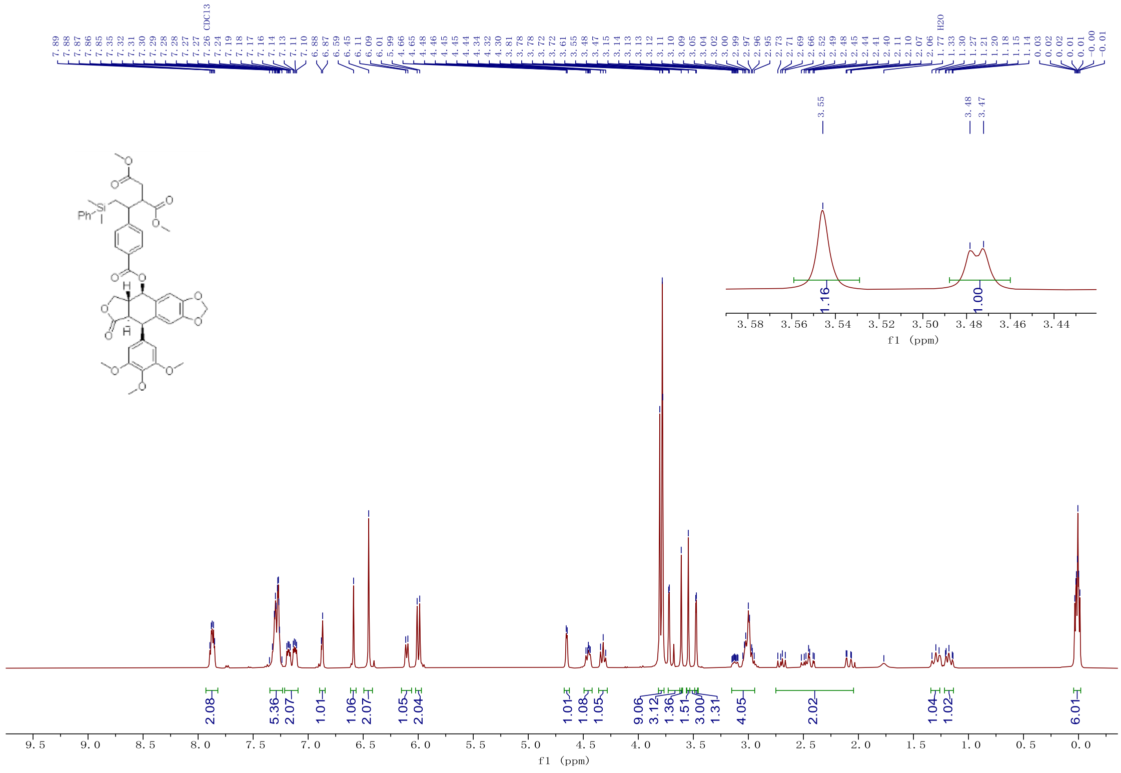

^{13}C NMR spectra of compound **d-63** in $CDCl_3$ (101 MHz): (<u>*see procedure*</u>)

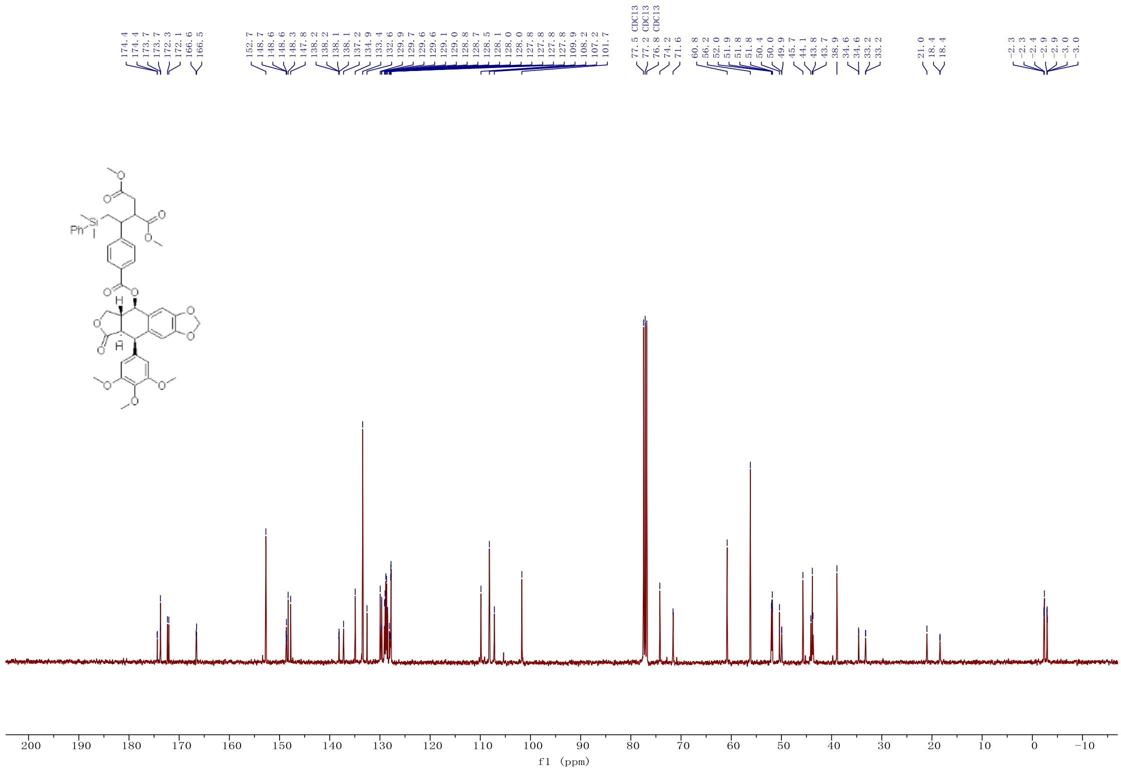

^{1}H NMR spectra of compound **d-64** in $CDCl_3$ (400 MHz): (see procedure)

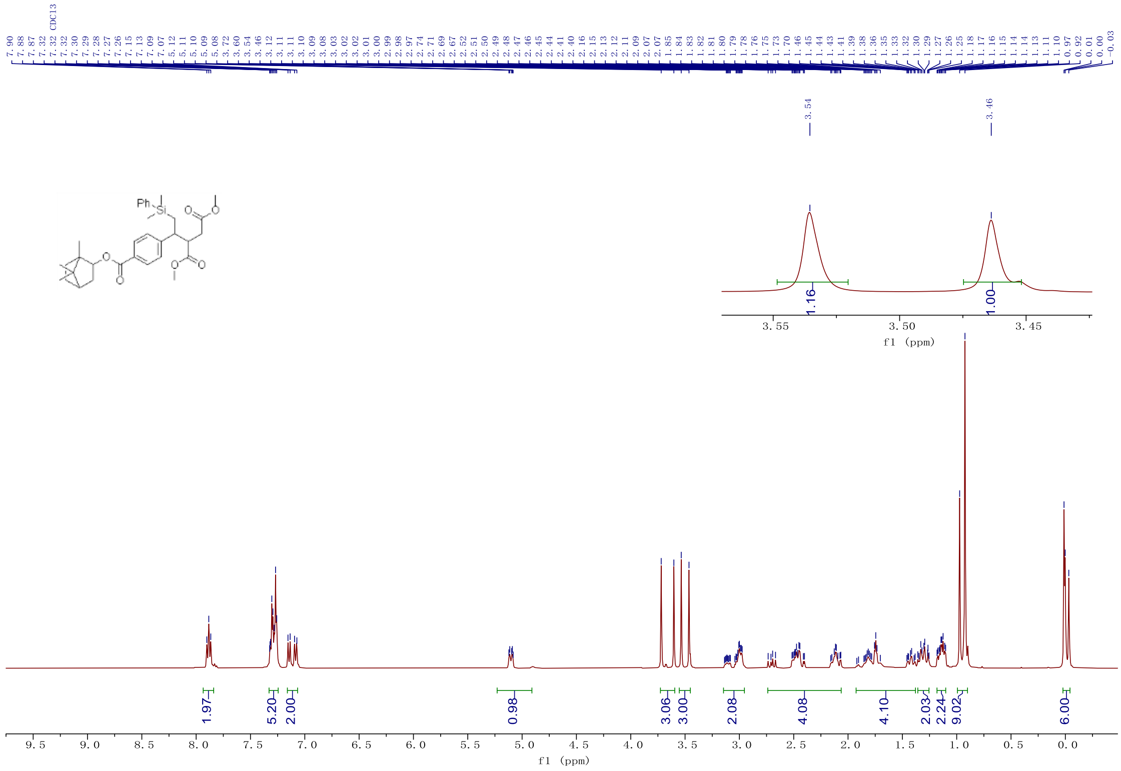

^{13}C NMR spectra of compound **d-64** in $CDCl_3$ (101 MHz): (see procedure)

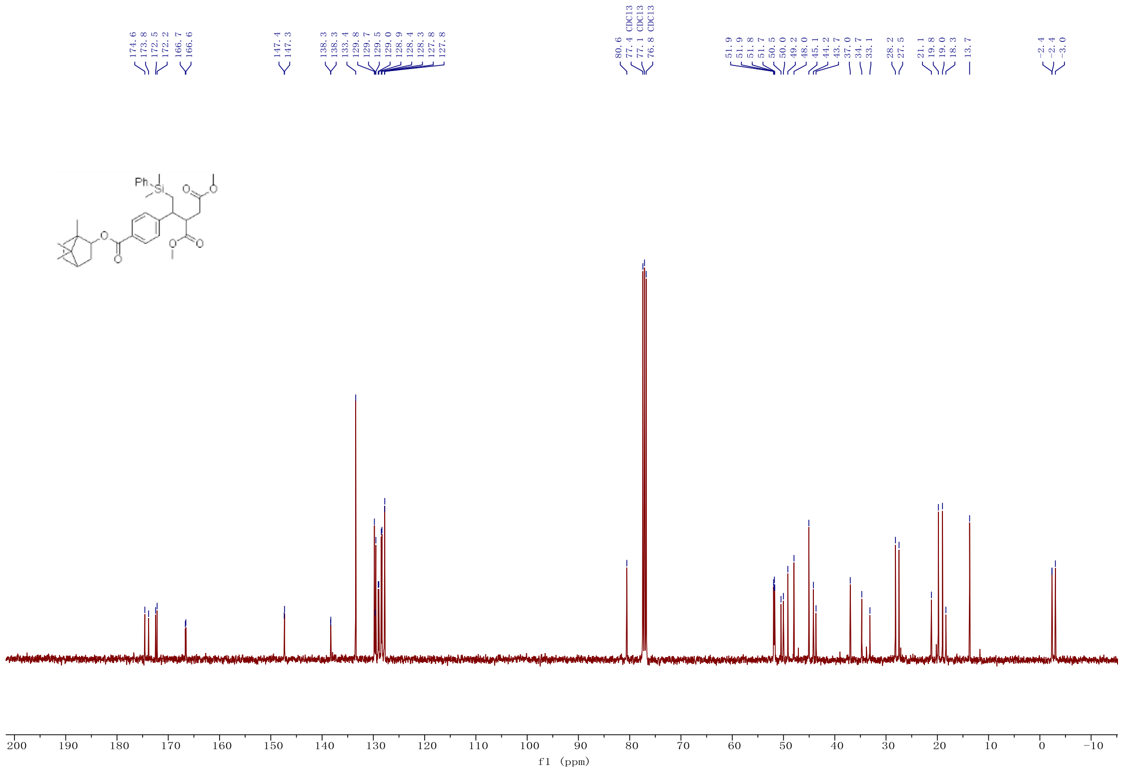

^{1}H NMR spectra of compound **d-65** in $CDCl_3$ (400 MHz): (*see procedure*)

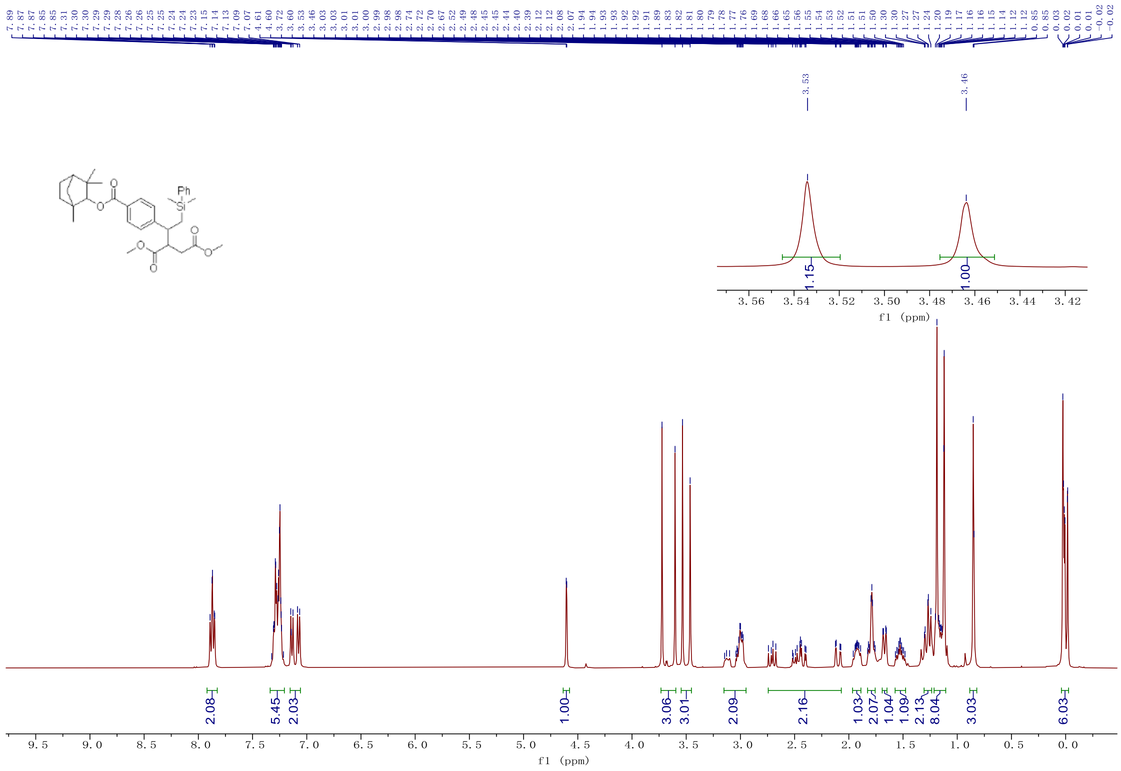

^{13}C NMR spectra of compound **d-65** in $CDCl_3$ (101 MHz): (*see procedure*)

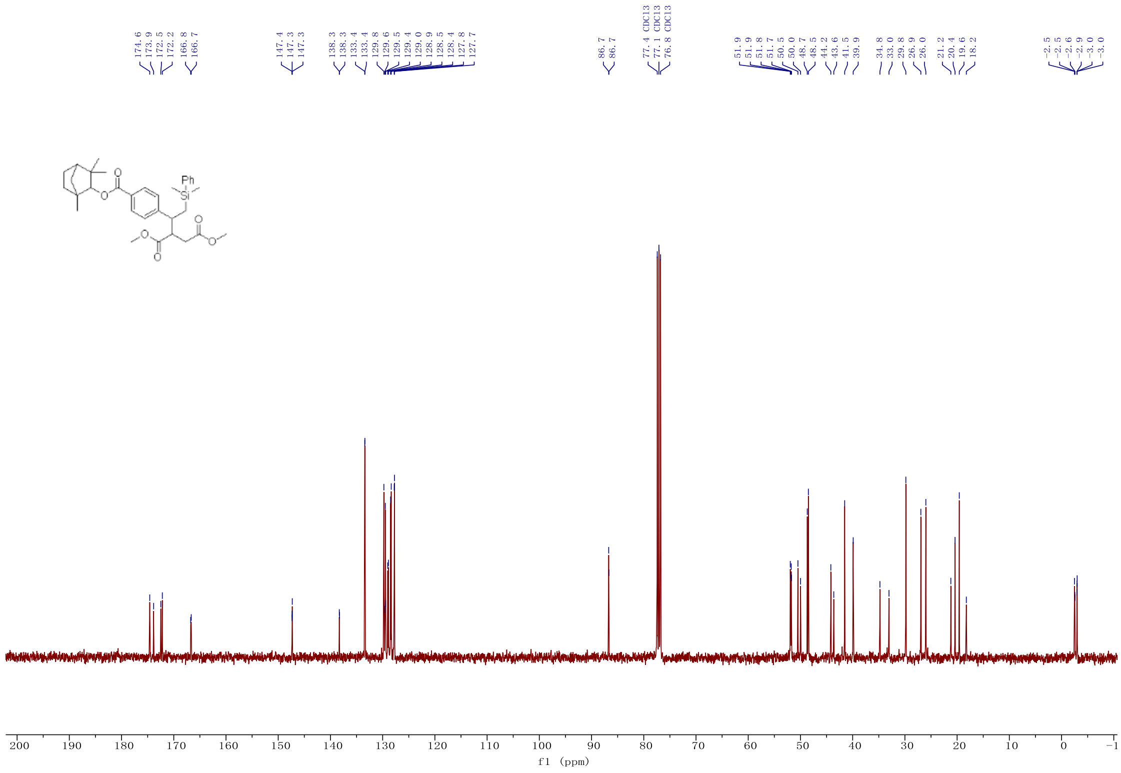

^{1}H NMR spectra of compound **d-66** in $CDCl_3$ (400 MHz): (*see procedure*)

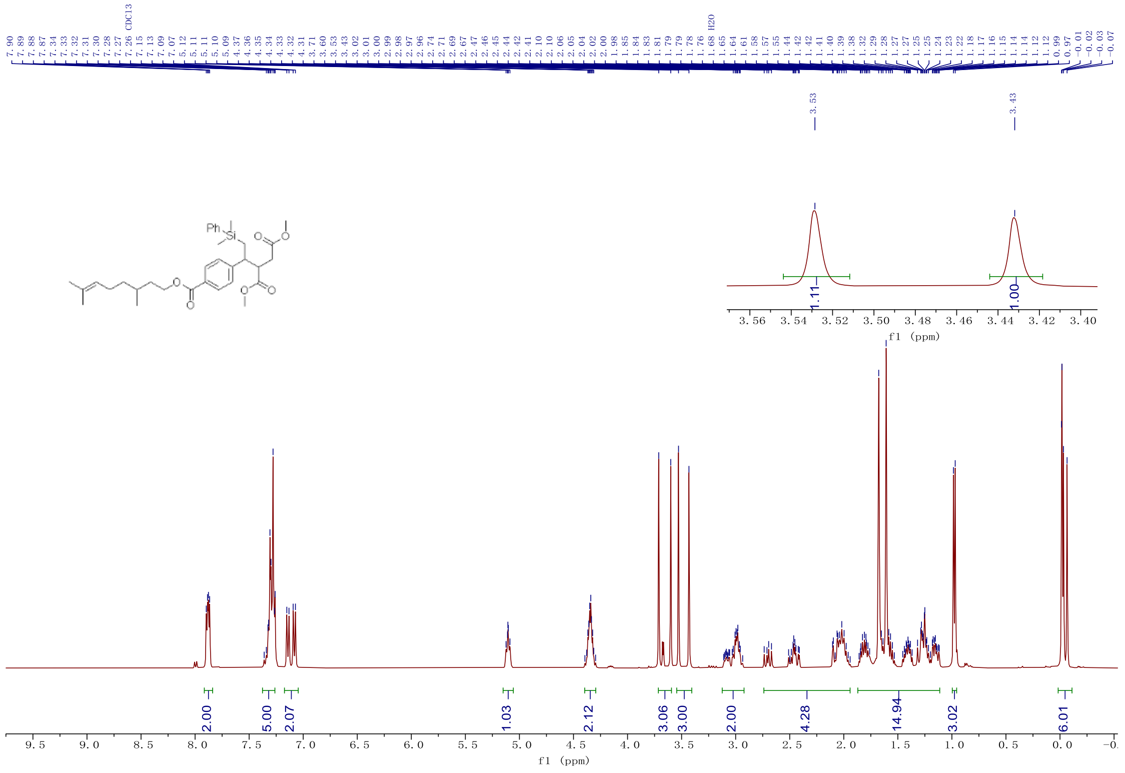

^{13}C NMR spectra of compound **d-66** in $CDCl_3$ (101 MHz): (*see procedure*)

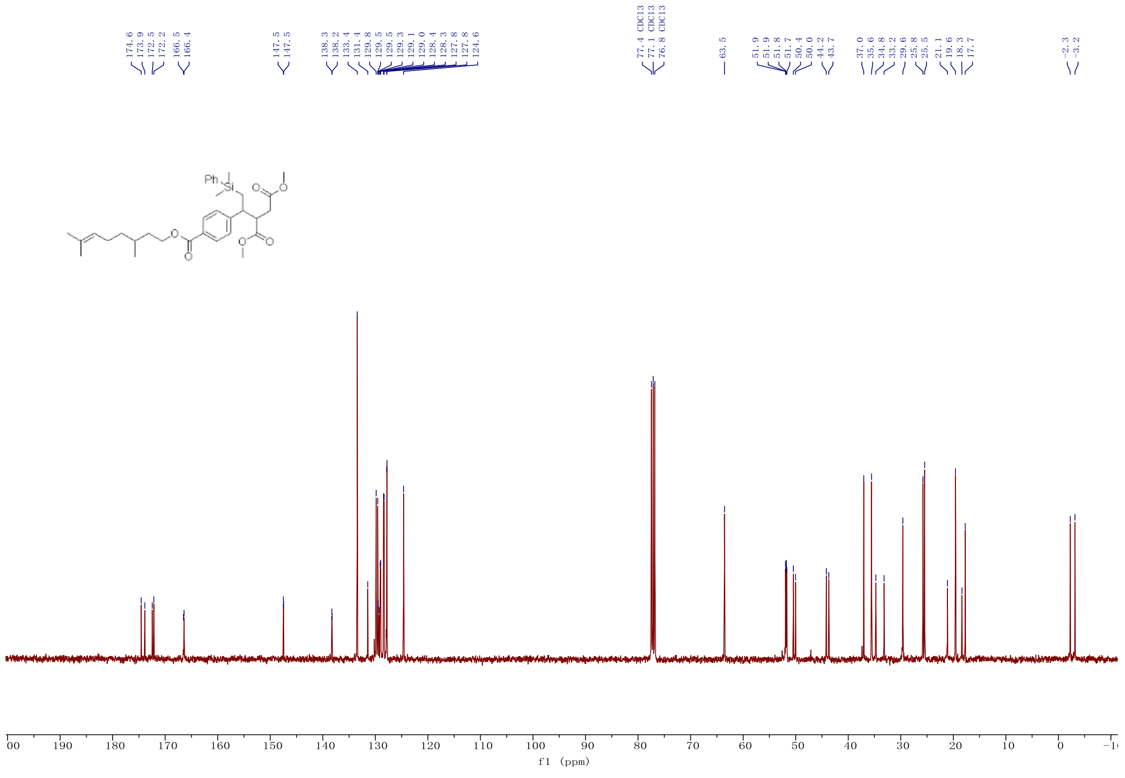

^{1}H NMR spectra of compound **d-67** in $CDCl_3$ (400 MHz): (*see procedure*)

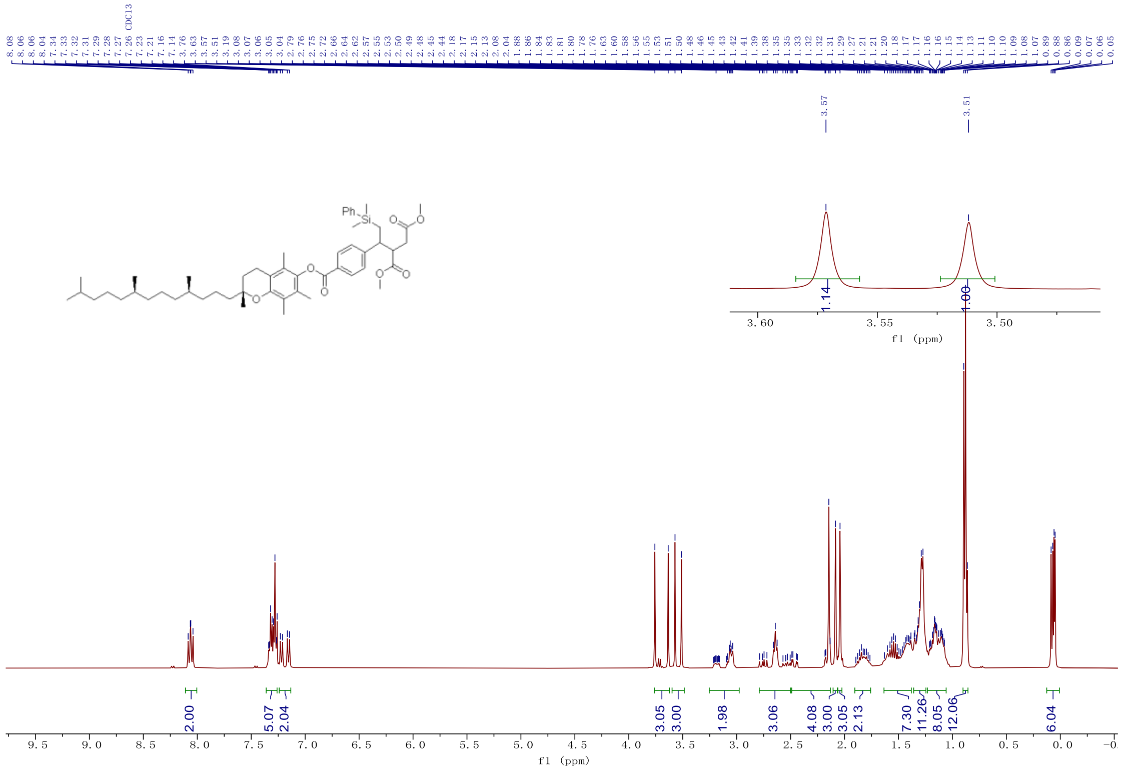

^{13}C NMR spectra of compound **d-67** in $CDCl_3$ (101 MHz): (*see procedure*)

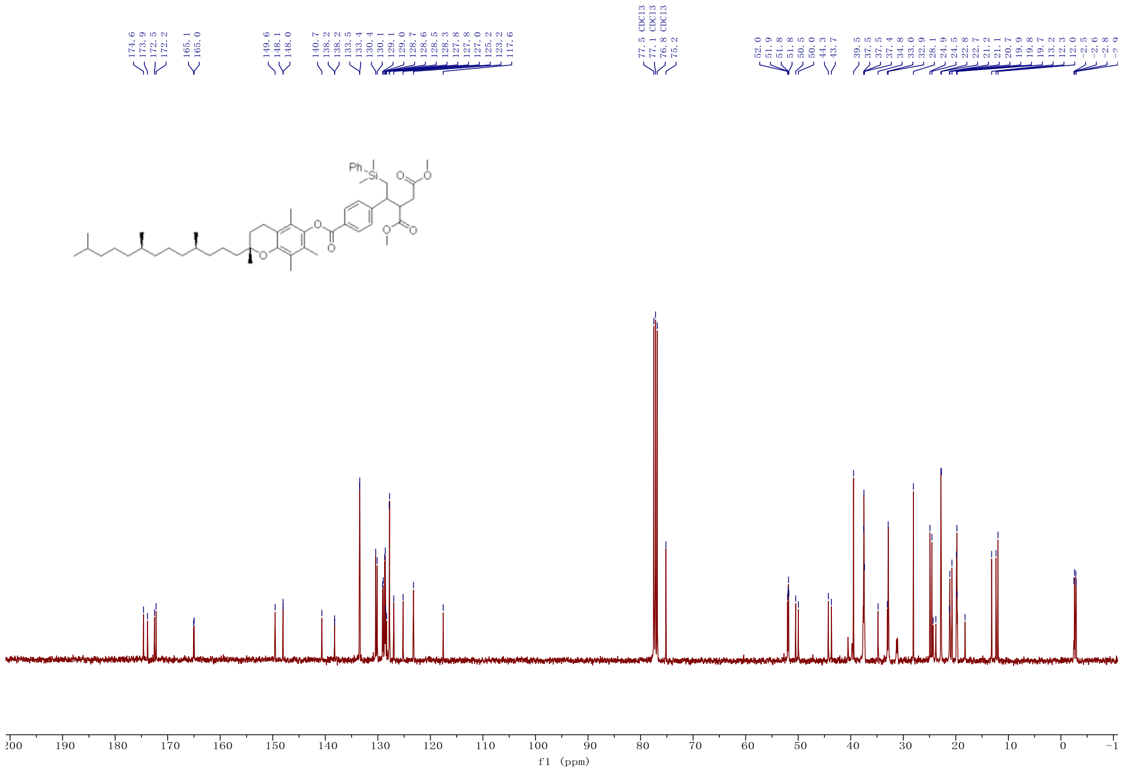

^{1}H NMR spectra of compound **d-68** in $CDCl_3$ (400 MHz): (*see procedure*)

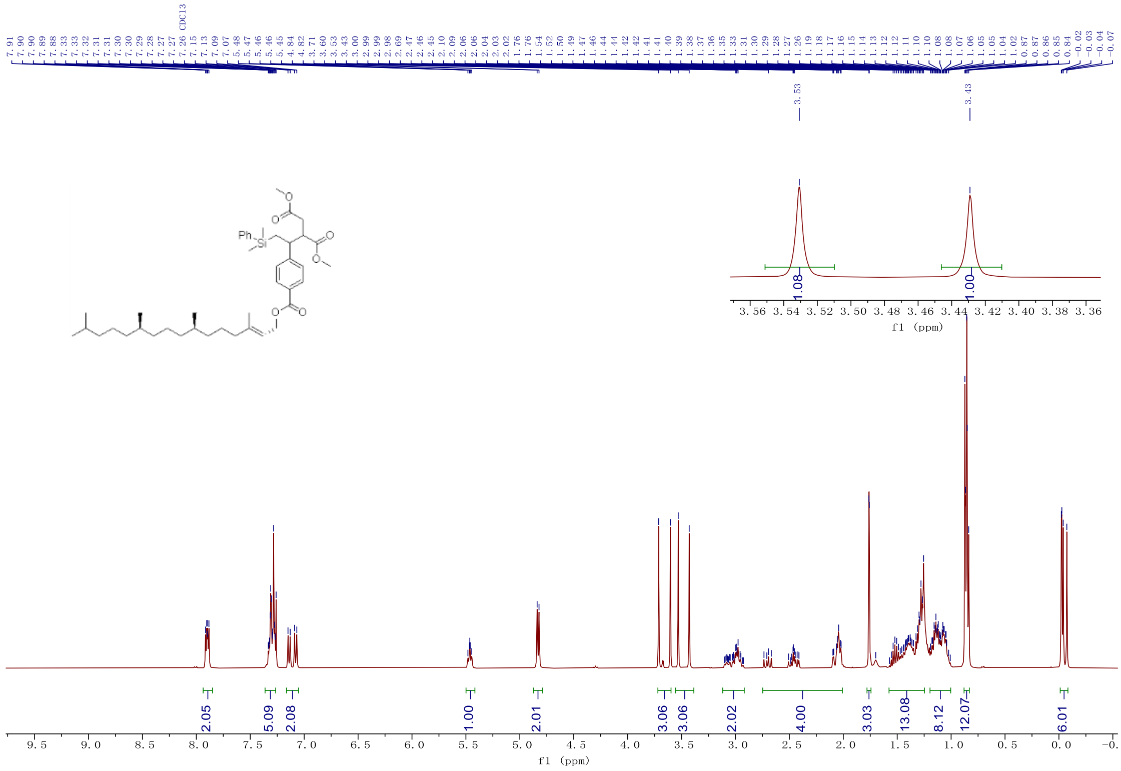

^{13}C NMR spectra of compound **d-68** in $CDCl_3$ (101 MHz): (*see procedure*)

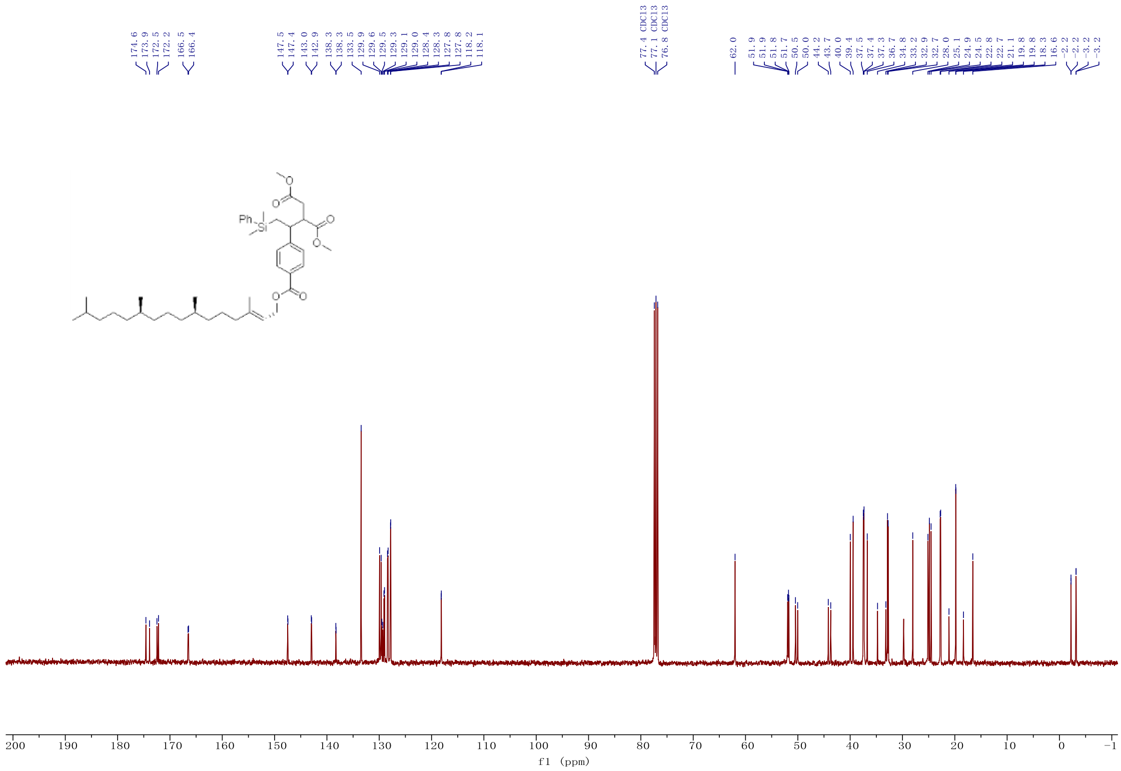

^{1}H NMR spectra of compound **d-69** in $CDCl_3$ (400 MHz): (*see procedure*)

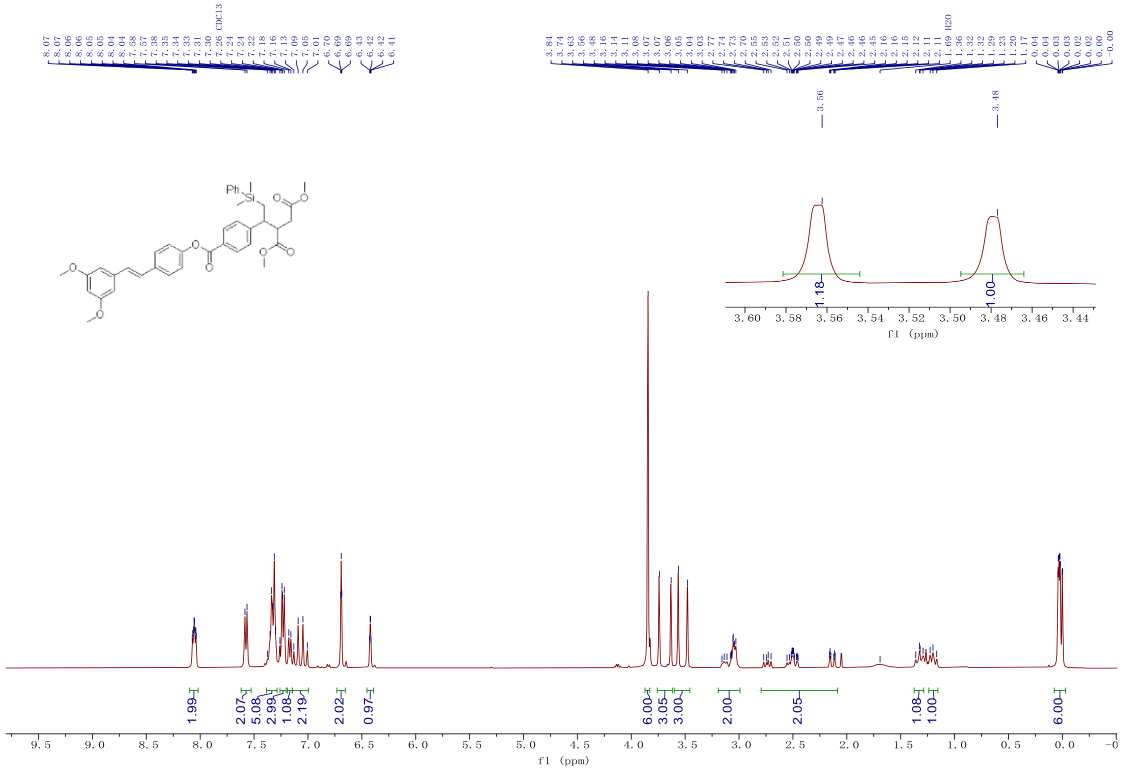

^{13}C NMR spectra of compound **d-69** in $CDCl_3$ (101 MHz): (*see procedure*)

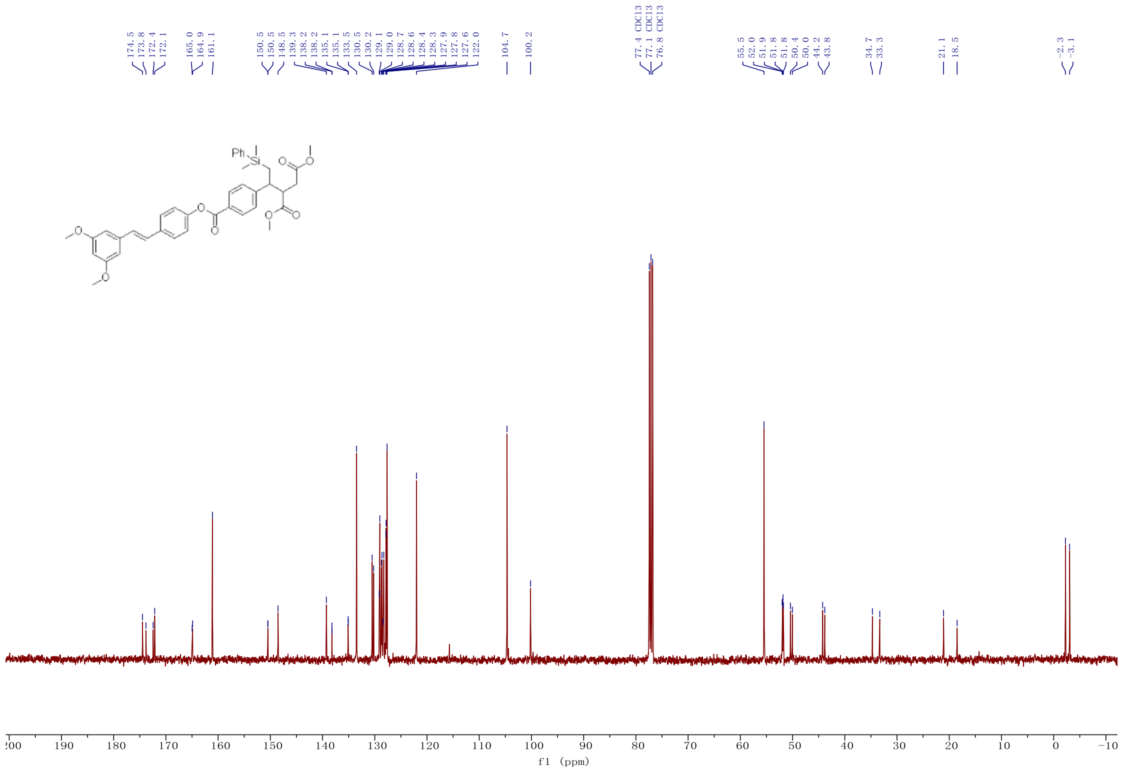

^{1}H NMR spectra of compound **d-70** in $CDCl_3$ (400 MHz): (*see procedure*)

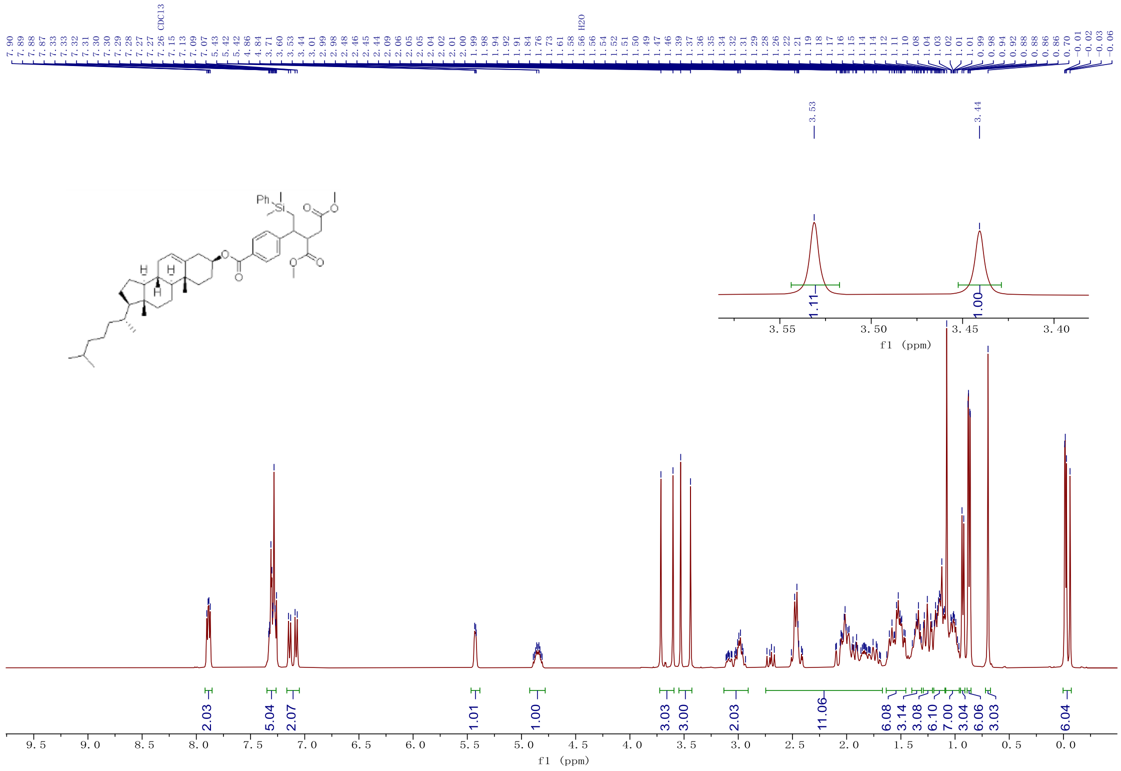

^{13}C NMR spectra of compound **d-70** in $CDCl_3$ (101 MHz): (*see procedure*)

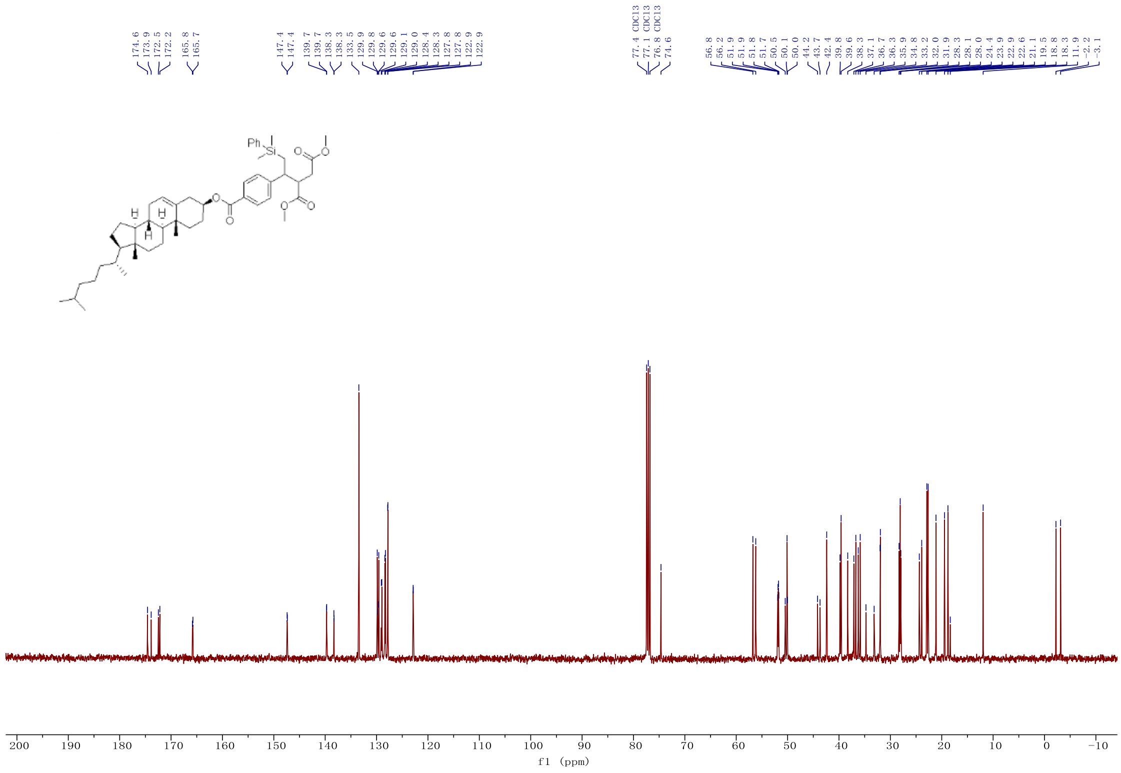

^{1}H NMR spectra of compound **d-71** in $CDCl_3$ (400 MHz): (*see procedure*)

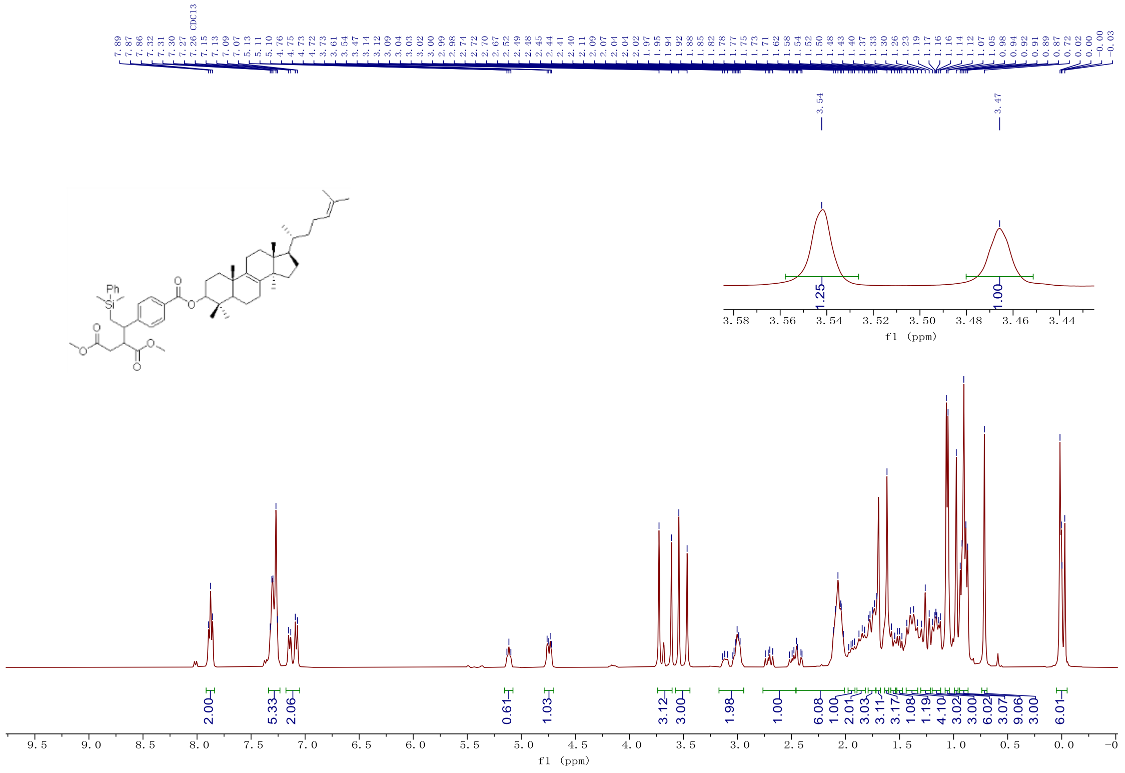

^{13}C NMR spectra of compound **d-71** in $CDCl_3$ (101 MHz): (*see procedure*)

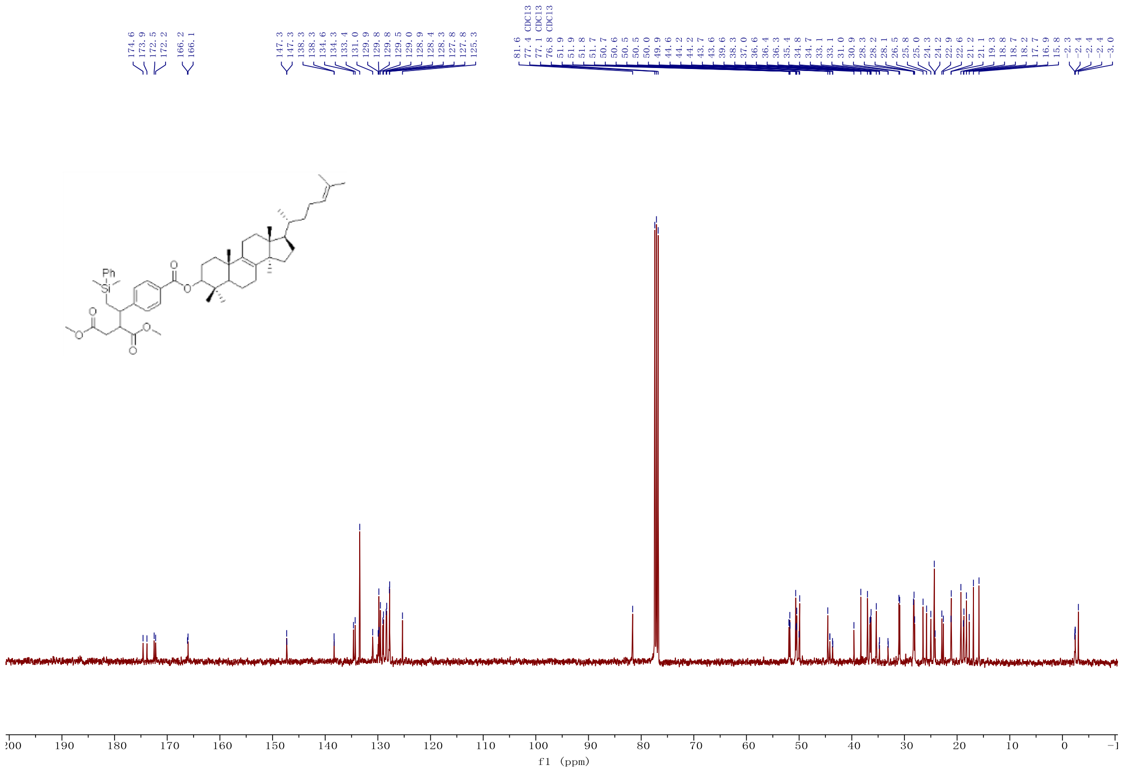

^{1}H NMR spectra of compound **d-72** in $CDCl_3$ (400 MHz): (*see procedure*)

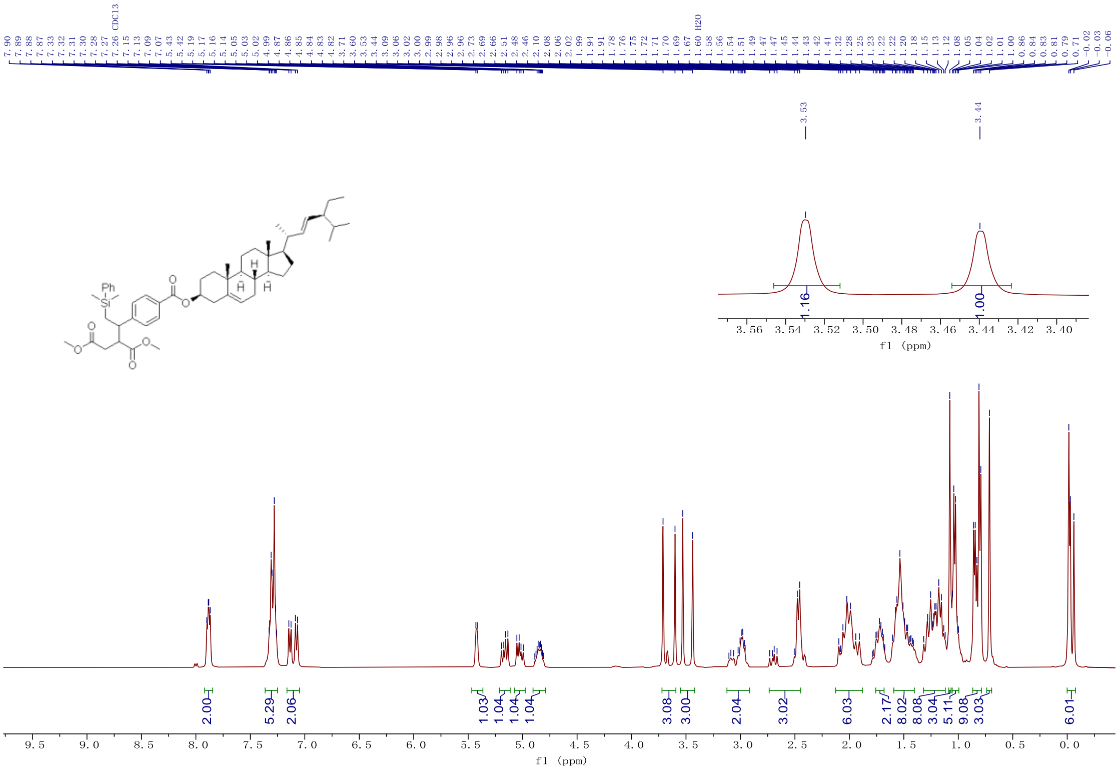

^{13}C NMR spectra of compound **d-72** in $CDCl_3$ (101 MHz): (*see procedure*)

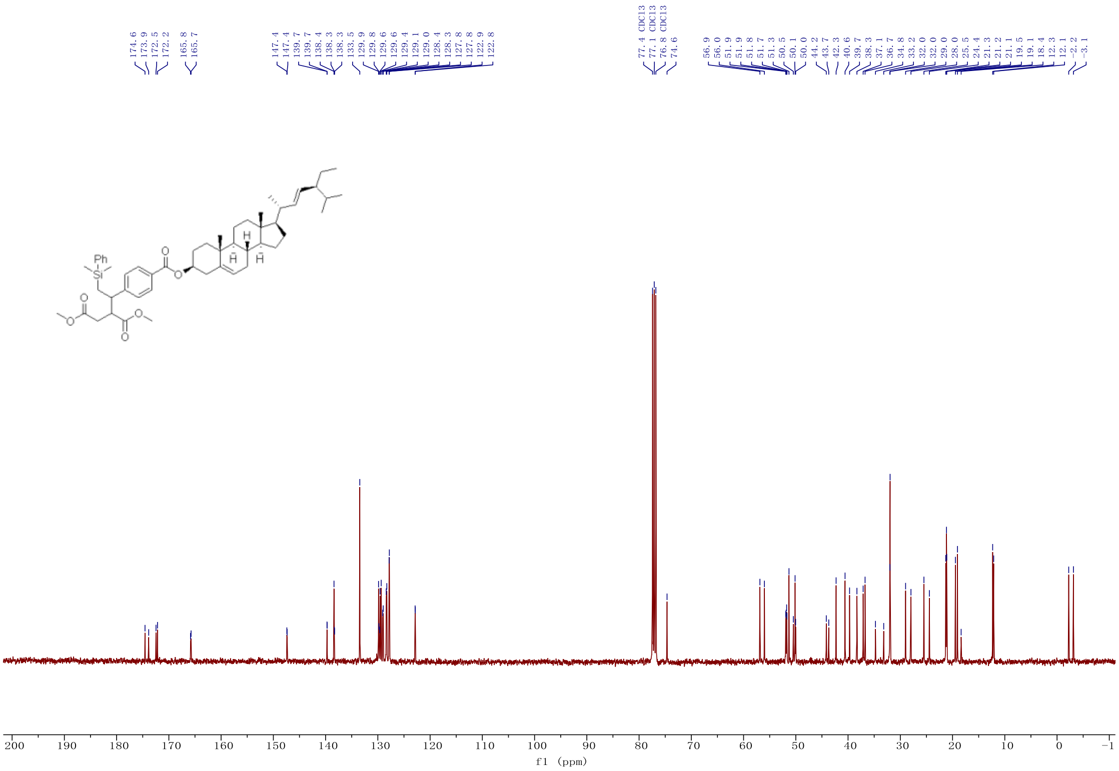

^{1}H NMR spectra of compound **d-73** in $CDCl_3$ (400 MHz): (*see procedure*)

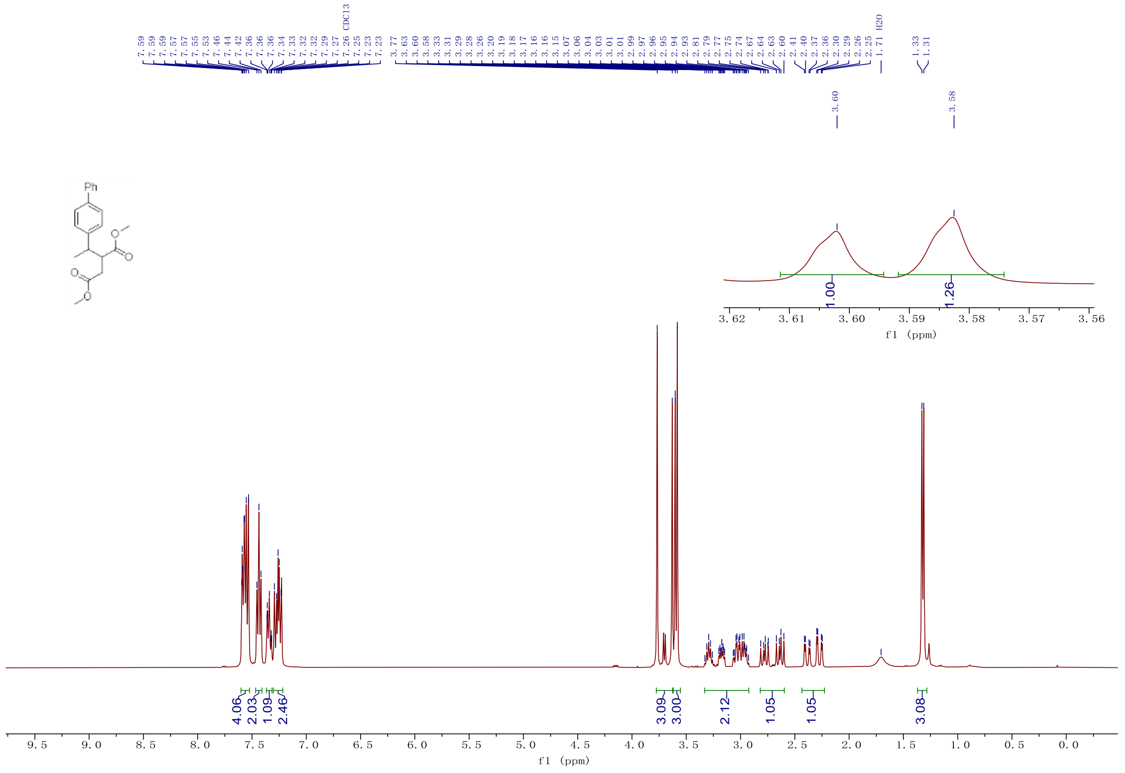

^{13}C NMR spectra of compound **d-73** in $CDCl_3$ (101 MHz): (*see procedure*)

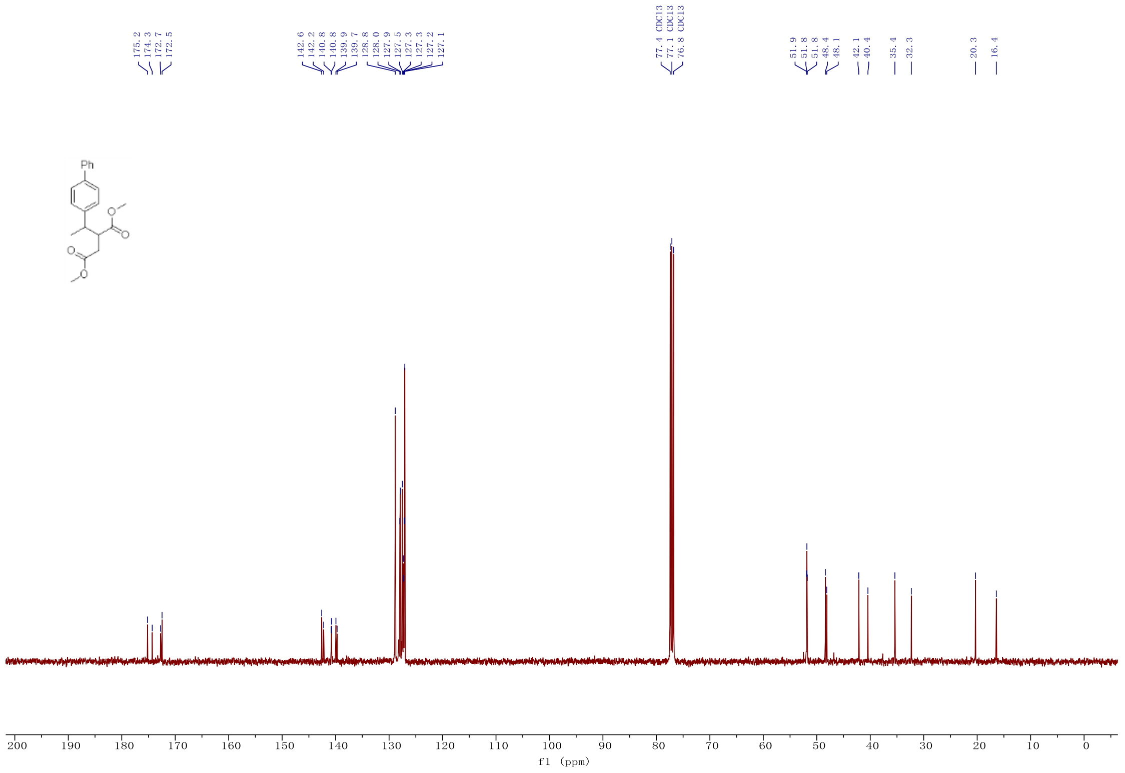

^{1}H NMR spectra of compound **d-74** in $CDCl_3$ (400 MHz): (*see procedure*)

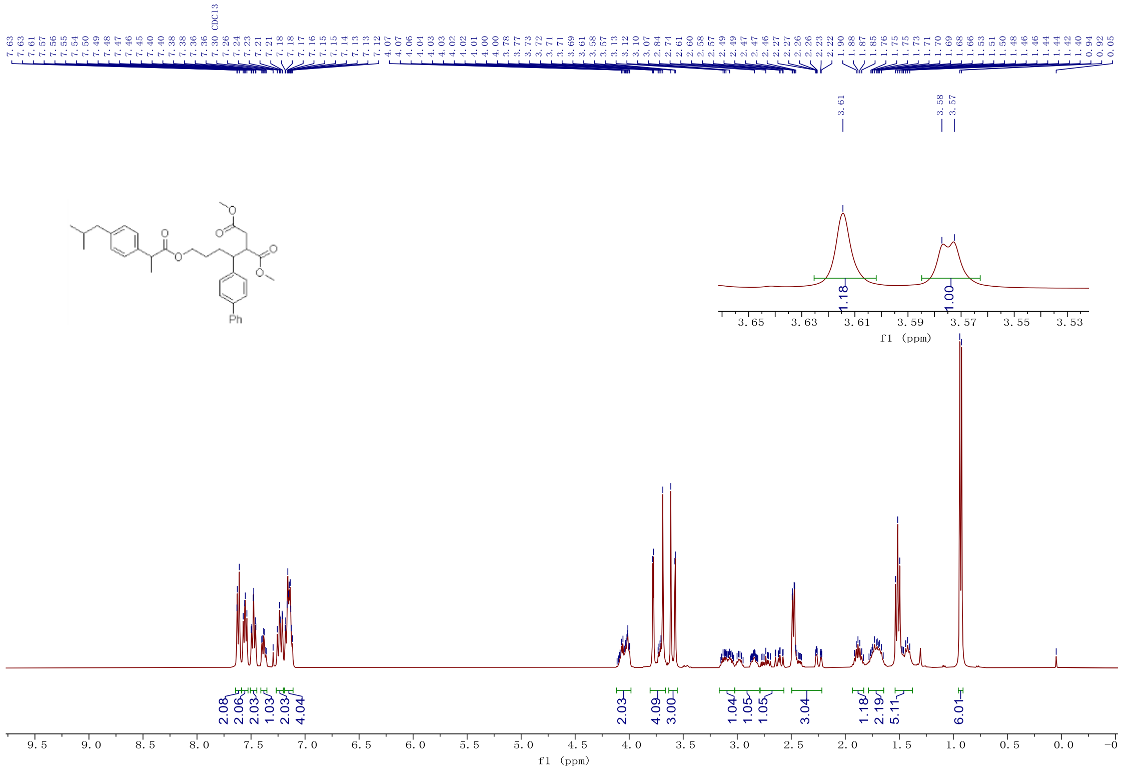

^{13}C NMR spectra of compound **d-74** in $CDCl_3$ (101 MHz): (*see procedure*)

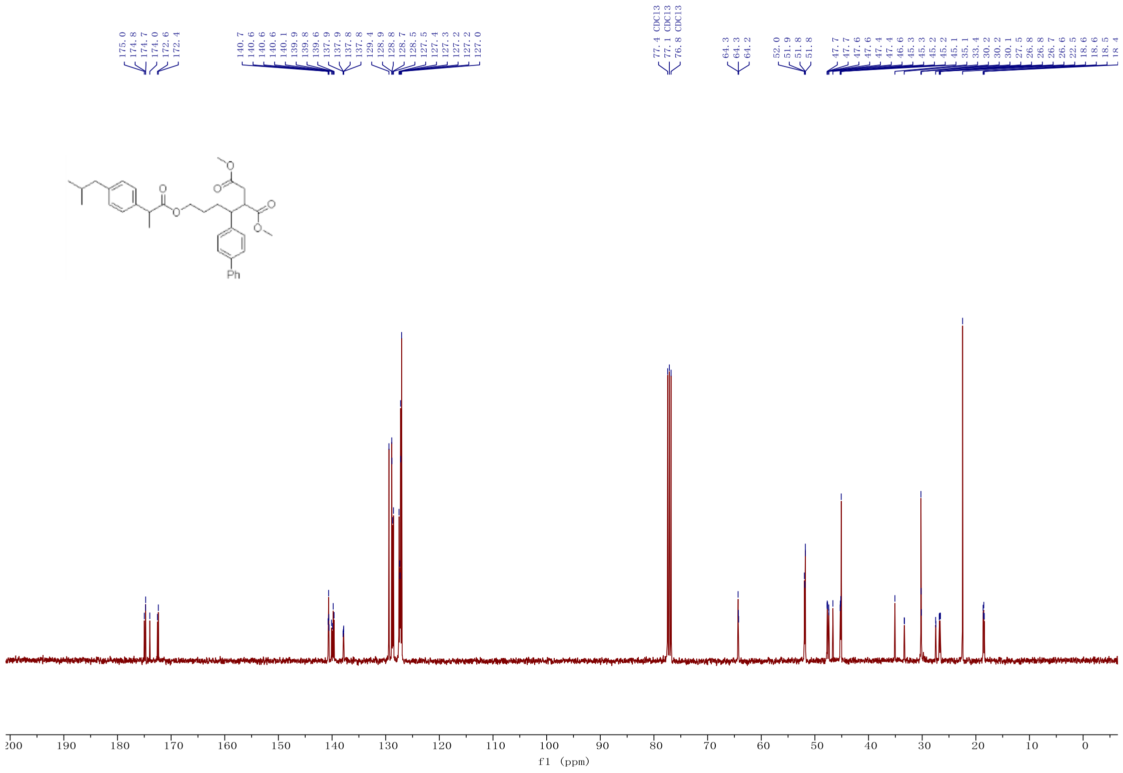

^{1}H NMR spectra of compound **d-75** in $CDCl_3$ (400 MHz): (*see procedure*)

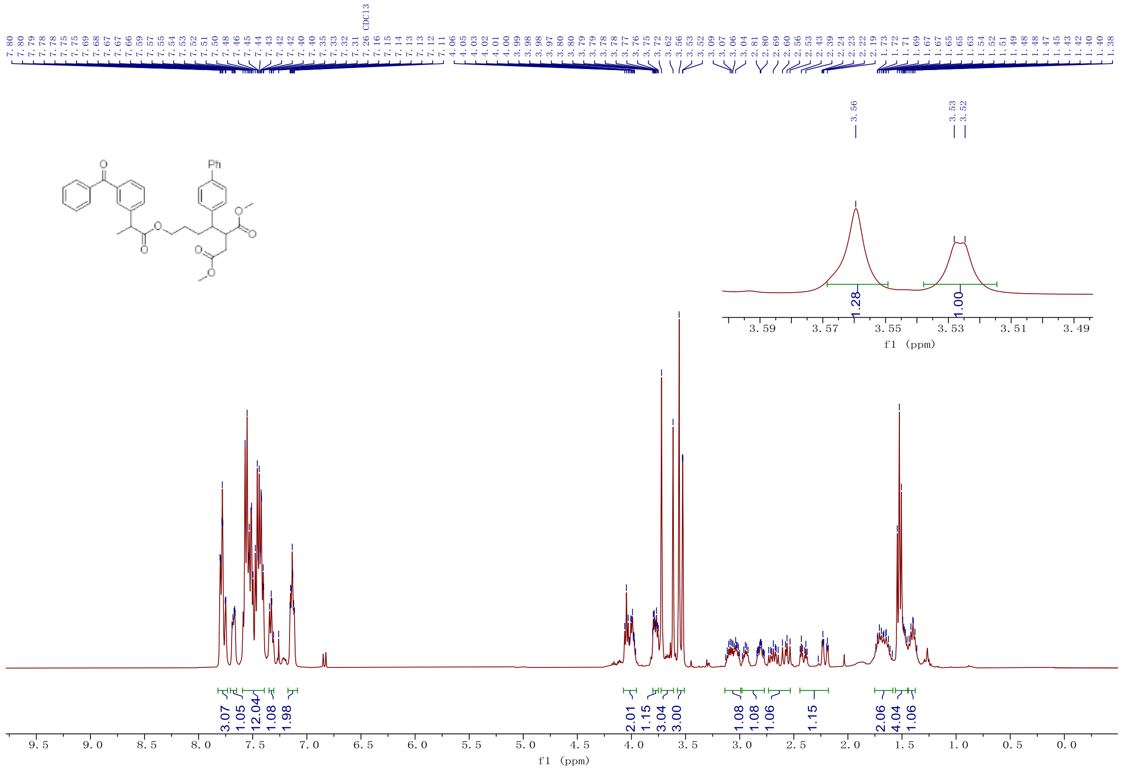

^{13}C NMR spectra of compound **d-75** in $CDCl_3$ (101 MHz): (*see procedure*)

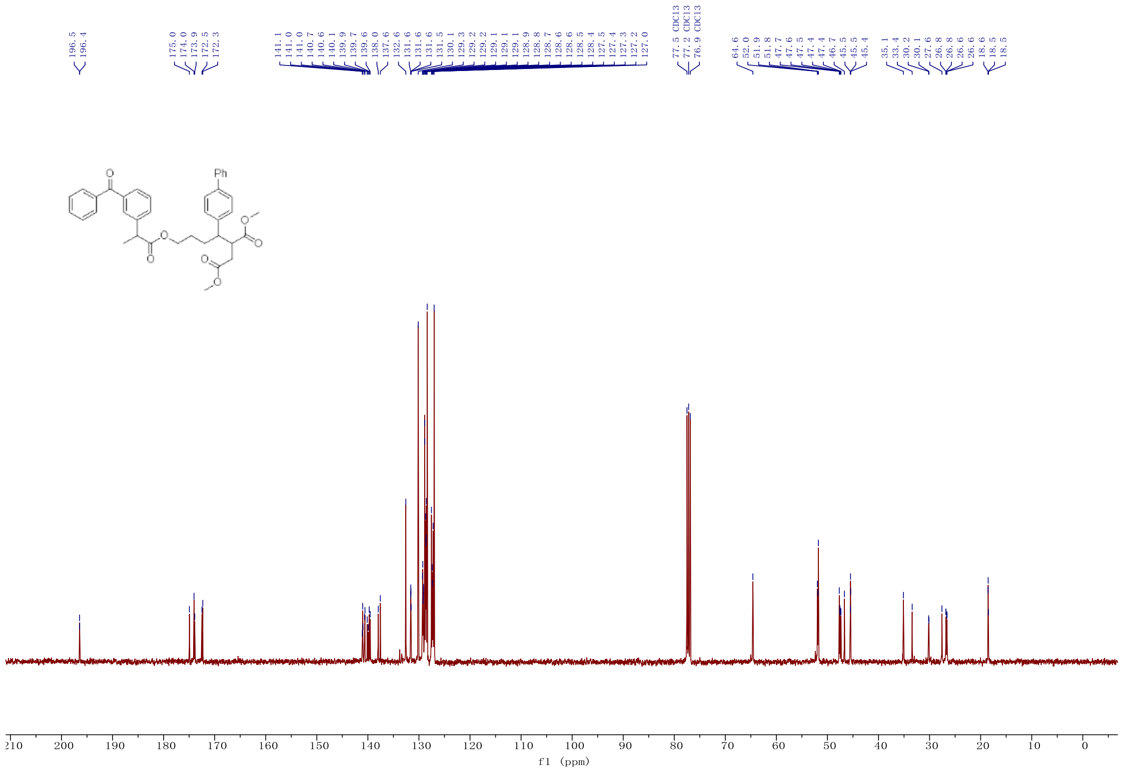

^{1}H NMR spectra of compound **d-76** in $CDCl_3$ (400 MHz): (see procedure)

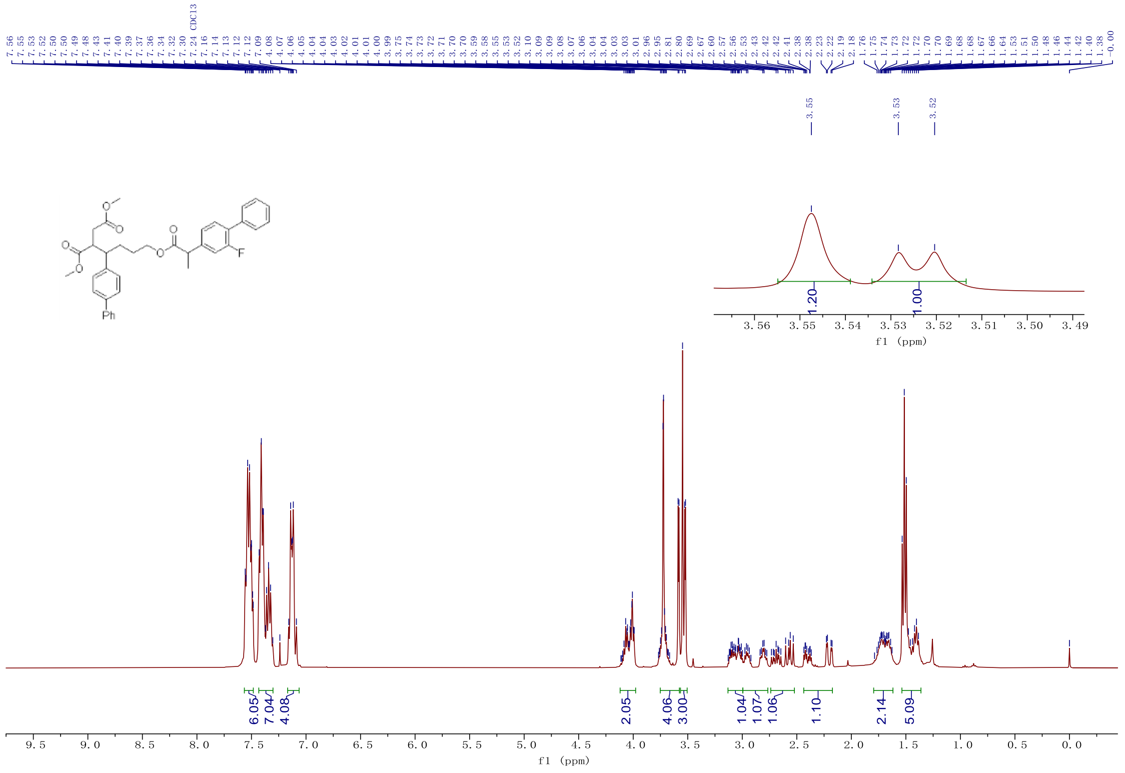

^{13}C NMR spectra of compound **d-76** in $CDCl_3$ (101 MHz): (see procedure)

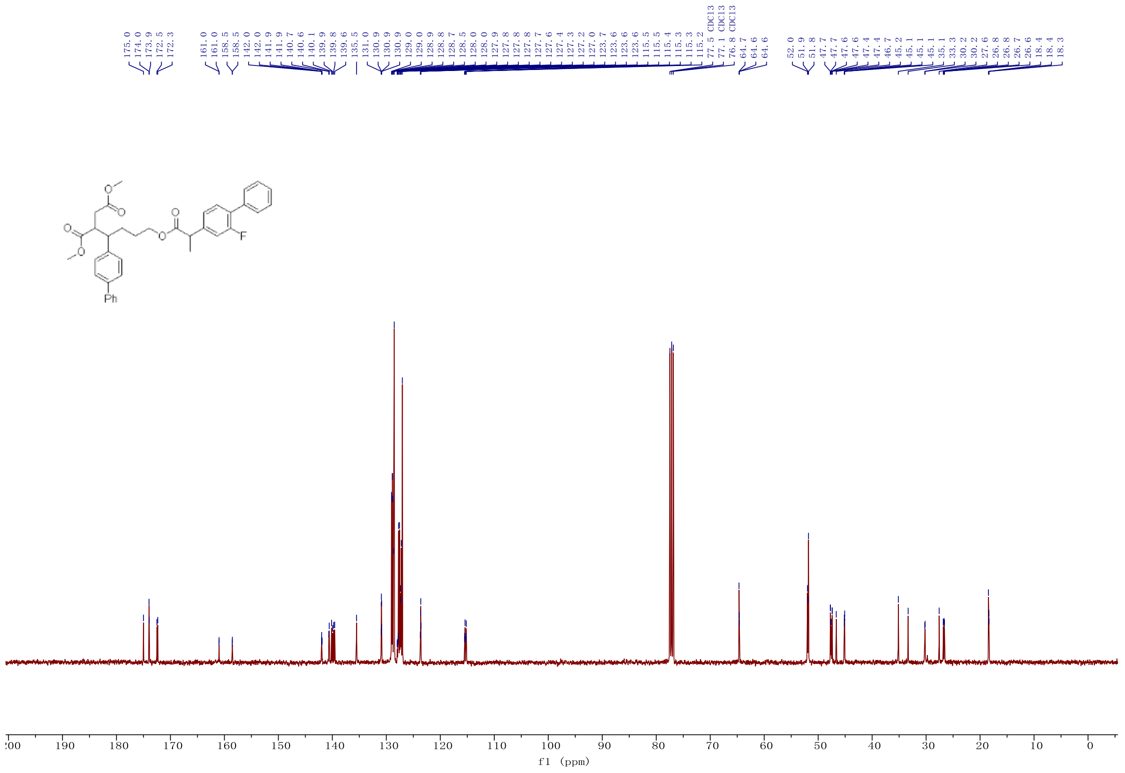

^{19}F NMR spectra of compound **d-76** in $CDCl_3$ (376 MHz): (*see procedure*)

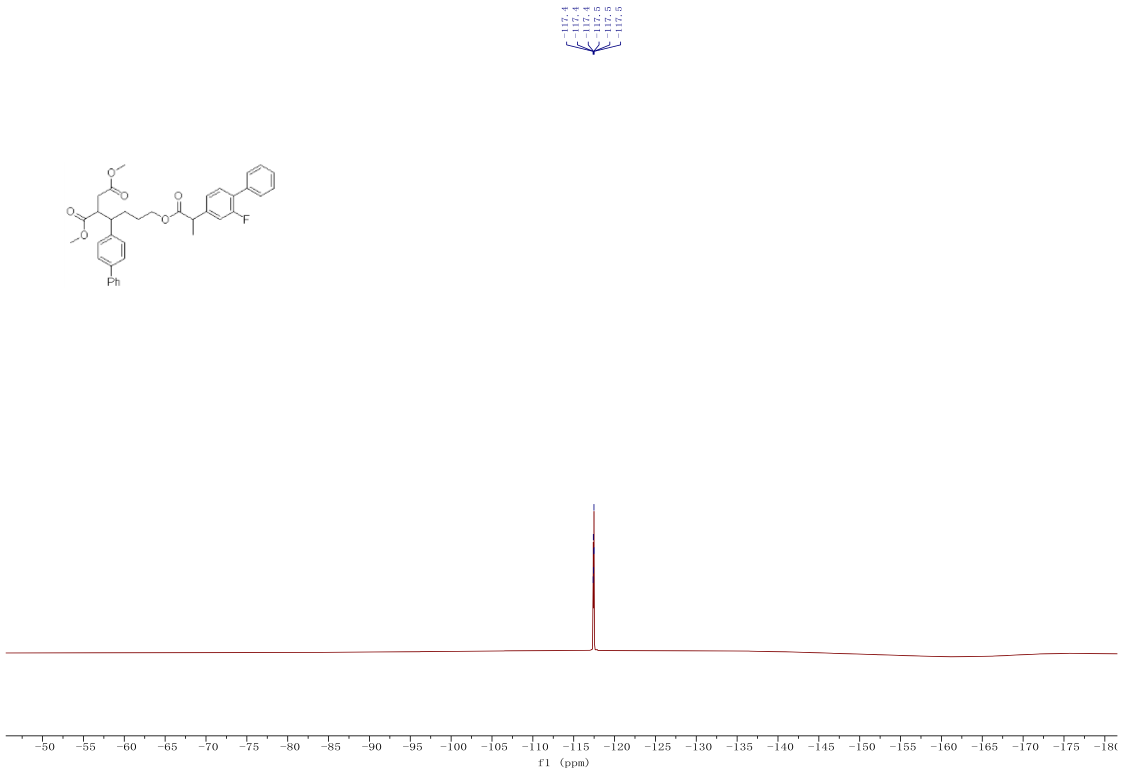

^{1}H NMR spectra of compound **d-77** in $CDCl_3$ (400 MHz): (*see procedure*)

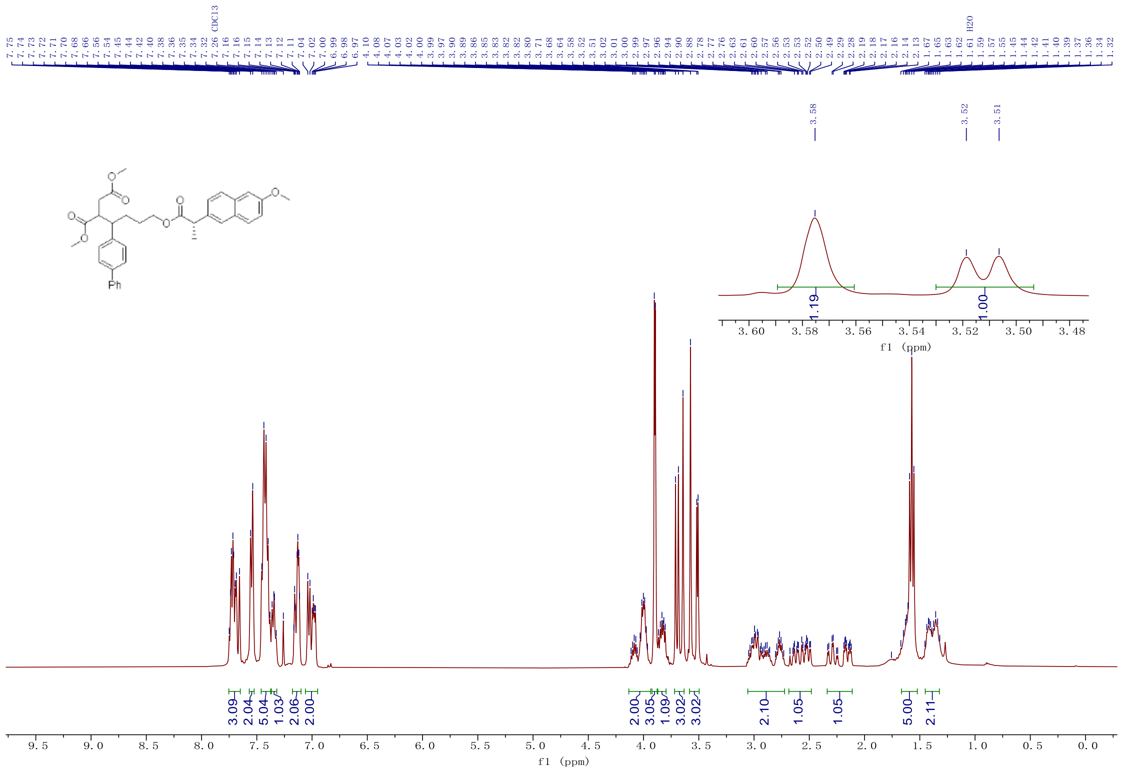

^{13}C NMR spectra of compound **d-77** in $CDCl_3$ (101 MHz): (<u>see procedure</u>)

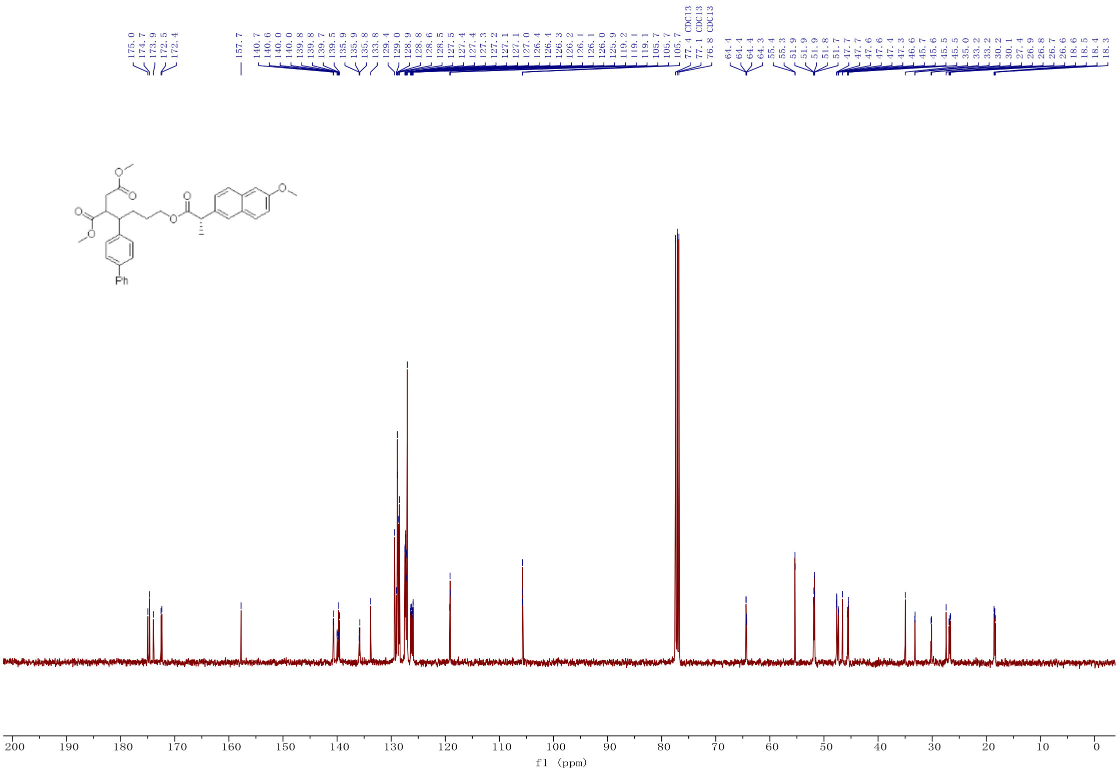

^{1}H NMR spectra of compound **d-78** in $CDCl_3$ (400 MHz): (<u>see procedure</u>)

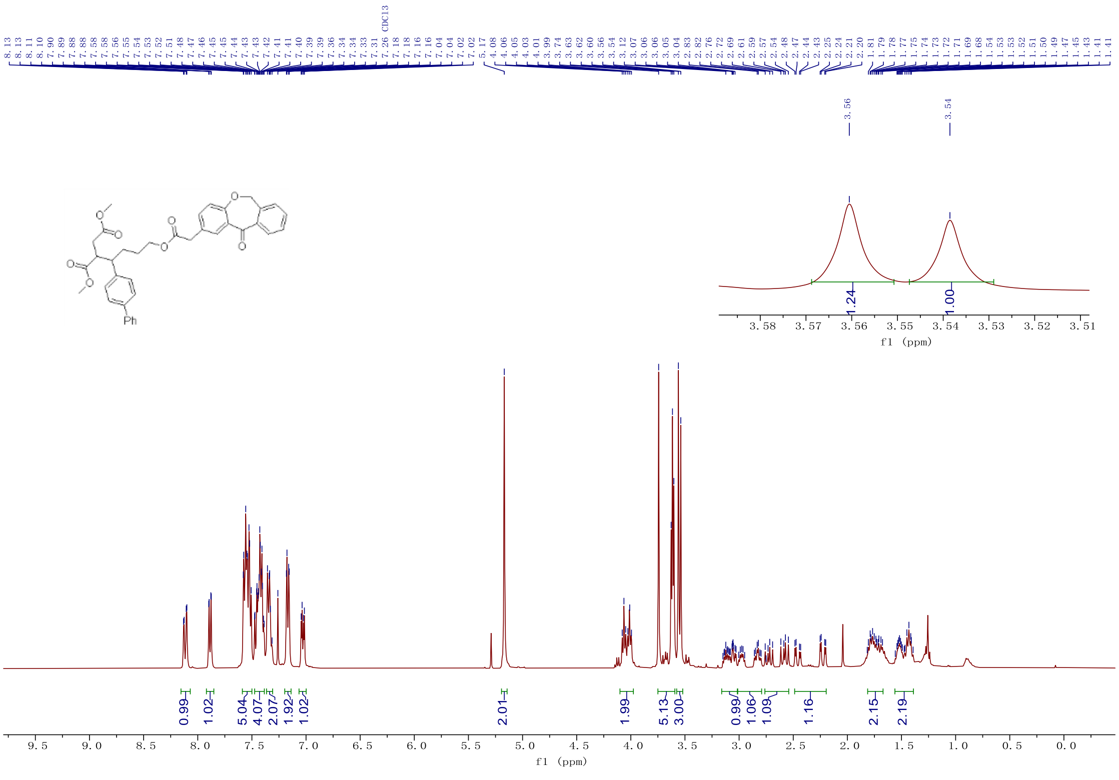

^{13}C NMR spectra of compound **d-78** in $CDCl_3$ (101 MHz): (*see procedure*)

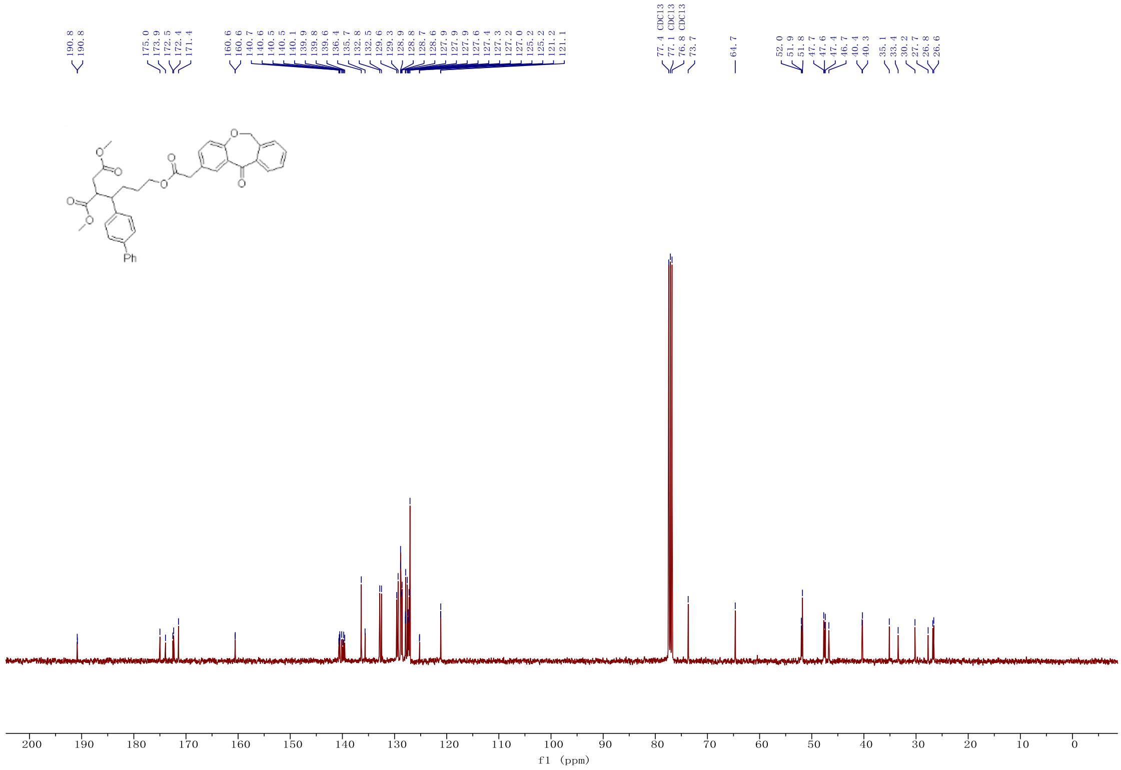

^{1}H NMR spectra of compound **d-79** in $CDCl_3$ (400 MHz): (*see procedure*)

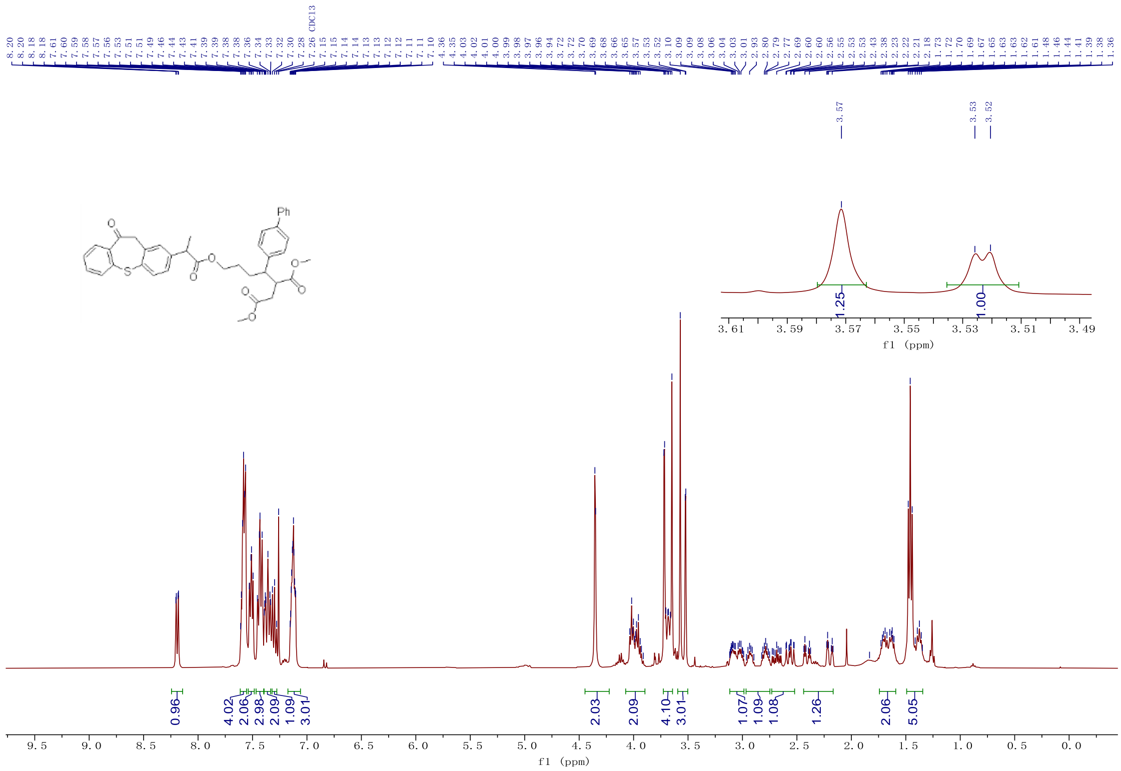

^{13}C NMR spectra of compound **d-79** in $CDCl_3$ (101 MHz): (see procedure)

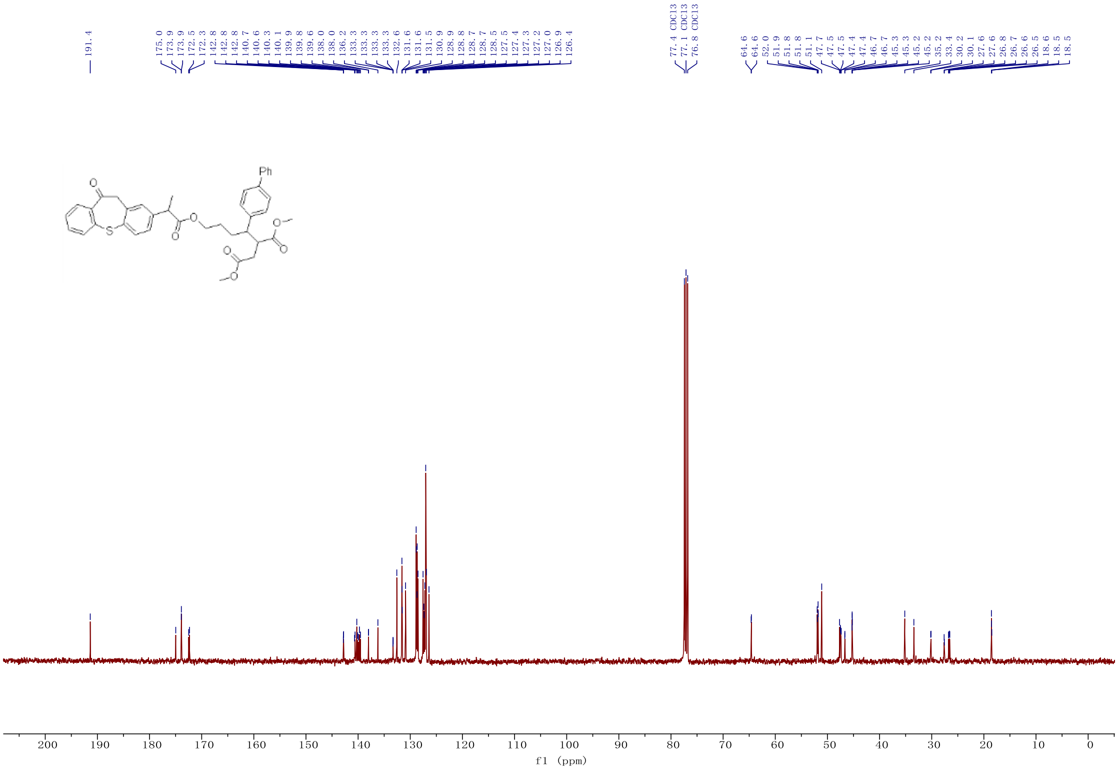

^{1}H NMR spectra of compound **d-80** in $CDCl_3$ (400 MHz): (see procedure)

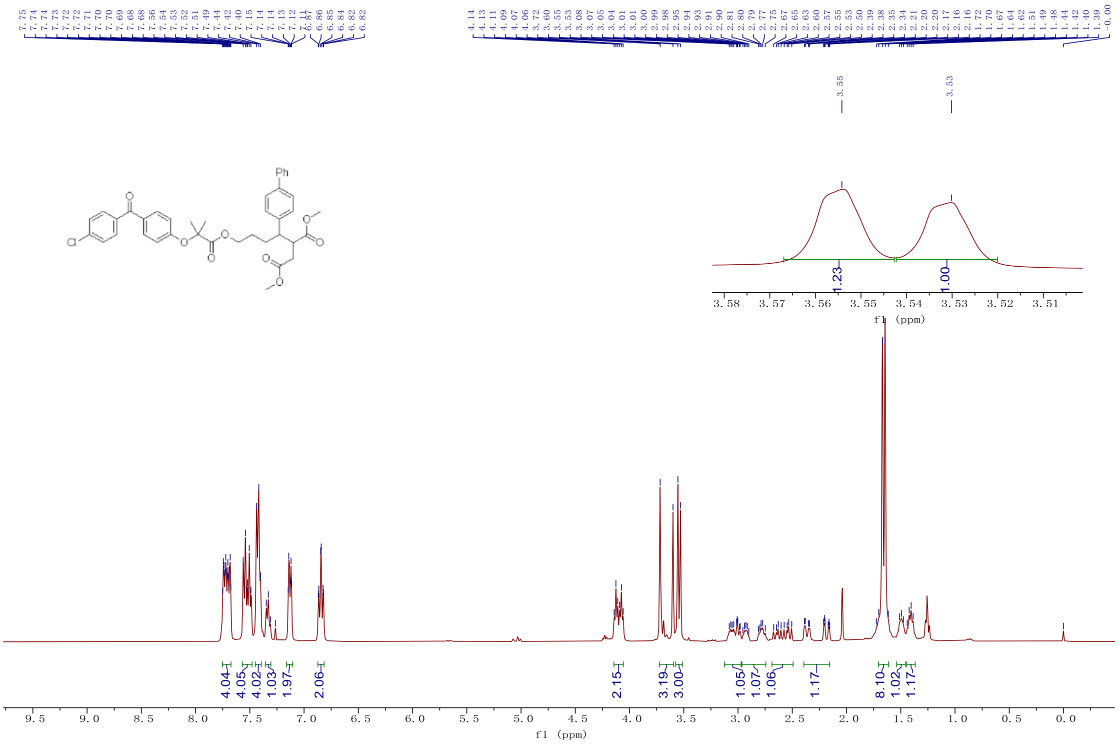

^{13}C NMR spectra of compound **d-80** in $CDCl_3$ (101 MHz): (see procedure)

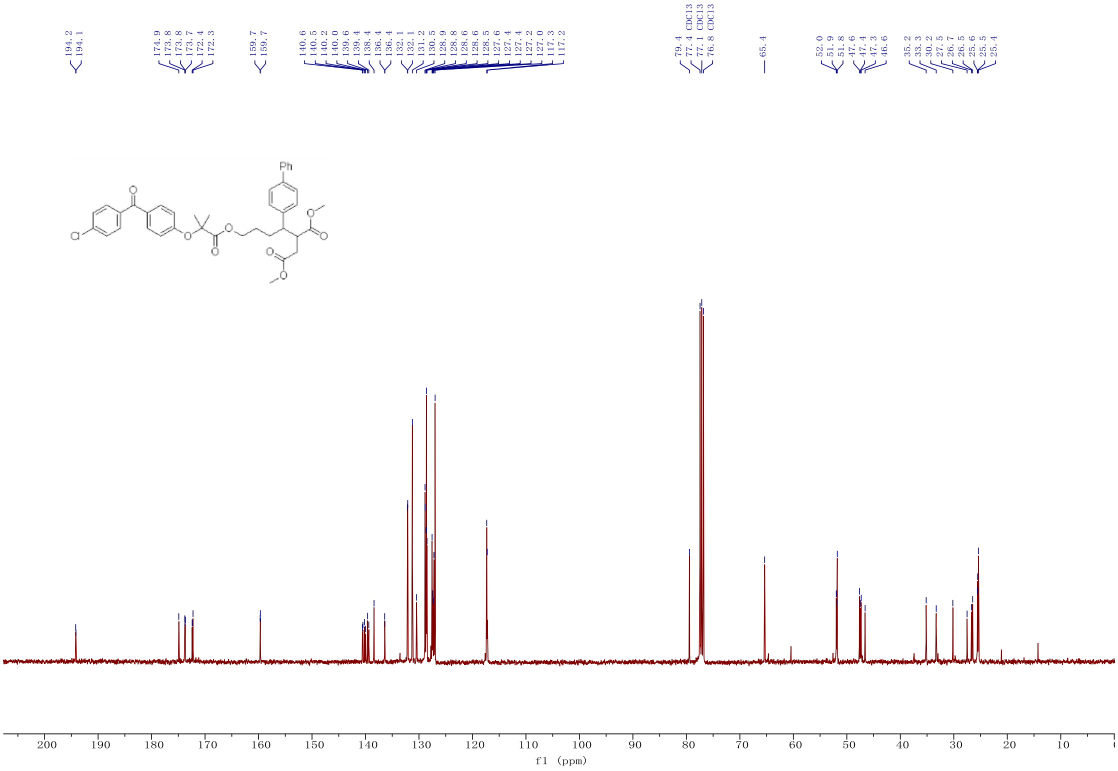

^{1}H NMR spectra of compound **e-1** in $CDCl_3$ (400 MHz): (see procedure)

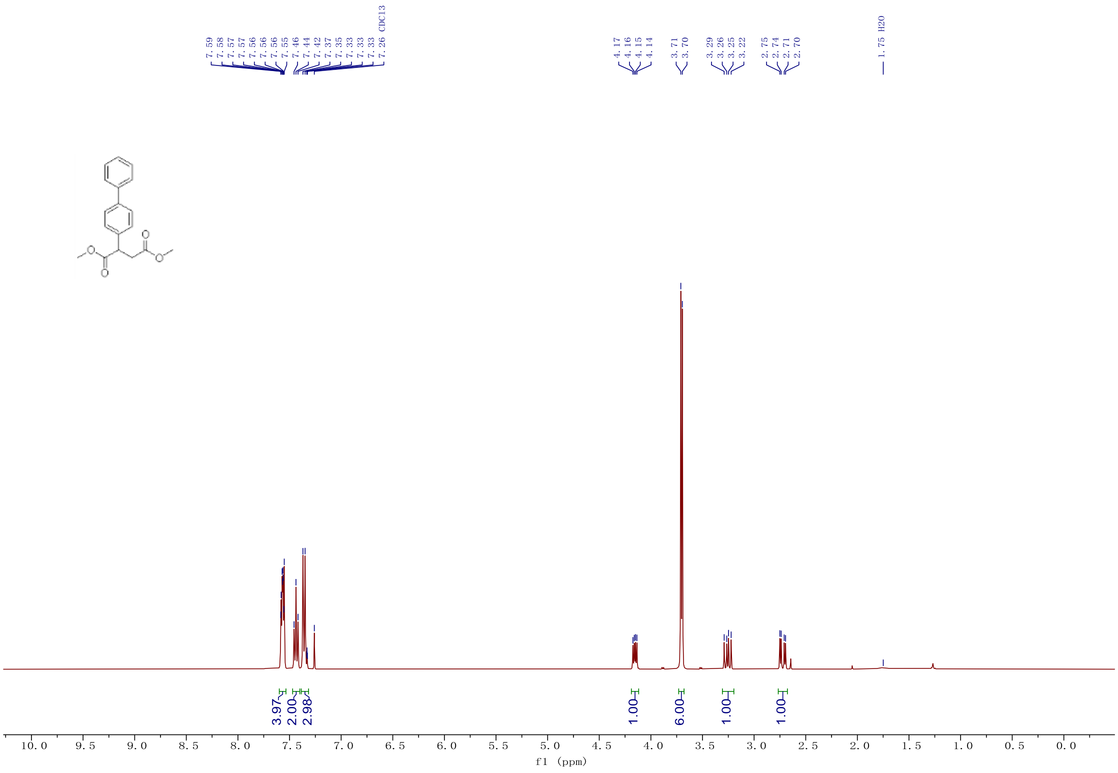

^{13}C NMR spectra of compound **e-1** in $CDCl_3$ (101 MHz): (*see procedure*)

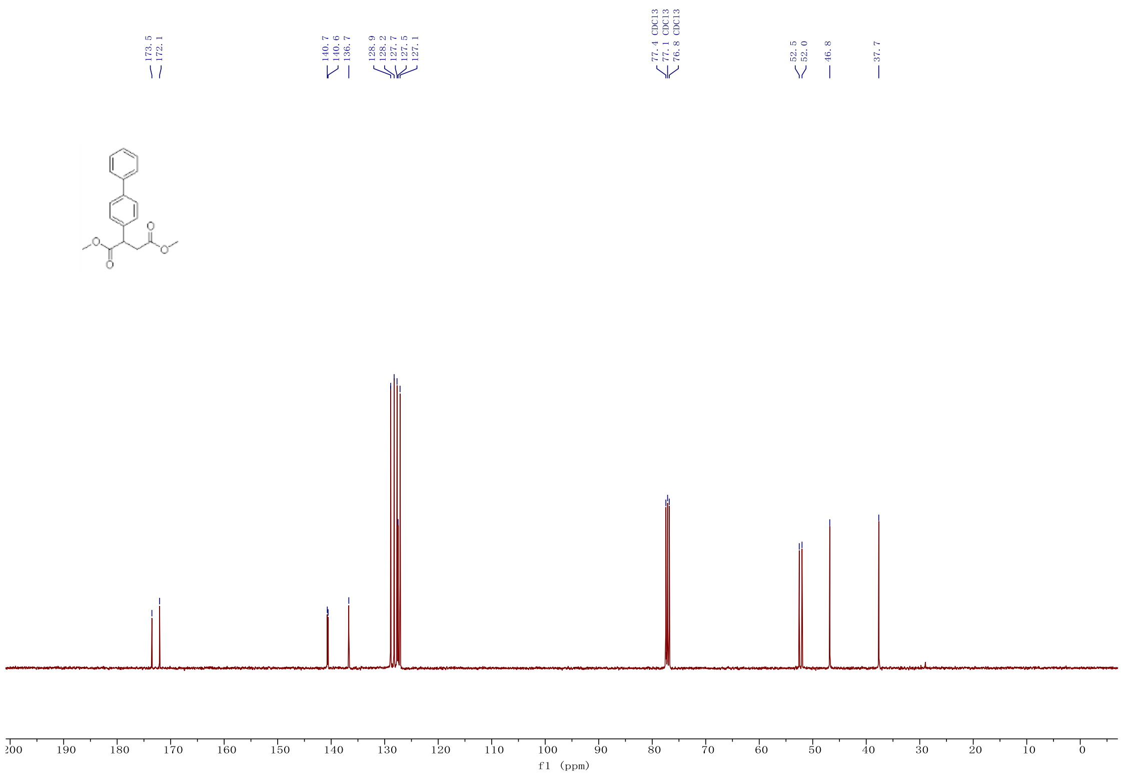

^{1}H NMR spectra of compound **d-81** in $CDCl_3$ (400 MHz): (*see procedure*)

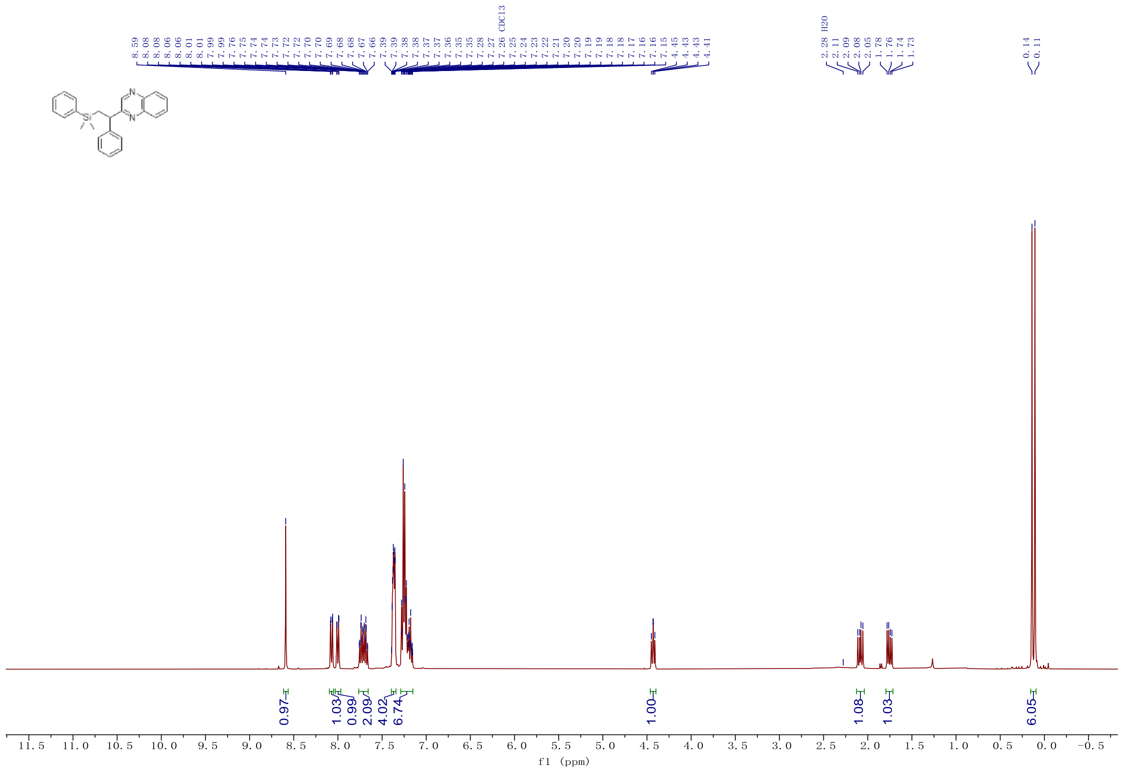

^{13}C NMR spectra of compound **d-81** in $CDCl_3$ (101 MHz): (<u>*see procedure*</u>)

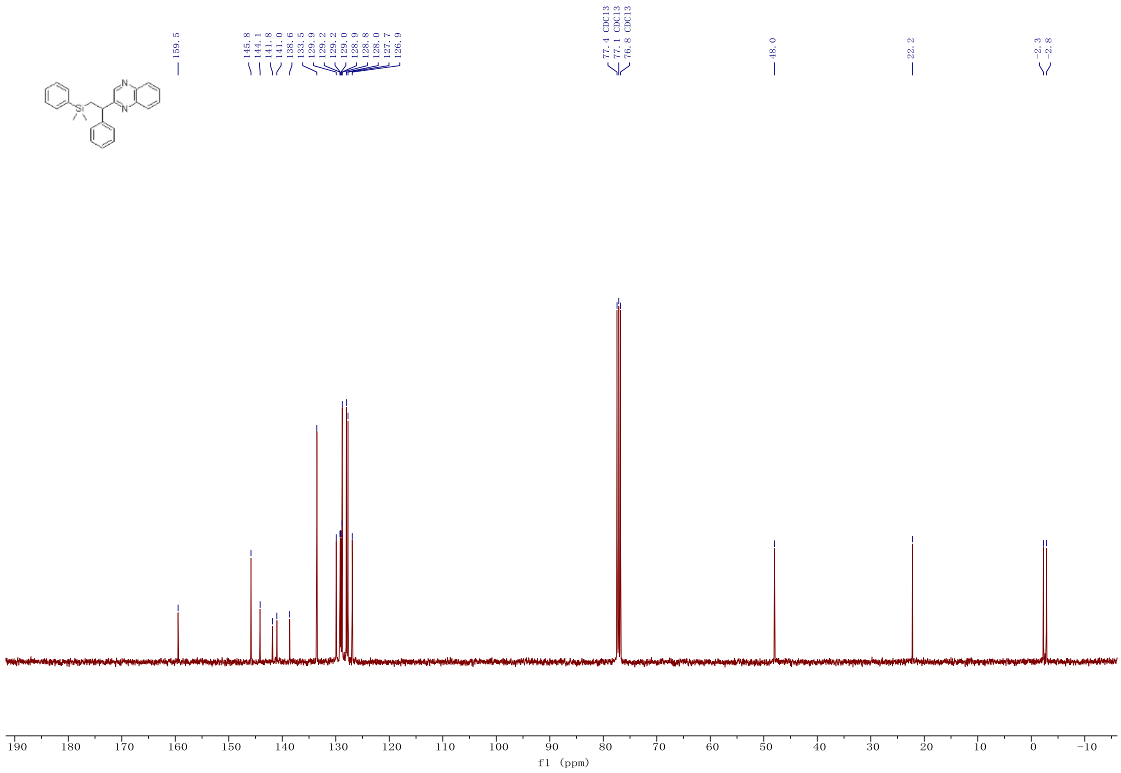

^{1}H NMR spectra of compound **d-82** in $CDCl_3$ (400 MHz): (<u>*see procedure*</u>)

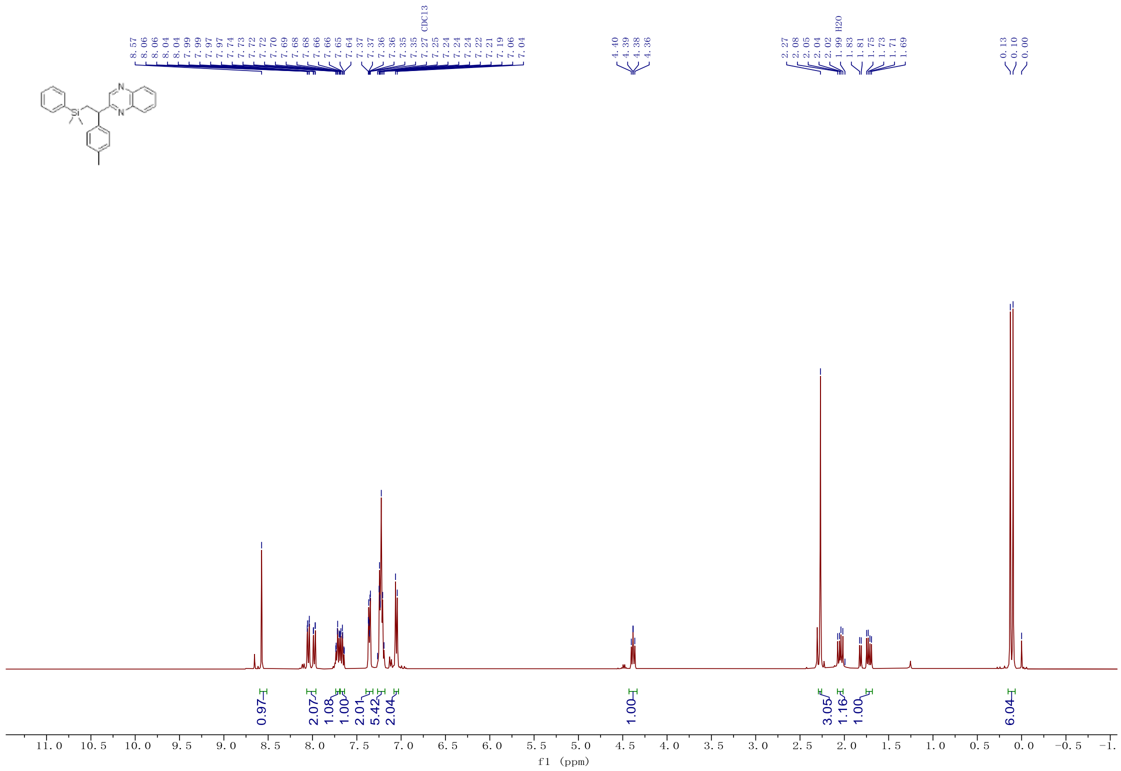

^{13}C NMR spectra of compound **d-82** in $CDCl_3$ (101 MHz): (*see procedure*)

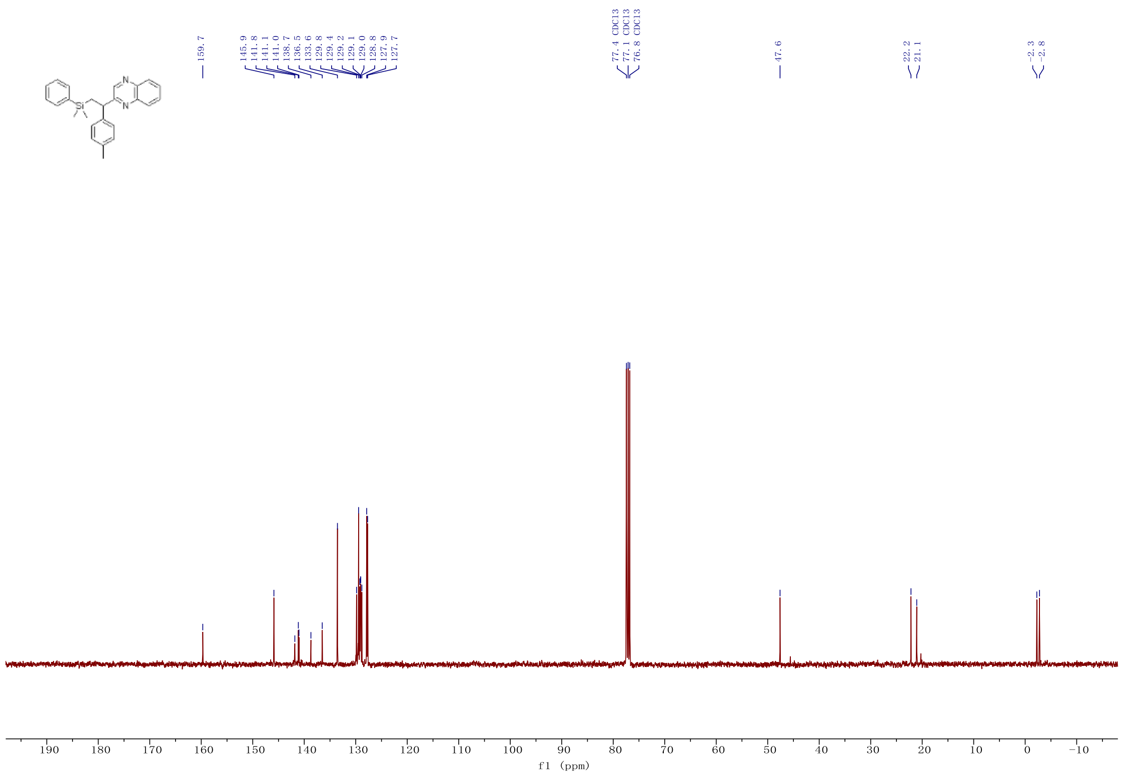

^{1}H NMR spectra of compound **d-83** in $CDCl_3$ (400 MHz): (*see procedure*)

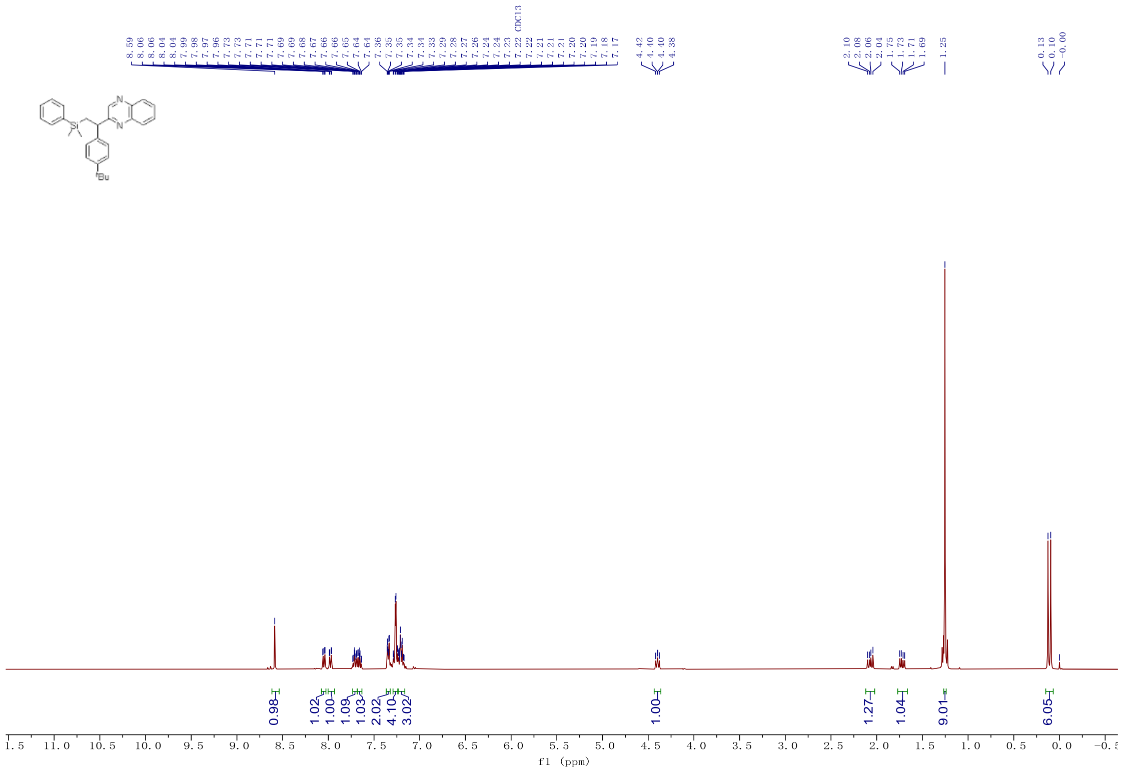

^{13}C NMR spectra of compound **d-83** in $CDCl_3$ (101 MHz): (*see procedure*)

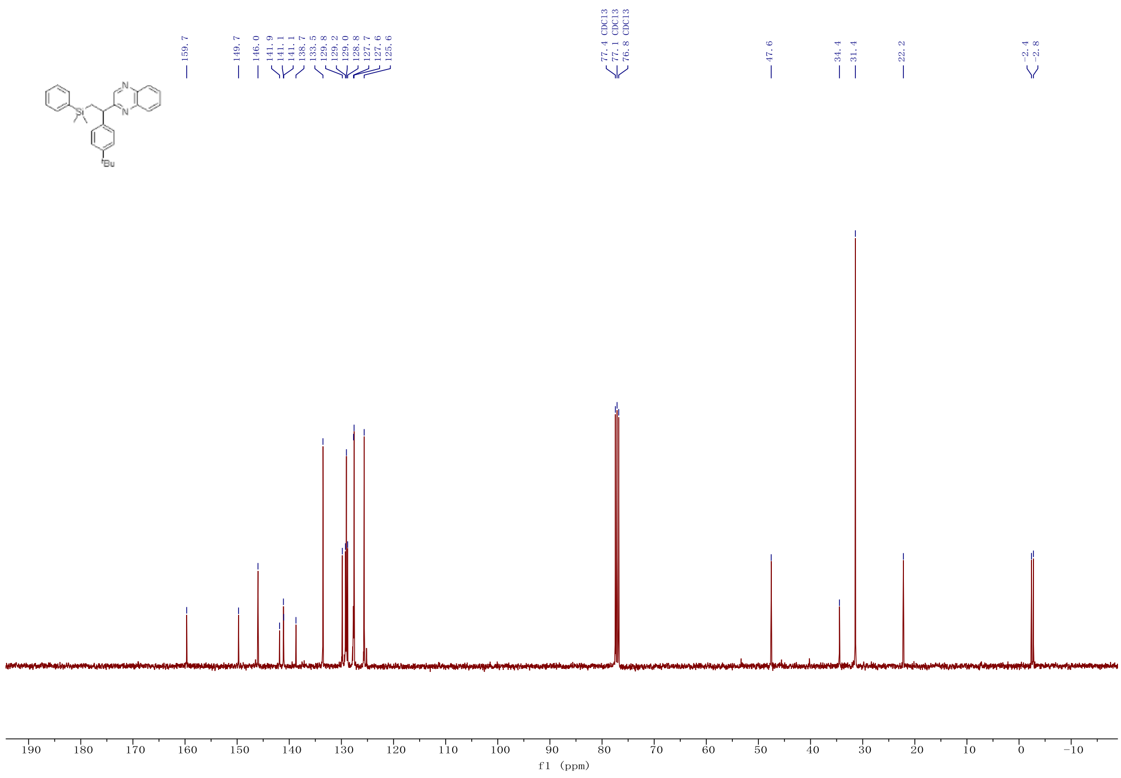

^{1}H NMR spectra of compound **d-84** in $CDCl_3$ (400 MHz): (*see procedure*)

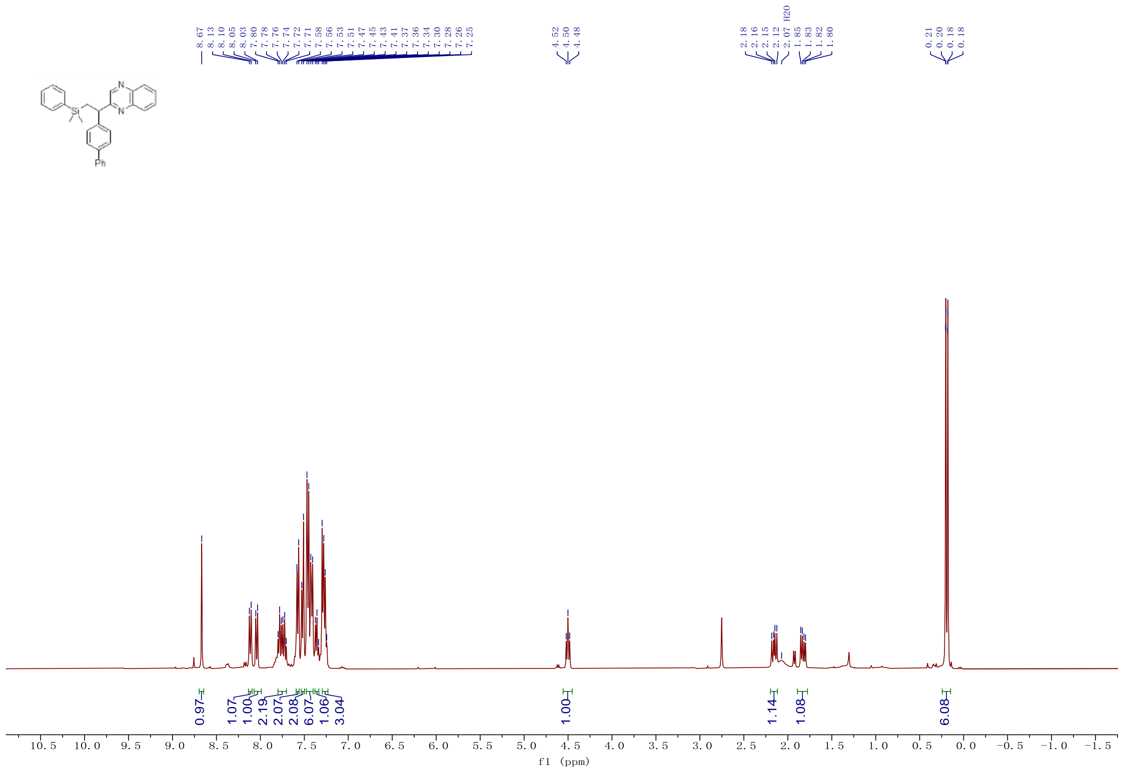

^{13}C NMR spectra of compound **d-84** in $CDCl_3$ (101 MHz): (*see procedure*)

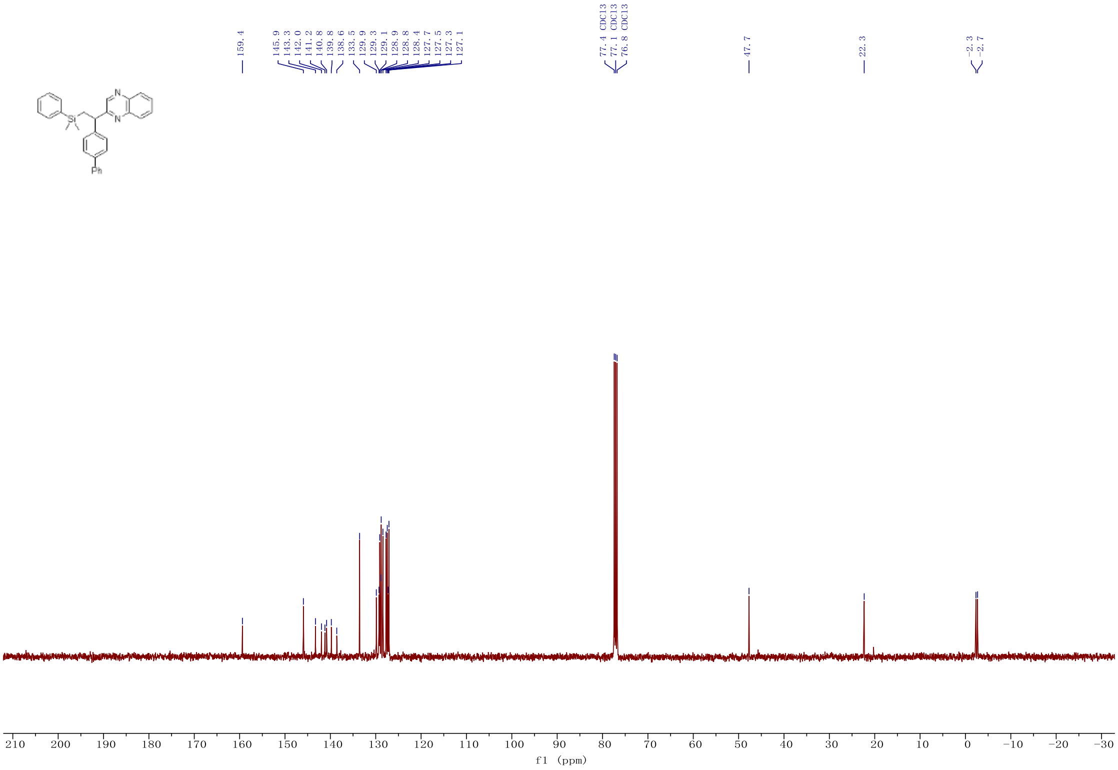

^{1}H NMR spectra of compound **d-85** in $CDCl_3$ (400 MHz): (*see procedure*)

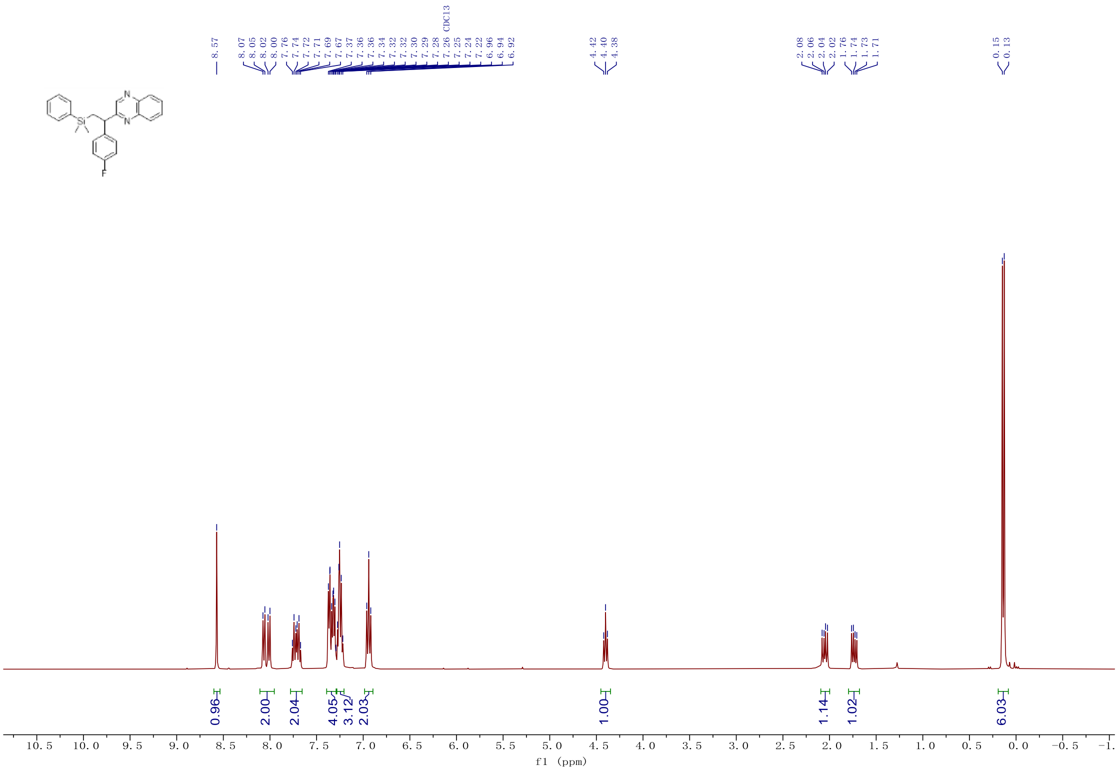

^{13}C NMR spectra of compound **d-85** in $CDCl_3$ (101 MHz): (*see procedure*)

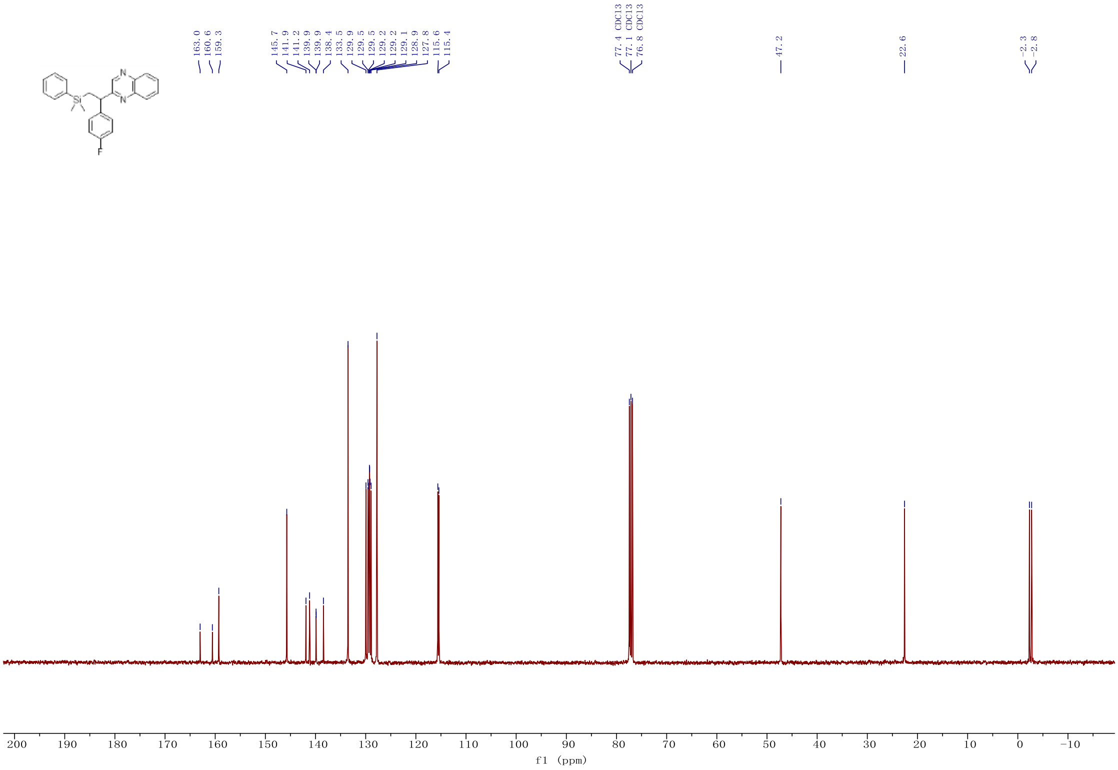

^{19}F NMR spectra of compound **d-85** in $CDCl_3$ (376 MHz): (*see procedure*)

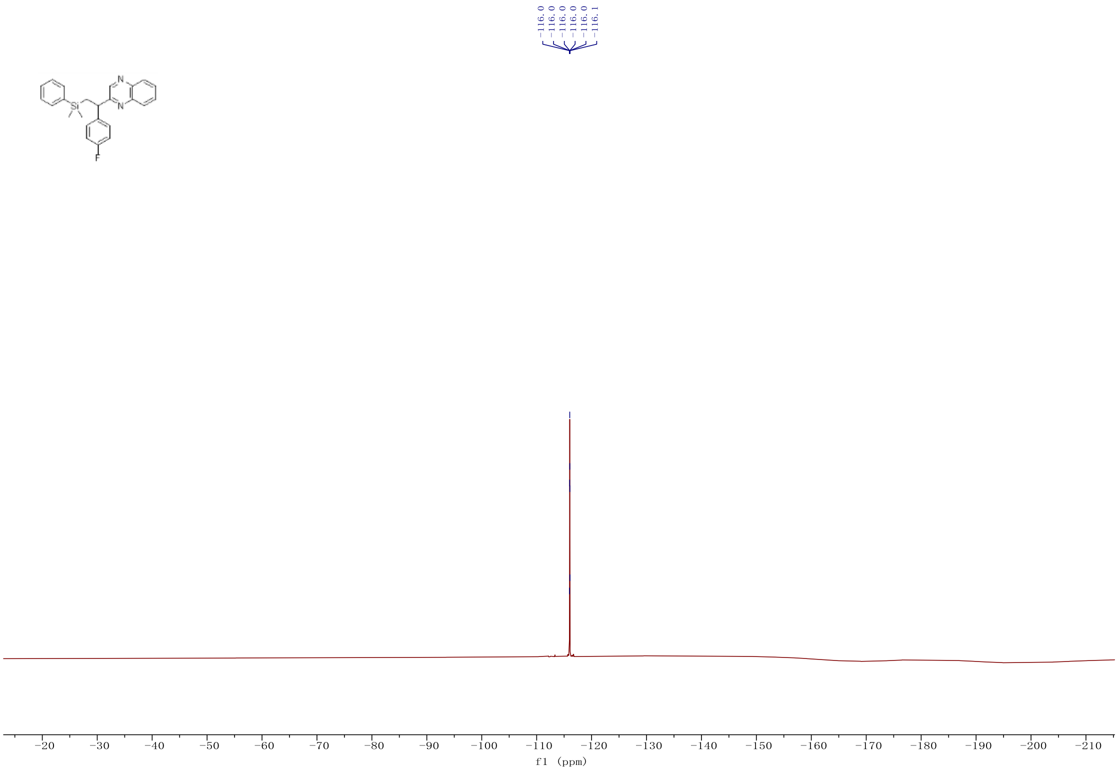

^{1}H NMR spectra of compound **d-86** in $CDCl_3$ (400 MHz): (*see procedure*)

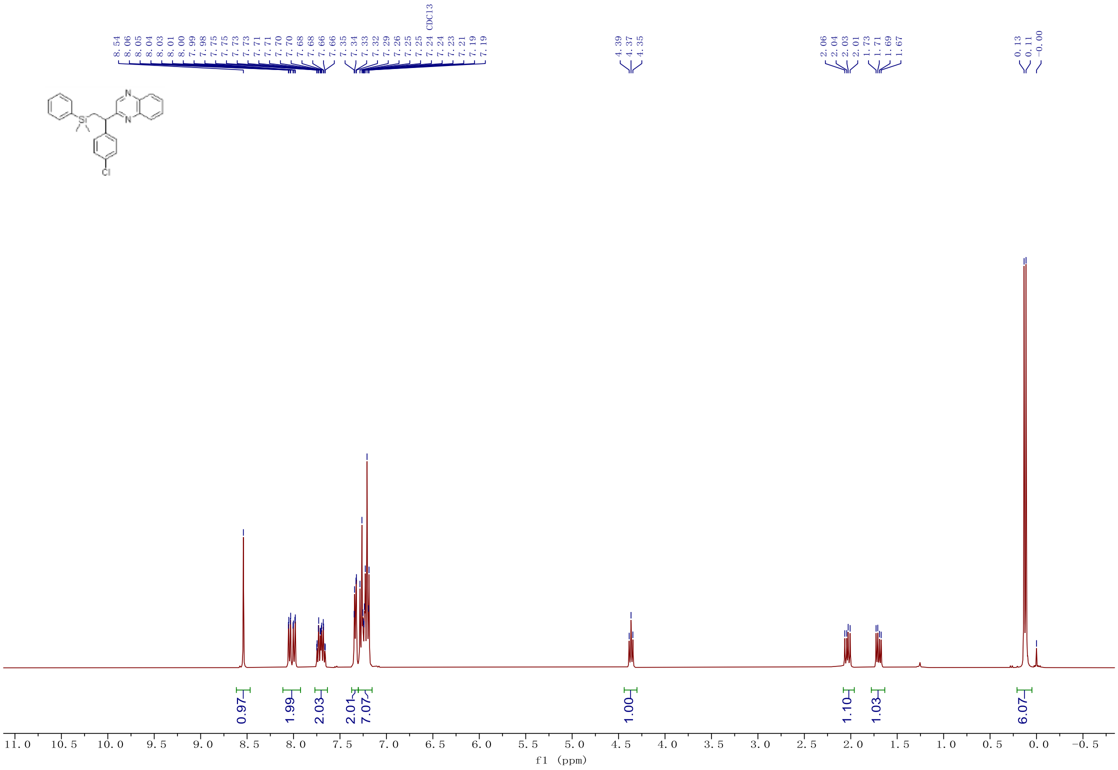

^{13}C NMR spectra of compound **d-86** in $CDCl_3$ (101 MHz): (*see procedure*)

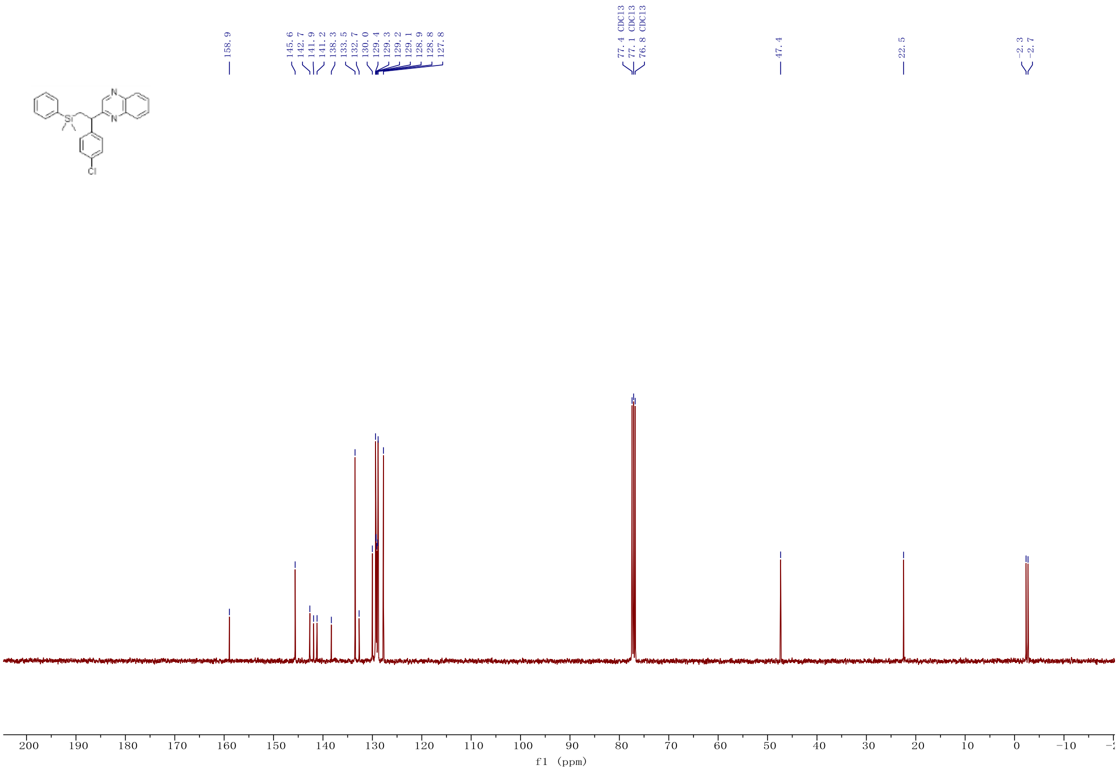

^{1}H NMR spectra of compound **d-87** in $CDCl_3$ (400 MHz): (*see procedure*)

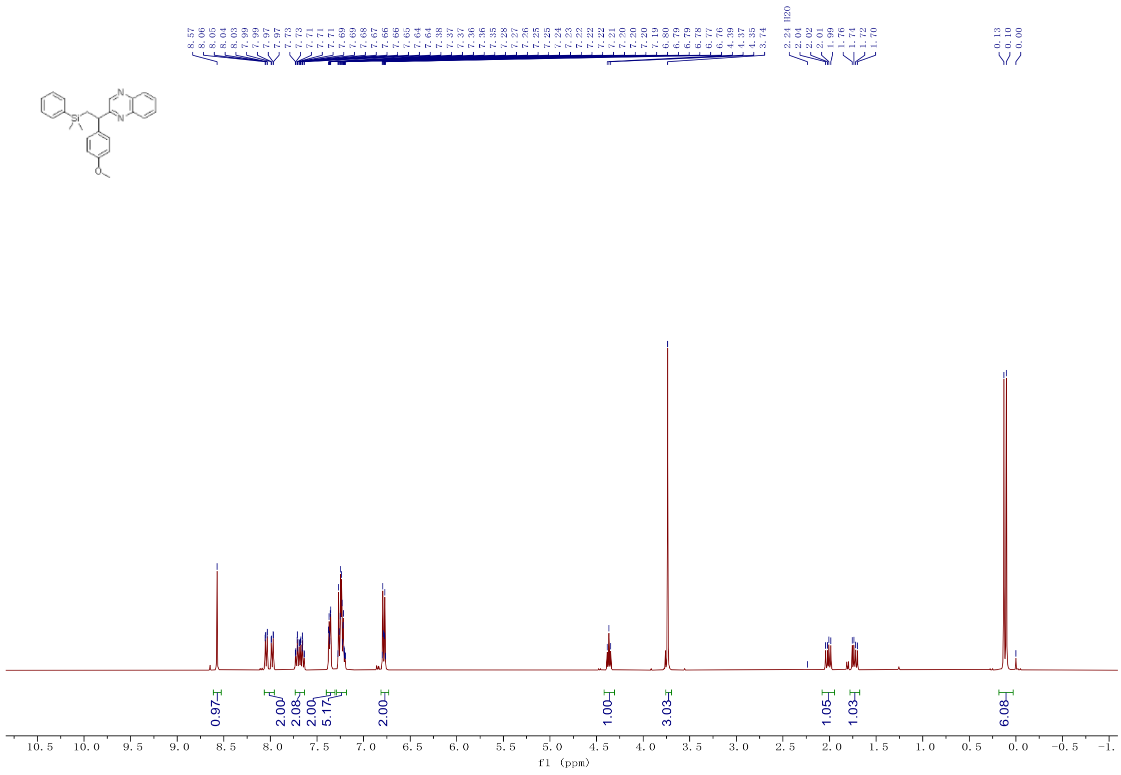

^{13}C NMR spectra of compound **d-87** in $CDCl_3$ (101 MHz): (*see procedure*)

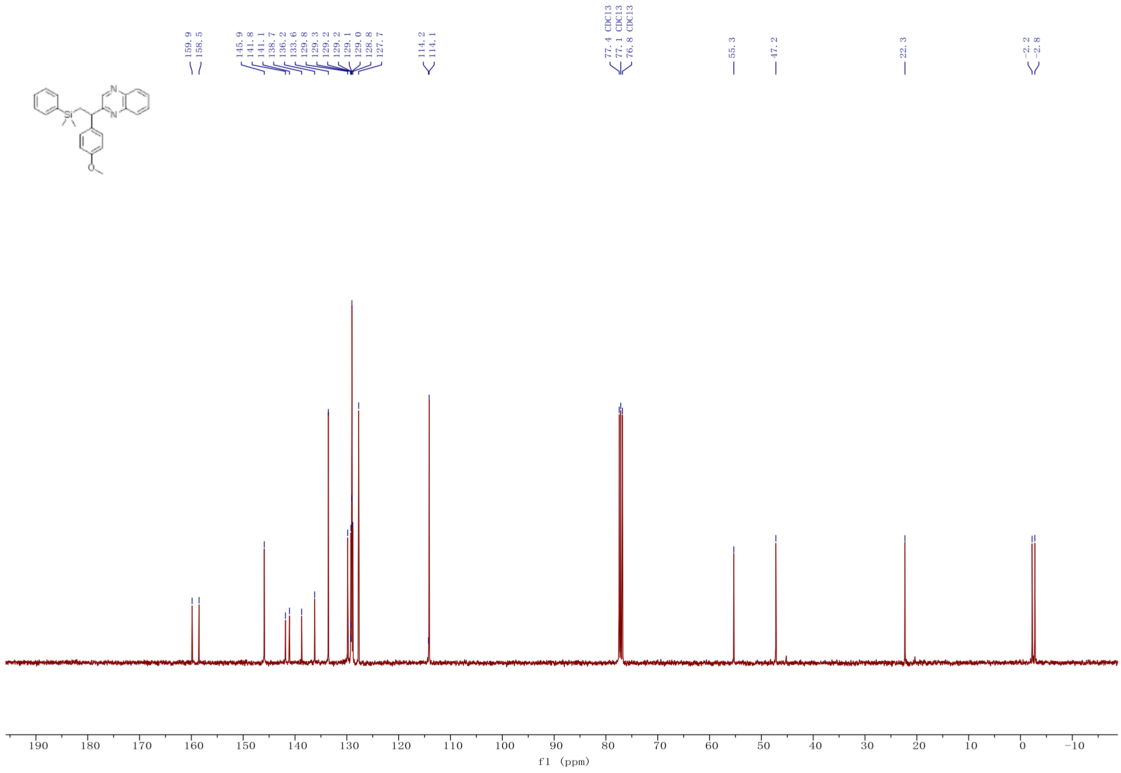

^{1}H NMR spectra of compound **d-88** in $CDCl_3$ (400 MHz): (*see procedure*)

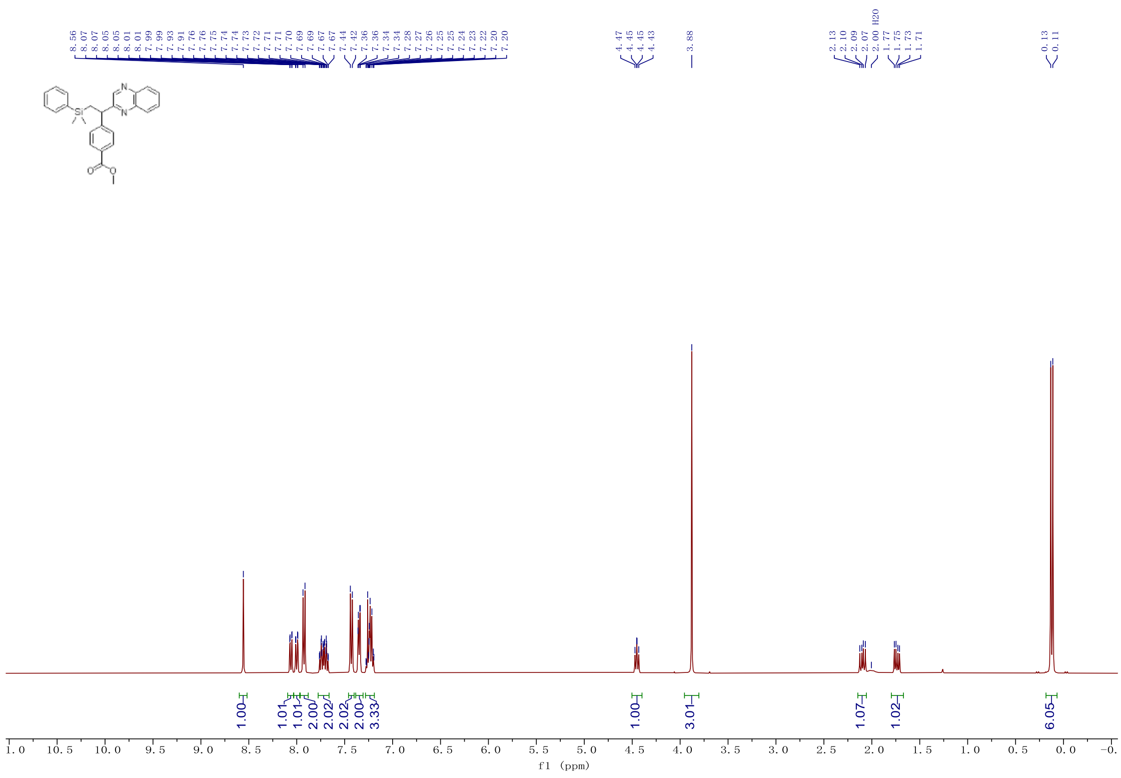

^{13}C NMR spectra of compound **d-88** in $CDCl_3$ (101 MHz): (*see procedure*)

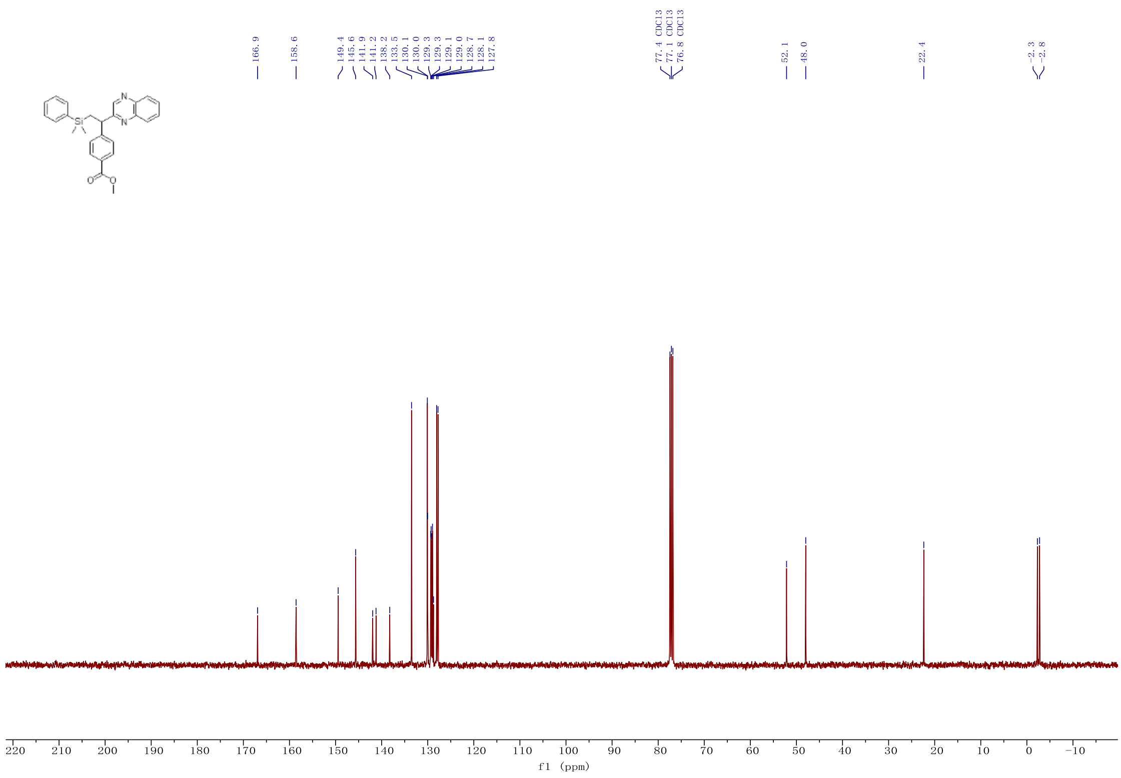

^{1}H NMR spectra of compound **d-89** in $CDCl_3$ (400 MHz): (<u>*see procedure*</u>)

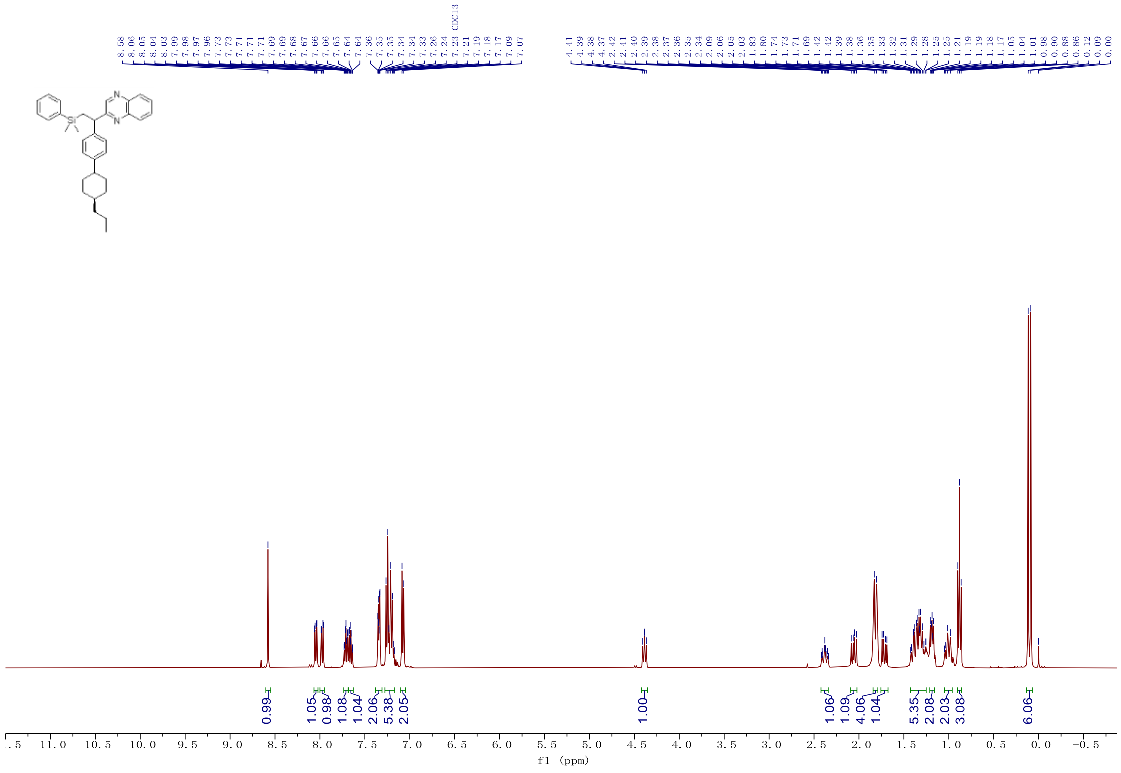

^{13}C NMR spectra of compound **d-89** in $CDCl_3$ (101 MHz): (<u>*see procedure*</u>)

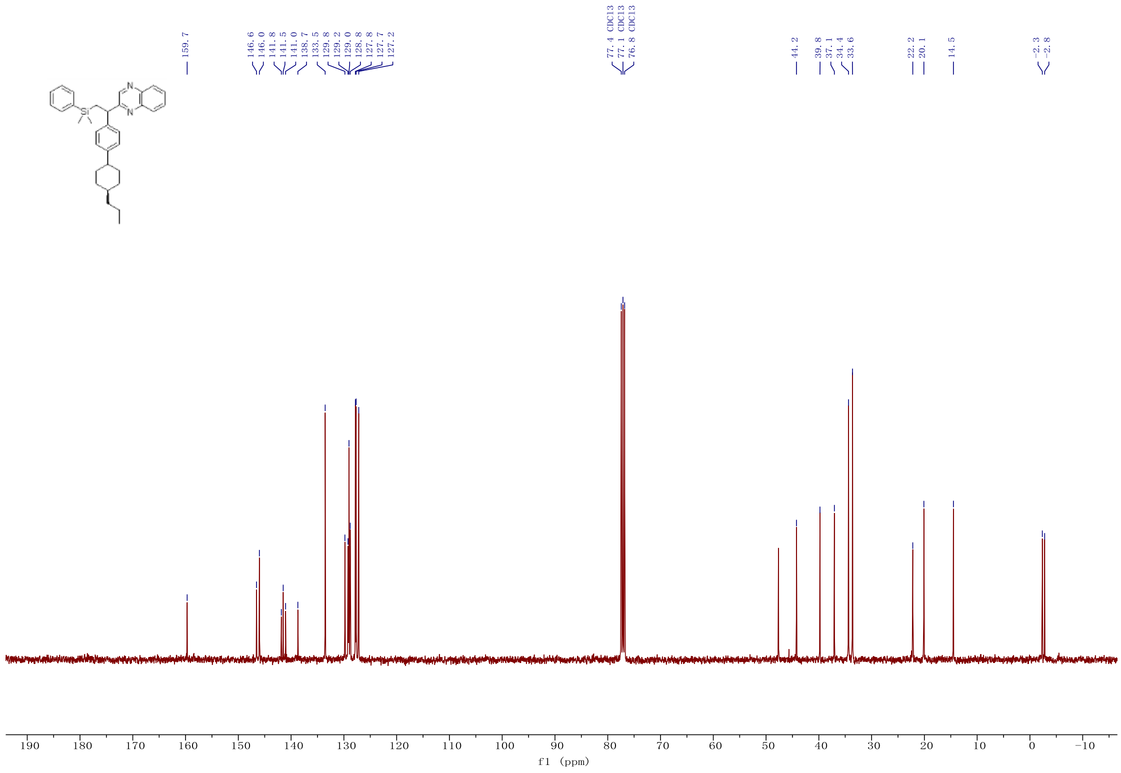

^{1}H NMR spectra of compound **d-90** in $CDCl_3$ (400 MHz): (*see procedure*)

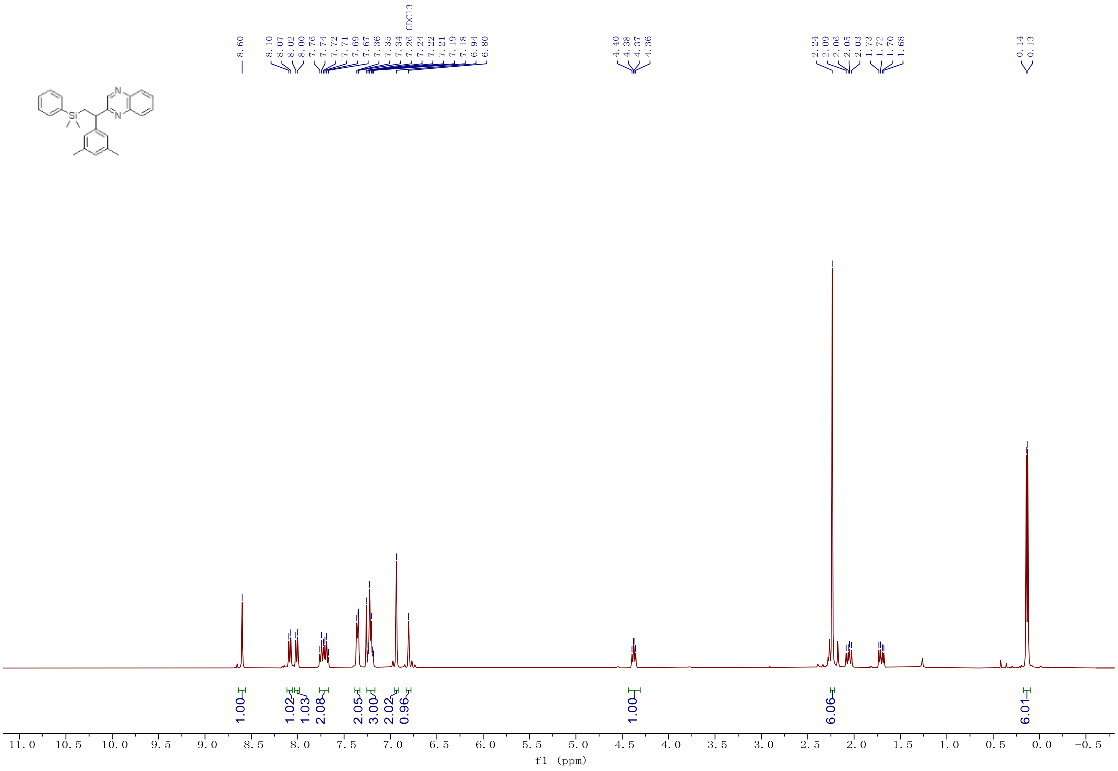

^{13}C NMR spectra of compound **d-90** in $CDCl_3$ (101 MHz): (*see procedure*)

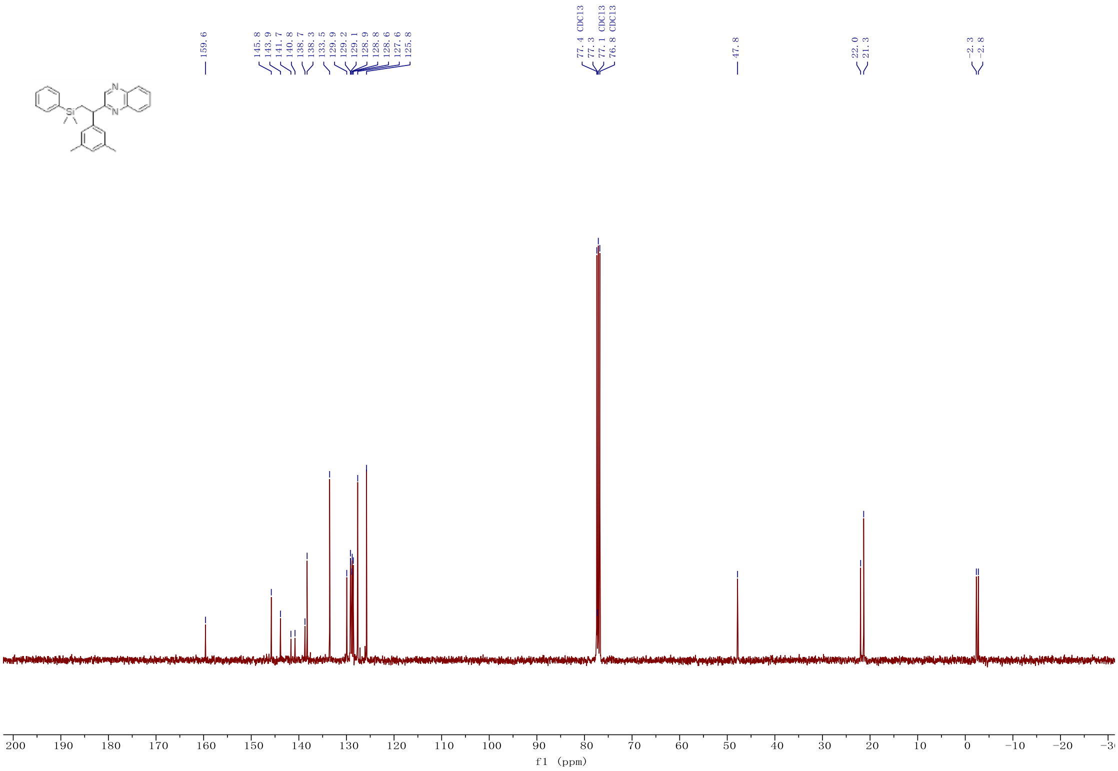

^{1}H NMR spectra of compound **d-91** in $CDCl_3$ (400 MHz): (*see procedure*)

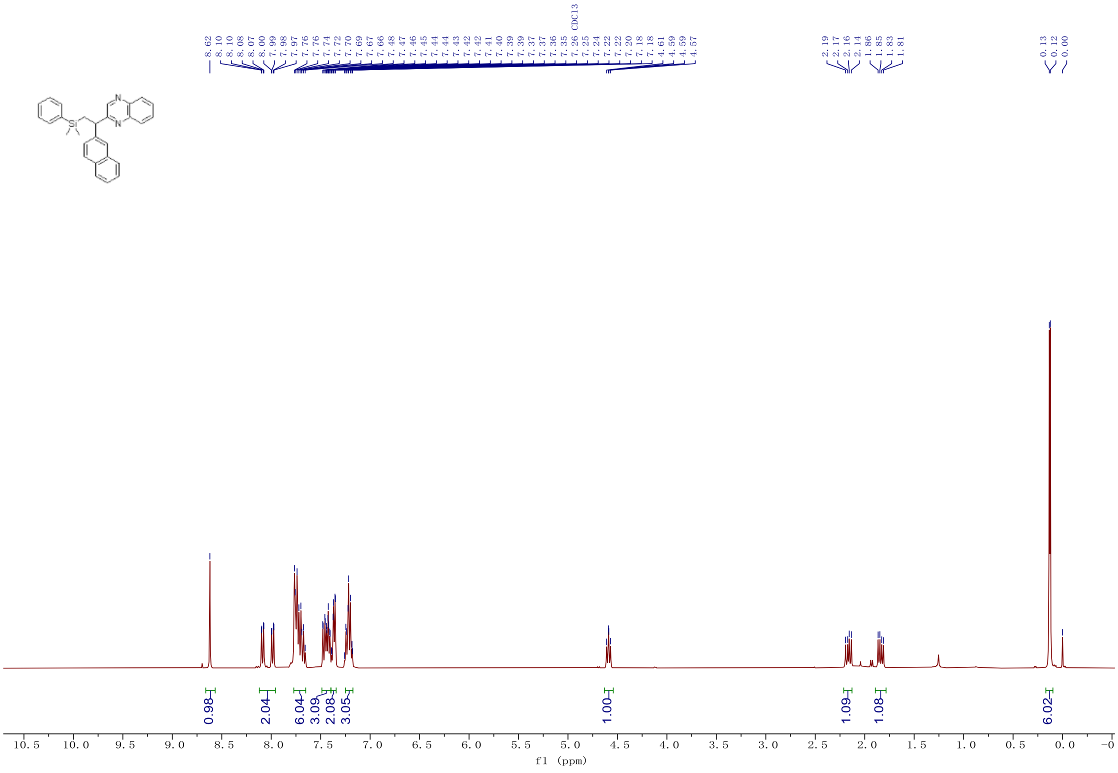

^{13}C NMR spectra of compound **d-91** in $CDCl_3$ (101 MHz): (*see procedure*)

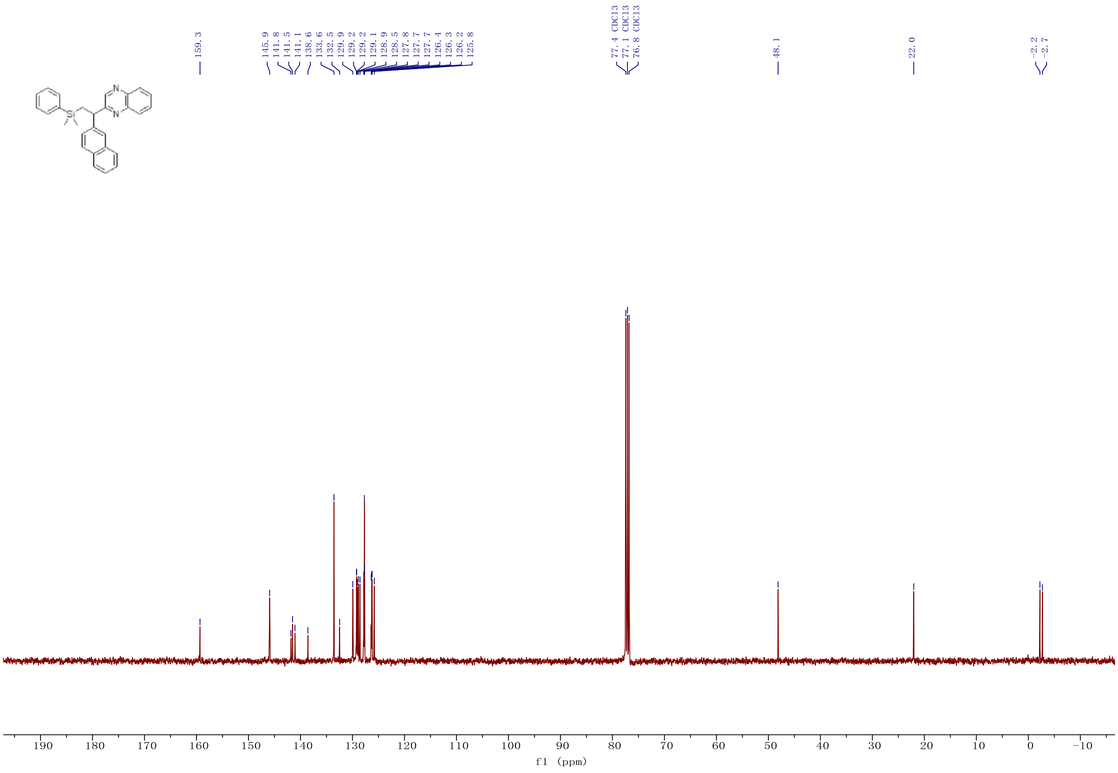

^{1}H NMR spectra of compound **d-92** in $CDCl_3$ (400 MHz): (*see procedure*)

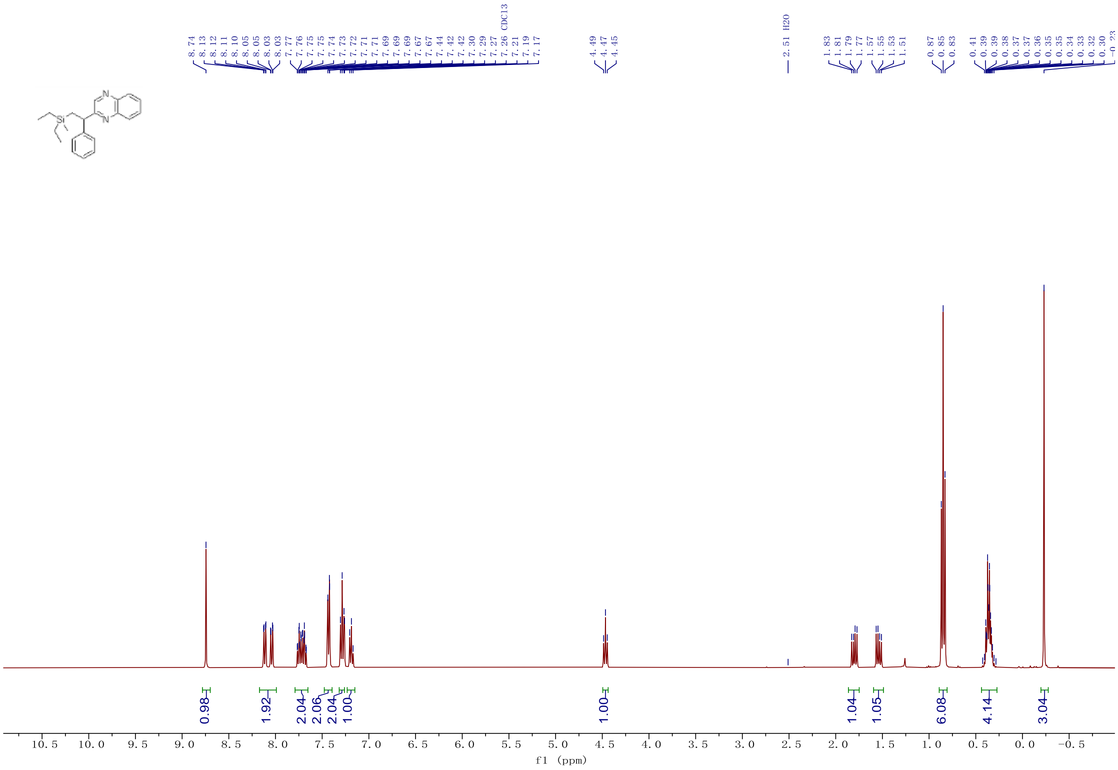

^{13}C NMR spectra of compound **d-92** in $CDCl_3$ (101 MHz): (*see procedure*)

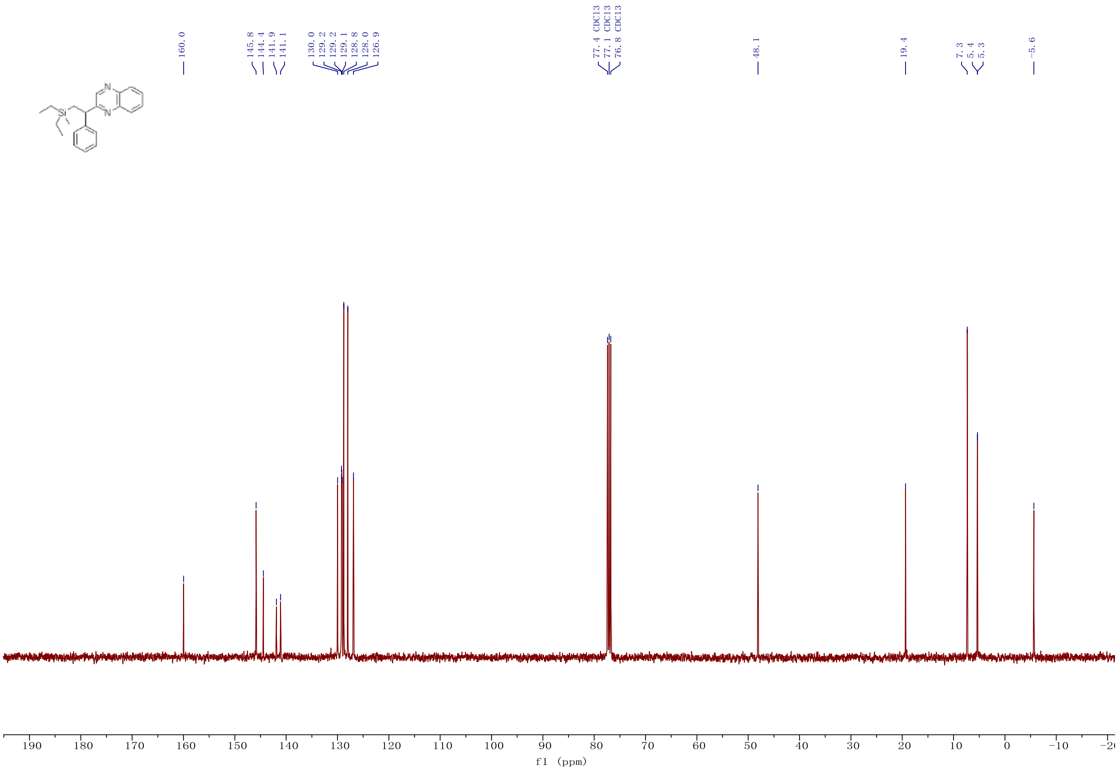

^{1}H NMR spectra of compound **d-93** in $CDCl_3$ (400 MHz): (*see procedure*)

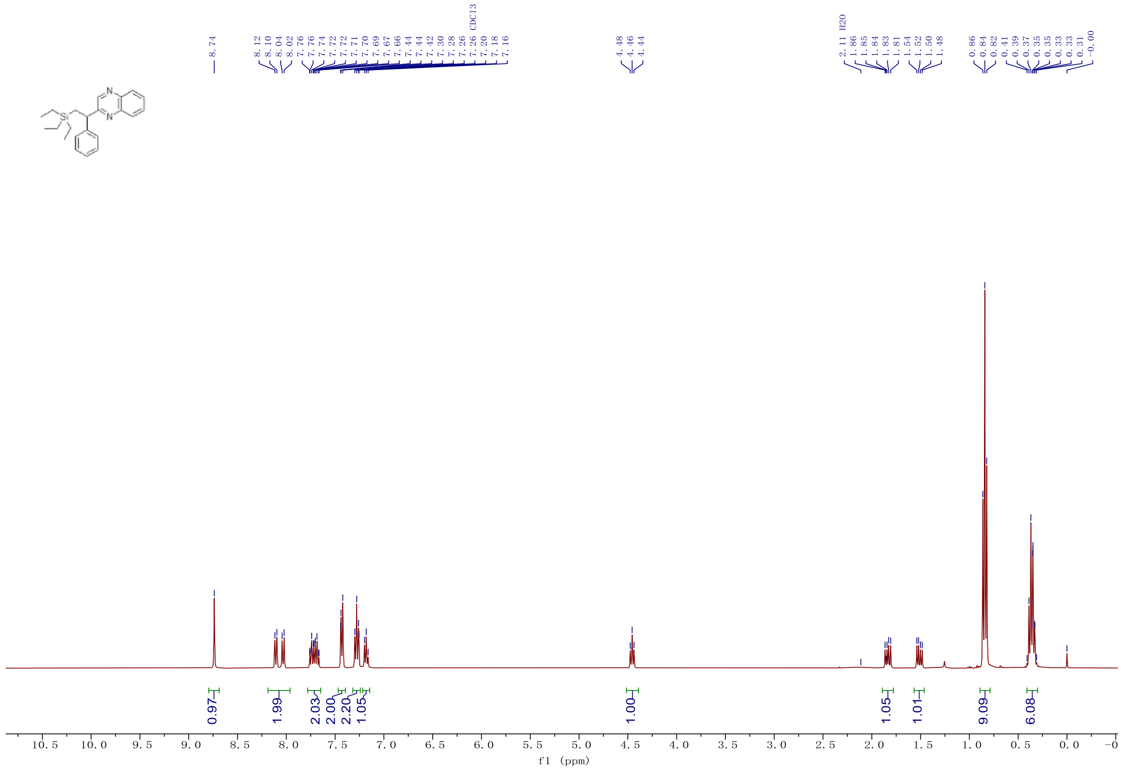

^{13}C NMR spectra of compound **d-93** in $CDCl_3$ (101 MHz): (*see procedure*)

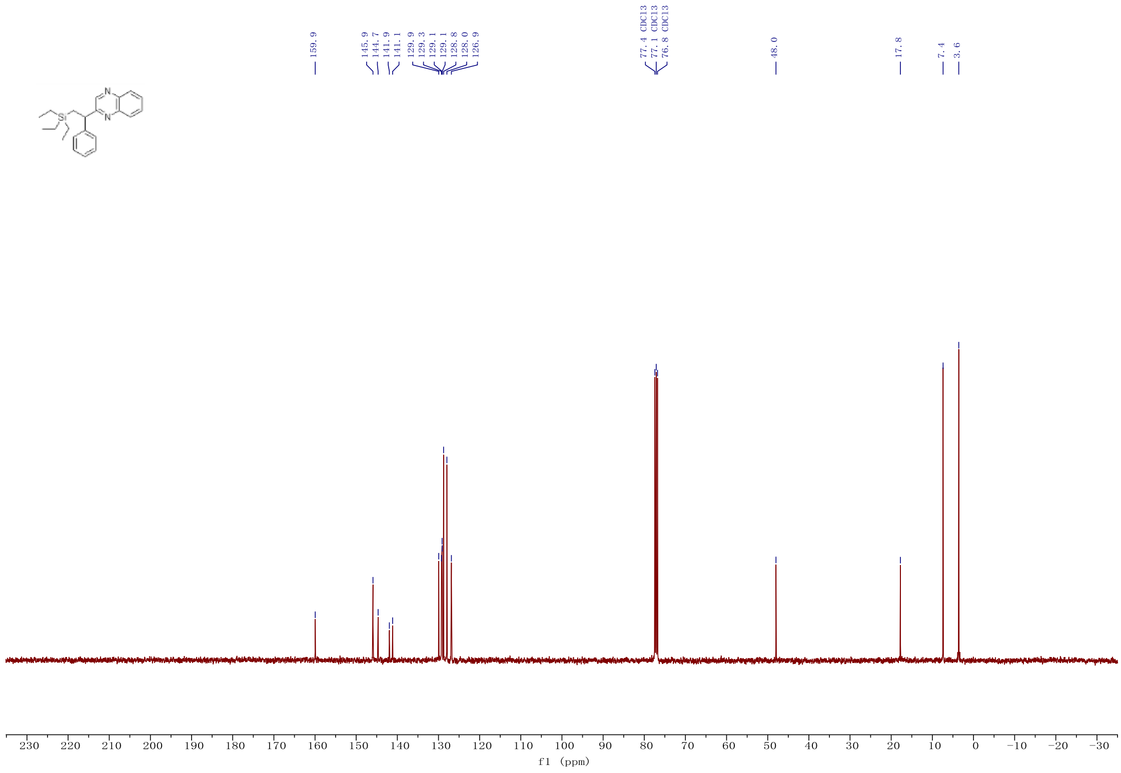

^{1}H NMR spectra of compound **d-94** in $CDCl_3$ (400 MHz): (*see procedure*)

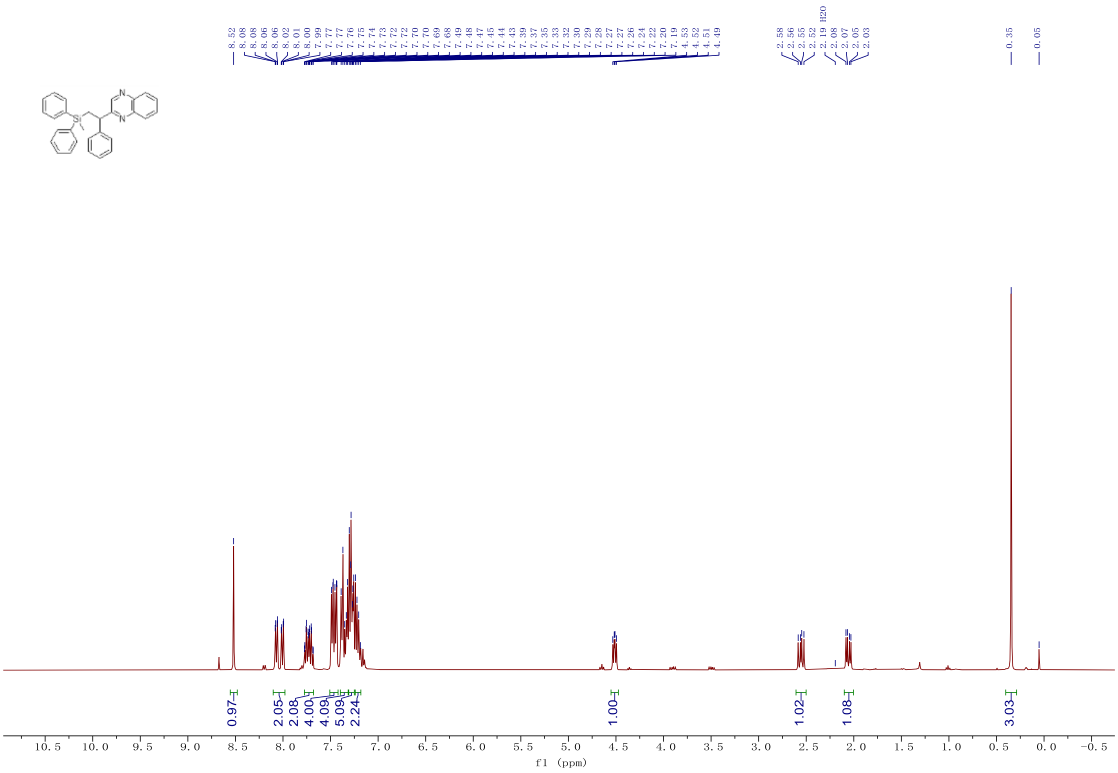

^{13}C NMR spectra of compound **d-94** in $CDCl_3$ (101 MHz): (*see procedure*)

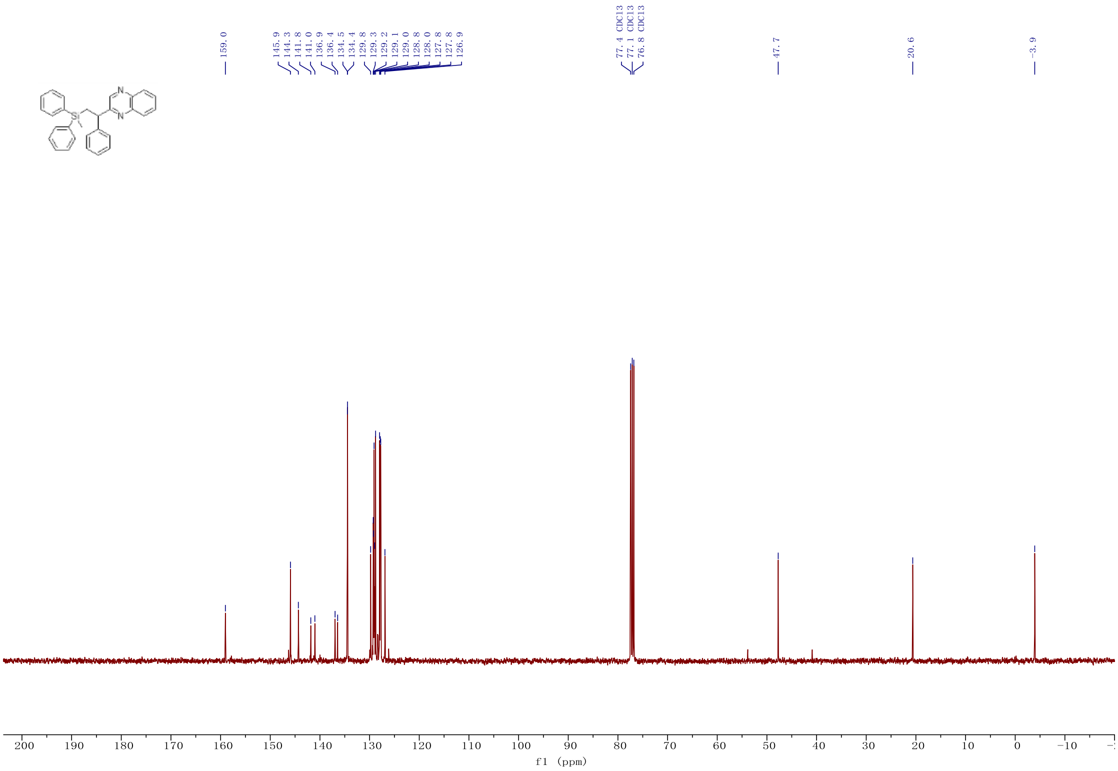

^{1}H NMR spectra of compound **d-95** in $CDCl_3$ (400 MHz): (*see procedure*)

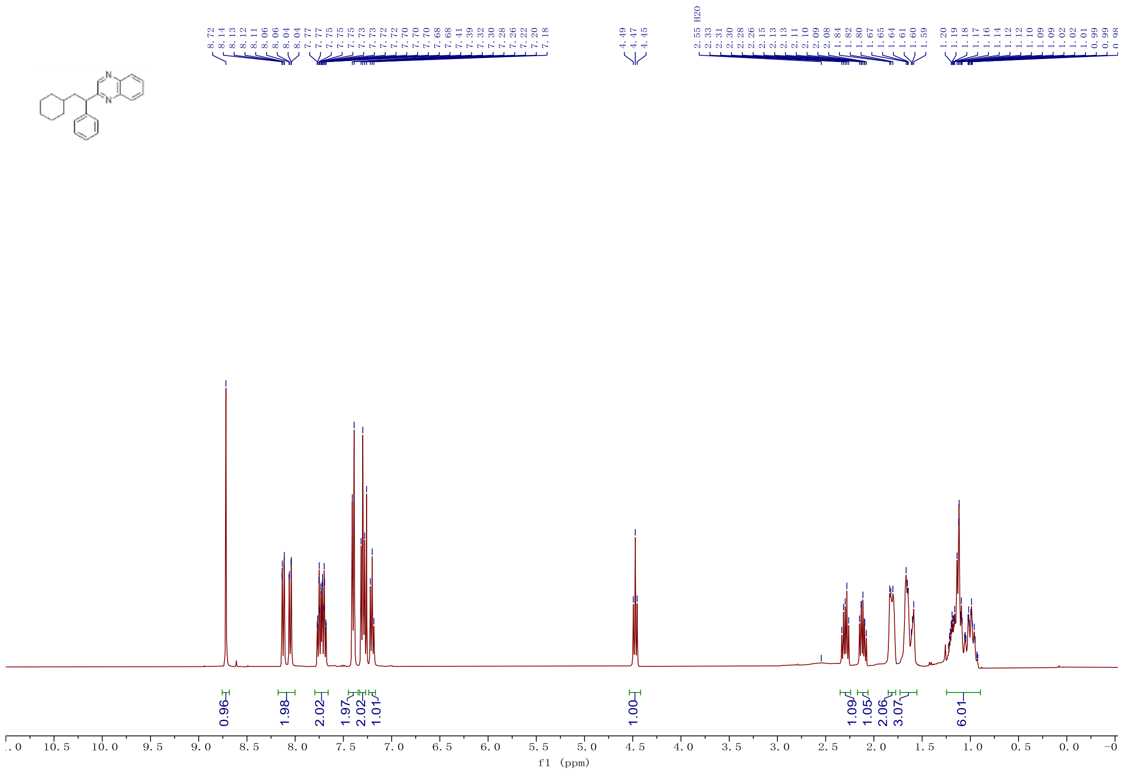

^{13}C NMR spectra of compound **d-95** in $CDCl_3$ (101 MHz): (*see procedure*)

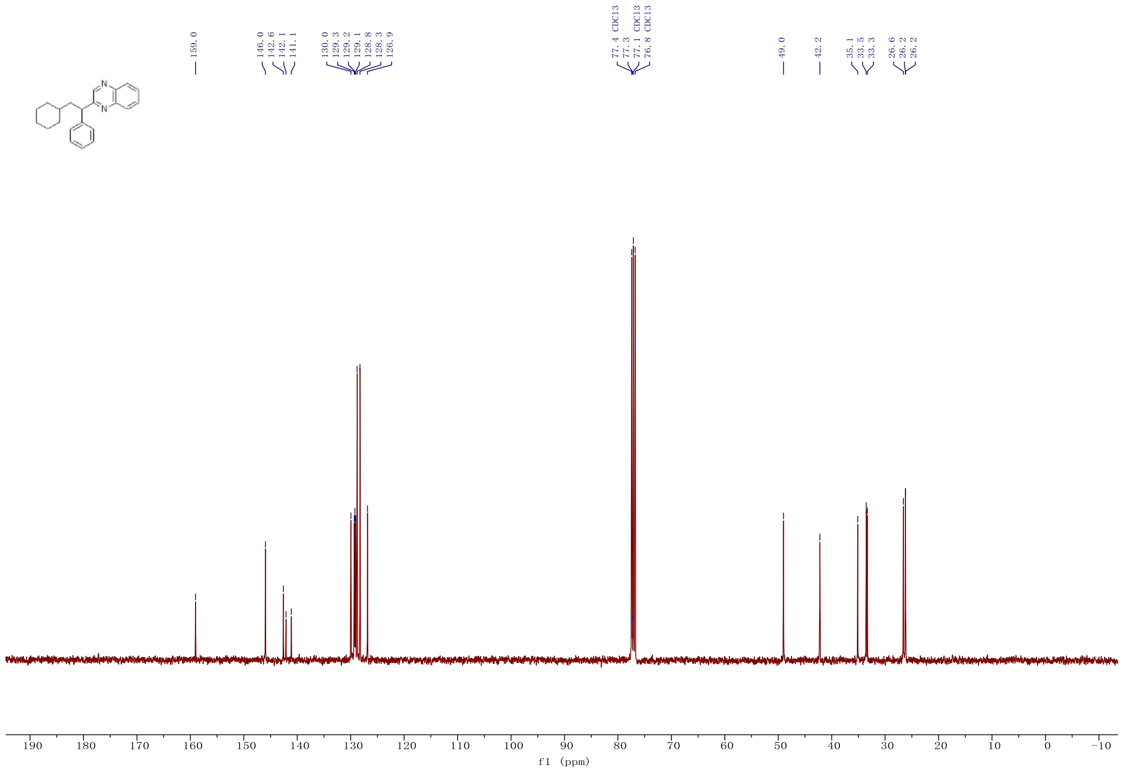

^{1}H NMR spectra of compound **d-96** in $CDCl_3$ (400 MHz): (*see procedure*)

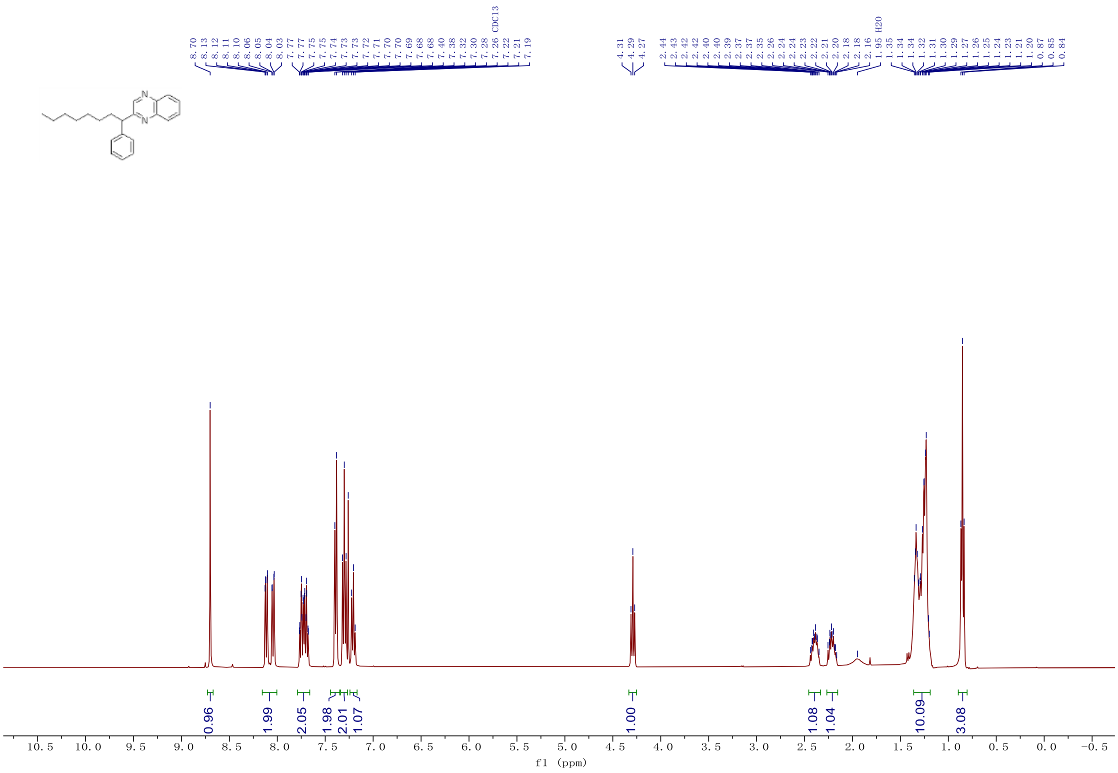

^{13}C NMR spectra of compound **d-96** in $CDCl_3$ (101 MHz): (*see procedure*)

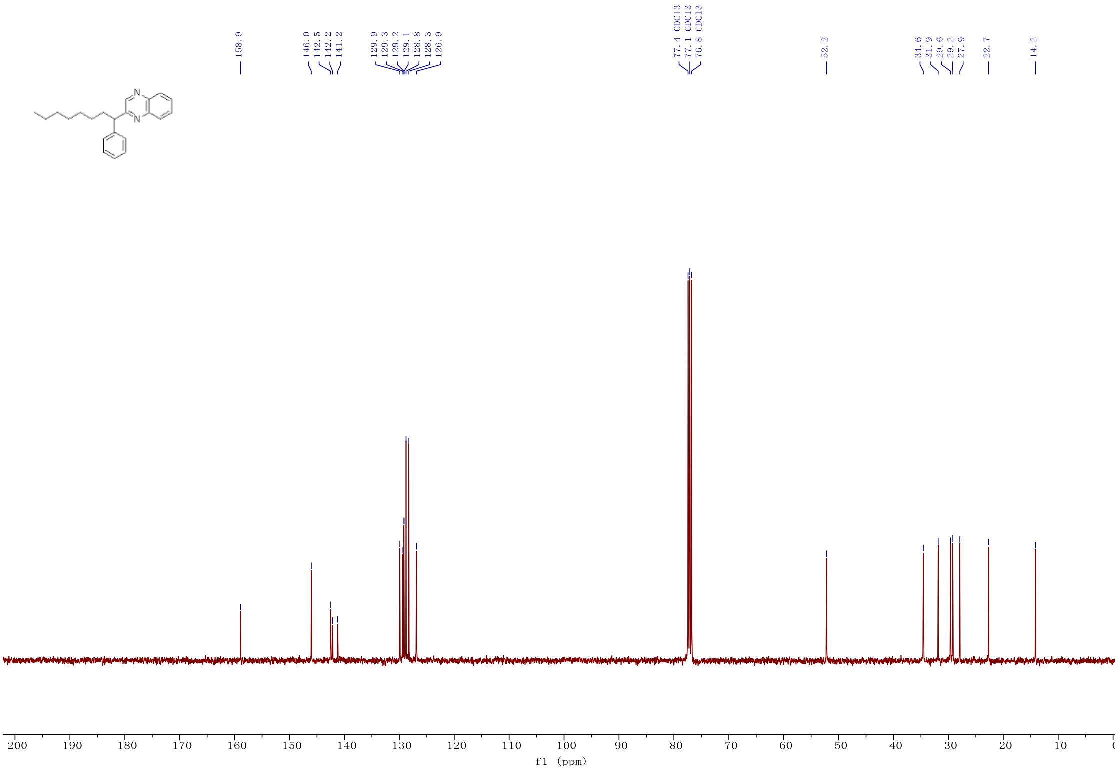

^{1}H NMR spectra of compound **d-97** in $CDCl_3$ (400 MHz): (*see procedure*)

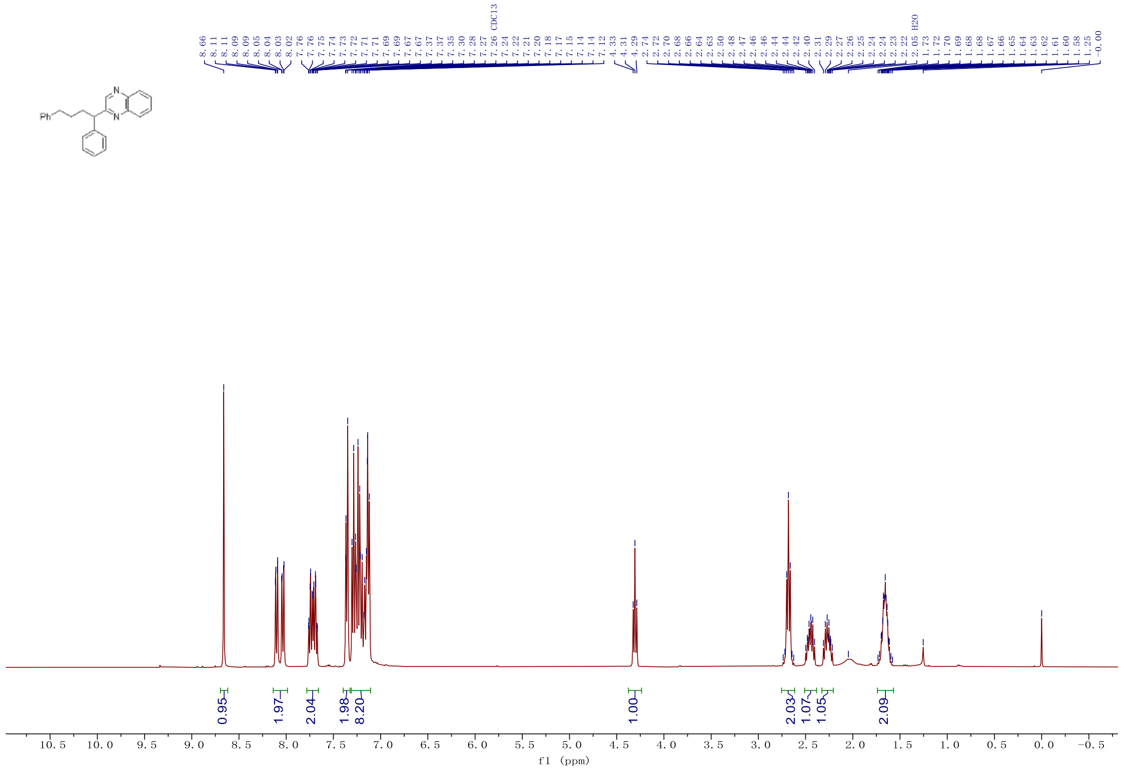

^{13}C NMR spectra of compound **d-97** in $CDCl_3$ (101 MHz): (*see procedure*)

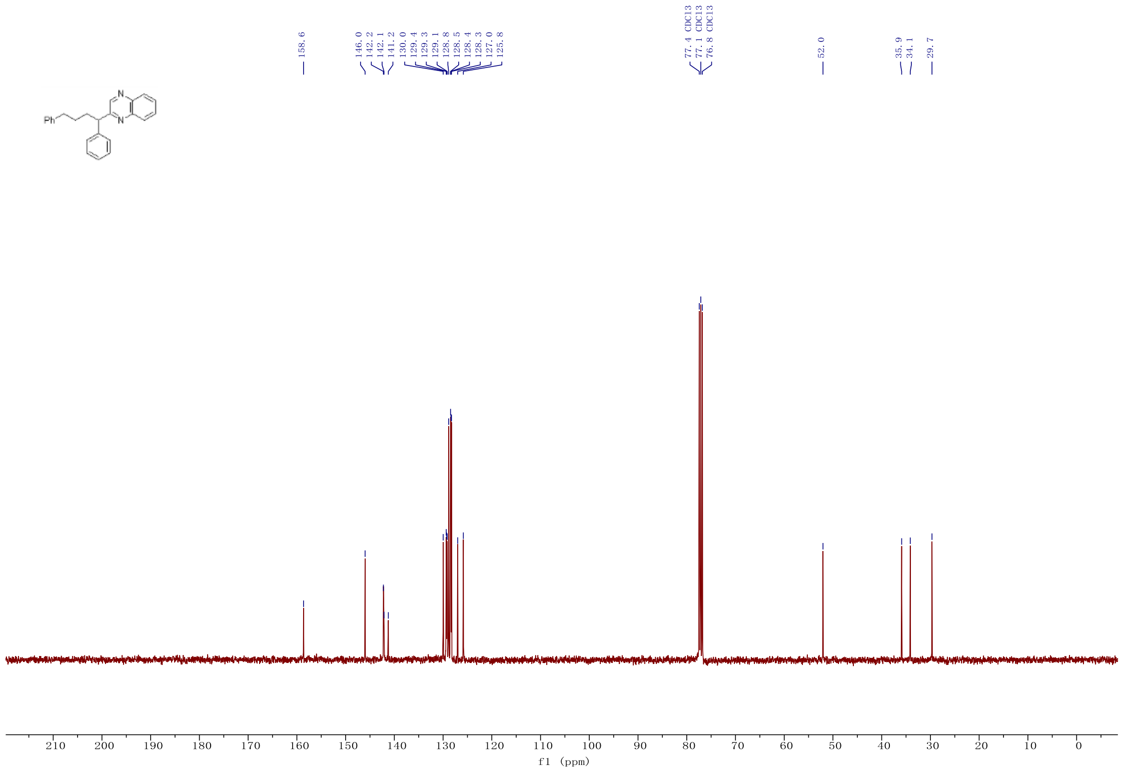

^{1}H NMR spectra of compound **d-98** in $CDCl_3$ (400 MHz): (<u>*see procedure*</u>)

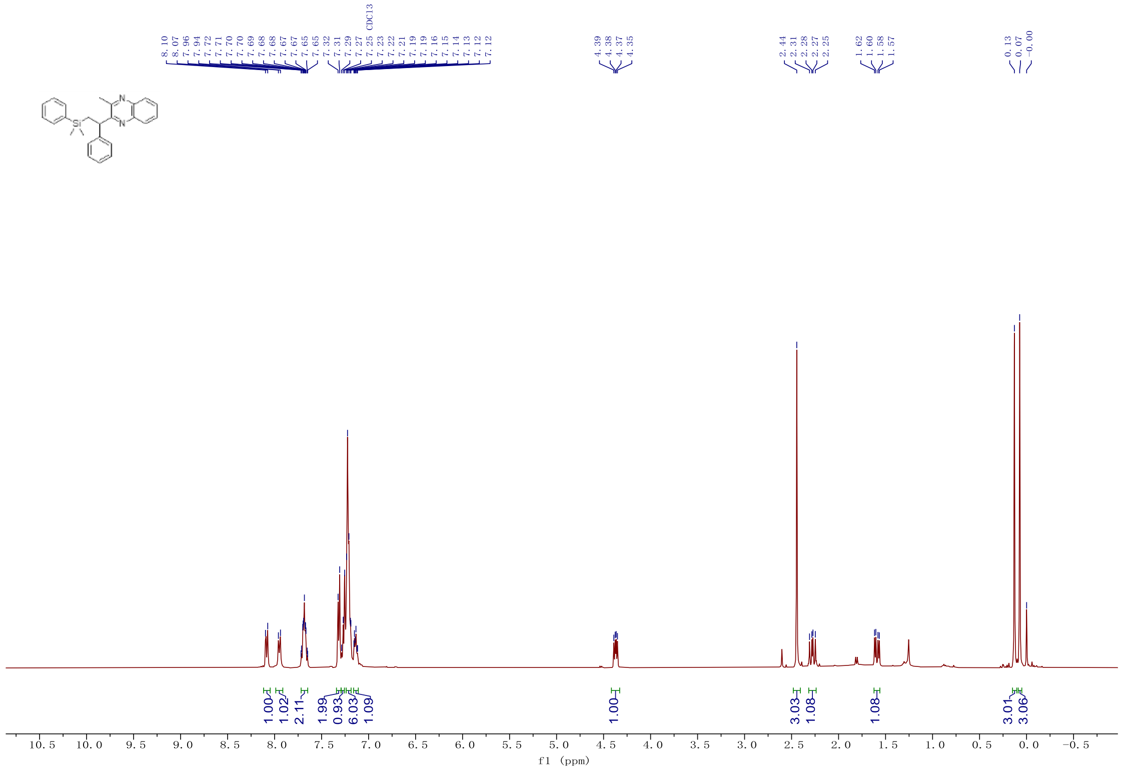

^{13}C NMR spectra of compound **d-98** in $CDCl_3$ (101 MHz): (<u>*see procedure*</u>)

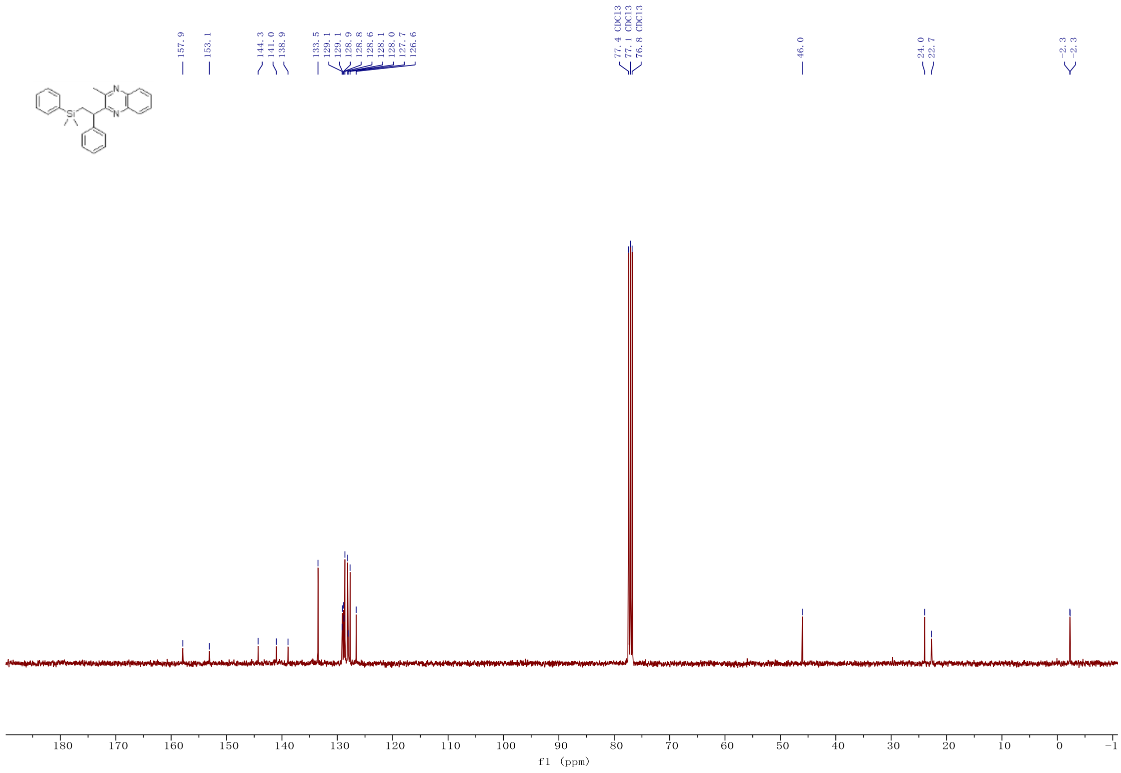

^{1}H NMR spectra of compound **d-99** in $CDCl_3$ (400 MHz): (*see procedure*)

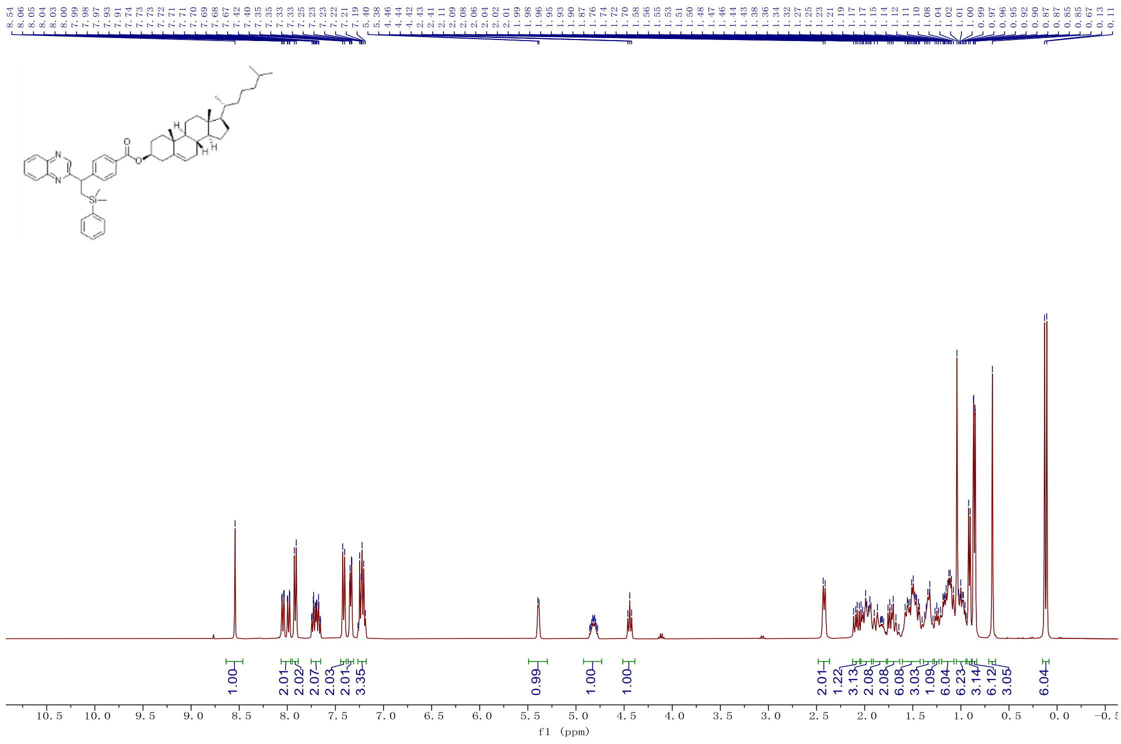

^{13}C NMR spectra of compound **d-99** in $CDCl_3$ (101 MHz): (*see procedure*)

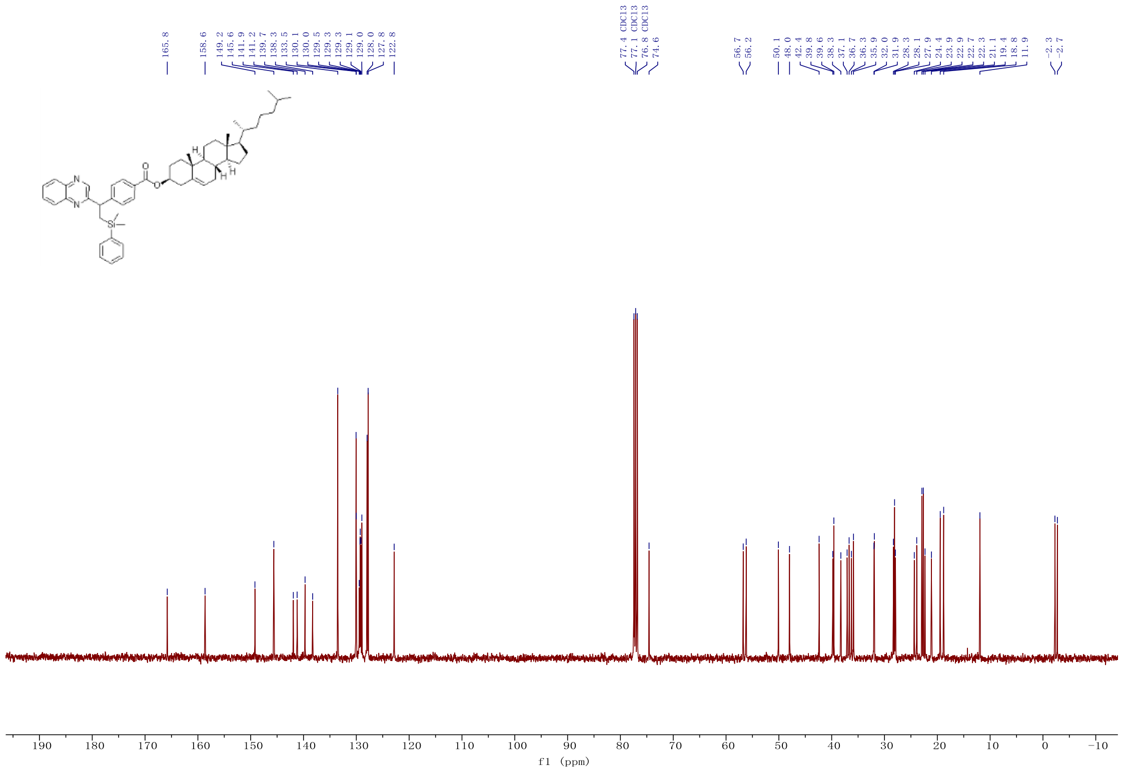

^{1}H NMR spectra of compound **d-100** in $CDCl_3$ (400 MHz): (*see procedure*)

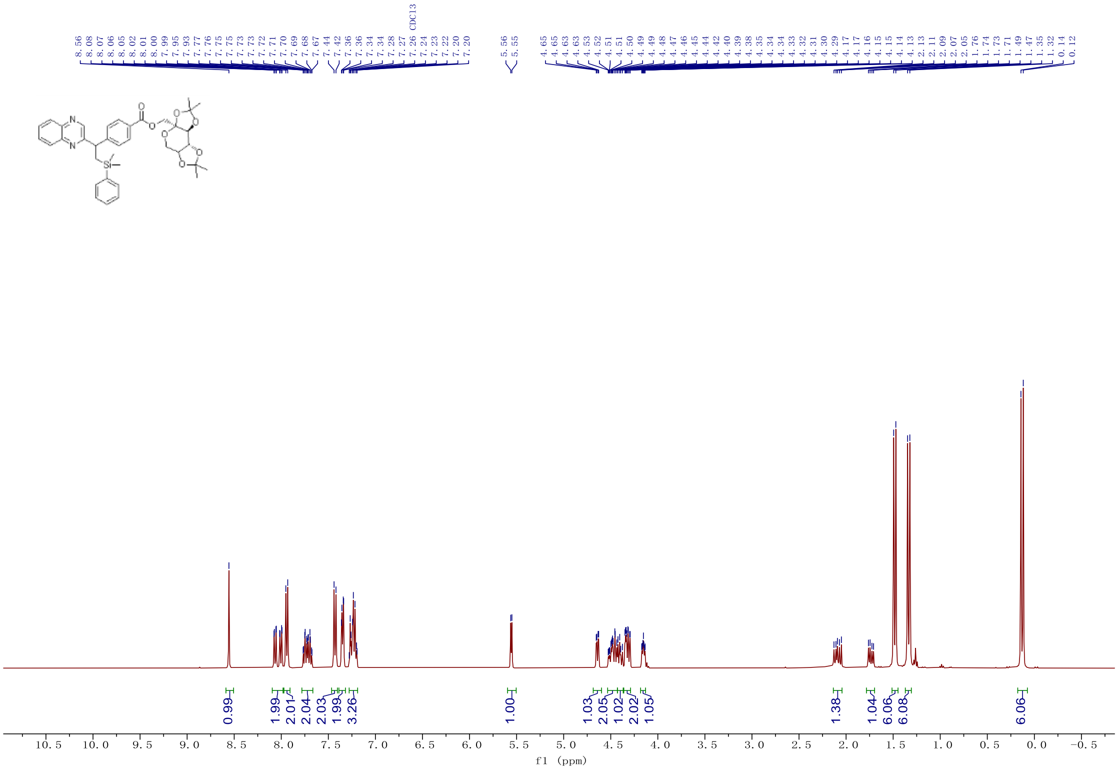

^{13}C NMR spectra of compound **d-100** in $CDCl_3$ (101 MHz): (*see procedure*)

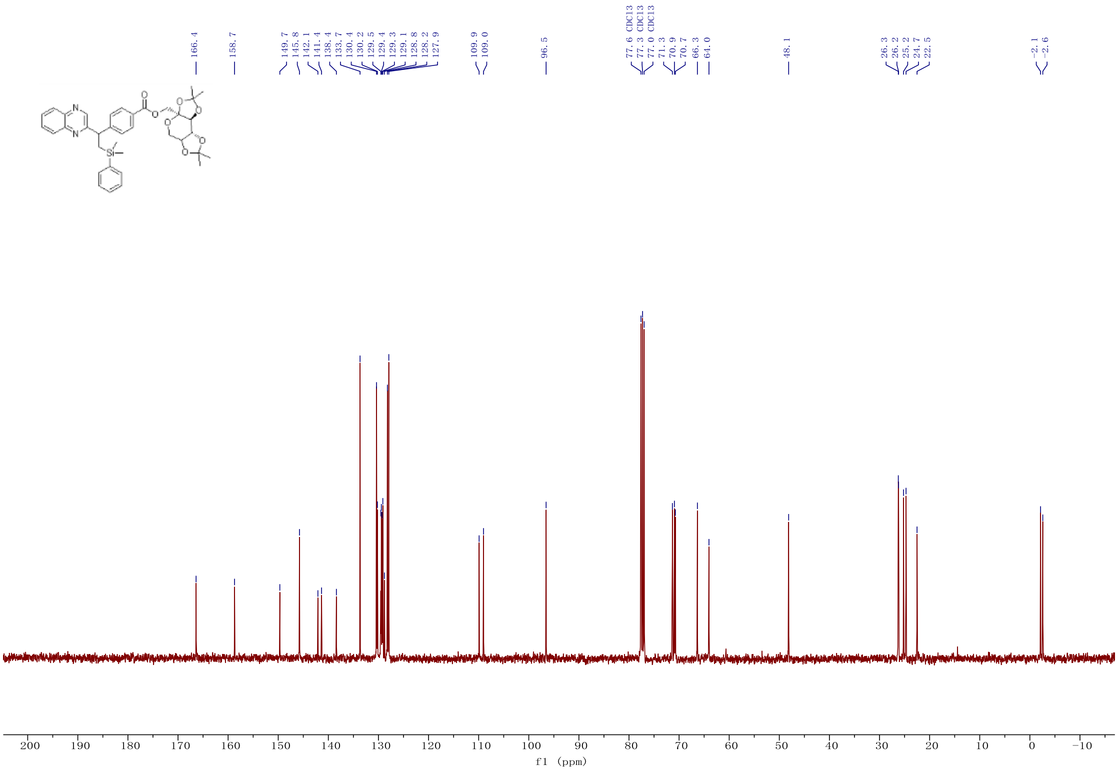

4. References

1. Huang, H.-G., Li, W., Zhong, D., Wang, H.-C., Zhao, J. & Liu, W.-B. Trifluoromethanesulfonyl azide as a bifunctional reagent for metal-free azidotrifluoromethylation of unactivated alkenes. *Chem. Sci.* **12**, 3210–3215 (2021).
2. Simion, A. M., Hashimoto, I., Mitoma, Y., Egashira, N. & Simion, C. O-Acylation of substituted phenols with various alkanoyl chlorides under phase-transfer catalyst conditions. *Synth. Commun.* **42**, 921–931 (2011).
3. Kobayashi, O., Uraguchi, D. & Yamakawa, T. Synthesis of α-trifluoromethylstyrene derivatives via Pd-catalyzed cross-coupling of 2-bromo-3,3,3-trifluoropropene and arylmagnesium bromides. *J. Mol. Catal. A: Chem.* **302**, 7–10 (2009).
4. Kondoh, A., Akahira, S., Oishi, M. & Terada, M. Enantioselective formal [3+2] cycloaddition of epoxides with imines under Brønsted base catalysis: synthesis of 1,3-oxazolidines with quaternary stereogenic center. *Angew. Chem. Int. Ed.* **57**, 6299–6303 (2018).
