# Supplementary material for: Synergistic Fe/Ni catalysis for electrochemical 1,1-difunctionalization of alkenes
Source: Nat Commun. 2026 May 2;17:5961. doi: 10.1038/s41467-026-72546-x (PMC13342334; doi:10.1038/s41467-026-72546-x)
Supplement: Supplementary file 2 — Transparent Peer Review file [file 41467_2026_72546_MOESM2_ESM.pdf]

nature portfolio

Peer Review File

Synergistic Fe/Ni Catalysis for Electrochemical 1,1-Difunctionalization of Alkenes

Corresponding Author: Professor Wujiong Xia

This manuscript has been previously reviewed at another journal. This document only contains information relating to versions considered at Nature Communications.

This file contains all reviewer reports in order by version, followed by all author rebuttals in order by version.

Version 1:

Reviewer comments:

Reviewer #2

(Remarks to the Author)
Xia and co workers report an intriguing electrochemical iron/nickel bimetallic synergistic catalytic system for the challenging 1,1 difunctionalization of alkenes using aryl halides and electron deficient alkenes. The key innovation rests in the elegant design of an electrochemical platform that seamlessly merges the redox properties of nickel with the Lewis acidic character of iron under precise control. By employing stable, readily accessible aryl halides as the aryl source, this strategy effectively circumvents conventional reliance on expensive and sensitive organoboron reagents. Compared with the prior work by Yin et al., this approach represents a notable advance in both sustainability and cost efficiency.

The authors have carefully addressed the reviewers' comments in the revised manuscript and supplymentary information, with particular improvements to the reaction scope, mechanistic elucidation, and overall clarity. The current version satisfies the rigorous standards of Nature Communications, and I therefore recommend its publication without further revision.

Reviewer #3

(Remarks to the Author)
I previously reviewed an earlier version of this manuscript for [redacted], and I am pleased to see that the authors have addressed the major concerns raised by me and other reviewers. The revised manuscript demonstrates substantial improvement and responds effectively to previous critiques. In particular, the expanded substrate scope, the addition of controlled potential electrolysis data, and the elucidation of the iron species' role through combined spectroscopic and electrochemical evidence all strengthen the study. The manuscript in its current form is well-organized, clearly written, and makes a convincing case for the novelty and utility of the synergistic Fe/Ni electrochemical system presented. In my view, it is nearly ready for publication in Nature Communications, pending only the following minor corrections:
1. In Fig. 4, Condition D, only NMP is used as the solvent. Please remove the "v/v = 2:1" notation.
2. In the Supplementary Information, the column header "Enter" in several optimization tables (e.g., Tables S5, S10, S11, S12) should be corrected to "Entry".

Reviewer #4

(Remarks to the Author)
This manuscript by Xia and co-workers presents an innovative electrochemical platform for the intermolecular 1,1-difunctionalization of alkenes with aryl halides and radical acceptors, enabled by an iron–nickel bimetallic system. Compared to Yin's work (ref. 17), this approach replaces expensive and air-sensitive organoboron reagents with readily available aryl halides, thereby improving both the practicality and sustainability of the transformation. The design is elegant and timely, employing electrochemically generated active Ni(I) species in combination with Fe(III) as a Lewis acid to expand the synthetic utility of aryl halides in alkene 1,1-difunctionalization. Particularly noteworthy is the dual role of Fe(III) species in modulating the reduction potential of radical acceptors, thereby preventing their premature reduction, which is a

mechanistic insight of significant value. This methodology offers an efficient and sustainable strategy for the modular construction of complex molecules and holds substantial synthetic potential. Both the manuscript and the supporting information are meticulously prepared, featuring thorough optimization studies, broad substrate scope, and detailed mechanistic investigations. In my view, this work represents a significant advance in the field of electrochemical alkene difunctionalization reactions. Therefore, I recommend publication in Nature Communications after the authors address the following minor points:

1) In the proposed catalytic cycle shown in Fig. 6d, the chain-walking process from intermediate k is depicted in an oversimplified manner. To accurately reflect the mechanism, this step should be explicitly illustrated as proceeding via β-hydride elimination to generate a Ni–H species, followed by hydride migration to complete the aryl migration. Please revise the scheme accordingly.
2) The reactions afford mixtures of diastereomers, typically in ratios of 1.1:1–1.3:1 d.r. Is there any approach to improve the diastereoselectivity?
3) In the Supporting Information, the synthesis of substrate (a-64) mentions the use of “3,5-difluoropicolinic acid.” This is clearly an error. Please verify and correct the reagent name.
4) The caption of Figure S9 in the SI describe as “Gram-scale photoreaction in continuous flow”. However, the experiment described is an electrochemical reaction in batch, not a photochemical one in flow. Please correct this error.

Open Access This Peer Review File is licensed under a Creative Commons Attribution 4.0 International License, which permits use, sharing, adaptation, distribution and reproduction in any medium or format, as long as you give appropriate credit to the original author(s) and the source, provide a link to the Creative Commons license, and indicate if changes were made.
In cases where reviewers are anonymous, credit should be given to 'Anonymous Referee' and the source.
The images or other third party material in this Peer Review File are included in the article’s Creative Commons license, unless indicated otherwise in a credit line to the material. If material is not included in the article’s Creative Commons license and your intended use is not permitted by statutory regulation or exceeds the permitted use, you will need to obtain permission directly from the copyright holder.
To view a copy of this license, visit https://creativecommons.org/licenses/by/4.0/

<u>Point-by-point response</u>

Reviewer #2 (Remarks to the Author)

Xia and co-workers report an intriguing electrochemical iron/nickel bimetallic synergistic catalytic system for the challenging 1,1 difunctionalization of alkenes using aryl halides and electron deficient alkenes. The key innovation rests in the elegant design of an electrochemical platform that seamlessly merges the redox properties of nickel with the Lewis acidic character of iron under precise control. By employing stable, readily accessible aryl halides as the aryl source, this strategy effectively circumvents conventional reliance on expensive and sensitive organoboron reagents. Compared with the prior work by Yin et al., this approach represents a notable advance in both sustainability and cost efficiency.

The authors have carefully addressed the reviewers' comments in the revised manuscript and supplementary information, with particular improvements to the reaction scope, mechanistic elucidation, and overall clarity. The current version satisfies the rigorous standards of Nature Communications, and I therefore recommend its publication without further revision.

<u>Response:</u> Thank you for your positive comments.

Reviewer #3 (Remarks to the Author)

I previously reviewed an earlier version of this manuscript for [redacted], and I am pleas ed to see that the authors have addressed the major concerns raised by me and other rev iewers. The revised manuscript demonstrates substantial improvement and responds eff ectively to previous critiques. In particular, the expanded substrate scope, the addition o f controlled potential electrolysis data, and the elucidation of the iron species' role t hrough combined spectroscopic and electrochemical evidence all strengthen the study. T he manuscript in its current form is well-organized, clearly written, and makes a c onvincing case for the novelty and utility of the synergistic Fe/Ni electrochemical system presented. In my view, it is nearly ready for publication in Nature Communications, pending only the following minor corrections:

1. In Fig. 4, Condition D, only NMP is used as the solvent. Please remove the "v/v = 2:1" notation.

<u>Response:</u> Thank you for this careful observation. We have removed the "v/v = 2:1" notation from **Condition D** in **Fig 4** as suggested. The figure has been updated accordingly in the revised manuscript to accurately reflect that NMP is used as the sole solvent. We appreciate your help in improving the clarity of our work. The updated figure is shown below.

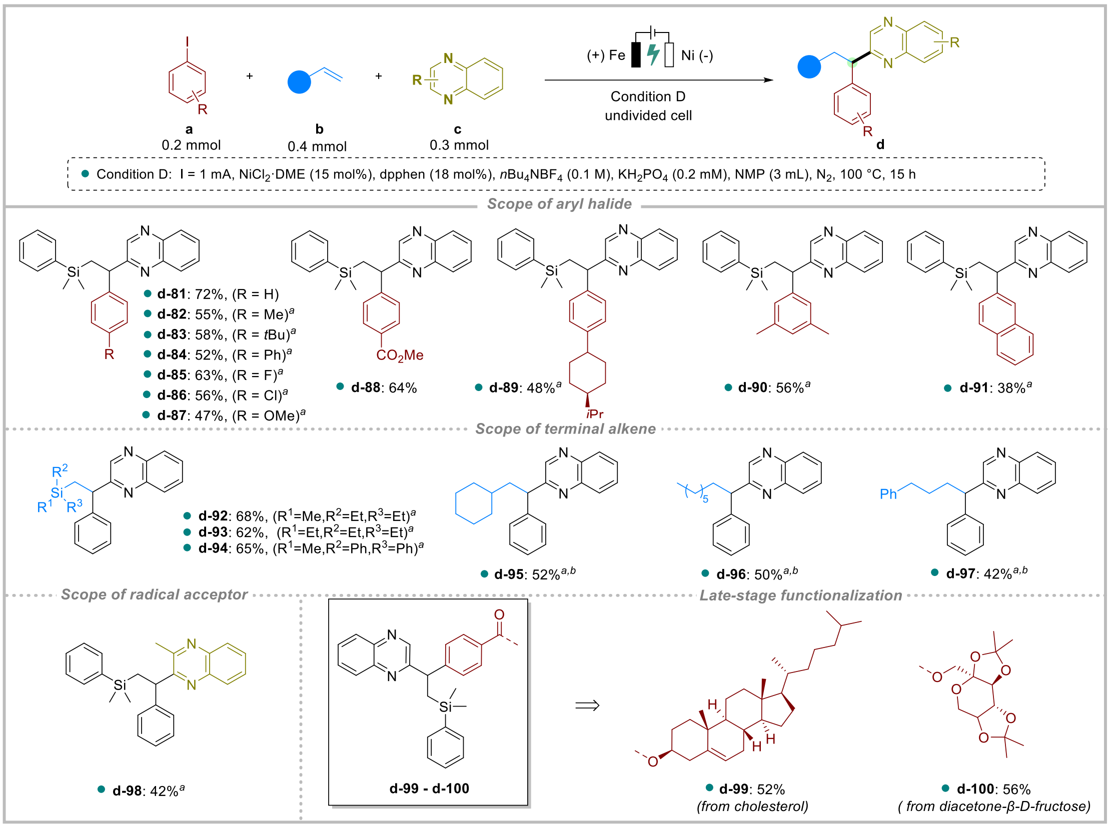

2. In the Supplementary Information, the column header "Enter" in several optimization tables (e.g., Tables S5, S10, S11, S12) should be corrected to "Entry".

Response: Thank you for this careful observation. We have corrected the column header from "Enter" to "Entry" in the indicated optimization tables in the Supplementary Information, including **Tables S5, S10, S11, and S12**, as well as checked other tables for similar typographical errors. The corrections have been made in the revised Supplementary Information file. We appreciate your attention to detail, which has helped improve the accuracy of our manuscript.

Reviewer #4 (Remarks to the Author)

This manuscript by Xia and co-workers presents an innovative electrochemical platform for the intermolecular 1,1-difunctionalization of alkenes with aryl halides and radical acceptors, enabled by an iron–nickel bimetallic system. Compared to Yin's work (ref. 17), this approach replaces expensive and air-sensitive organoboron reagents with readily available aryl halides, thereby improving both the practicality and sustainability of the transformation. The design is elegant and timely, employing electrochemically generated

active Ni(I) species in combination with Fe(III) as a Lewis acid to expand the synthetic utility of aryl halides in alkene 1,1-difunctionalization. Particularly noteworthy is the dual role of Fe(III) species in modulating the reduction potential of radical acceptors, thereby preventing their premature reduction, which is a mechanistic insight of significant value. This methodology offers an efficient and sustainable strategy for the modular construction of complex molecules and holds substantial synthetic potential. Both the manuscript and the supporting information are meticulously prepared, featuring thorough optimization studies, broad substrate scope, and detailed mechanistic investigations. In my view, this work represents a significant advance in the field of electrochemical alkene difunctionalization reactions. Therefore, I recommend publication in Nature Communications after the authors address the following minor points:

1) In the proposed catalytic cycle shown in Fig. 6d, the chain-walking process from intermediate k is depicted in an oversimplified manner. To accurately reflect the mechanism, this step should be explicitly illustrated as proceeding via β-hydride elimination to generate a Ni–H species, followed by hydride migration to complete the aryl migration. Please revise the scheme accordingly.

__Response:__ Thank you for this insightful suggestion. We have revised **Fig 6d** in the manuscript to more accurately depict the chain-walking process from intermediate **k**. As suggested, the updated scheme now explicitly shows: (1) **β-hydride elimination** to generate a Ni–H species, followed by (2) **hydride migration** to complete the aryl migration. This modification provides a clearer and more mechanistically accurate representation of the catalytic cycle. We appreciate your guidance in improving the rigor of our mechanistic discussion. The updated figure is shown below.

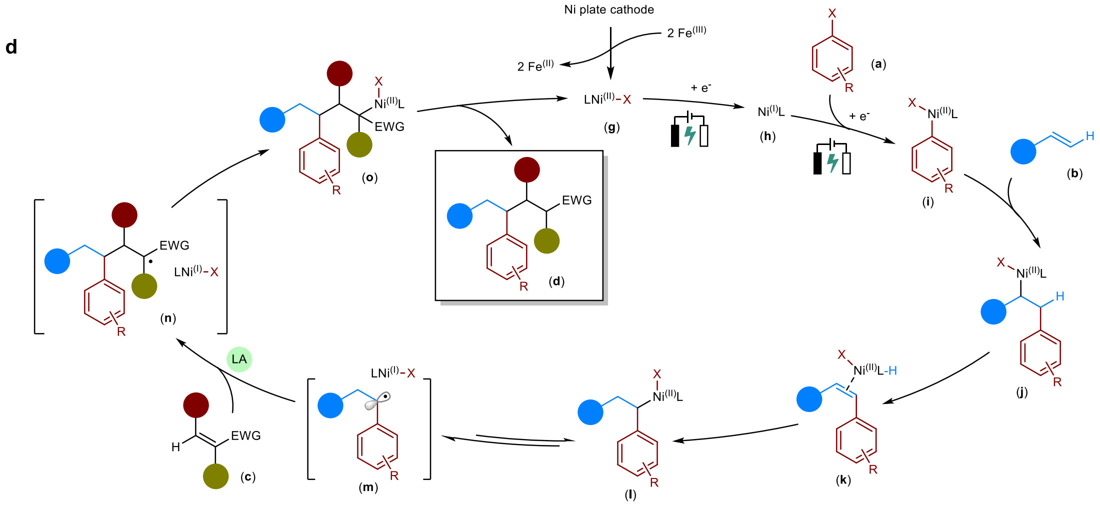

2) The reactions afford mixtures of diastereomers, typically in ratios of 1.1:1 – 1.3:1 d.r. Is there any approach to improve the diastereoselectivity?

Response: We thank the reviewer for raising this important question regarding diastereoselectivity. Notably, during the previous submission to [redacted], a similar concern was raised by another reviewer. Based on that suggestion, we conducted a systematic ligand s creening to investigate the influence of ligand structure on diastereoselectivity. As shown in **Table S11** in the Supplementary Information, a series of bidentate nitrogen and phosph ine ligands were evaluated under standard conditions in an effort to improve the diaster eomeric ratio (d.r.). However, the experimental results showed that diastereoselectivity remain ed within the range of 1.1:1 to 1.3:1 for all ligands tested, with no significant impro vement achieved through ligand modulation.

After in-depth analysis, we believe this outcome can be rationally explained by the stereochemical nature of radical addition: the key step of this reaction involves the addition of a benzyl radical to an electron-deficient alkene, and this benzyl radical possesses a sp^2-hybridized planar structure. It is well established that such carbon-centered radical additions to alkenes are kinetically fast and stereorandom, typically affording *trans* and *cis* isomers in near-statistical ratios. This is the fundamental reason for the observed ~1:1 d.r. values.

We also note that stereocontrol faces the same challenge in pioneering works within the field of alkene 1,1-difunctionalization, as exemplified by the study from Yin and co-workers (*Nat. Catal.* **2023**, *6,* 1030–1041). This comparison indicates that our results are highly consistent with the consensus in the field, indirectly supporting the validity and generality of our proposed mechanism. We again thank the reviewer for this insightful question, which has prompted us to provide this detailed clarification in our response.

Table S11. Ligand-controlled diastereoselectivity and yield

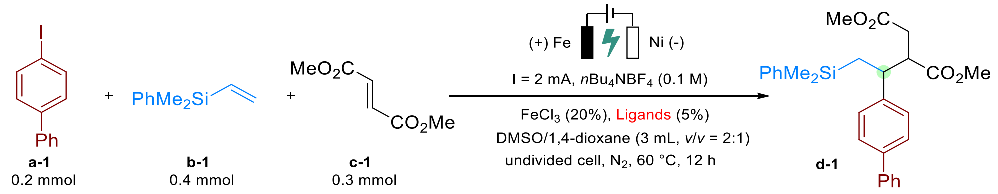

Entry	Ligand	Diastereoselectivity	Yield (%)[a]
**1**	L1	-	trace
**2**	L2	-	trace
**3**	L3	-	N.D.
**4**	L4	-	trace
**5**	L5	-	trace
**6**	L6	-	N.D.
**7**	L7	-	N.D.
**8**	L8	-	trace
**9**	L9	-	N.D.
**10**	L10	-	trace
**11**	L11	1.1:1	12
**12**	L12	-	trace

**13**	L13	1.2:1	13
**14**	L14	1.1:1	23
**15**	L15	-	N.D.
**16**	L16	-	N.D.
**17**	L17	-	trace
**18**	L18	-	N.D.
**19**	L19	-	trace
**20**	L20	-	trace
**21**	L21	-	trace
**22**	L22	1.2:1	20
**23**	L23	1.1:1	25
**24**	PPh_3	1.2:1	32
**25**	P-(Ph-4OMe)$_3$	1.3:1	16

[a] Yield determined by ^{1}H NMR analysis using 1,3,5-trimethoxybenzene as internal standard. N.D. = no detected.

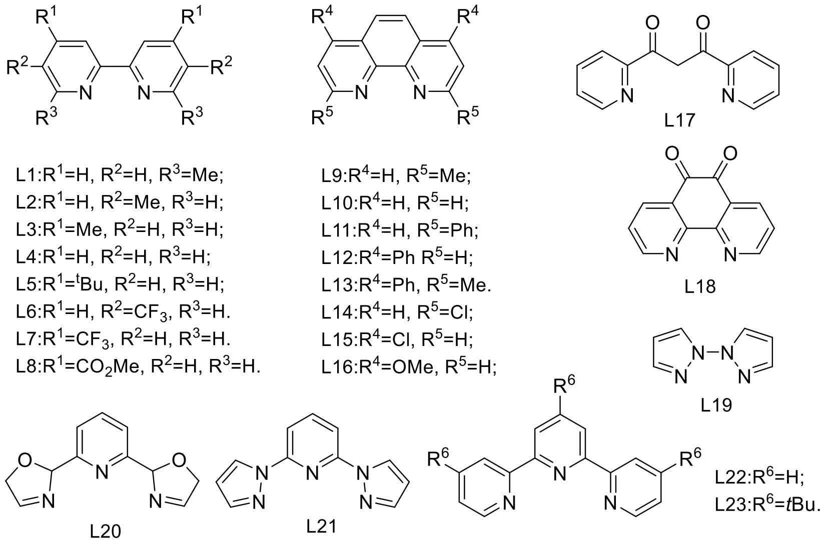

3) In the Supporting Information, the synthesis of substrate (a-64) mentions the use of "3,5-difluoropicolinic acid." This is clearly an error. Please verify and correct the reagent name.

Response: Thank you for identifying this error. We have verified the reagent used in the synthesis of substrate **a-64** and confirm that it is indeed **4-Iodobenzoic acid**, not 3,5-difluoropicolinic acid. The corrected sentence in the Supplementary Information now reads: "**4-Iodobenzoic acid (1240 mg, 5.0 mmol)**." We apologize for this mistake and appreciate your careful review, which has helped improve the accuracy of our Supplementary Information.

4) The caption of Figure S9 in the SI describe as "Gram-scale photoreaction in continuous flow". However, the experiment described is an electrochemical reaction in batch, not a

photochemical one in flow. Please correct this error.

Response: Thank you for pointing out this error. We have corrected the caption of **Figure S9** in the Supplementary Information. The revised caption now reads: "**Figure S9. Setup for gram-scale electrochemical reaction.**" This change accurately reflects that the experiment is an electrochemical reaction performed in batch mode. We apologize for this oversight and appreciate your help in improving the accuracy of our Supplementary Information.
